# Supplementary material for: Targeting a Pleckstrin Homology Domain with a Lysine-Reactive Covalent Binder
Source: J Med Chem. 2026 May 14;69(10):12099–121. doi: 10.1021/acs.jmedchem.5c03818 (PMC13224087; doi:10.1021/acs.jmedchem.5c03818)
Supplement: Supplementary file 1 [file jm5c03818_si_001.pdf]

Supporting Information

Targeting a Pleckstrin Homology Domain with a Lysine-Reactive Covalent Binder

Rebekah M. West,[†,#] Radu Costin Bizga Nicolescu,[†,#] Paul Brear,[‡,#] James Wagstaff,[‡] Beata K. Blaszczyk,[‡] Tomas Deingruber,[†] Matthew G. Sanders,[‖] F. Javier Pérez-Areales,[†] David R. Spring,[†,*] and Marko Hyvönen[‡,**]

[†] Yusuf Hamied Department of Chemistry, University of Cambridge, Cambridge, CB2 1EW, UK
[‡] Department of Biochemistry, University of Cambridge, Cambridge, CB2 1GA, UK
[‖] AstraZeneca, Oncology Targeted Discovery, Oncology R&D, The Discovery Centre, 1 Francis Crick Avenue, Cambridge, CB2 0AA, UK

[*] E-mail: spring@ch.cam.ac.uk
[**] E-mail: mh256@cam.ac.uk

Supporting Information

S1 X-RAY CRYSTALLOGRAPHY S3

S2 COMPOUNDS NMR SPECTRA S14

S3 COMPOUNDS HPLC TRACES S123

S4 MASS SPECTROMETRY STUDIES S143

S4.1 Deconvoluted Spectra for Figure 8A and 8B S143
S4.2 Deconvoluted Spectra for Figure 8C, Reversibility Study S158
S4.3 pH and time dependence of reaction, Figure S80 and Deconvoluted Spectra S164
S4.4 Negative Controls - Deconvoluted Spectra S171
S4.5 Enantiomerically Pure Ligands S174

S5 MALDI-TOF S175

S6 DIFFERENTIAL SCANNING FLUORIMETRY (DSF) STUDIES S176

S6.1 Raw Data for Figure 9 .... S176
S6.2 Supplementary Figures S101A and S101B S177
S6.3 Raw data for Supplementary Figures S101A and S101B S178

S7 STATISTICS S181

S7.1 Normality Tests S181
S7.2 Correlation Tests S182

S8 SEPARATION OF ENANTIOMERS S183

S8.1 Supercritical fluid chromatography S183

S9 COMPUTATIONAL WORK S185

S9.1 Mixed Solvent Molecular Dynamics S185
S9.2 Virtual Screening S185

S1 X-Ray Crystallography

Table S1. Crystal structure data collection, data reduction and refinement statistics.

Protein	BtkS	BtkS	BtkS	BtkS
PDB code	6TUH	7I9I	7I96	9T23
Ligand	**1**	**2**	**4**	**5**
**Data Collection:**				
Beamline	DIAMOND BEAMLINE I24	DIAMOND BEAMLINE i04-1	DIAMOND BEAMLINE i04-1	DIAMOND BEAMLINE i03
Wavelength (Å)	0.9687	0.9763	0.9179	0.9763
Resolution range (Å)	66.720-2.250 (2.38-2.25)	56.08 - 1.83 (1.86- 1.83)	78.67 - 2.52 (2.56 - 2.52)	57.12 - 1.84 (1.88 - 1.84)
Space group	P 1 21 1	P 1 2_1 1	P 1 2_1 1	P 1 2_1 1
Cell (a b c) (Å)	67.76 66.06 80.27	67.95 66.14 79.84	69.10 67.57 79.97	68.89 66.27 79.57
Cell (α β γ) (°)	90.00 100.06 90.00	90.00 100.24 90.00	90.00 100.38 90.00	90.00 101.88 90.00
Total reflections	134635 (20450)	1895375 (89115)	190655 (9769)	415394 (17983)
Unique reflections	32838 (4756)	61602 (3078)	24550 (1217)	60236 (2737)
Multiplicity	4.1 (4.3)	30.8 (29.0)	7.8 (8.0)	6.9 (6.6)
Completeness (%)	98.400 (97.8)	100.0 (100.0)	99.4 (98.5)	99.5 (91.9)
Mean I/sigma(I)	7.0 (1.0)	8.7 (0.2)	8.7 (0.5)	7.1 (0.3)
R-merge	0.121 (0.79)	0.195 (8.65)	0.199 (3.23)	0.145 (3.44)
R-pim	0.067 (0.43)	0.035 (1.62)	0.077 (1.22)	0.060 (1.44)
CC-half	0.987 (0.787)	0.999 (0.45)	0.991 (0.17)	0.994 (0.33)
**Refinement:**				
Refinement resolution range (Å)	66.72- 2.25 (2.32-2.25)	26.53 - 1.83 (1.94 - 1.83)	78.67 - 2.52 (2.54 - 2.52)	33.71 - 1.84 (1.87 - 1.84)
No. reflections	32823 (2812)	50423 (1009)	24507 (491)	58330 (1167)
No. reflections (Rfree)	1598 (135)	2541 (64)	1246 (30)	1943 (91)
R-factor	0.238 (0.2374)	0.242 (0.373)	0.250 (0.403)	0.265 (0.420)
Rfree	0.276 (0.2677)	0.292 (0.423)	0.308 (0.425)	0.319 (0.476)
Number of total atoms	5182	5562	5472	5749
atoms for macromolecules	5036	5307	5352	5552
atoms for ligands	65	79	40	26
atoms for waters	81	176	80	171
Average B-factor (Å^2)	61.62	57.4	73.8	53.7
RMS(bonds) (Å)	0.007	0.009	0.007	0.009
RMS(bond angles) (°)	0.93	1.04	1	1.02
RMS(dihedral angles) (°)	2.70	3.49	3.2	3.51

Protein	BtkA	BtkS	BtkS	BtkS
PDB code	7I90	7I97	7I92	7I98
Ligand	**7**	**8**	**9**	**10**
**Data Collection:**				
Beamline	DIAMOND BEAMLINE i04-1	DIAMOND BEAMLINE i03	DIAMOND BEAMLINE i03	DIAMOND BEAMLINE i03
Wavelength (Å)	0.9762	0.9762	0.9763	0.9763
Resolution range (Å)	56.64 - 1.99 (2.03 - 1.99)	47.11 - 1.95 (1.98 - 1.95)	54.88 - 2.63 (2.68 - 2.63)	57.05 - 2.07 (2.11 - 2.07)
Space group	P 1 2_1 1	P 1 2_1 1	P 1 2_1 1	P 1 2_1 1
Cell (a b c) (Å)	68.96 67.06 79.92	68.94 67.21 79.72	66.31 65.85 78.91	68.98 66.56 79.46
Cell (α β γ) (°)	90.00 100.36 90.00	90.00 100.35 90.00	90.00 99.93 90.00	90.00 101.69 90.00
Total reflections	338150 (16487)	95286 (4578)	139076 (6593)	215394 (10032)
Unique reflections	49146 (2382)	38801 (1939)	19995 (924)	43163 (2112)
Multiplicity	6.9 (6.9)	2.5 (2.4)	7.0 (7.1)	5.0 (4.8)
Completeness (%)	99.9 (97.4)	75.7 (76.4)	99.8 (94.4)	100.0 (99.5)
Mean I/sigma(I)	3.5 (0.1)	5.8 (0.3)	4.3 (0.4)	10.9 (0.5)
R-merge	0.184 (5.77)	0.095 (2.44)	0.420 (3.75)	0.122 (2.47)
R-pim	0.076 (2.35)	0.068 (1.80)	0.171 (1.50)	0.061 (1.28)
CC-half	0.990 (0.33)	0.990 (0.12)	0.978 (0.35)	0.993 (0.24)
**Refinement:**				
Refinement resolution range (Å)	37.02 - 1.99 (2.12 - 1.99)	56.57 - 1.69 (1.71 - 1.69)	54.88 - 2.63 (2.65 - 2.63)	30.12 - 2.07 (2.08 - 2.07)
No. reflections	36799 (736)	77676 (1554)	19946 (416)	42853 (858)
No. reflections (Rfree)	1800 (36)	3802 (75)	967 (25)	2135 (45)
R-factor	0.240 (0.538)	0.249 (0.466)	0.273 (0.450)	0.249 (0.387)
Rfree	0.314 (0.508)	0.291 (0.505)	0.363 (0.472)	0.303 (0.365)
Number of total atoms	5613	5603	5548	5592
atoms for macromolecules	5272	5225	5352	5296
atoms for ligands	83	83	100	88
atoms for waters	258	295	96	208
Average B-factor ($Å^2$)	71.5	44.5	70.1	58.9
RMS(bonds) (Å)	0.009	0.011	0.007	0.008
RMS(bond angles) (°)	1.2	1.25	0.98	1.02
RMS(dihedral angles) (°)	3.06	3.73	3.14	3.48

Protein	BtkS	BtkS	BtkS	BtkS
PDB code	9T3M	9T3G	7I91	7I9D
Ligand	**11**	**12**	**13**	**14**
**Data Collection:**				
Beamline	DIAMOND BEAMLINE i03	DIAMOND BEAMLINE i04	DIAMOND BEAMLINE i03	DIAMOND BEAMLINE i03
Wavelength (Å)	0.9763	0.9762	0.9763	0.9763
Resolution range (Å)	67.60 - 1.80 (1.83 - 1.80)	47.11 - 1.95 (1.98 - 1.95)	57.05 - 1.93 (1.96 - 1.93)	67.23 - 1.80 (1.83 - 1.80)
Space group	$P\ 1\ 2_1\ 1$	$P\ 1\ 2_1\ 1$	$P\ 1\ 2_1\ 1$	$P\ 1\ 2_1\ 1$
Cell (a b c) (Å)	68.59 67.07 79.93	68.39 66.23 79.56	68.83 66.42 79.59	68.52 66.60 79.63
Cell (α β γ) (°)	90.00 99.77 90.00	90.00 101.46 90.00	90.00 101.76 90.00	90.00 101.09 90.00
Total reflections	464122 (23522)	95286 (4578)	2560839 (126031)	452977 (22089)
Unique reflections	66443 (3312)	38801 (1939)	52937 (2572)	65293 (3144)
Multiplicity	7.0 (7.1)	2.5 (2.4)	48.4 (49.0)	6.9 (7.0)
Completeness (%)	100.0 (99.4)	75.7 (76.4)	99.9 (96.9)	99.9 (98.2)
Mean I/sigma(I)	9.0 (0.2)	5.8 (0.3)	5.3 (0.2)	15.8 (0.3)
R-merge	0.130 (4.41)	0.095 (2.44)	0.421 (9.99)	0.066 (3.27)
R-pim	0.053 (1.76)	0.068 (1.80)	0.061 (1.57)	0.027 (1.32)
CC-half	0.998 (0.27)	0.990 (0.12)	0.997 (0.36)	0.999 (0.34)
**Refinement:**				
Refinement resolution range (Å)	67.60 - 1.80 (1.82 - 1.80)	47.11 - 1.95 (1.97 - 1.95)	21.94 - 1.93 (1.97 - 1.93)	67.23 - 1.80 (1.81 - 1.80)
No. reflections	65010 (1301)	36201 (725)	49641 (993)	64777 (1296)
No. reflections (Rfree)	3277 (154)	1724 (81)	2502 (45)	3271 (65)
R-factor	0.245 (0.570)	0.261 (0.477)	0.255 (0.572)	0.245 (0.442)
Rfree	0.298 (0.566)	0.334 (0.591)	0.322 (0.542)	0.297 (0.449)
Number of total atoms	5471	5265	5546	5544
atoms for macromolecules	5113	5090	5289	5274
atoms for ligands	47	77	57	65
atoms for waters	311	98	200	205
Average B-factor (Å^2)	47.1	57.1	62.2	56.1
RMS(bonds) (Å)	0.009	0.009	0.009	0.009
RMS(bond angles) (°)	1.05	1.07	1.03	1.01
RMS(dihedral angles) (°)	3.73	3.44	3.57	3.76

Protein	Btk	BtkS	BtkWT	BtkS
PDB code	7I9F	9T1V	7I9E	9T0T
Ligand	**16**	**17**	**18**	**19**
**Data Collection:**				
Beamline	DIAMOND BEAMLINE i03	DIAMOND BEAMLINE i03	DIAMOND BEAMLINE i03	DIAMOND BEAMLINE i03
Wavelength (Å)	0.9763	0.9763	0.9763	0.9184
Resolution range (Å)	56.14 - 2.04 (2.07 - 2.04)	47.41 - 1.95 (1.98 - 1.95)	56.41 - 1.91 (1.94 - 1.91)	50.93 - 1.73 (1.76 - 1.73)
Space group	$P\ 1\ 2_1\ 1$	$P\ 1\ 2_1\ 1$	$P\ 1\ 2_1\ 1$	$P\ 1\ 2_1\ 1$
Cell (a b c) (Å)	68.58 67.63 79.53	66.82 66.28 79.26	68.75 67.32 79.56	68.99 66.66 79.86
Cell (α β γ) (°)	90.00 99.83 90.00	90.00 99.95 90.00	90.00 100.28 90.00	90.00 98.68 90.00
Total reflections	320897 (14914)	95286 (4769)	388145 (19500)	523123 (26377)
Unique reflections	45711 (2213)	38802 (1939)	55583 (2738)	74878 (3698)
Multiplicity	7.0 (6.7)	2.5 (2.4)	7.0 (7.1)	7.0 (7.1)
Completeness (%)	99.7 (97.8)	75.7 (76.0)	99.9 (98.5)	100.0 (99.7)
Mean I/sigma(I)	8.2 (0.4)	5.8 (0.3)	7.6 (0.2)	13.2 (0.2)
R-merge	0.172 (6.63)	0.095 (2.43)	0.147 (5.29)	0.080 (4.95)
R-pim	0.070 (2.77)	0.068 (1.81)	0.060 (2.12)	0.033 (1.99)
CC-half	0.997 (0.34)	0.99 (0.12)	0.998 (0.27)	0.998 (0.32)
**Refinement:**				
Refinement resolution range (Å)	56.14 - 2.04 (2.06 - 2.04)	55.24 - 2.41 (2.43 - 2.41)	56.41 - 1.91 (1.96 - 1.91)	24.66 - 1.73 (1.76 - 1.73)
No. reflections	43795 (876)	26468 (530)	51914 (1039)	71430 (1429)
No. reflections (Rfree)	2165 (41)	1351 (63)	2602 (50)	1970 (92)
R-factor	0.263 (0.450)	0.263 (0.433)	0.266 (0.530)	0.248 (0.513)
Rfree	0.323 (0.420)	0.329 (0.454)	0.318 (0.540)	0.301 (0.560)
Number of total atoms	5591	5535	5579	5981
atoms for macromolecules	5352	5316	5344	5561
atoms for ligands	84	80	66	128
atoms for waters	155	139	169	292
Average B-factor ($Å^2$)	73.3	61.2	63.3	55.3
RMS(bonds) (Å)	0.008	0.007	0.009	0.009
RMS(bond angles) (°)	1.04	0.98	1.01	1.03
RMS(dihedral angles) (°)	3.46	2.99	3.54	3.58

Protein	Btk	Btk	Btk	BtkA
PDB code	7I99	9RL9	7I9H	7I95
Ligand	**22**	**24**	**26**	**25**
**Data Collection:**				
Beamline	DIAMOND BEAMLINE i03	DIAMOND BEAMLINE i03	DIAMOND BEAMLINE i04	DIAMOND BEAMLINE i03
Wavelength (Å)	0.9763	0.9763	0.9537	0.9763
Resolution range (Å)	56.37 - 1.97 (2.00 - 1.97)	56.24 - 2.11 (2.15 - 2.11)	56.38 - 2.59 (2.63 - 2.59)	56.32 - 1.99 (2.02 - 1.99)
Space group	P 1 2_1 1	P 1 2_1 1	P 1 2_1 1	P 1 2_1 1
Cell (a b c) (Å)	68.51 67.51 79.46	68.34 66.38 79.70	68.26 66.72 79.62	67.96 66.11 79.63
Cell (α β γ) (°)	90.00 100.55 90.00	90.00 100.25 90.00	90.00 100.76 90.00	90.00 100.97 90.00
Total reflections	352128 (18044)	284078 (14307)	153878 (7760)	332943 (16493)
Unique reflections	50397 (2504)	40587 (1998)	22122 (1107)	47744 (2319)
Multiplicity	7.0 (7.2)	7.0 (7.2)	7.0 (7.0)	7.0 (7.1)
Completeness (%)	99.6 (98.3)	99.9 (99.3)	100.0 (99.1)	100.0 (98.8)
Mean I/sigma(I)	8.7 (0.3)	10.3 (0.4)	7.8 (0.4)	6.7 (0.3)
R-merge	0.130 (6.34)	0.123 (2.71)	0.189 (2.73)	0.152 (3.65)
R-pim	0.053 (2.53)	0.050 (1.09)	0.077 (1.11)	0.062 (1.47)
CC-half	0.998 (0.36)	0.998 (0.31)	0.997 (0.28)	0.997 (0.42)
**Refinement:**				
Refinement resolution range (Å)	56.37 - 1.97 (2.00 - 1.97)	30.56 - 2.11 (2.12 - 2.11)	56.38 - 2.59 (2.61 - 2.59)	56.32 - 1.99 (2.02 - 1.99)
No. reflections	48289 (966)	40484 (810)	22087 (442)	46051 (922)
No. reflections (Rfree)	2339 (39)	2029 (37)	1175 (18)	2004 (40)
R-factor	0.263 (0.452)	0.262 (0.437)	0.251 (0.398)	0.254 (0.510)
Rfree	0.316 (0.520)	0.333 (0.573)	0.343 (0.364)	0.316 (0.486)
Number of total atoms	5632	5768	5459	5851
atoms for macromolecules	5373	5552	5282	5552
atoms for ligands	80	76	24	92
atoms for waters	179	140	153	207
Average B-factor ($Å^2$)	66.3	69.1	79.9	61.3
RMS(bonds) (Å)	0.008	0.008	0.007	0.008
RMS(bond angles) (°)	1.09	1.01	0.89	1
RMS(dihedral angles) (°)	3.58	3.3	2.68	3.41

Protein	BtkS	Btk	Btk	BtkS
PDB code	7I93	9RL9	7I9G	9T21
Ligand	**27**	**28**	**28**	**29**
**Data Collection:**				
Beamline	DIAMOND BEAMLINE i04	DIAMOND BEAMLINE i03	DIAMOND BEAMLINE i03	DIAMOND BEAMLINE i04
Wavelength (Å)	0.9537	0.9763	0.9763	0.9537
Resolution range (Å)	56.58 - 1.91 (1.94 - 1.91)	56.24 - 2.11 (2.15 - 2.11)	57.74 - 1.70 (1.73 - 1.70)	47.99 - 1.87 (1.90 - 1.87)
Space group	P 1 2_1 1	P 1 2_1 1	P 1 2_1 1	P 1 2_1 1
Cell (a b c) (Å)	68.48 67.15 79.82	68.34 66.38 79.70	69.63 66.43 79.46	69.29 67.57 79.81
Cell (α β γ) (°)	90.00 100.86 90.00	90.00 100.25 90.00	90.00 102.63 90.00	90.00 100.28 90.00
Total reflections	384433 (18903)	284078 (14307)	542465 (26474)	417902 (20600)
Unique reflections	54217 (2626)	40587 (1998)	77629 (3701)	60135 (2930)
Multiplicity	7.1 (7.2)	7.0 (7.2)	7.0 (7.2)	6.9 (7.0)
Completeness (%)	98.0 (96.3)	99.9 (99.3)	99.4 (96.0)	100.0 (98.7)
Mean I/sigma(I)	9.6 (0.2)	10.3 (0.4)	14.2 (0.4)	8.7 (0.2)
R-merge	0.115 (4.55)	0.123 (2.71)	0.077 (3.21)	0.138 (14.58)
R-pim	0.046 (1.83)	0.050 (1.09)	0.031 (1.28)	0.056 (5.93)
CC-half	0.998 (0.38)	0.998 (0.31)	0.999 (0.26)	0.997 (0.37)
**Refinement:**				
Refinement resolution range (Å)	27.29 - 1.91 (1.93 - 1.91)	30.56 - 2.11 (2.12 - 2.11)	57.74 - 1.70 (1.71 - 1.70)	37.00 - 1.87 (1.91 - 1.87)
No. reflections	52008 (1041)	40484 (810)	77532 (1551)	56561 (1132)
No. reflections (Rfree)	2603 (61)	2029 (37)	1997 (54)	2787 (132)
R-factor	0.258 (0.589)	0.262 (0.437)	0.259 (0.439)	0.248 (0.496)
Rfree	0.317 (0.614)	0.333 (0.573)	0.285 (0.476)	0.307 (0.536)
Number of total atoms	5516	5768	5836	6145
atoms for macromolecules	5186	5552	5552	5561
atoms for ligands	88	76	21	74
atoms for waters	242	140	263	510
Average B-factor (Å^2)	61.2	69.1	46.5	53.8
RMS(bonds) (Å)	0.008	0.008	0.009	0.01
RMS(bond angles) (°)	1.01	1.01	1.05	1.09
RMS(dihedral angles) (°)	3.42	3.3	3.91	3.41

Protein	BtkA	BtkA	Btk	BtkA
PDB code	7I9C	9RMO	7I9b	9RN5
Ligand	**30**	**31**	**32**	**33**
**Data Collection:**				
Beamline	DIAMOND BEAMLINE i03	DIAMOND BEAMLINE i04-1	DIAMOND BEAMLINE i04	DIAMOND BEAMLINE i04
Wavelength (Å)	0.9188	0.9762	0.9537	0.9537
Resolution range (Å)	56.40 - 1.92 (1.950- 1.92)	56.53 - 1.80 (1.83 - 1.80)	56.29 - 2.77 (2.82 - 2.77)	51.00 - 1.83 (1.86 - 1.83)
Space group	$P\ 1\ 2_1\ 1$	$P\ 1\ 2_1\ 1$	$P\ 1\ 2_1\ 1$	$P\ 1\ 2_1\ 1$
Cell (a b c) (Å)	68.15 66.36 79.62	68.92 66.88 79.94	68.00 66.03 79.69	68.98 67.15 79.78
Cell (α β γ) (°)	90.00 100.95 90.00	90.00 100.11 90.00	90.00 100.81 90.00	90.00 100.58 90.00
Total reflections	362968 (16508)	464304 (23663)	124445 (6027)	439862 (22282)
Unique reflections	53350 (2635)	66462 (3318)	17891 (879)	63456 (3134)
Multiplicity	6.8 (6.3)	7.0 (7.1)	7.0 (6.9)	6.9 (7.1)
Completeness (%)	99.8 (99.2)	100.0 (99.8)	100.0 (99.2)	99.8 (98.1)
Mean I/sigma(I)	6.3 (0.2)	7.8 (0.2)	6.5 (0.5)	10.3 (0.3)
R-merge	0.176 (3.19)	0.148 (4.59)	0.263 (2.50)	0.105 (3.41)
R-pim	0.074 (1.40)	0.060 (1.84)	0.107 (1.03)	0.043 (1.37)
CC-half	0.980 (0.25)	0.997 (0.32)	0.990 (0.28)	0.998 (0.57)
**Refinement:**				
Refinement resolution range (Å)	22.30 - 1.94 (1.95 - 1.94)	56.53 - 1.80 (1.83 - 1.80)	56.29 - 2.77 (2.79 - 2.77)	51.01 - 1.83 (1.84 - 1.83)
No. reflections	47517 (951)	64254 (1286)	17852 (380)	62580 (1252)
No. reflections (Rfree)	1722 (32)	3187 (74)	951 (24)	3105 (58)
R-factor	0.304 (0.660)	0.251 (0.508)	0.243 (0.378)	0.269 (0.544)
Rfree	0.344 (0.711)	0.293 (0.517)	0.331 (0.371)	0.322 (0.526)
Number of total atoms	5500	5610	5420	5441
atoms for macromolecules	5356	5239	5309	5188
atoms for ligands	77	79	75	78
atoms for waters	67	292	36	175
Average B-factor (Å^2)	52.4	47.5	77.8	54.1
RMS(bonds) (Å)	0.008	0.009	0.007	0.009
RMS(bond angles) (°)	0.99	1	0.91	1.04
RMS(dihedral angles) (°)	3.2	3.66	2.72	3.56

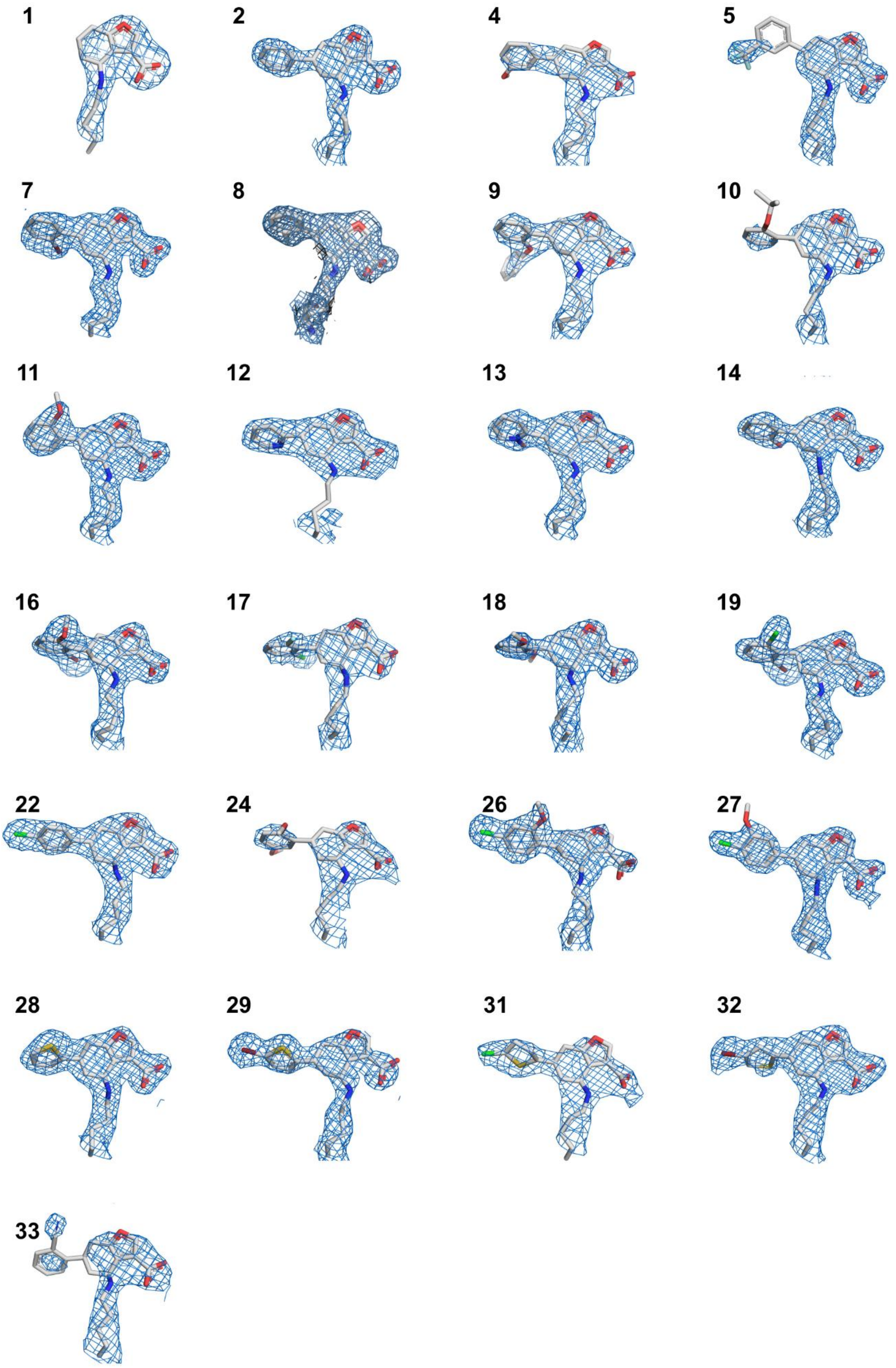

Figure S1. The 2mFo-DFc electron density maps of BTK PH domain binder complexes contoured at 1.0σ before ligand fitting for the crystal structures described.

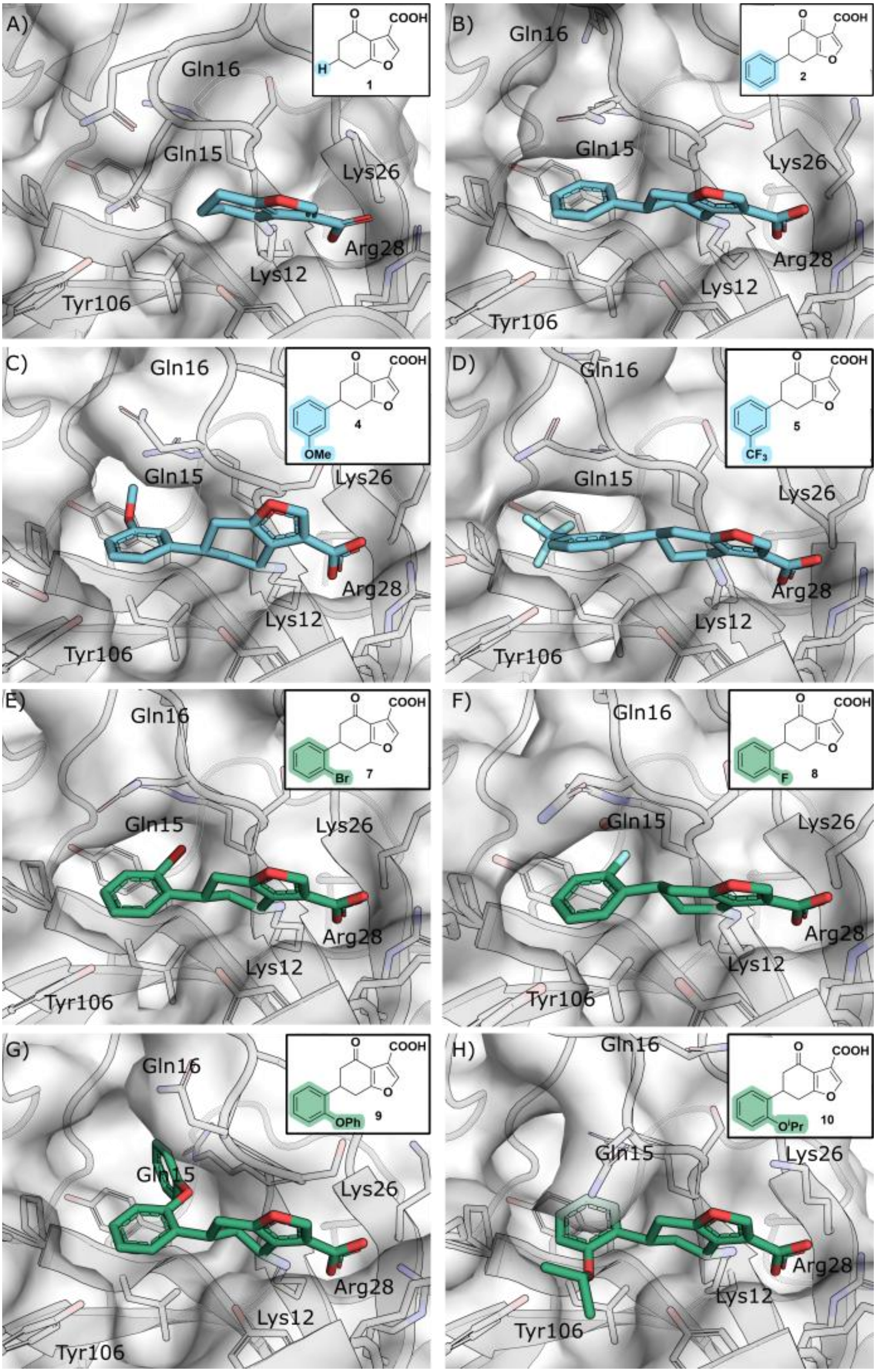
A)
Gln16
Gln15
Lys26
Arg28
Lys12
Tyr106
COOH
1
B)
Gln16
Gln15
Lys26
Arg28
Lys12
Tyr106
COOH
2
C)
Gln16
Gln15
Lys26
Arg28
Lys12
Tyr106
COOH
OMe
4
D)
Gln16
Gln15
Lys26
Arg28
Lys12
Tyr106
COOH
CF3
5
E)
Gln16
Gln15
Lys26
Arg28
Lys12
Tyr106
COOH
Br
7
F)
Gln16
Gln15
Lys26
Arg28
Lys12
Tyr106
COOH
F
8
G)
Gln16
Gln15
Lys26
Arg28
Lys12
Tyr106
COOH
OPh
9
H)
Gln16
Gln15
Lys26
Arg28
Lys12
Tyr106
COOH
10

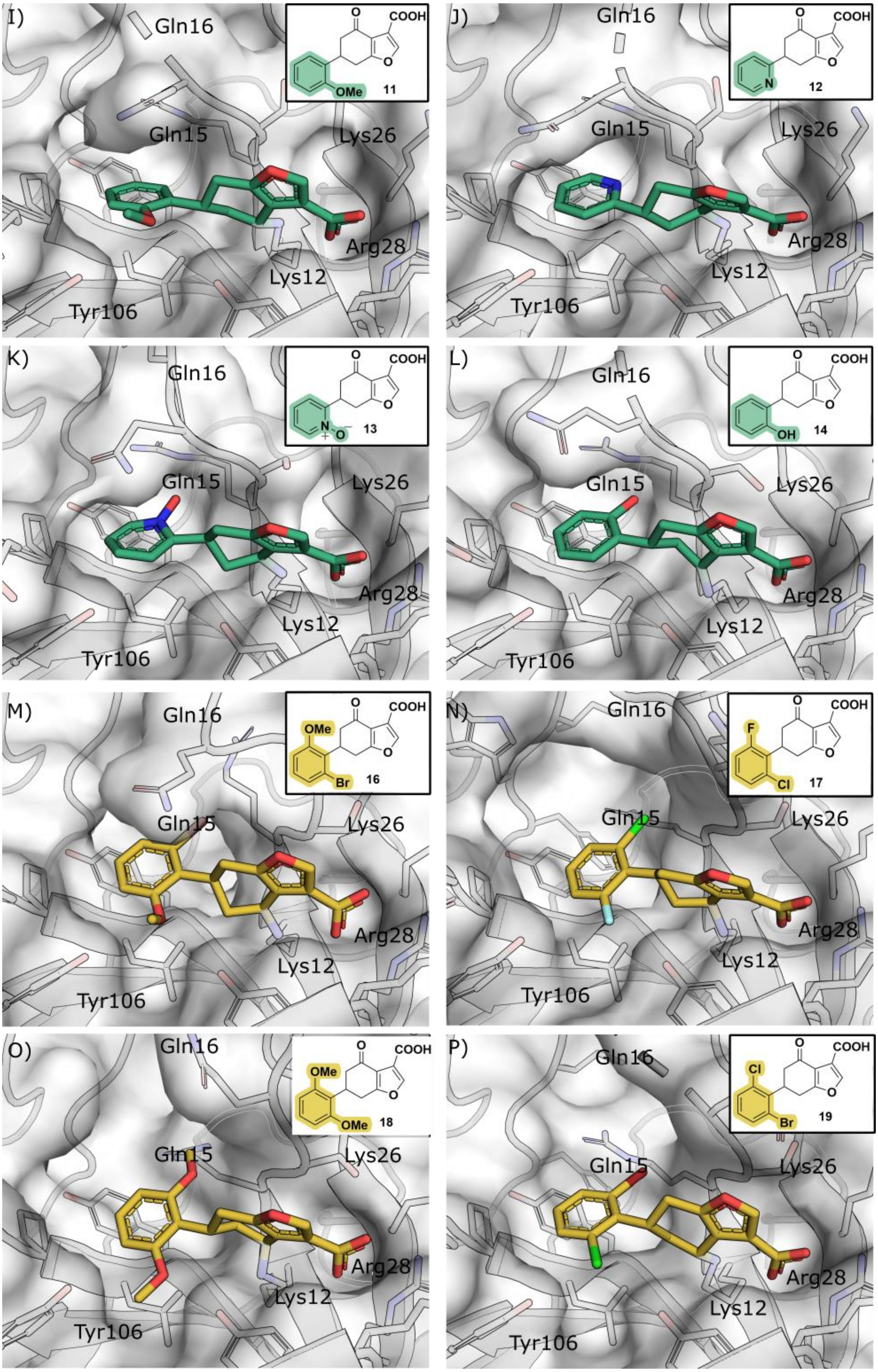
I)
Gln16
Gln15
Lys26
Arg28
Lys12
Tyr106
O
COOH
OMe
11
J)
Gln16
Gln15
Lys26
Arg28
Lys12
Tyr106
N
12
K)
Gln16
Gln15
Lys26
Arg28
Lys12
Tyr106
13
L)
Gln16
Gln15
Lys26
Arg28
Lys12
Tyr106
OH
14
M)
Gln16
Gln15
Lys26
Arg28
Lys12
Tyr106
OMe
Br
16
N)
Gln16
Gln15
Lys26
Arg28
Lys12
Tyr106
F
Cl
17
O)
Gln16
Gln15
Lys26
Arg28
Lys12
Tyr106
OMe
OMe
18
P)
Gln16
Gln15
Lys26
Arg28
Lys12
Tyr106
Cl
Br
19

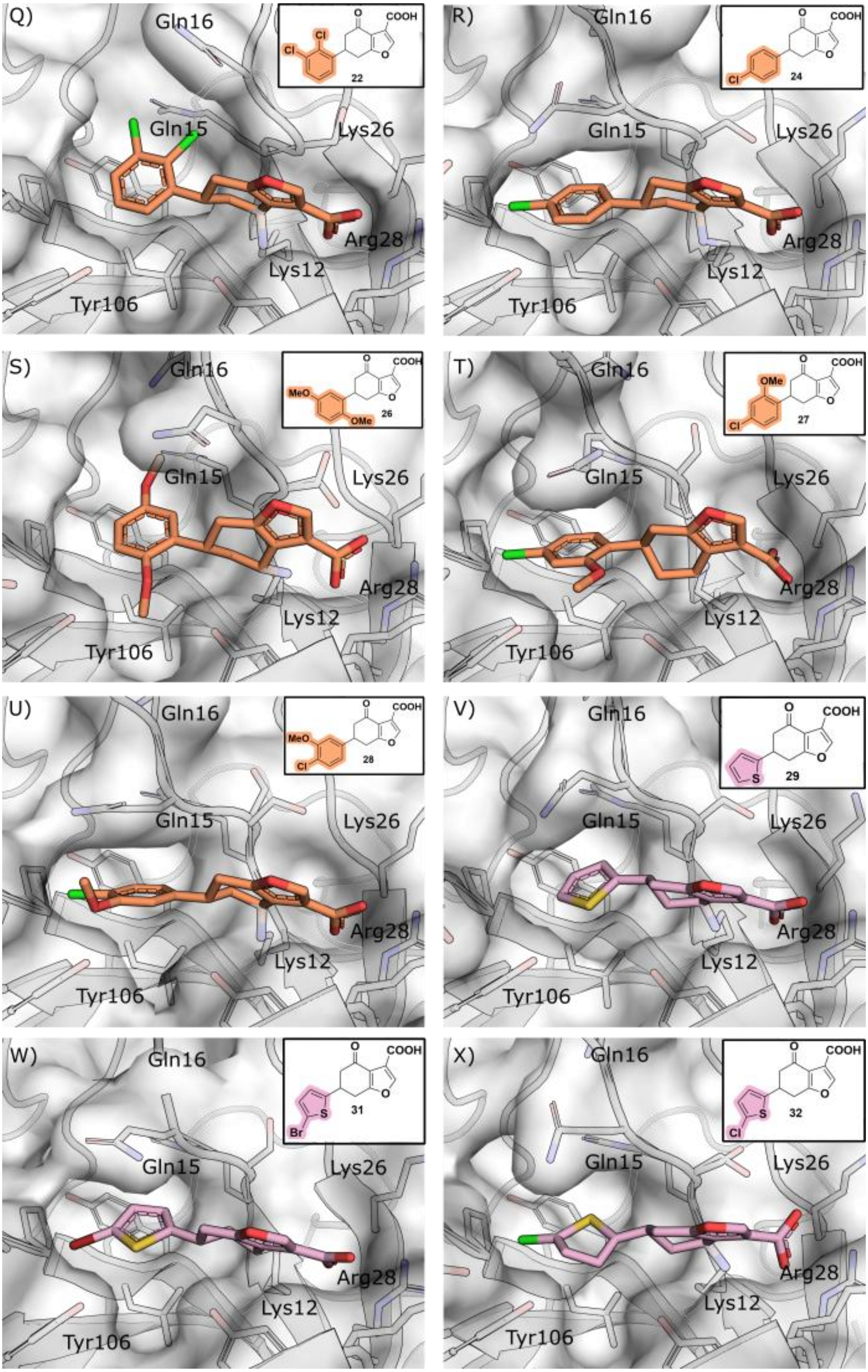

Q)
Gln16
Gln15
Lys26
Arg28
Lys12
Tyr106
COOH
Cl
Cl
22
R)
Gln16
Gln15
Lys26
Arg28
Lys12
Tyr106
COOH
Cl
24
S)
Gln16
Gln15
Lys26
Arg28
Lys12
Tyr106
COOH
MeO
OMe
26
T)
Gln16
Gln15
Lys26
Arg28
Lys12
Tyr106
COOH
OMe
Cl
27
U)
Gln16
Gln15
Lys26
Arg28
Lys12
Tyr106
COOH
MeO
Cl
28
V)
Gln16
Gln15
Lys26
Arg28
Lys12
Tyr106
COOH
S
29
W)
Gln16
Gln15
Lys26
Arg28
Lys12
Tyr106
COOH
S
Br
31
X)
Gln16
Gln15
Lys26
Arg28
Lys12
Tyr106
COOH
S
Cl
32

--

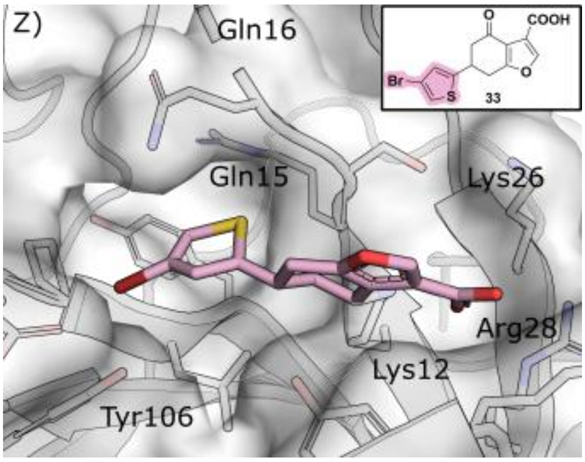

Figure S2. The crystal structures of all the compounds in this study bound to the BTK PH domain. Key residues are highlighted.

S2 Compounds NMR Spectra

4-oxo-4,5,6,7-tetrahydrobenzofuran-3-carboxylic acid (**1**) ^{1}H NMR (400 MHz, $CDCl_3$)

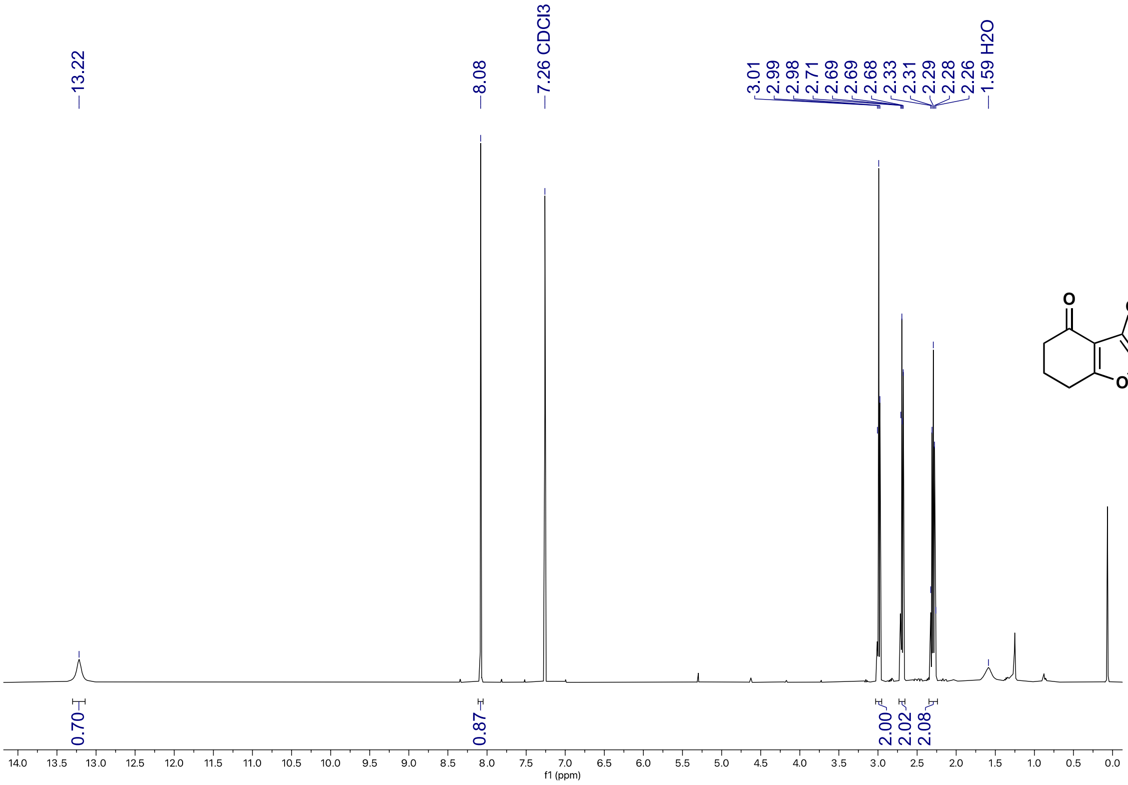

4-oxo-4,5,6,7-tetrahydrobenzofuran-3-carboxylic acid (**1**) ^{13}C NMR (101 MHz, $CDCl_3$)

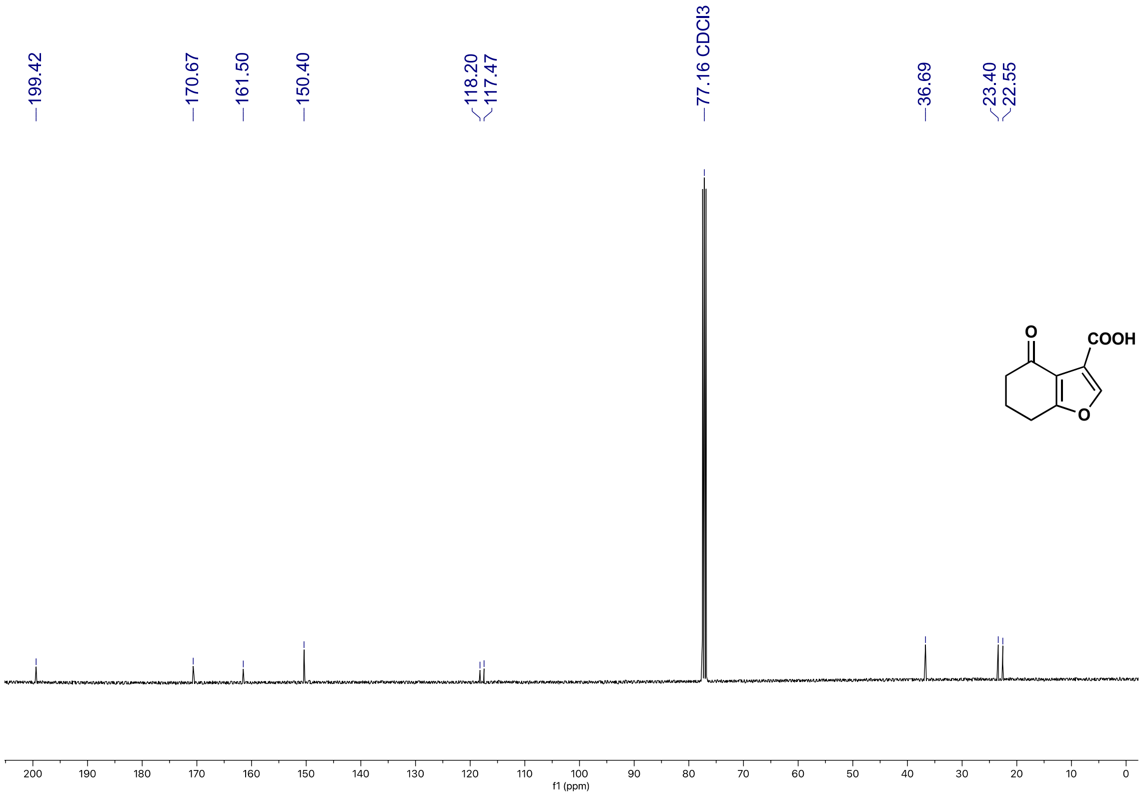

5-phenylcyclohexane-1,3-dione (**2c**) ^{1}H NMR (500 MHz, CD_3OD)

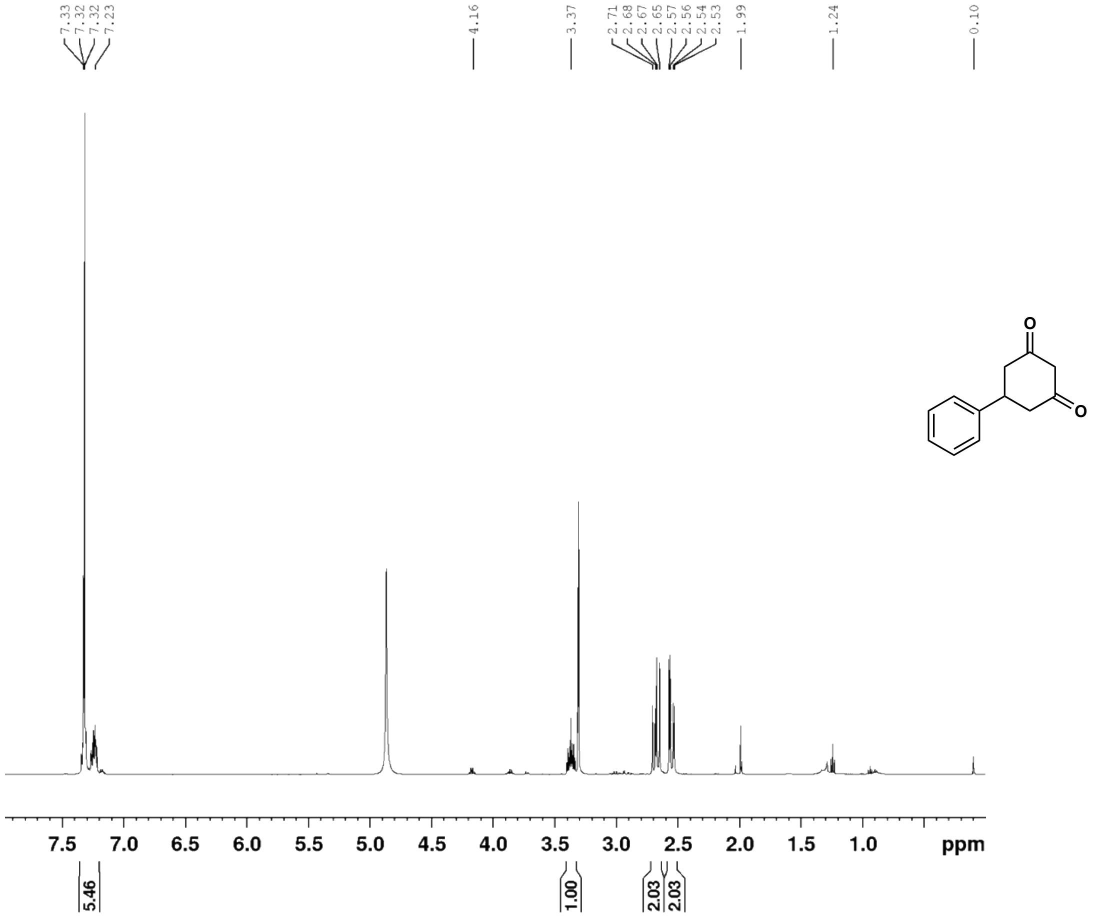

5-phenylcyclohexane-1,3-dione (**2c**) ^{13}C NMR (125 MHz, CD_3OD)

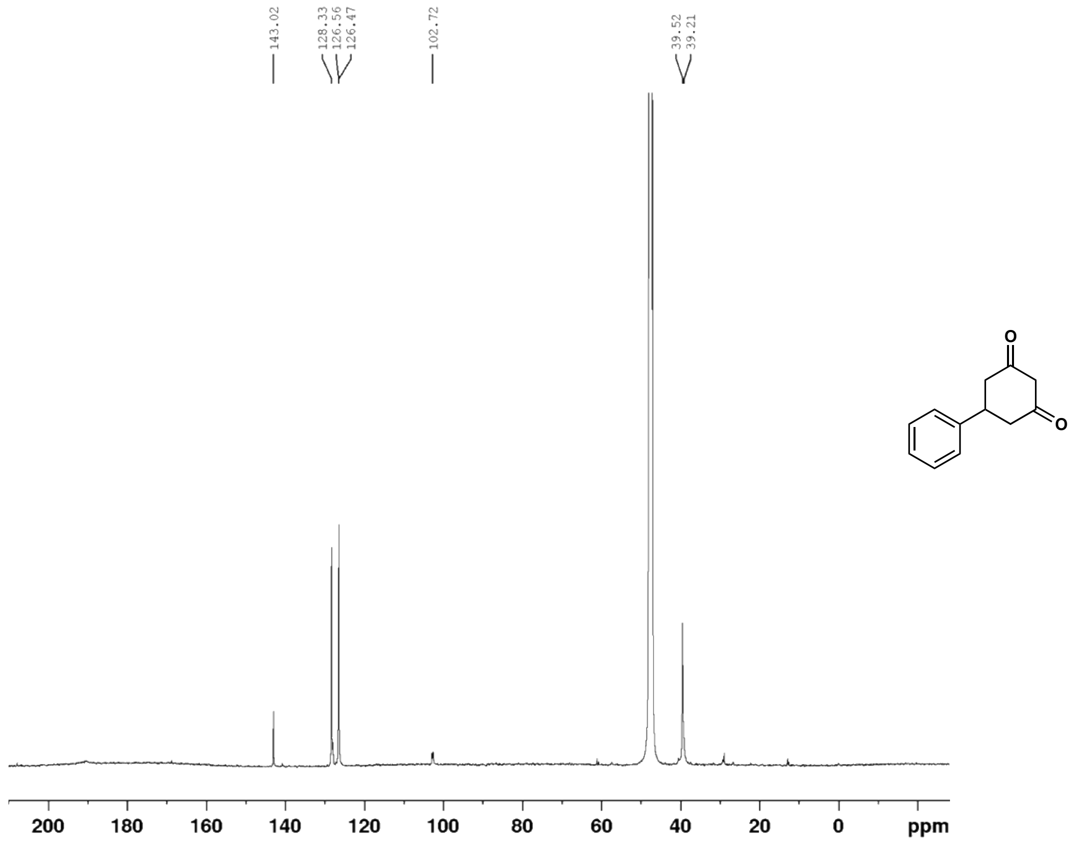

3-carboxy-6-phenyl-4,5,6,7-tetrahydrobenzofuran-4-one (**2**) ^{1}H NMR (500 MHz, $CDCl_3$)

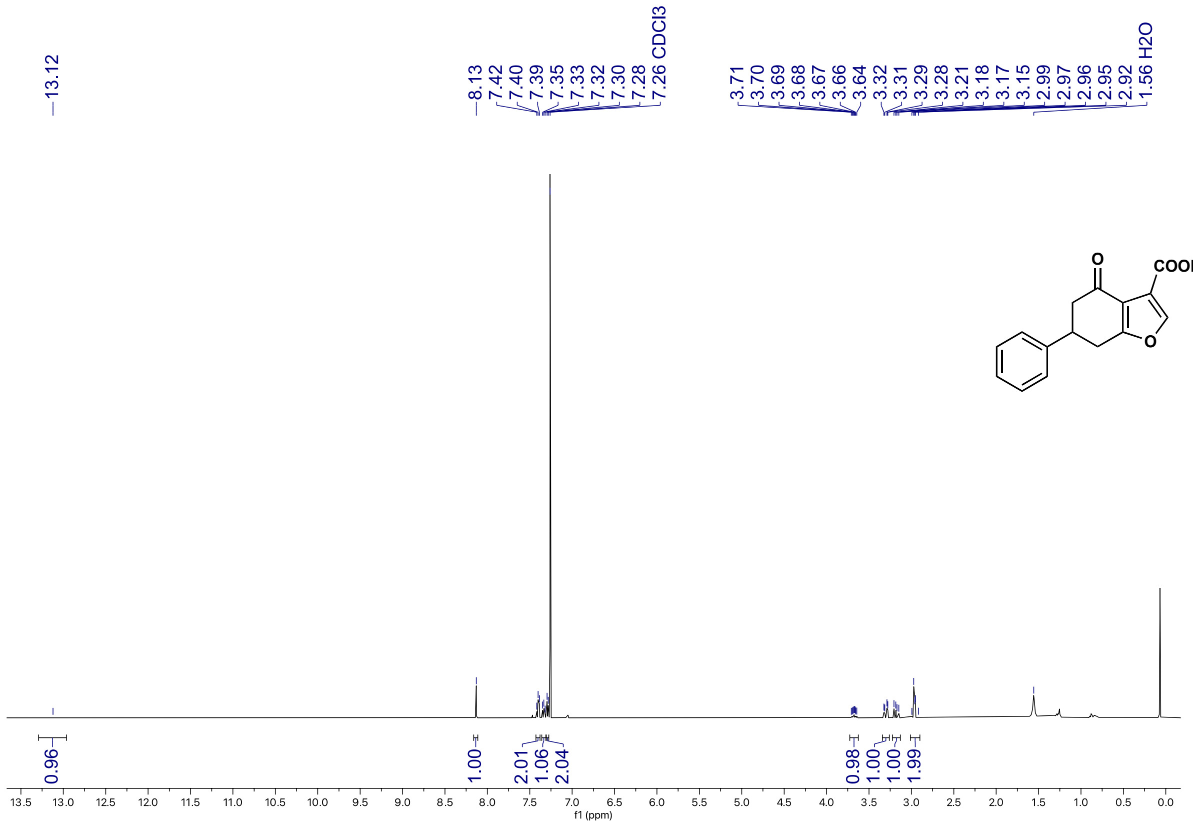

3-carboxy-6-phenyl-4,5,6,7-tetrahydrobenzofuran-4-one (**2**) ^{13}C NMR (126 MHz, $CDCl_3$)

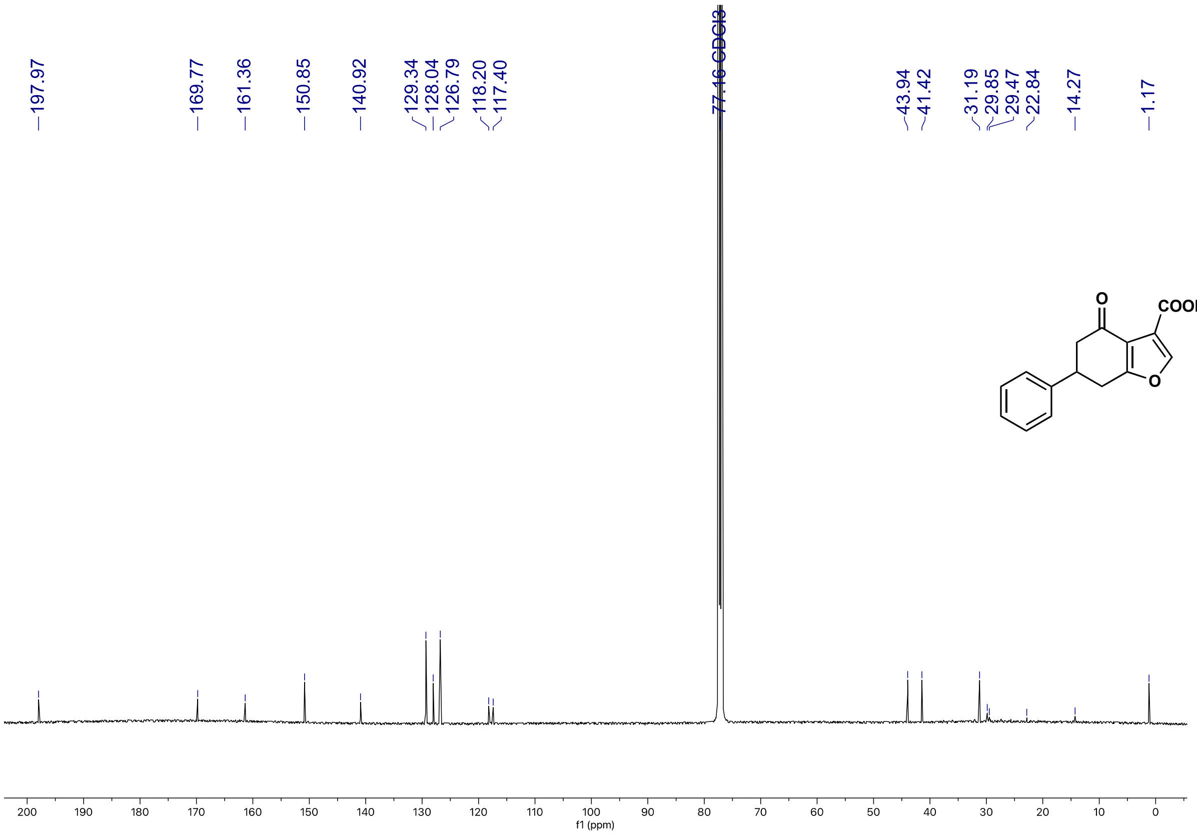

(*E*)-4-(naphthalen-2-yl)but-3-en-2-one (**3b**) ^{1}H NMR (500 MHz, $CDCl_3$)

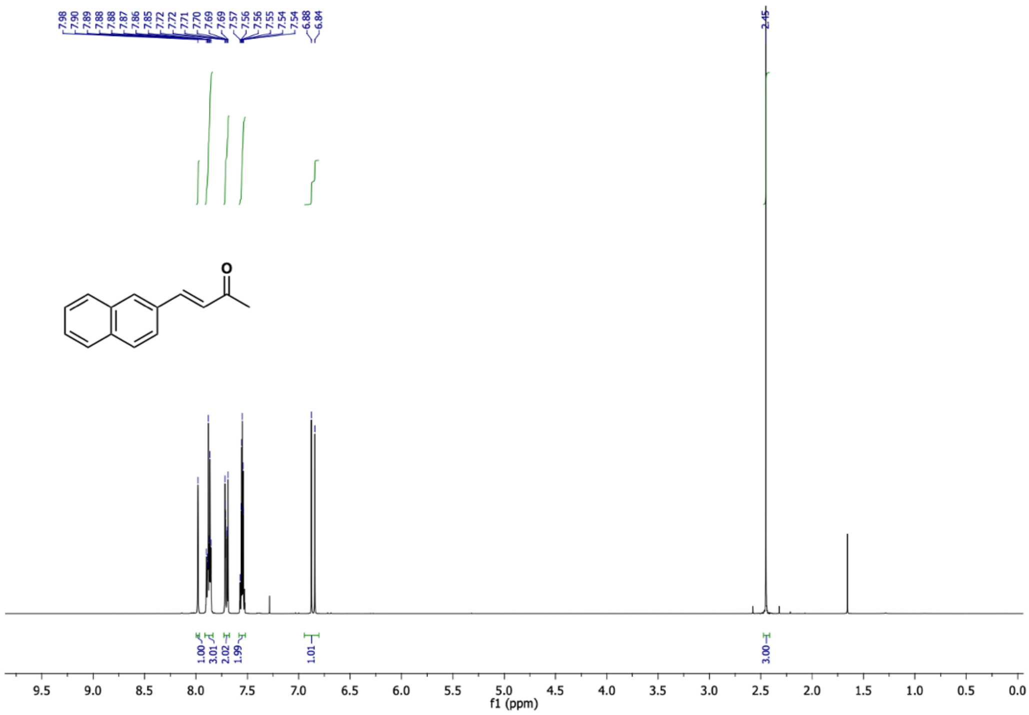

(*E*)-4-(naphthalen-2-yl)but-3-en-2-one (**3b**) ^{13}C NMR (126 MHz, $CDCl_3$)

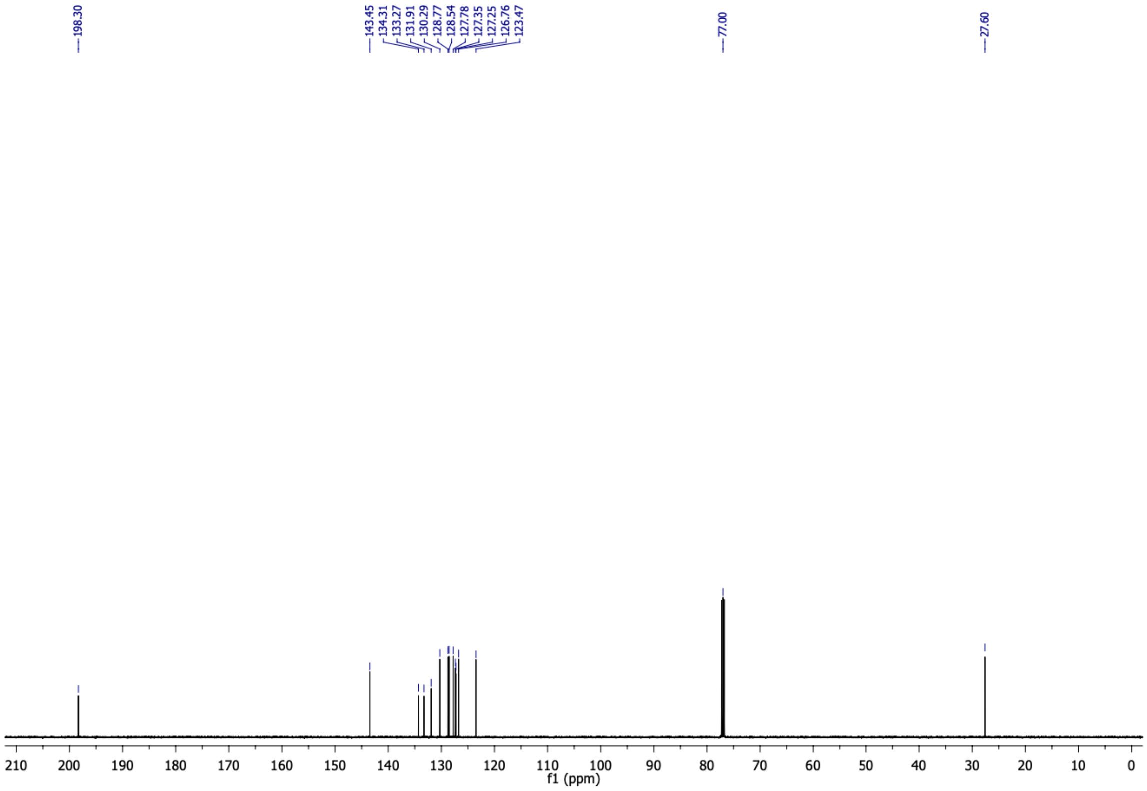

5-(naphthalen-2-yl)cyclohexane-1,3-dione (**3c**) ^{1}H NMR (500 MHz, d_6-DMSO)

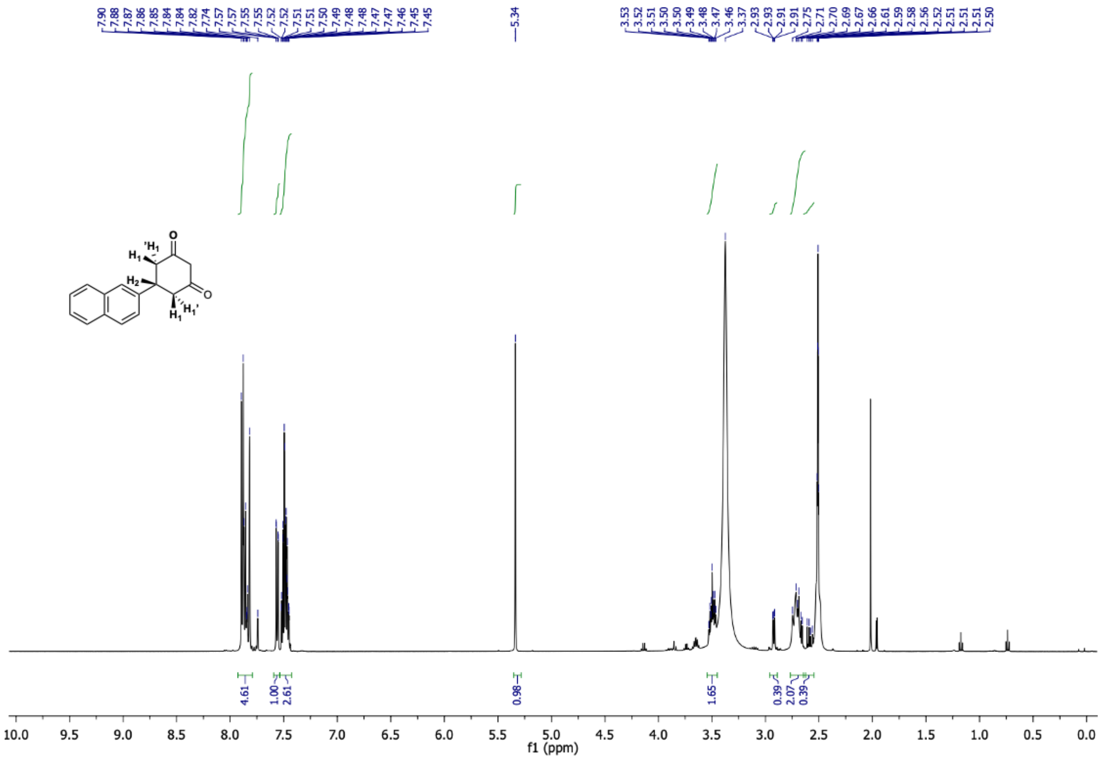

5-(naphthalen-2-yl)cyclohexane-1,3-dione (**3c**) ^{13}C NMR (126 MHz, d_6-DMSO)

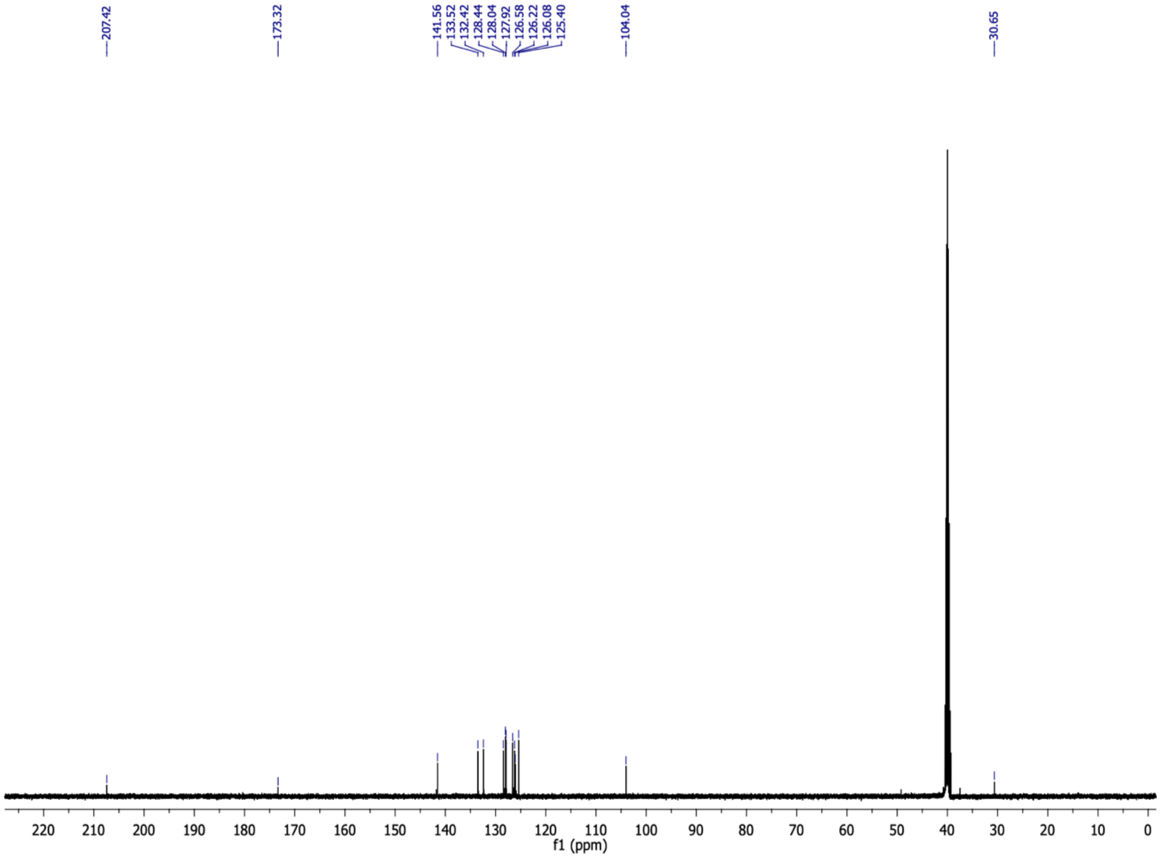

6-(naphthalen-2-yl)-4-oxo-4,5,6,7-tetrahydrobenzofuran-3-carboxylic acid (**3**) ^{1}H NMR (500 MHz, $CDCl_3$)

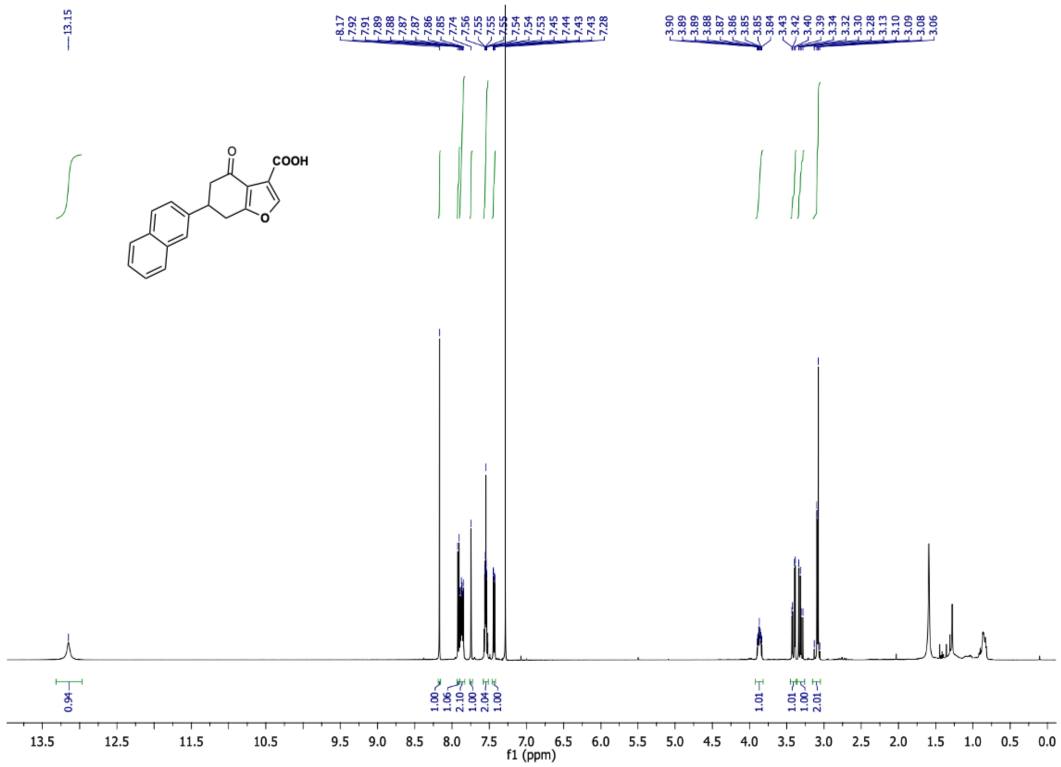

6-(naphthalen-2-yl)-4-oxo-4,5,6,7-tetrahydrobenzofuran-3-carboxylic acid (**3**) ^{13}C NMR (126 MHz, $CDCl_3$)

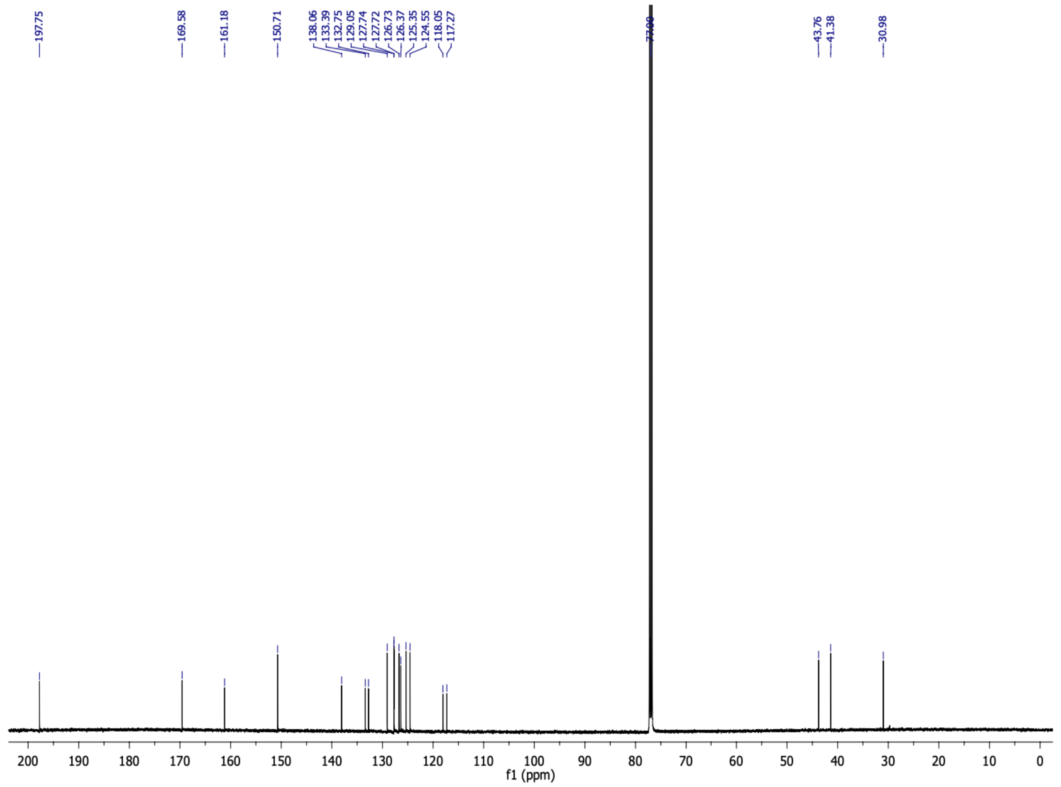

(*E*)-4-(3-methoxyphenyl)but-3-en-2-one (**4b**) ^{1}H NMR (500 MHz, $CDCl_3$)

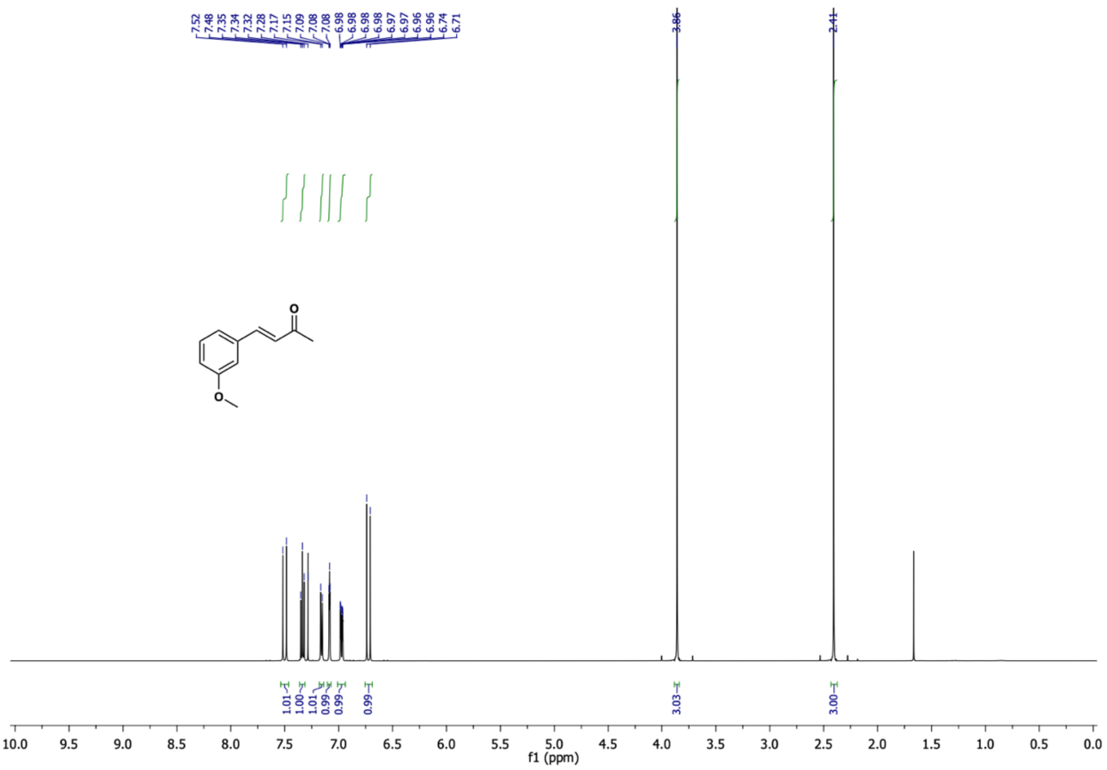

(*E*)-4-(3-methoxyphenyl)but-3-en-2-one (**4b**) ^{13}C NMR (126 MHz, $CDCl_3$)

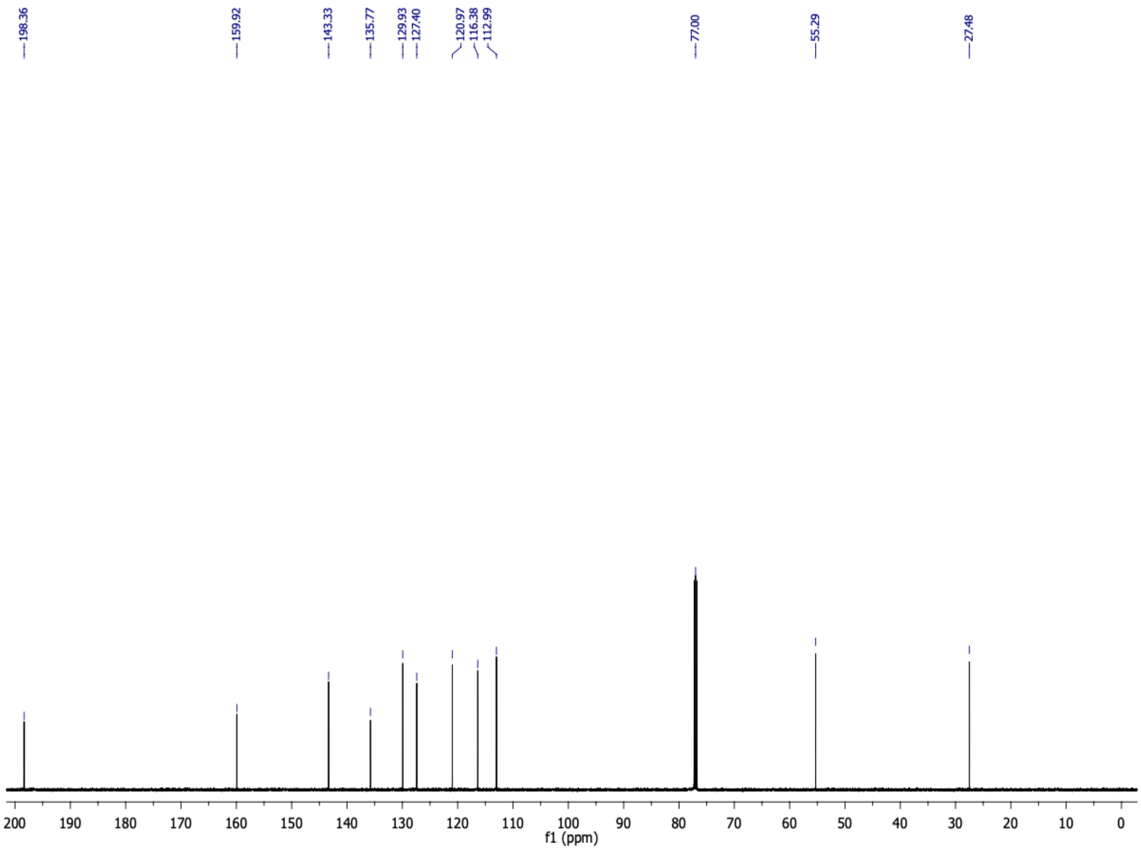

5-(3-methoxyphenyl)cyclohexane-1,3-dione (**4c**) ^{1}H NMR (500 MHz, CD_3CN)

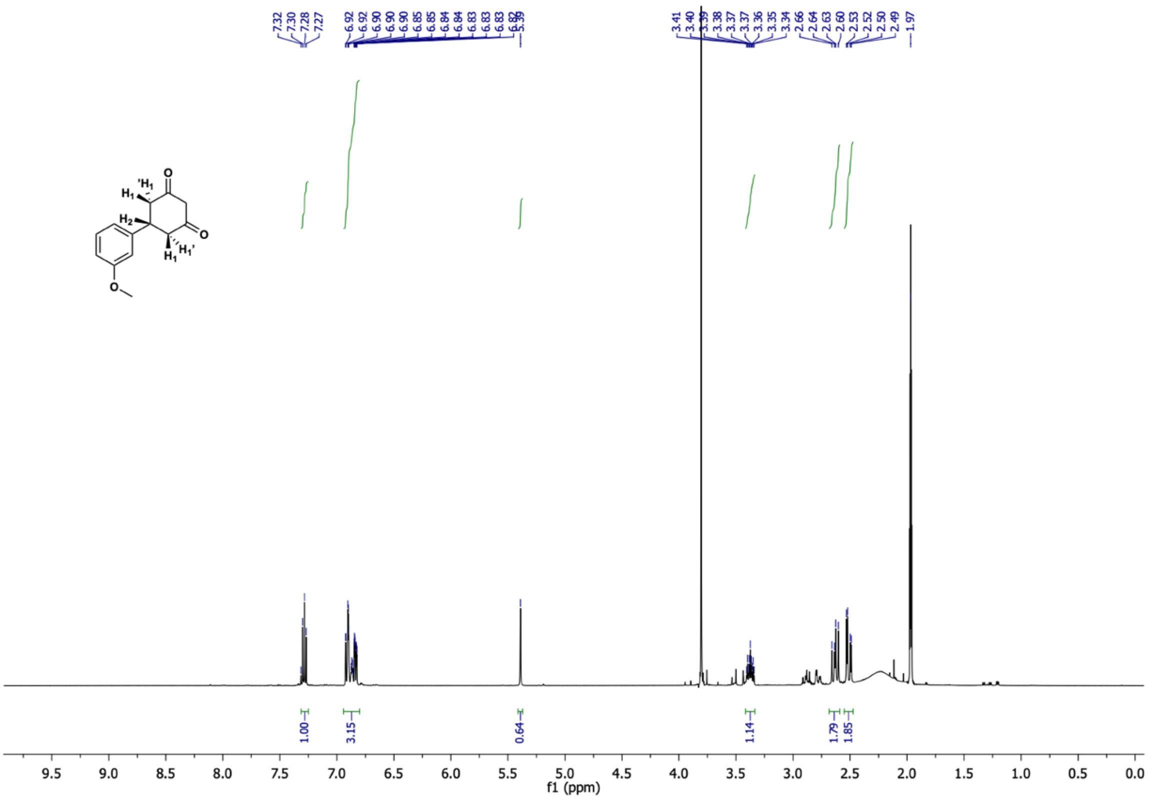

5-(3-methoxyphenyl)cyclohexane-1,3-dione (**4c**) ^{13}C NMR (126 MHz, CD_3CN)

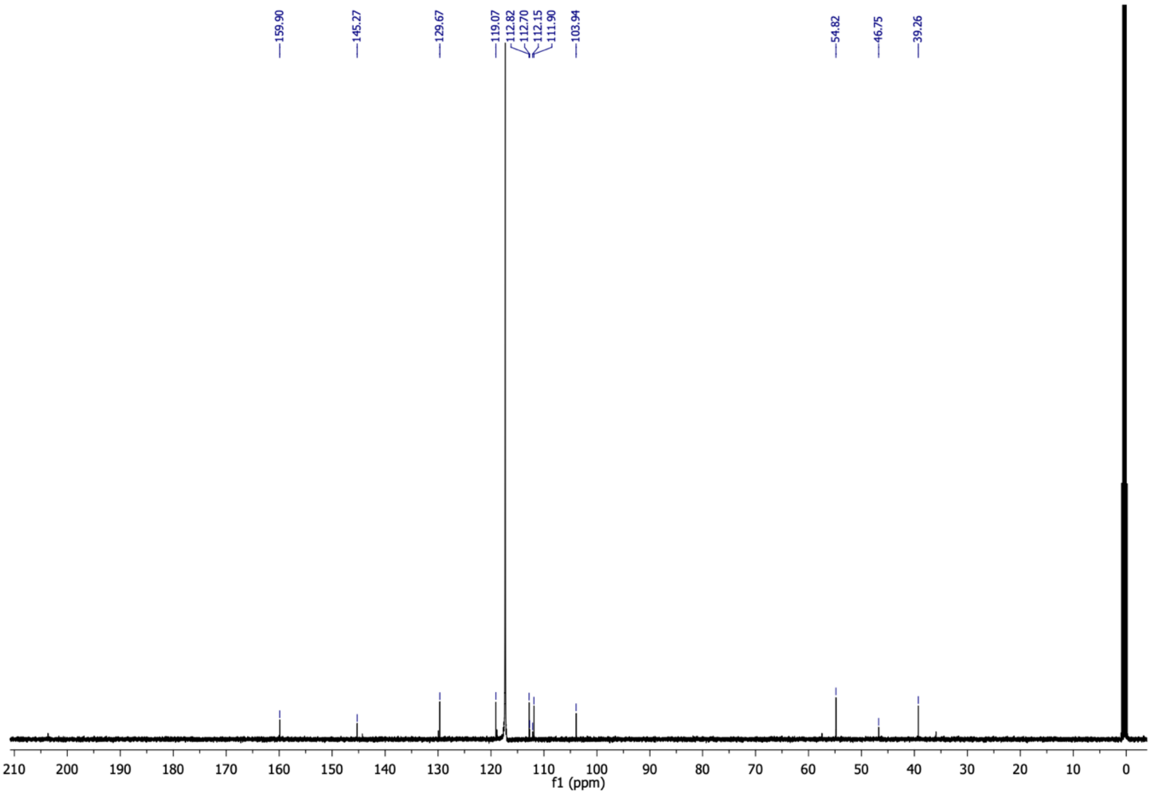

6-(3-methoxyphenyl)-4-oxo-4,5,6,7-tetrahydrobenzofuran-3-carboxylic acid (**4**) ^{1}H NMR (500 MHz, $CDCl_3$)

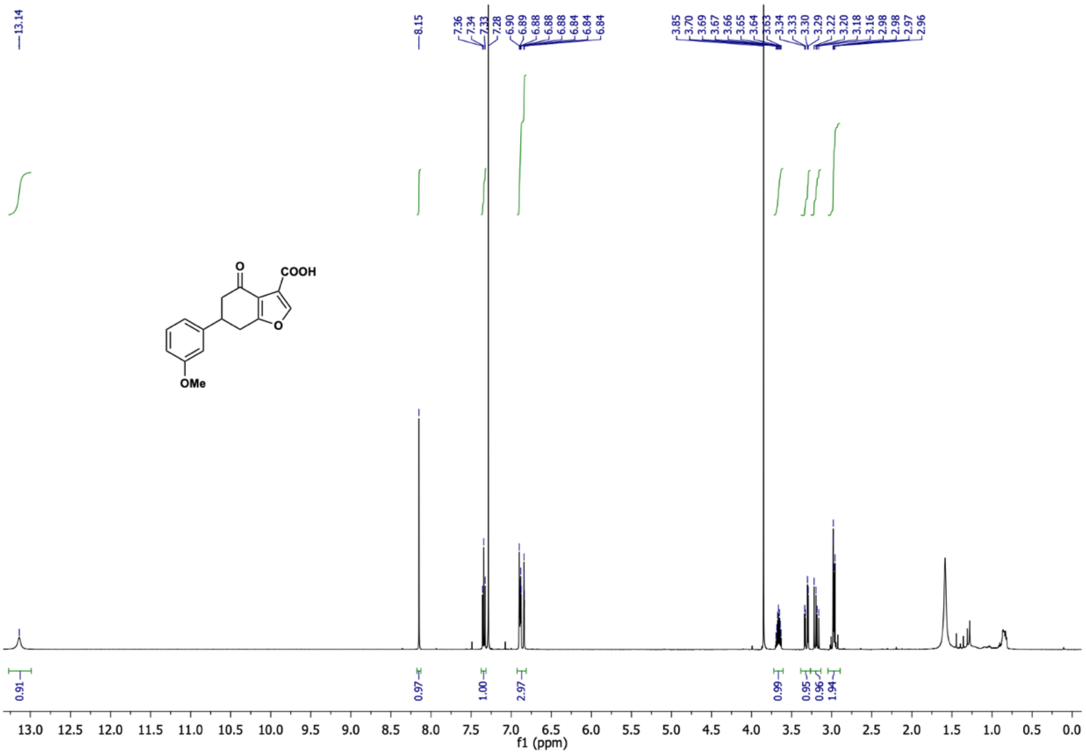

6-(3-methoxyphenyl)-4-oxo-4,5,6,7-tetrahydrobenzofuran-3-carboxylic acid (**4**) ^{13}C NMR (126 MHz, $CDCl_3$)

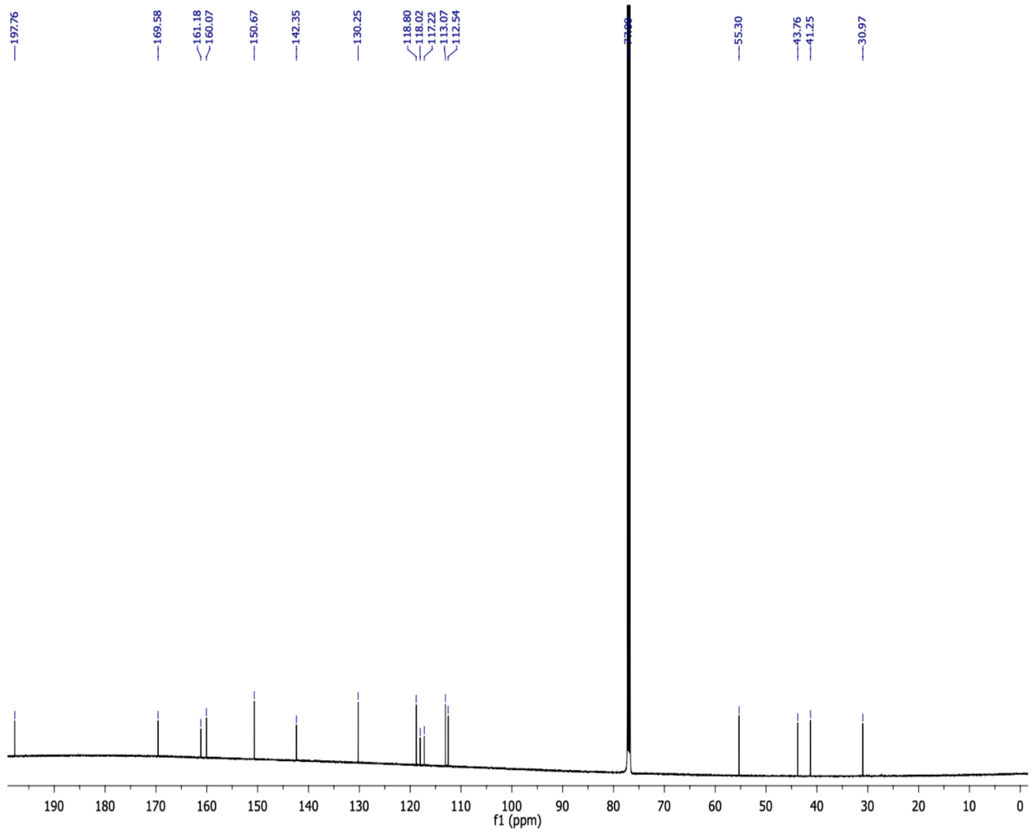

(*E*)-4-(3-(trifluoromethyl)phenyl)but-3-en-2-one (**5b**) ^{1}H NMR (500 MHz, $CDCl_3$)

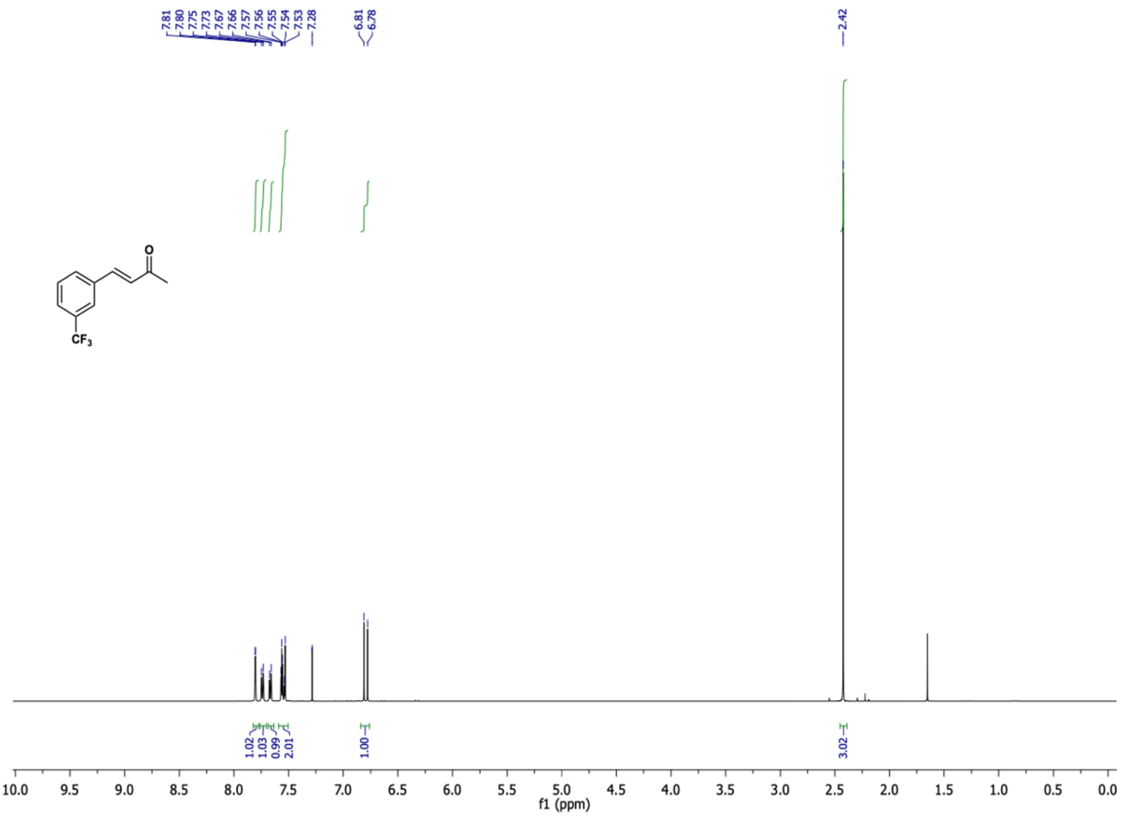

(*E*)-4-(3-(trifluoromethyl)phenyl)but-3-en-2-one (**5b**) ^{13}C NMR (126 MHz, $CDCl_3$)

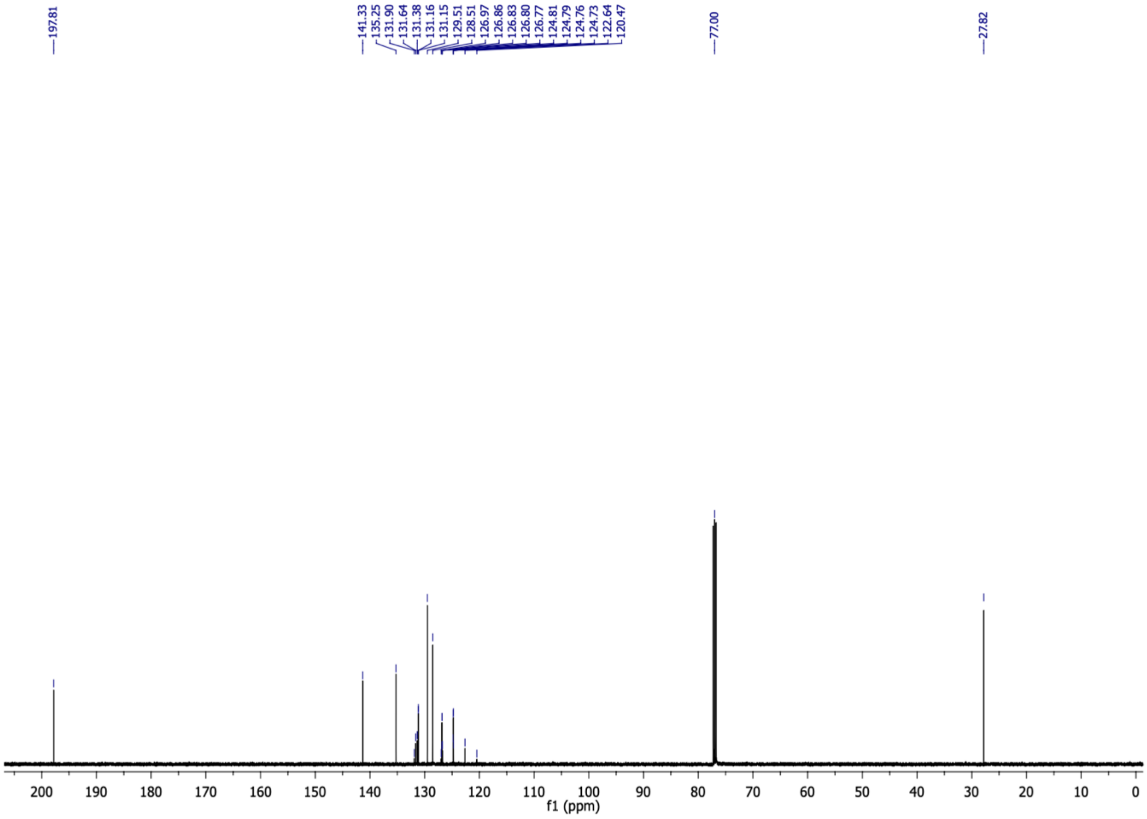

(*E*)-4-(3-(trifluoromethyl)phenyl)but-3-en-2-one (**5b**) **^{19}F NMR** (470 MHz, $CDCl_3$)

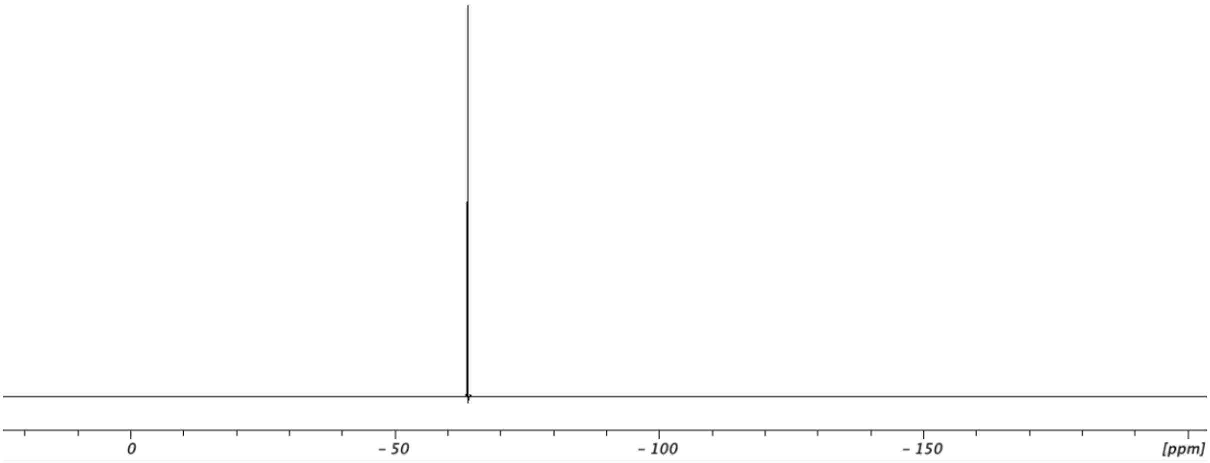

5-(3-(trifluoromethyl)phenyl)cyclohexane-1,3-dione (**5c**) ^{1}H NMR (500 MHz, CD_3OD)

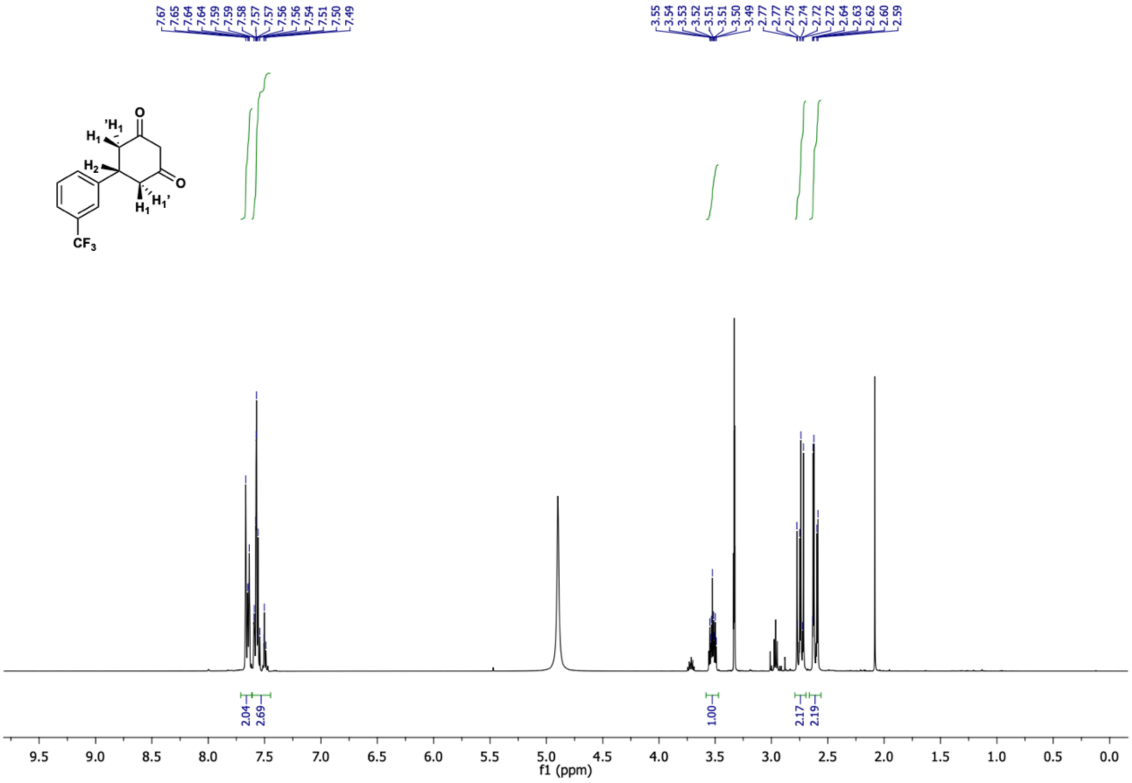

5-(3-(trifluoromethyl)phenyl)cyclohexane-1,3-dione (**5c**) ^{13}C NMR (126 MHz, CD_3OD)

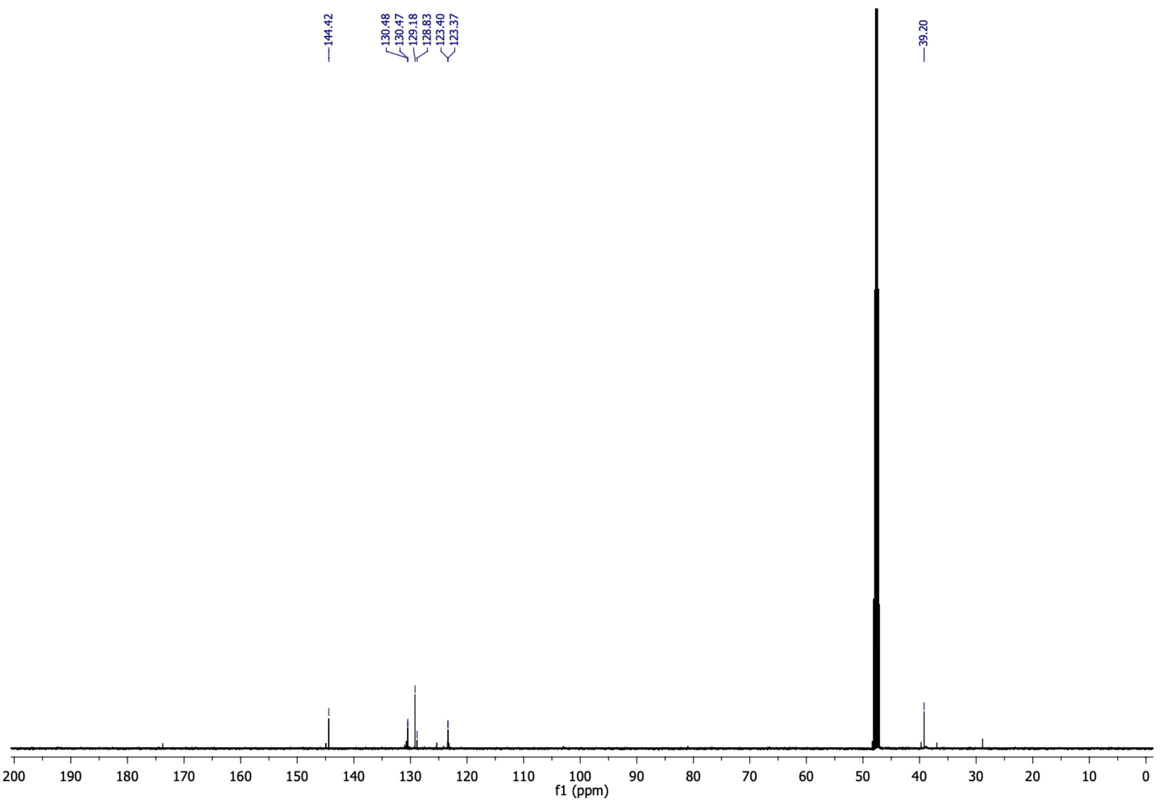

5-(3-(trifluoromethyl)phenyl)cyclohexane-1,3-dione (**5c**) ^{19}F NMR (470 MHz, $CDCl_3$)

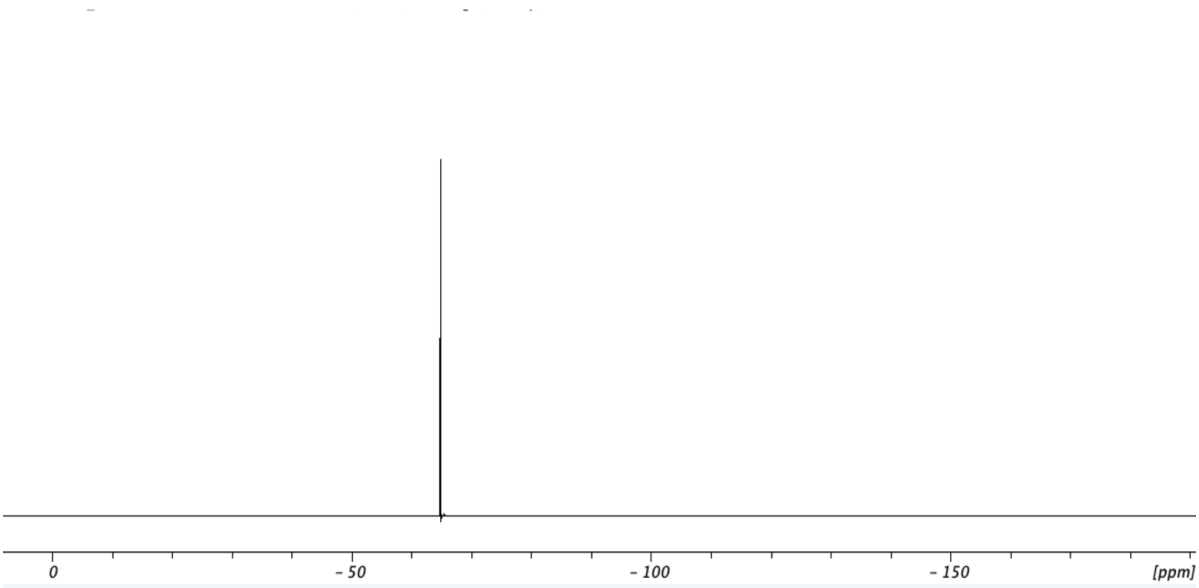

4-oxo-6-(3-(trifluoromethyl)phenyl)-4,5,6,7-tetrahydrobenzofuran-3-carboxylic acid (**5**) ^{1}H NMR (500 MHz, $CDCl_3$)

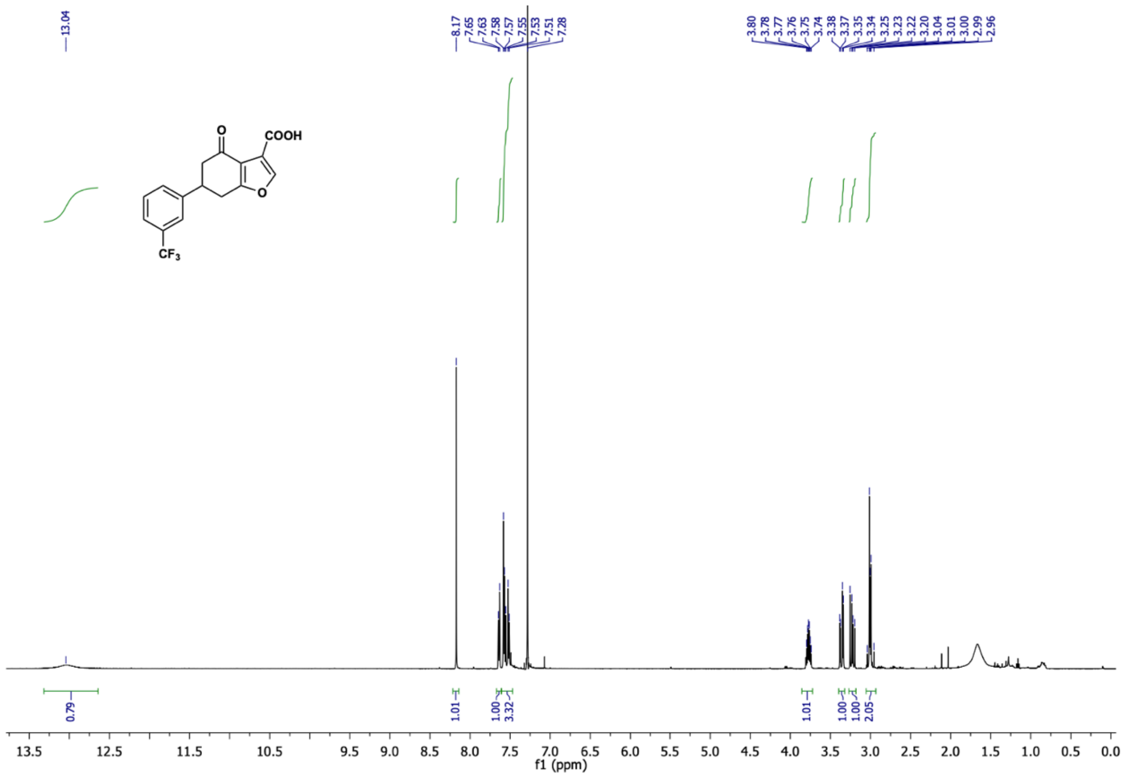

4-oxo-6-(3-(trifluoromethyl)phenyl)-4,5,6,7-tetrahydrobenzofuran-3-carboxylic acid (**5**) ^{13}C NMR (126 MHz, $CDCl_3$)

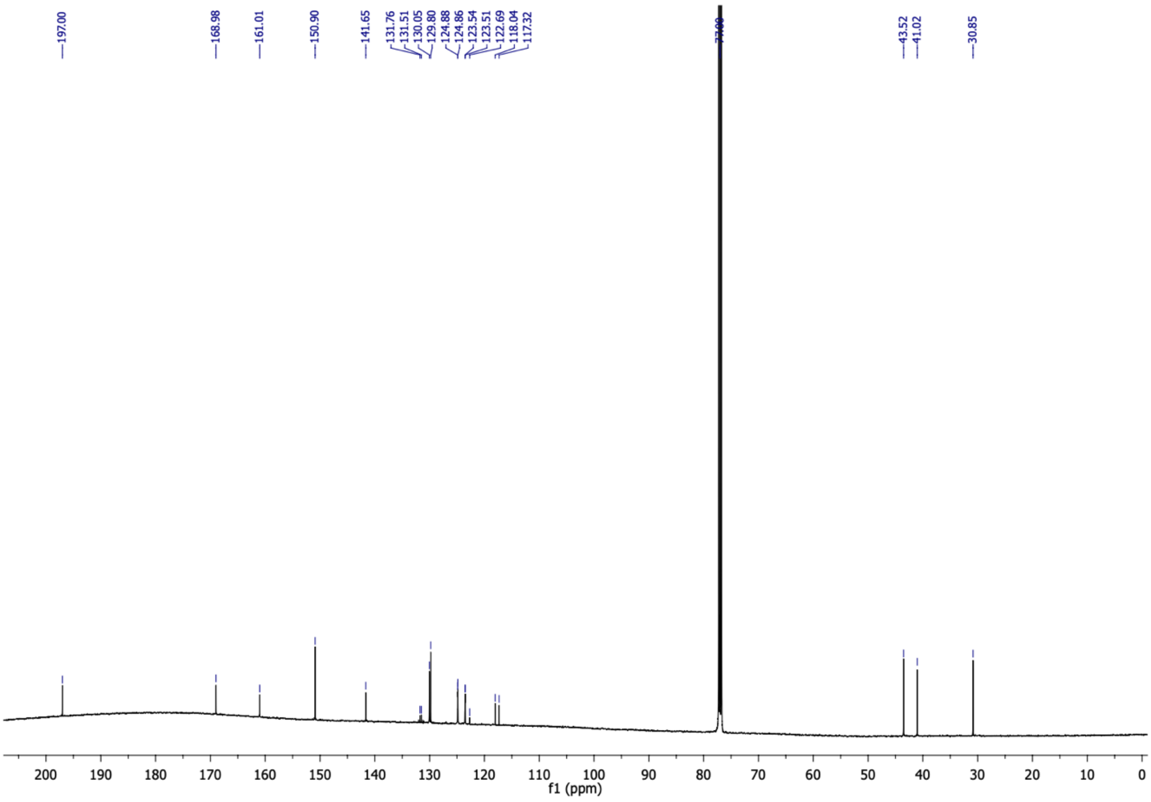

4-oxo-6-(3-(trifluoromethyl)phenyl)-4,5,6,7-tetrahydrobenzofuran-3-carboxylic acid (**5**) ^{19}F NMR (470 MHz, $CDCl_3$)

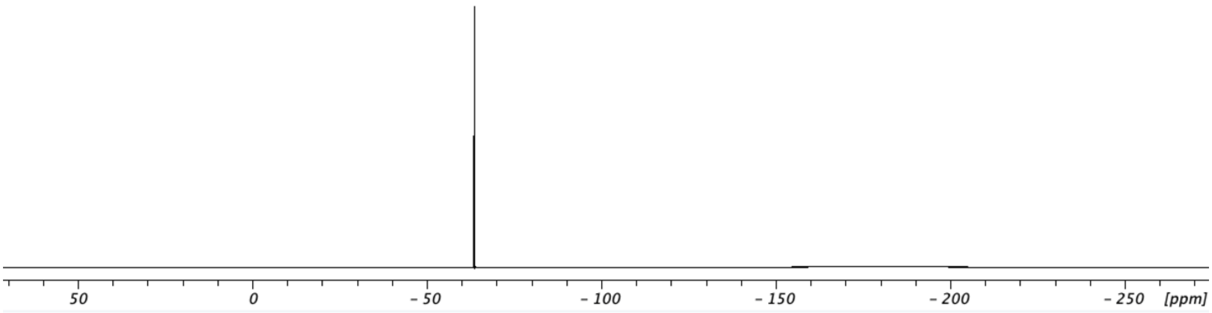

(E)-4-(benzofuran-2-yl)but-3-en-2-one (**6b**) ^{1}H NMR (400 MHz, $CDCl_3$)

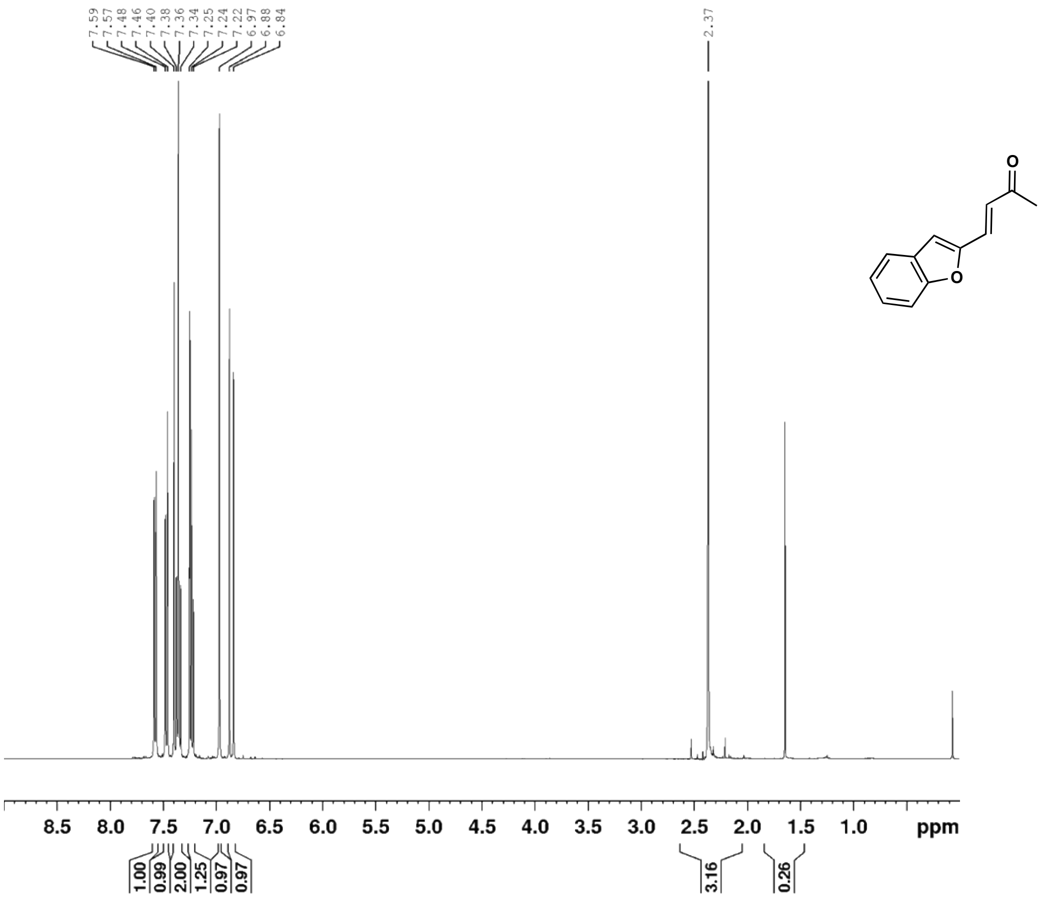

(E)-4-(benzofuran-2-yl)but-3-en-2-one (**6b**) ^{13}C NMR (101 MHz, $CDCl_3$)

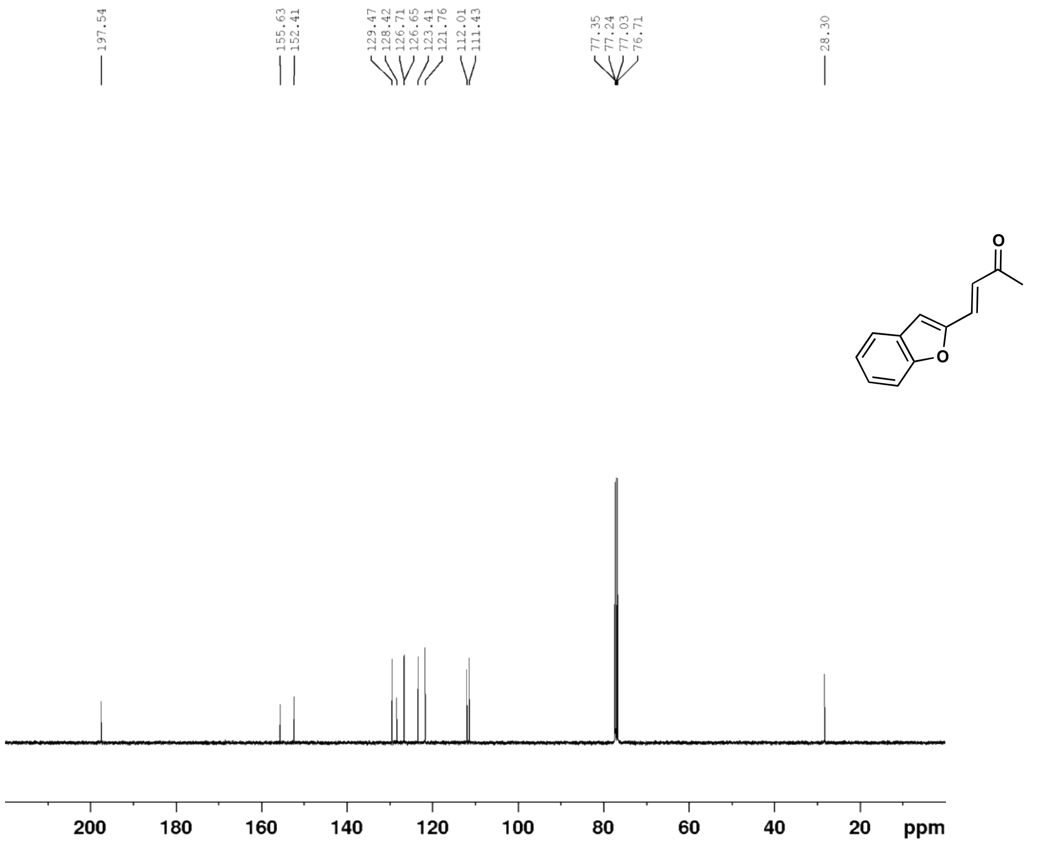

5-(benzofuran-2-yl)cyclohexane-1,3-dione (**6c**) ^{1}H NMR (400 MHz, CD_3OD)

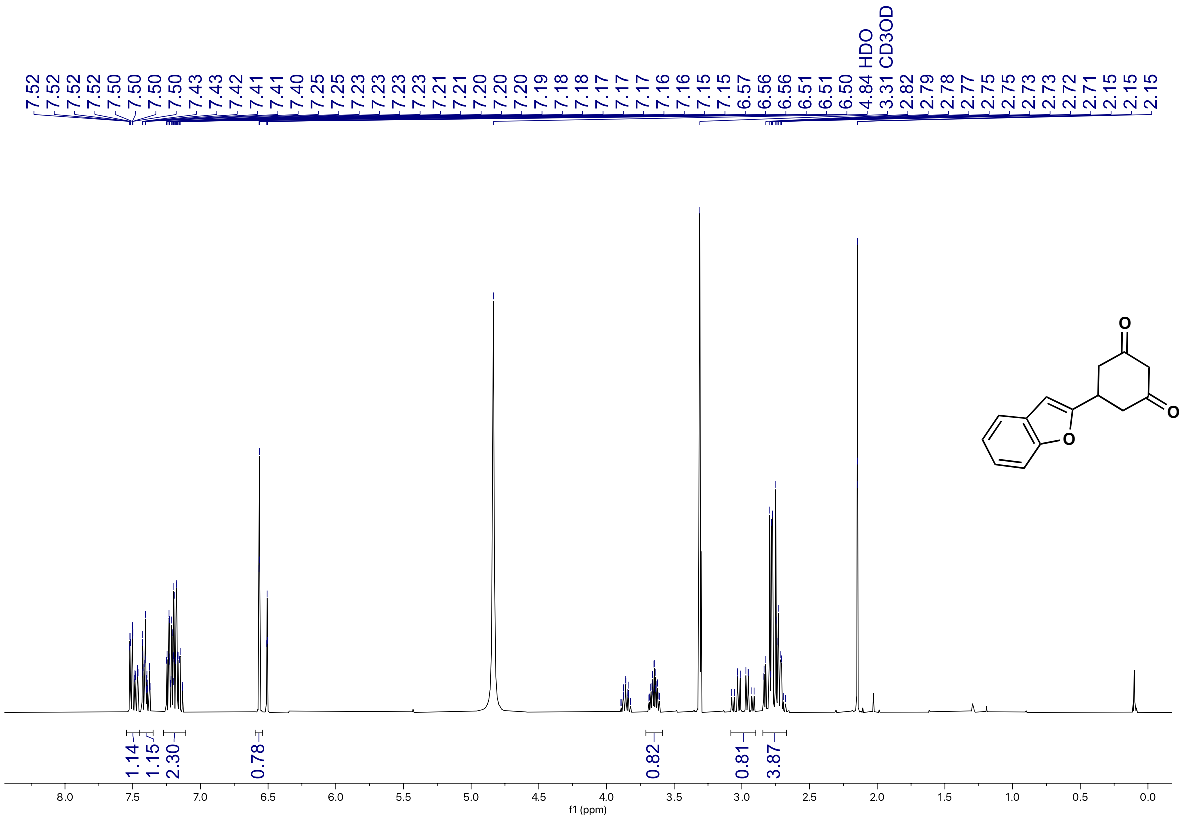

5-(benzofuran-2-yl)cyclohexane-1,3-dione (**6c**) ^{13}C NMR (101 MHz, CD_3OD)

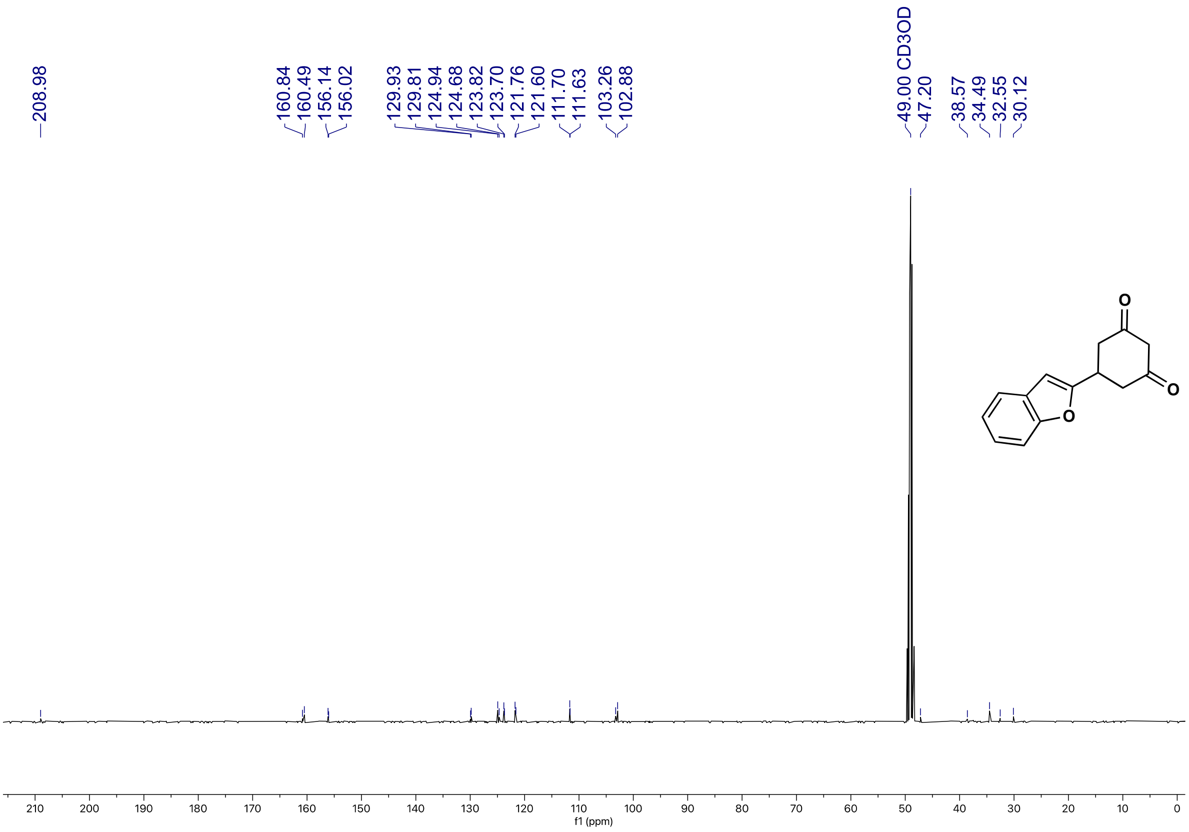

4'-oxo-4',5',6',7'-tetrahydro-[2,6'-bibenzofuran]-3'-carboxylic acid (**6**) ^{1}H NMR (500 MHz, CD_3OD)

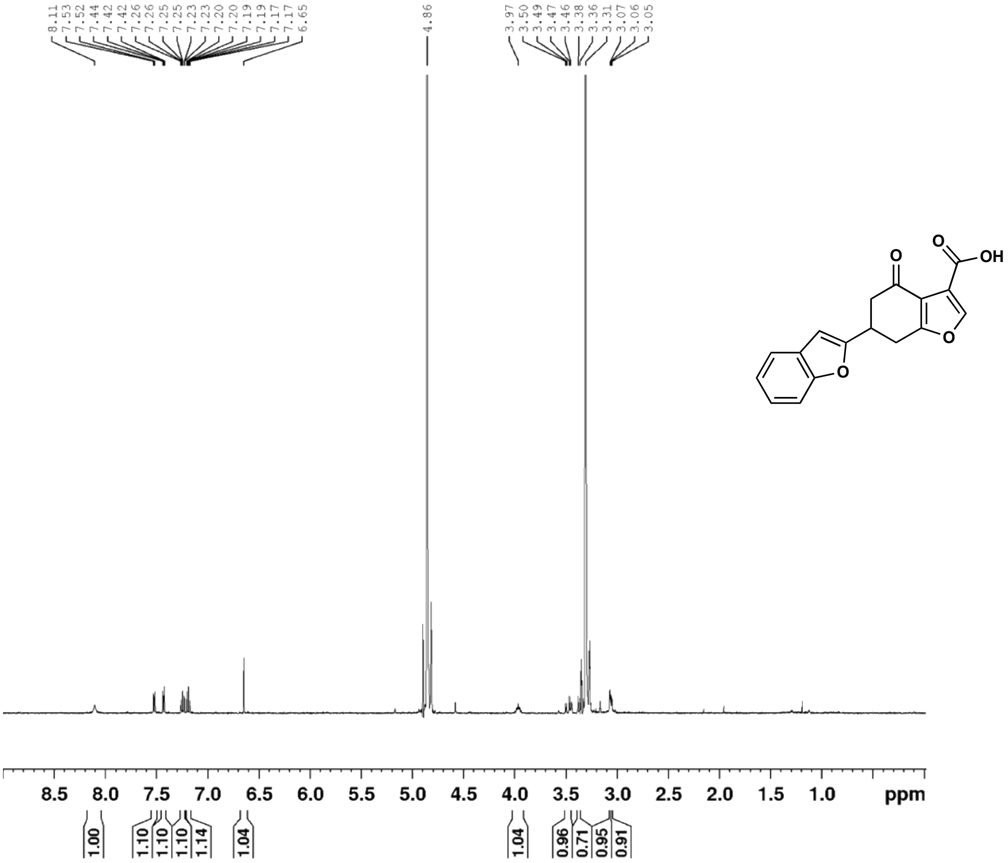

4'-oxo-4',5',6',7'-tetrahydro-[2,6'-bibenzofuran]-3'-carboxylic acid (**6**) ^{13}C DEPT NMR (125 MHz, CD_3OD)

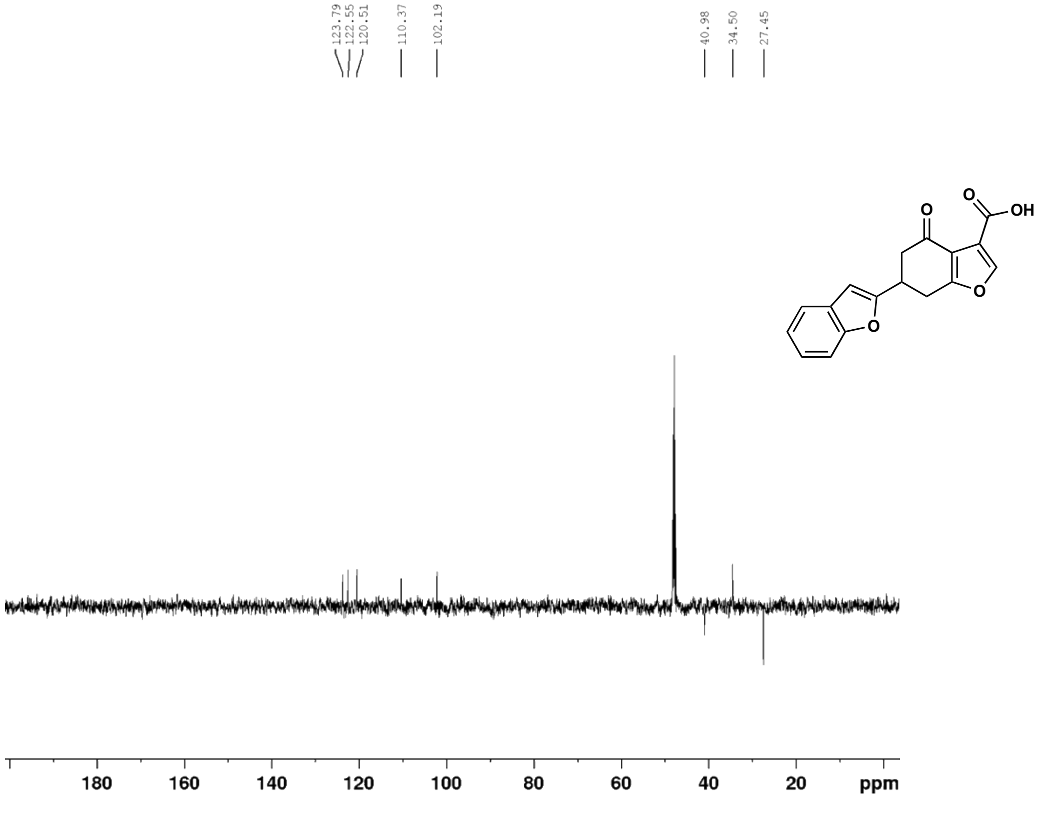

(*E*)-4-(2-bromophenyl)but-3-en-2-one (**7b**) ^{1}H NMR (500 MHz, $CDCl_3$)

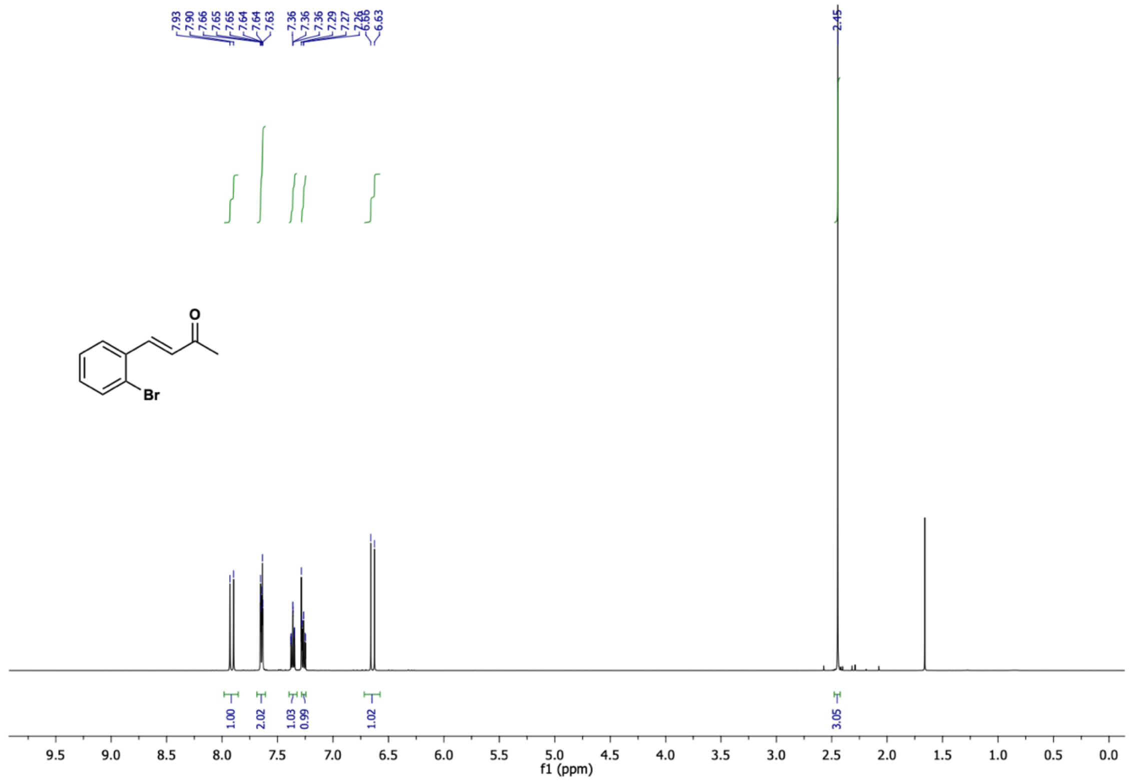

(*E*)-4-(2-bromophenyl)but-3-en-2-one (**7b**) ^{13}C NMR (126 MHz, $CDCl_3$)

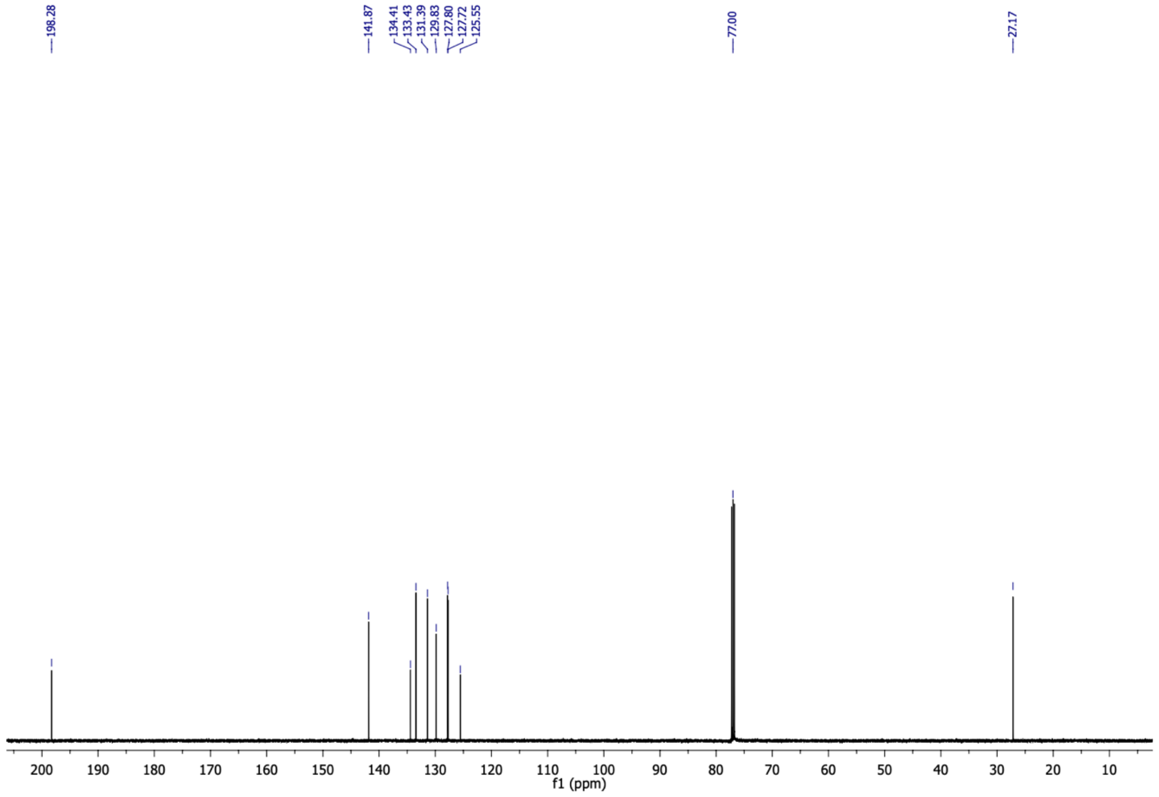

5-(2-bromophenyl)cyclohexane-1,3-dione (**7c**) ^{1}H NMR (500 MHz, CD_3OD)

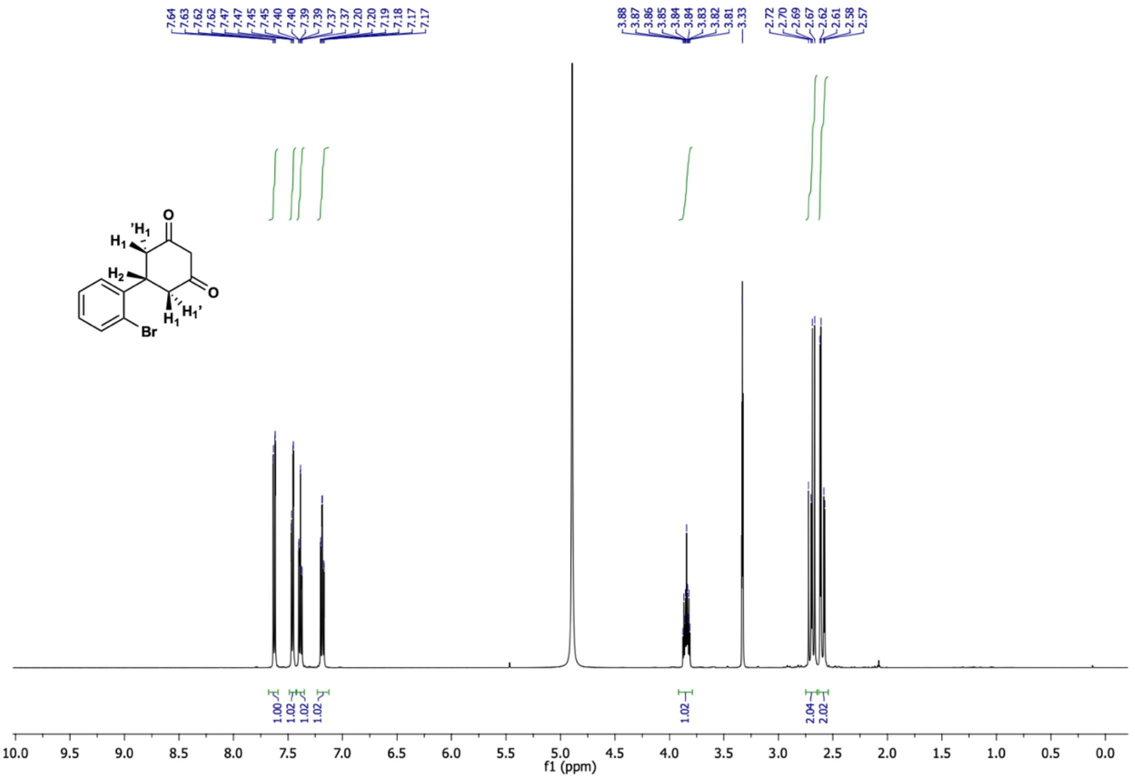

5-(2-bromophenyl)cyclohexane-1,3-dione (**7c**) ^{13}C NMR (126 MHz, CD_3OD)

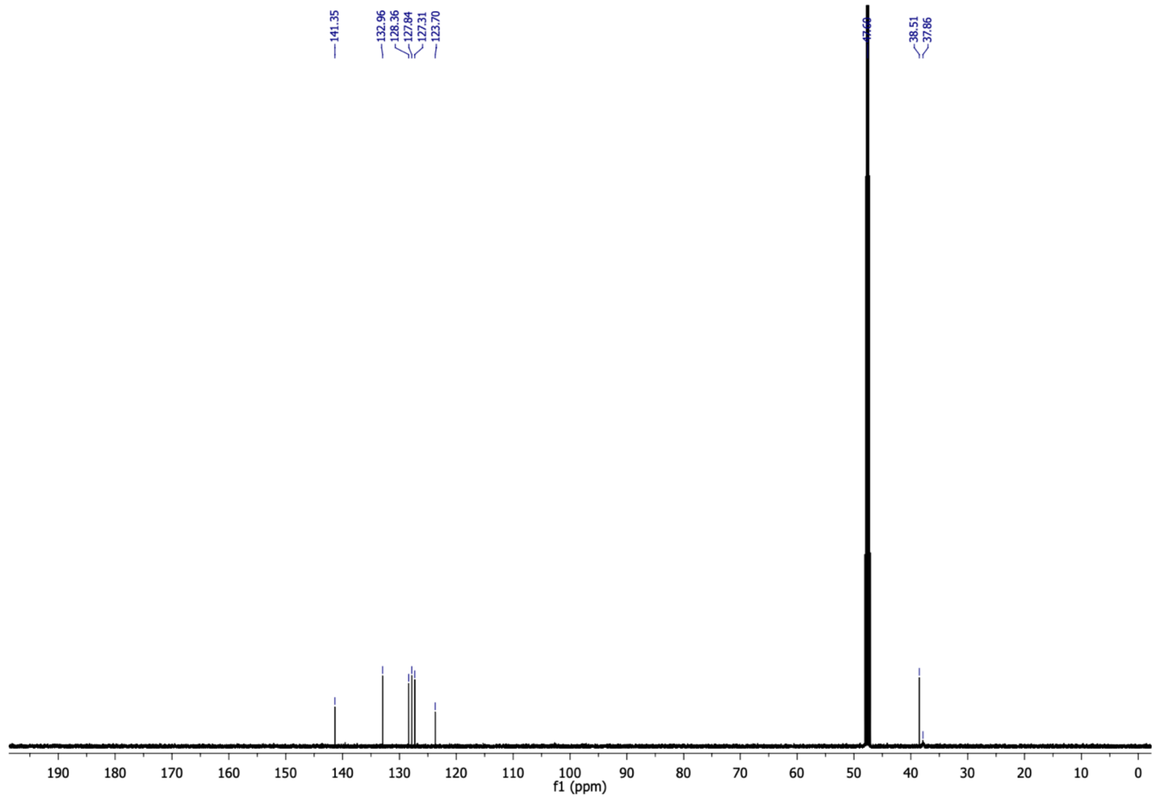

6-(2-bromophenyl)-4-oxo-4,5,6,7-tetrahydrobenzofuran-3-carboxylic acid (**7**) ^{1}H NMR (500 MHz, $CDCl_3$)

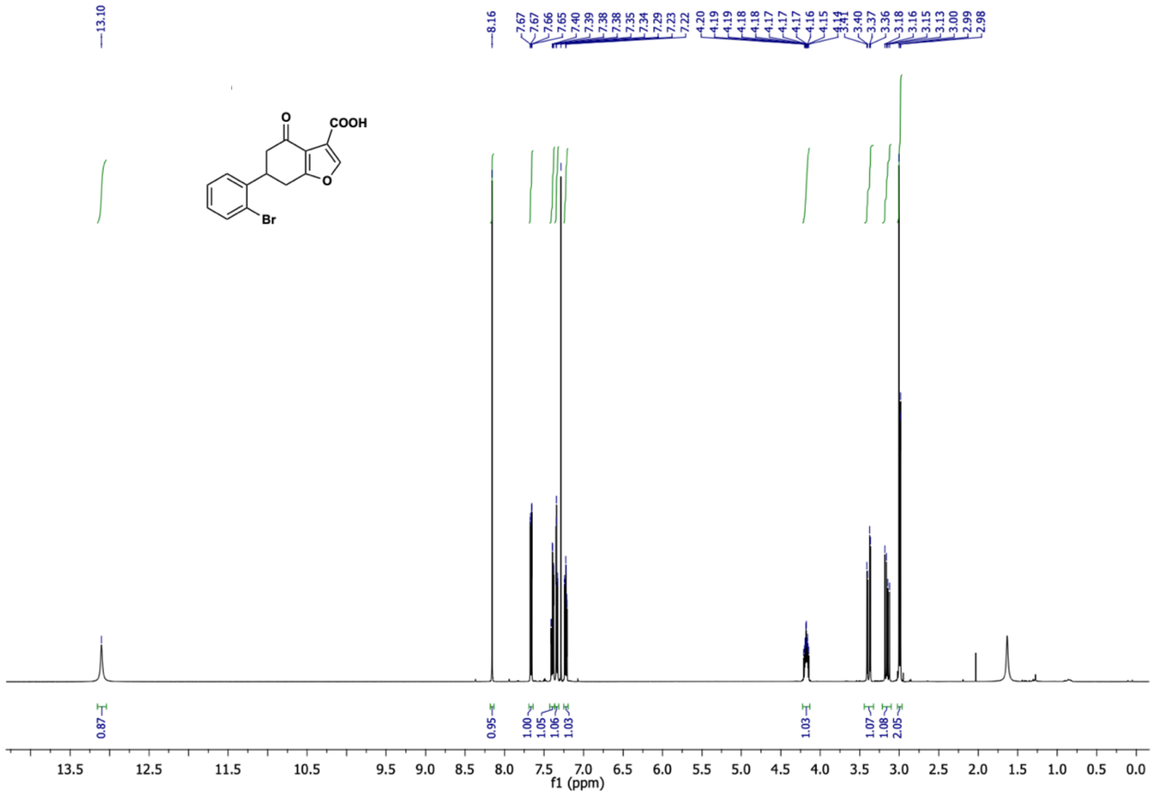

6-(2-bromophenyl)-4-oxo-4,5,6,7-tetrahydrobenzofuran-3-carboxylic acid (**7**) ^{13}C NMR (126 MHz, $CDCl_3$)

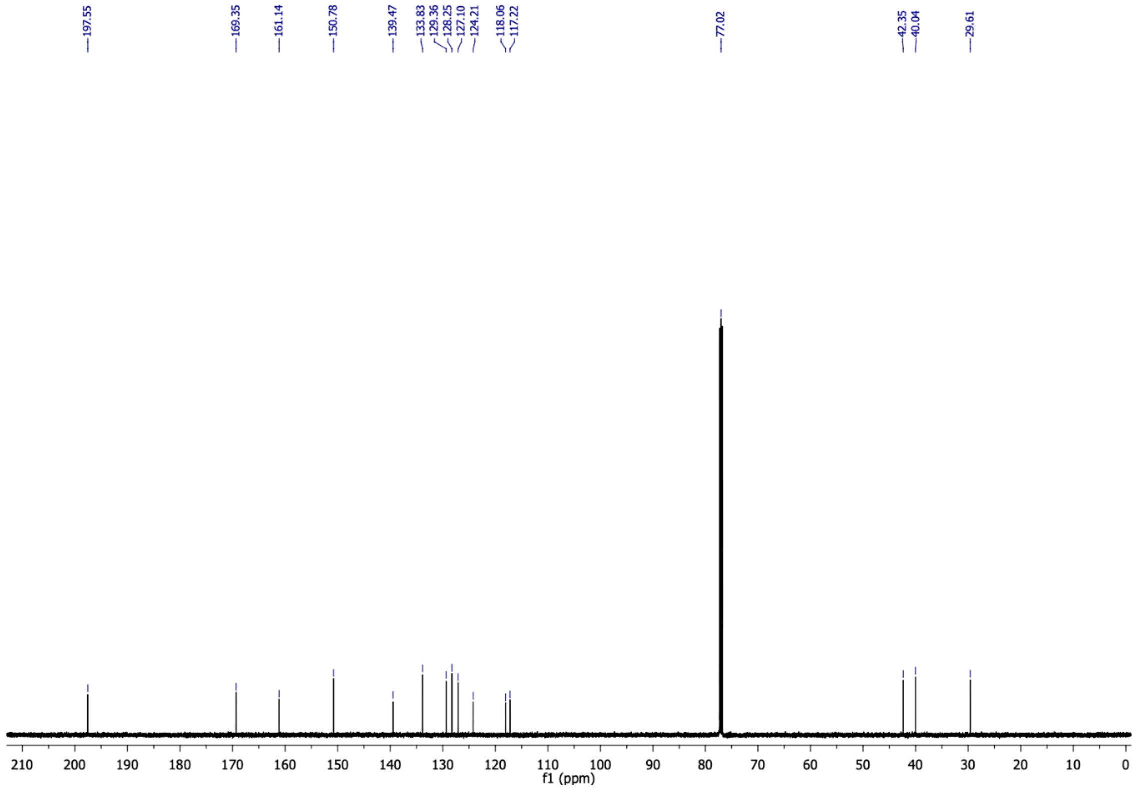

(*E*)-4-(2-fluorophenyl)but-3-en-2-one (**8b**) ^{1}H NMR (500 MHz, $CDCl_3$)

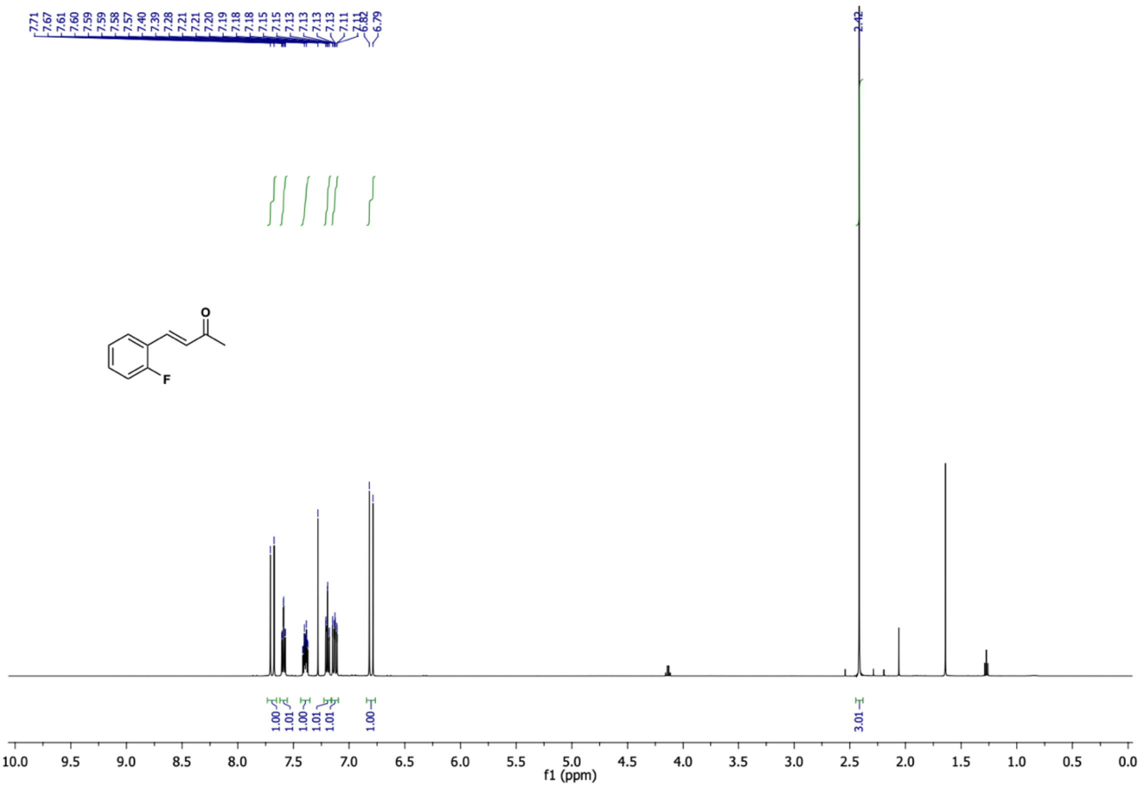

(*E*)-4-(2-fluorophenyl)but-3-en-2-one (**8b**) ^{13}C NMR (126 MHz, $CDCl_3$)

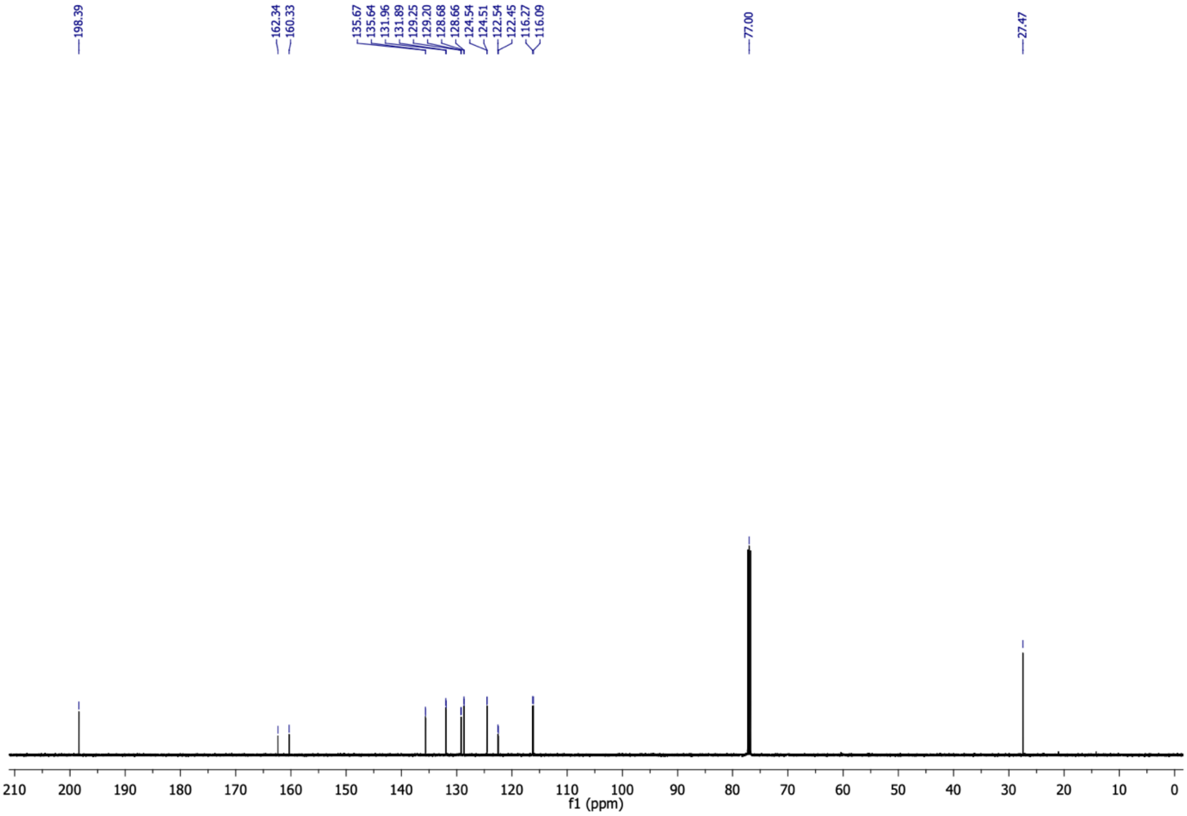

(*E*)-4-(2-fluorophenyl)but-3-en-2-one (**8b**) ^{19}F NMR (376 MHz, $CDCl_3$)

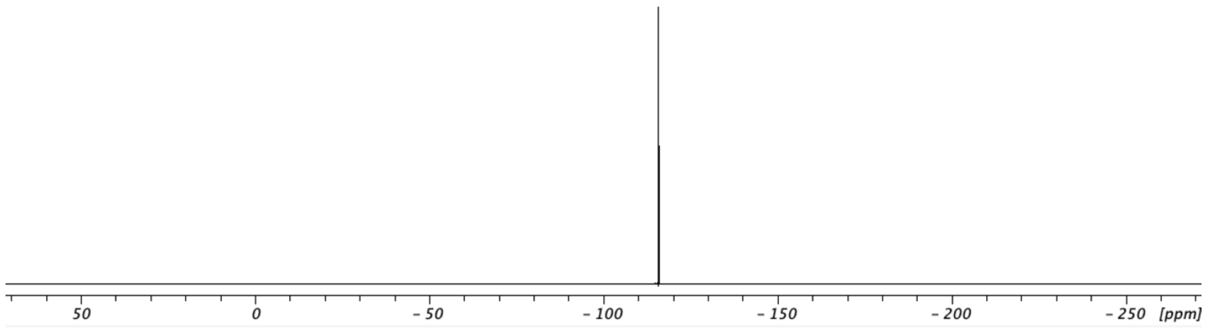

5-(2-fluorophenyl)cyclohexane-1,3-dione (**8c**) ^{1}H NMR (500 MHz, CD_3OD)

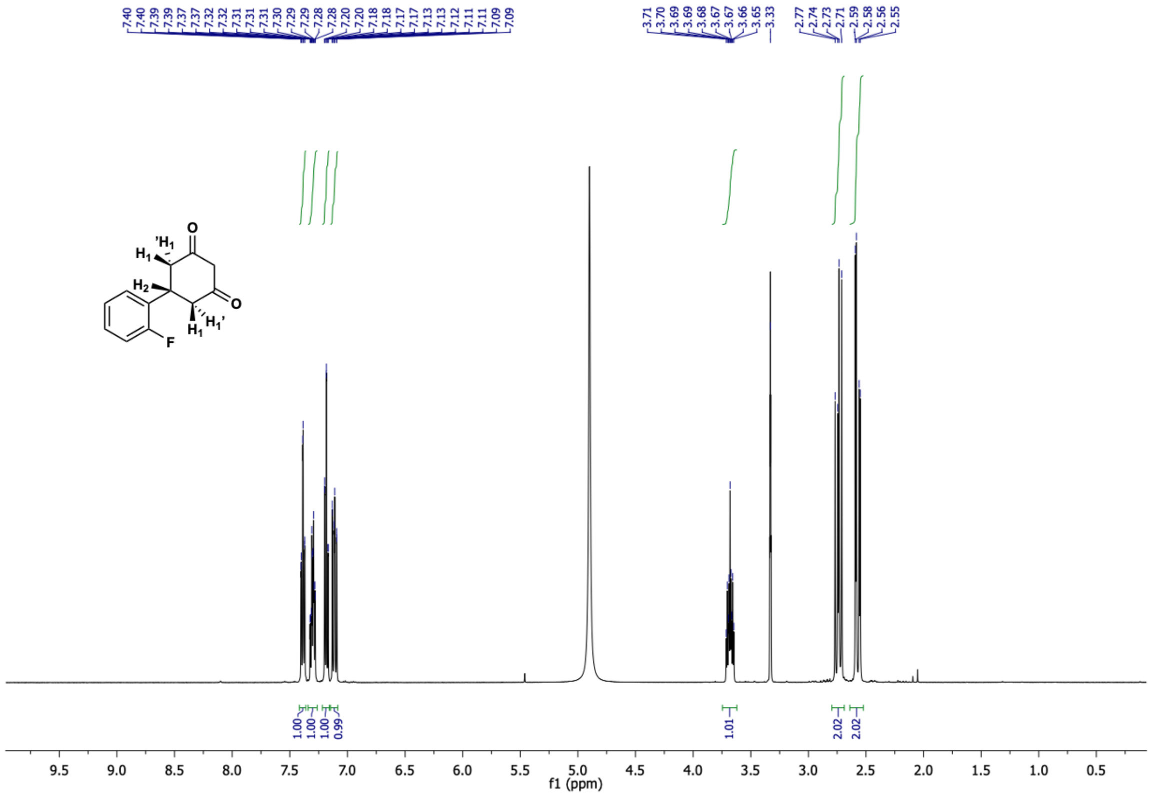

5-(2-fluorophenyl)cyclohexane-1,3-dione (**8c**) ^{13}C NMR (126 MHz, CD_3OD)

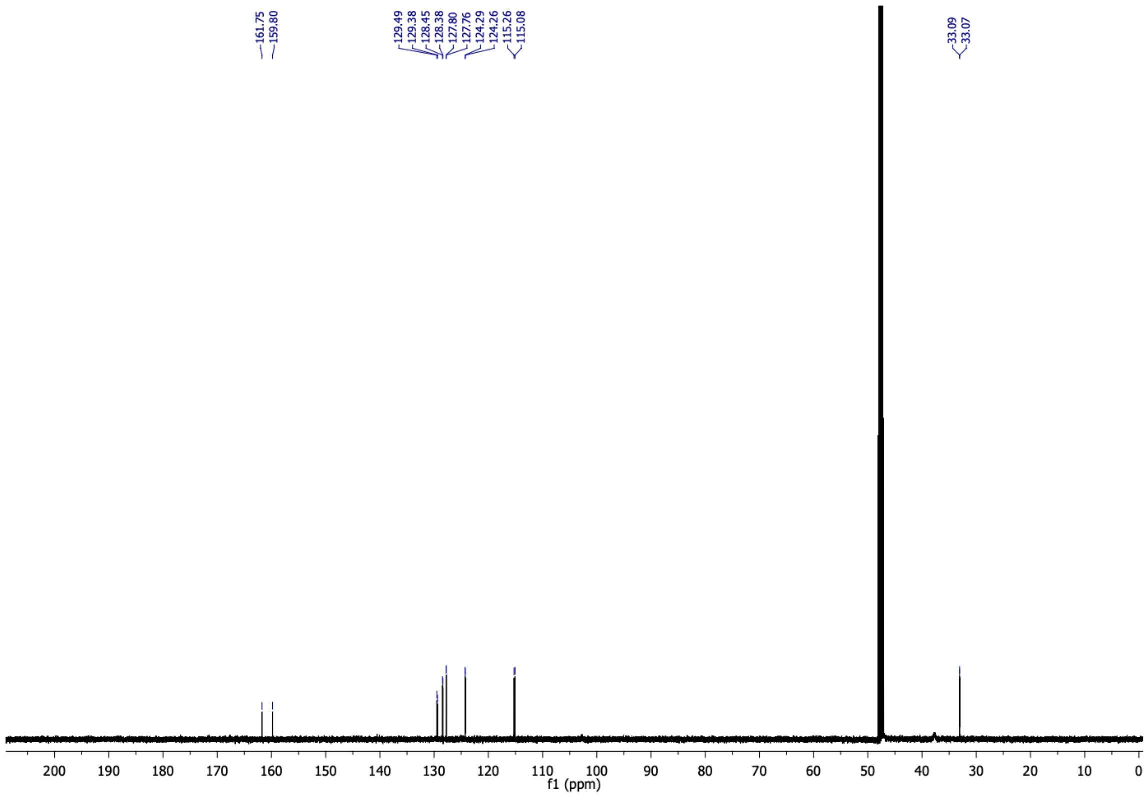

5-(2-fluorophenyl)cyclohexane-1,3-dione (**8c**) ^{19}F NMR (376 MHz, $CDCl_3$)

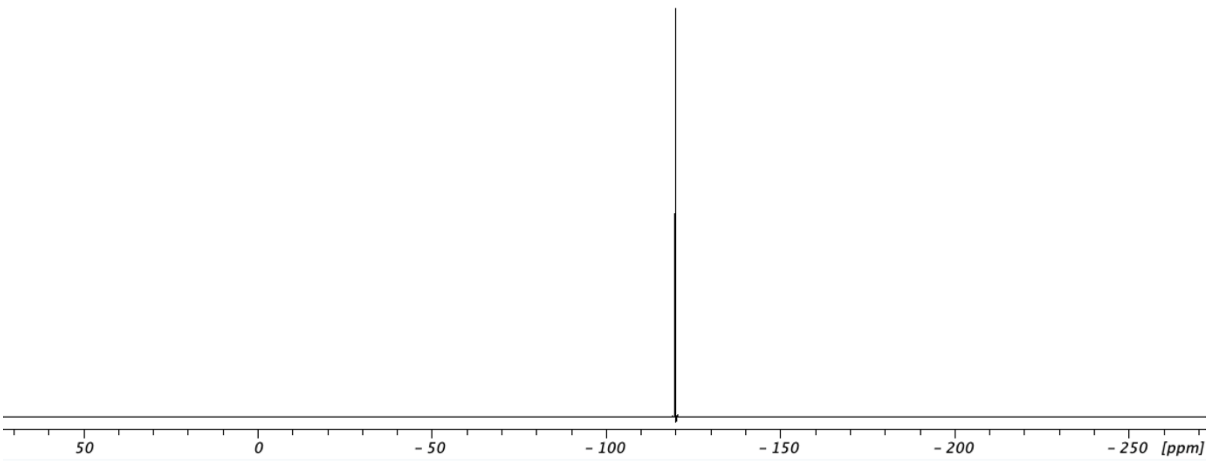

6-(2-fluorophenyl)-4-oxo-4,5,6,7-tetrahydrobenzofuran-3-carboxylic acid (**8**) ^{1}H NMR (500 MHz, $CDCl_3$)

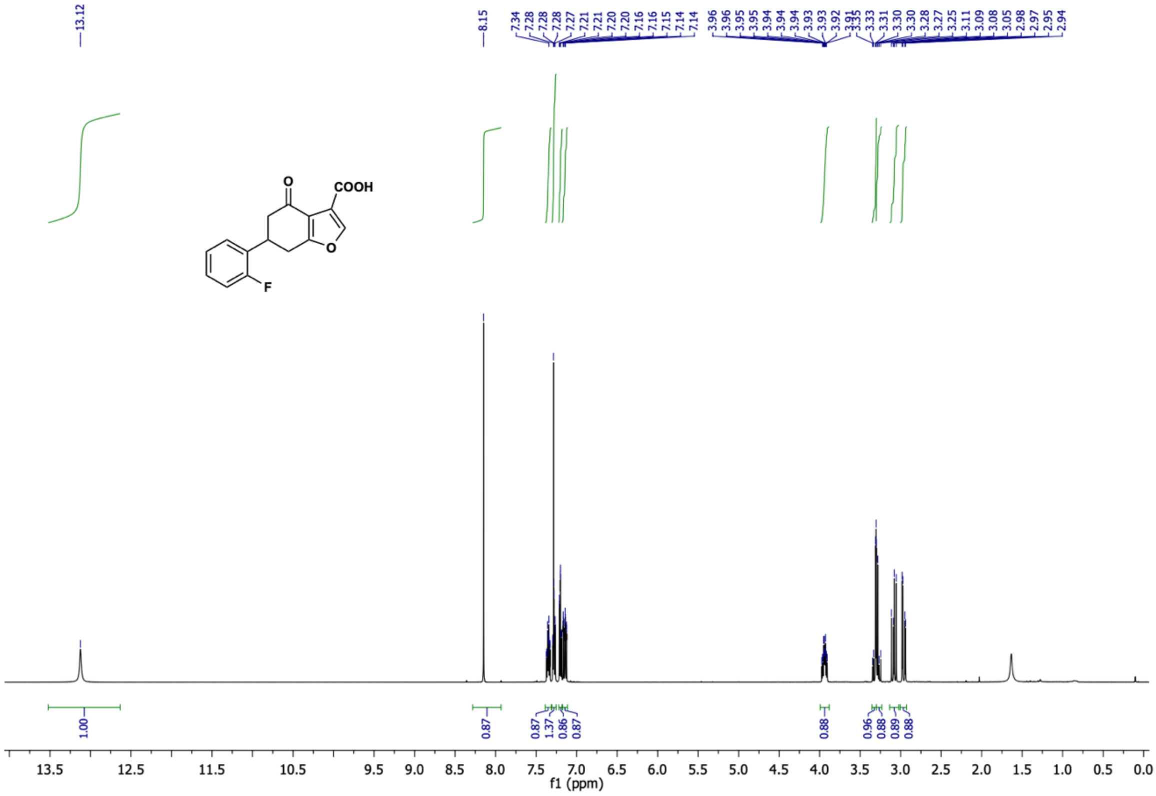

6-(2-fluorophenyl)-4-oxo-4,5,6,7-tetrahydrobenzofuran-3-carboxylic acid (**8**) ^{13}C NMR (126 MHz, $CDCl_3$)

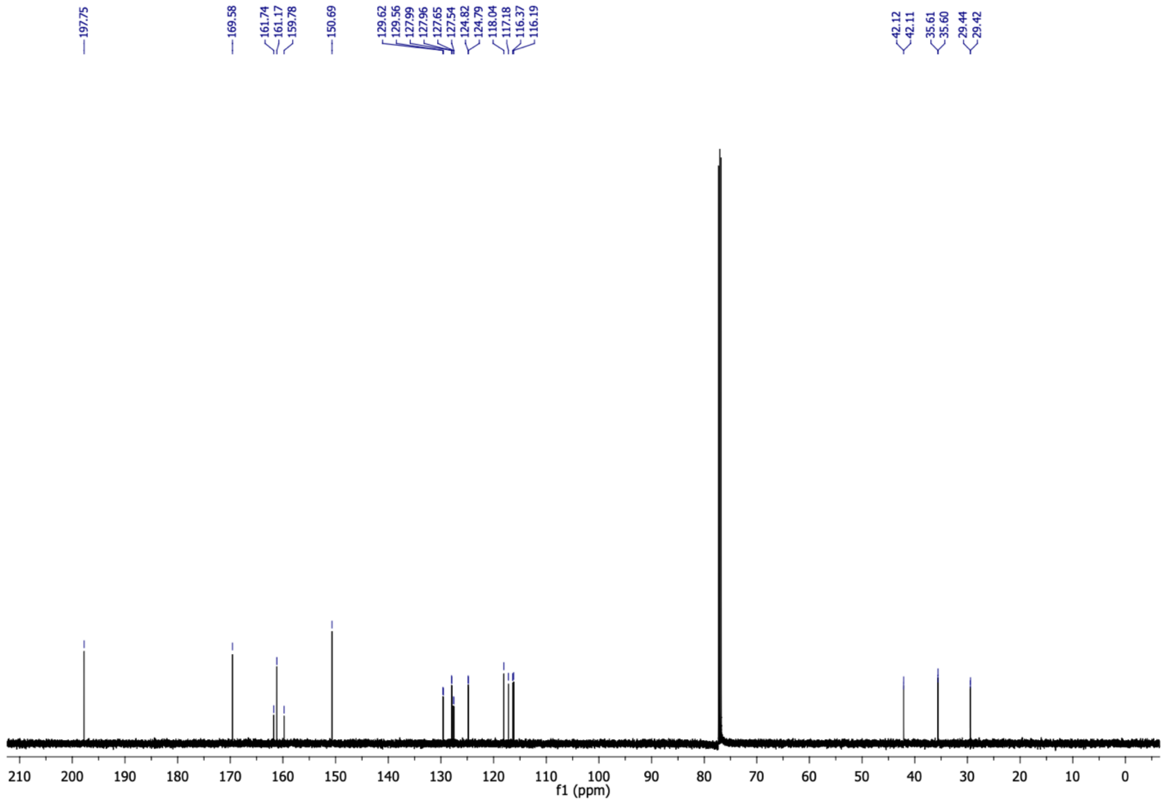

6-(2-fluorophenyl)-4-oxo-4,5,6,7-tetrahydrobenzofuran-3-carboxylic acid (**8**) ^{19}F NMR (376 MHz, $CDCl_3$)

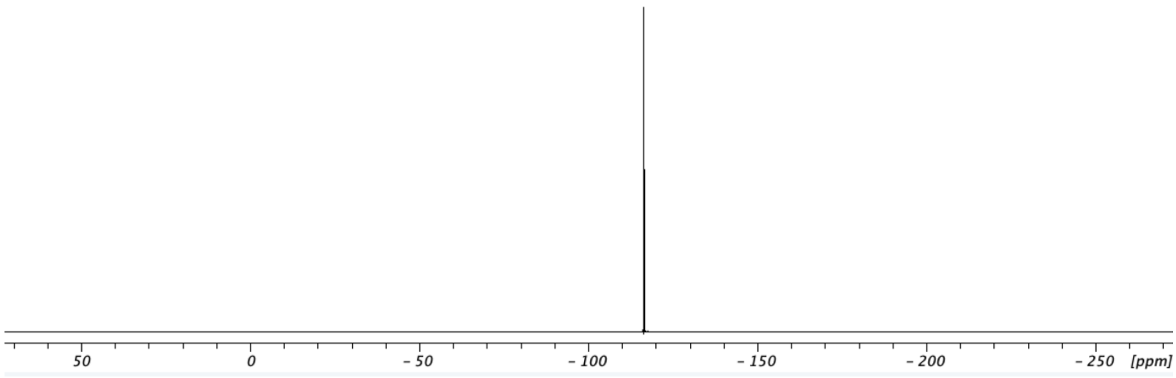

(*E*)-4-(2-phenoxyphenyl)but-3-en-2-one (**9b**) ^{1}H NMR (500 MHz, $CDCl_3$)

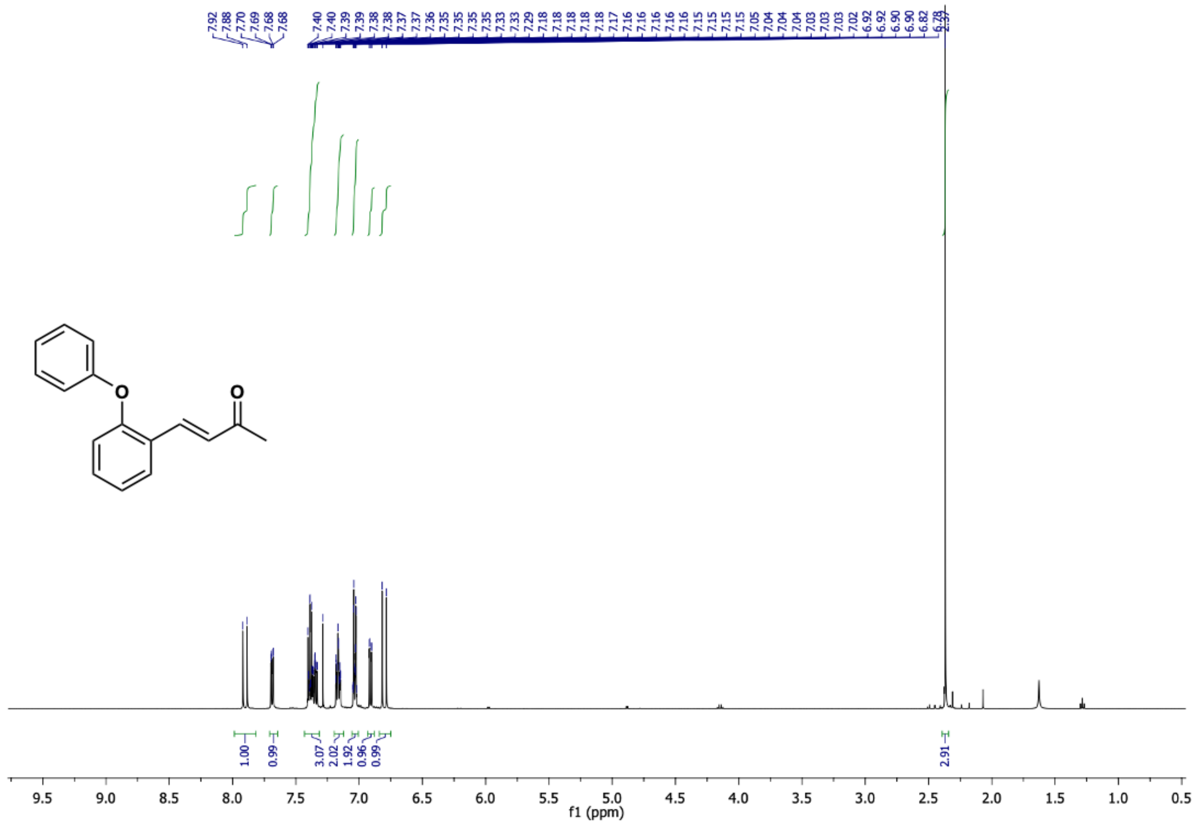

(*E*)-4-(2-phenoxyphenyl)but-3-en-2-one (**9b**) ^{13}C NMR (126 MHz, $CDCl_3$)

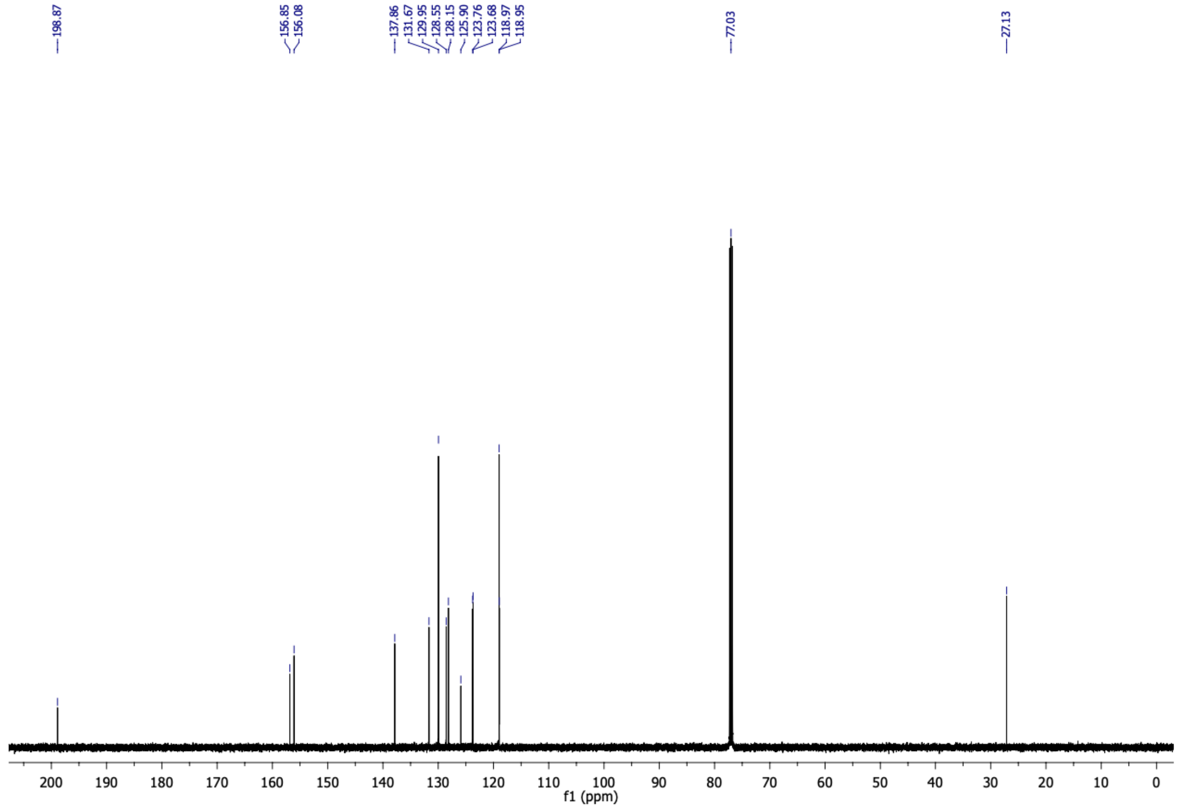

5-(2-phenoxyphenyl)cyclohexane-1,3-dione (**9c**) ^{1}H NMR (500 MHz, CD_3OD)

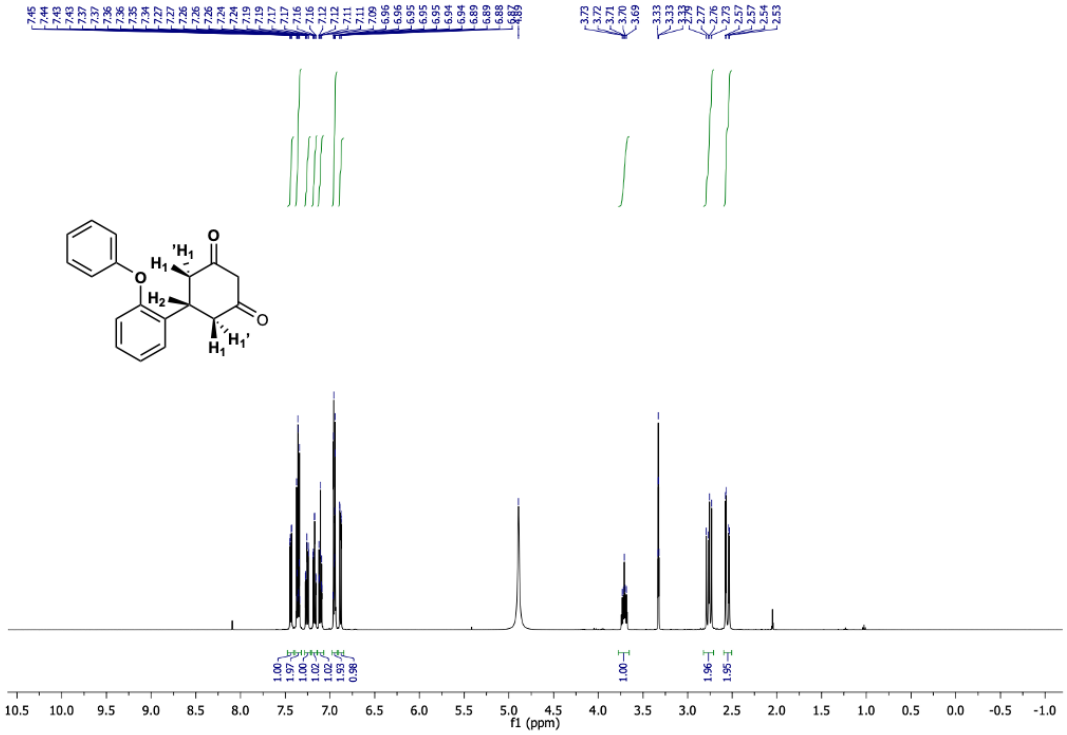

5-(2-phenoxyphenyl)cyclohexane-1,3-dione (**9c**) ^{13}C NMR (126 MHz, CD_3OD)

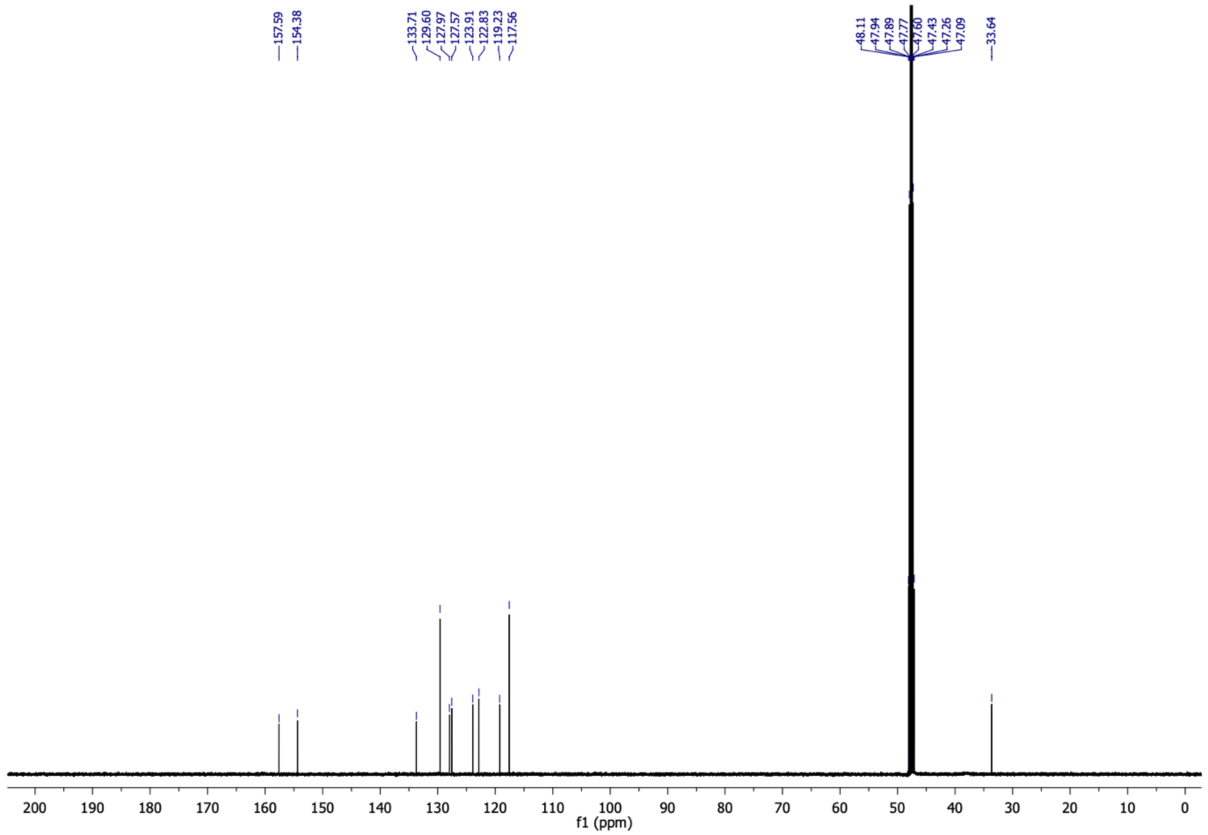

4-oxo-6-(2-phenoxyphenyl)-4,5,6,7-tetrahydrobenzofuran-3-carboxylic acid (**9**) ^{1}H NMR (400 MHz, $CDCl_3$)

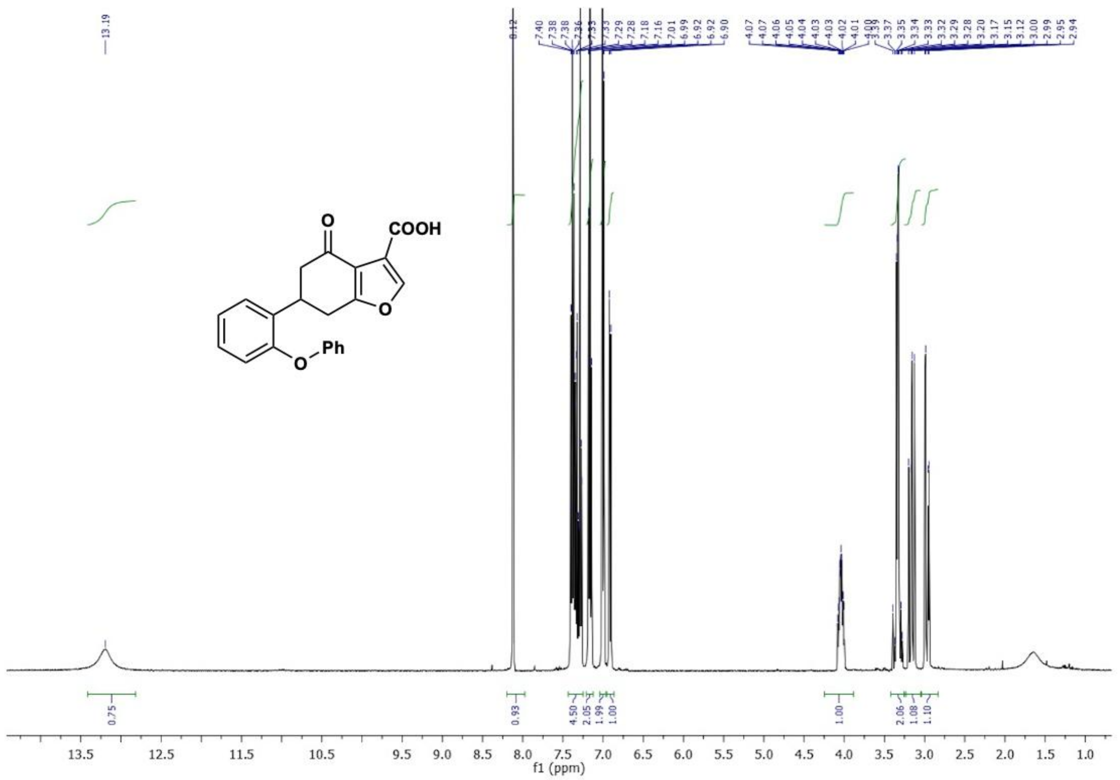

4-oxo-6-(2-phenoxyphenyl)-4,5,6,7-tetrahydrobenzofuran-3-carboxylic acid (**9**) ^{13}C NMR (101 MHz, $CDCl_3$)

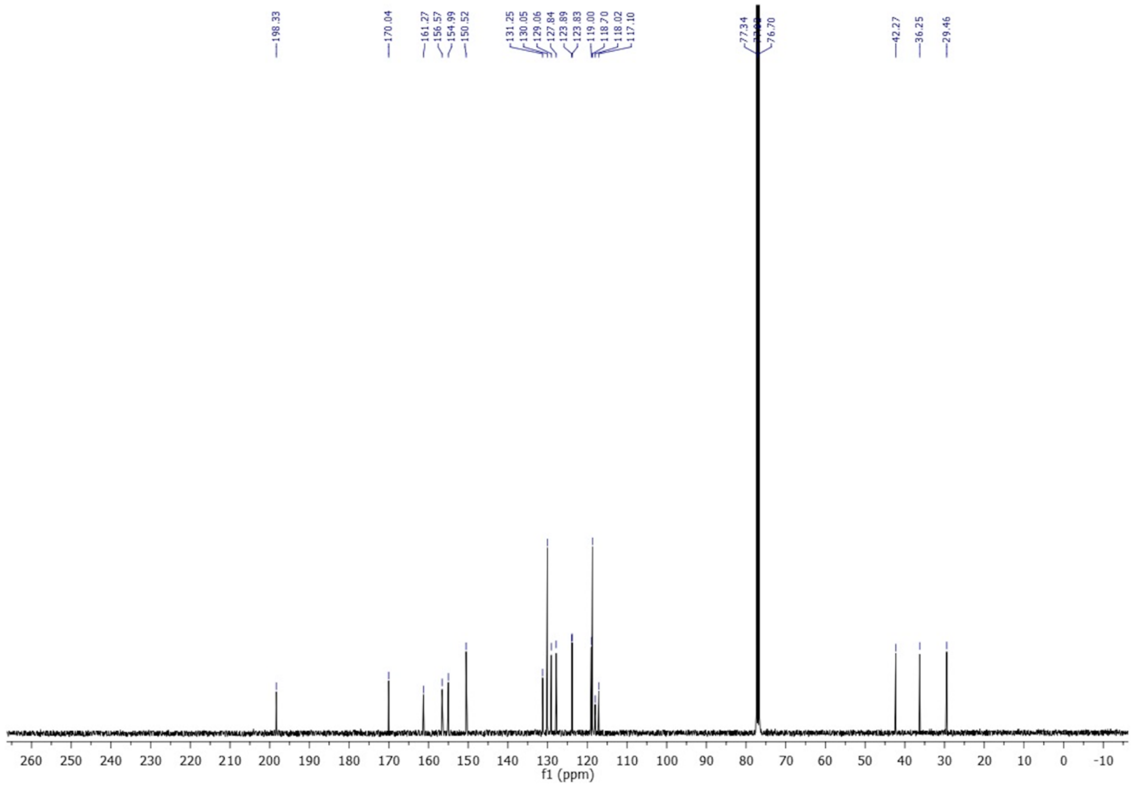

2-isopropoxybenzaldehyde (**10a**) ^{1}H NMR (500 MHz, $CDCl_3$)

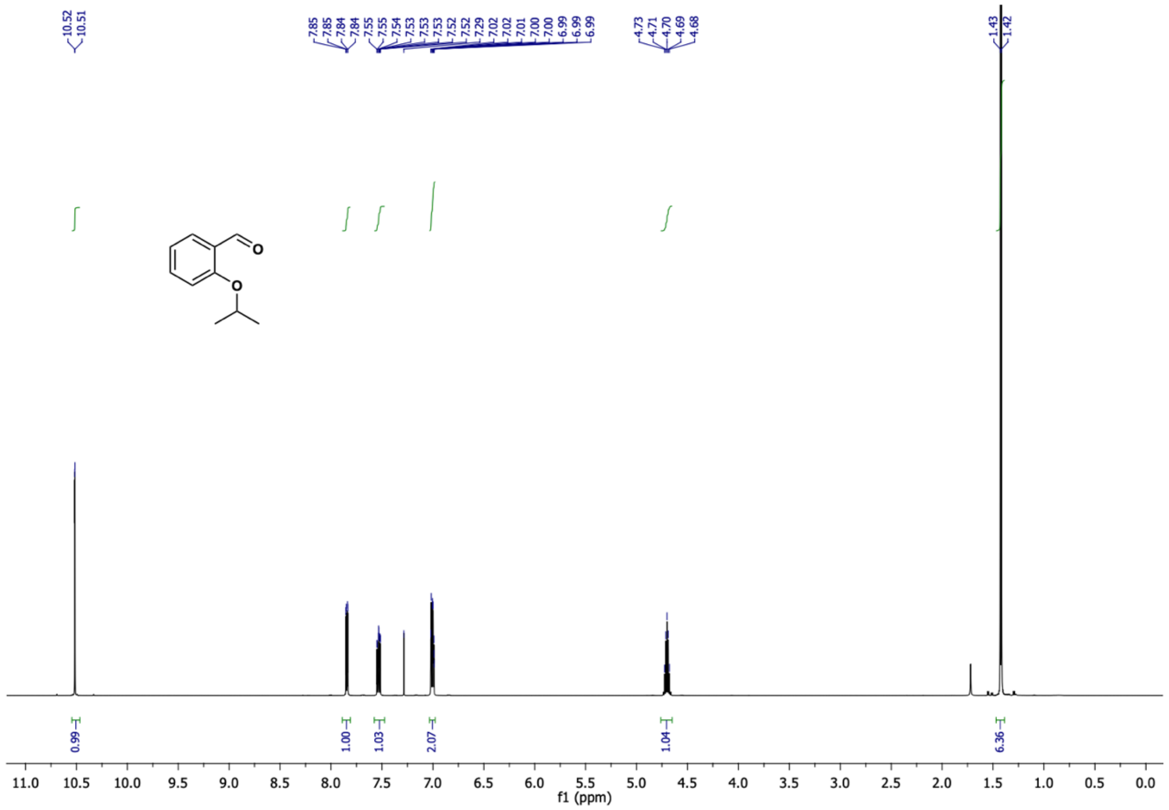

2-isopropoxybenzaldehyde (**10a**) ^{13}C NMR (126 MHz, $CDCl_3$)

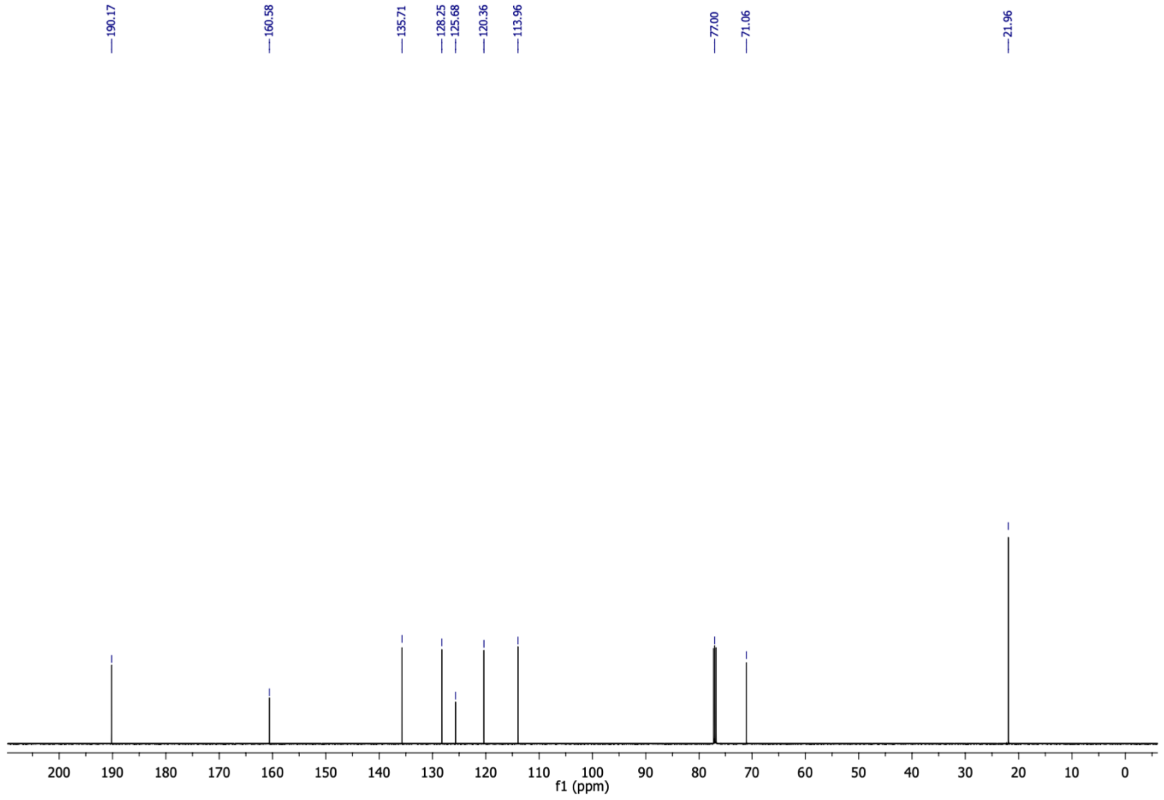

(E)-4-(2-isopropoxyphenyl)but-3-en-2-one (**10b**) ^{1}H NMR (500 MHz, $CDCl_3$)

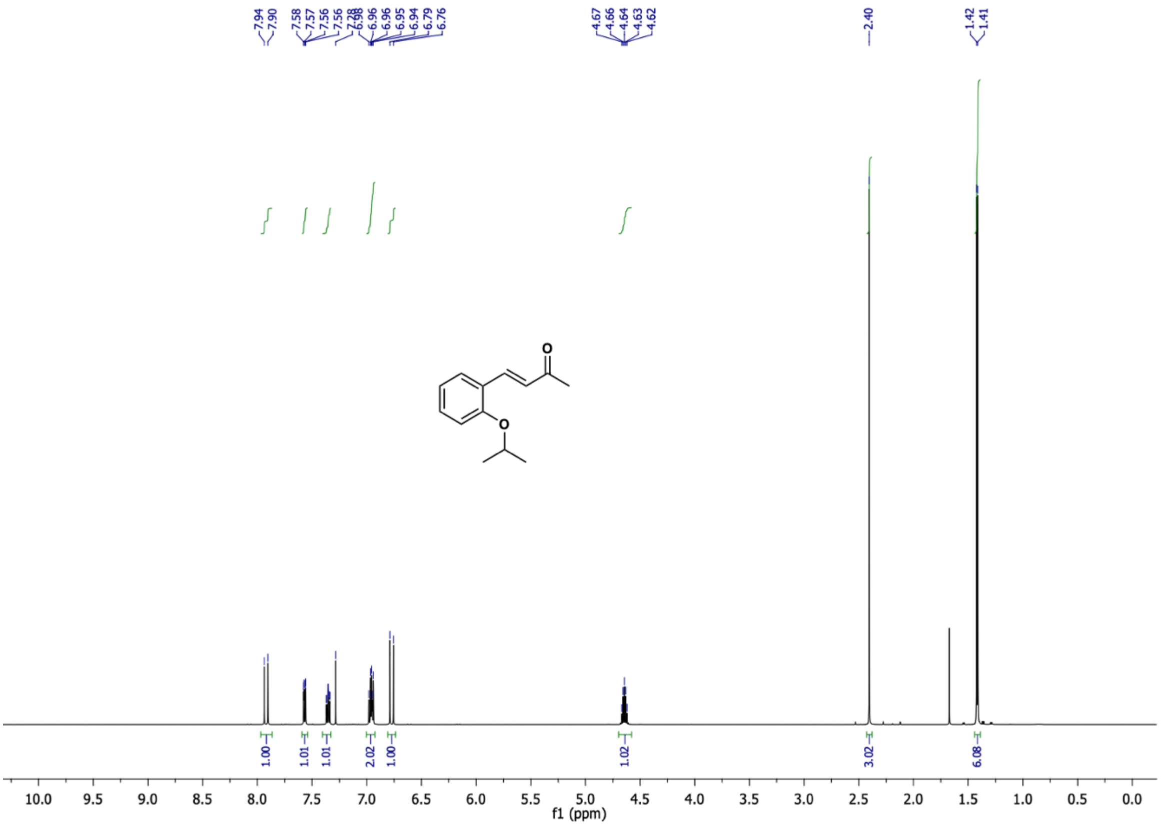

(E)-4-(2-isopropoxyphenyl)but-3-en-2-one (**10b**) ^{13}C NMR (126 MHz, $CDCl_3$)

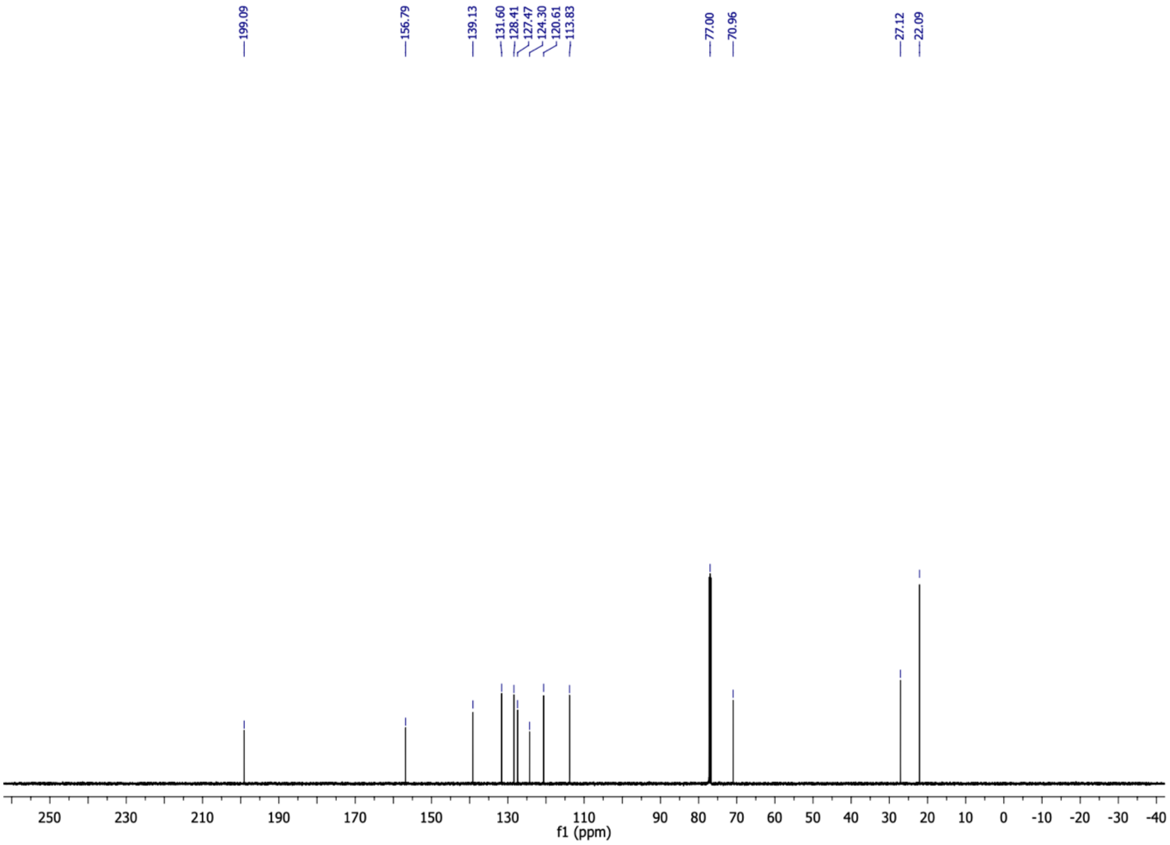

5-(2-isopropoxyphenyl)cyclohexane-1,3-dione (**10c**) ^{1}H NMR (500 MHz, CD_3OD)

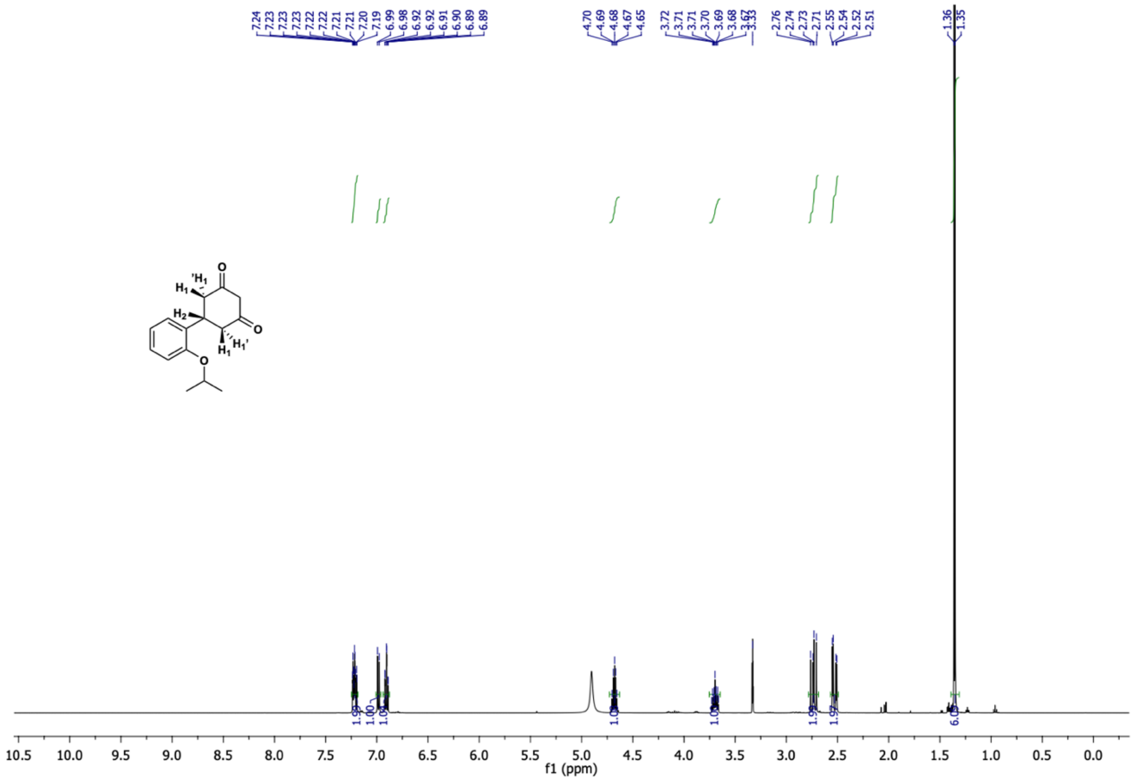

5-(2-isopropoxyphenyl)cyclohexane-1,3-dione (**10c**) ^{13}C NMR (126 MHz, CD_3OD)

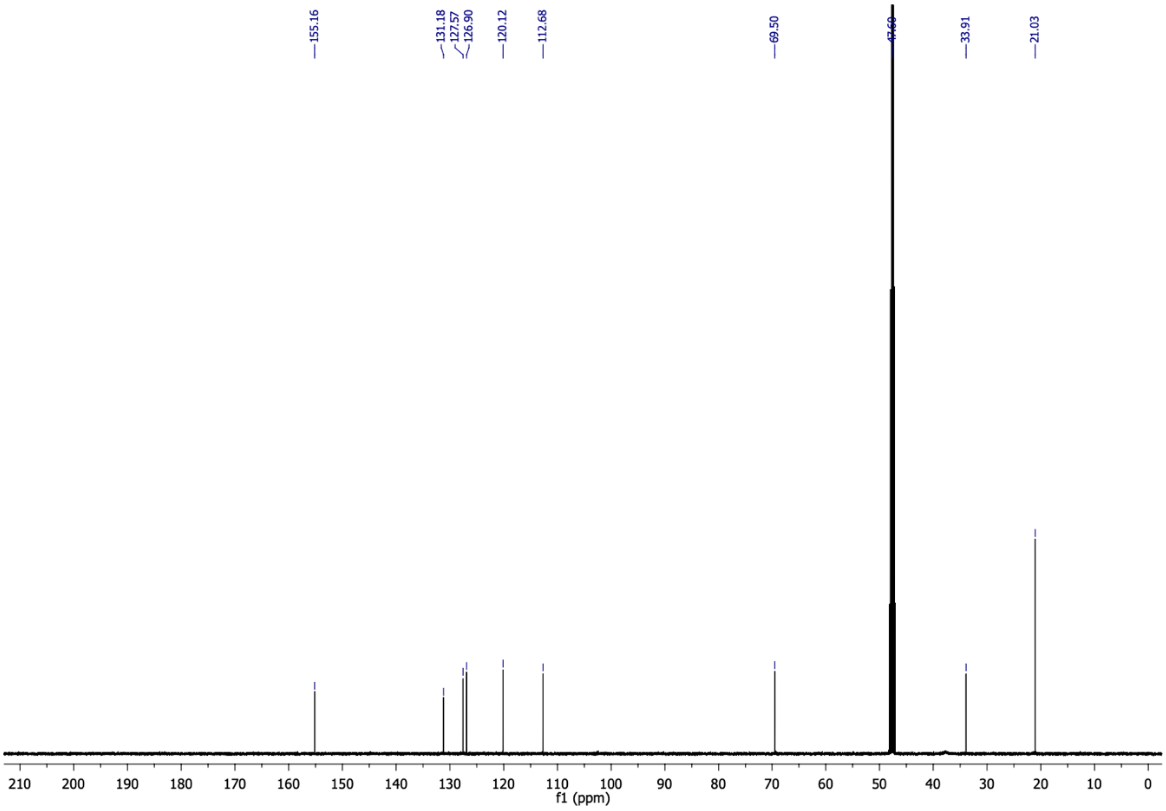

6-(2-isopropoxyphenyl)-4-oxo-4,5,6,7-tetrahydrobenzofuran-3-carboxylic acid (**10**) ^{1}H NMR (500 MHz, $CDCl_3$)

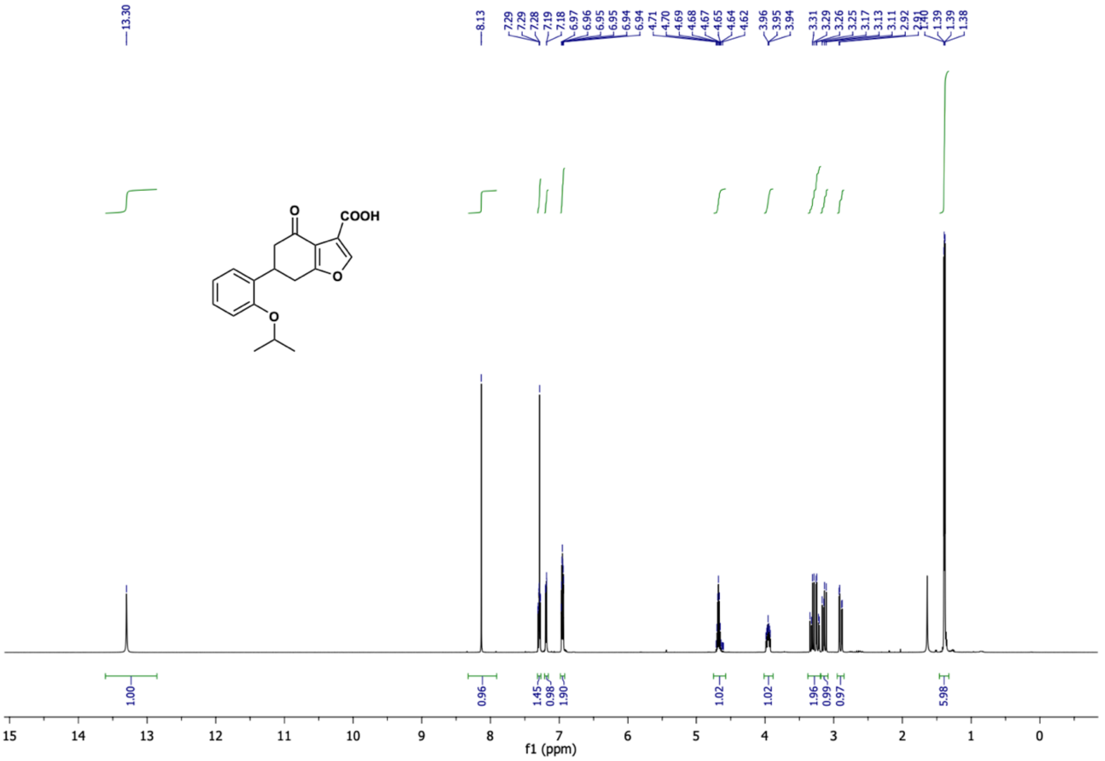

6-(2-isopropoxyphenyl)-4-oxo-4,5,6,7-tetrahydrobenzofuran-3-carboxylic acid (**10**) ^{13}C NMR (126 MHz, $CDCl_3$)

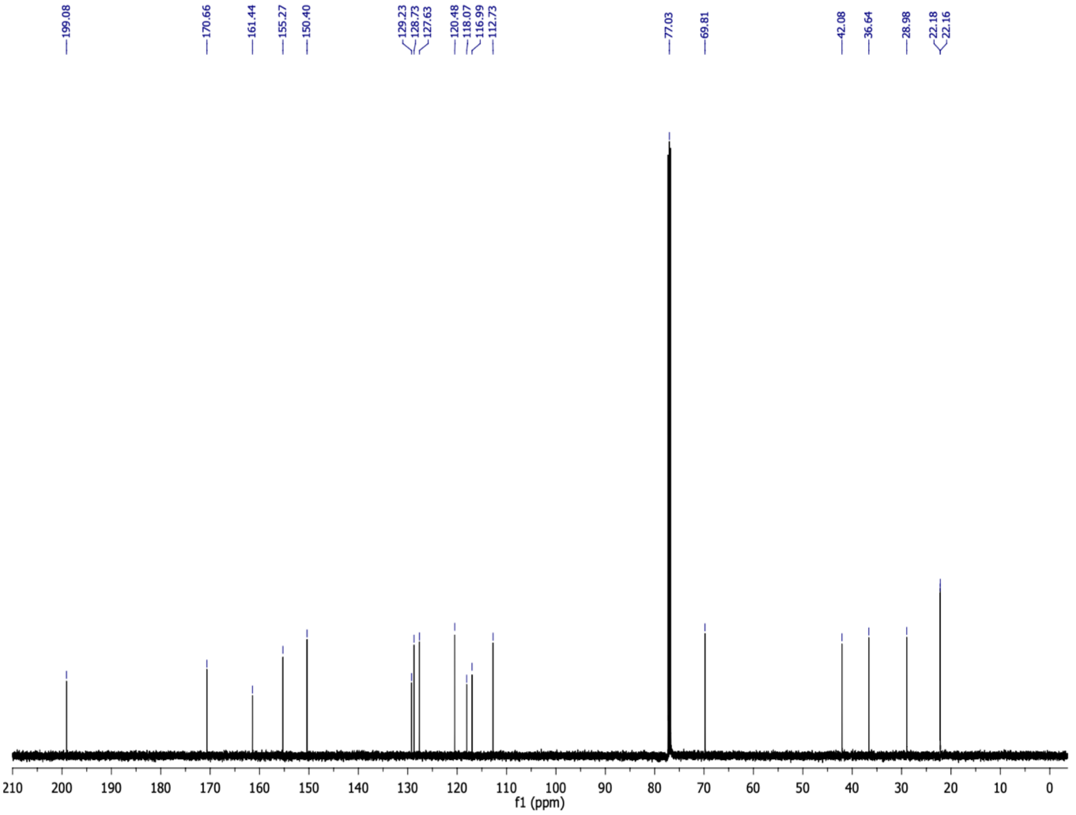

(*E*)-4-(2-methoxyphenyl)but-3-en-2-one (**11b**) ^{1}H NMR (500 MHz, $CDCl_3$)

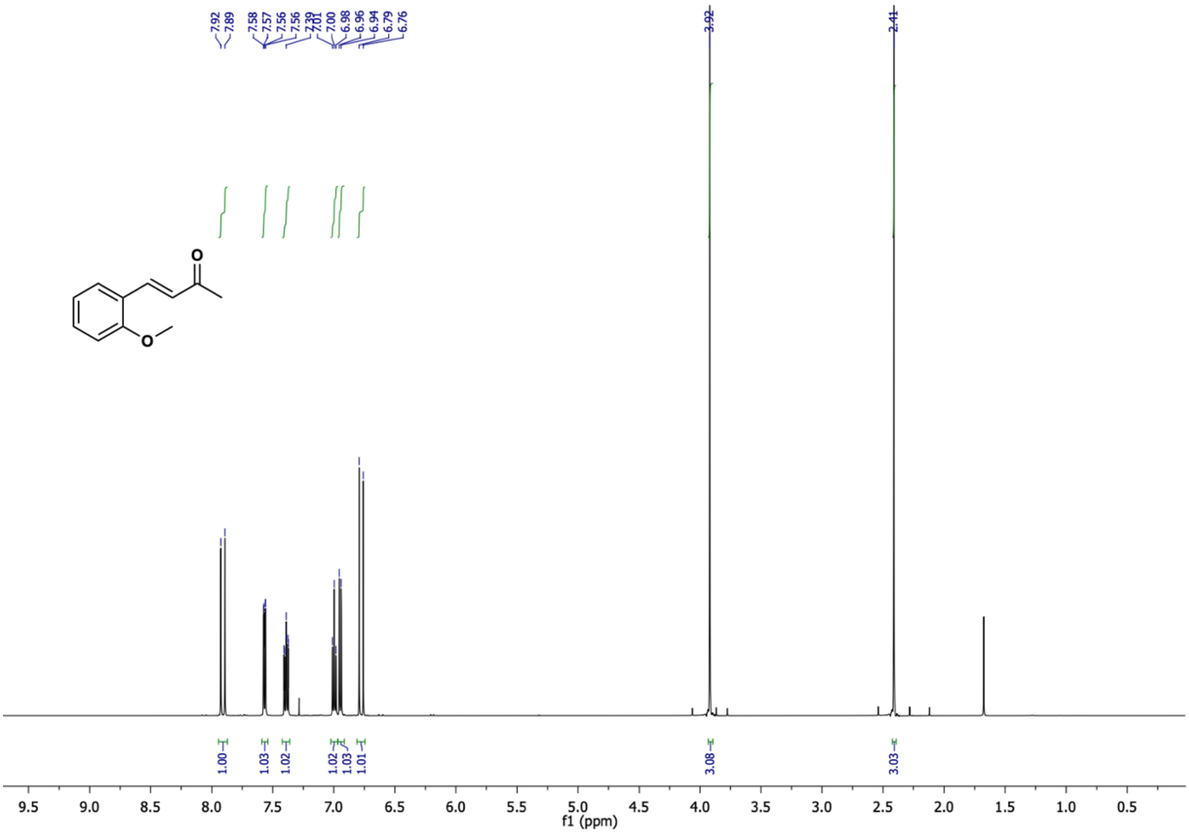

(*E*)-4-(2-methoxyphenyl)but-3-en-2-one (**11b**) ^{13}C NMR (126 MHz, $CDCl_3$)

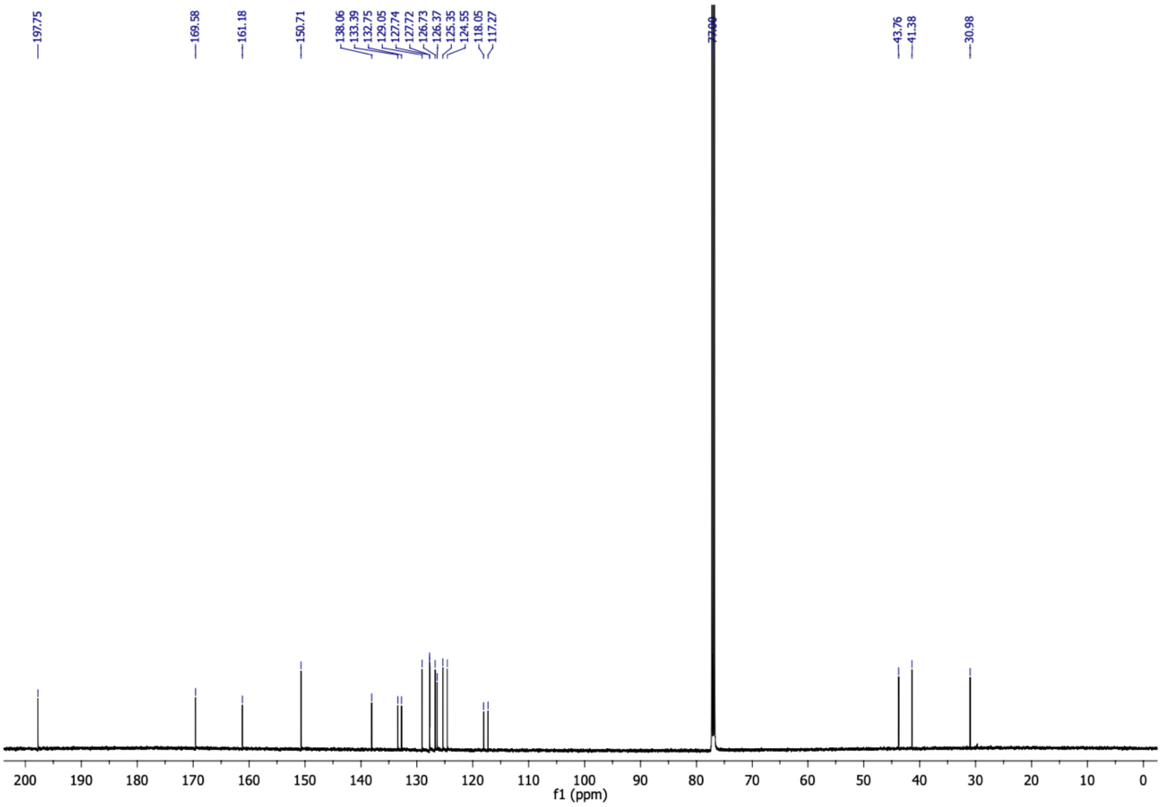

5-(2-methoxyphenyl)cyclohexane-1,3-dione (**11c**) ^{1}H NMR (500 MHz, CD_3OD)

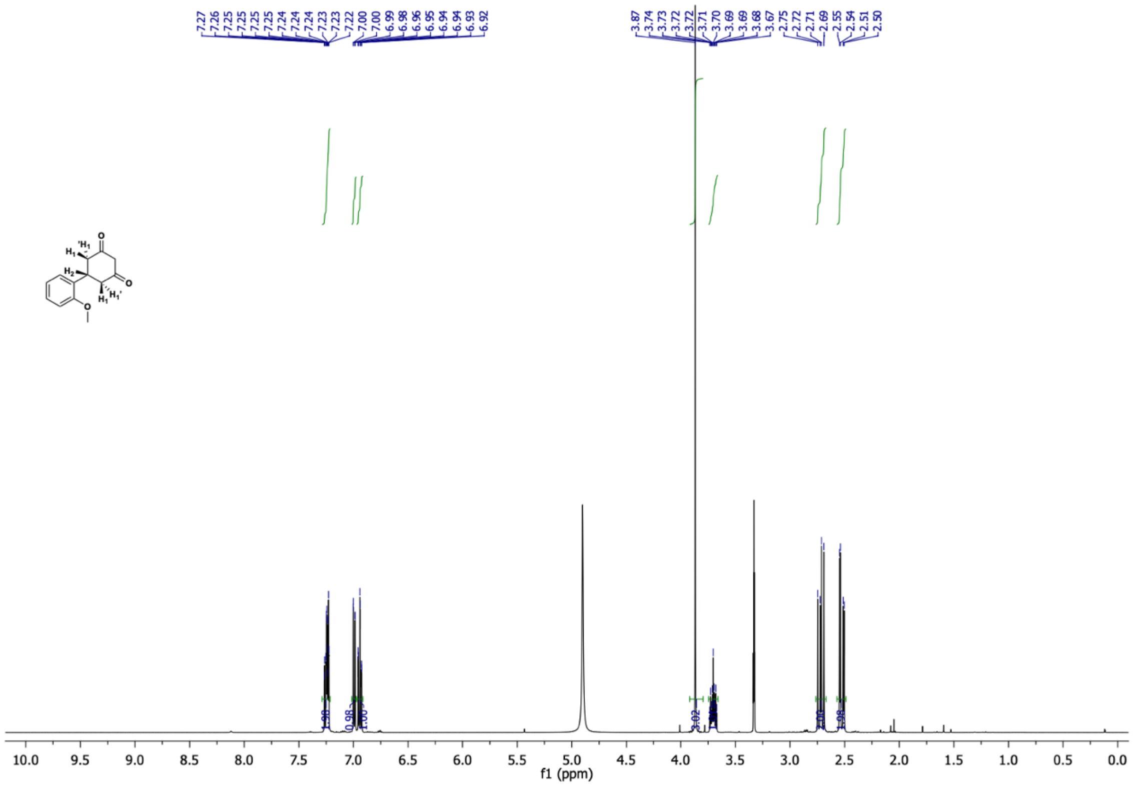

5-(2-methoxyphenyl)cyclohexane-1,3-dione (**11c**) ^{13}C NMR (126 MHz, CD_3OD)

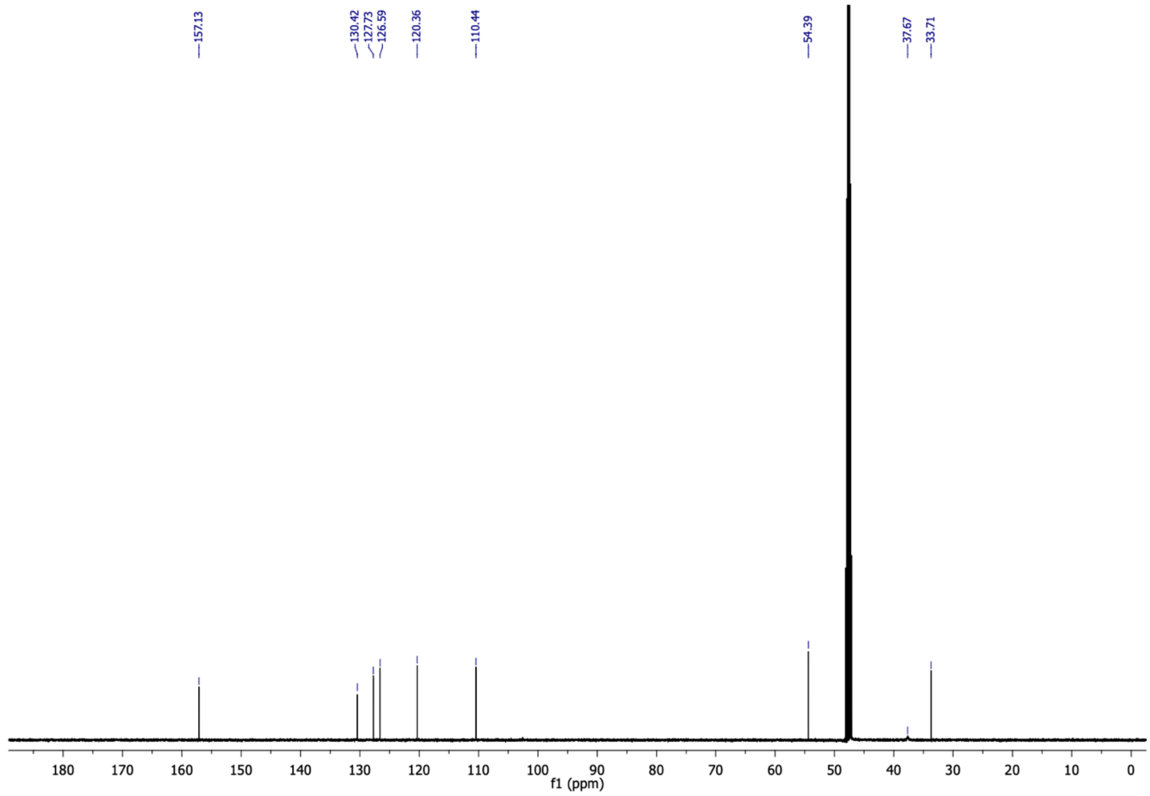

6-(2-methoxyphenyl)-4-oxo-4,5,6,7-tetrahydrobenzofuran-3-carboxylic acid (**11**) ^{1}**H NMR** (500 MHz, $CDCl_3$)

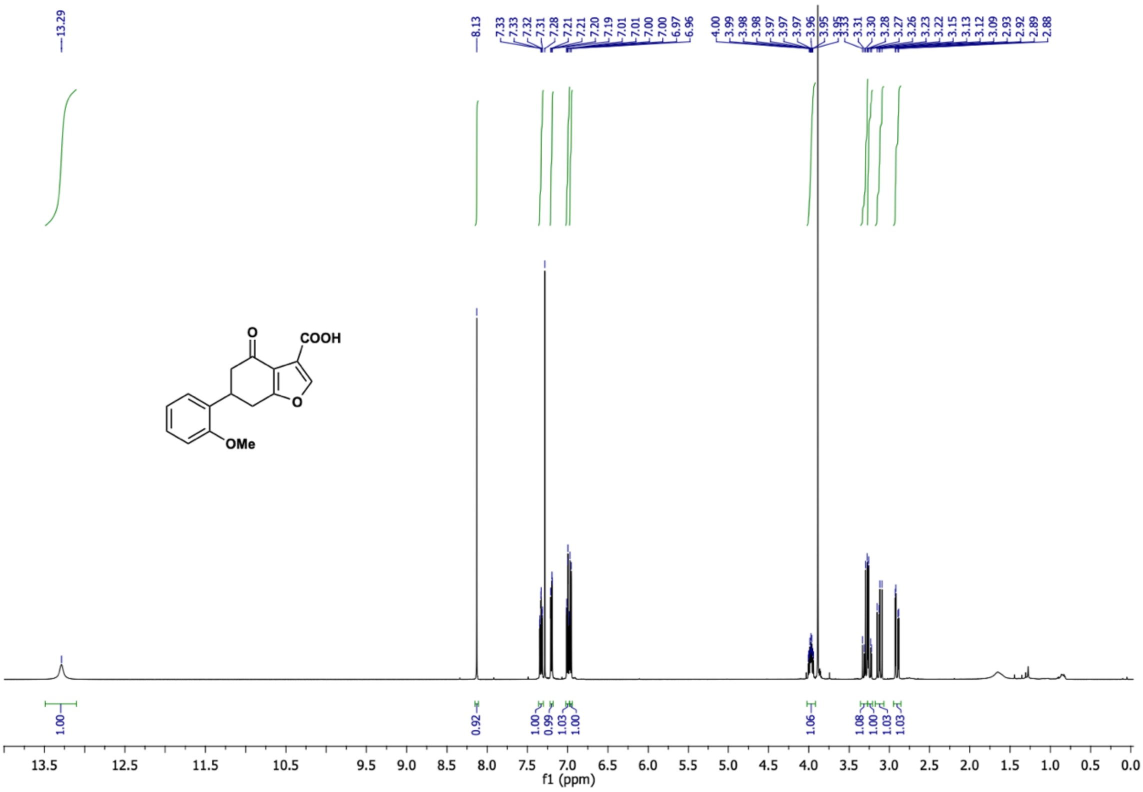

6-(2-methoxyphenyl)-4-oxo-4,5,6,7-tetrahydrobenzofuran-3-carboxylic acid (**11**) ^{13}C NMR (126 MHz, $CDCl_3$)

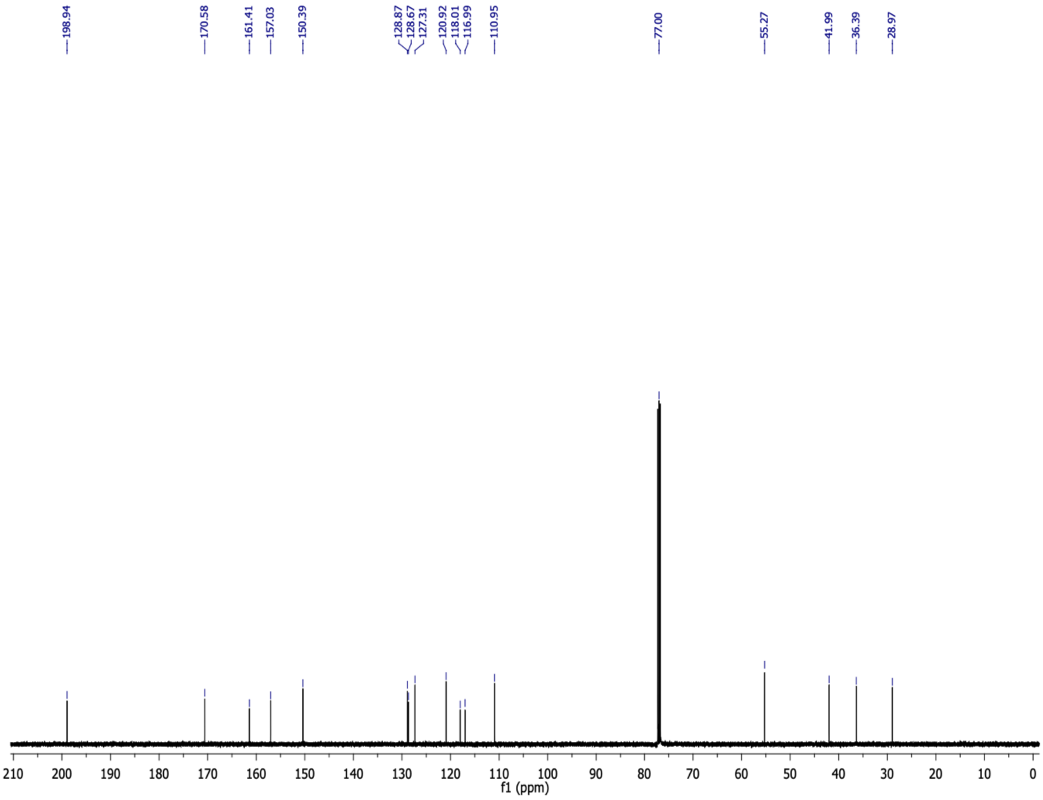

(*E*)-4-(pyridin-2-yl)but-3-en-2-one (**12b**) ^{1}H NMR (500 MHz, $CDCl_3$)

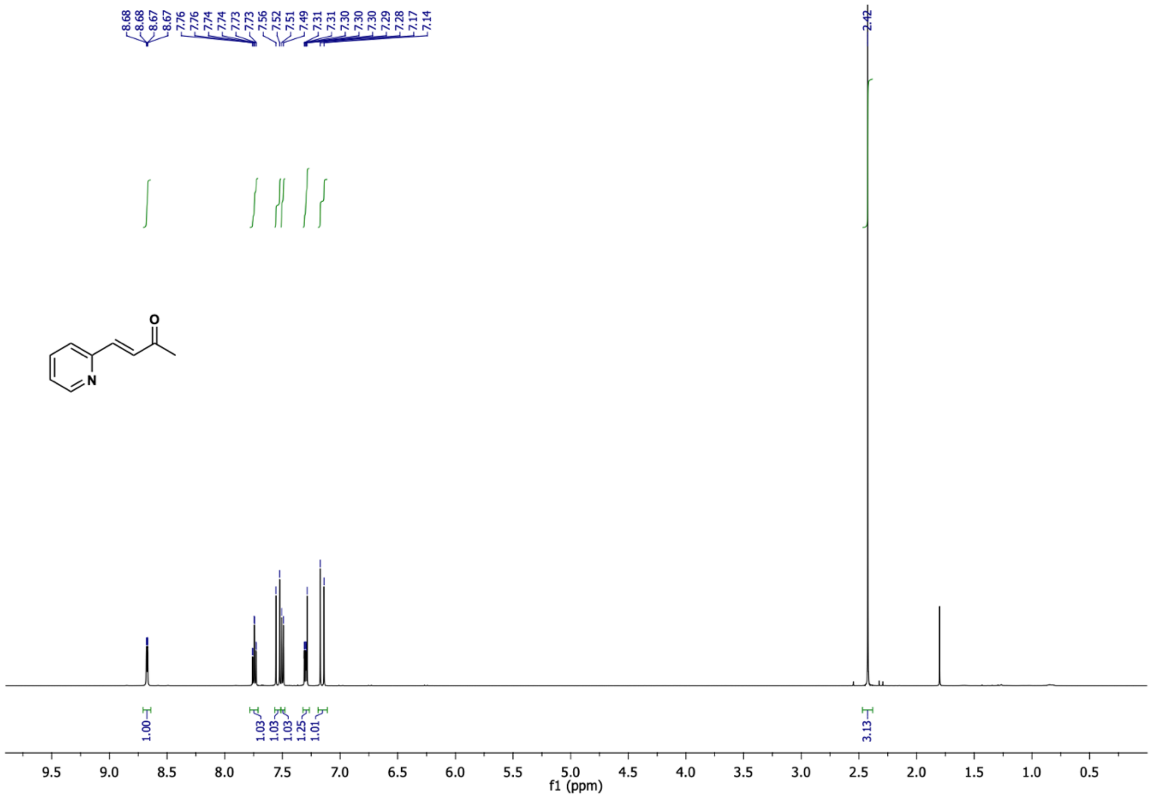

(*E*)-4-(pyridin-2-yl)but-3-en-2-one (**12b**) ^{13}C NMR (126 MHz, $CDCl_3$)

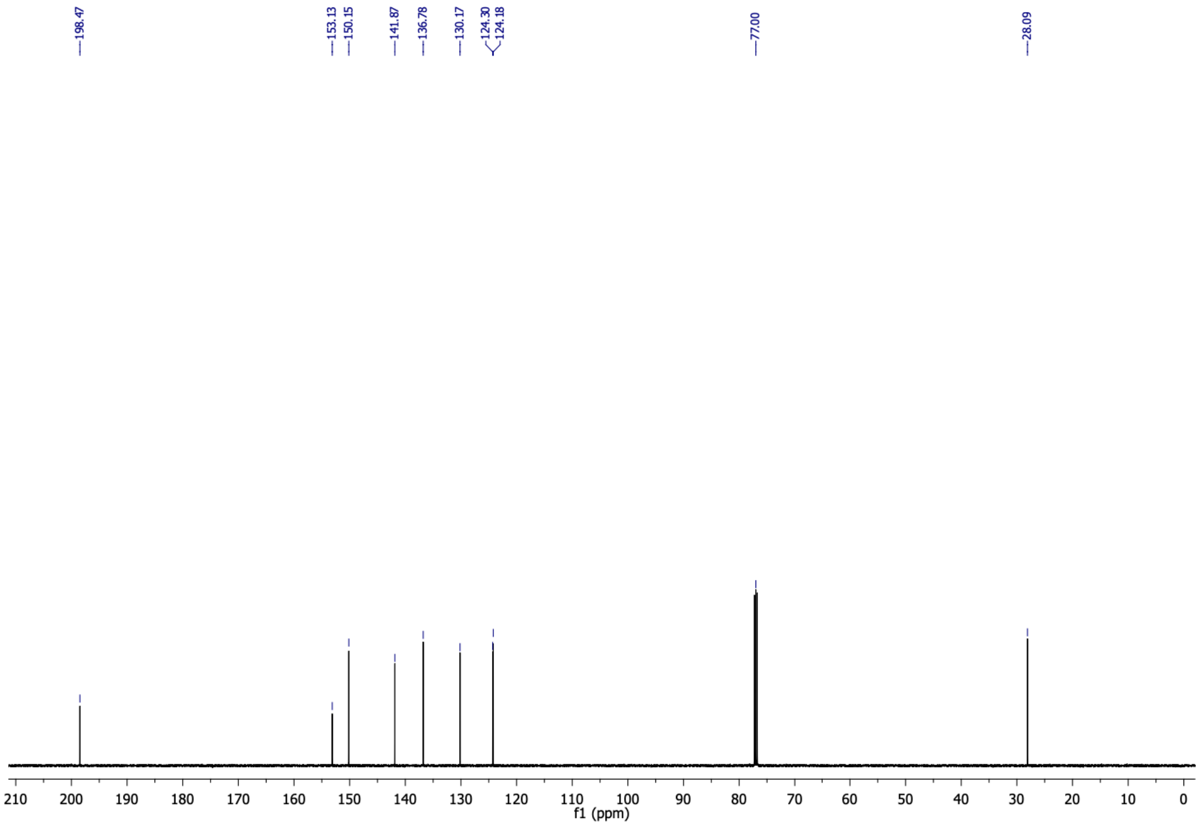

5-(pyridin-2-yl)cyclohexane-1,3-dione (**12c**) ^{1}H NMR (500 MHz, CD_3OD)

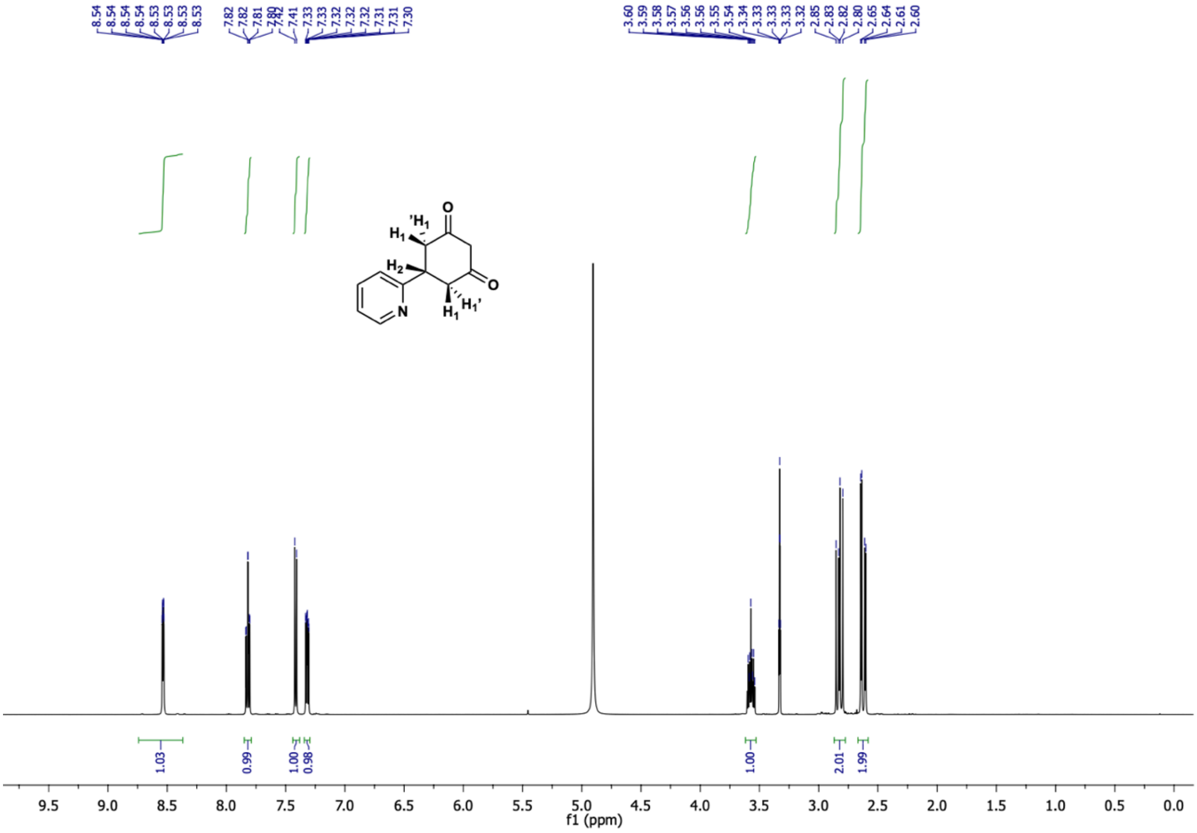

5-(pyridin-2-yl)cyclohexane-1,3-dione (**12c**) ^{13}C NMR (126 MHz, CD_3OD)

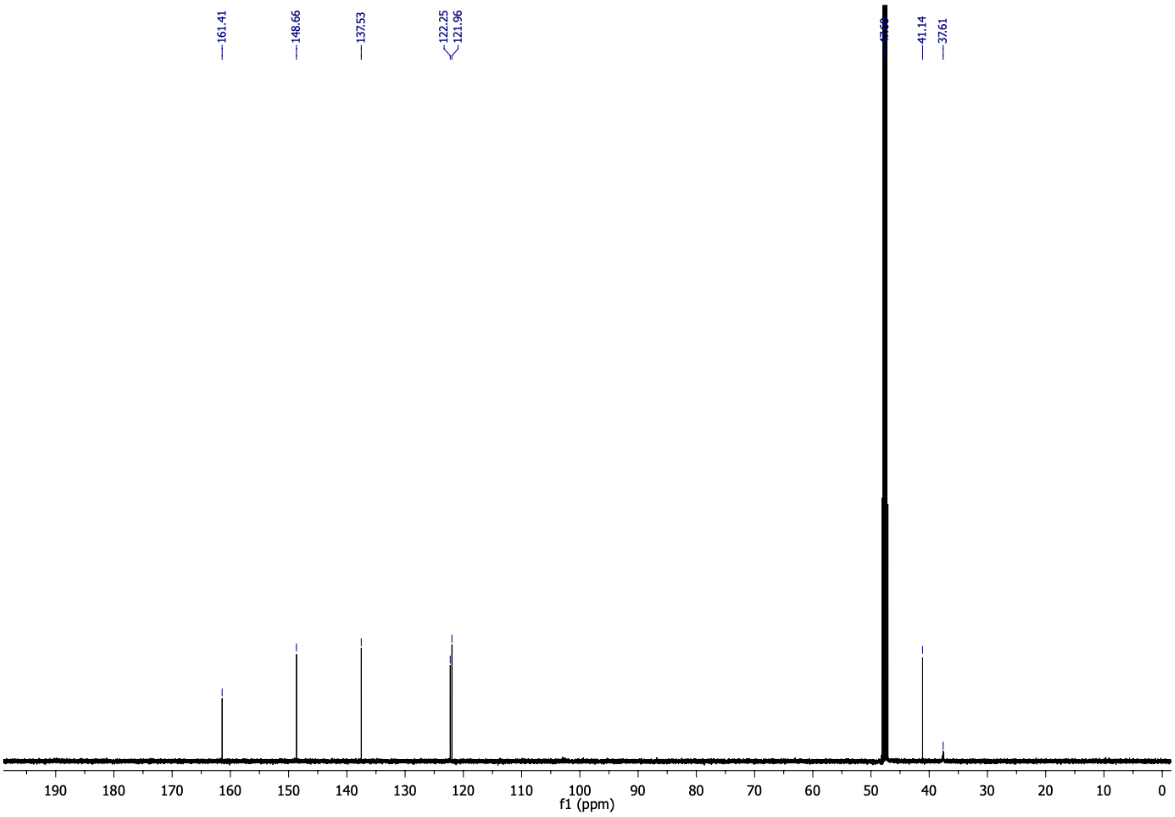

4-oxo-6-(pyridin-2-yl)-4,5,6,7-tetrahydrobenzofuran-3-carboxylic acid (**12**) ^{1}H NMR (500 MHz, $CDCl_3$)

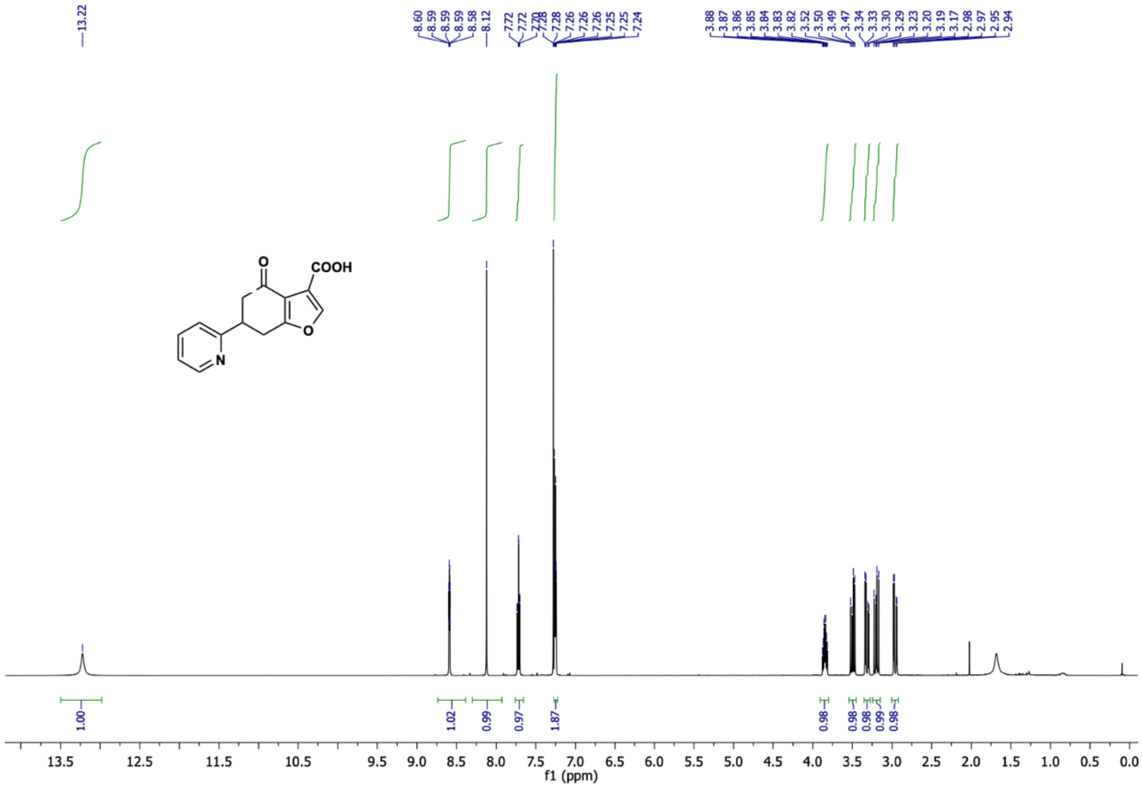

4-oxo-6-(pyridin-2-yl)-4,5,6,7-tetrahydrobenzofuran-3-carboxylic acid (**12**) ^{13}C NMR (126 MHz, $CDCl_3$)

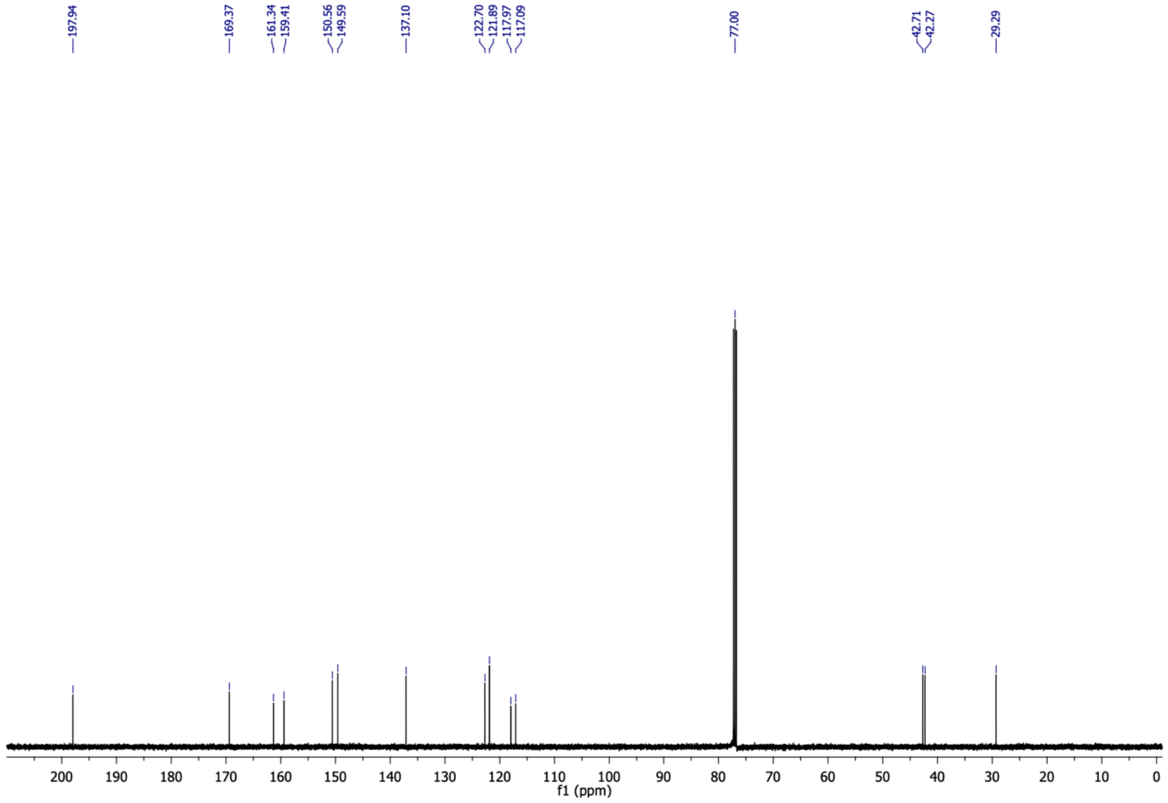

2-(3-carboxy-4-oxo-4,5,6,7-tetrahydrobenzofuran-6-yl)pyridine 1-oxide (**13**) ^{1}H NMR (500 MHz, $CDCl_3$)

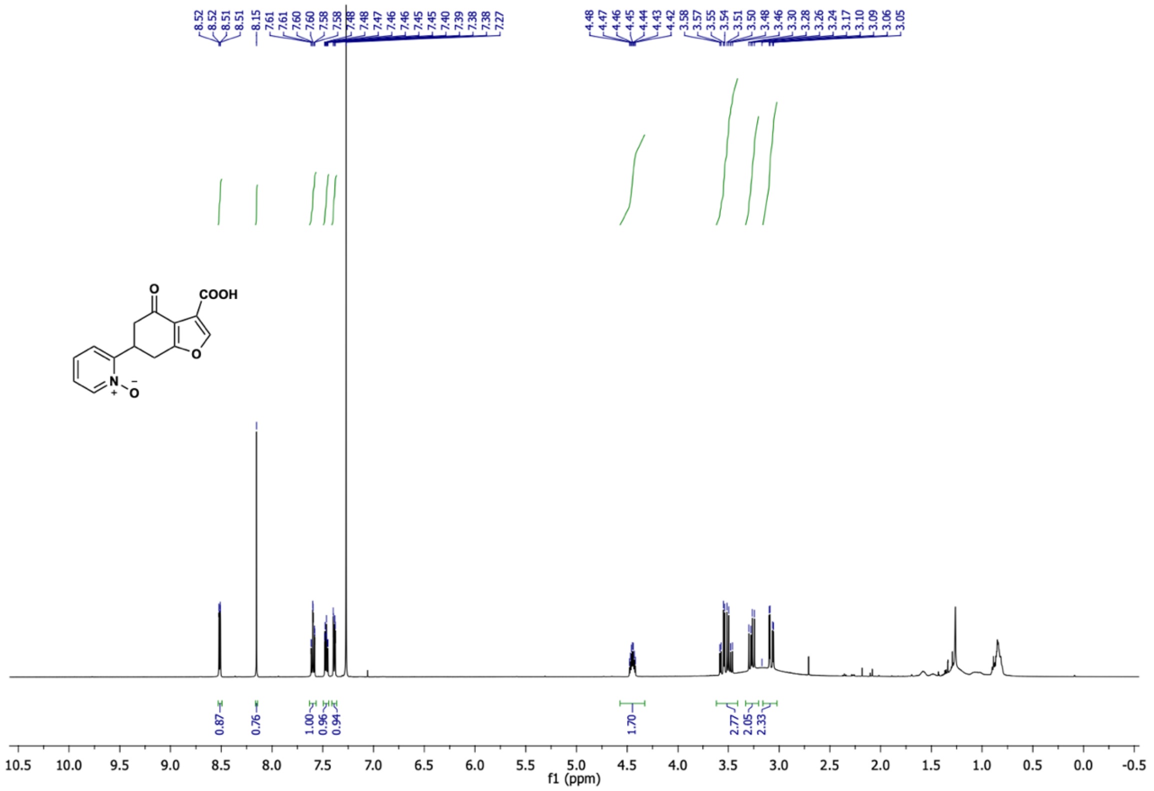

2-(3-carboxy-4-oxo-4,5,6,7-tetrahydrobenzofuran-6-yl)pyridine 1-oxide (**13**) ^{13}C NMR (126 MHz, $CDCl_3$)

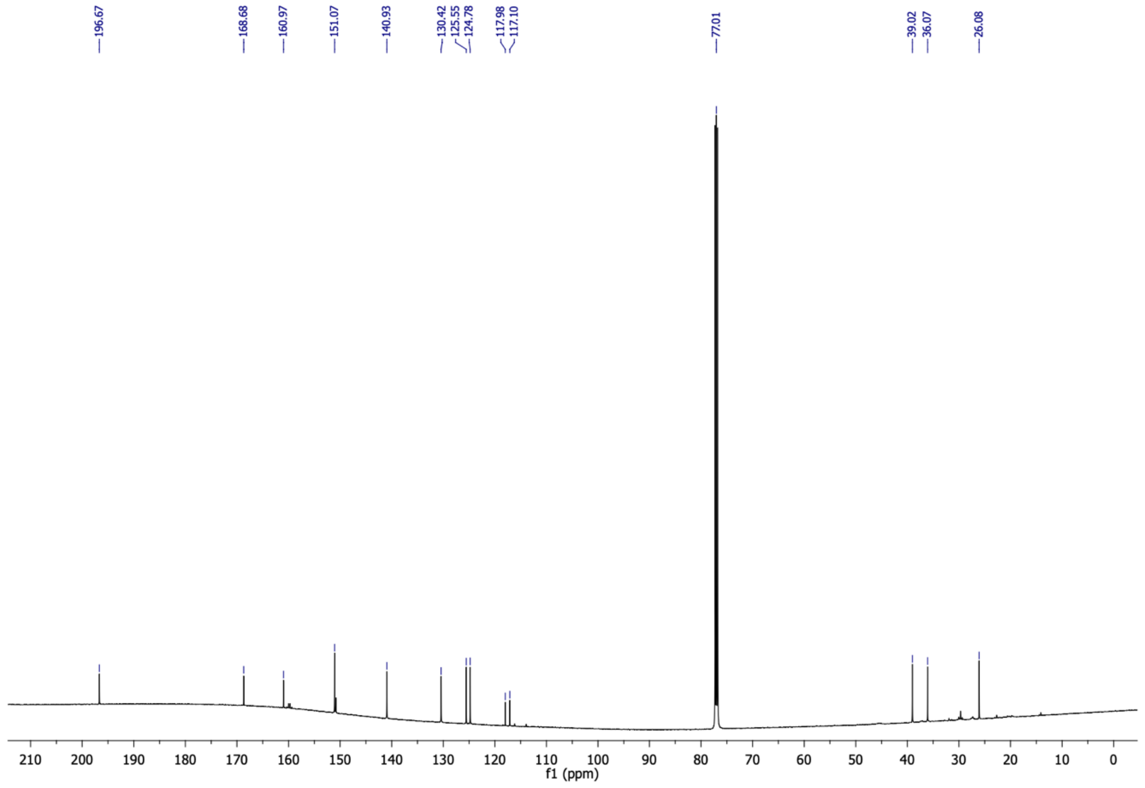

6-(2-hydroxyphenyl)-4-oxo-4,5,6,7-tetrahydrobenzofuran-3-carboxylic acid (**14**) ^{1}H NMR (500 MHz, $CDCl_3$)

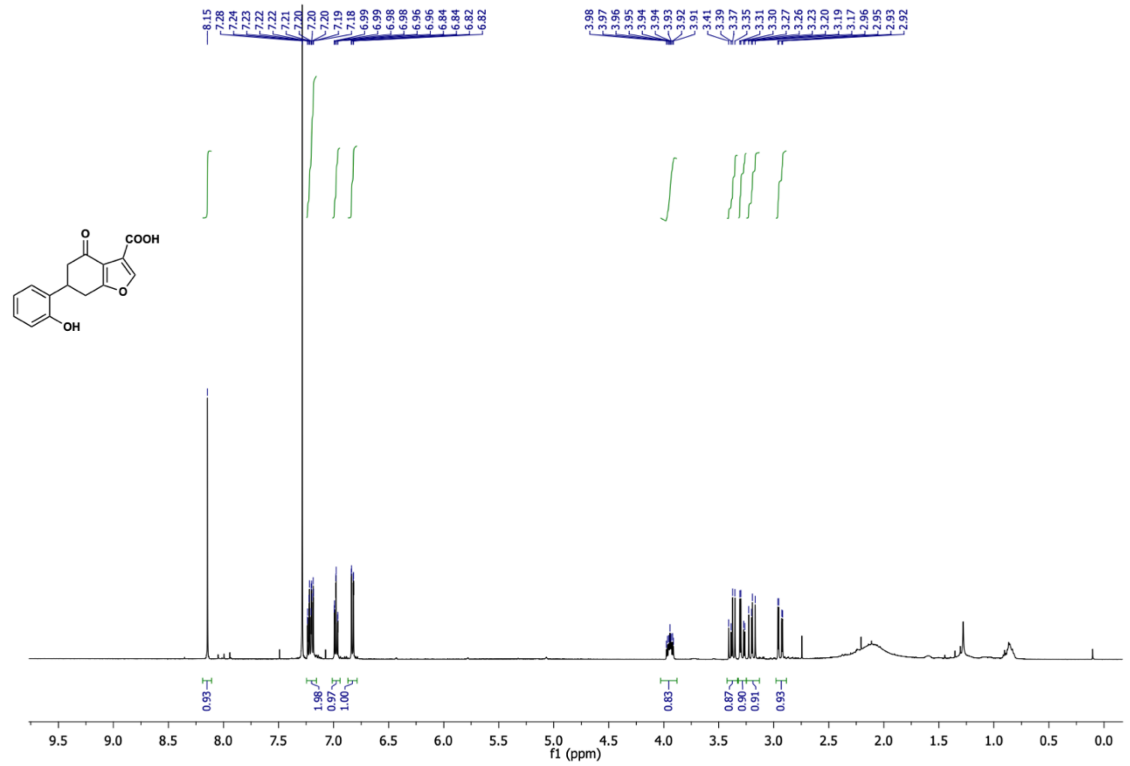

6-(2-hydroxyphenyl)-4-oxo-4,5,6,7-tetrahydrobenzofuran-3-carboxylic acid (**14**) ^{13}C NMR (126 MHz, $CDCl_3$)

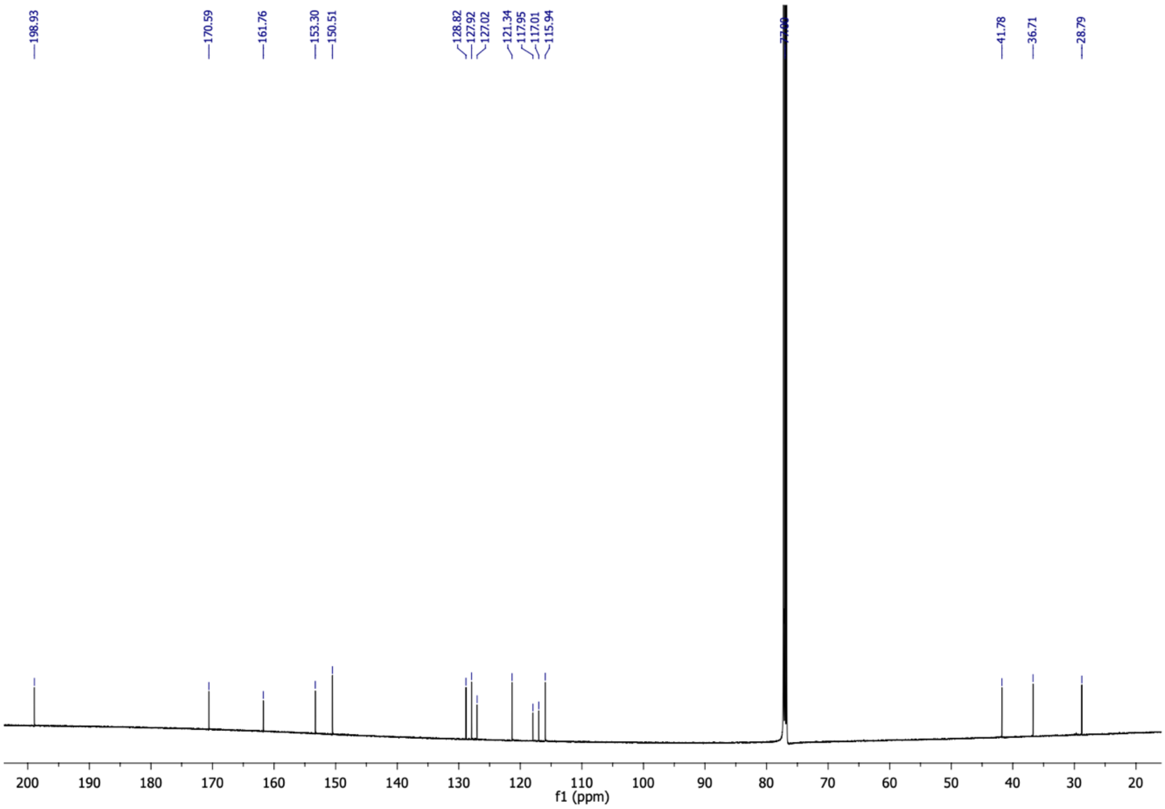

6-([1,1'-biphenyl]-2-yl)-4-oxo-4,5,6,7-tetrahydrobenzofuran-3-carboxylic acid (**15**) ^{1}H NMR (500 MHz, $CDCl_3$)

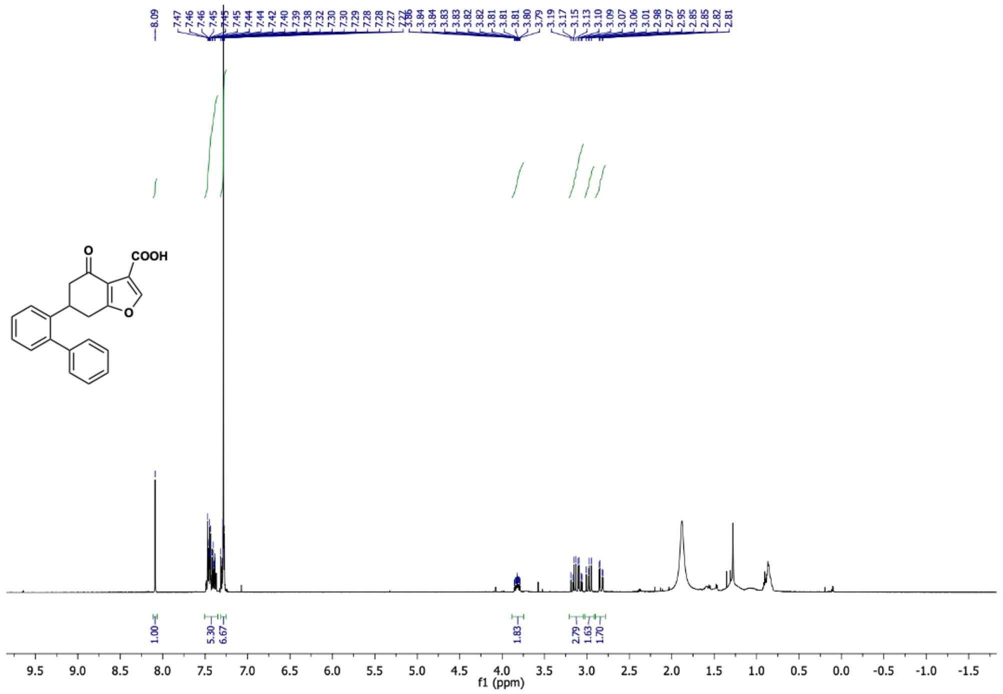

6-([1,1'-biphenyl]-2-yl)-4-oxo-4,5,6,7-tetrahydrobenzofuran-3-carboxylic acid (**15**) ^{13}C NMR (126 MHz, $CDCl_3$)

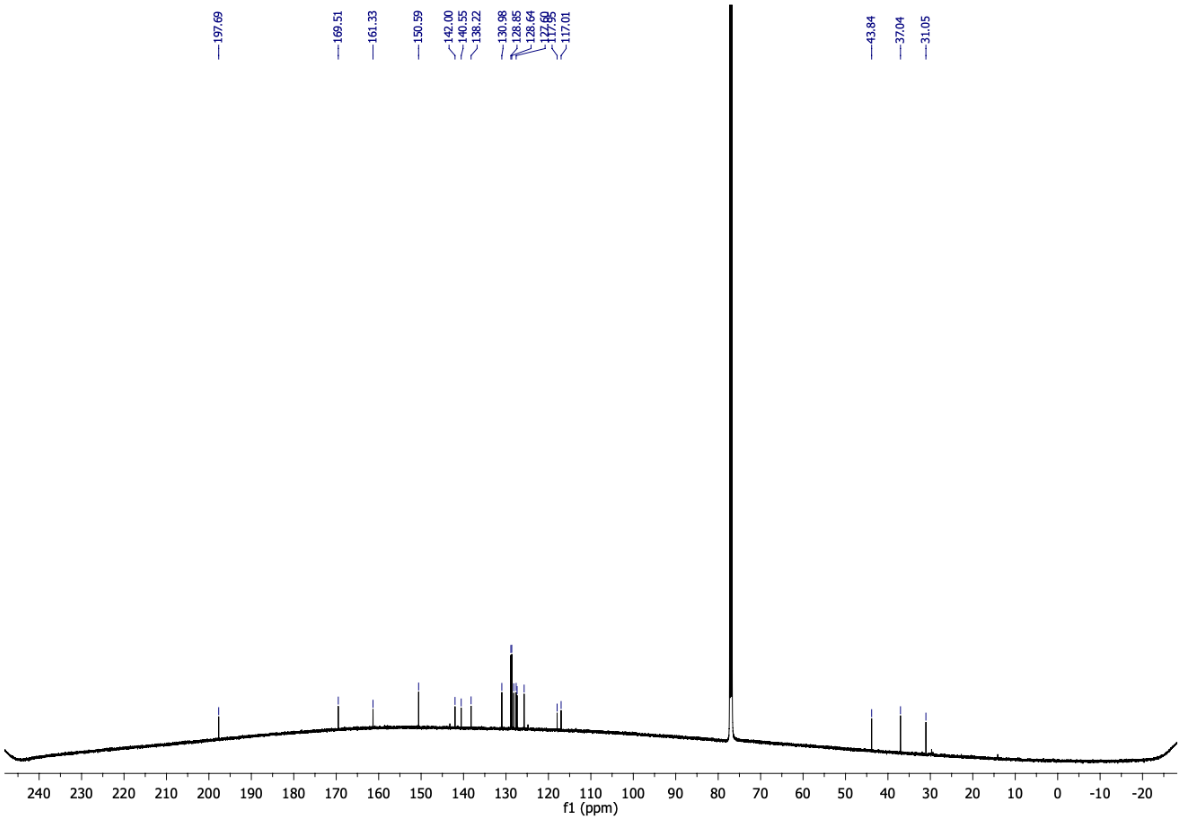

2-bromo-6-methoxybenzaldehyde (**16a**) ^{1}H NMR (500 MHz, $CDCl_3$)

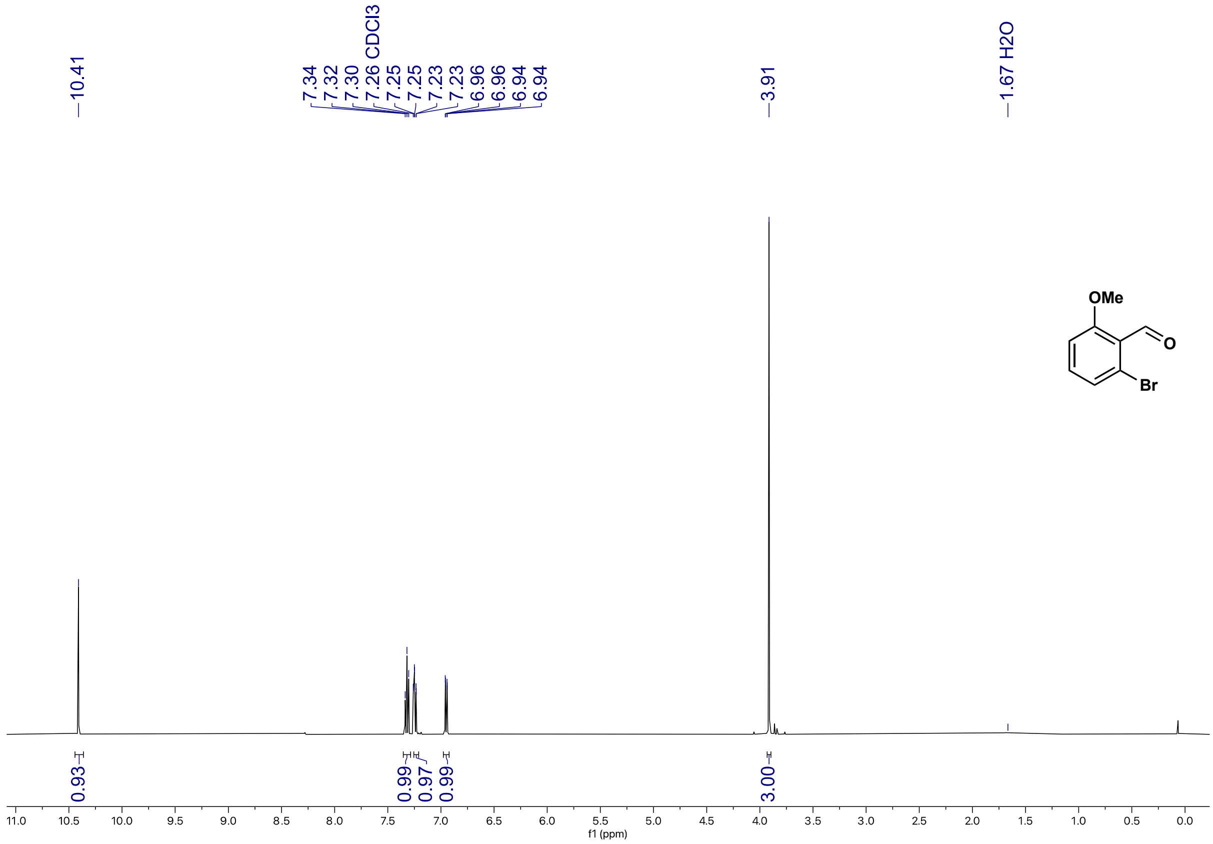

2-bromo-6-methoxybenzaldehyde (**16a**) ^{13}C NMR (126 MHz, $CDCl_3$)

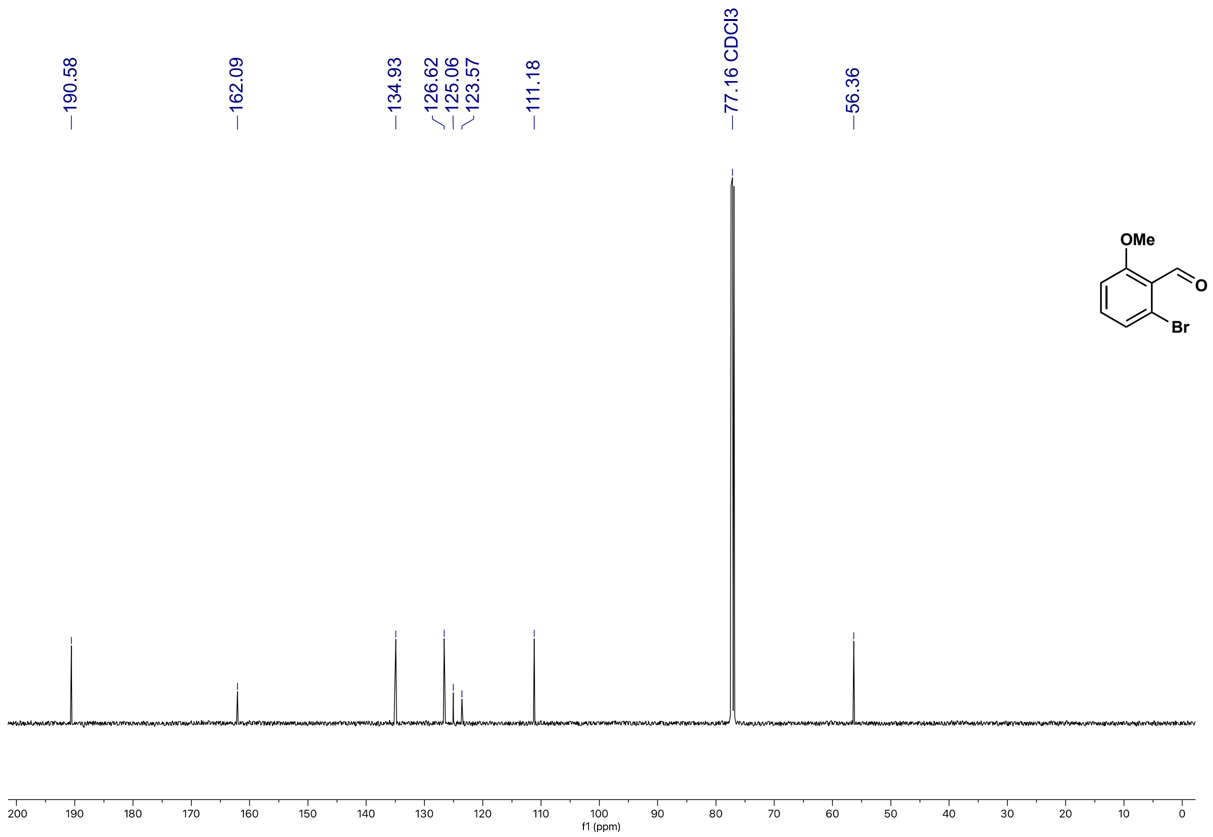

(E)-4-(2-bromo-6-methoxyphenyl)but-3-en-2-one (**16b**) ^{1}H NMR (500 MHz, $CDCl_3$)

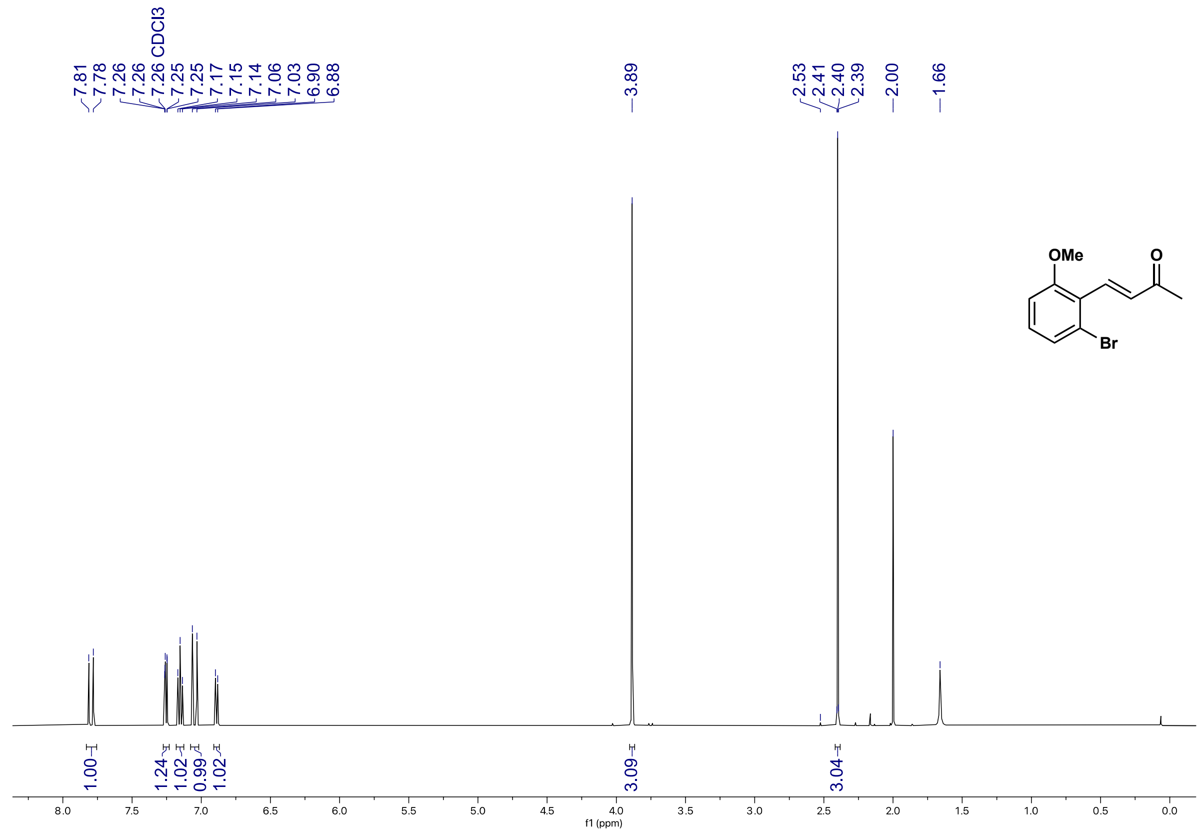

(E)-4-(2-bromo-6-methoxyphenyl)but-3-en-2-one (**16b**) ^{13}C NMR (126 MHz, $CDCl_3$)

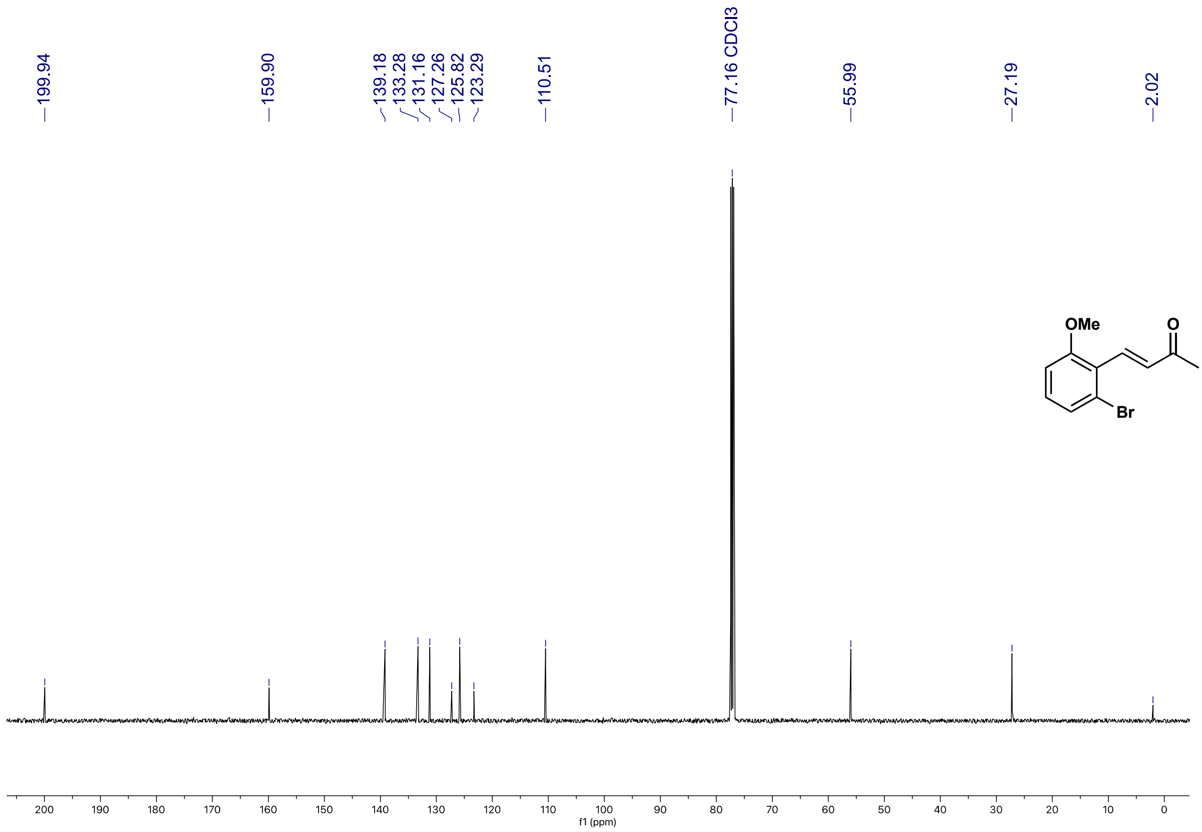

5-(2-bromo-6-methoxyphenyl)cyclohexane-1,3-dione (**16c**) ^{1}H NMR (500 MHz, d_6-DMSO)

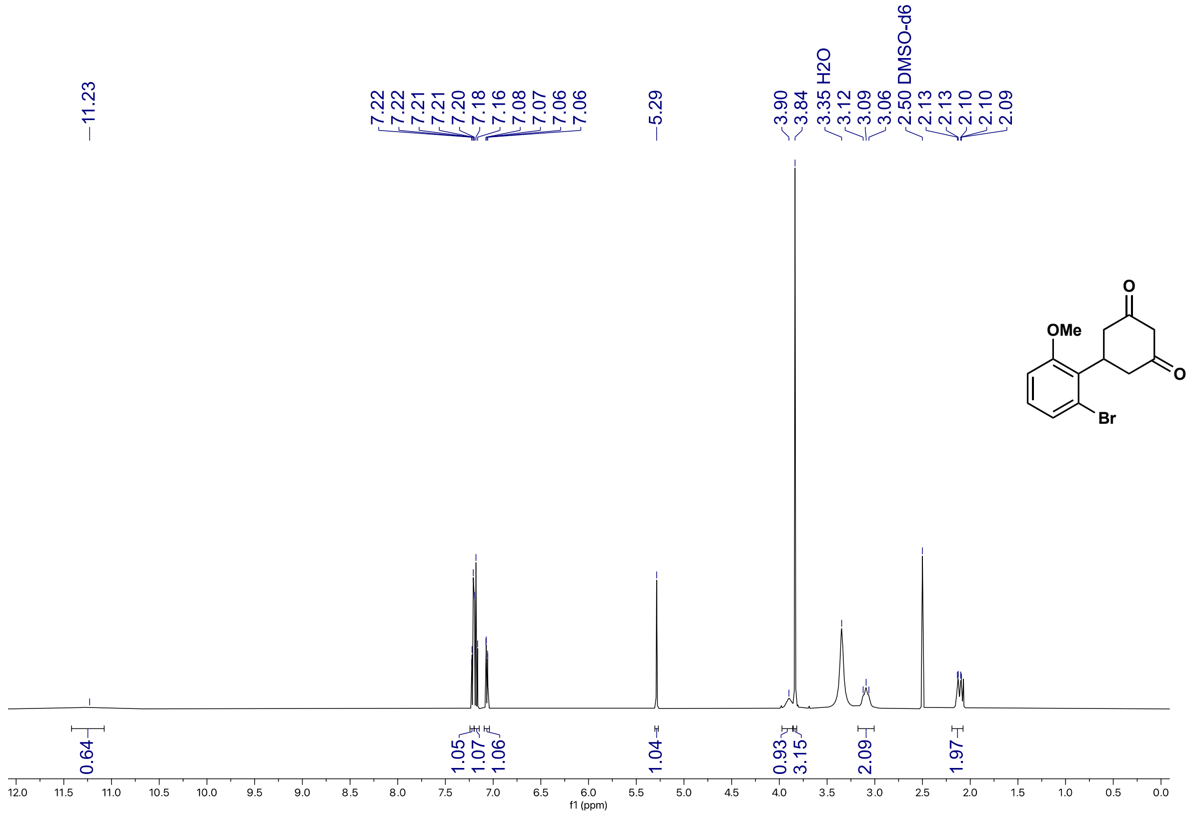

5-(2-bromo-6-methoxyphenyl)cyclohexane-1,3-dione (**16c**) ^{13}C NMR (126 MHz, d_6-DMSO)

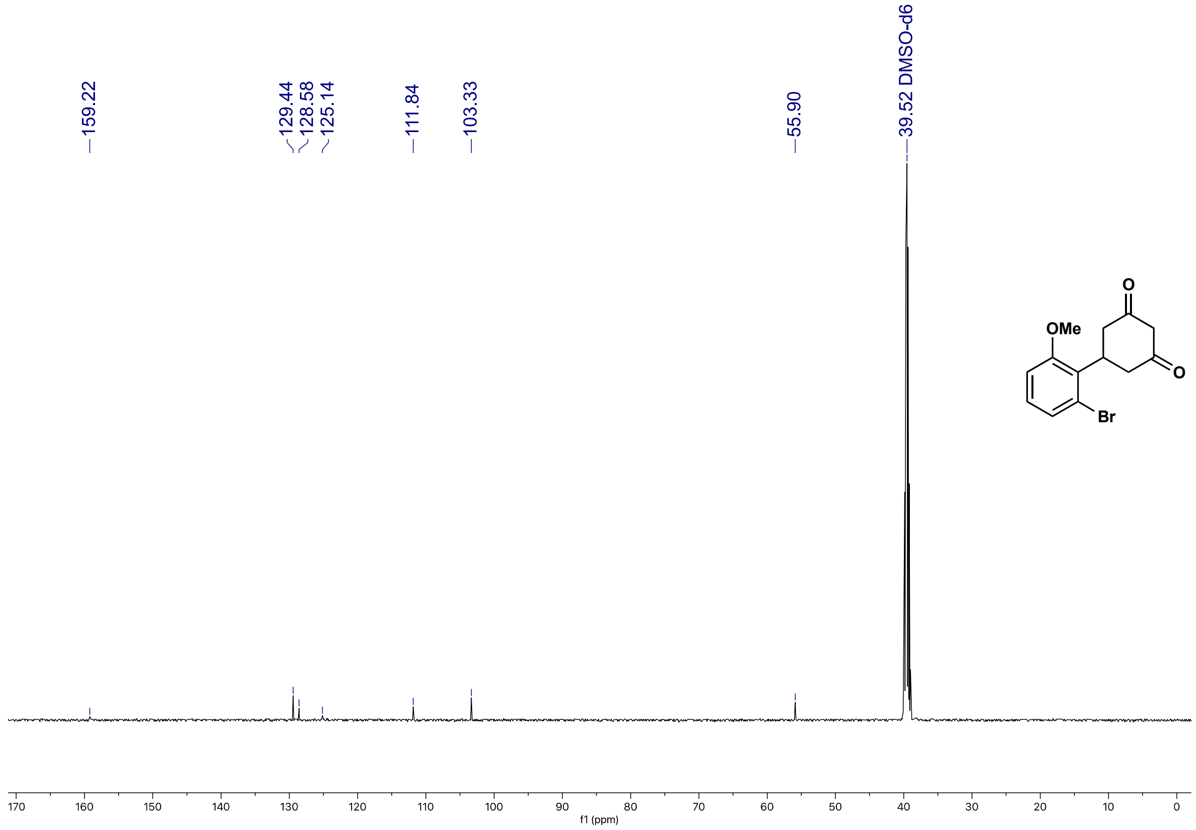

6-(2-bromo-6-methoxyphenyl)-4-oxo-4,5,6,7-tetrahydrobenzofuran-3-carboxylic acid (**16**) ^{1}H NMR (500 MHz, $CDCl_3$)

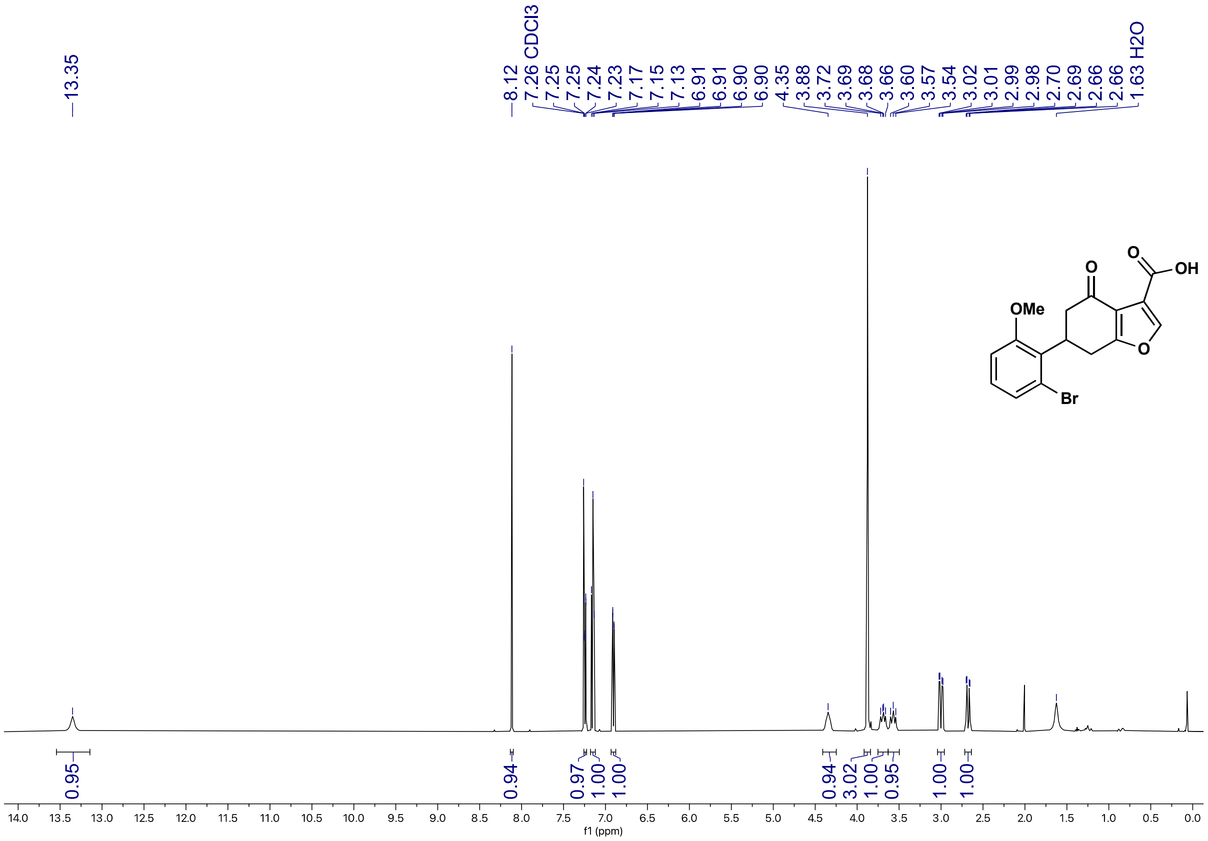

6-(2-bromo-6-methoxyphenyl)-4-oxo-4,5,6,7-tetrahydrobenzofuran-3-carboxylic acid (**16**) ^{13}C NMR (126 MHz, $CDCl_3$)

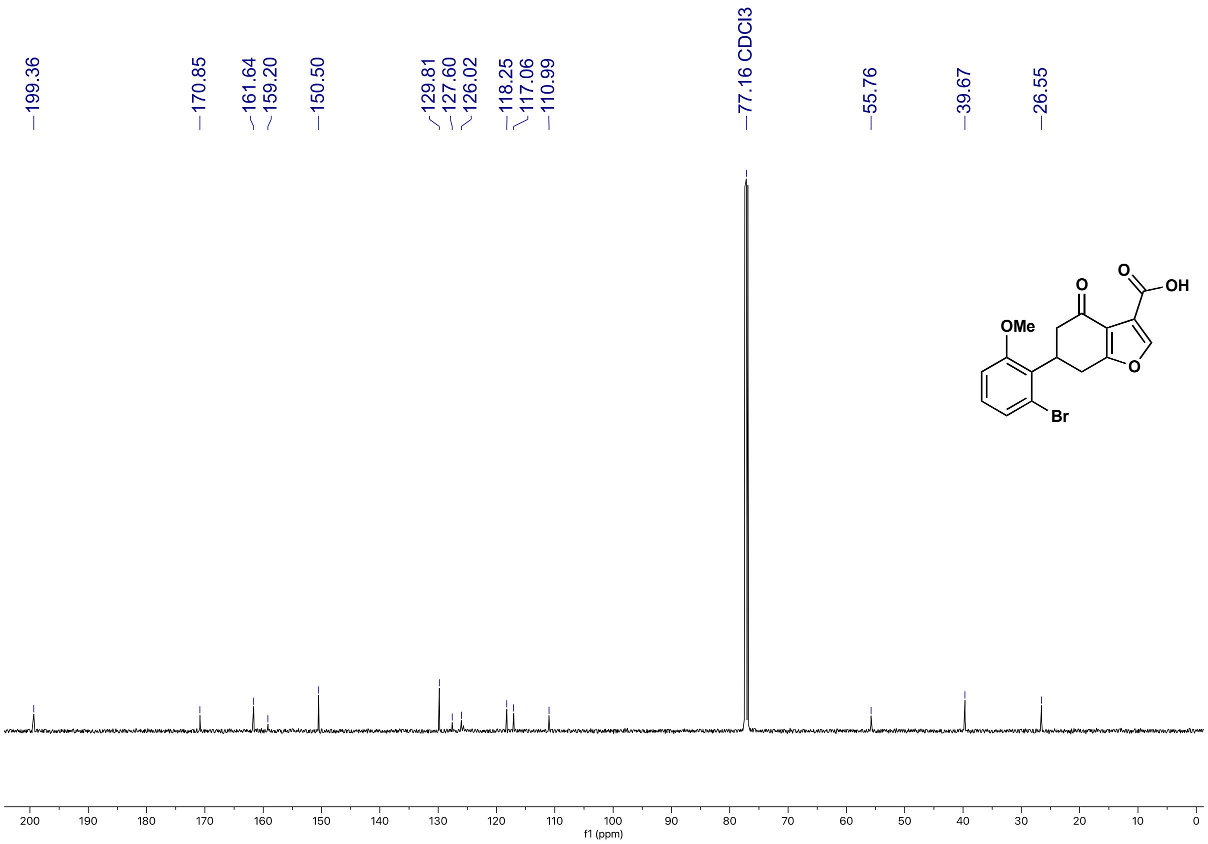

ethyl 6-(2-bromo-6-methoxyphenyl)-4-oxo-4,5,6,7-tetrahydrobenzofuran-3-carboxylate (**16d**)
^{1}H NMR (500 MHz, $CDCl_3$)

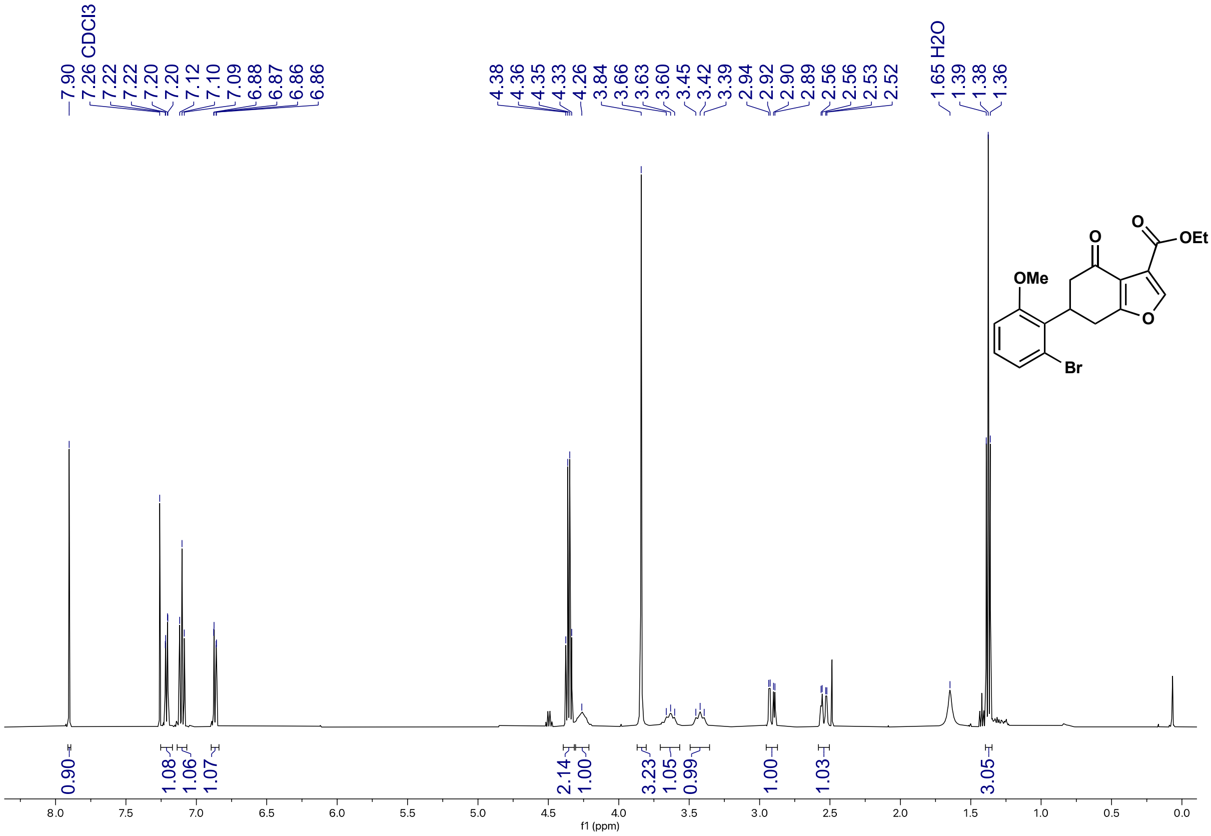

ethyl 6-(2-bromo-6-methoxyphenyl)-4-oxo-4,5,6,7-tetrahydrobenzofuran-3-carboxylate (**16d**)
^{13}C NMR (126 MHz, $CDCl_3$)

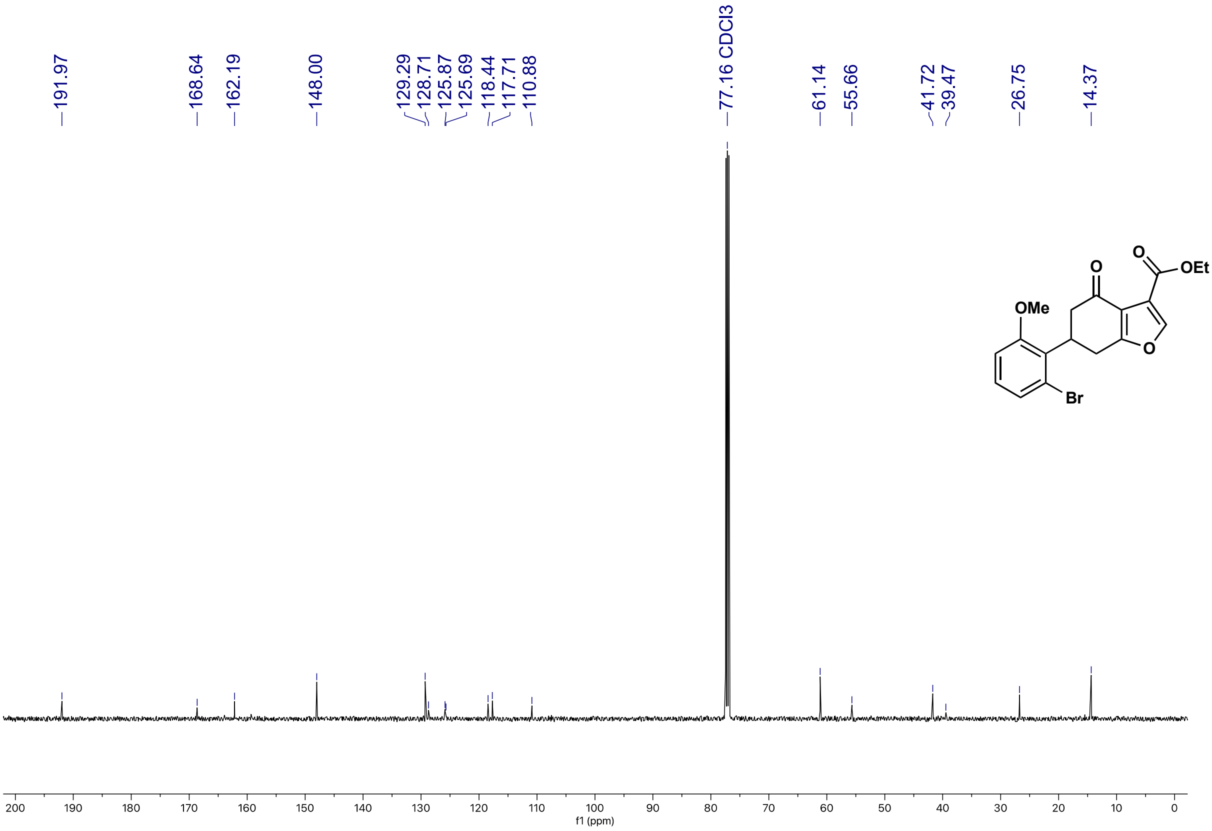

(*E*)-4-(2-chloro-6-fluorophenyl)but-3-en-2-one (**17b**) ^{1}H NMR (500 MHz, $CDCl_3$)

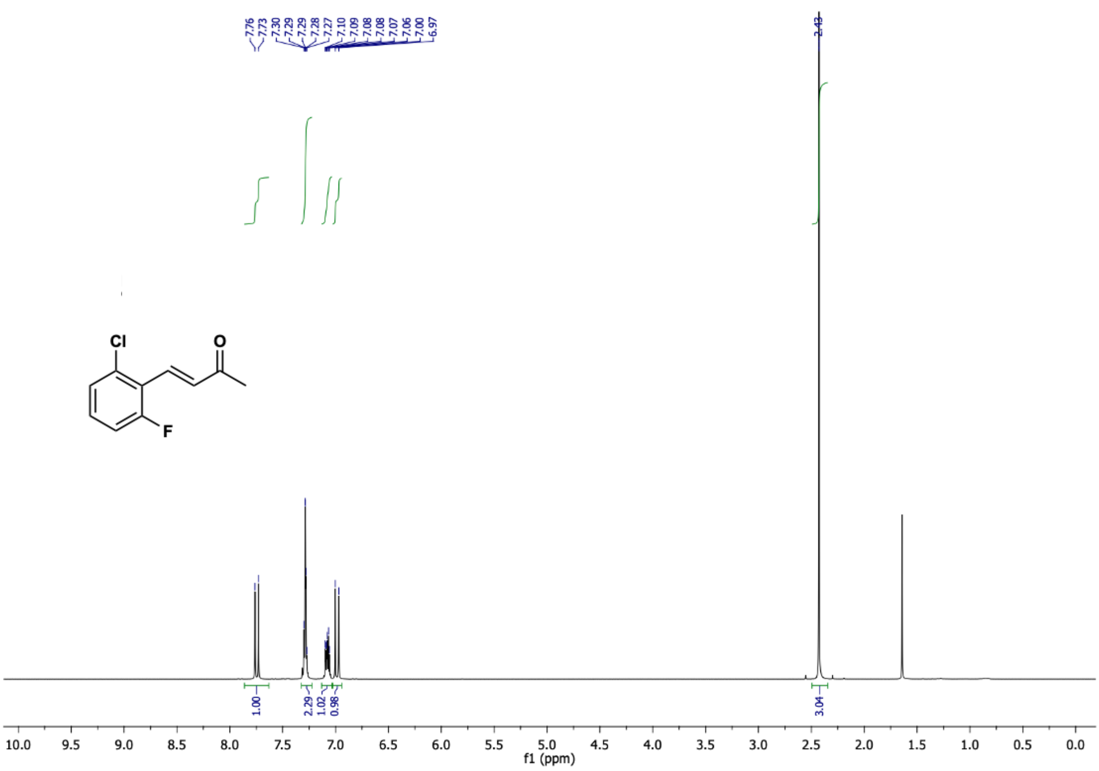

(*E*)-4-(2-chloro-6-fluorophenyl)but-3-en-2-one (**17b**) ^{13}C NMR (126 MHz, $CDCl_3$)

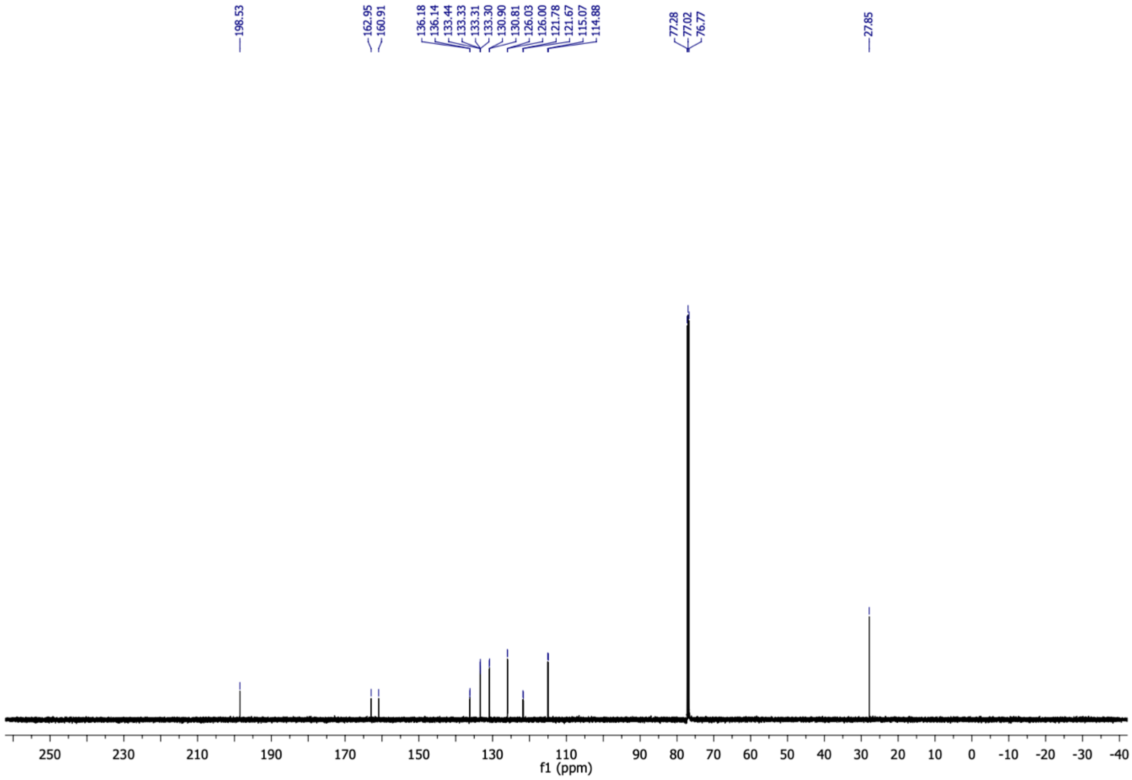

(*E*)-4-(2-chloro-6-fluorophenyl)but-3-en-2-one (**17b**) ^{19}F NMR (470 MHz, $CDCl_3$)

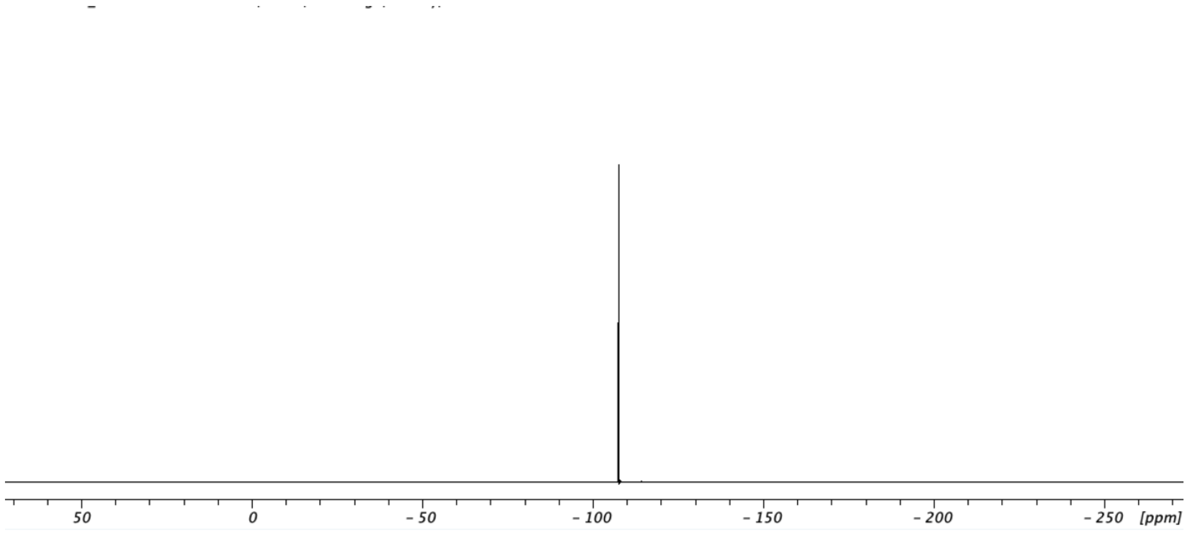

5-(2-chloro-6-fluorophenyl)cyclohexane-1,3-dione (**17c**) ^{1}H NMR (500 MHz, CD_3OD)

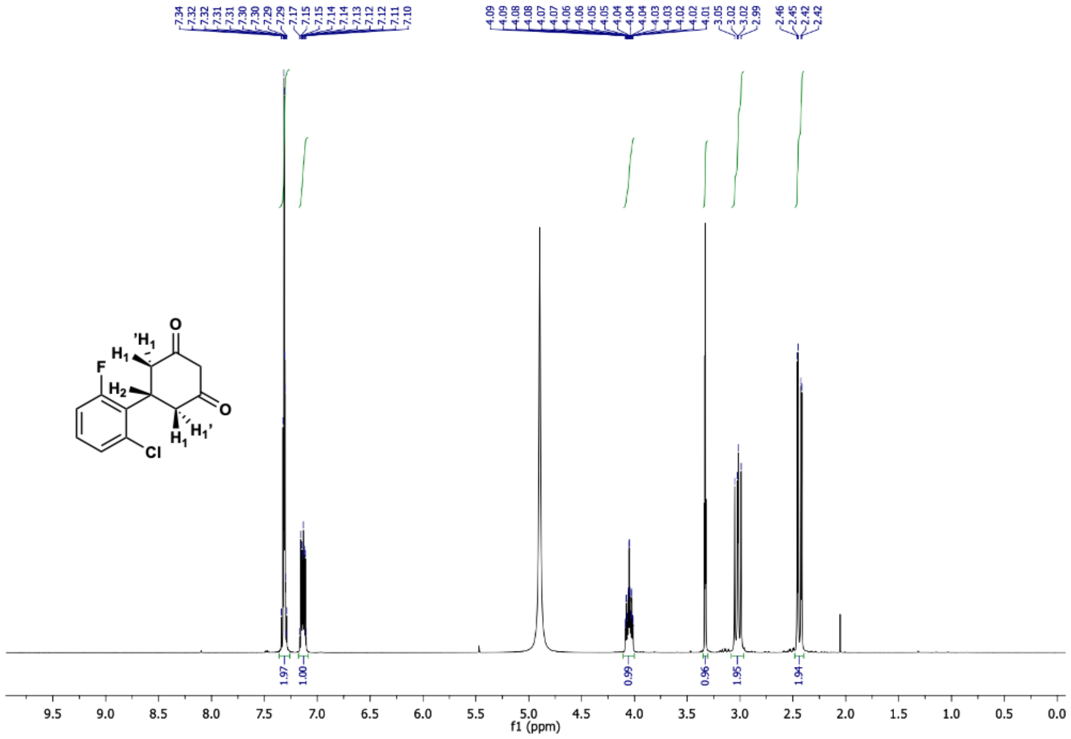

5-(2-chloro-6-fluorophenyl)cyclohexane-1,3-dione (**17c**) ^{13}C NMR (126 MHz, CD_3OD)

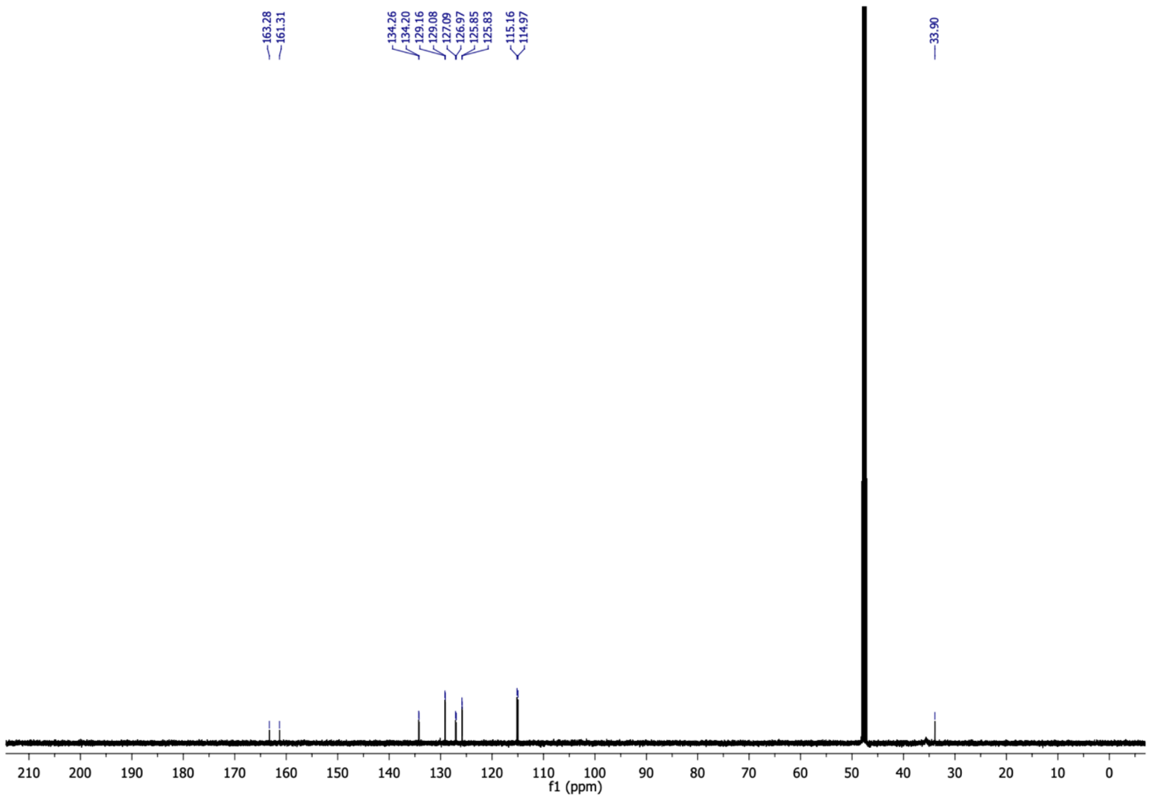

5-(2-chloro-6-fluorophenyl)cyclohexane-1,3-dione (**17c**) ^{19}F NMR (470 MHz, CD_3OD)

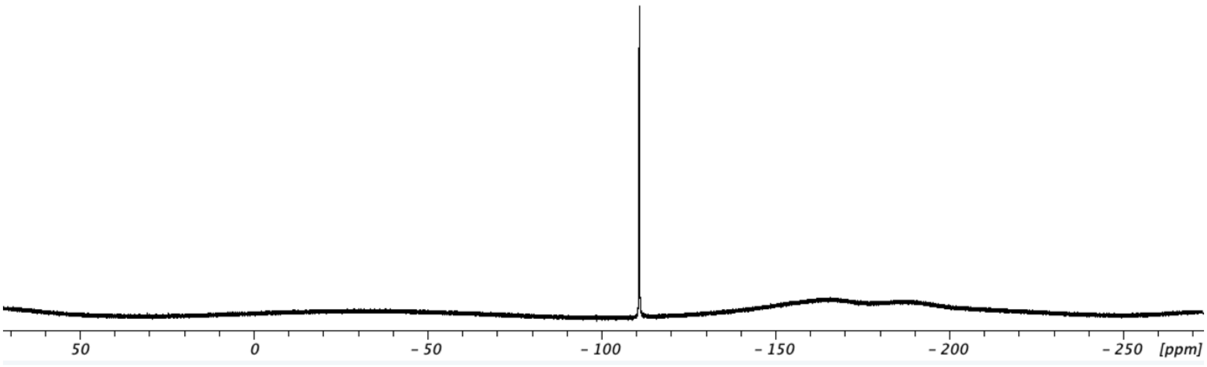

6-(2-chloro-6-fluorophenyl)-4-oxo-4,5,6,7-tetrahydrobenzofuran-3-carboxylic acid (**17**) ^{1}H NMR (400 MHz, $CDCl_3$)

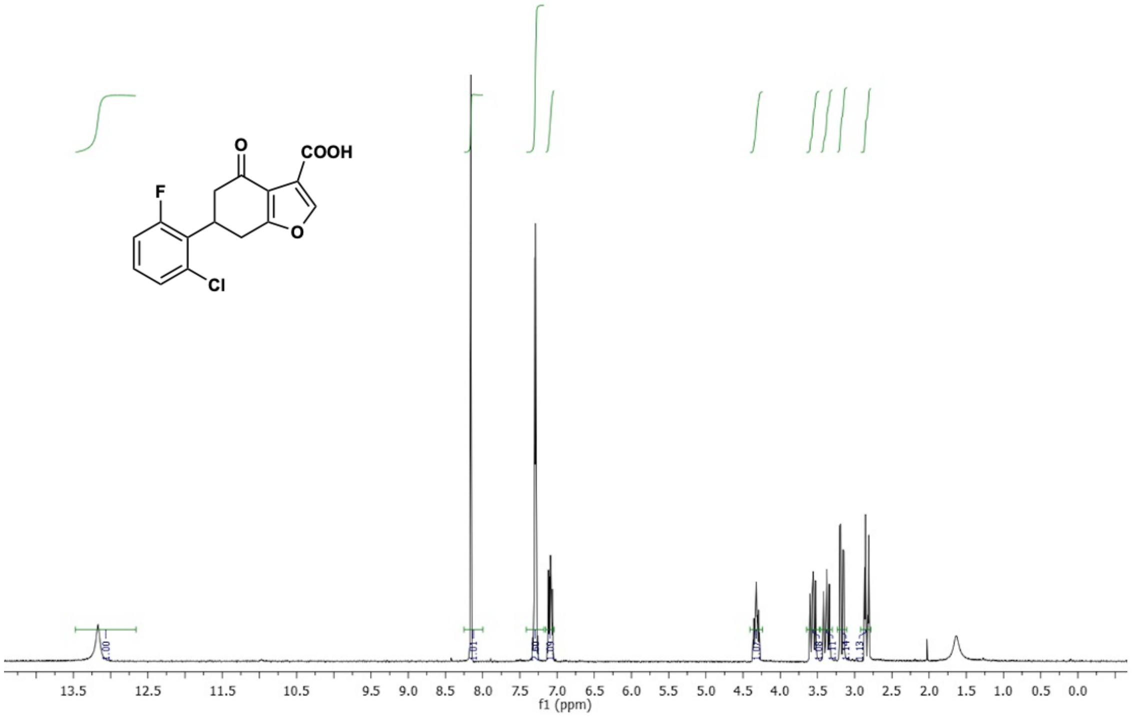

6-(2-chloro-6-fluorophenyl)-4-oxo-4,5,6,7-tetrahydrobenzofuran-3-carboxylic acid (**17**) ^{13}C NMR (101 MHz, $CDCl_3$)

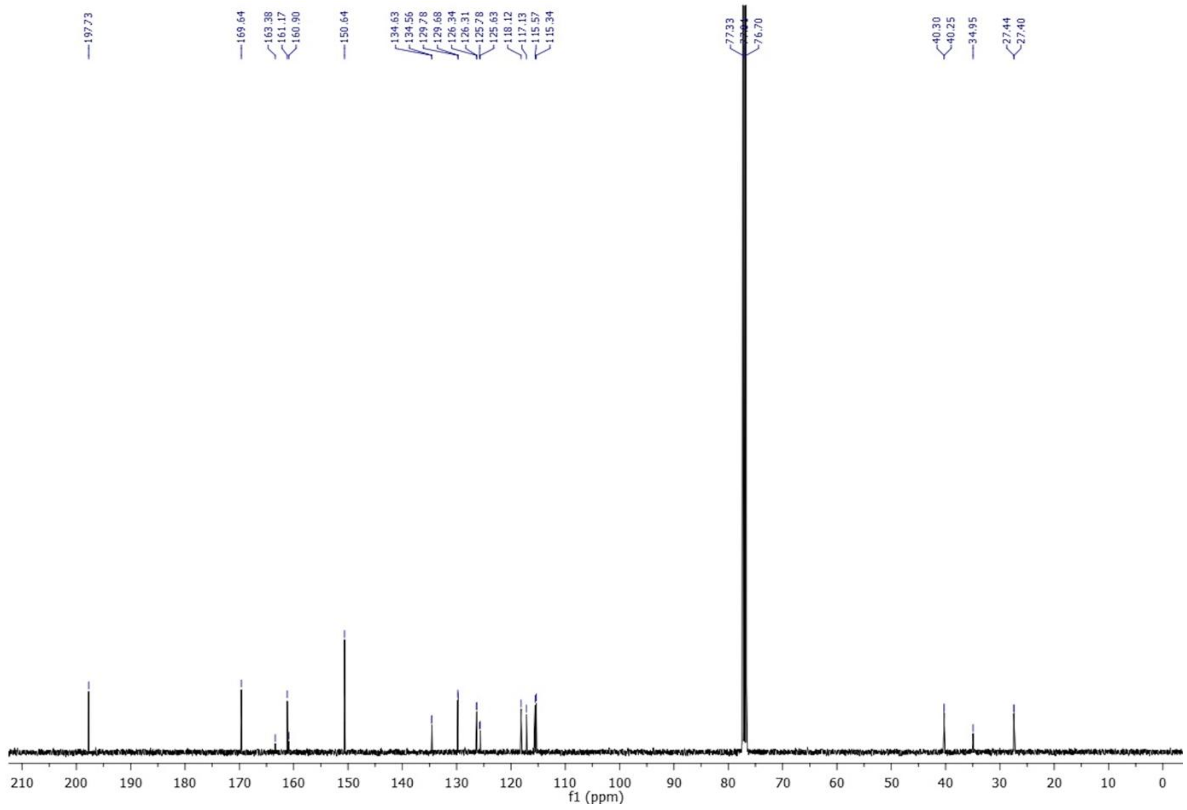

6-(2-chloro-6-fluorophenyl)-4-oxo-4,5,6,7-tetrahydrobenzofuran-3-carboxylic acid (**17**) ^{19}F NMR (376 MHz, $CDCl_3$)

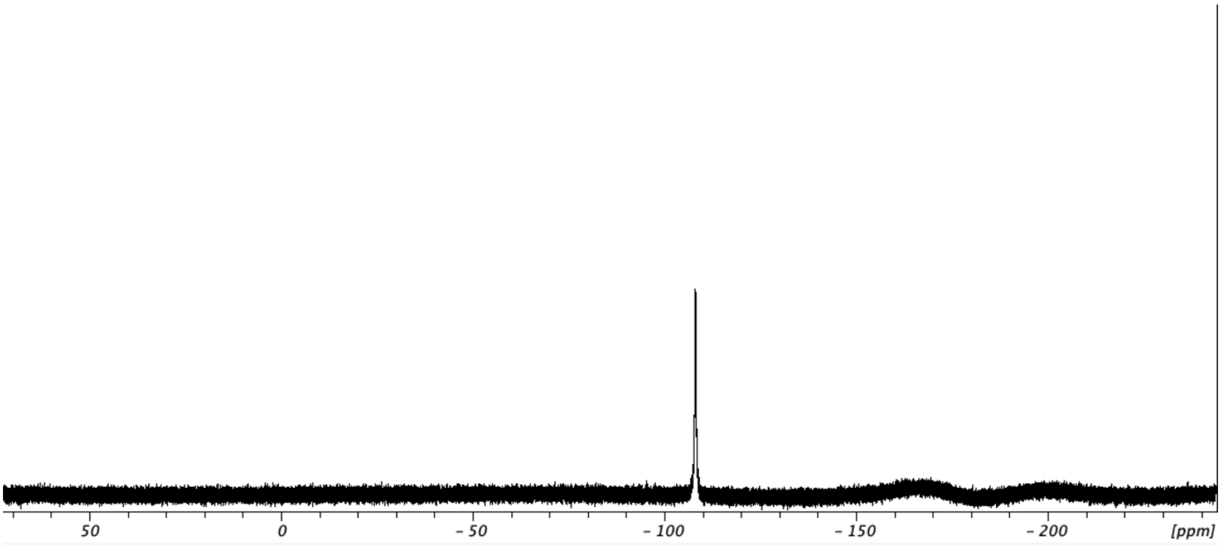

6-(2-chloro-6-fluorophenyl)-4-hydroxy-4,5,6,7-tetrahydrobenzofuran-3-carboxylic acid (**17e**) ^{1}H NMR (500 MHz, $CDCl_3$)

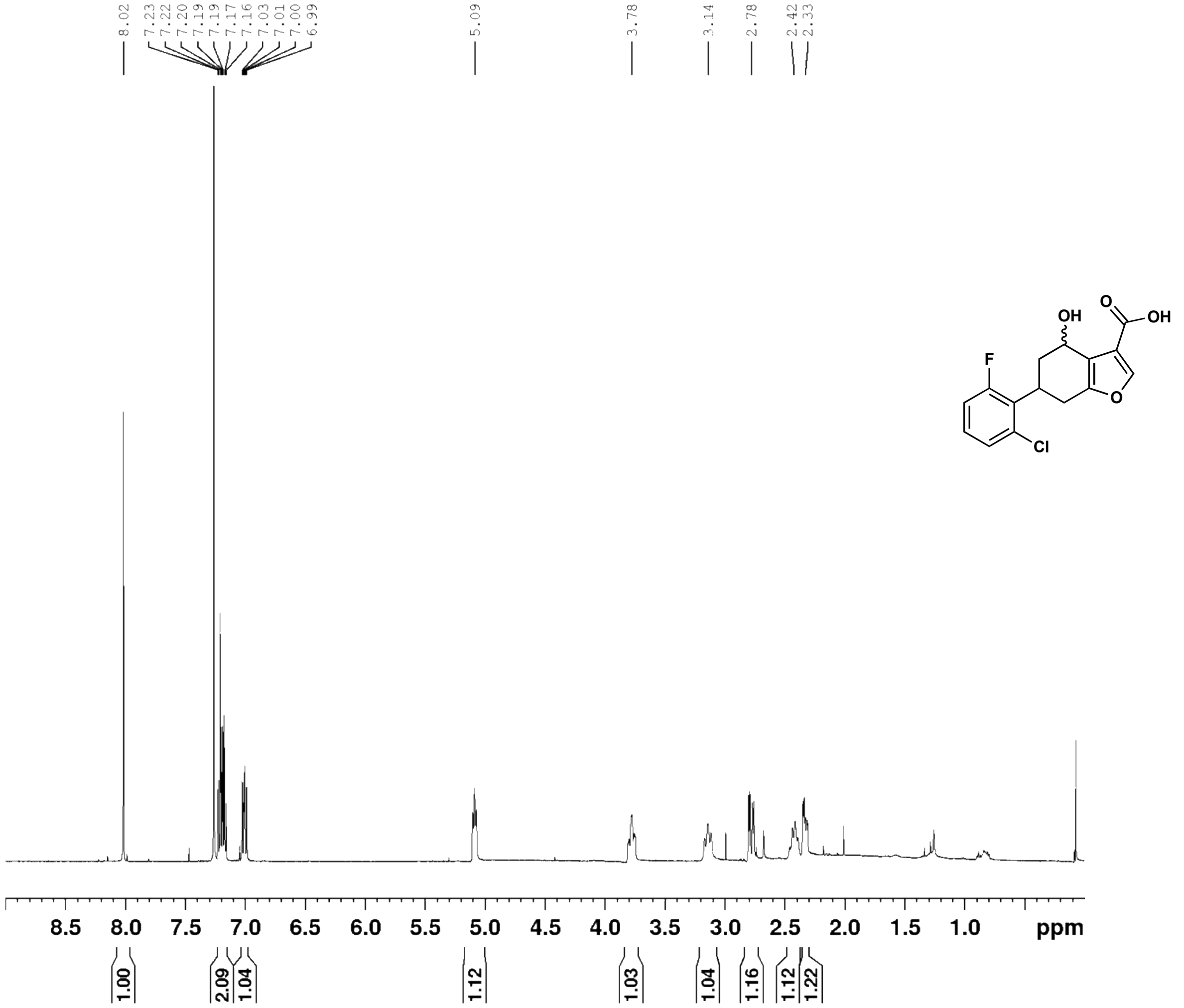

6-(2-chloro-6-fluorophenyl)-4-hydroxy-4,5,6,7-tetrahydrobenzofuran-3-carboxylic acid (**17e**)
^{13}C NMR (126 MHz, $CDCl_3$)

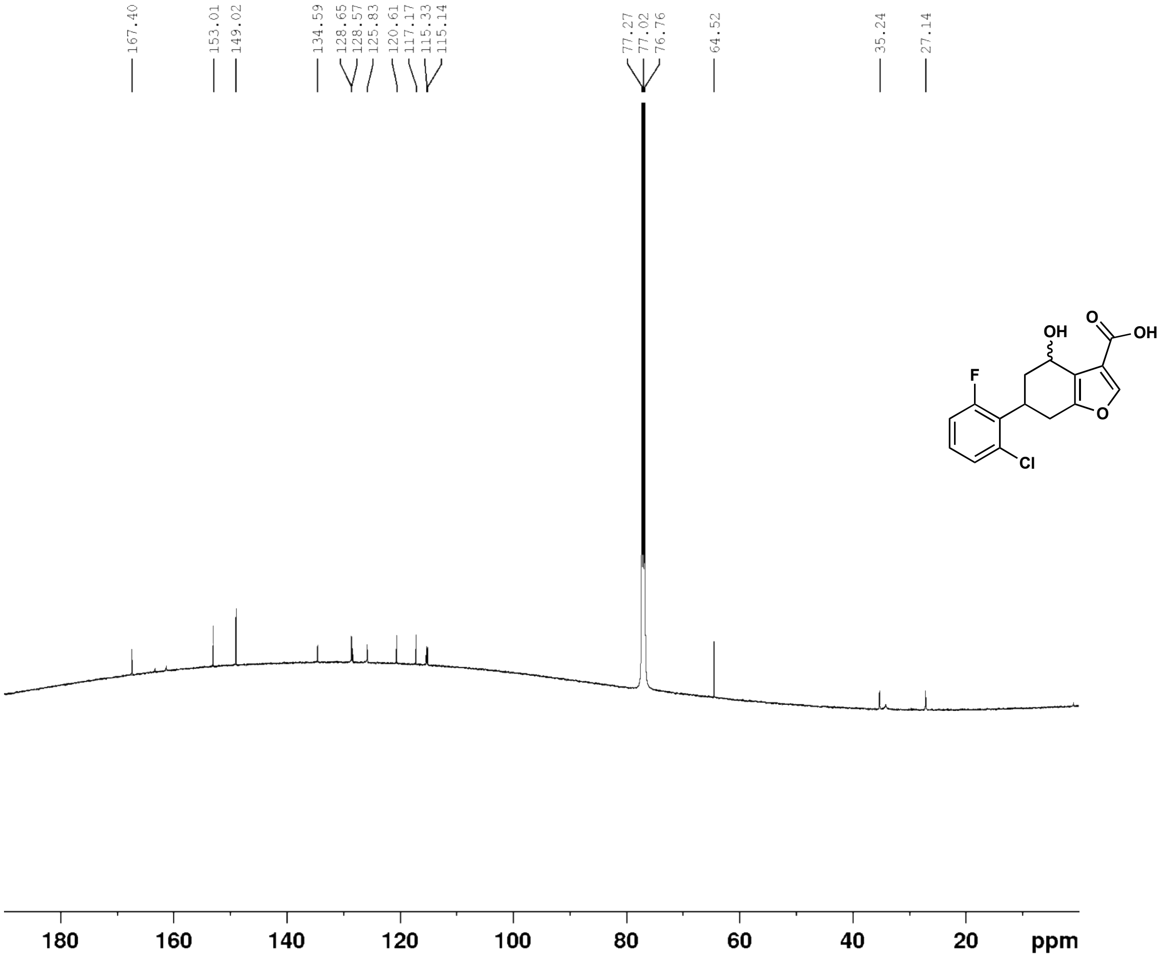

(*E*)-4-(2,6-dimethoxyphenyl)but-3-en-2-one (**18b**) ^{1}H NMR (500 MHz, $CDCl_3$)

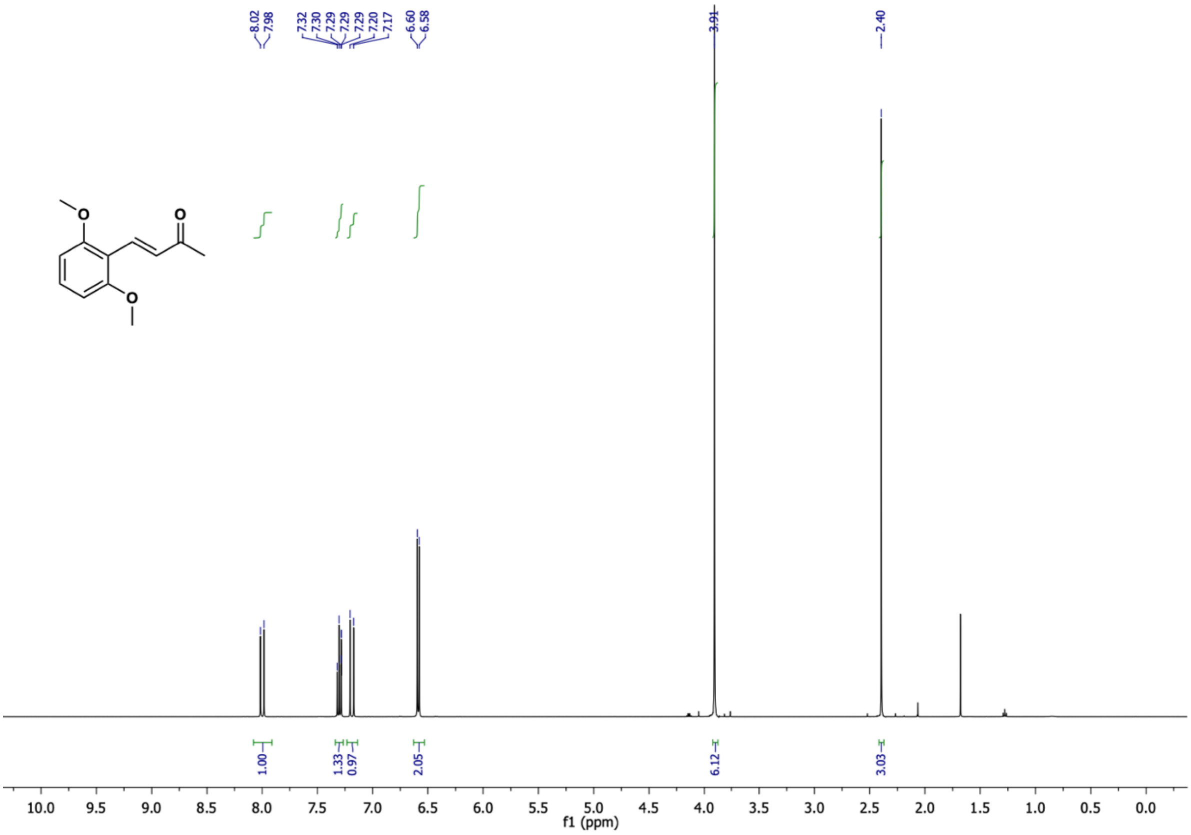

(*E*)-4-(2,6-dimethoxyphenyl)but-3-en-2-one (**18b**) ^{13}C NMR (126 MHz, $CDCl_3$)

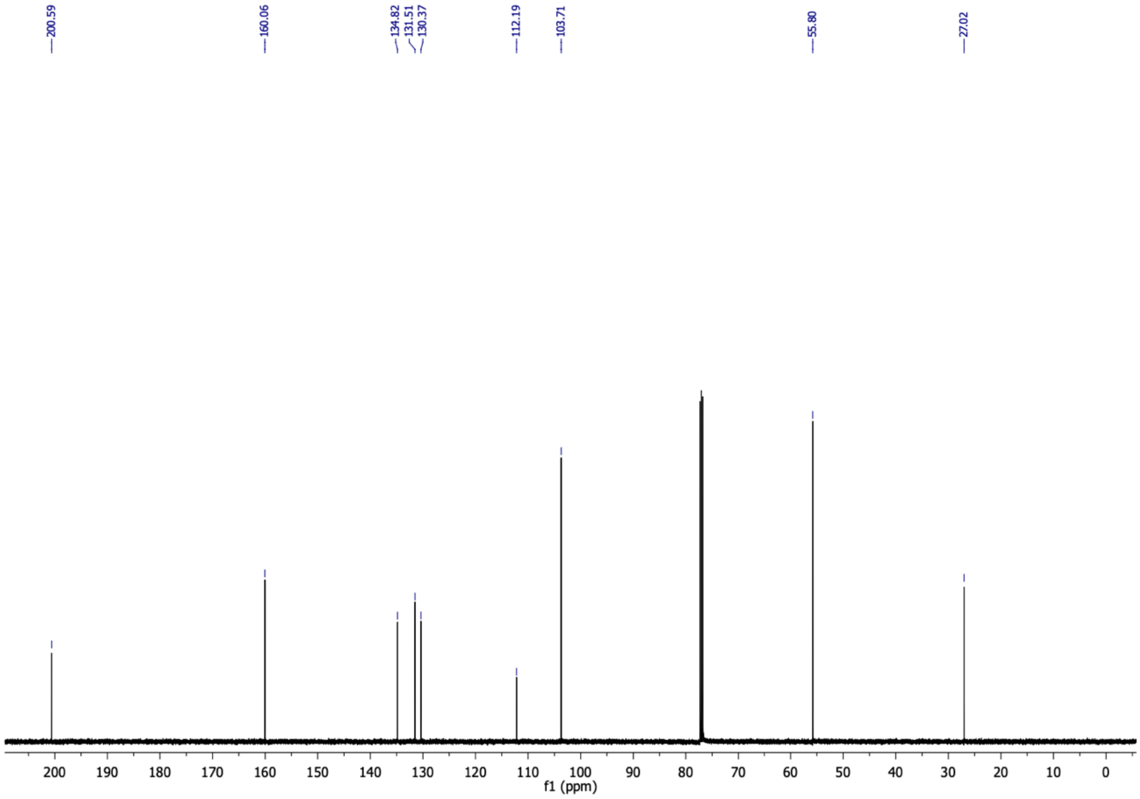

5-(2,6-dimethoxyphenyl)cyclohexane-1,3-dione (**18c**) ^{1}H NMR (500 MHz, CD_3OD)

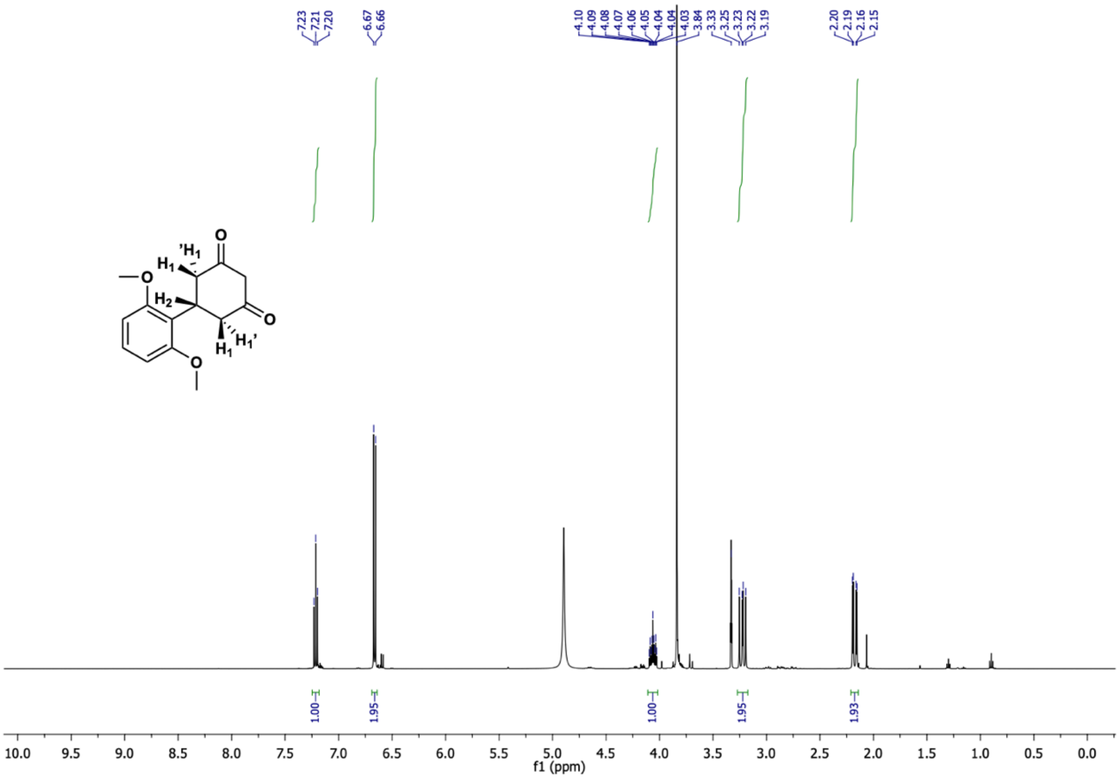

5-(2,6-dimethoxyphenyl)cyclohexane-1,3-dione (**18c**) ^{13}C NMR (126 MHz, CD_3OD)

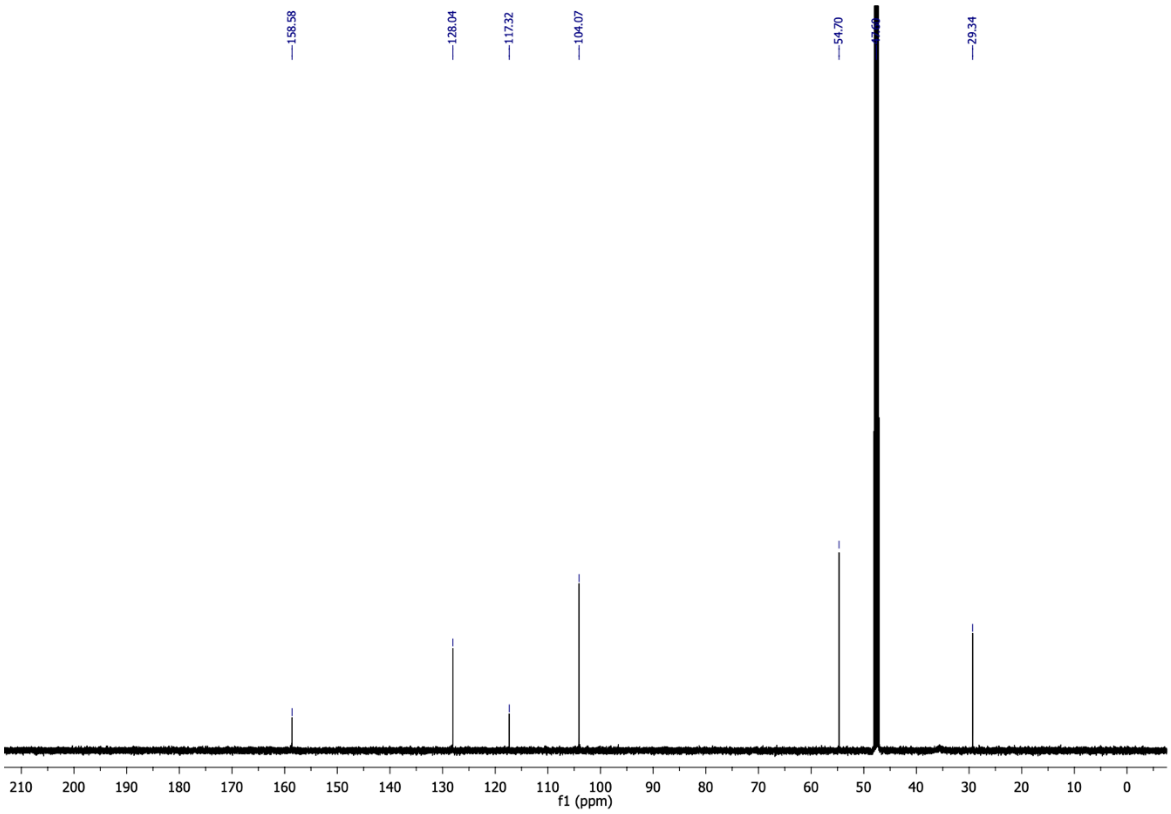

6-(2,6-dimethoxyphenyl)-4-oxo-4,5,6,7-tetrahydrobenzofuran-3-carboxylic acid (**18**) ^{1}H NMR (500 MHz, $CDCl_3$)

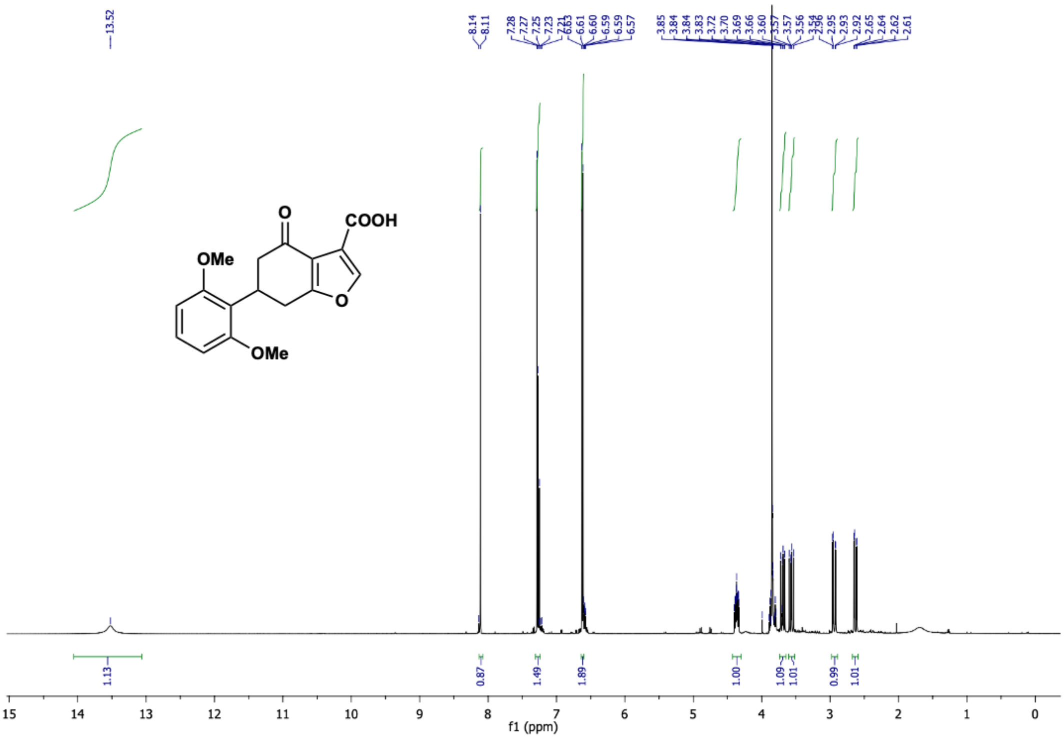

6-(2,6-dimethoxyphenyl)-4-oxo-4,5,6,7-tetrahydrobenzofuran-3-carboxylic acid (**18**) ^{13}C NMR (126 MHz, $CDCl_3$)

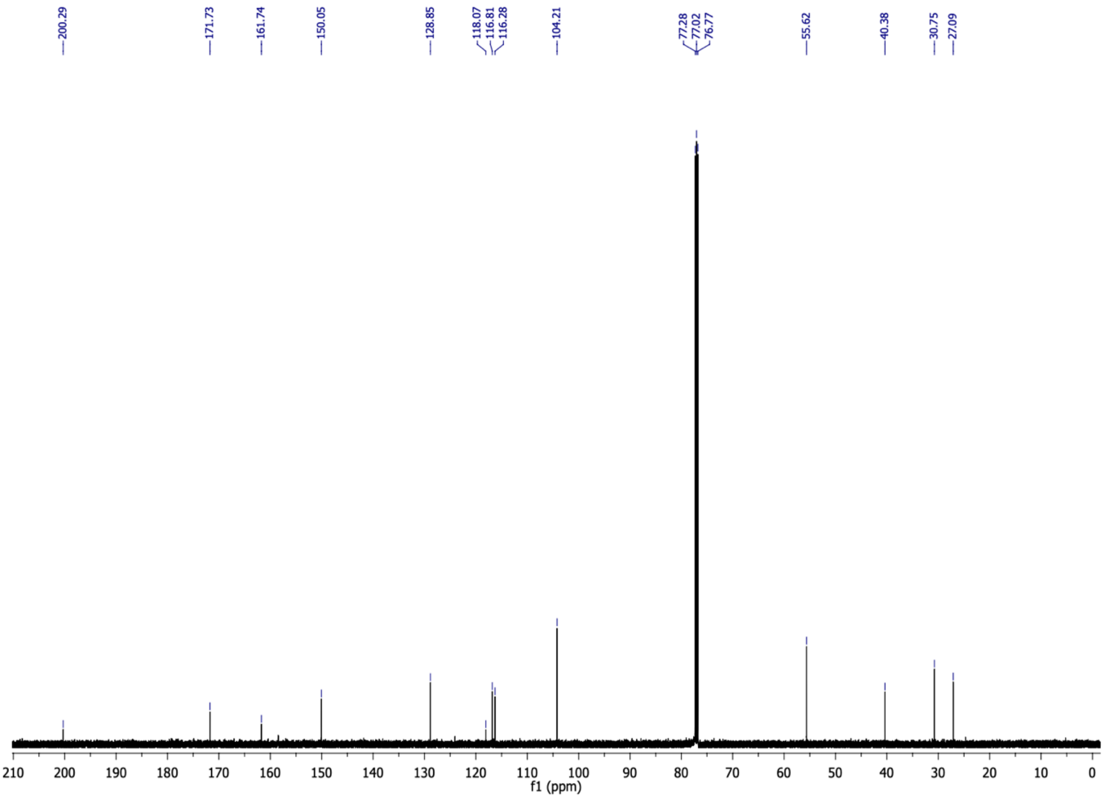

(E)-4-(2-bromo-6-chlorophenyl)but-3-en-2-one (**19b**) ^{1}H NMR (500 MHz, $CDCl_3$)

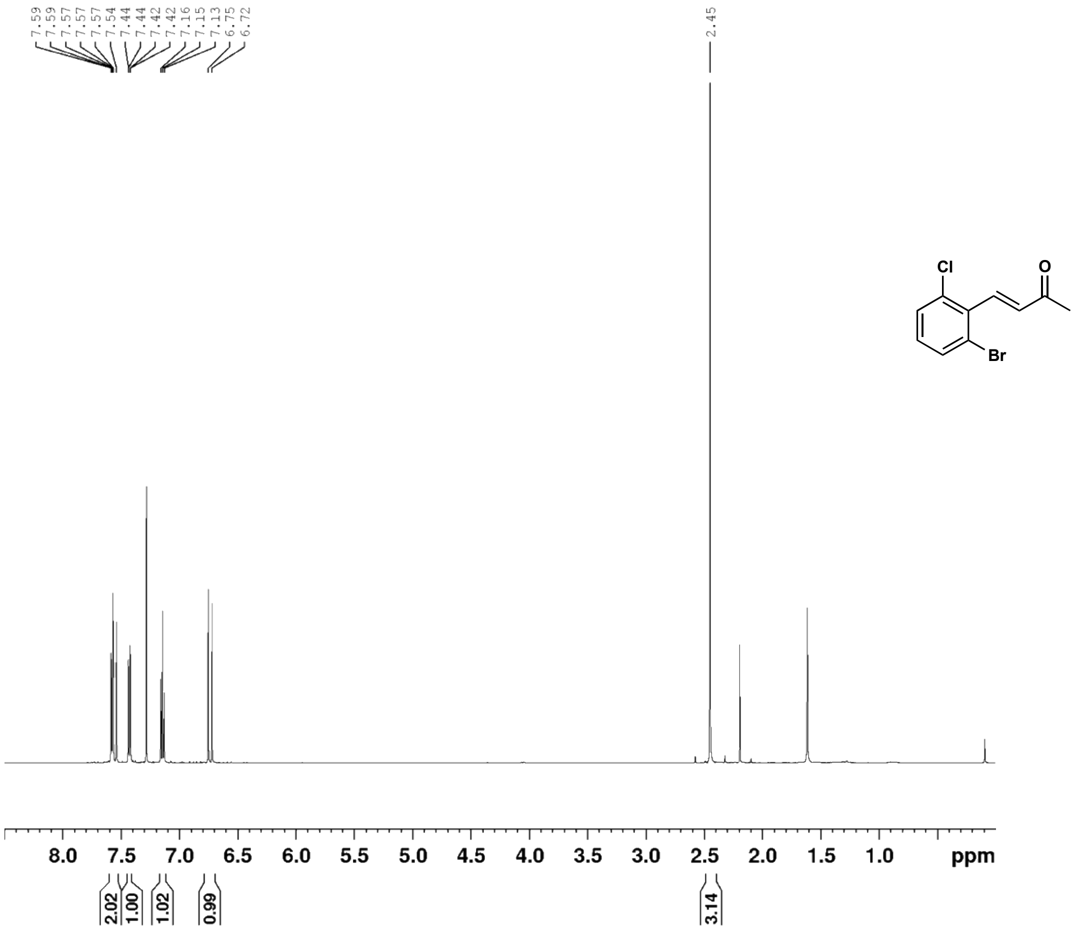

(E)-4-(2-bromo-6-chlorophenyl)but-3-en-2-one (**19b**) ^{13}C NMR (126 MHz, $CDCl_3$)

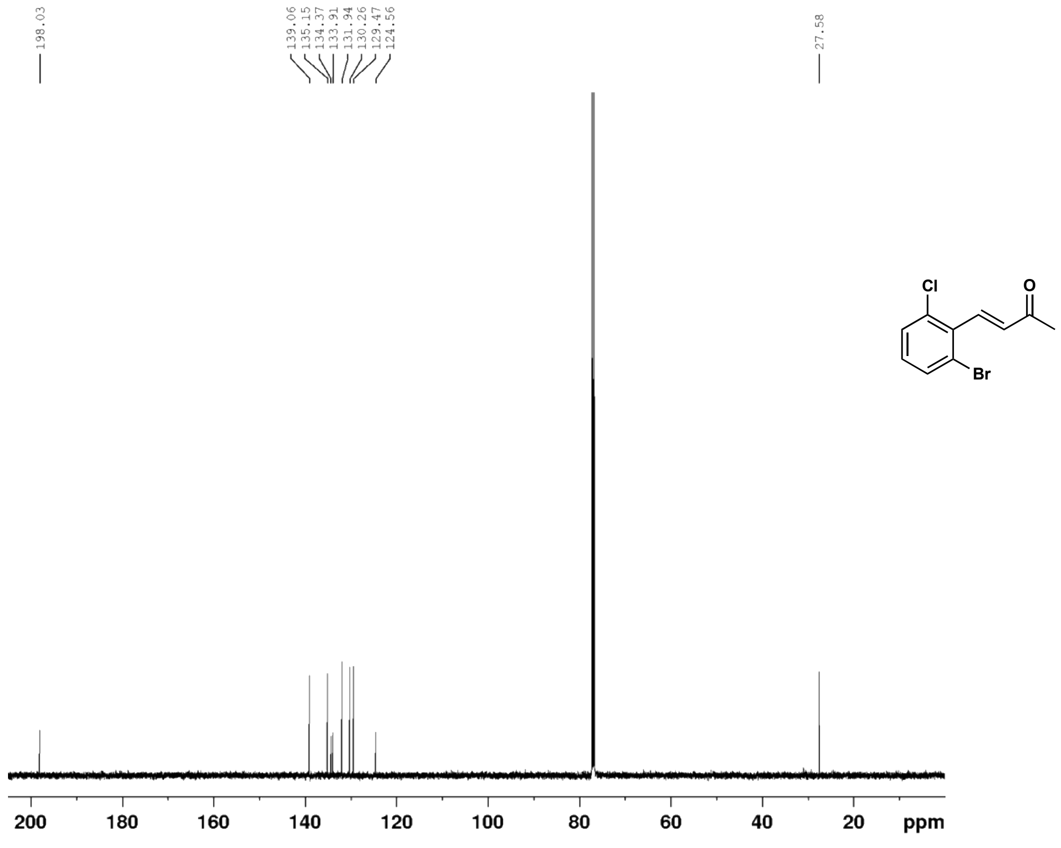

5-(2-bromo-6-chlorophenyl)cyclohexane-1,3-dione (**19c**) ^{1}H NMR (500 MHz, CD_3OD)

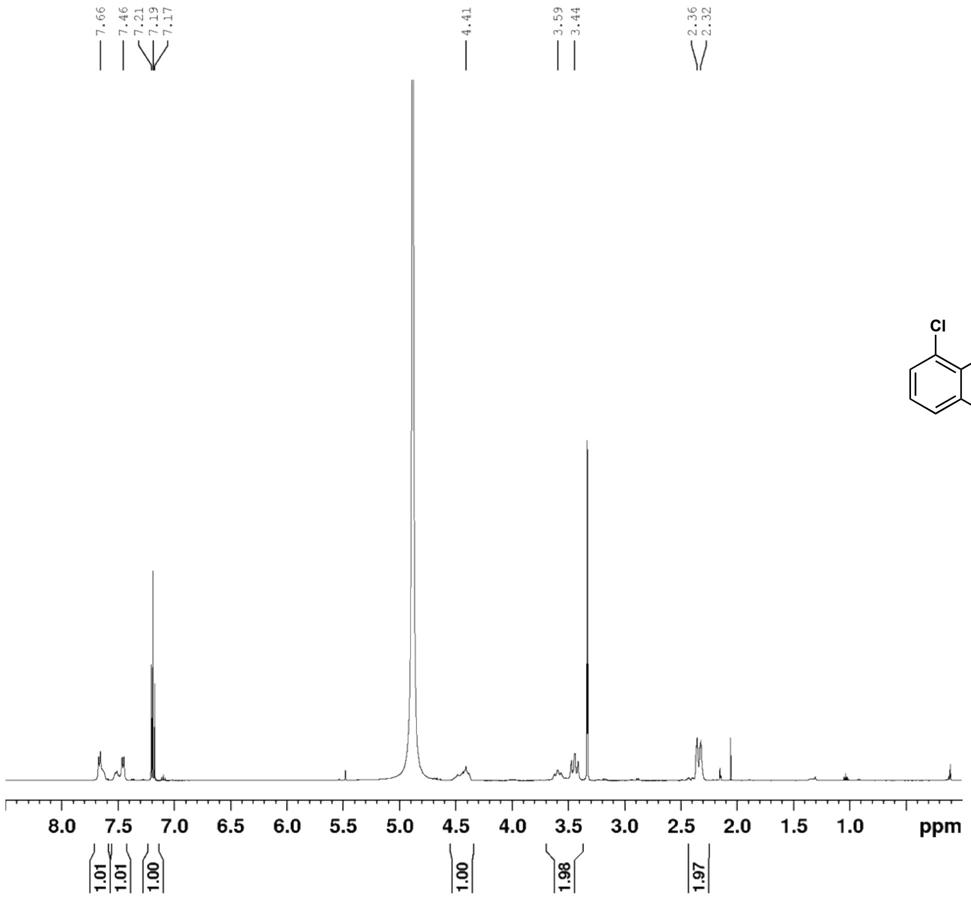

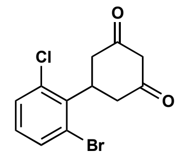

5-(2-bromo-6-chlorophenyl)cyclohexane-1,3-dione (**19c**) ^{13}C NMR (126 MHz, CD_3OD)

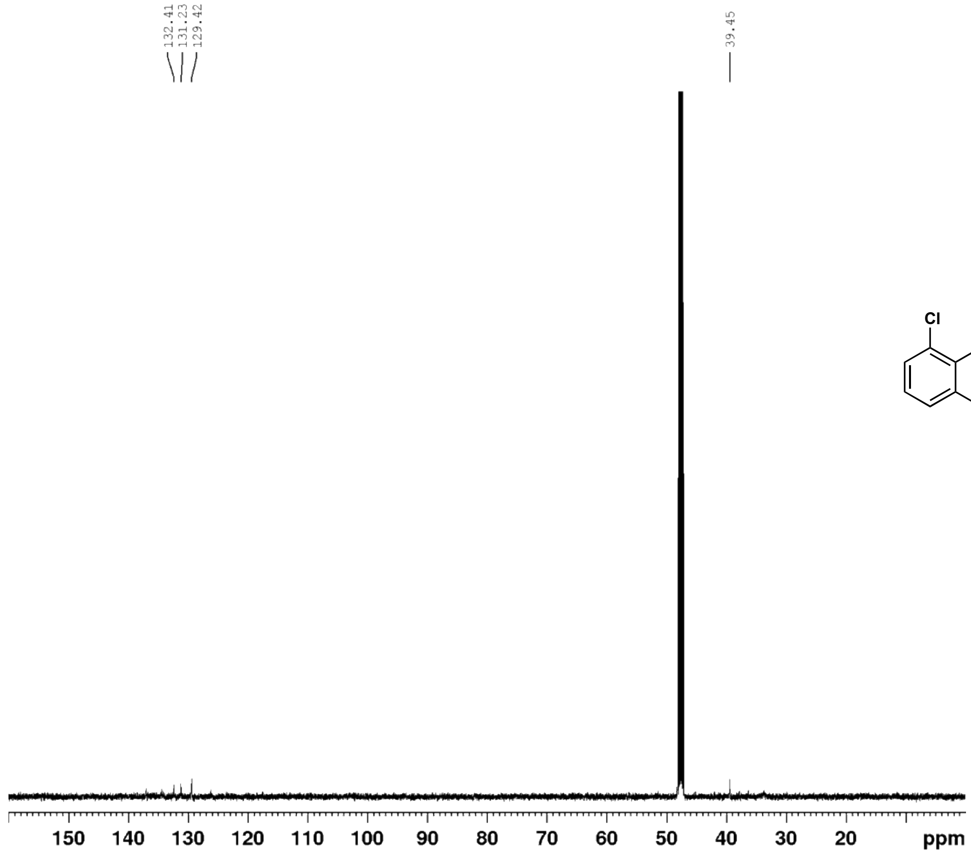

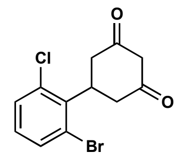

6-(2-bromo-6-chlorophenyl)-4-oxo-4,5,6,7-tetrahydrobenzofuran-3-carboxylic acid (**19**) ^{1}H NMR (500 MHz, $CDCl_3$)

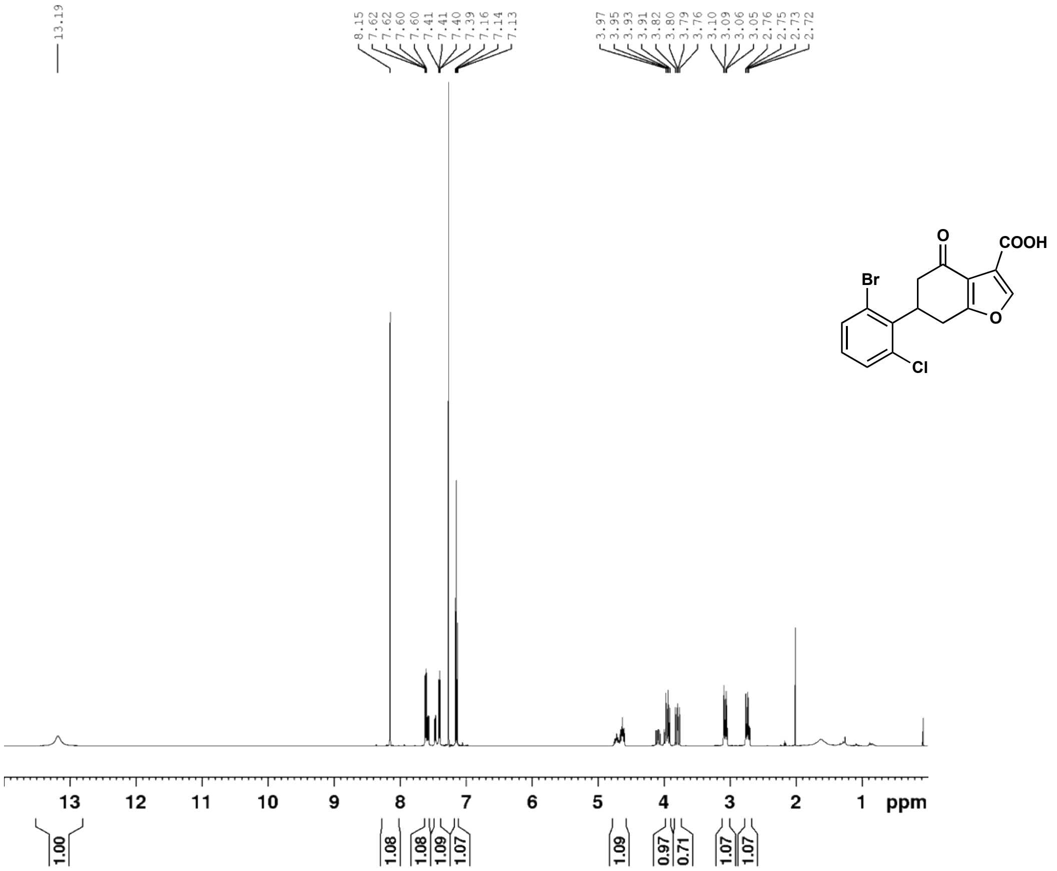

6-(2-bromo-6-chlorophenyl)-4-oxo-4,5,6,7-tetrahydrobenzofuran-3-carboxylic acid (**19**) ^{13}C NMR (126 MHz, $CDCl_3$)

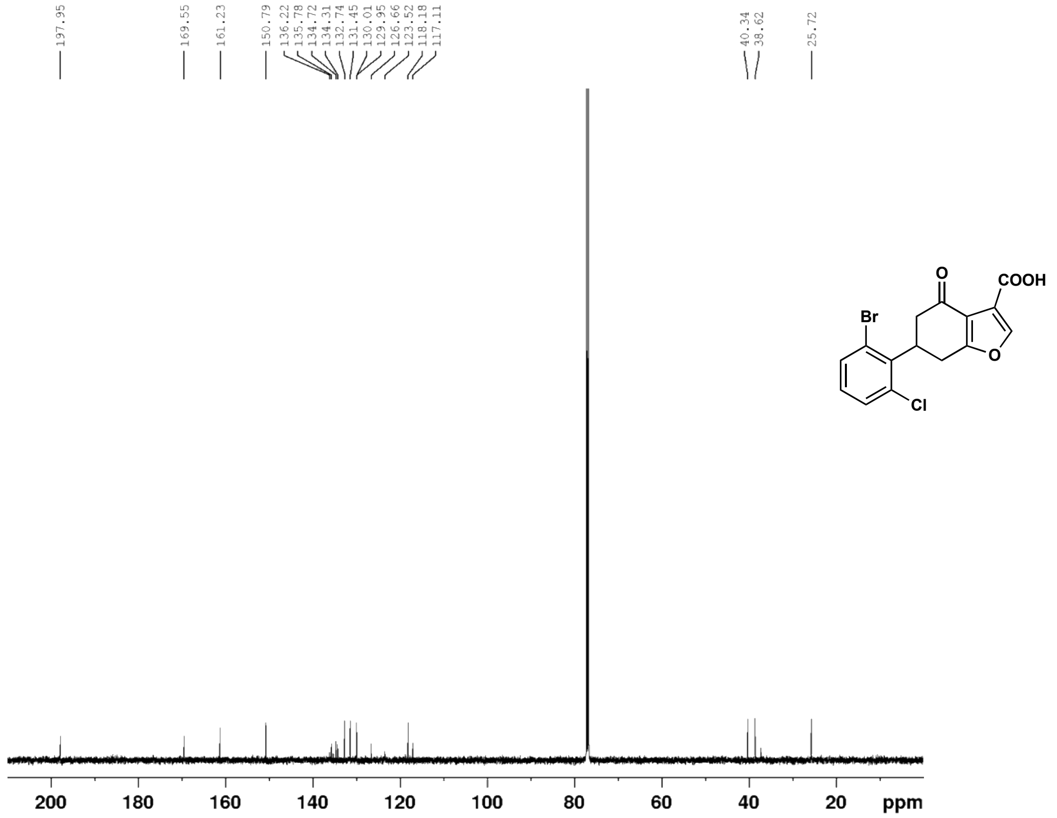

2-bromo-6-butoxybenzaldehyde (**20a**) ^{1}H NMR (500 MHz, $CDCl_3$)

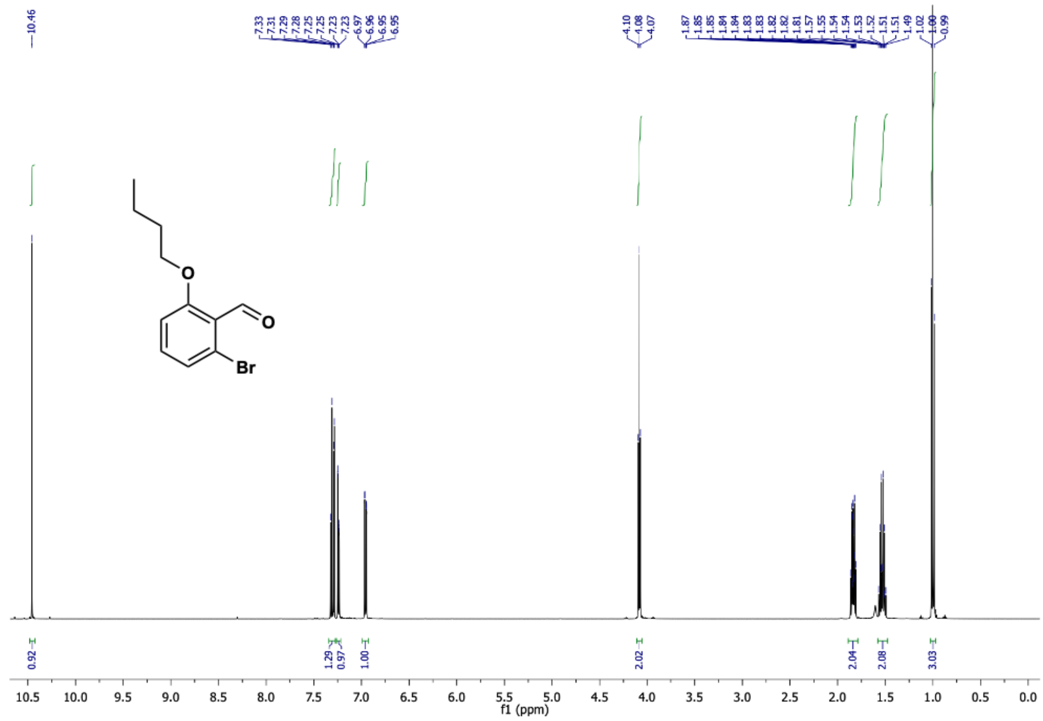

2-bromo-6-butoxybenzaldehyde (**20a**) ^{13}C NMR (126 MHz, $CDCl_3$)

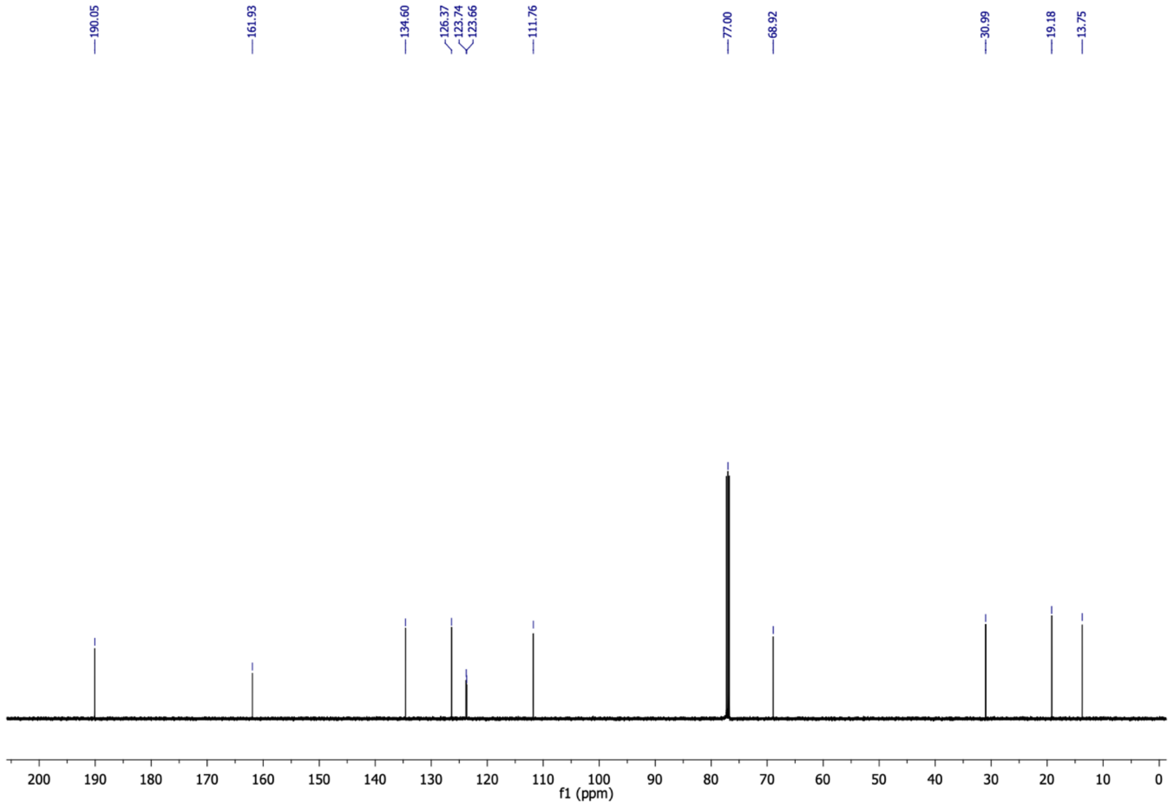

(*E*)-4-(2-bromo-6-butoxyphenyl)but-3-en-2-one (**20b**) ^{1}H NMR (500 MHz, $CDCl_3$)

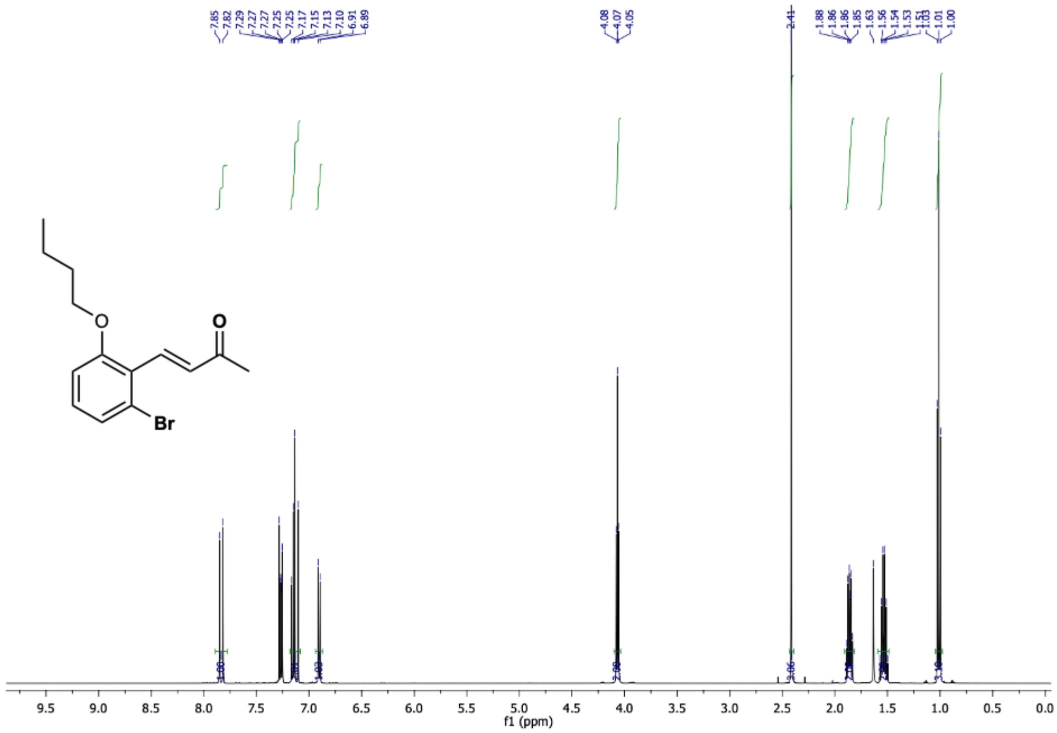

(*E*)-4-(2-bromo-6-butoxyphenyl)but-3-en-2-one (**20b**) ^{13}C NMR (126 MHz, $CDCl_3$)

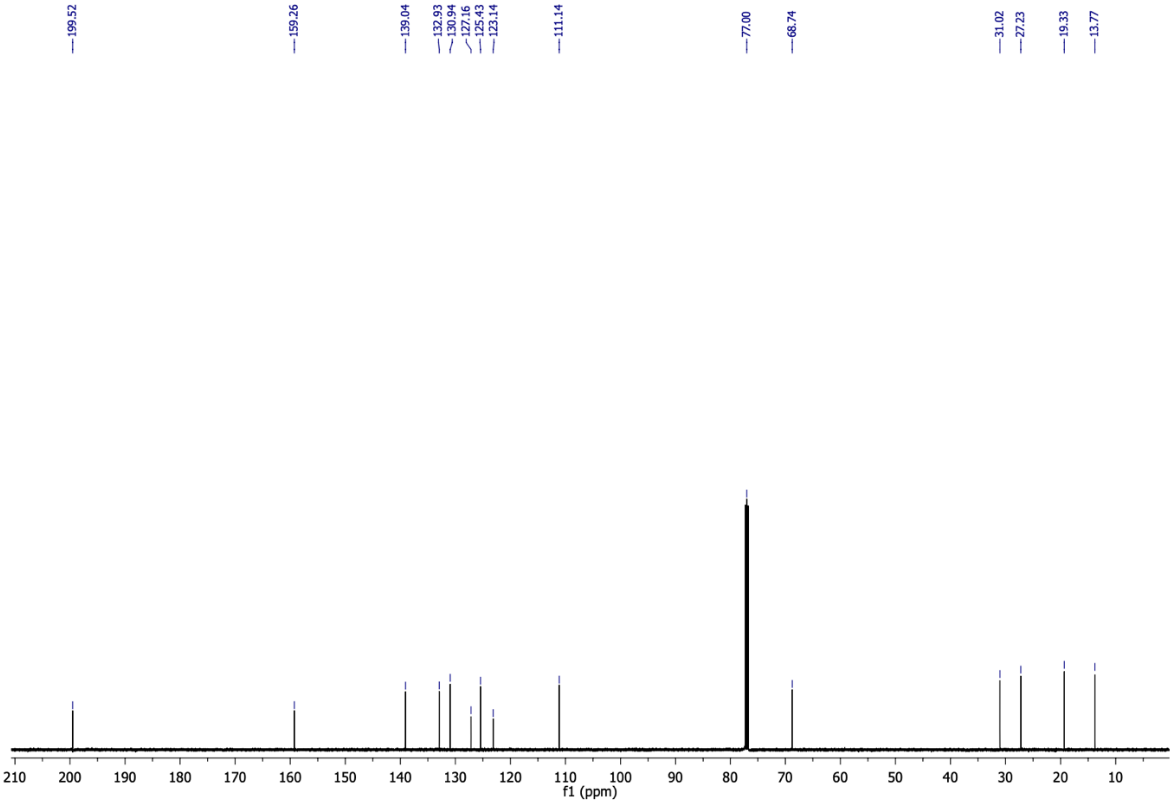

5-(2-bromo-6-butoxyphenyl)cyclohexane-1,3-dione (**20c**) ^{1}H NMR (500 MHz, CD_3OD)

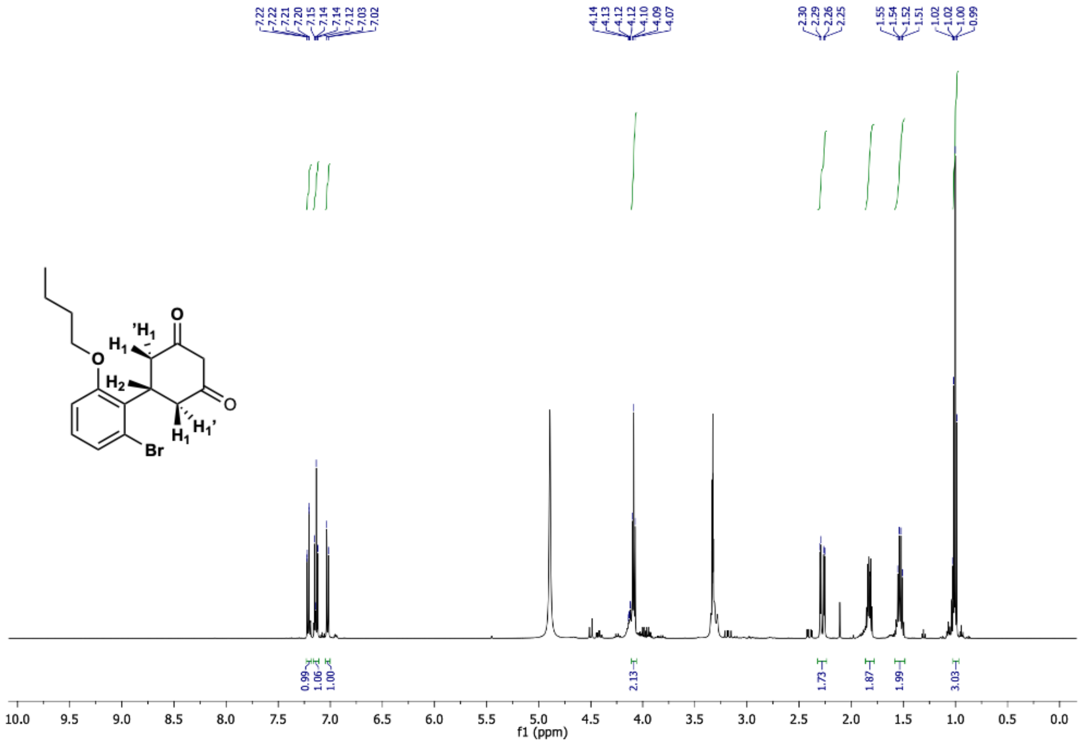

5-(2-bromo-6-butoxyphenyl)cyclohexane-1,3-dione (**20c**) ^{13}C NMR (126 MHz, CD_3OD)

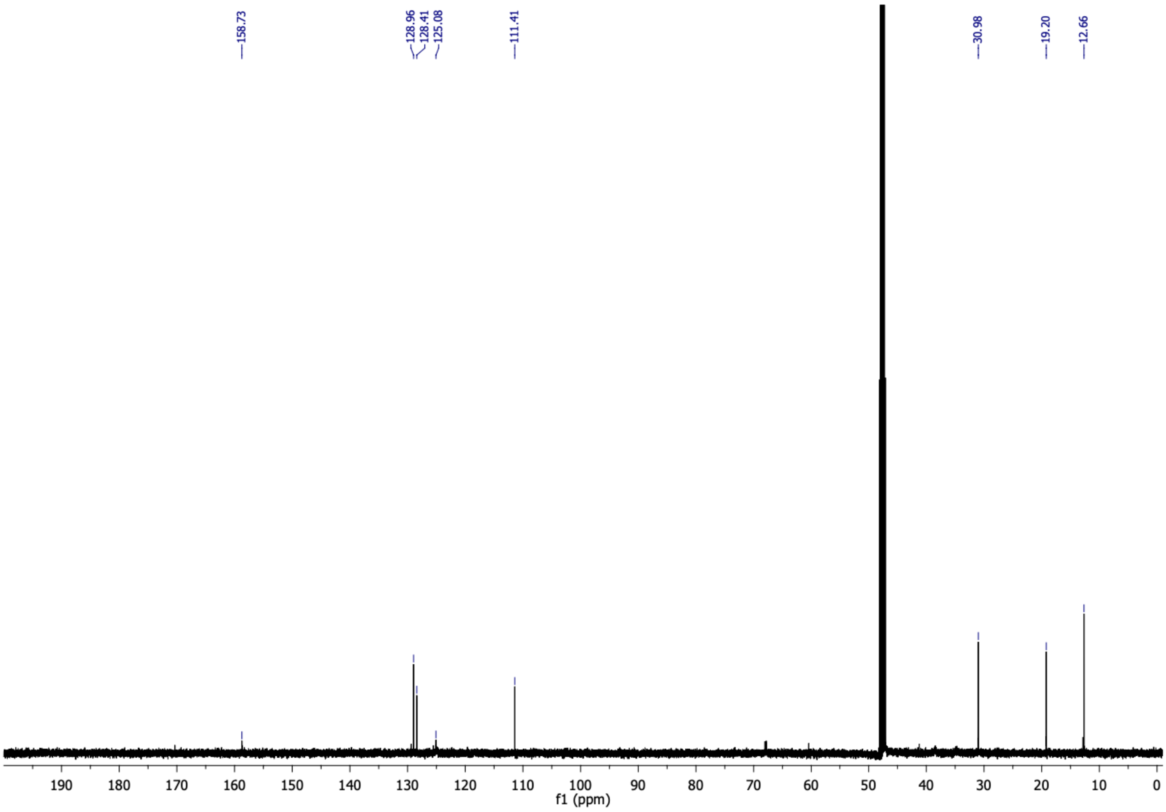

6-(2-bromo-6-butoxyphenyl)-4-oxo-4,5,6,7-tetrahydrobenzofuran-3-carboxylic acid (**20**) ^{1}H NMR (500 MHz, $CDCl_3$)

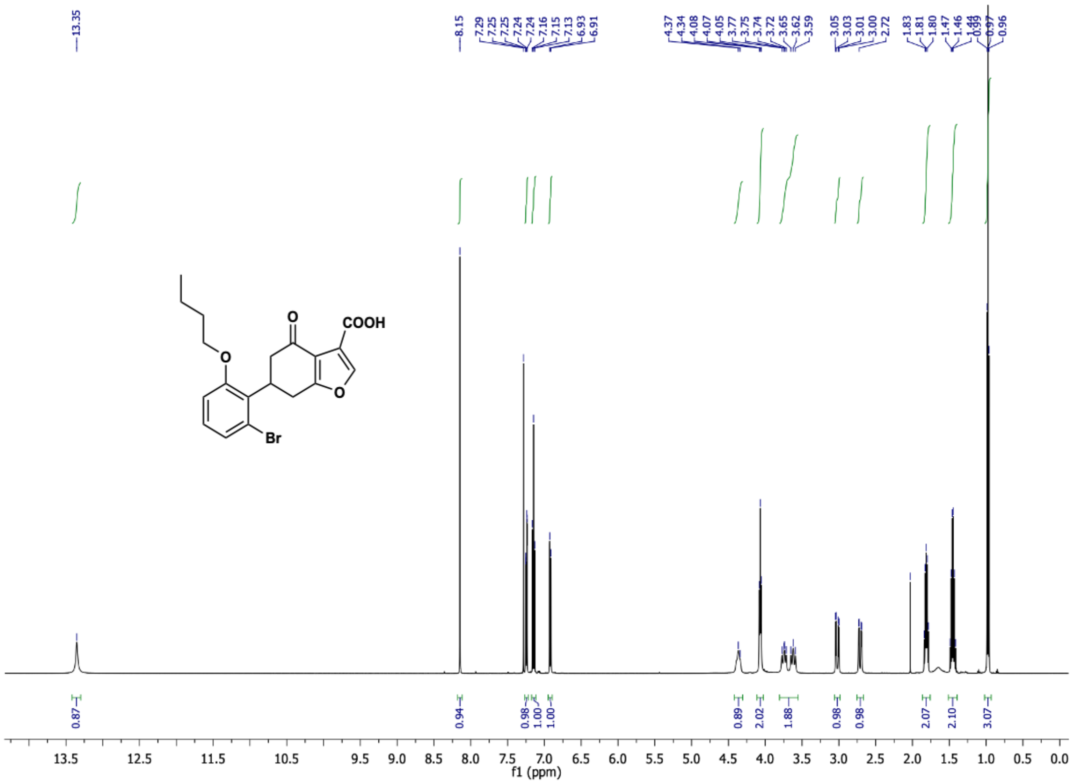

6-(2-bromo-6-butoxyphenyl)-4-oxo-4,5,6,7-tetrahydrobenzofuran-3-carboxylic acid (**20**) ^{13}C NMR (126 MHz, $CDCl_3$)

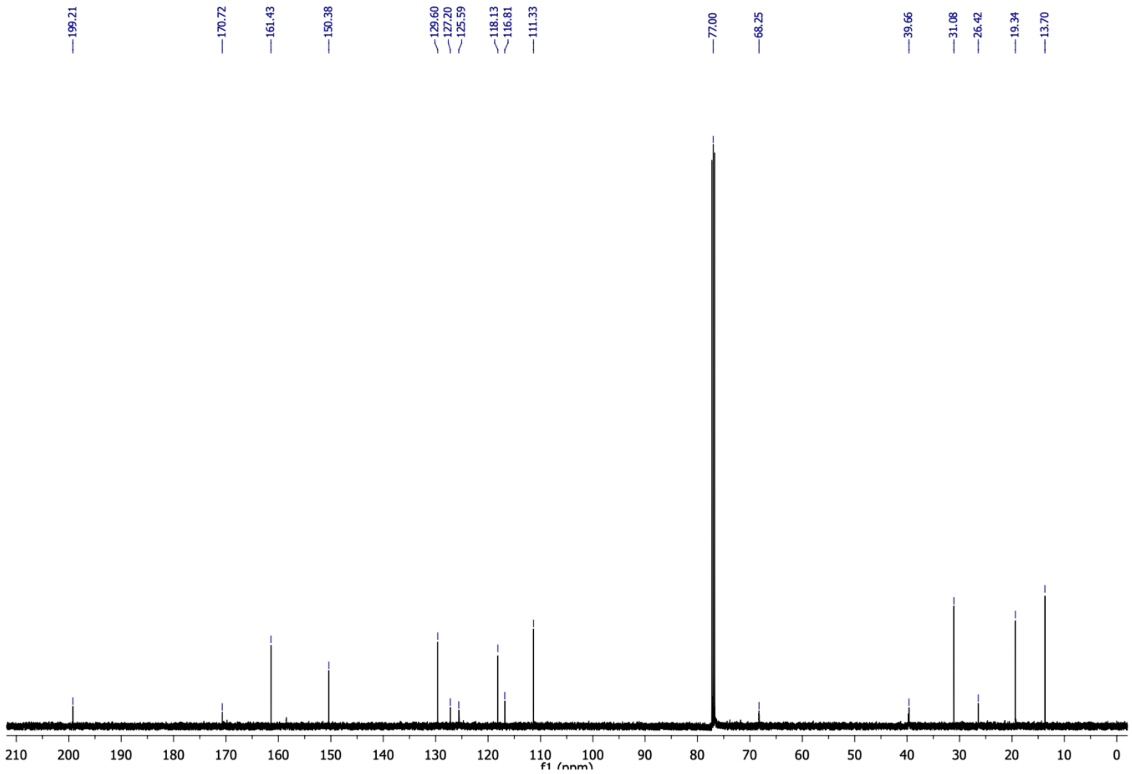

2-methoxy-6-(2-methoxyethoxy)benzaldehyde (**21a**) ^{1}H NMR (500 MHz, $CDCl_3$)

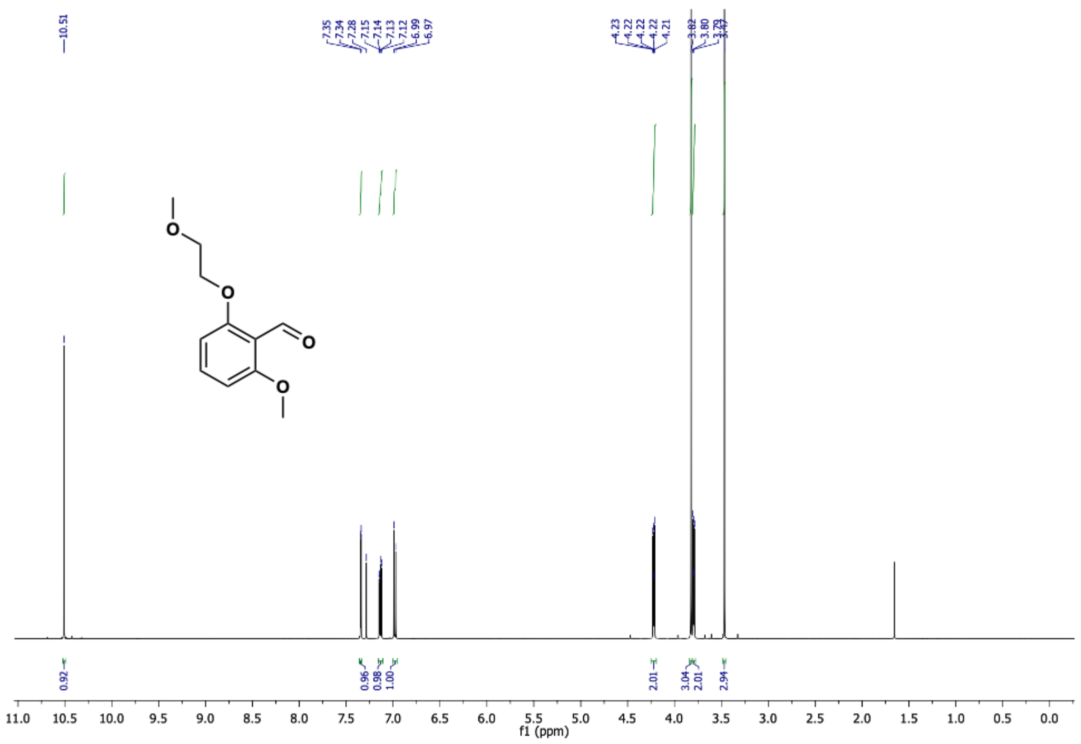

2-methoxy-6-(2-methoxyethoxy)benzaldehyde (**21a**) ^{13}C NMR (126 MHz, $CDCl_3$)

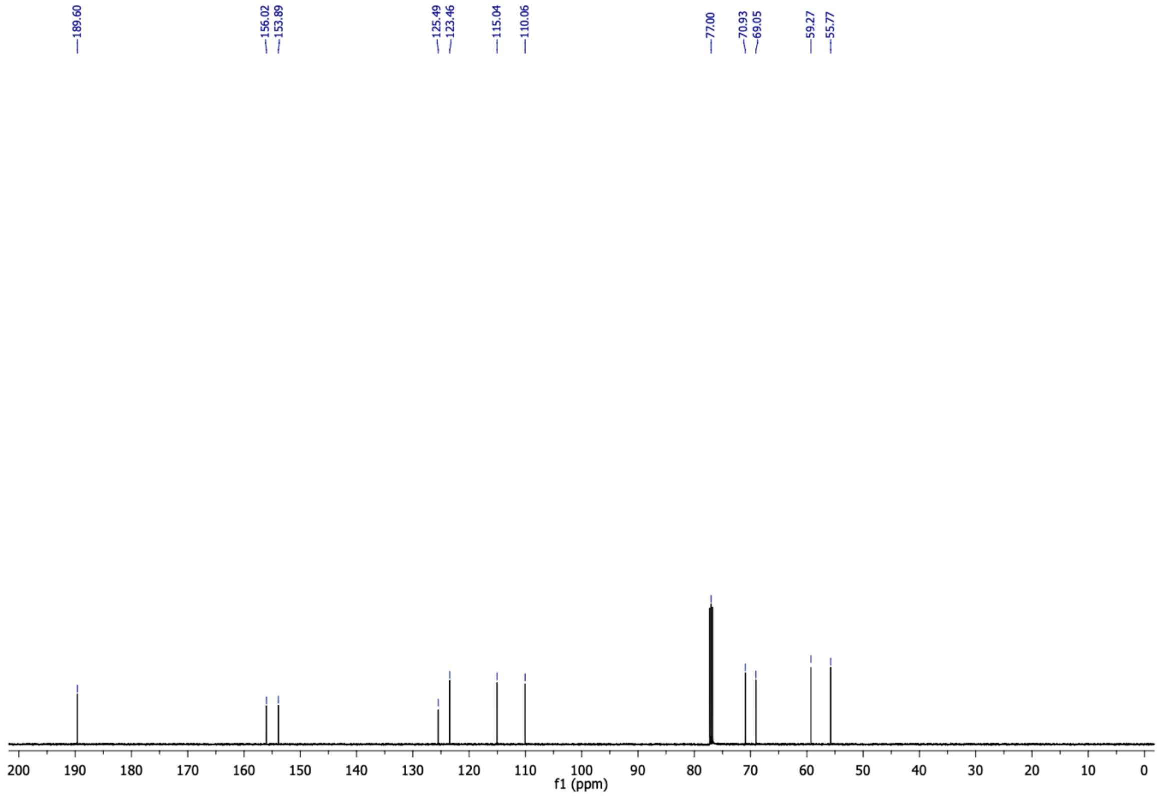

(*E*)-4-(2-methoxy-6-(2-methoxyethoxy)phenyl)but-3-en-2-one (**21b**) ^{1}H NMR (500 MHz, $CDCl_3$)

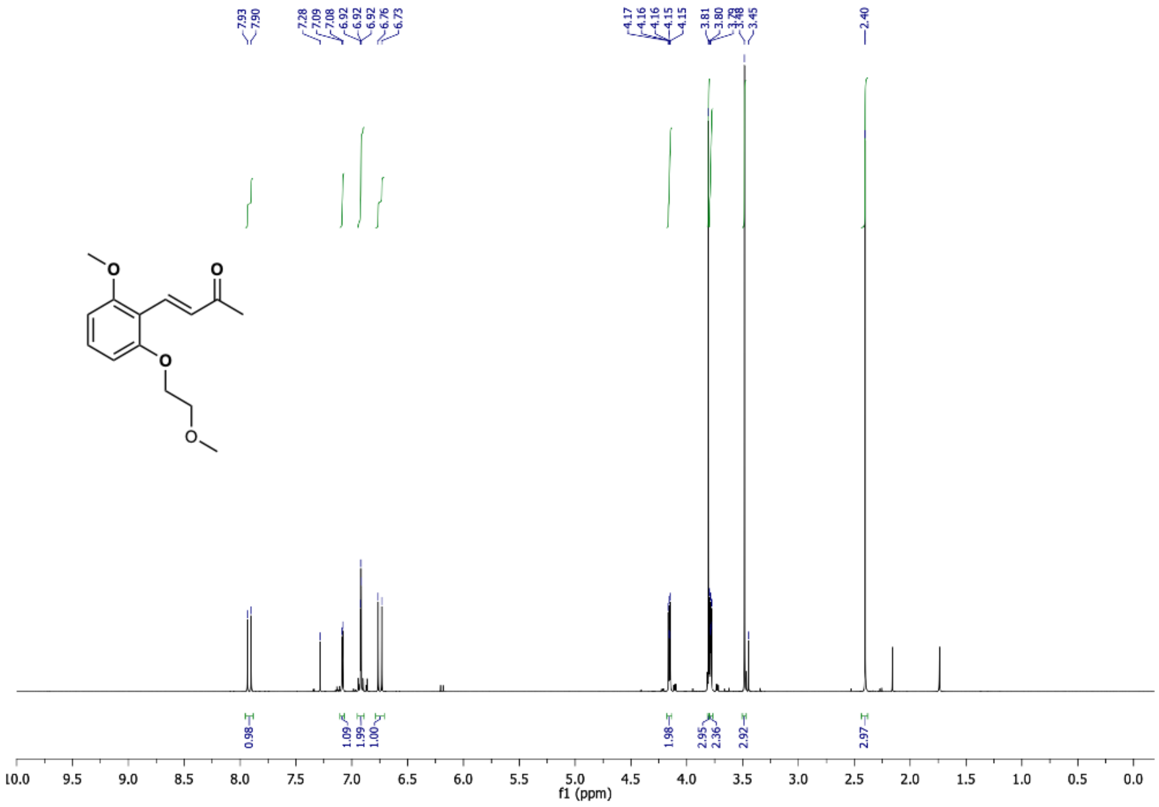

(*E*)-4-(2-methoxy-6-(2-methoxyethoxy)phenyl)but-3-en-2-one (**21b**) ^{13}C NMR (126 MHz, $CDCl_3$)

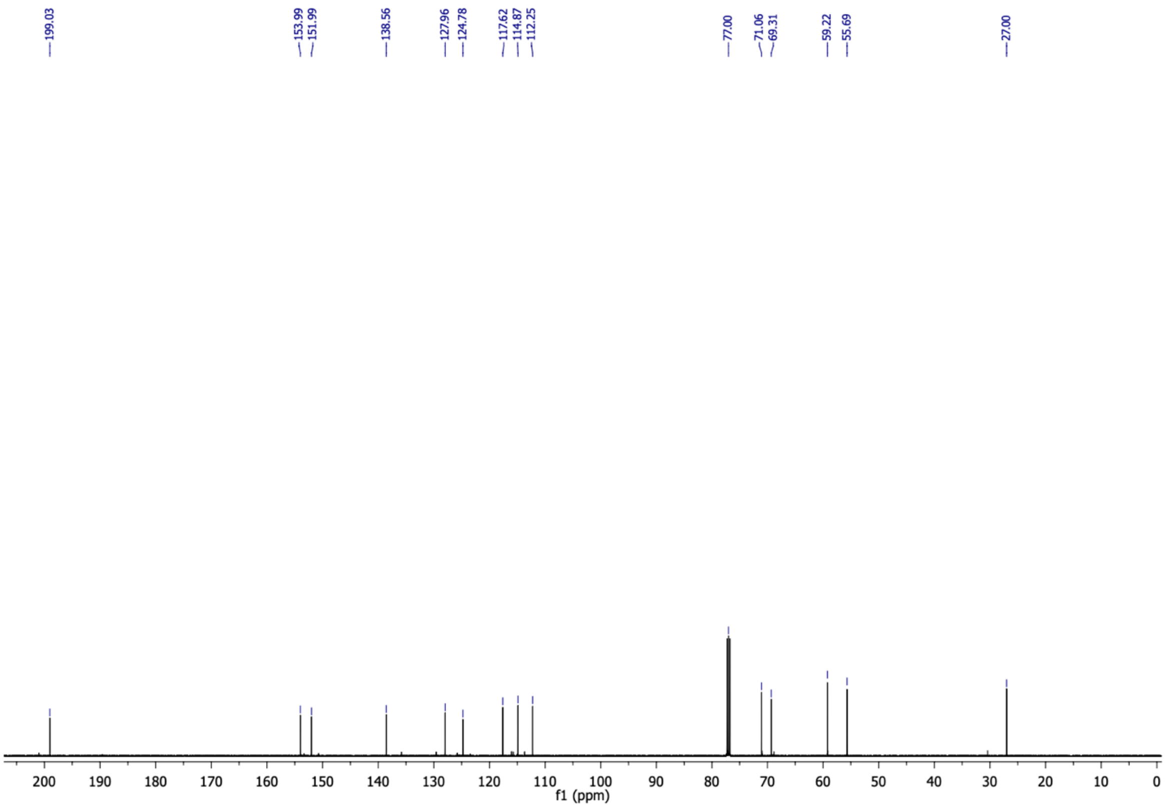

5-(2-methoxy-6-(2-methoxyethoxy)phenyl)cyclohexane-1,3-dione (**21c**) ^{1}H NMR (500 MHz, CD_3OD)

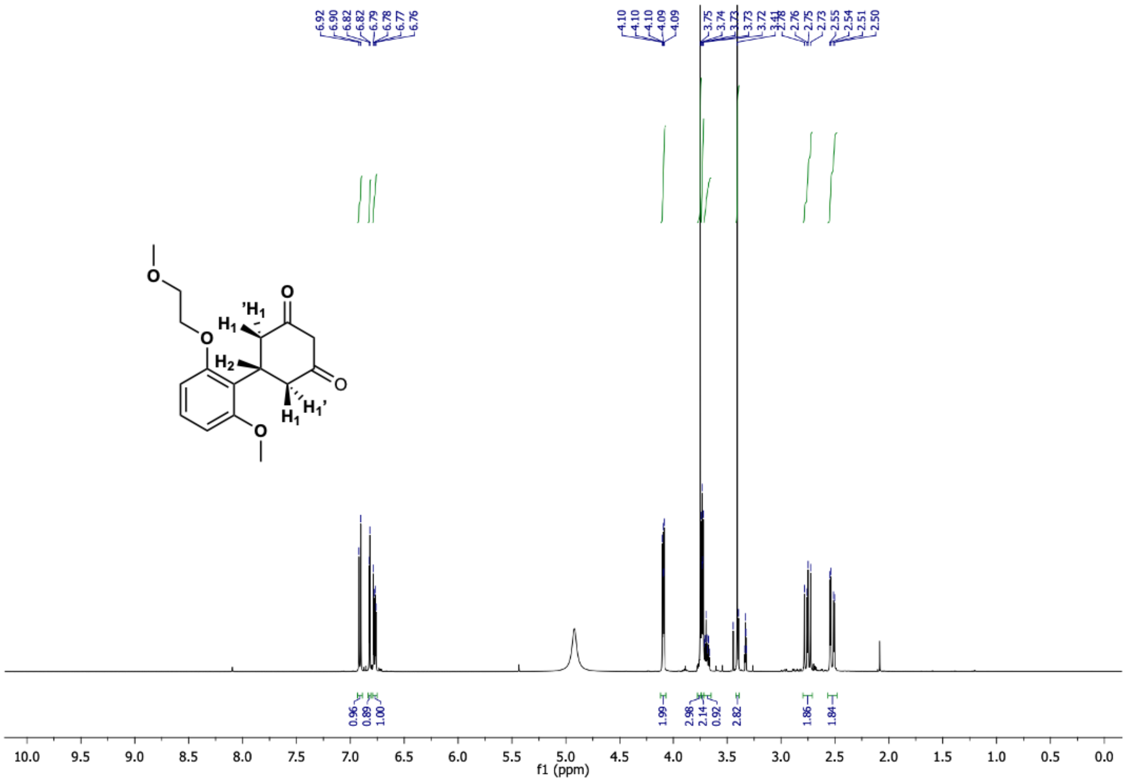

5-(2-methoxy-6-(2-methoxyethoxy)phenyl)cyclohexane-1,3-dione (**21c**) ^{13}C NMR (126 MHz, CD_3OD)

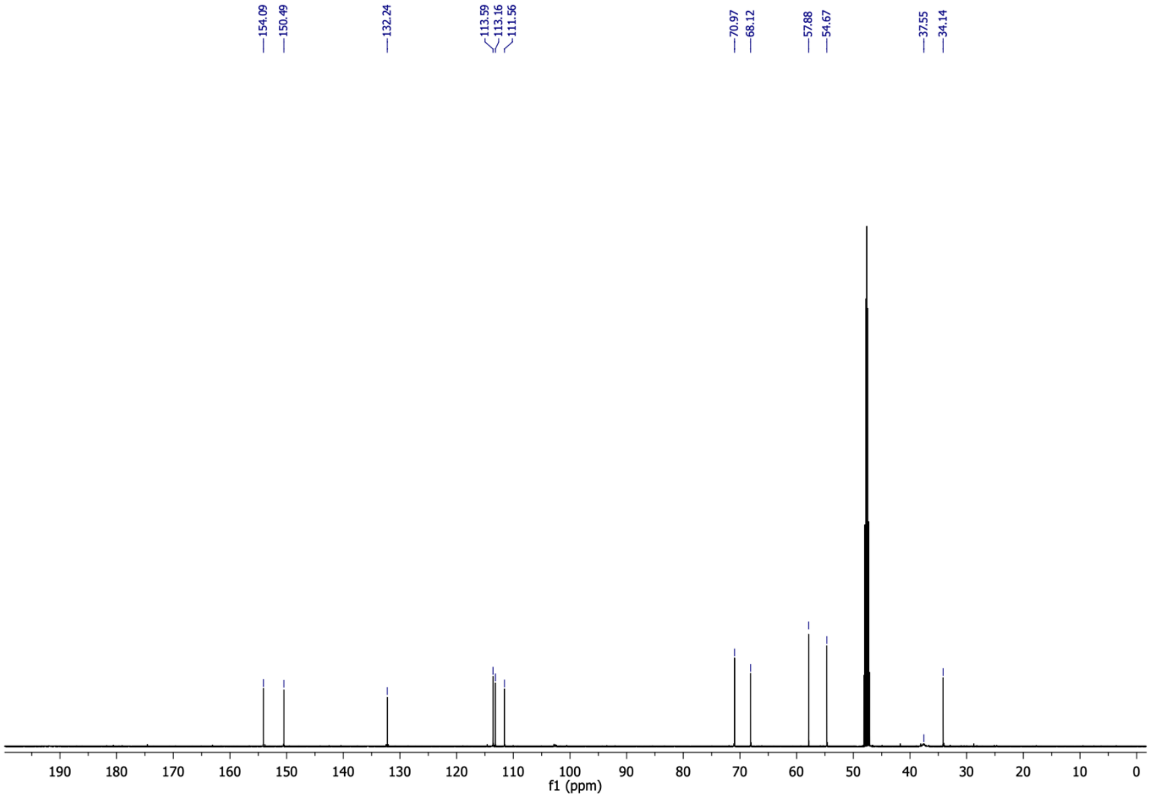

6-(2-methoxy-6-(2-methoxyethoxy)phenyl)-4-oxo-4,5,6,7-tetrahydrobenzofuran-3-carboxylic acid (**21**) ^{1}H NMR (500 MHz, $CDCl_3$)

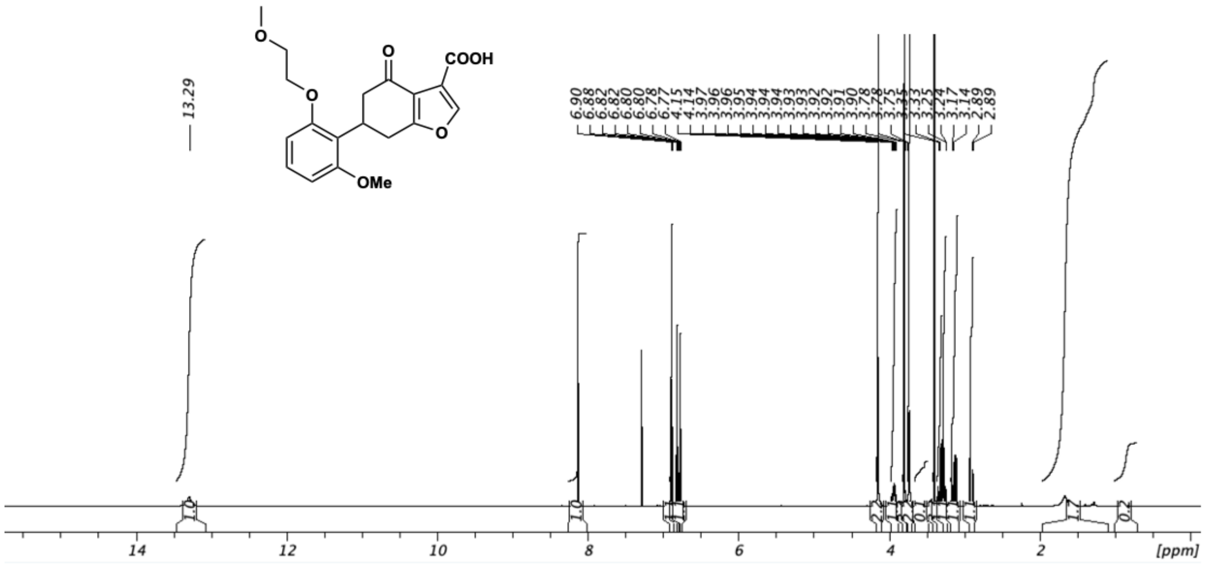

6-(2-methoxy-6-(2-methoxyethoxy)phenyl)-4-oxo-4,5,6,7-tetrahydrobenzofuran-3-carboxylic acid (**21**) ^{13}C NMR (126 MHz, $CDCl_3$)

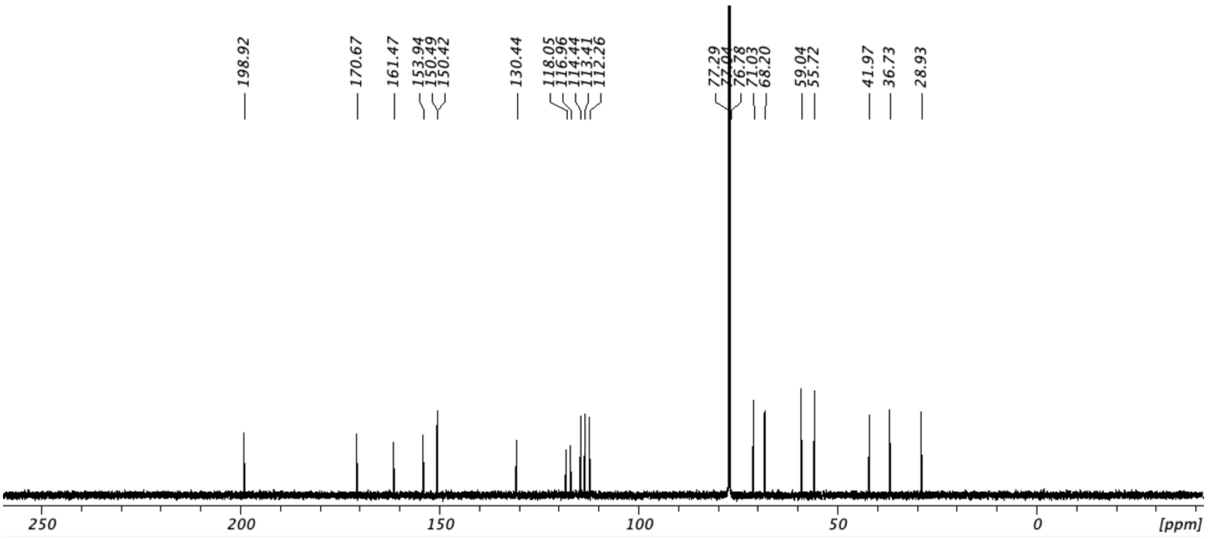

(*E*)-4-(2,3-dichlorophenyl)but-3-en-2-one (**22b**) ^{1}H NMR (500 MHz, $CDCl_3$)

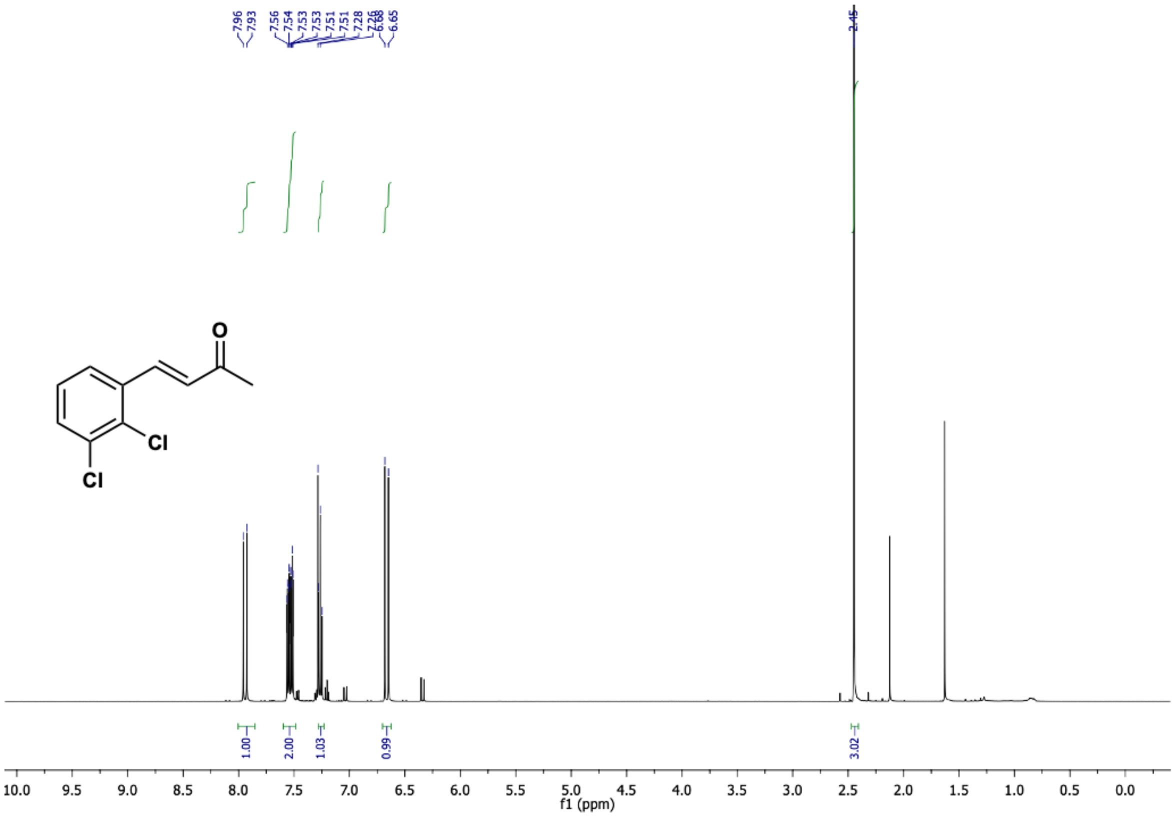

(*E*)-4-(2,3-dichlorophenyl)but-3-en-2-one (**22b**) ^{13}C NMR (126 MHz, $CDCl_3$)

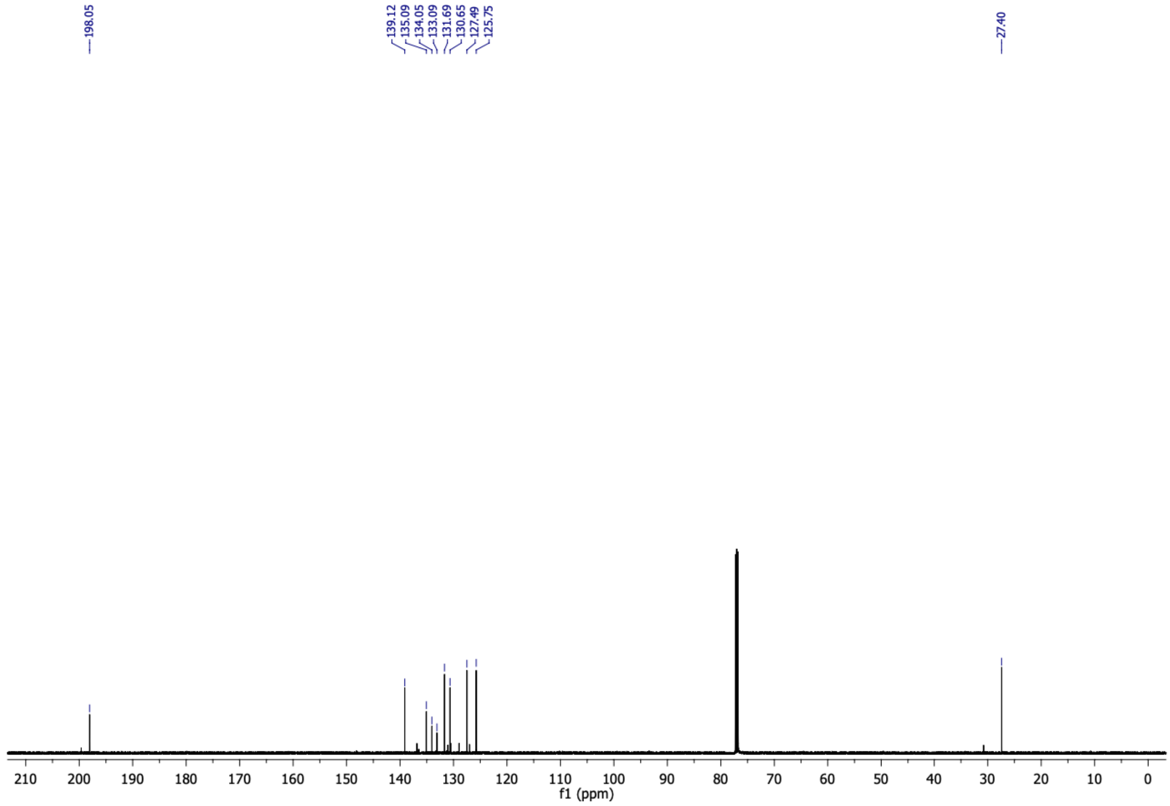

5-(2,3-dichlorophenyl)cyclohexane-1,3-dione (**22c**) ^{1}H NMR (500 MHz, d_6-DMSO)

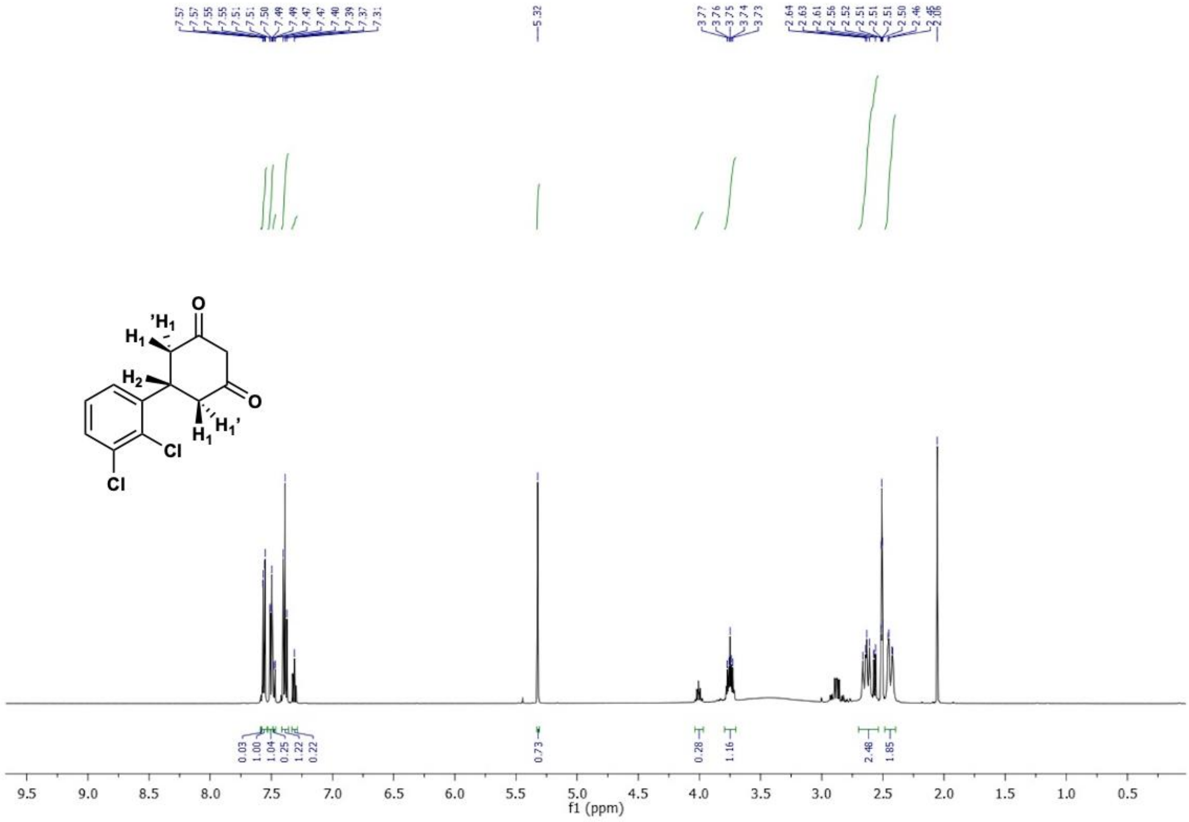

5-(2,3-dichlorophenyl)cyclohexane-1,3-dione (**22c**) ^{13}C NMR (126 MHz, d_6-DMSO)

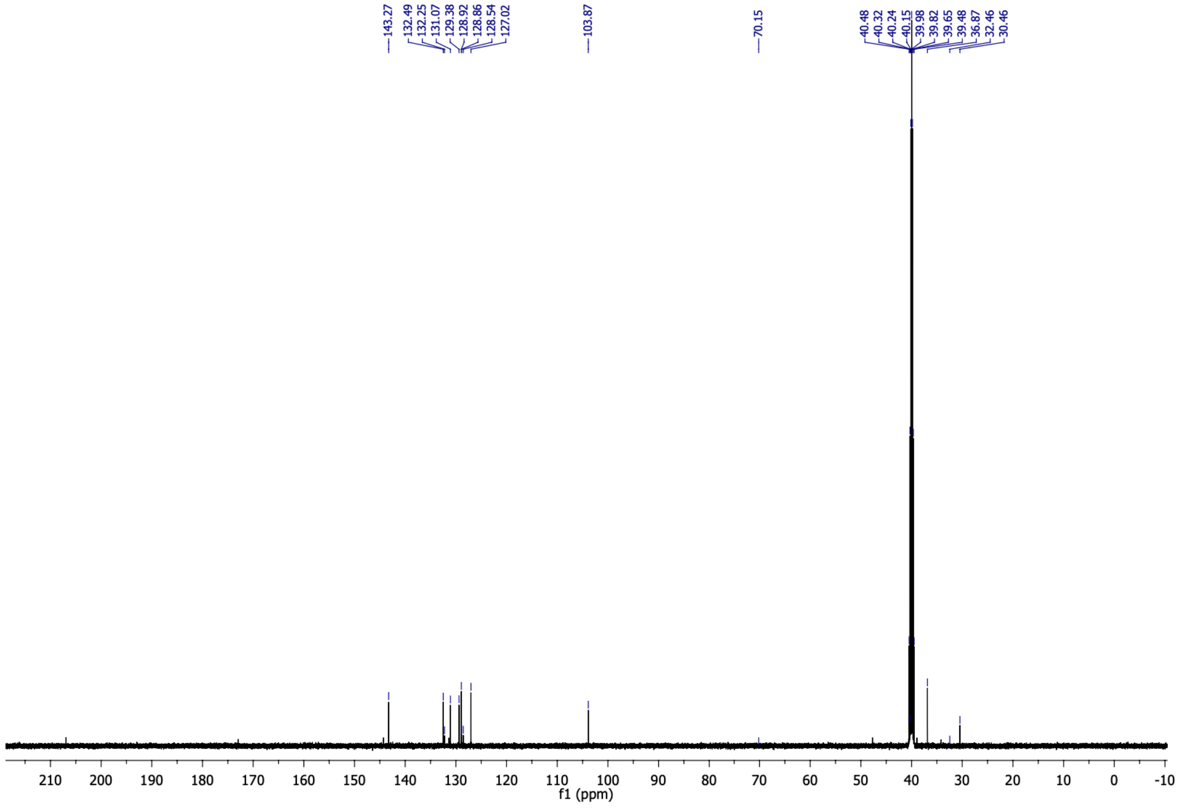

6-(2,3-dichlorophenyl)-4-oxo-4,5,6,7-tetrahydrobenzofuran-3-carboxylic acid (**22**) ^{1}H NMR (500 MHz, $CDCl_3$)

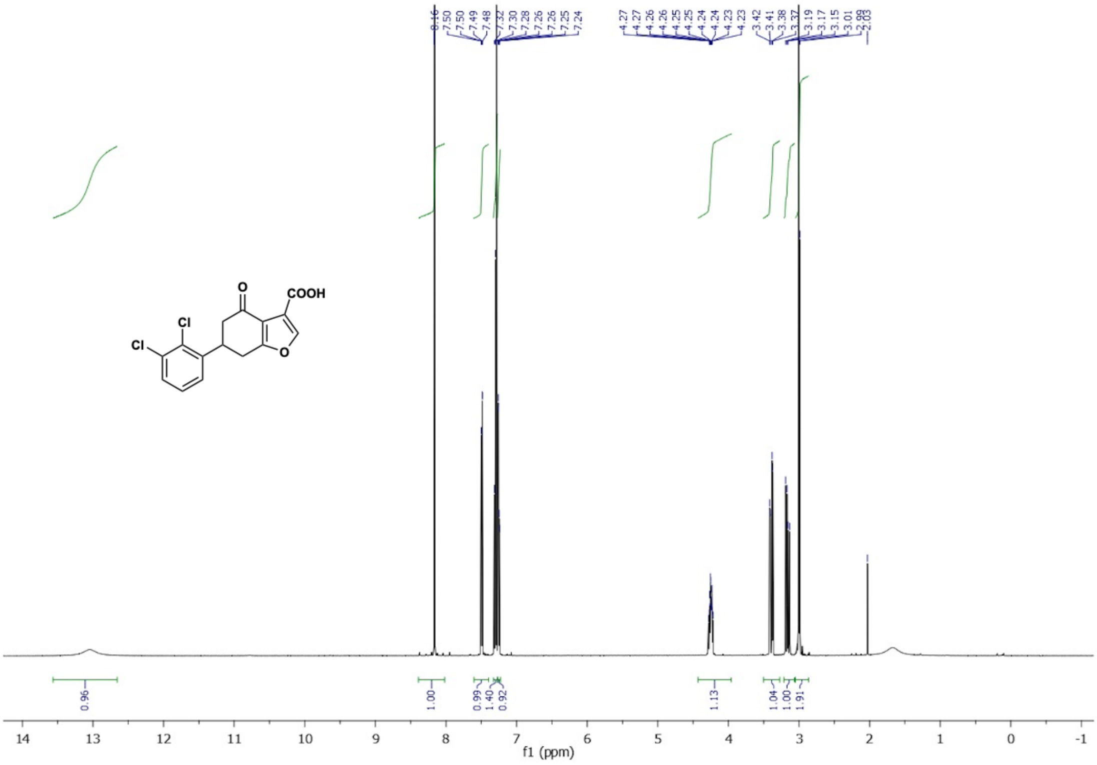

6-(2,3-dichlorophenyl)-4-oxo-4,5,6,7-tetrahydrobenzofuran-3-carboxylic acid (**22**) ^{13}C NMR (126 MHz, $CDCl_3$)

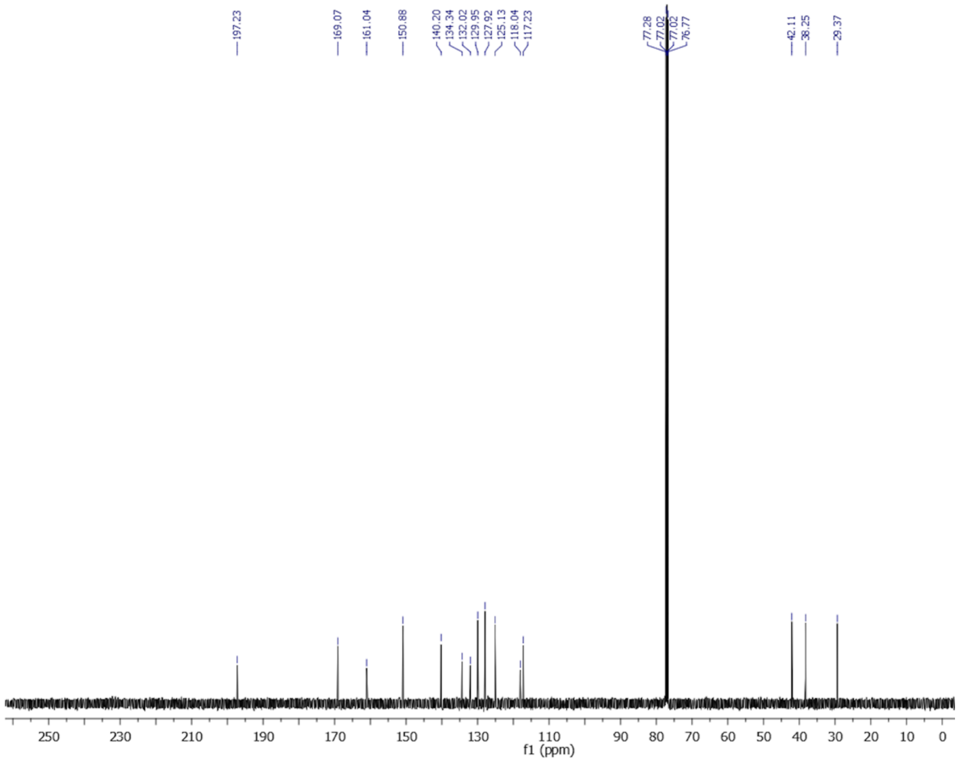

(E)-4-(2,3,6-trichlorophenyl)but-3-en-2-one (**23b**) ^{1}H NMR (500 MHz, $CDCl_3$)

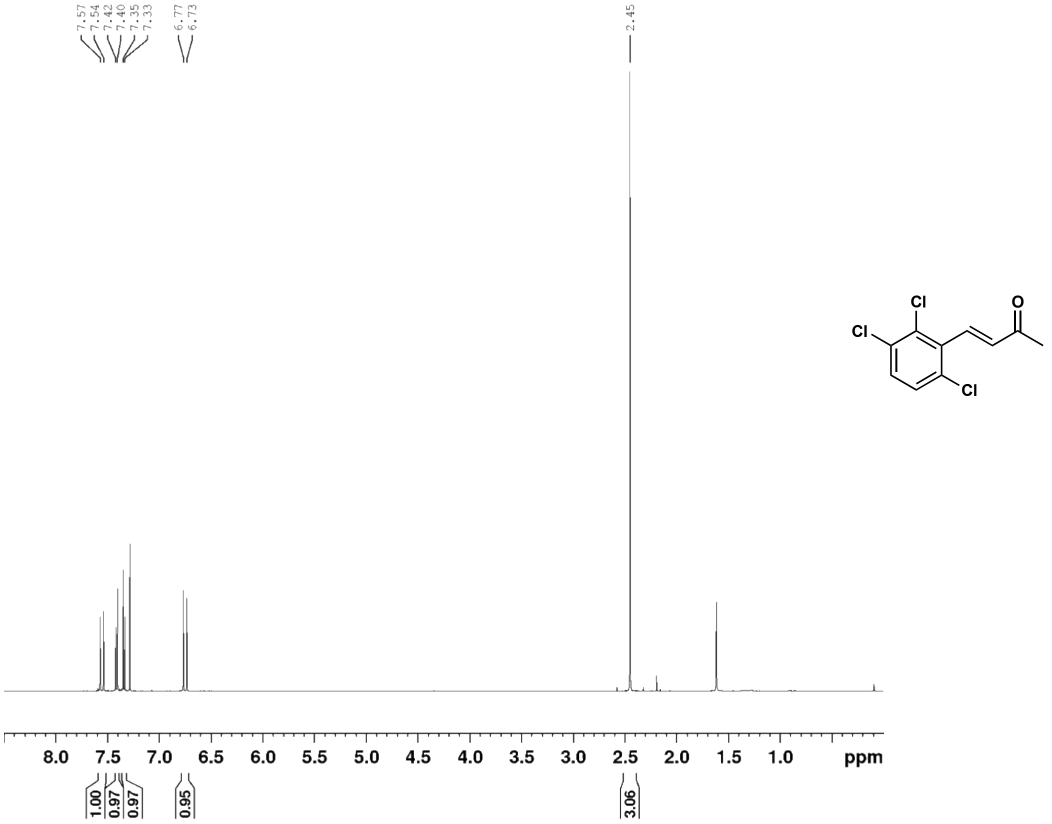

(E)-4-(2,3,6-trichlorophenyl)but-3-en-2-one (**23b**) ^{13}C NMR (126 MHz, $CDCl_3$)

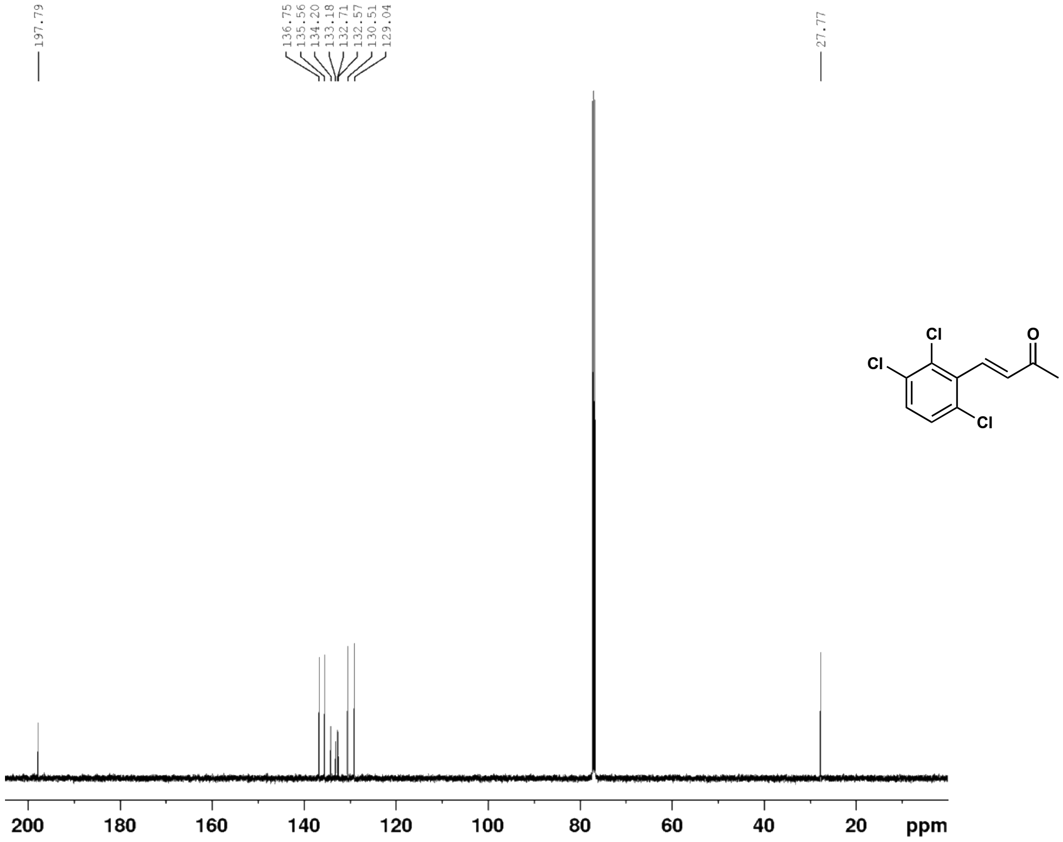

5-(2,3,6-trichlorophenyl)cyclohexane-1,3-dione (**23c**) ^{1}H NMR (500 MHz, CD_3OD)

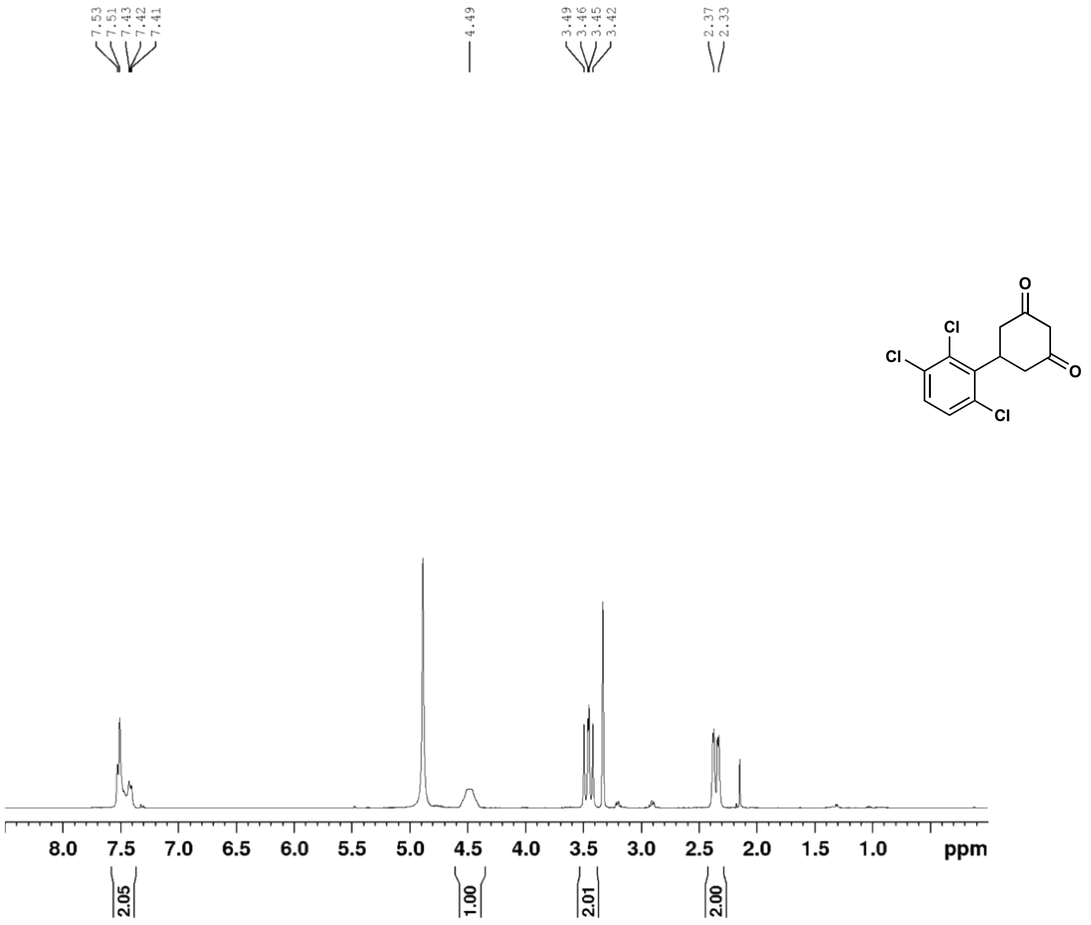

5-(2,3,6-trichlorophenyl)cyclohexane-1,3-dione (**23c**) ^{13}C NMR (126 MHz, CD_3OD)

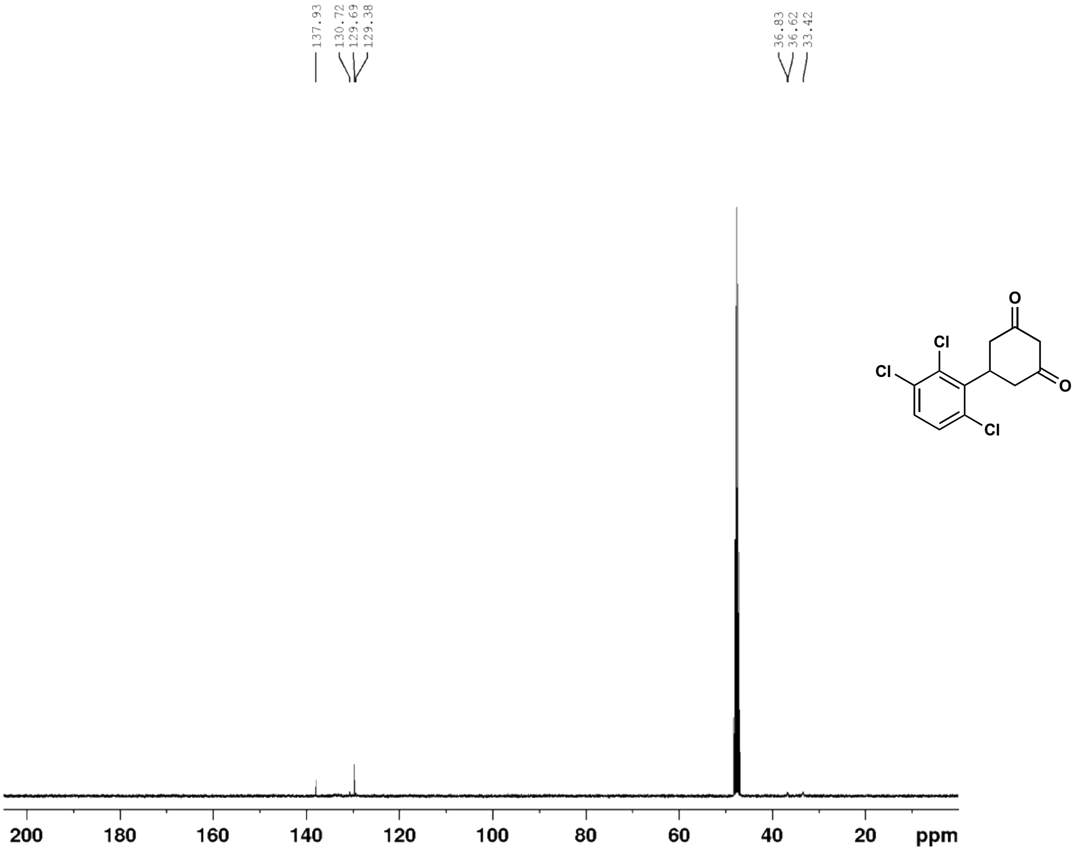

4-oxo-6-(2,3,6-trichlorophenyl)-4,5,6,7-tetrahydrobenzofuran-3-carboxylic acid (**23**) ^{1}H NMR (500 MHz, $CDCl_3$)

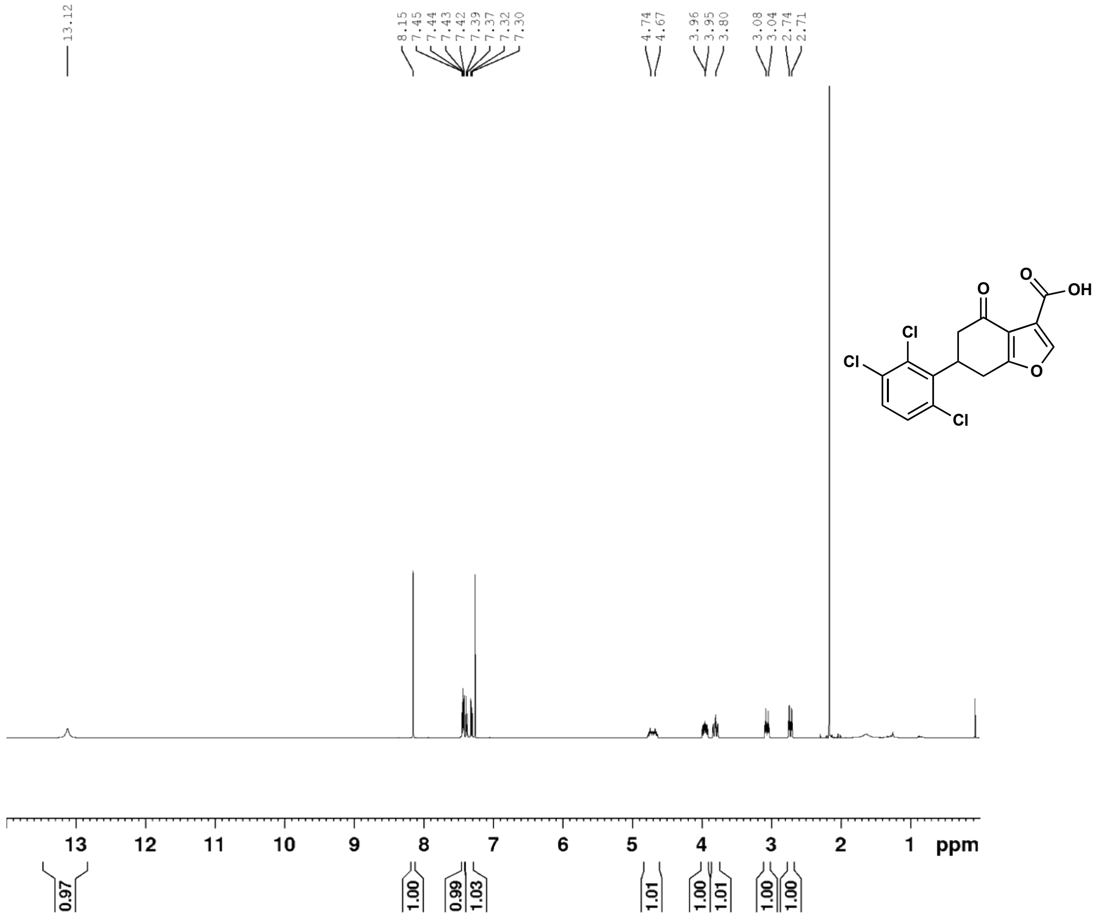

4-oxo-6-(2,3,6-trichlorophenyl)-4,5,6,7-tetrahydrobenzofuran-3-carboxylic acid (**23**) ^{13}C NMR (126 MHz, $CDCl_3$)

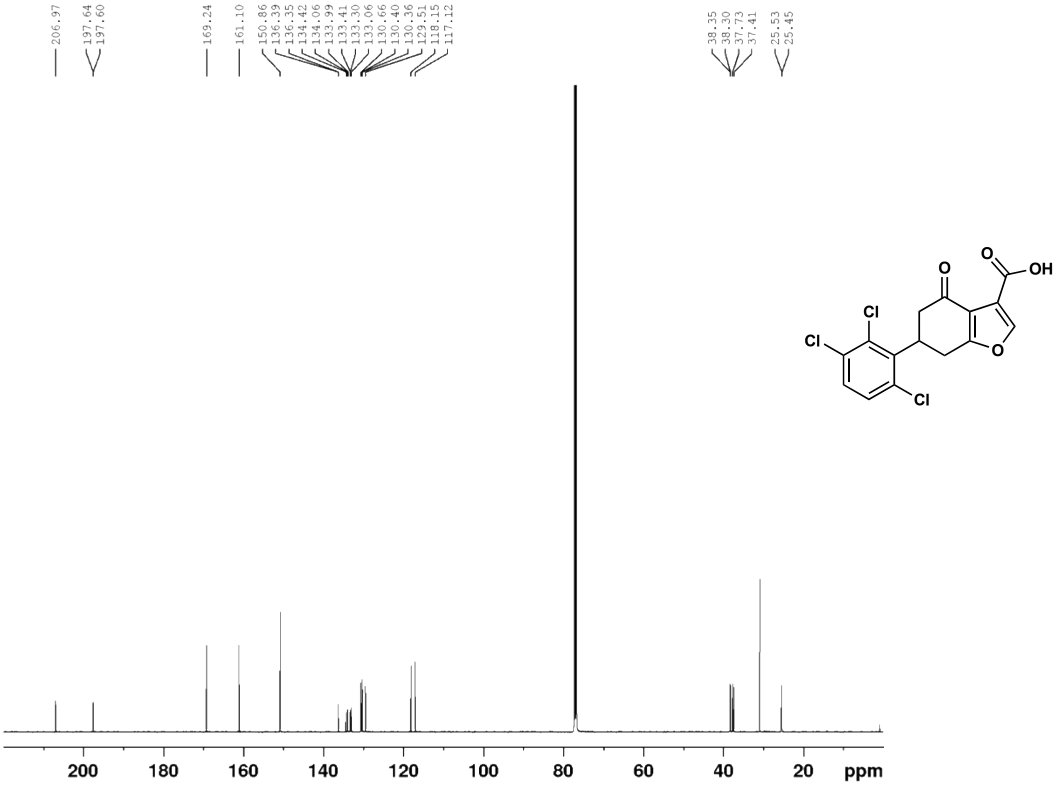

(E)-4-(4-chlorophenyl)but-3-en-2-one (**24b**) ^{1}H NMR (400 MHz, $CDCl_3$)

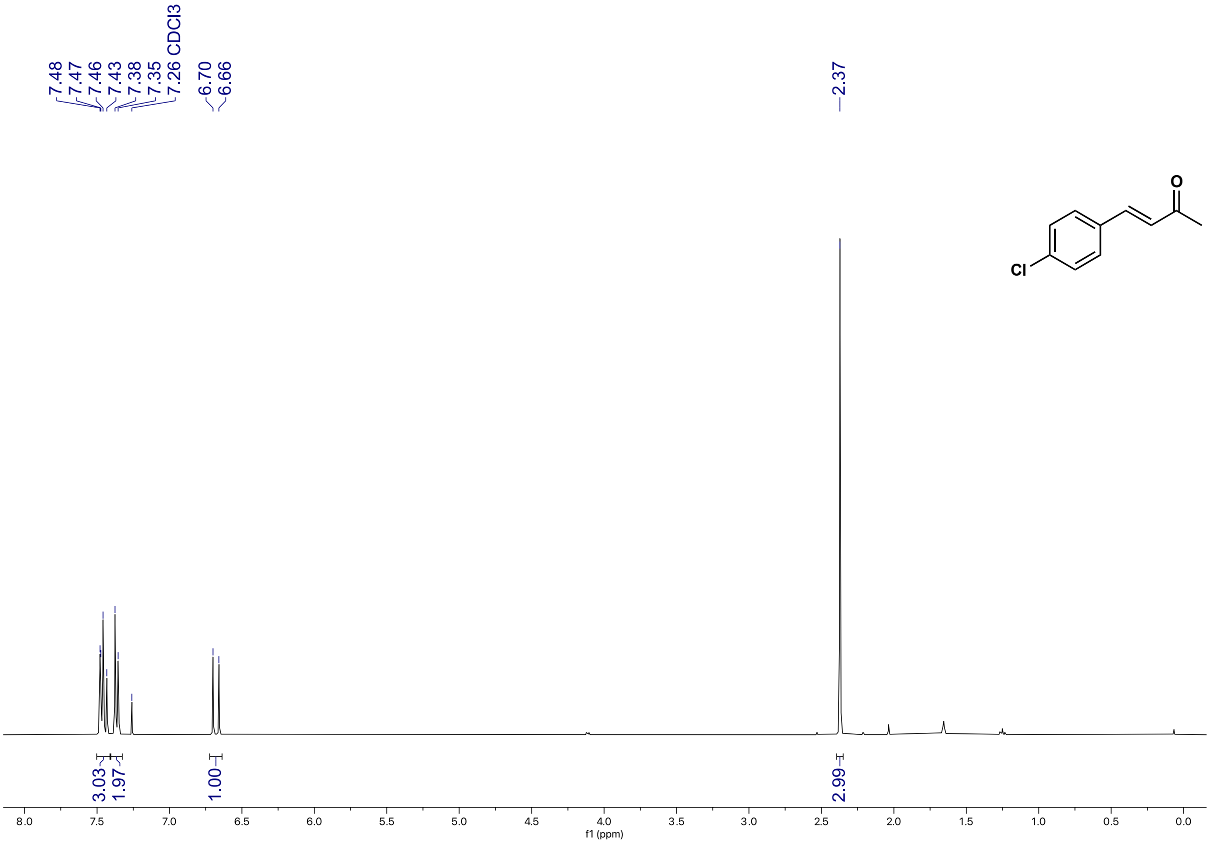

(E)-4-(4-chlorophenyl)but-3-en-2-one (**24b**) ^{13}C NMR (101 MHz, $CDCl_3$)

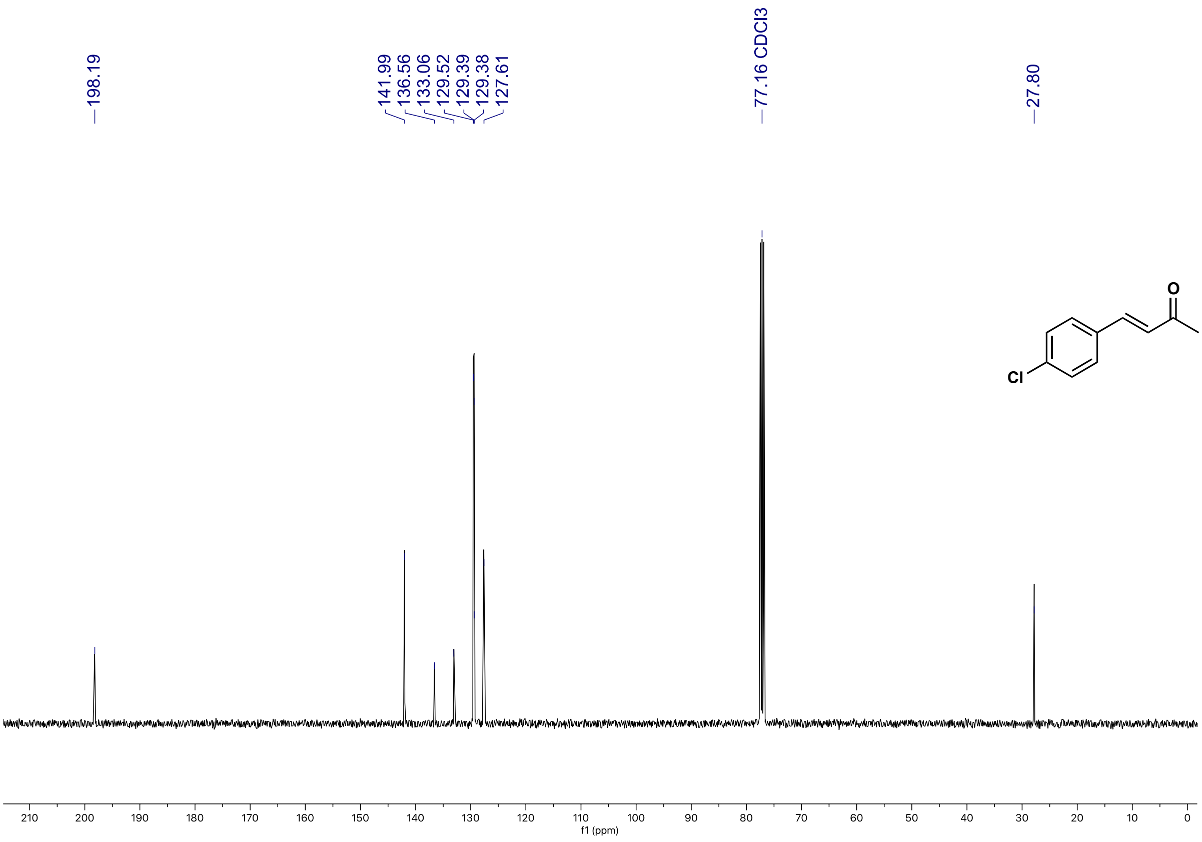

5-(4-chlorophenyl)cyclohexane-1,3-dione (**24c**) ^{1}H NMR (500 MHz, d_6-DMSO)

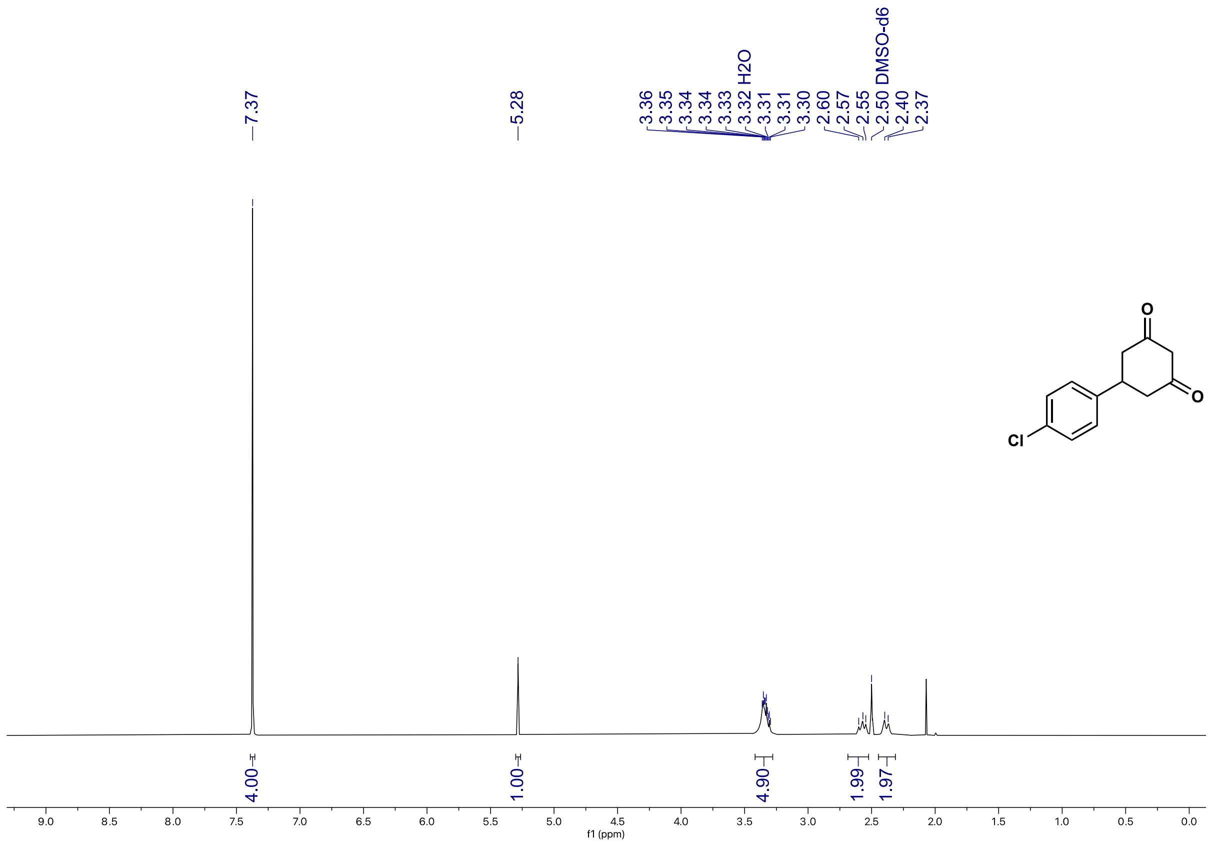

5-(4-chlorophenyl)cyclohexane-1,3-dione (**24c**) ^{13}C NMR (126 MHz, d_6-DMSO)

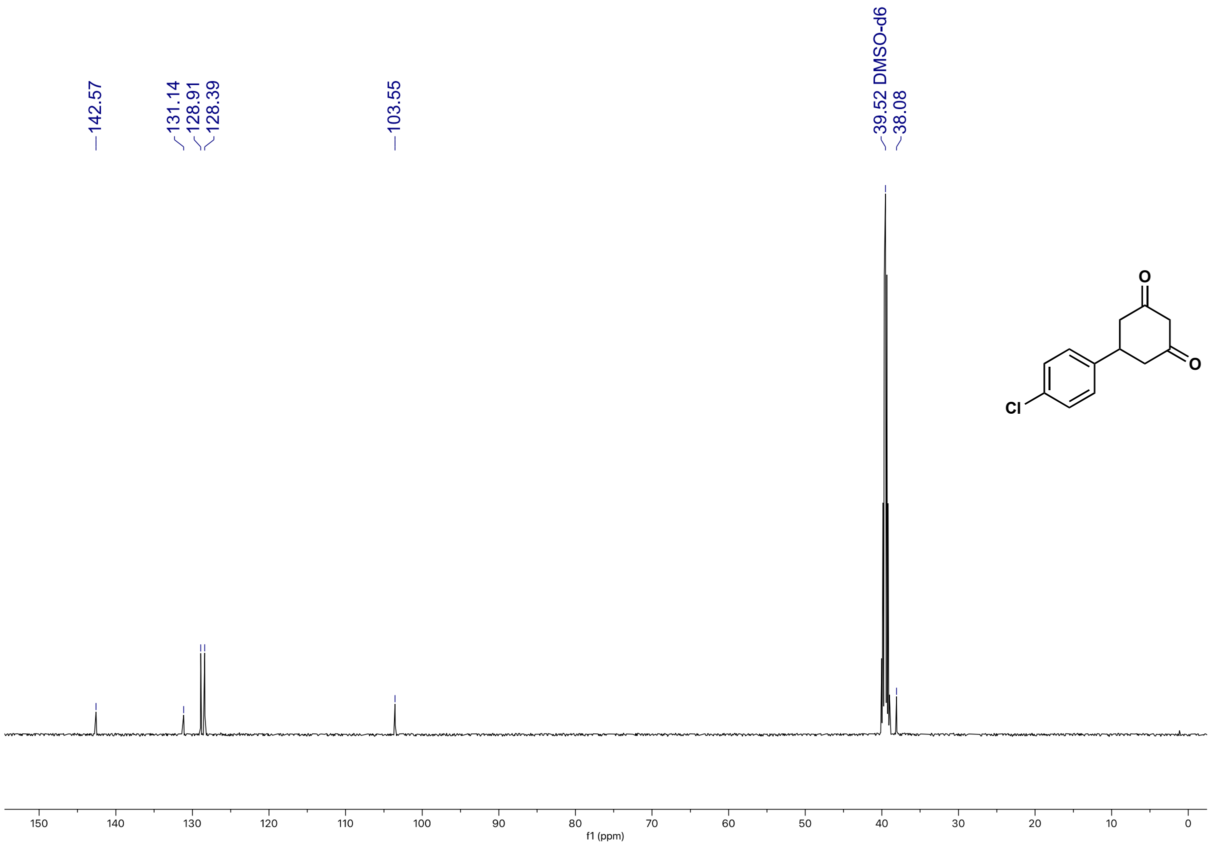

6-(4-chlorophenyl)-4-oxo-4,5,6,7-tetrahydrobenzofuran-3-carboxylic acid (**24**) ^{1}H NMR (500 MHz, $CDCl_3$)

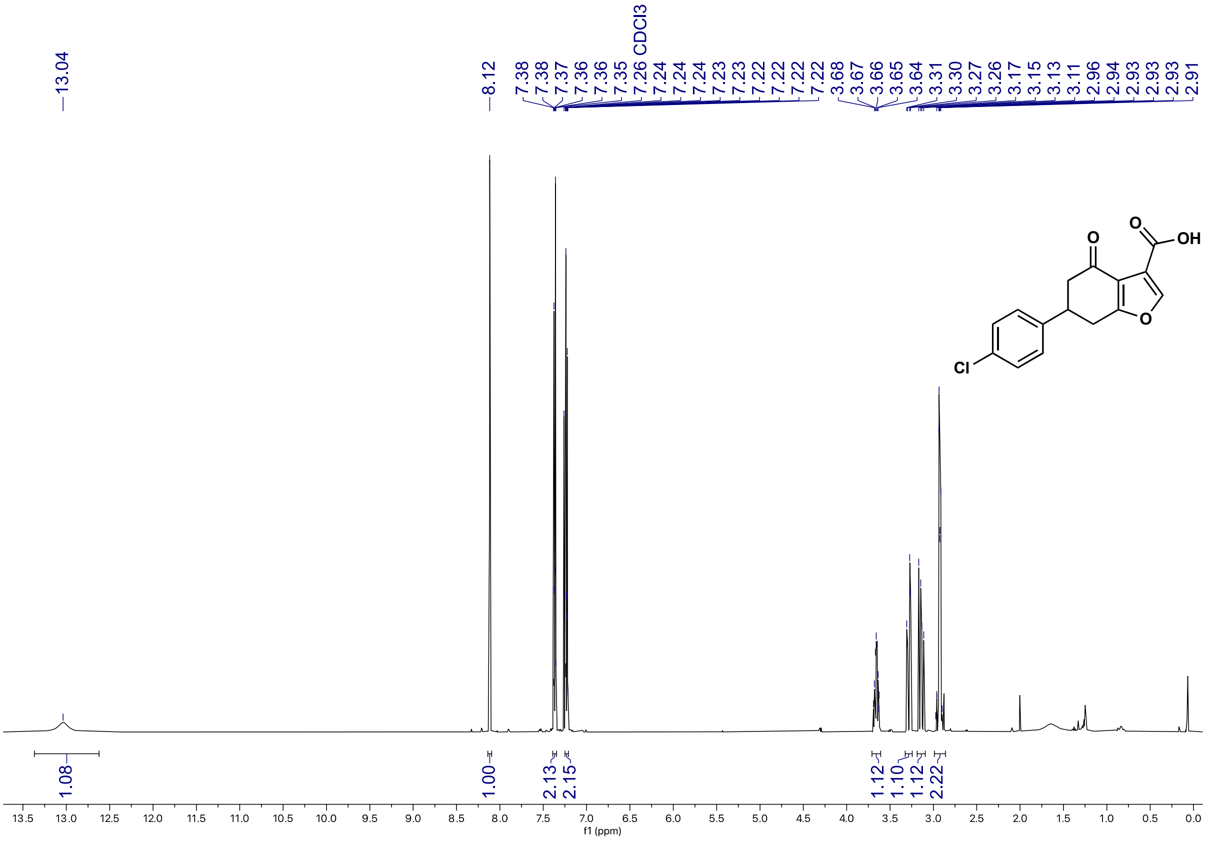

6-(4-chlorophenyl)-4-oxo-4,5,6,7-tetrahydrobenzofuran-3-carboxylic acid (**24**) ^{13}C NMR (126 MHz, $CDCl_3$)

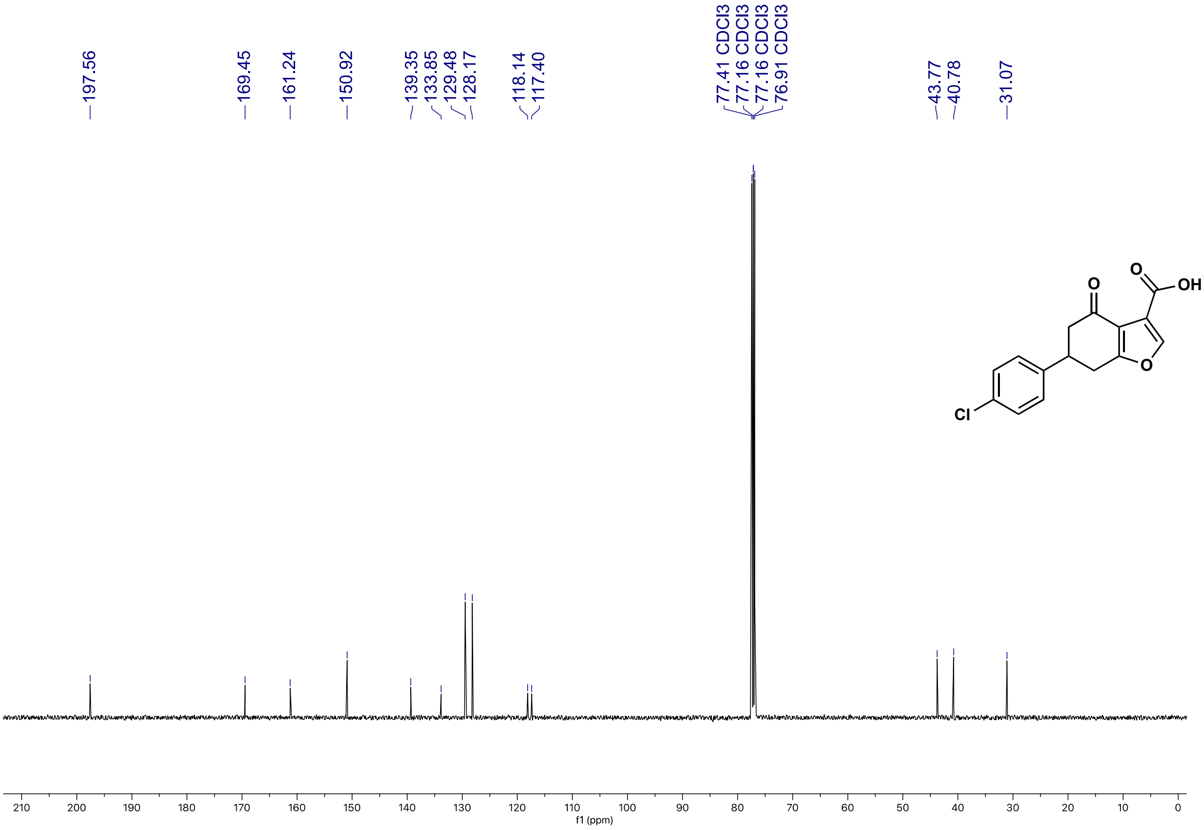

ethyl 6-(4-chlorophenyl)-4-oxo-4,5,6,7-tetrahydrobenzofuran-3-carboxylate (**24d**) ^{1}H NMR (700 MHz, $CDCl_3$)

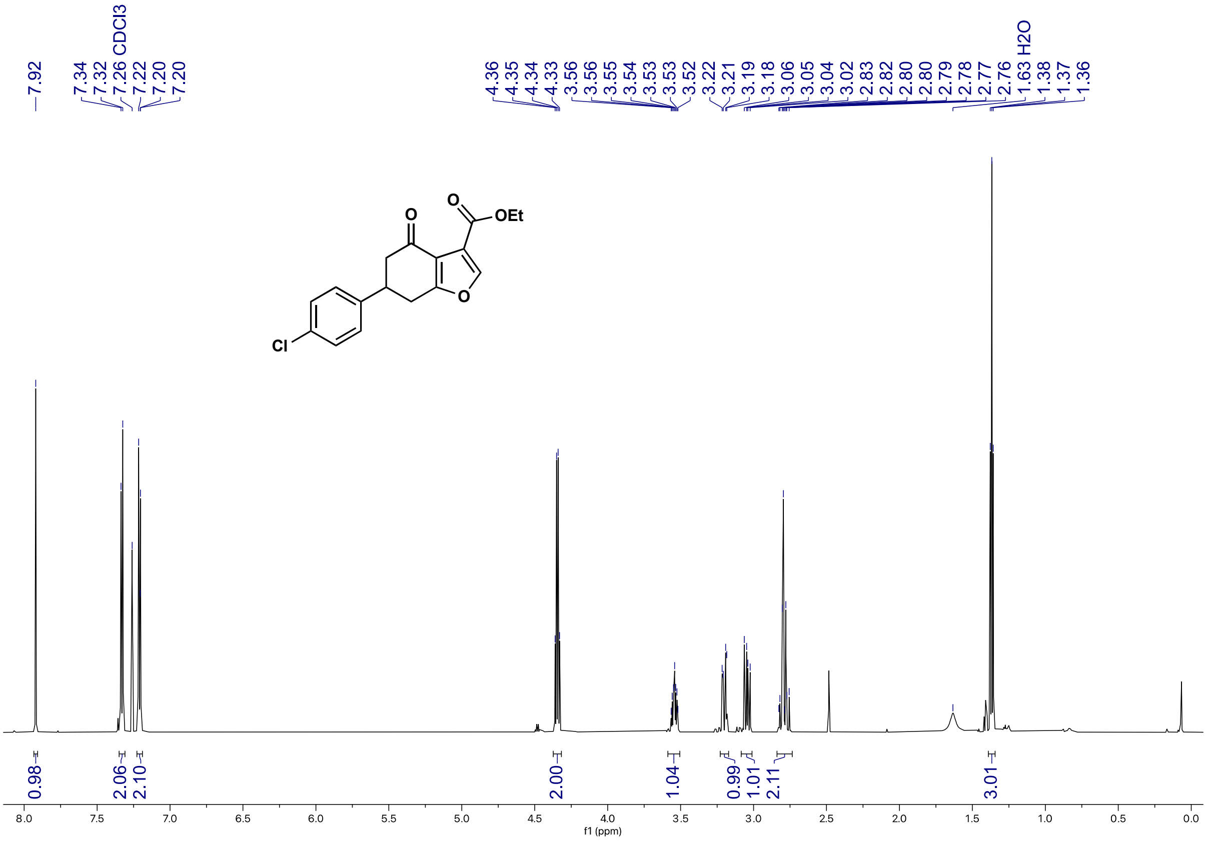

ethyl 6-(4-chlorophenyl)-4-oxo-4,5,6,7-tetrahydrobenzofuran-3-carboxylate (**24d**) ^{13}C NMR (176 MHz, $CDCl_3$)

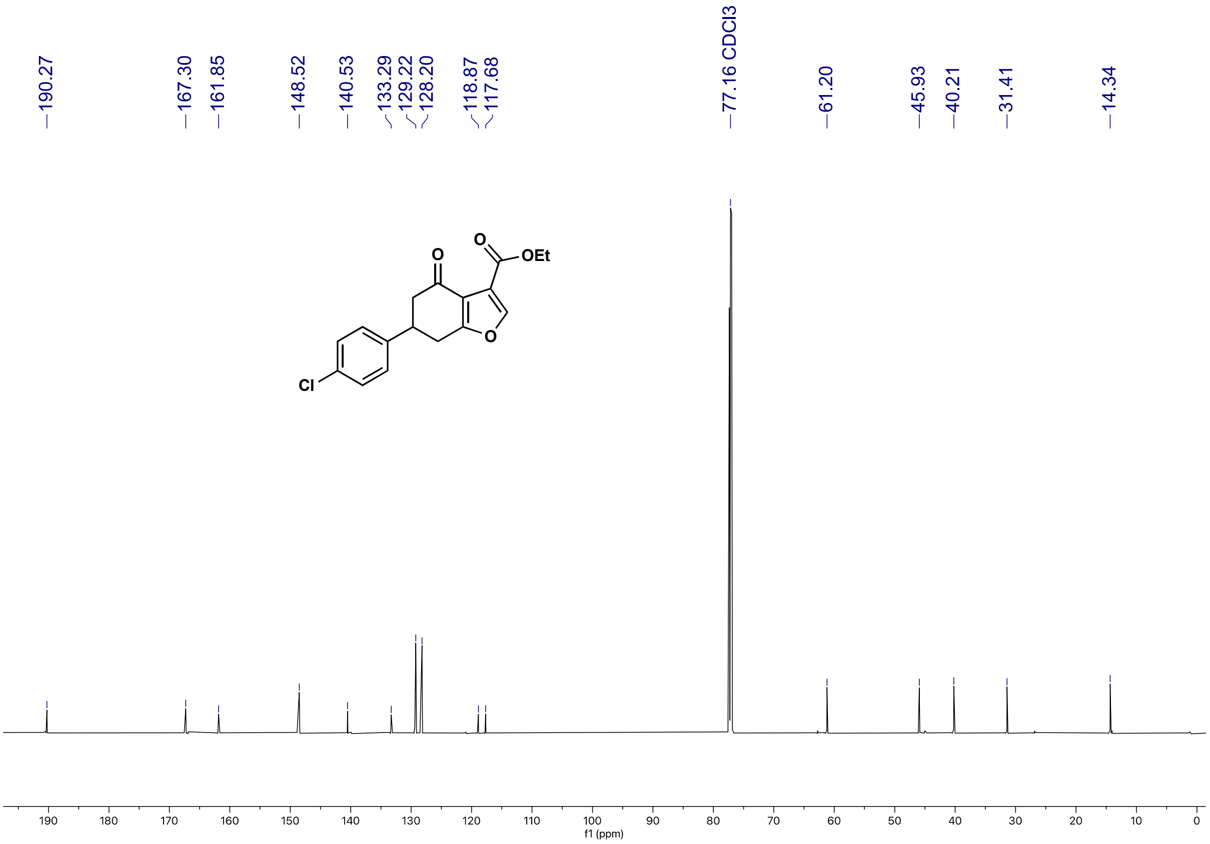

(E)-4-(2-chloro-3-methoxyphenyl)but-3-en-2-one (**25b**) ^1H NMR (500 MHz, $CDCl_3$)

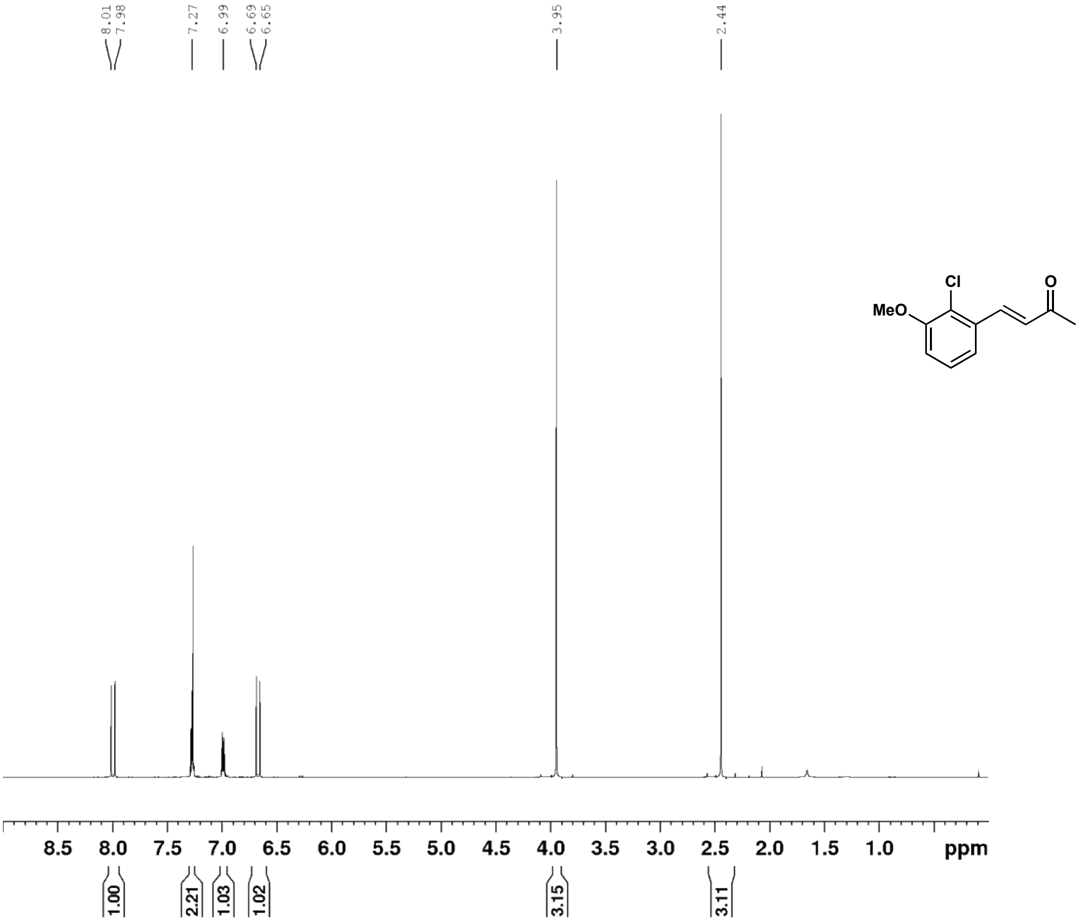

(E)-4-(2-chloro-3-methoxyphenyl)but-3-en-2-one (**25b**) ^{13}C NMR (126 MHz, $CDCl_3$)

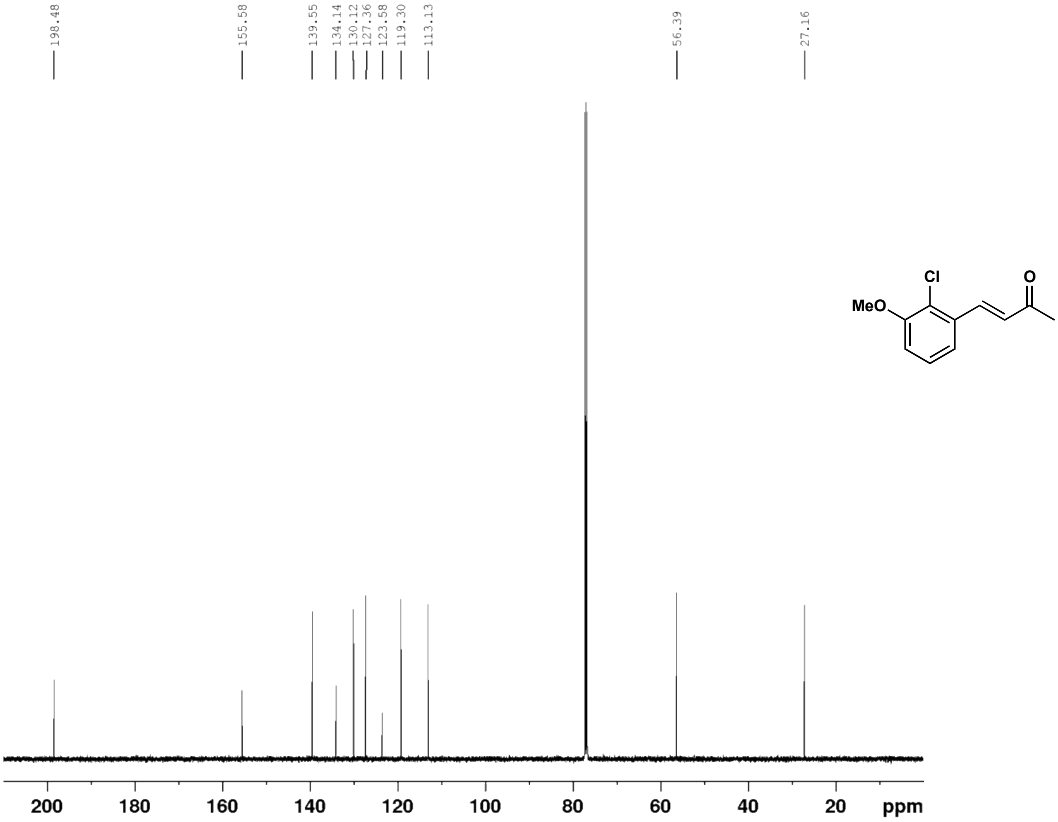

5-(2-chloro-3-methoxyphenyl)cyclohexane-1,3-dione (**25c**) ^{1}H NMR (500 MHz, CD_3OD)

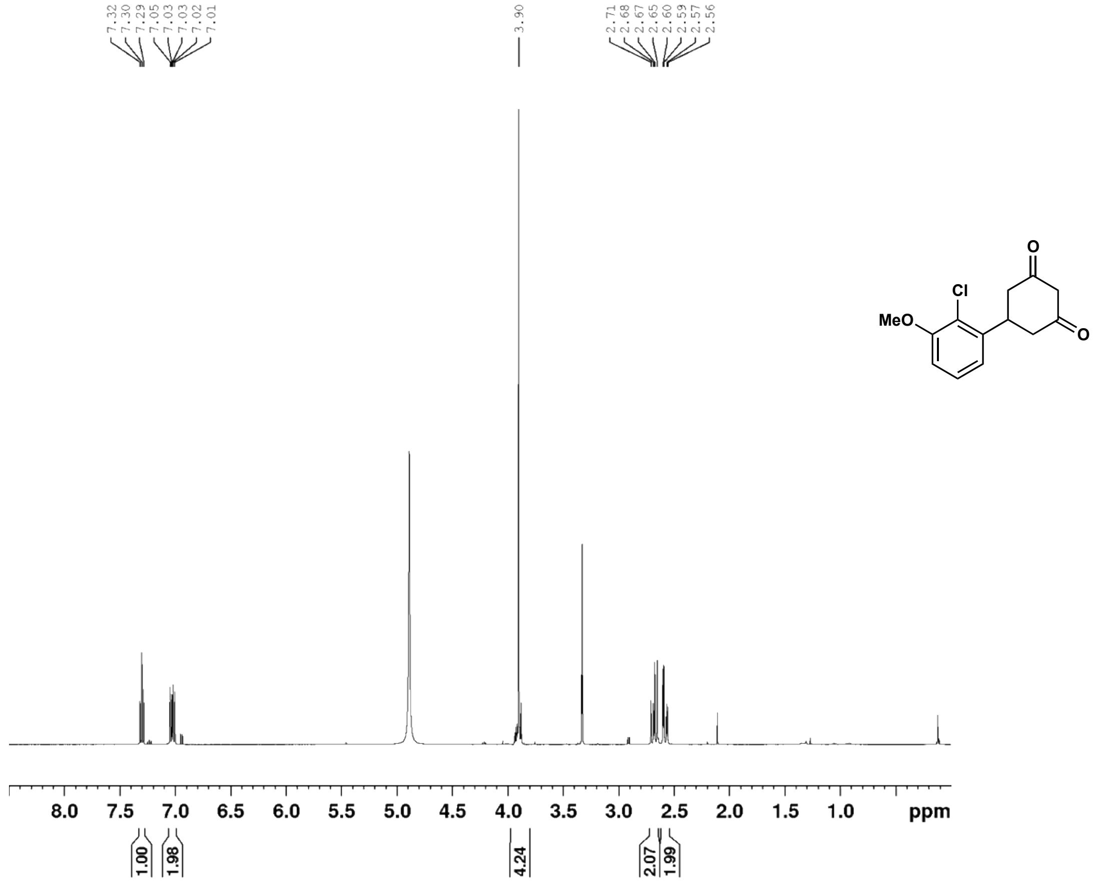

5-(2-chloro-3-methoxyphenyl)cyclohexane-1,3-dione (**25c**) ^{13}C NMR (126 MHz, CD_3OD)

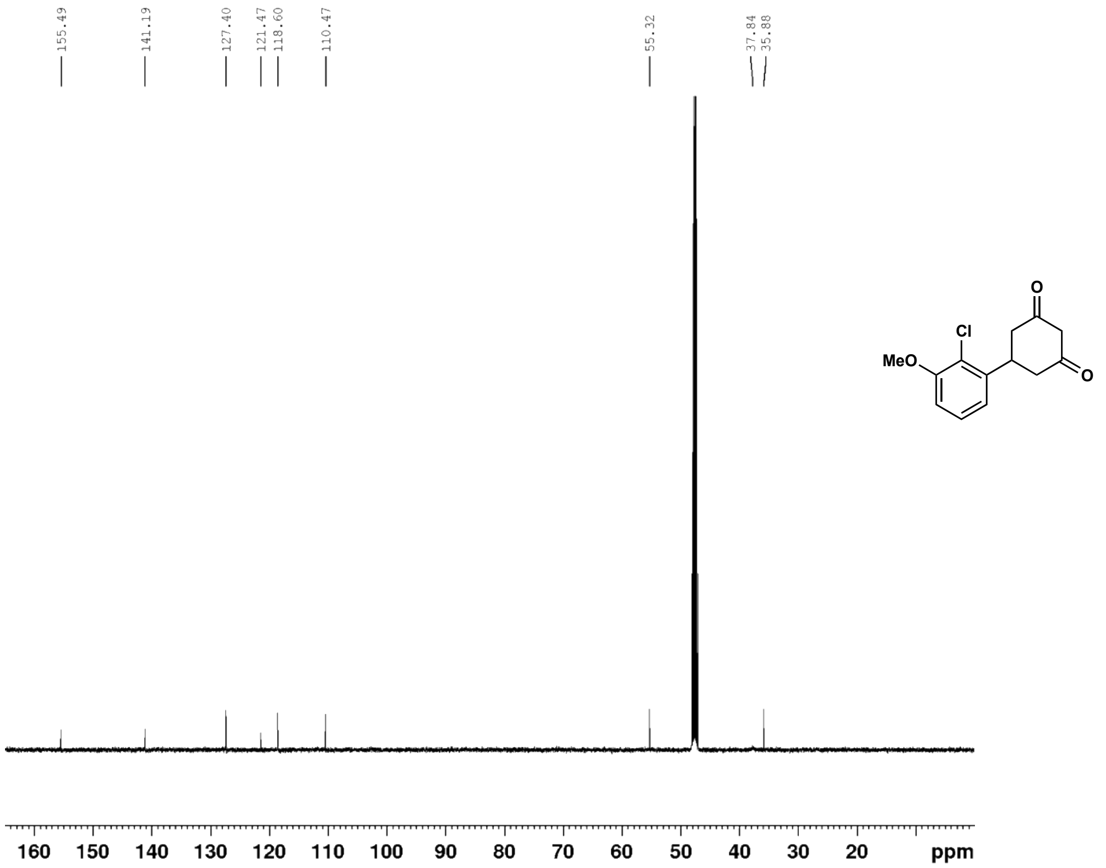

6-(2-chloro-3-methoxyphenyl)-4-oxo-4,5,6,7-tetrahydrobenzofuran-3-carboxylic acid (**25**) ^{1}H NMR (500 MHz, $CDCl_3$)

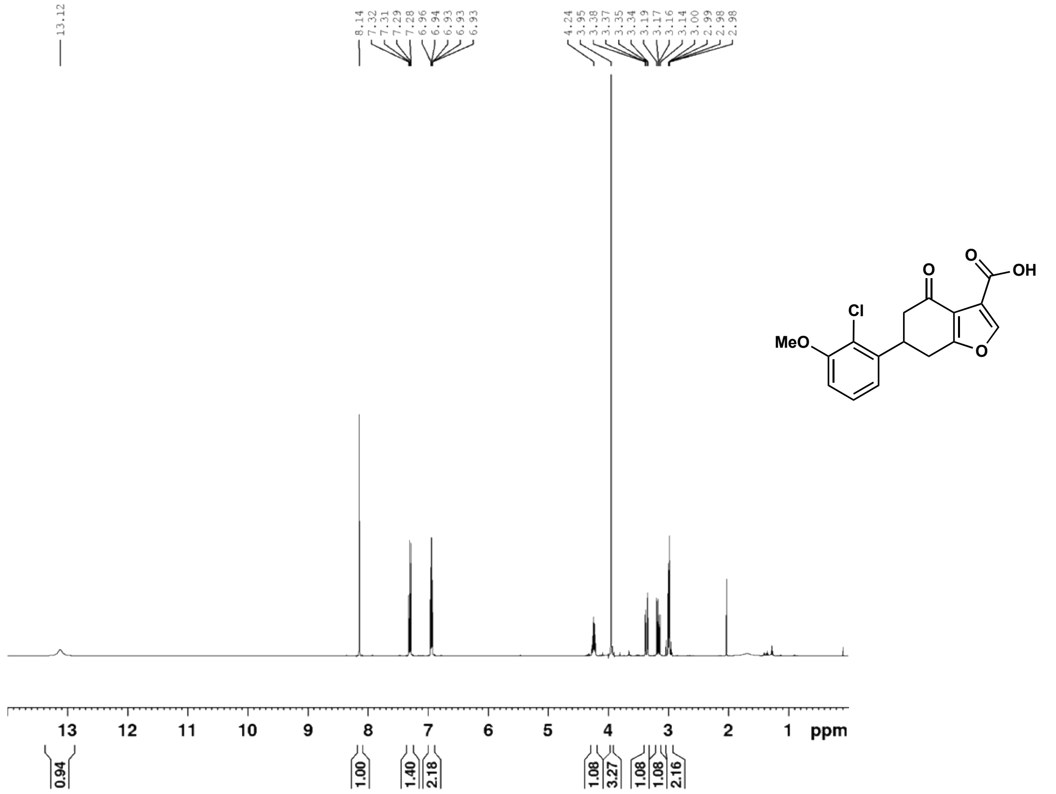

6-(2-chloro-3-methoxyphenyl)-4-oxo-4,5,6,7-tetrahydrobenzofuran-3-carboxylic acid (**25**) ^{13}C NMR (126 MHz, $CDCl_3$)

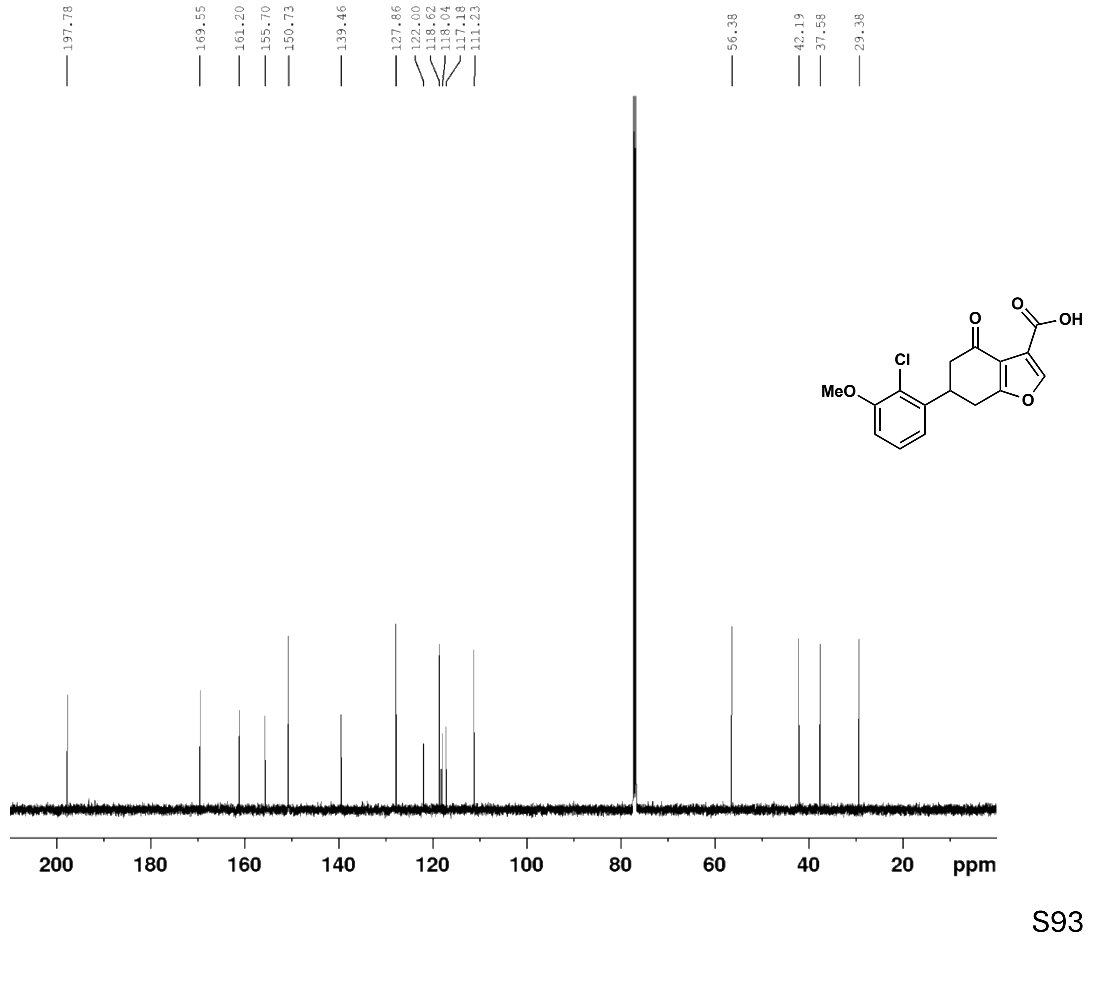

(E)-4-(2,5-dimethoxyphenyl)but-3-en-2-one (**26b**) ^{1}H NMR (500 MHz, $CDCl_3$)

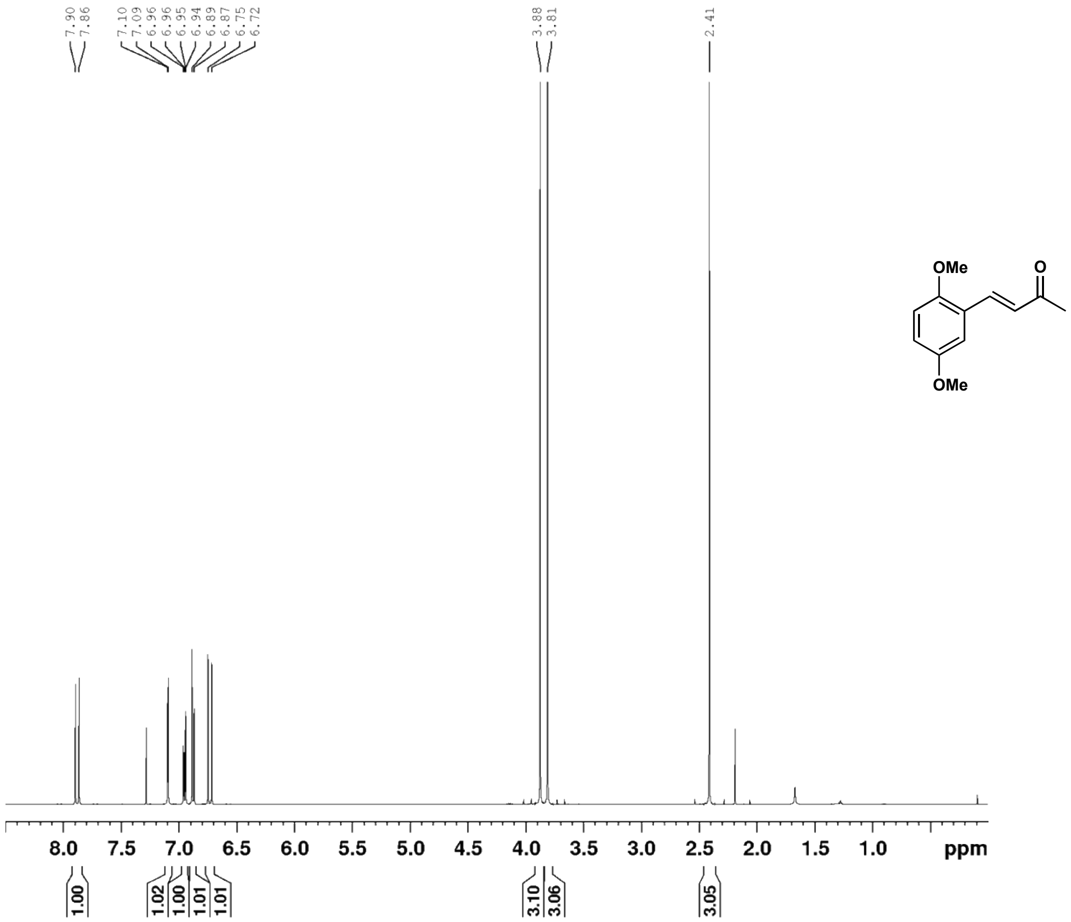

(E)-4-(2,5-dimethoxyphenyl)but-3-en-2-one (**26b**) ^{13}C NMR (126 MHz, $CDCl_3$)

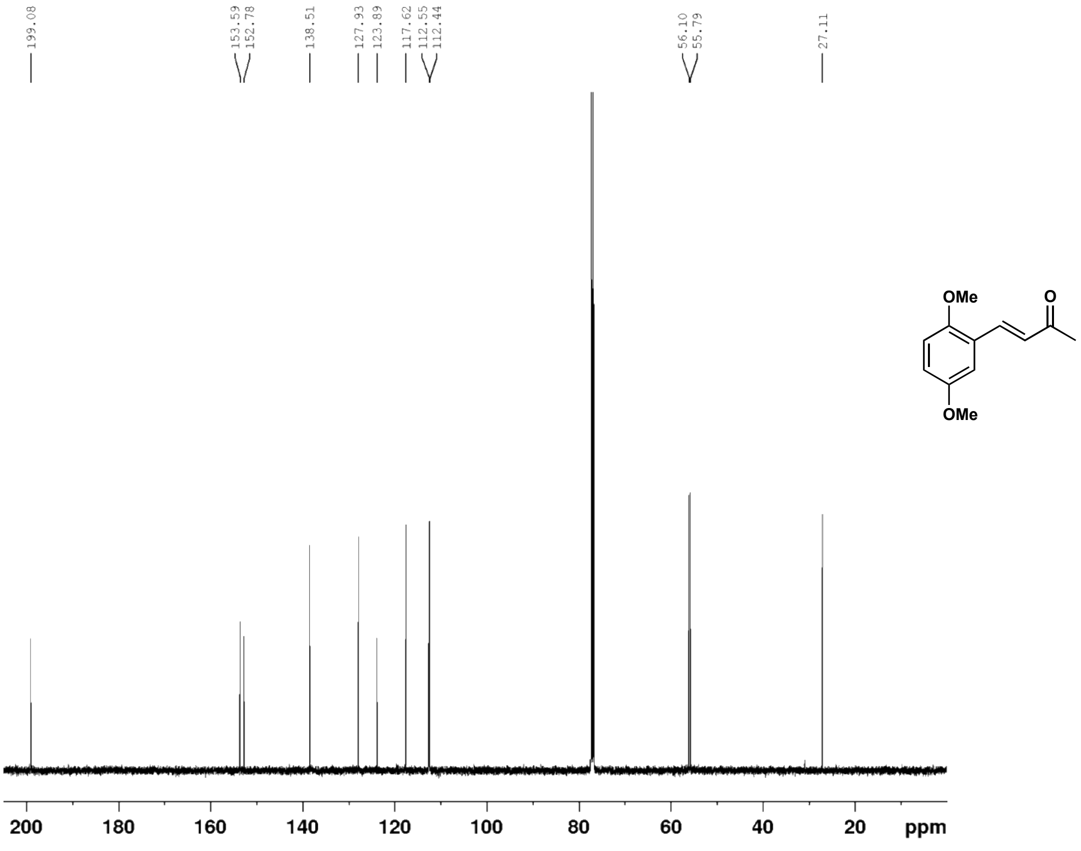

5-(2,5-dimethoxyphenyl)cyclohexane-1,3-dione (**26c**) ^{1}H NMR (500 MHz, CD_3OD)

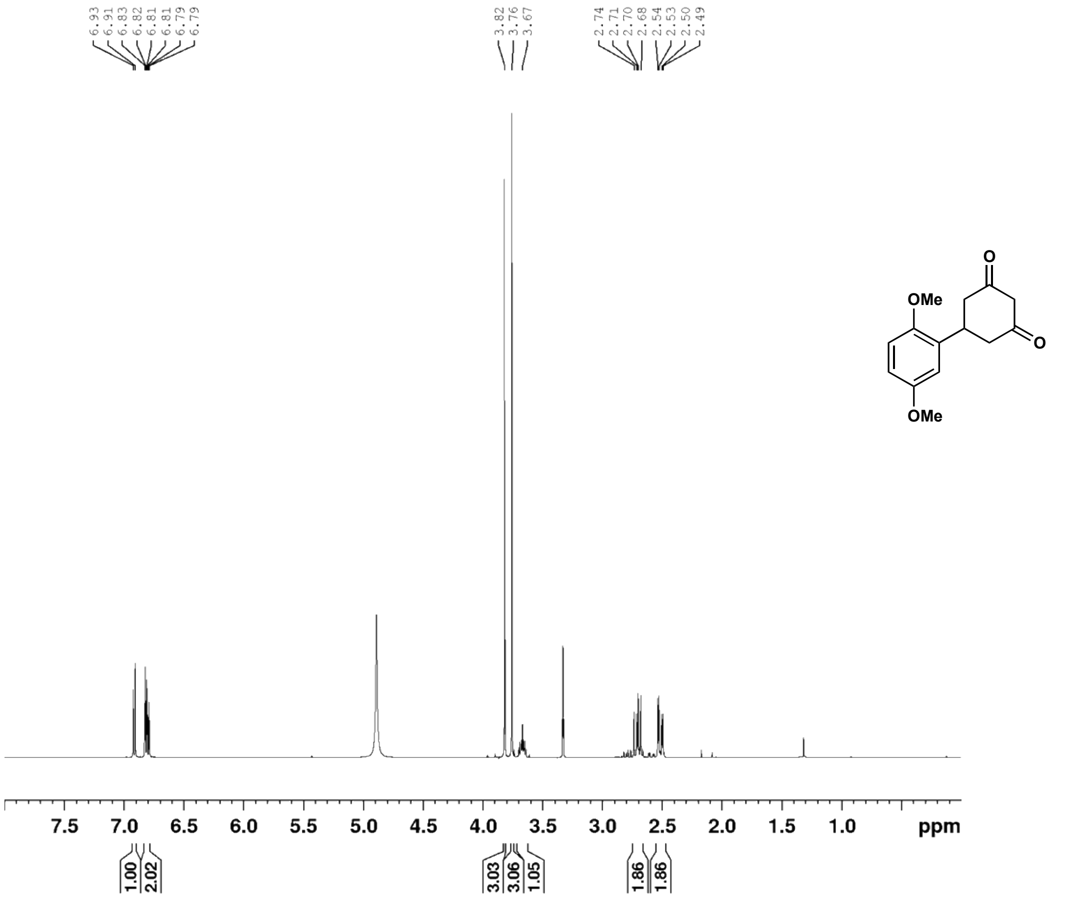

5-(2,5-dimethoxyphenyl)cyclohexane-1,3-dione (**26c**) ^{13}C NMR (126 MHz, CD_3OD)

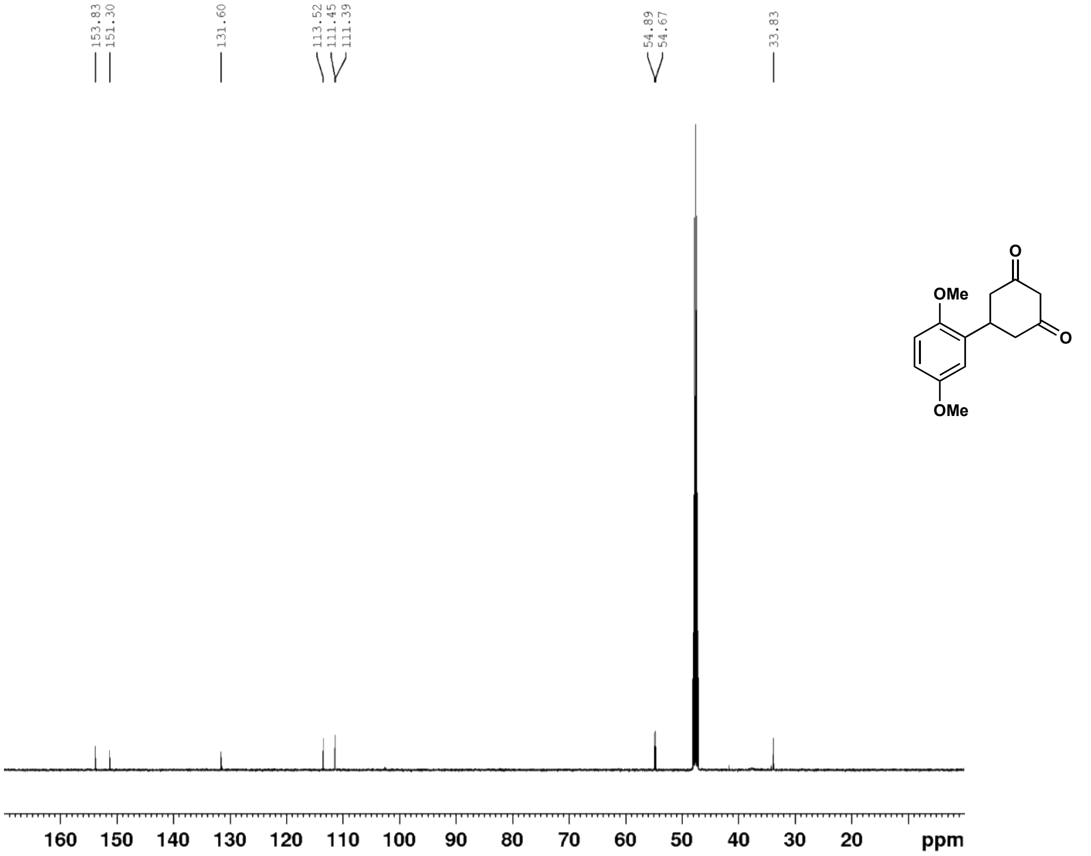

6-(2,5-dimethoxyphenyl)-4-oxo-4,5,6,7-tetrahydrobenzofuran-3-carboxylic acid (**26**) ^{1}H NMR (500 MHz, d_6-DMSO)

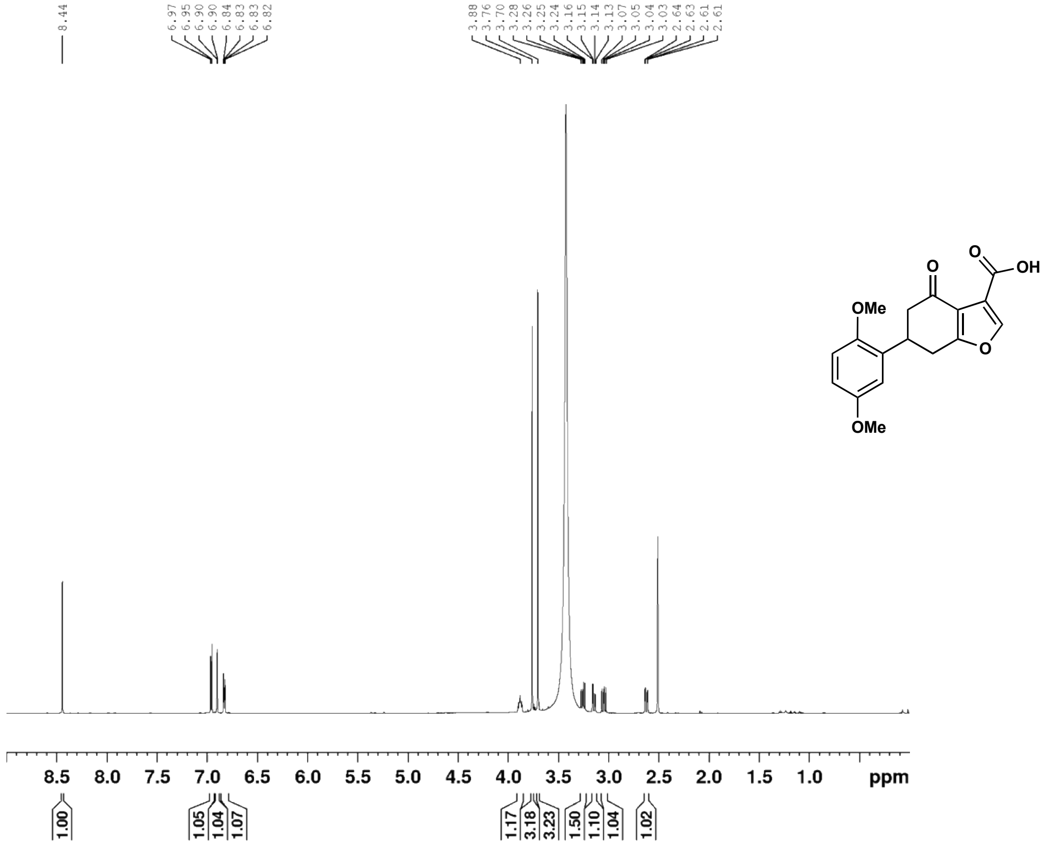

6-(2,5-dimethoxyphenyl)-4-oxo-4,5,6,7-tetrahydrobenzofuran-3-carboxylic acid (**26**) ^{13}C NMR (126 MHz, d_6-DMSO)

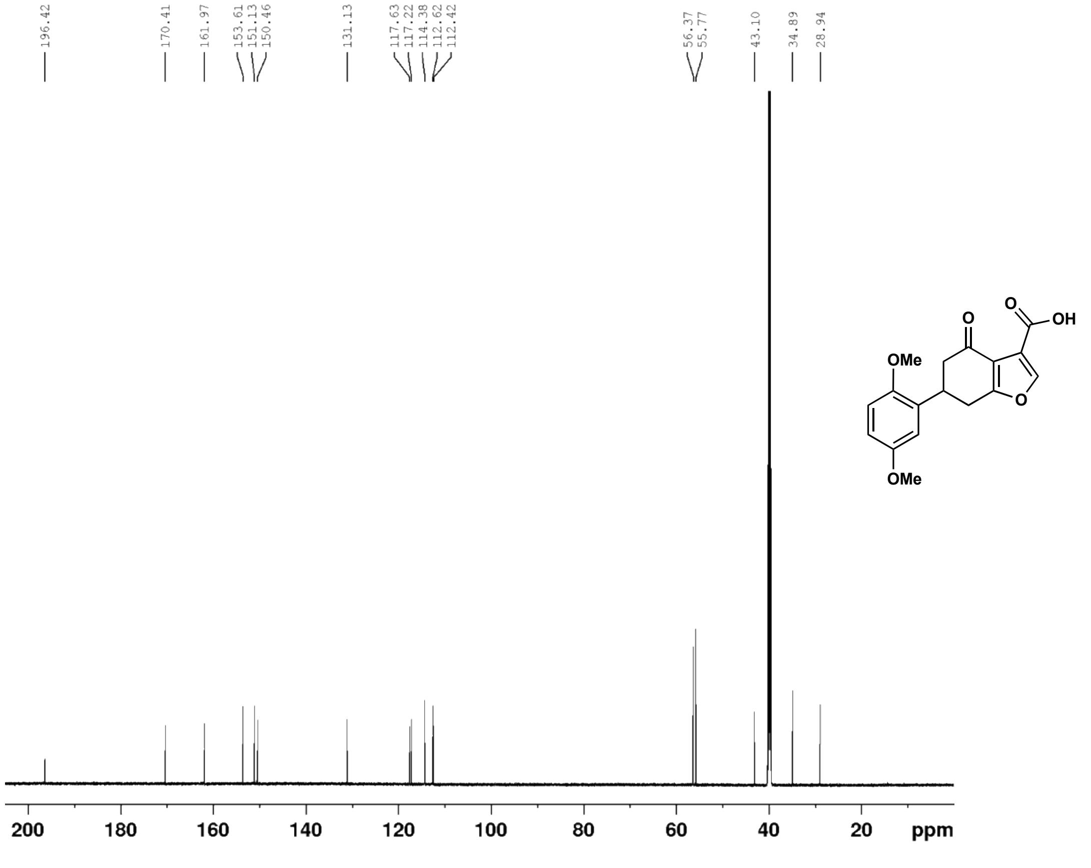

(E)-4-(4-chloro-2-methoxyphenyl)but-3-en-2-one (**27b**) ^{1}H NMR (500 MHz, $CDCl_3$)

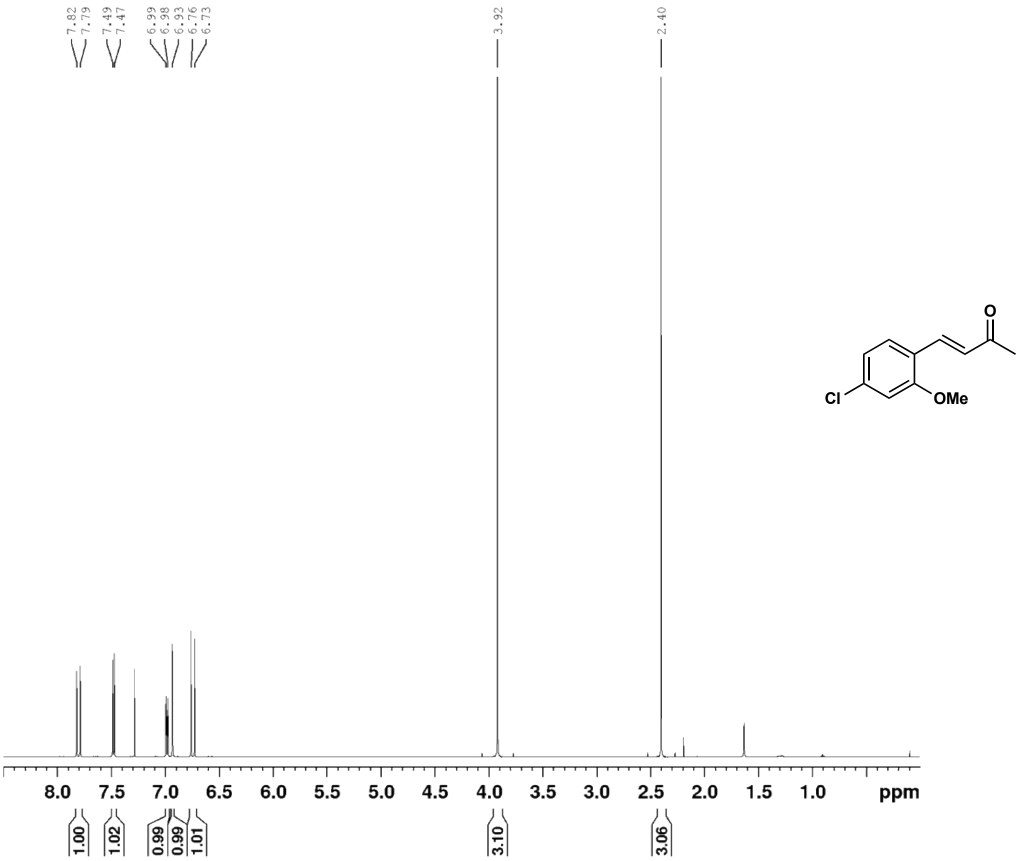

(E)-4-(4-chloro-2-methoxyphenyl)but-3-en-2-one (**27b**) ^{13}C NMR (126 MHz, $CDCl_3$)

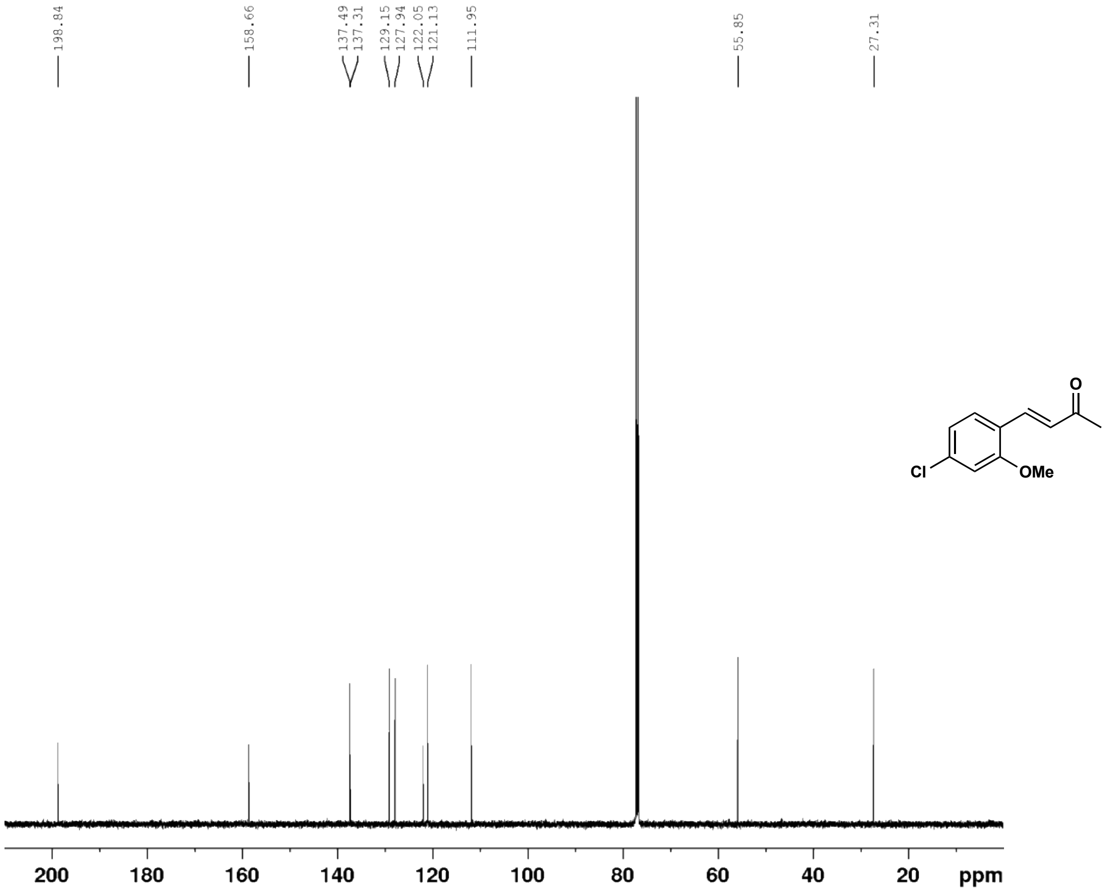

5-(4-chloro-2-methoxyphenyl)cyclohexane-1,3-dione (**27c**) ^{1}H NMR (500 MHz, d_6-DMSO)

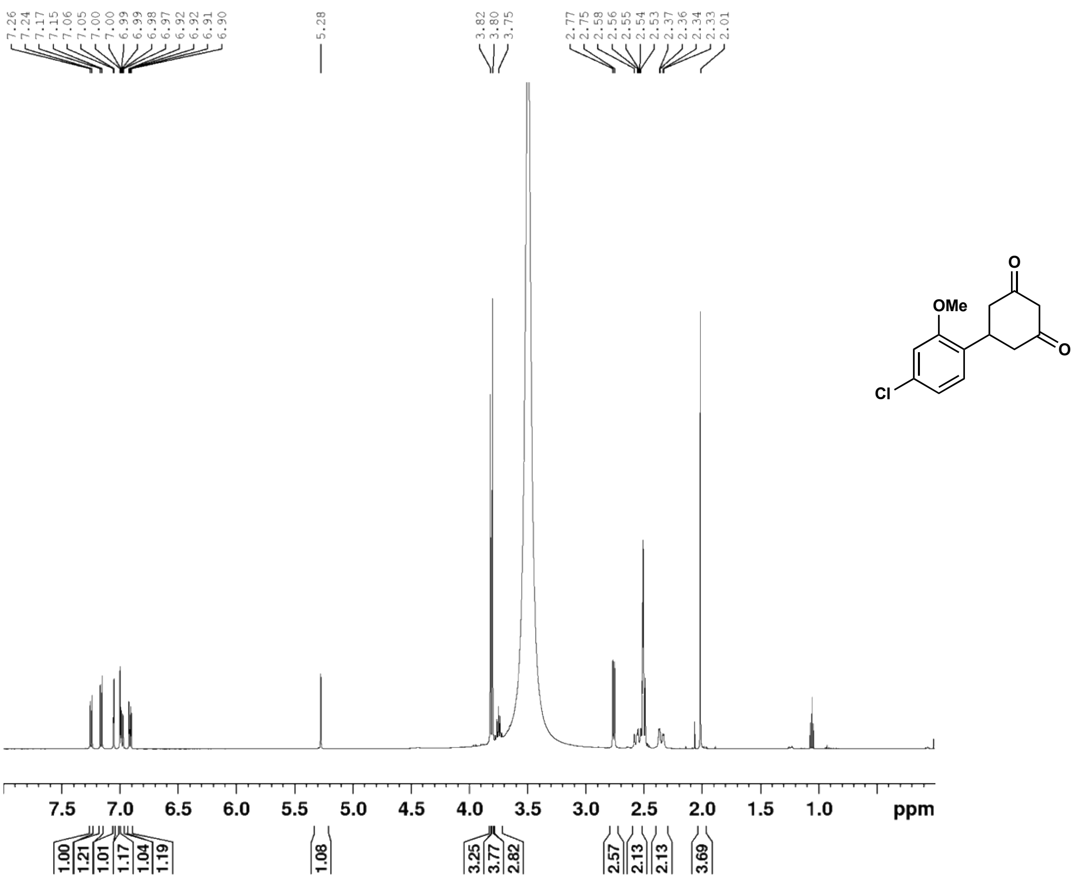

5-(4-chloro-2-methoxyphenyl)cyclohexane-1,3-dione (**27c**) ^{13}C NMR (126 MHz, d_6-DMSO)

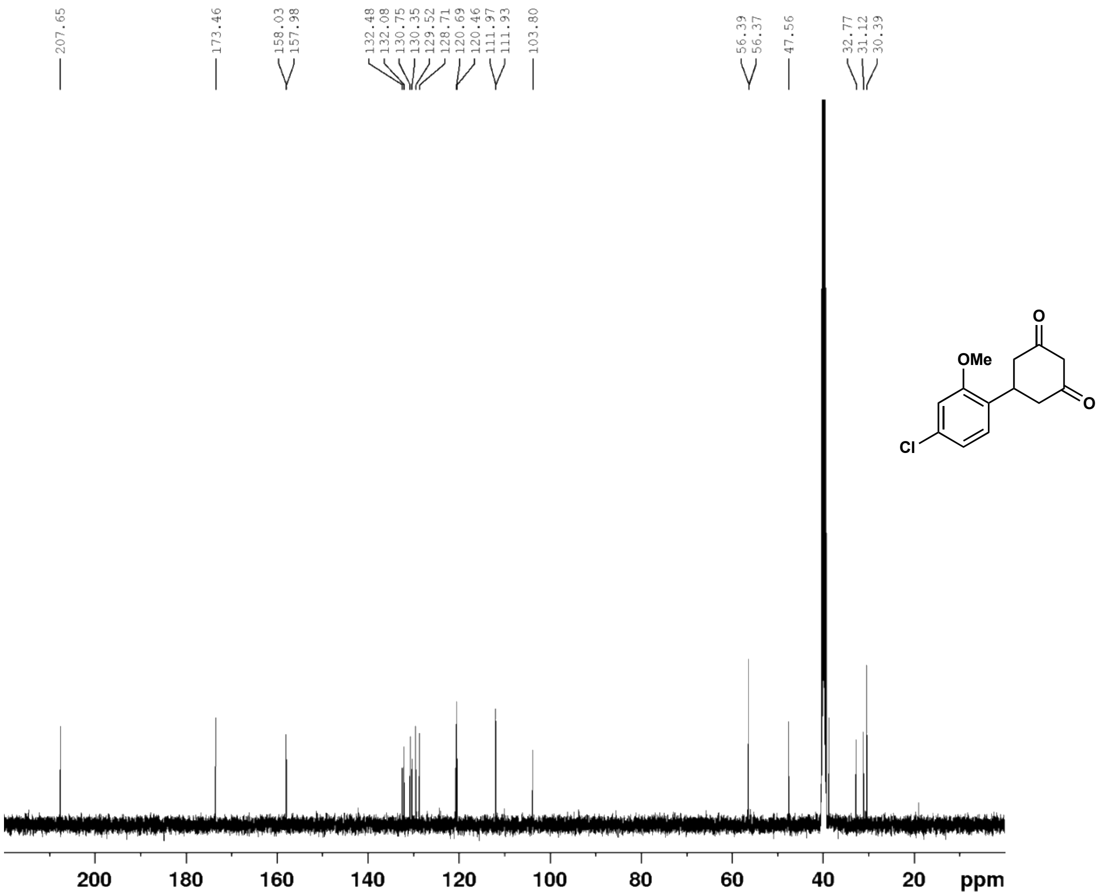

6-(4-chloro-2-methoxyphenyl)-4-oxo-4,5,6,7-tetrahydrobenzofuran-3-carboxylic acid (**27**) ^{1}H NMR (500 MHz, $CDCl_3$)

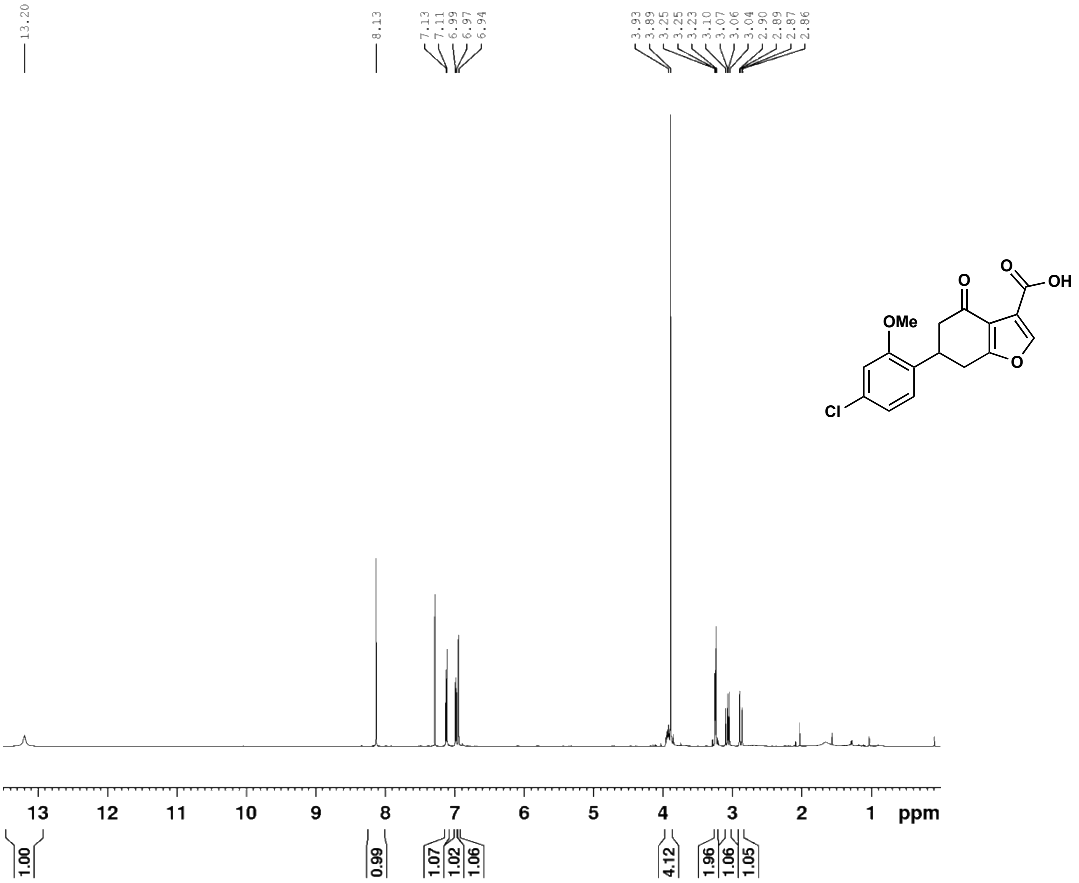

6-(4-chloro-2-methoxyphenyl)-4-oxo-4,5,6,7-tetrahydrobenzofuran-3-carboxylic acid (**27**) ^{13}C NMR (126 MHz, $CDCl_3$)

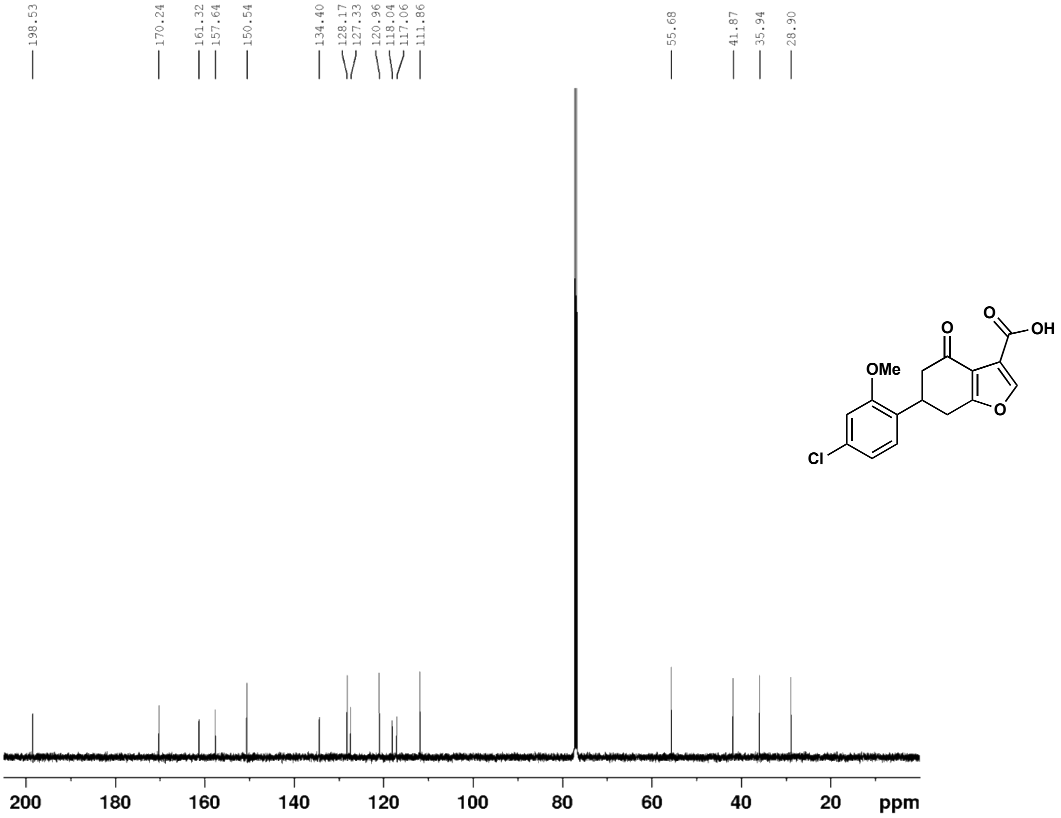

(E)-4-(4-chloro-3-methoxyphenyl)but-3-en-2-one (**28b**) ^{1}H NMR (500 MHz, $CDCl_3$)

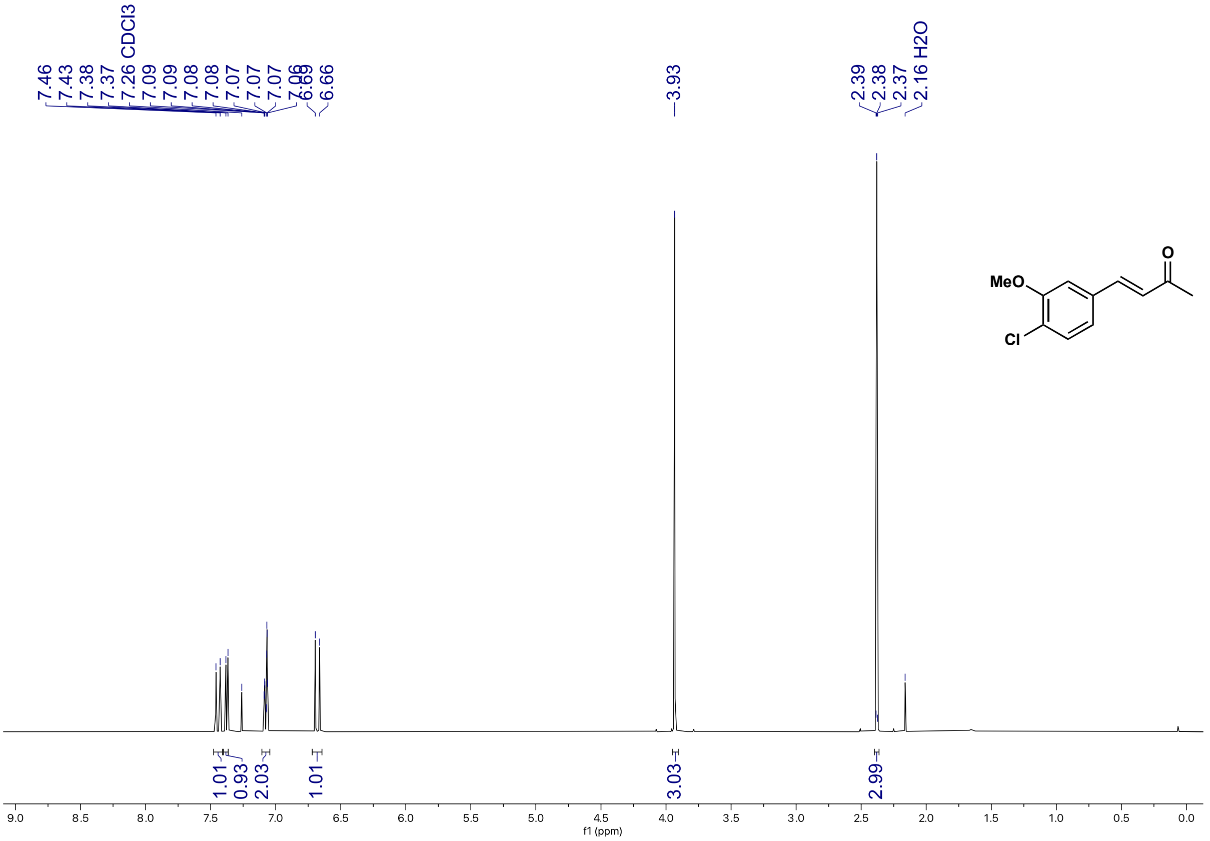

(E)-4-(4-chloro-3-methoxyphenyl)but-3-en-2-one (**28b**) ^{13}C NMR (126 MHz, $CDCl_3$)

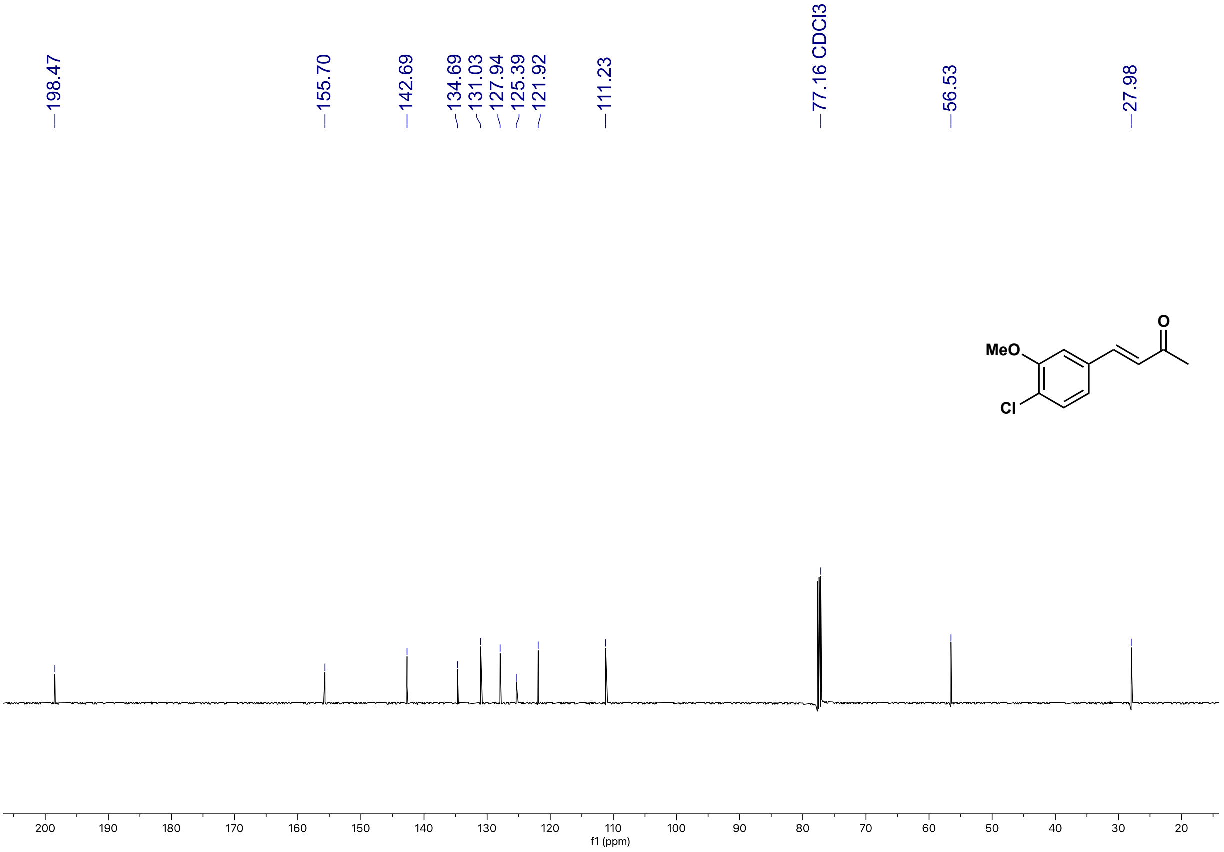

5-(4-chloro-3-methoxyphenyl)cyclohexane-1,3-dione (**28c**) ^{1}H NMR (500 MHz, CD_3OD)

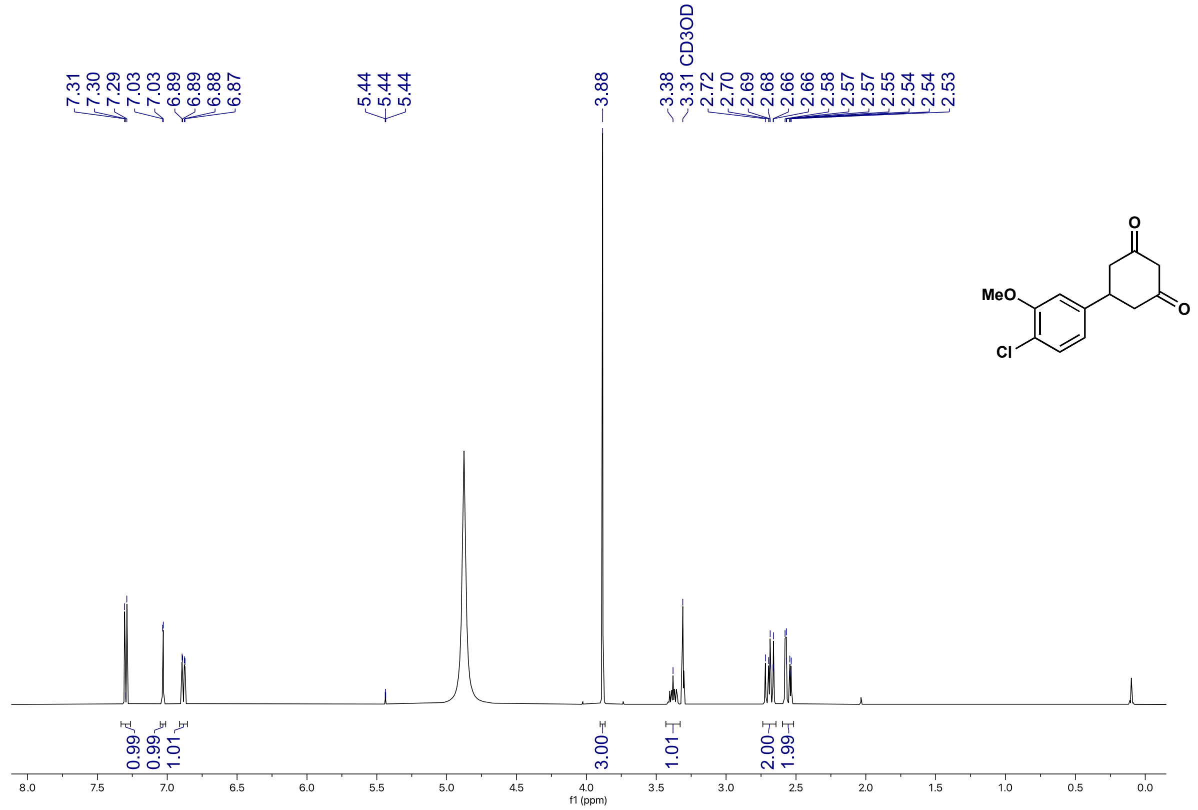

5-(4-chloro-3-methoxyphenyl)cyclohexane-1,3-dione (**28c**) ^{13}C NMR (126 MHz, CD_3OD)

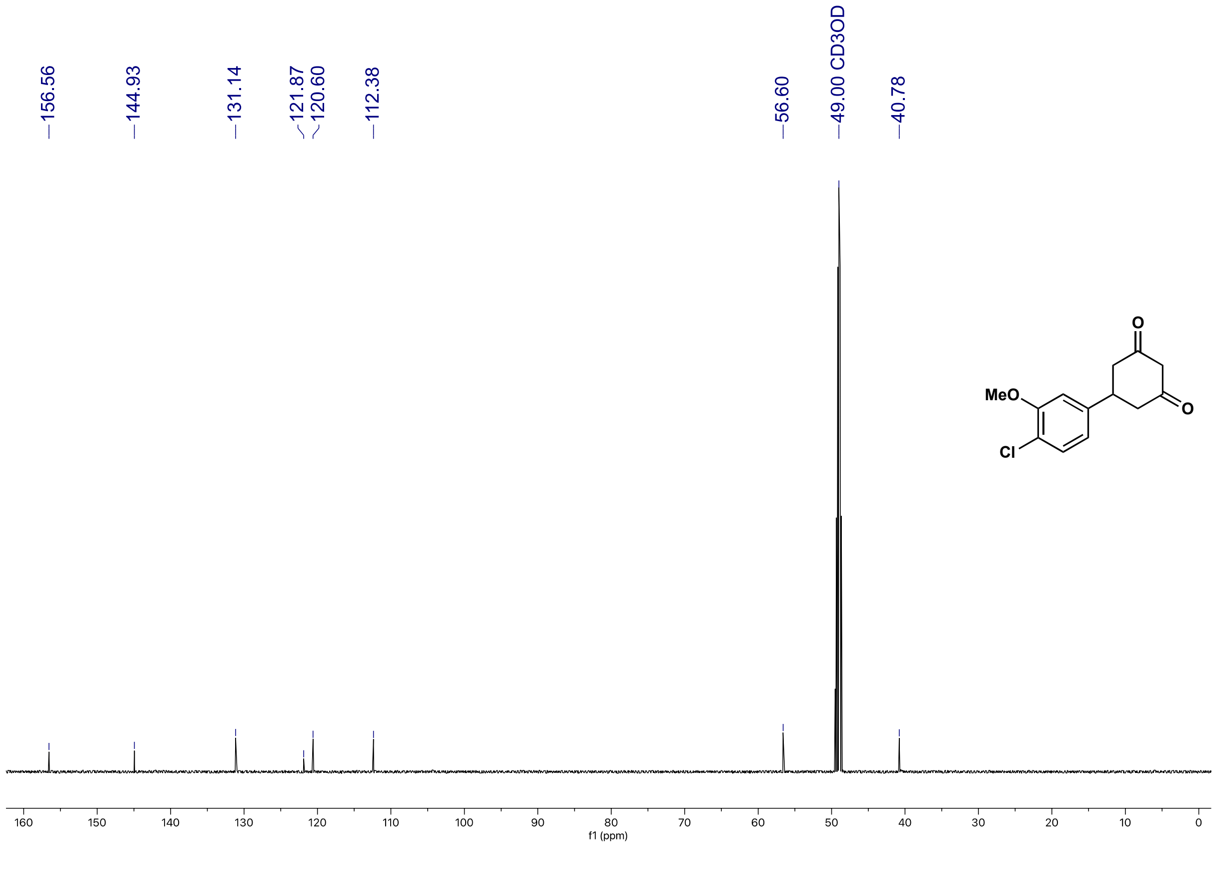

6-(4-chloro-3-methoxyphenyl)-4-oxo-4,5,6,7-tetrahydrobenzofuran-3-carboxylic acid (**28**) ^{1}H NMR (500 MHz, d_6-DMSO)

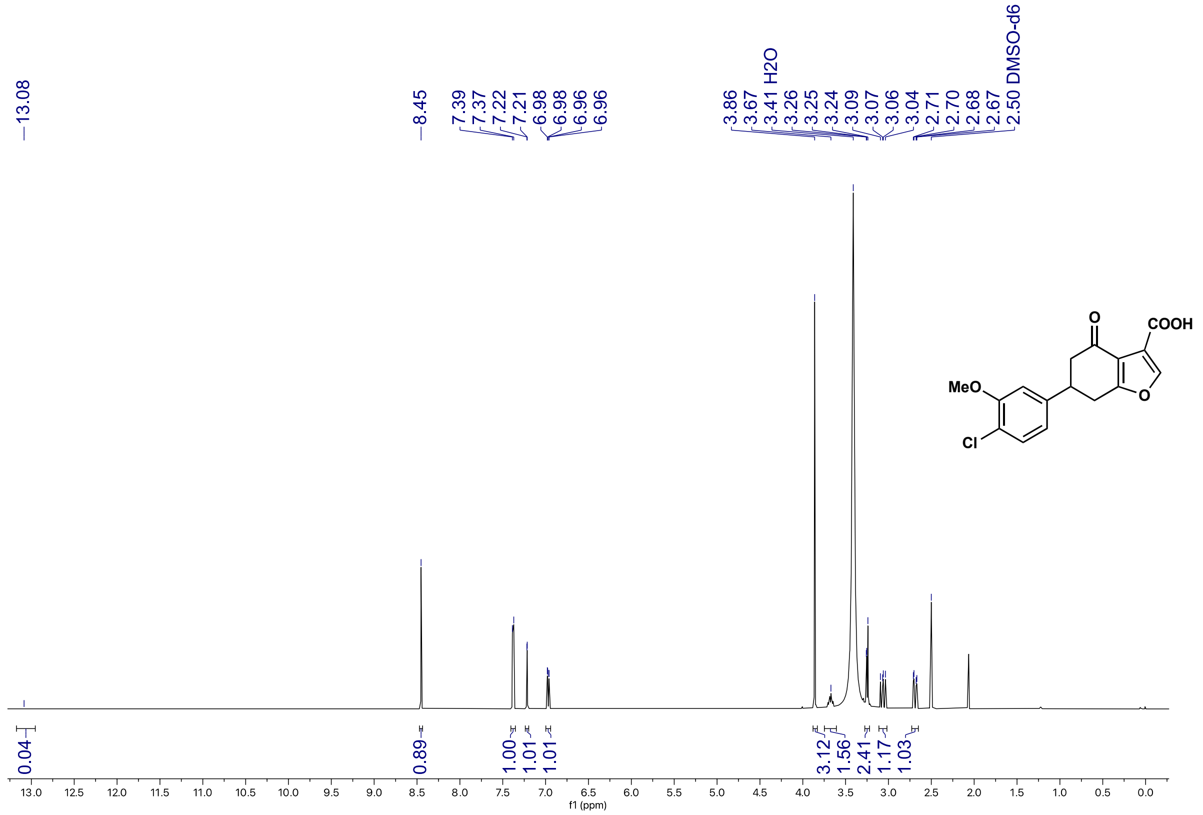

6-(4-chloro-3-methoxyphenyl)-4-oxo-4,5,6,7-tetrahydrobenzofuran-3-carboxylic acid (**28**) ^{13}C NMR (126 MHz, d_6-DMSO)

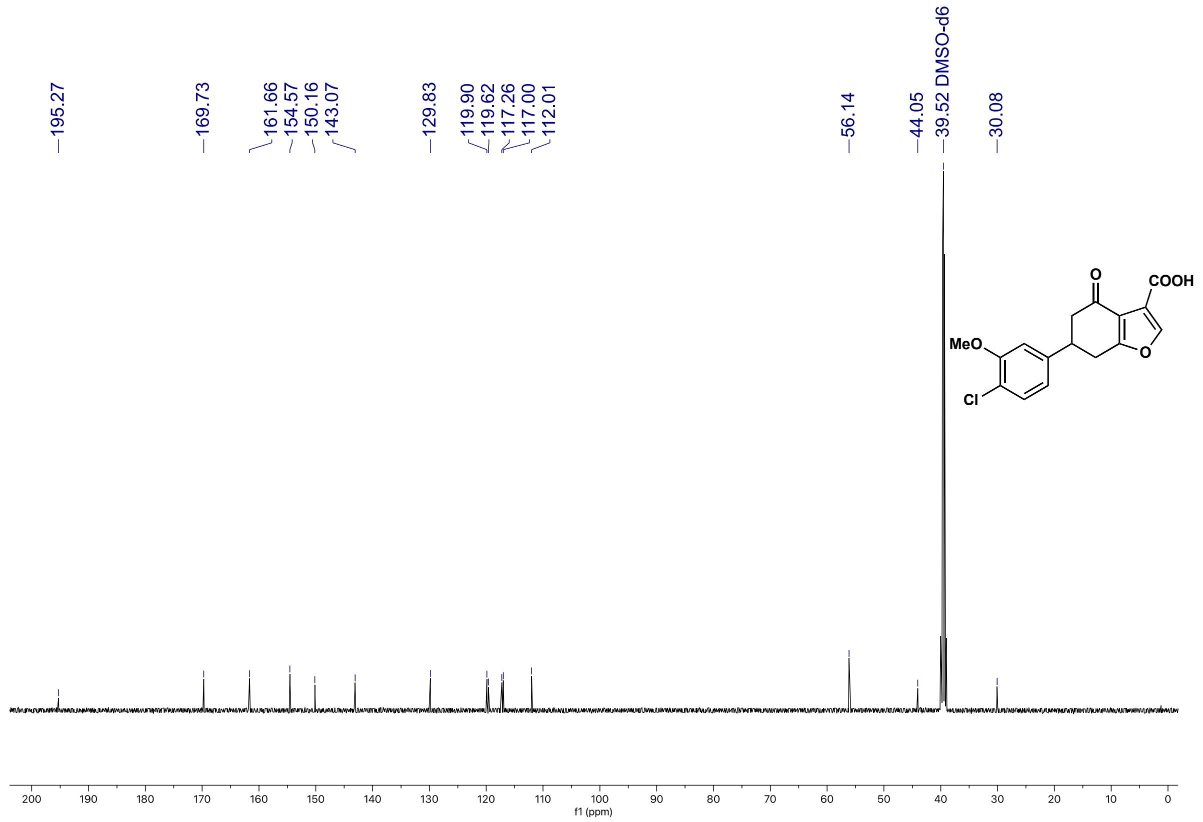

5-(thiophen-2-yl)cyclohexane-1,3-dione (**29c**) ^{1}H NMR (500 MHz, CD_3OD)

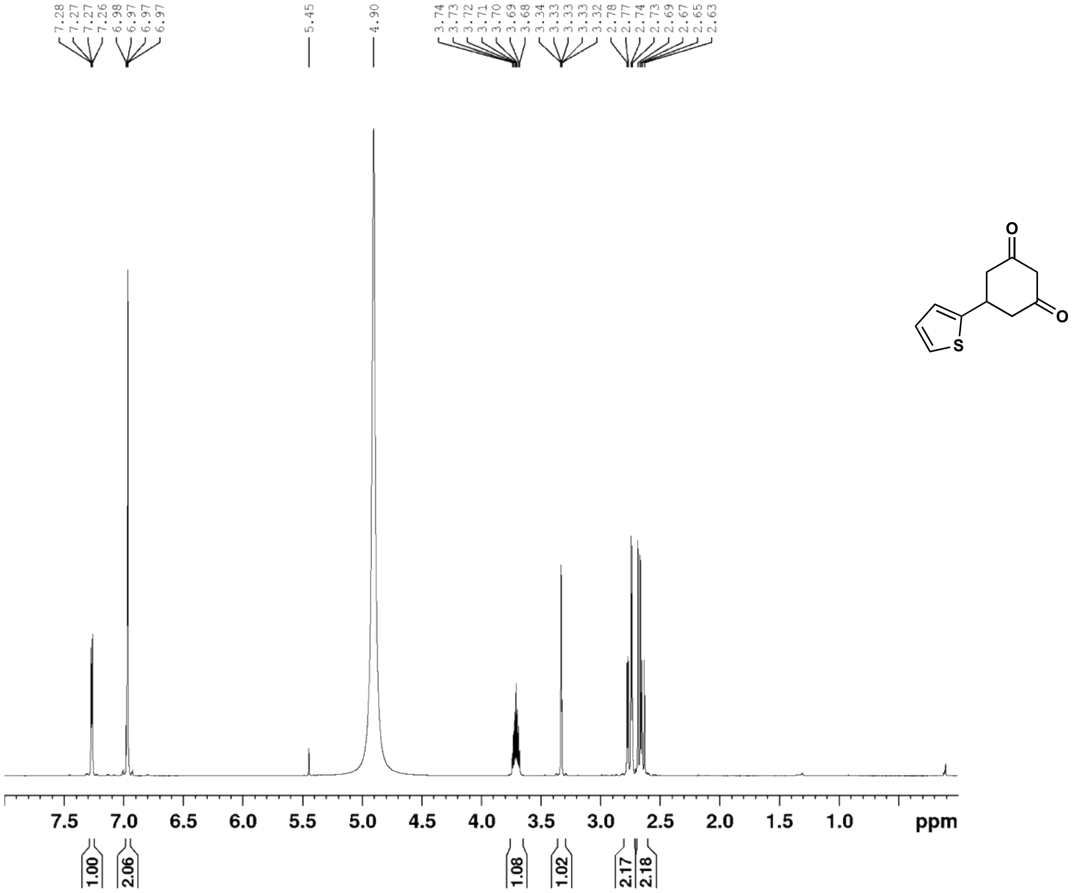

5-(thiophen-2-yl)cyclohexane-1,3-dione (**29c**) ^{13}C NMR (126 MHz, CD_3OD)

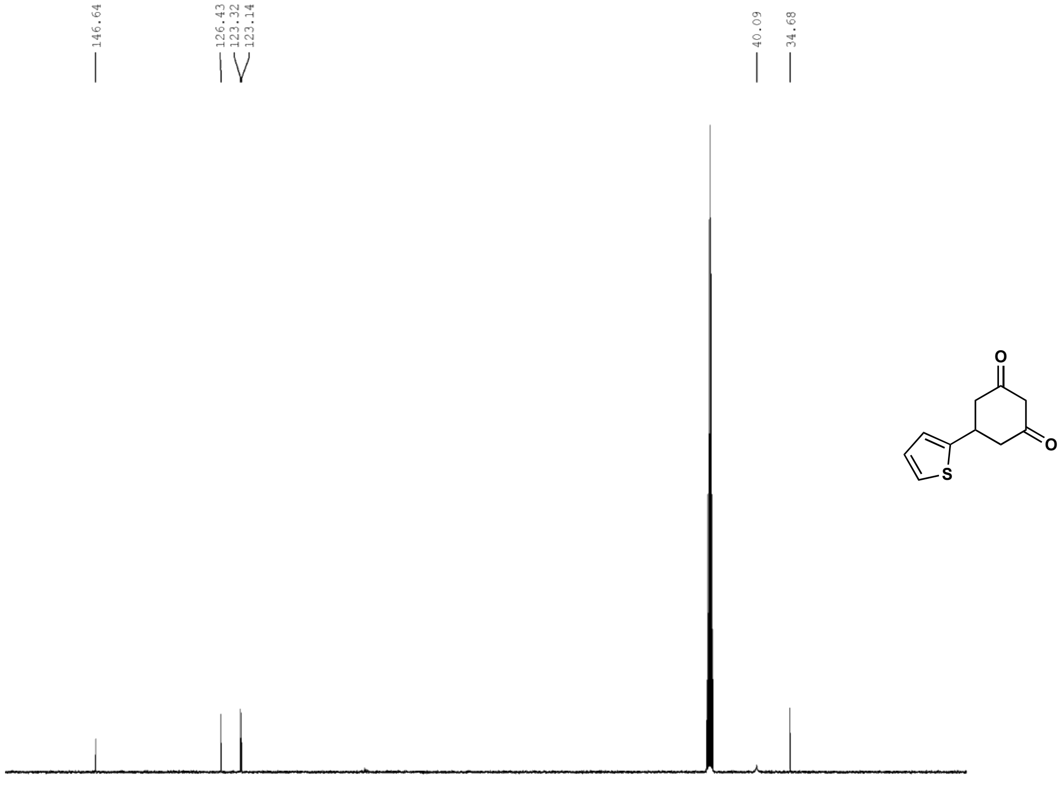

4-oxo-6-(thiophen-2-yl)-4,5,6,7-tetrahydrobenzofuran-3-carboxylic acid (**29**) ^{1}H NMR (500 MHz, $CDCl_3$)

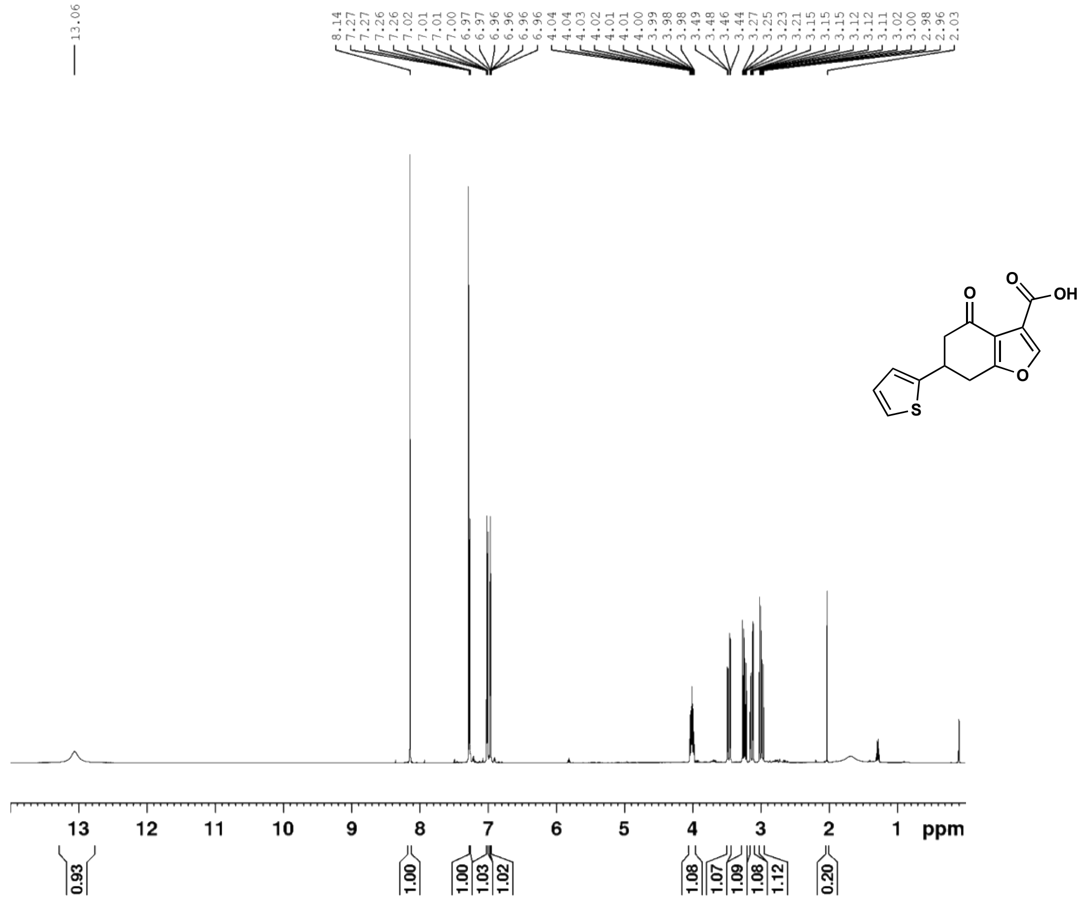

4-oxo-6-(thiophen-2-yl)-4,5,6,7-tetrahydrobenzofuran-3-carboxylic acid (**29**) ^{13}C NMR (126 MHz, $CDCl_3$)

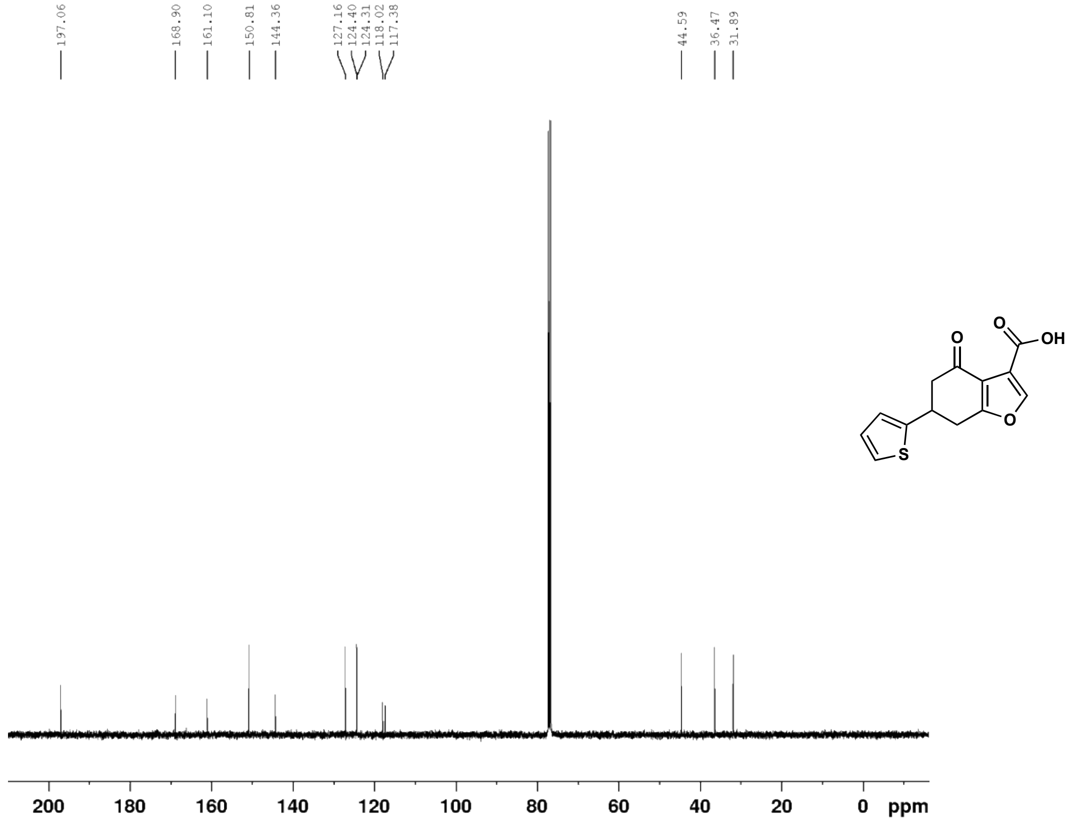

(E)-4-(3-bromothiophen-2-yl)but-3-en-2-one (**30b**) ^1H NMR (500 MHz, $CDCl_3$)

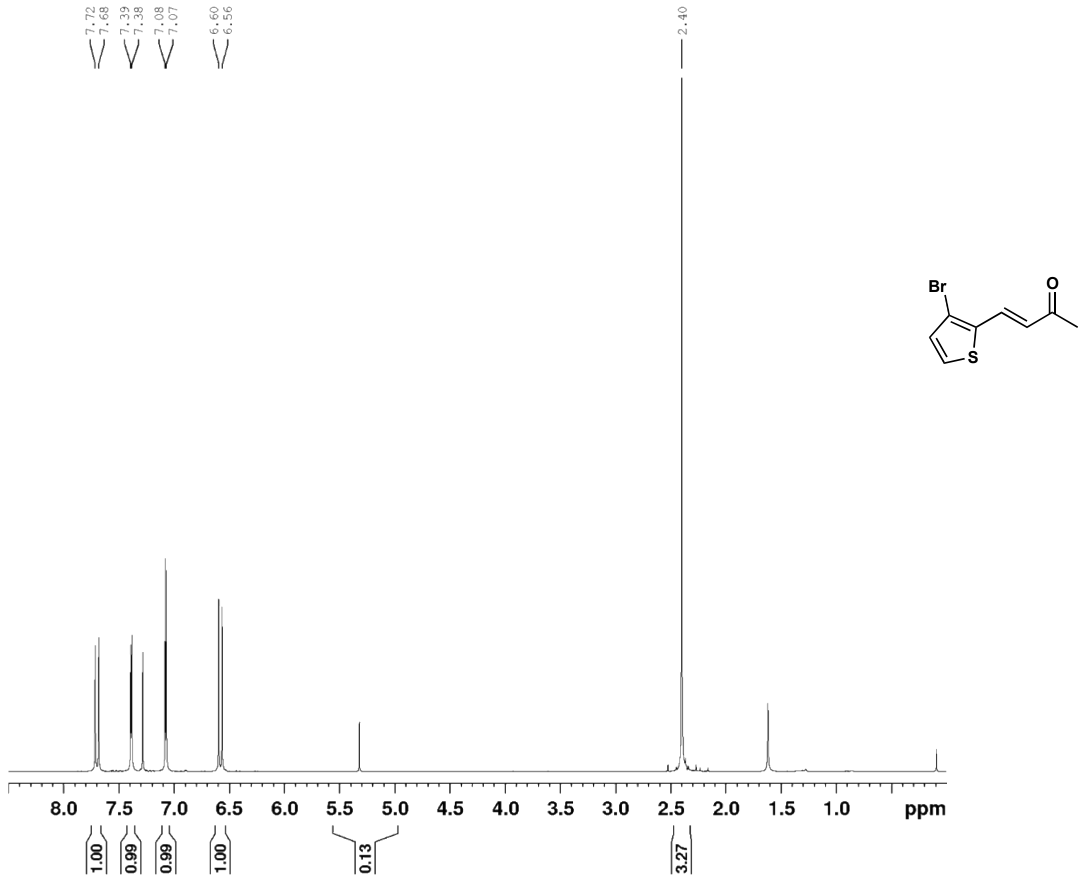

(E)-4-(3-bromothiophen-2-yl)but-3-en-2-one (**30b**) ^{13}C NMR (126 MHz, $CDCl_3$)

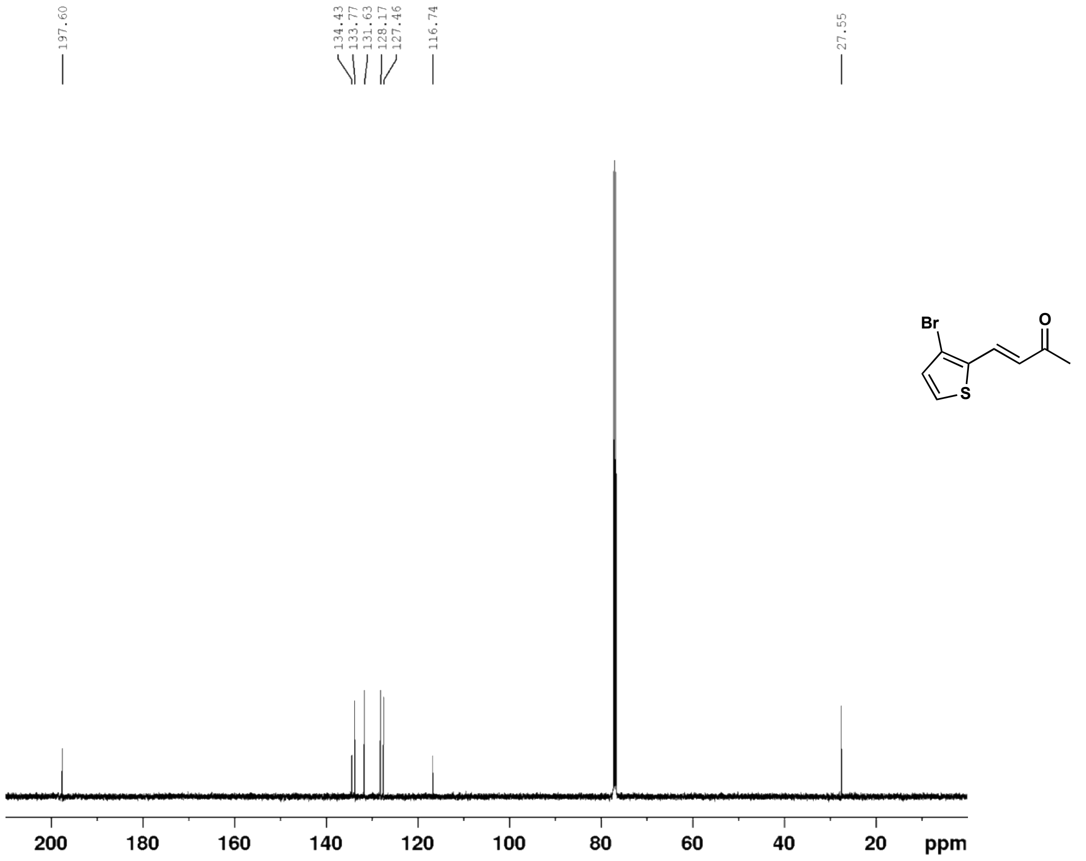

5-(3-bromothiophen-2-yl)cyclohexane-1,3-dione (**30c**) ^{1}H NMR (500 MHz, CD_3OD)

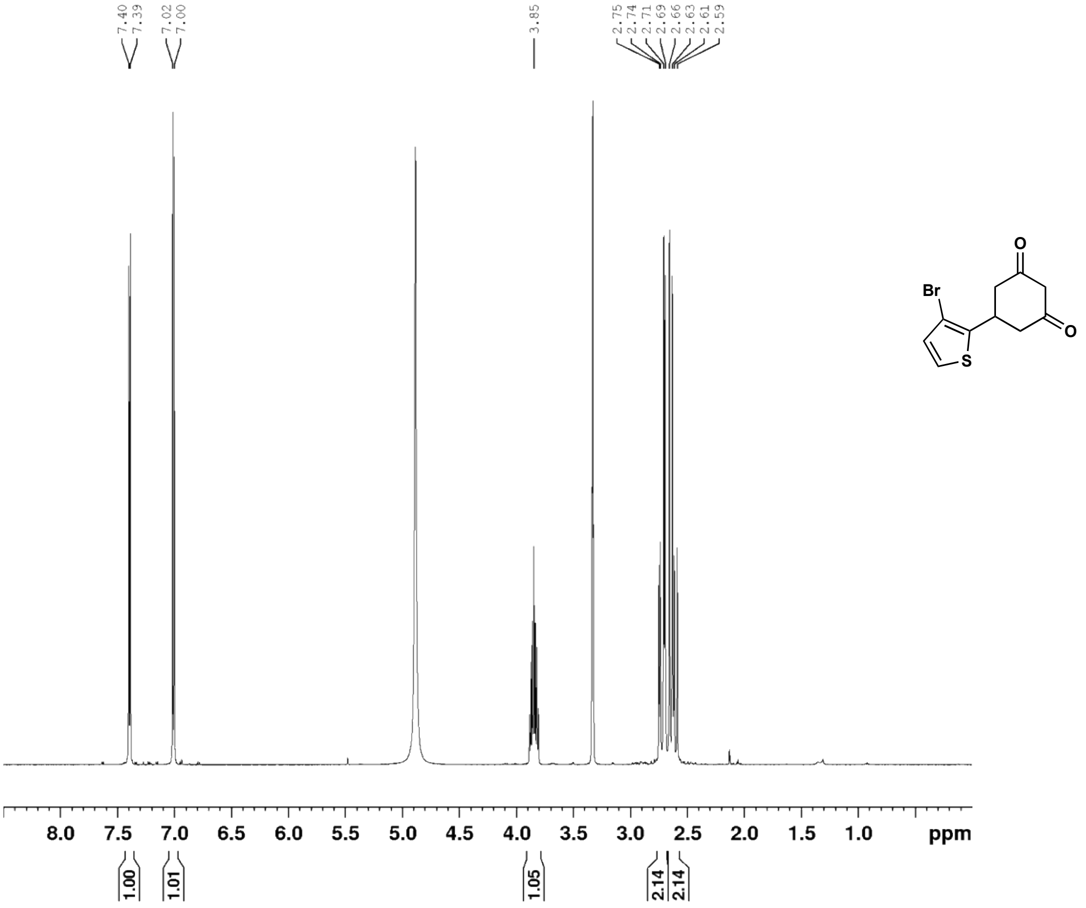

5-(3-bromothiophen-2-yl)cyclohexane-1,3-dione (**30c**) ^{13}C NMR (126 MHz, CD_3OD)

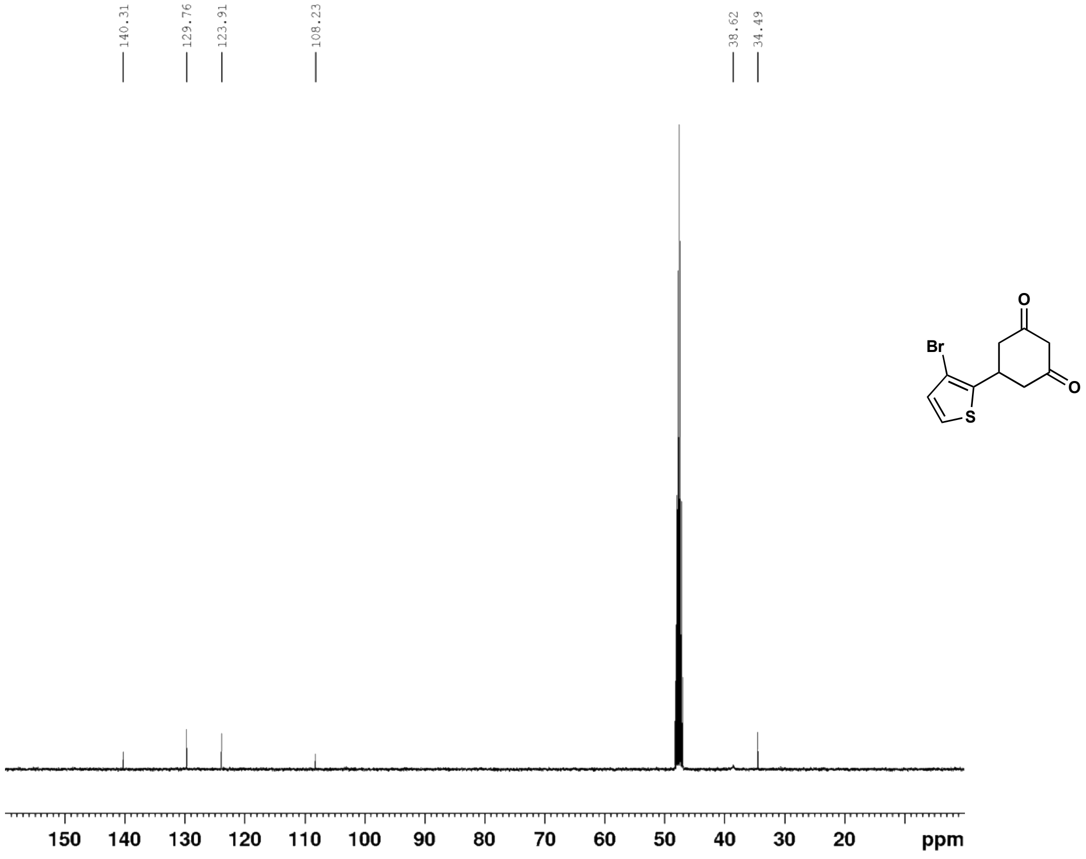

6-(3-bromothiophen-2-yl)-4-oxo-4,5,6,7-tetrahydrobenzofuran-3-carboxylic acid (**30**) ^{1}H NMR (500 MHz, $CDCl_3$)

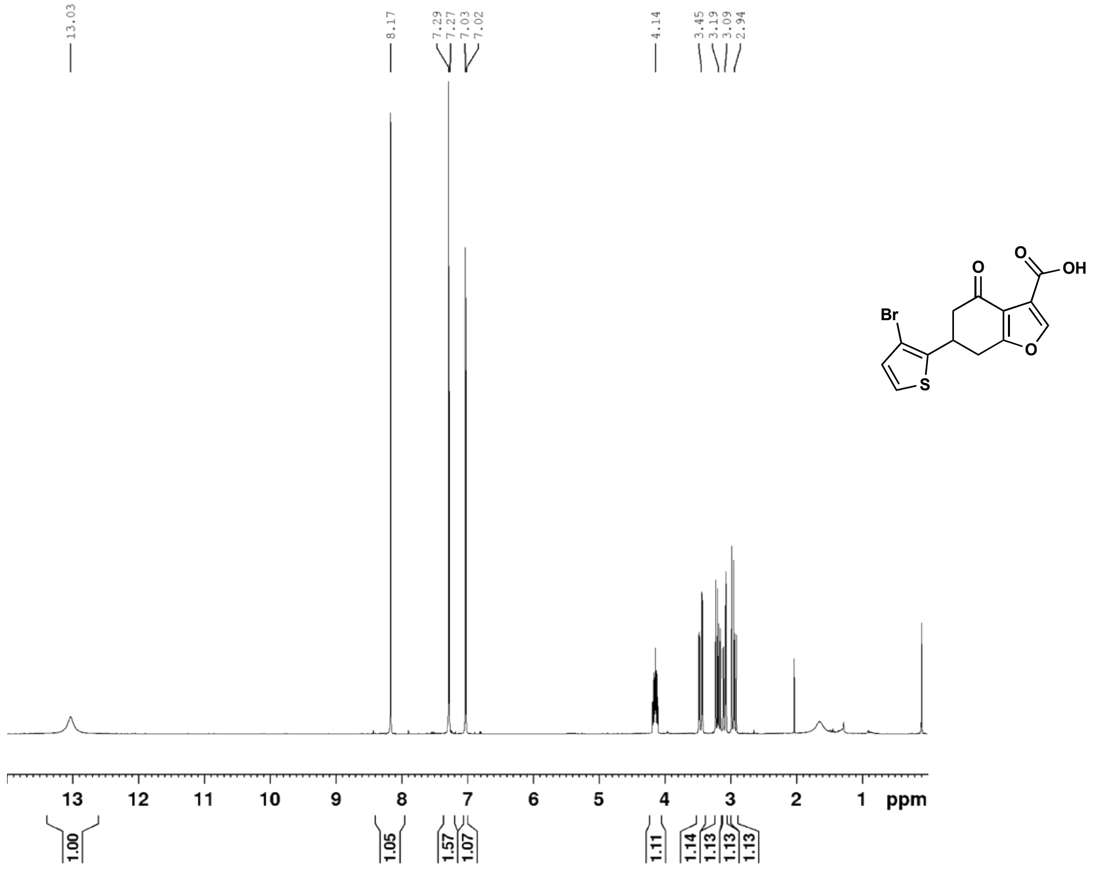

6-(3-bromothiophen-2-yl)-4-oxo-4,5,6,7-tetrahydrobenzofuran-3-carboxylic acid (**30**) ^{13}C NMR (126 MHz, $CDCl_3$)

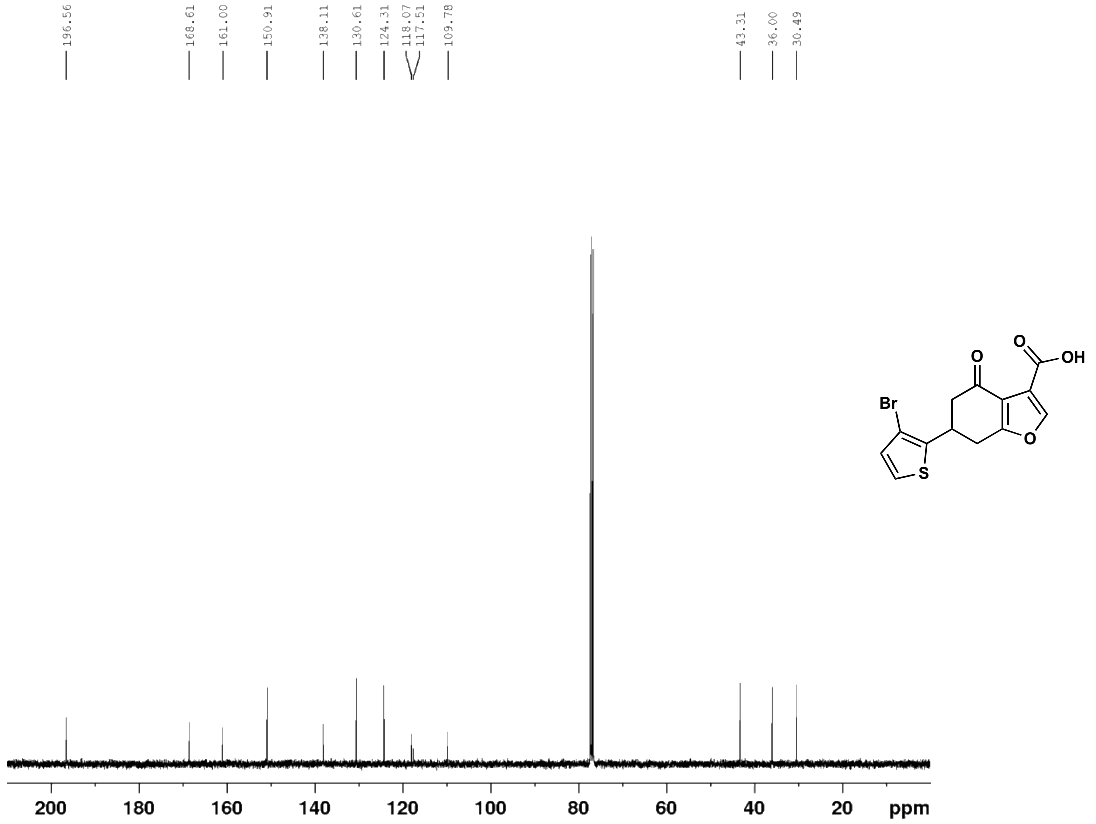

(E)-4-(5-bromothiophen-2-yl)but-3-en-2-one (**31b**) ^{1}H NMR (500 MHz, $CDCl_3$)

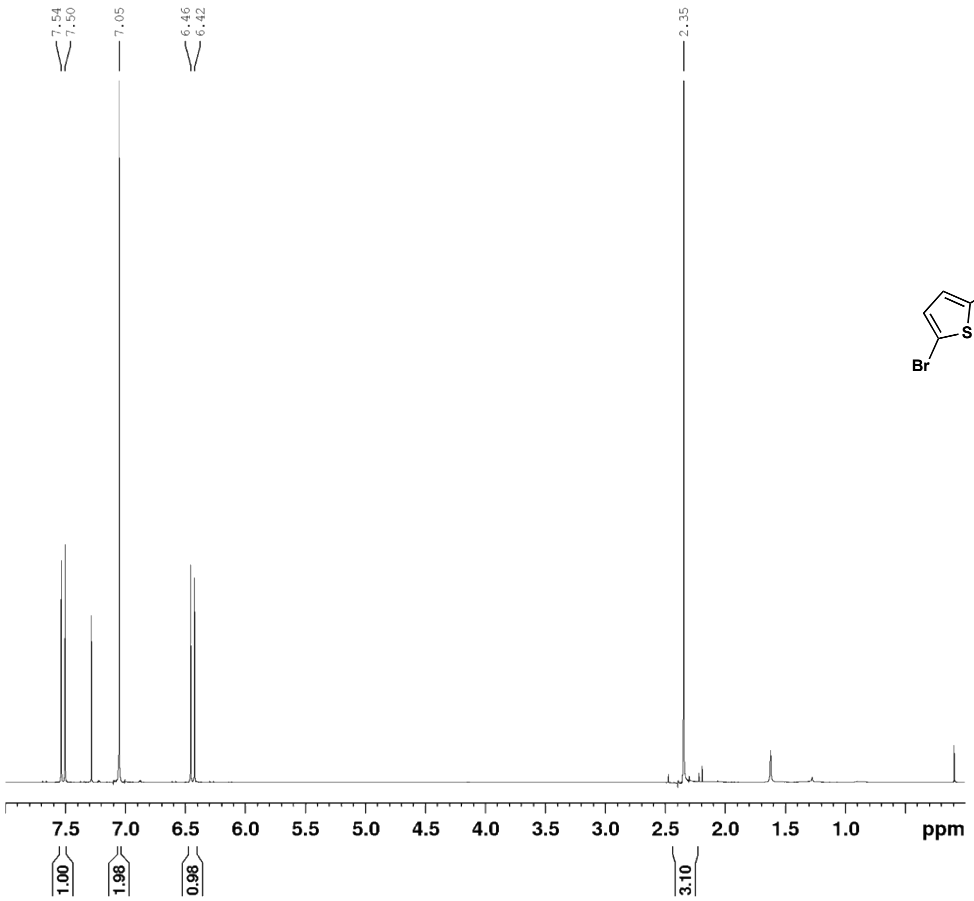

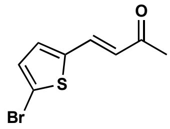

(E)-4-(5-bromothiophen-2-yl)but-3-en-2-one (**31b**) ^{13}C NMR (126 MHz, $CDCl_3$)

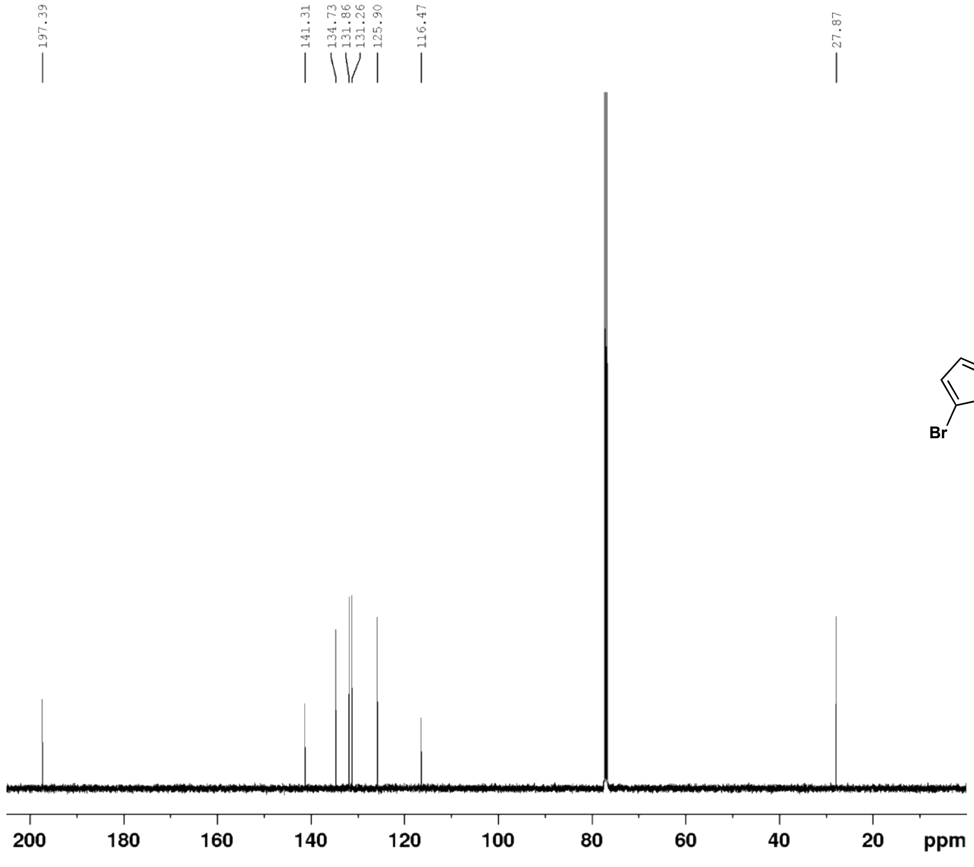

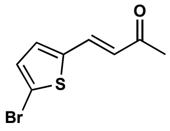

5-(5-bromothiophen-2-yl)cyclohexane-1,3-dione (**31c**) ^{1}H NMR (500 MHz, CD_3OD)

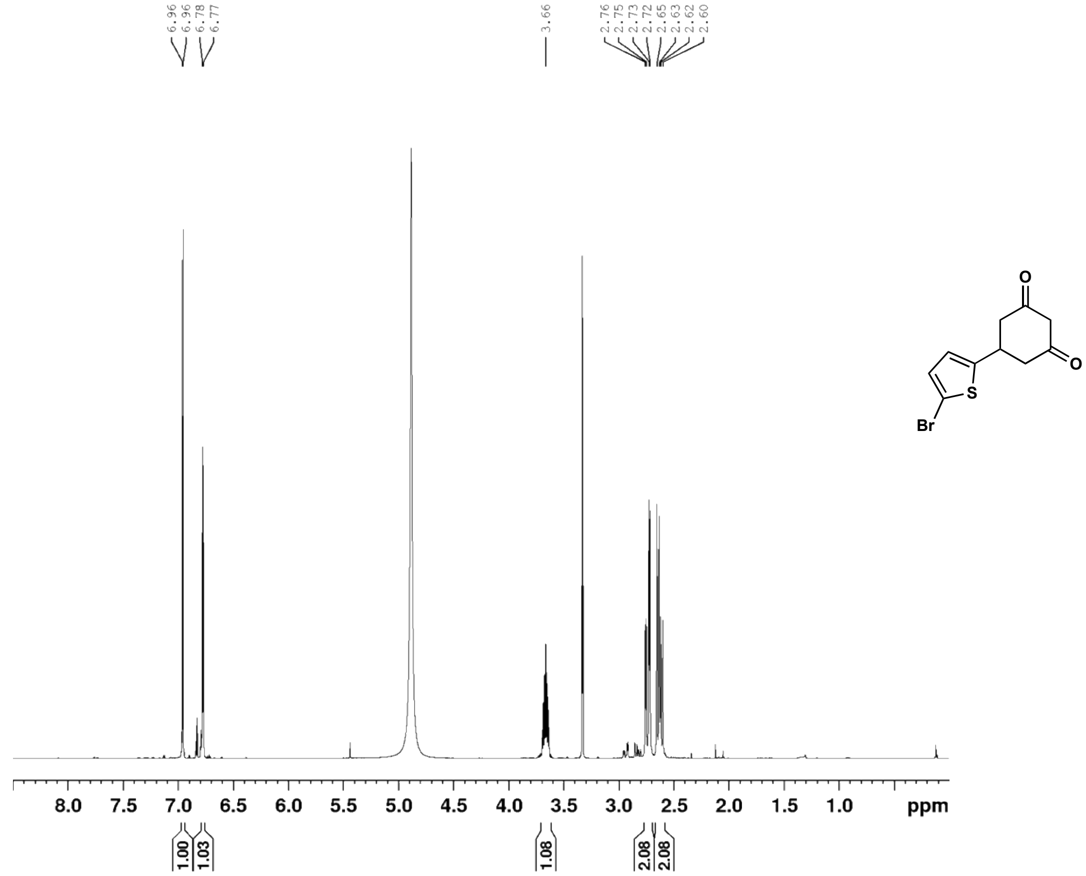

5-(5-bromothiophen-2-yl)cyclohexane-1,3-dione (**31c**) ^{13}C NMR (126 MHz, CD_3OD)

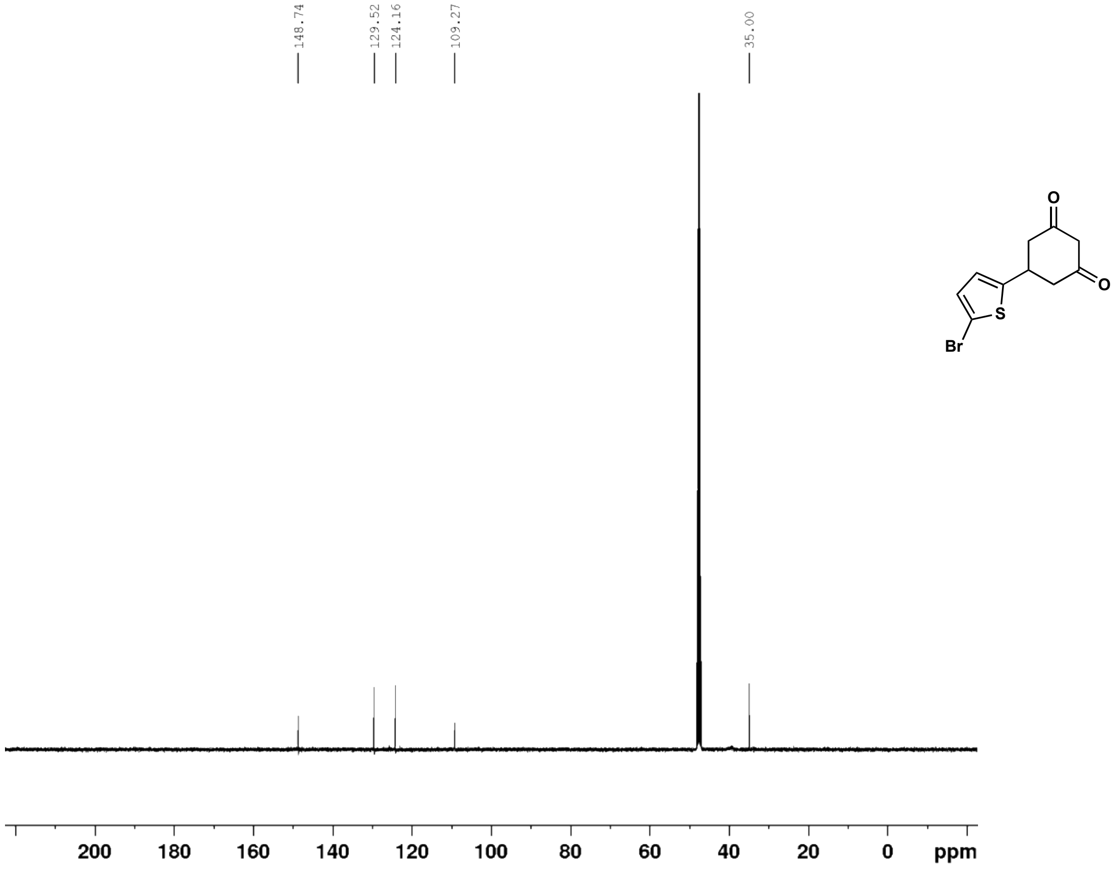

6-(5-bromothiophen-2-yl)-4-oxo-4,5,6,7-tetrahydrobenzofuran-3-carboxylic acid (**31**) ^{1}H NMR (500 MHz, d$_6$-DMSO)

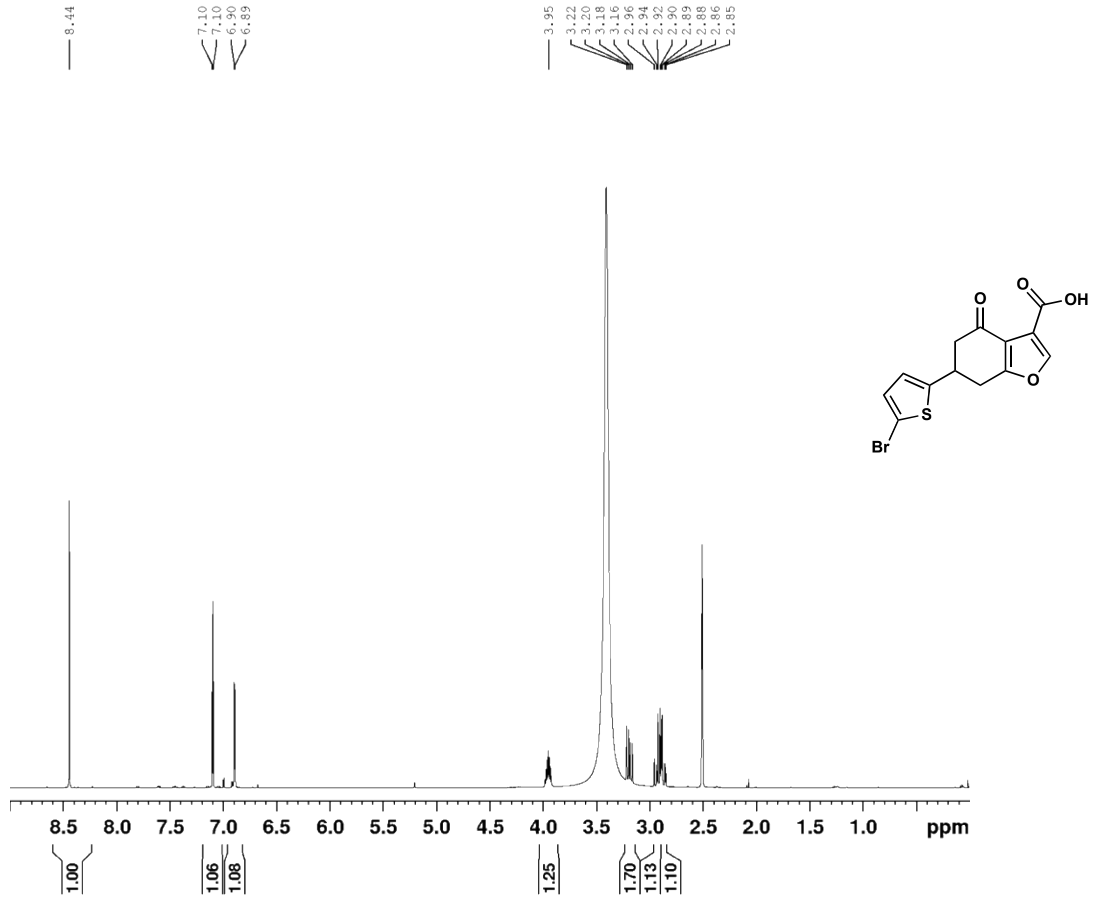

6-(5-bromothiophen-2-yl)-4-oxo-4,5,6,7-tetrahydrobenzofuran-3-carboxylic acid (**31**) ^{13}C NMR (126 MHz, d$_6$-DMSO)

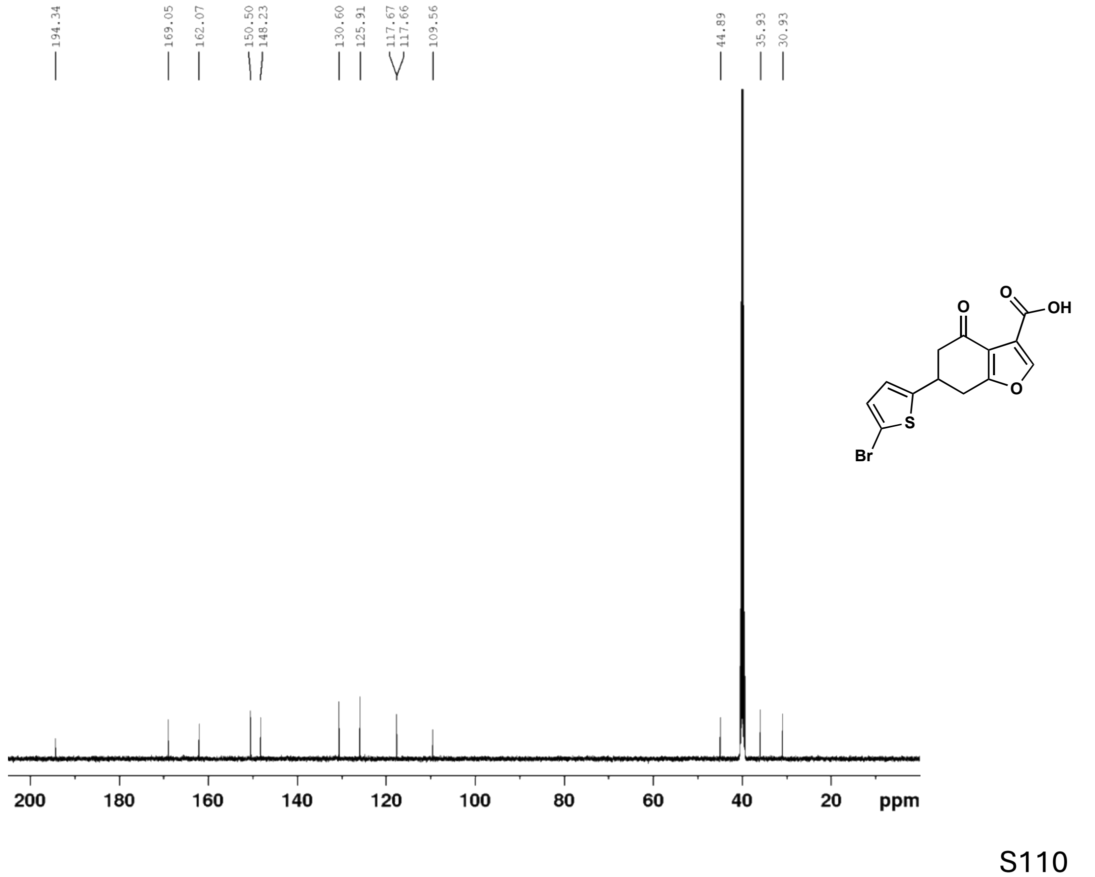

(E)-4-(5-chlorothiophen-2-yl)but-3-en-2-one (**32b**) ^{1}H NMR (500 MHz, $CDCl_3$)

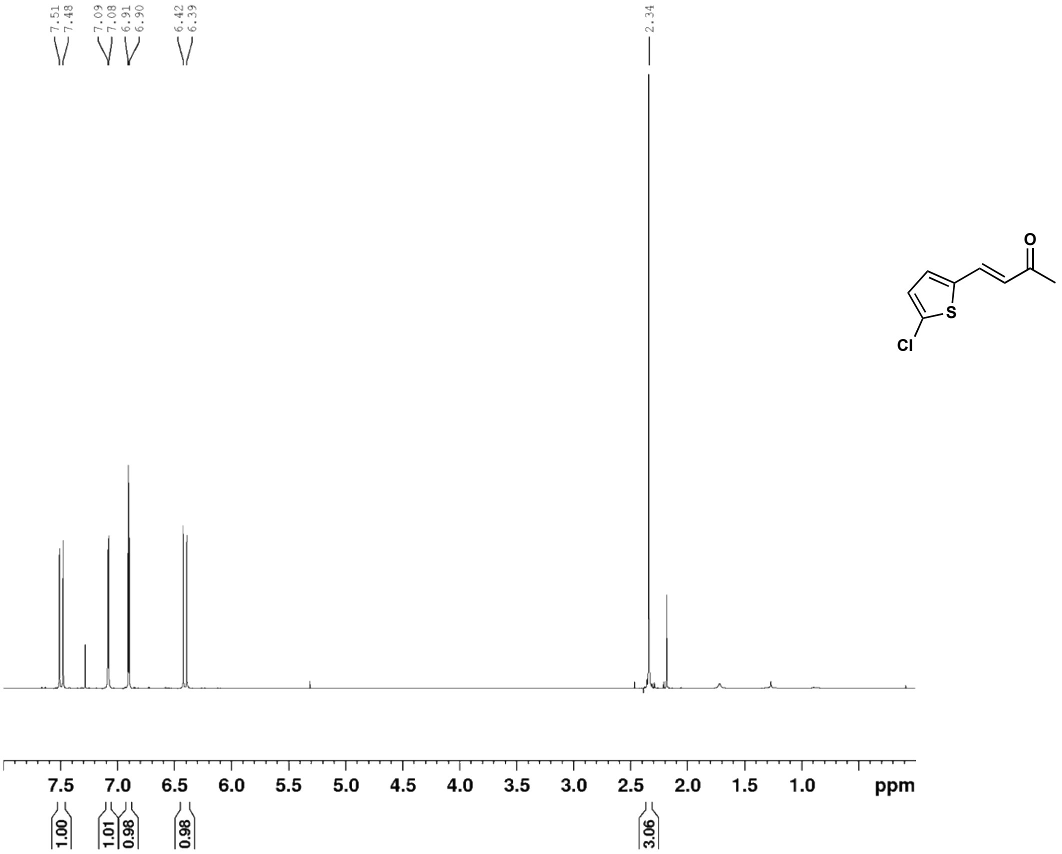

(E)-4-(5-chlorothiophen-2-yl)but-3-en-2-one (**32b**) ^{13}C NMR (126 MHz, $CDCl_3$)

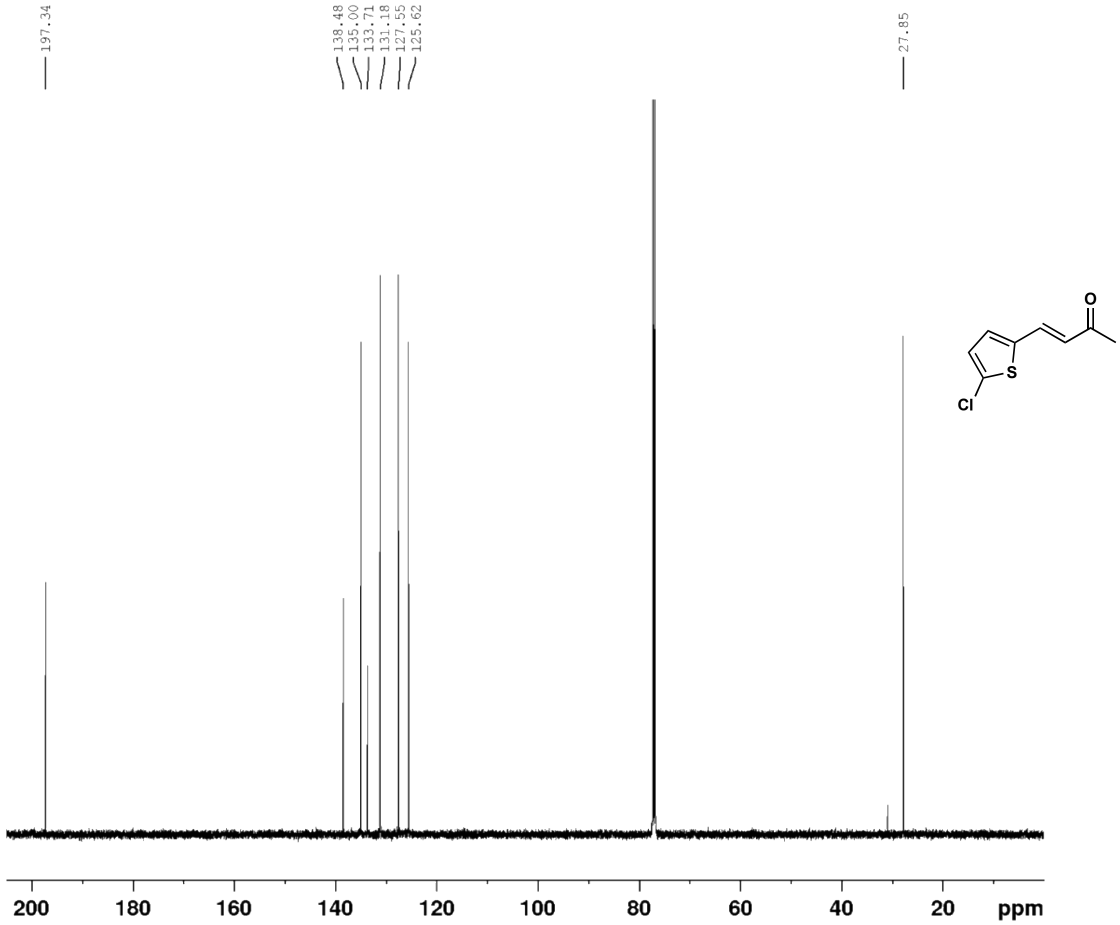

5-(5-chlorothiophen-2-yl)cyclohexane-1,3-dione (**32c**) ^{1}H NMR (500 MHz, CD_3OD)

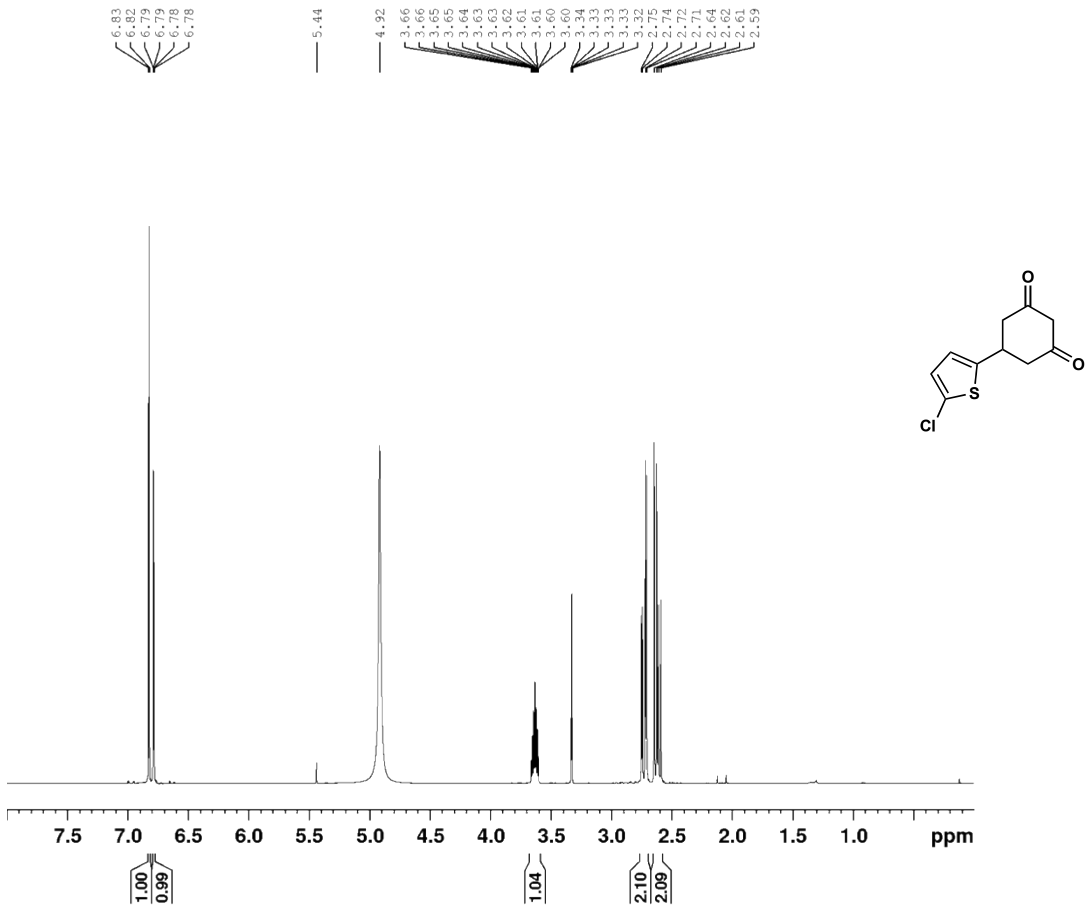

5-(5-chlorothiophen-2-yl)cyclohexane-1,3-dione (**32c**) ^{13}C NMR (126 MHz, CD_3OD)

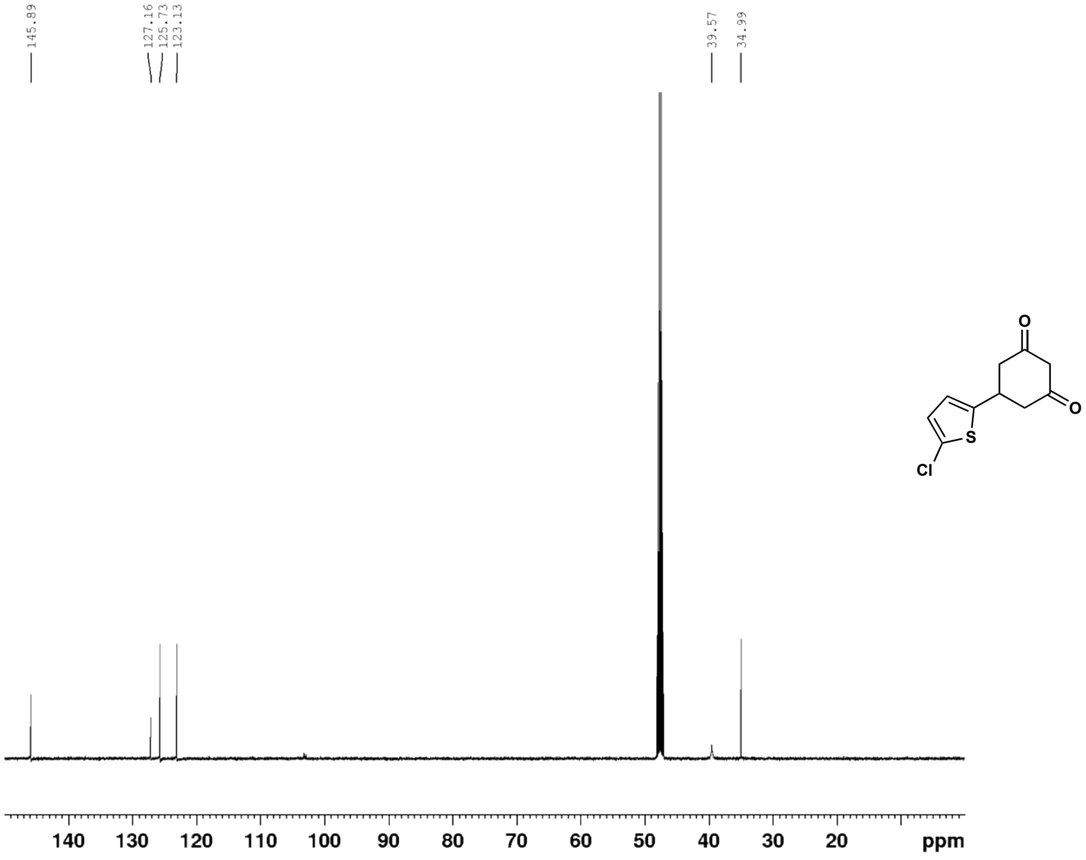

6-(5-chlorothiophen-2-yl)-4-oxo-4,5,6,7-tetrahydrobenzofuran-3-carboxylic acid (**32**) ^{1}H NMR (500 MHz, d_6-DMSO)

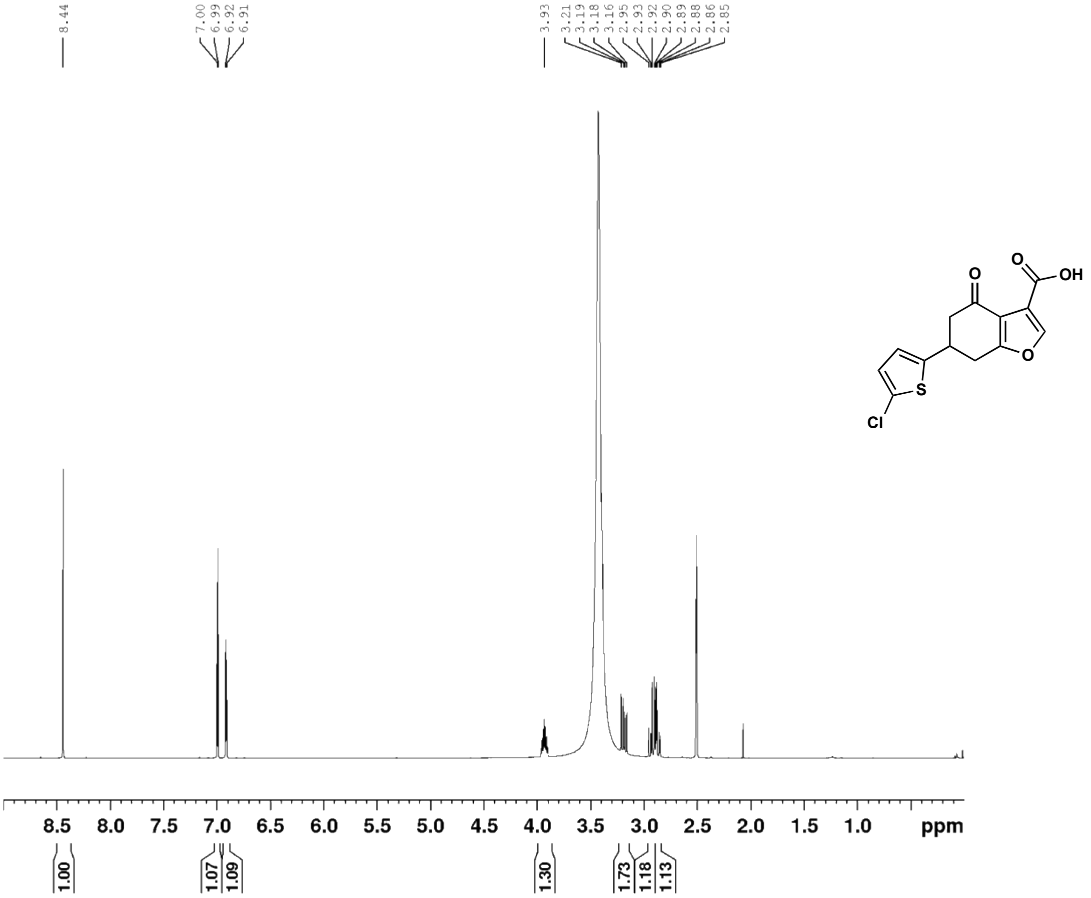

6-(5-chlorothiophen-2-yl)-4-oxo-4,5,6,7-tetrahydrobenzofuran-3-carboxylic acid (**32**) ^{13}C NMR (126 MHz, d_6-DMSO)

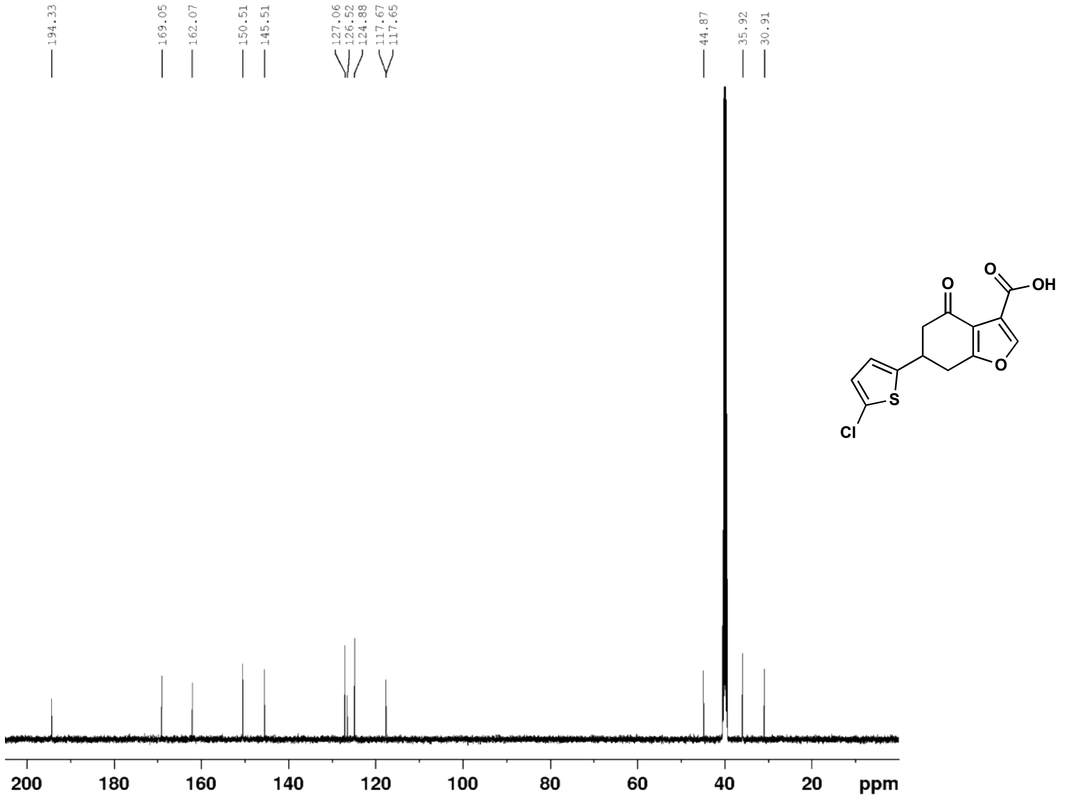

(E)-4-(4-bromothiophen-2-yl)but-3-en-2-one (**33b**) ^{1}H NMR (500 MHz, $CDCl_3$)

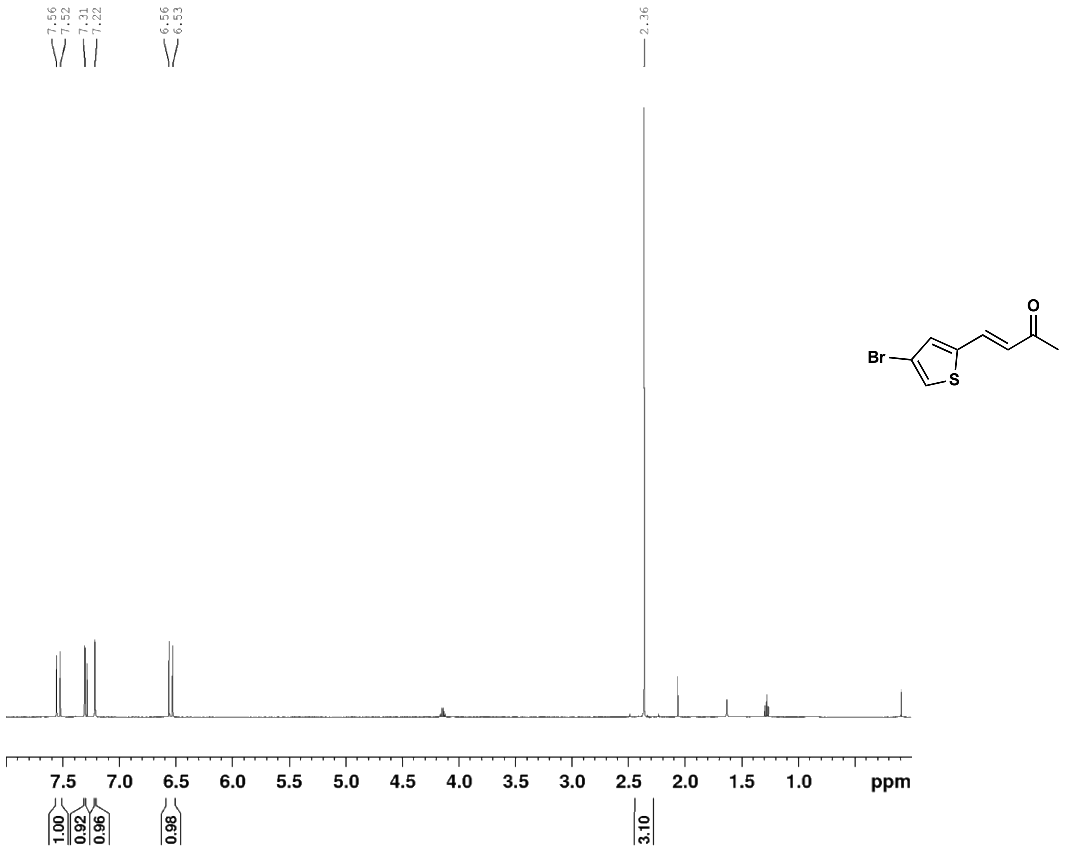

(E)-4-(4-bromothiophen-2-yl)but-3-en-2-one (**33b**) ^{13}C NMR (126 MHz, $CDCl_3$)

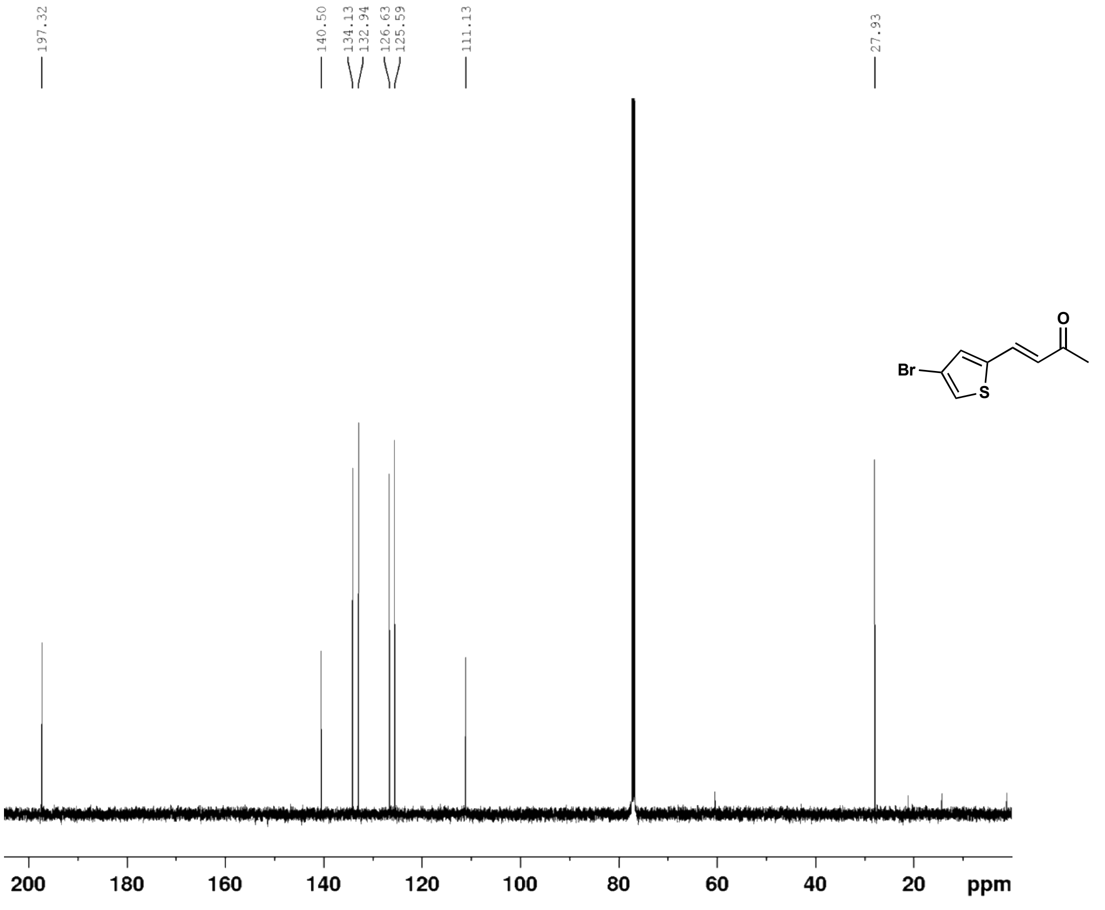

5-(4-bromothiophen-2-yl)cyclohexane-1,3-dione (**33c**) ^{1}H NMR (500 MHz, CD_3OD)

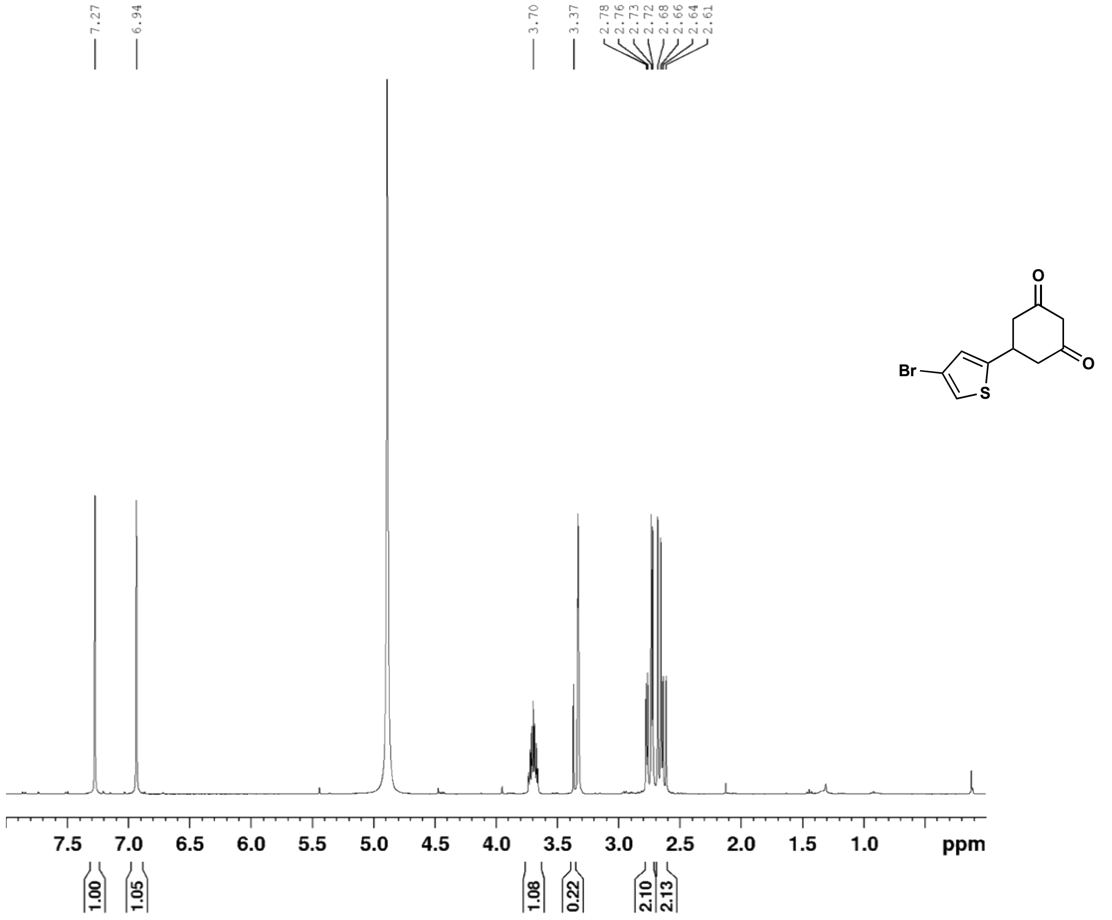

5-(4-bromothiophen-2-yl)cyclohexane-1,3-dione (**33c**) ^{13}C NMR (126 MHz, CD_3OD)

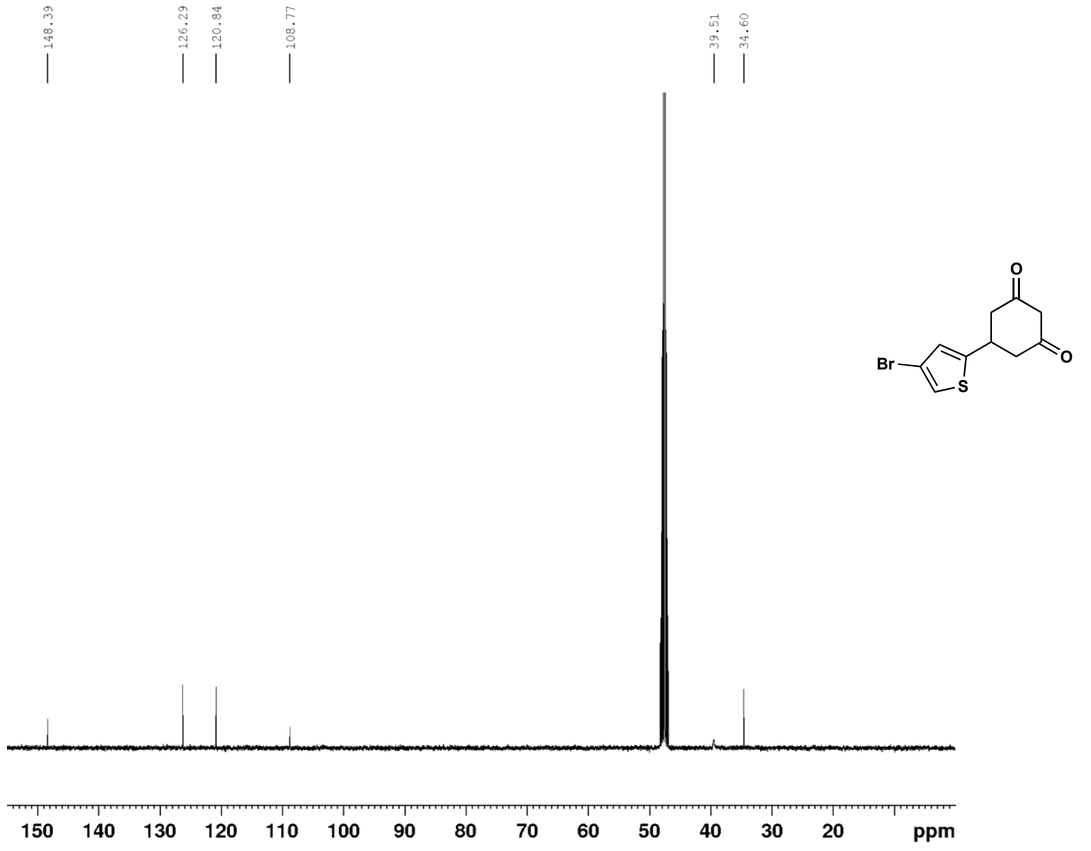

6-(4-bromothiophen-2-yl)-4-oxo-4,5,6,7-tetrahydrobenzofuran-3-carboxylic acid (**33**) ^{1}H NMR (500 MHz, d_6-DMSO)

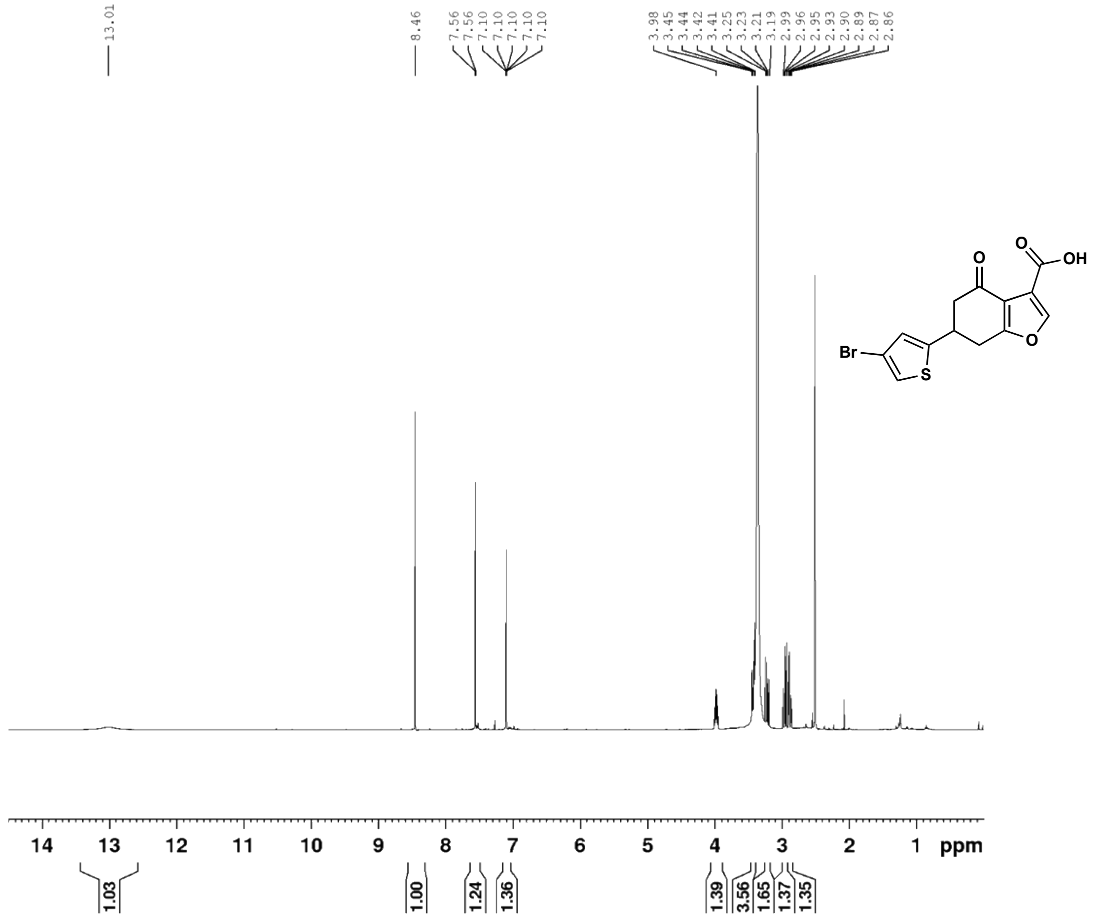

6-(4-bromothiophen-2-yl)-4-oxo-4,5,6,7-tetrahydrobenzofuran-3-carboxylic acid (**33**) ^{13}C NMR (126 MHz, d_6-DMSO)

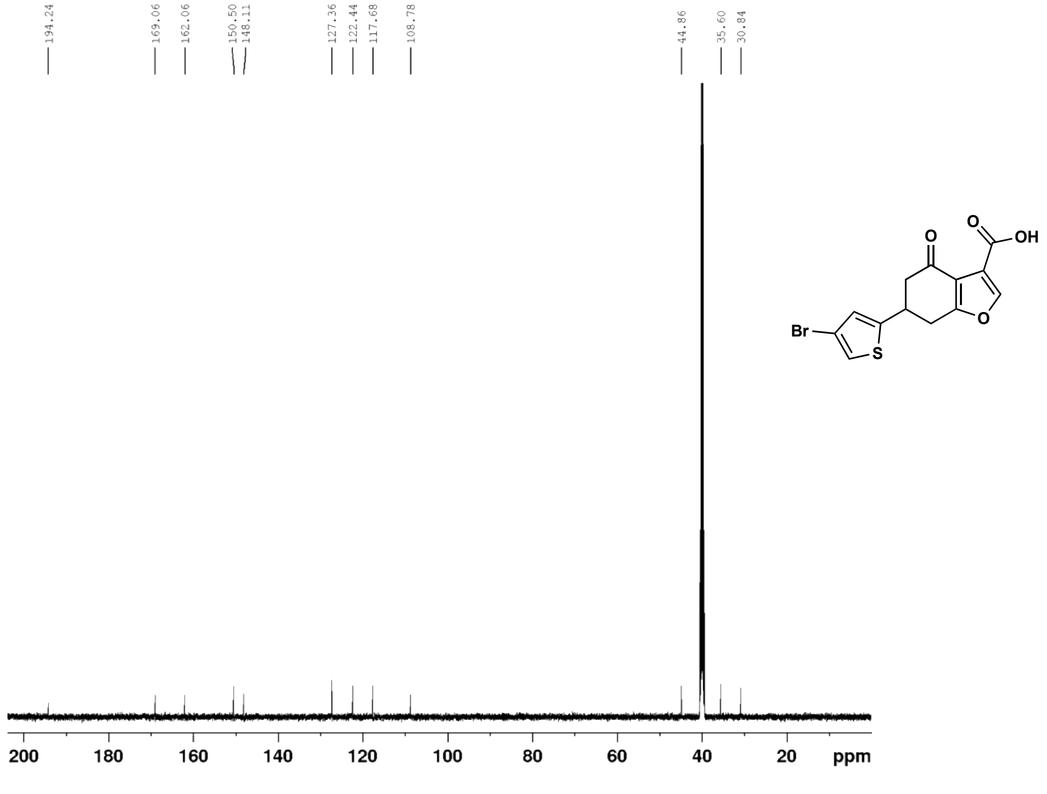

ethyl 6-(4-bromothiophen-2-yl)-4-oxo-4,5,6,7-tetrahydrobenzofuran-3-carboxylate (**33d**) ^{1}H NMR (500 MHz, $CDCl_3$)

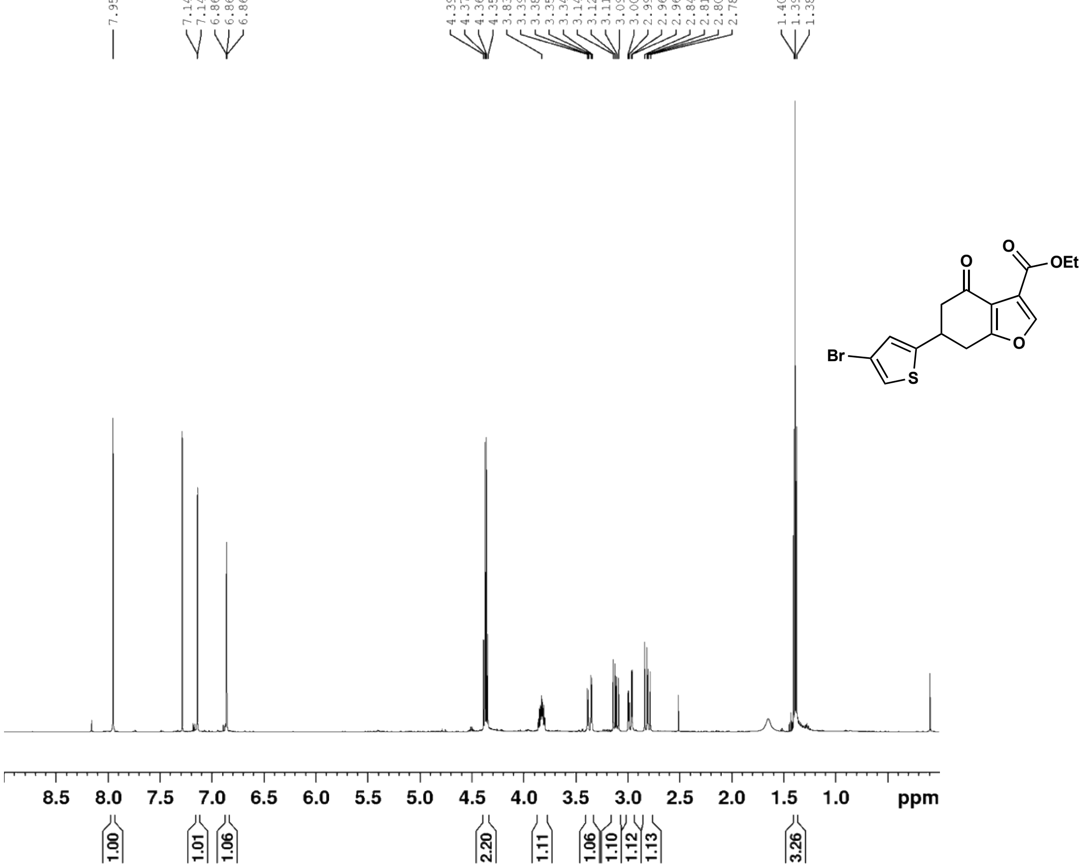

ethyl 6-(4-bromothiophen-2-yl)-4-oxo-4,5,6,7-tetrahydrobenzofuran-3-carboxylate (**33d**) ^{13}C NMR (126 MHz, $CDCl_3$)

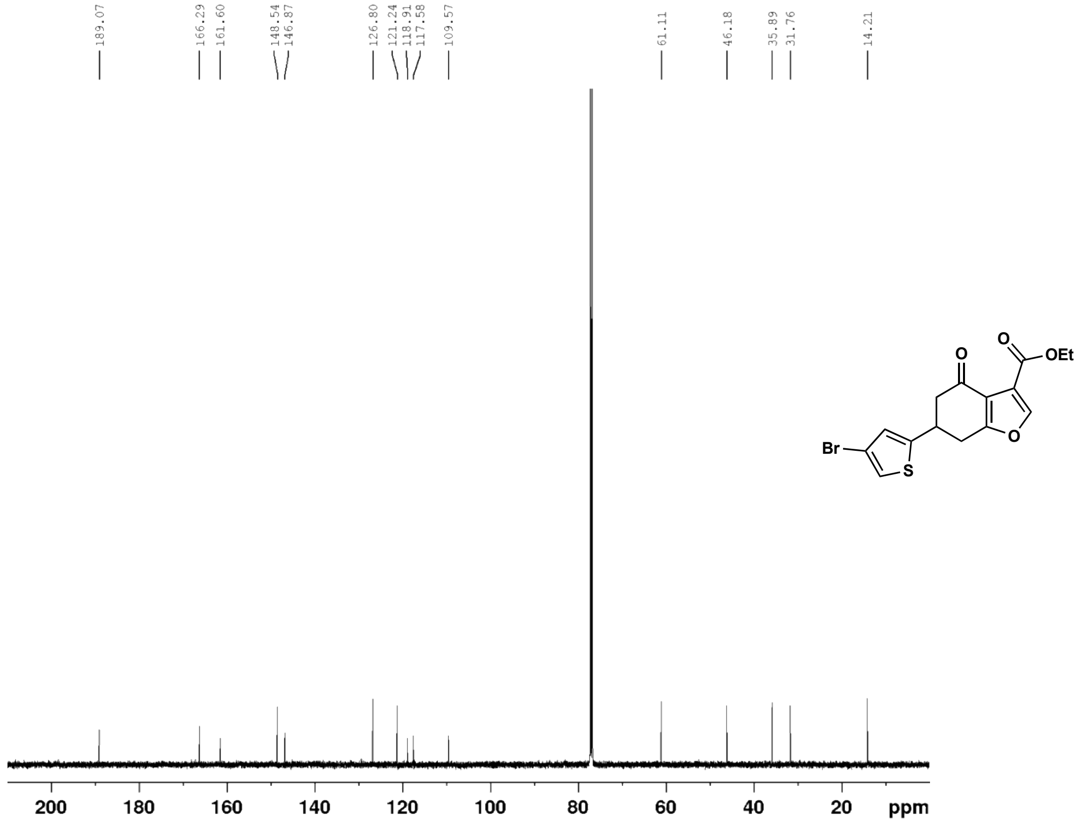

(E)-4-(4,5-dibromothiophen-2-yl)but-3-en-2-one (**34b**) ^{1}H NMR (500 MHz, $CDCl_3$)

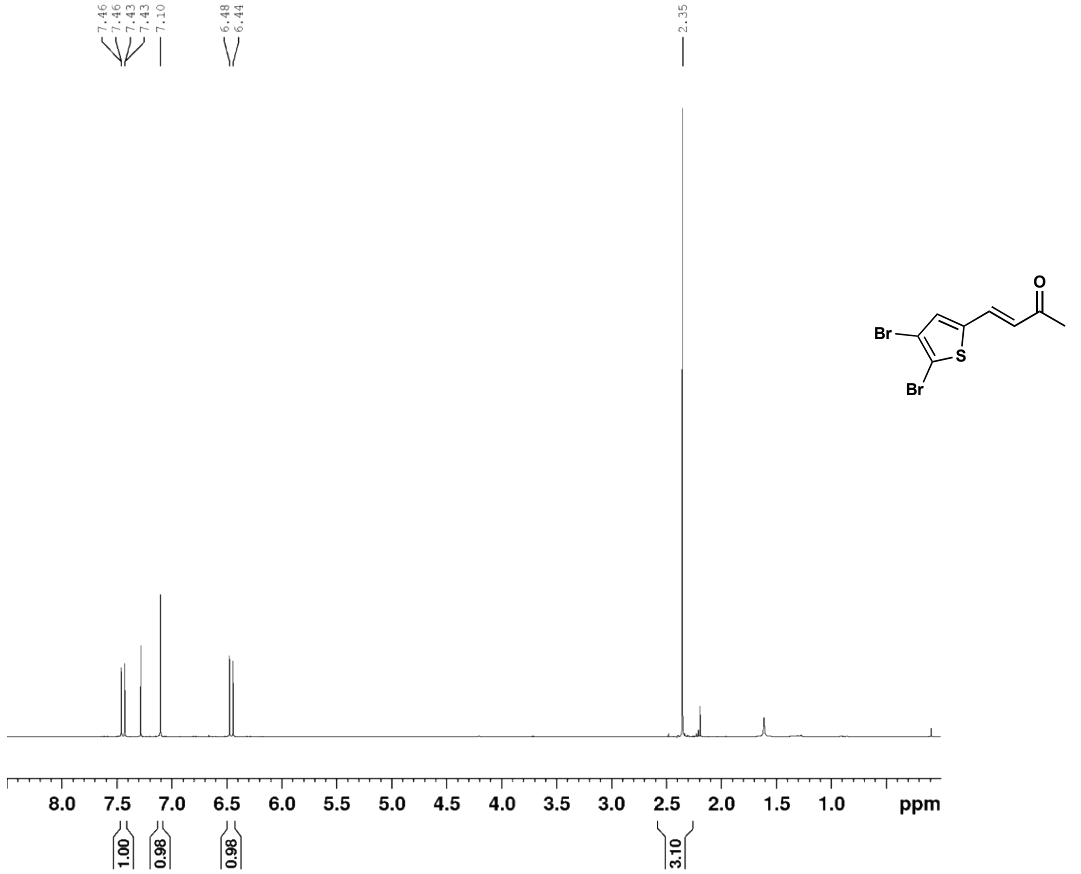

(E)-4-(4,5-dibromothiophen-2-yl)but-3-en-2-one (**34b**) ^{13}C NMR (126 MHz, $CDCl_3$)

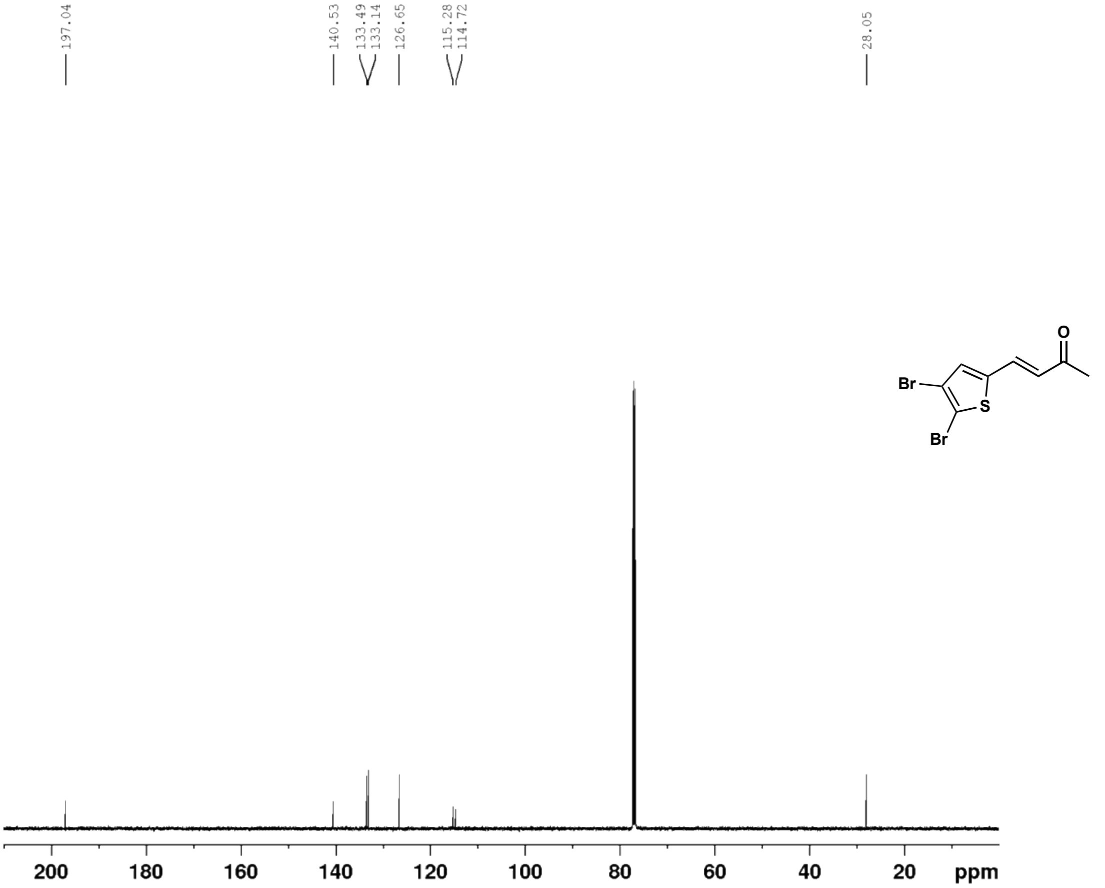

5-(4,5-dibromothiophen-2-yl)cyclohexane-1,3-dione (**34c**) ^{1}H NMR (500 MHz, d_6-DMSO)

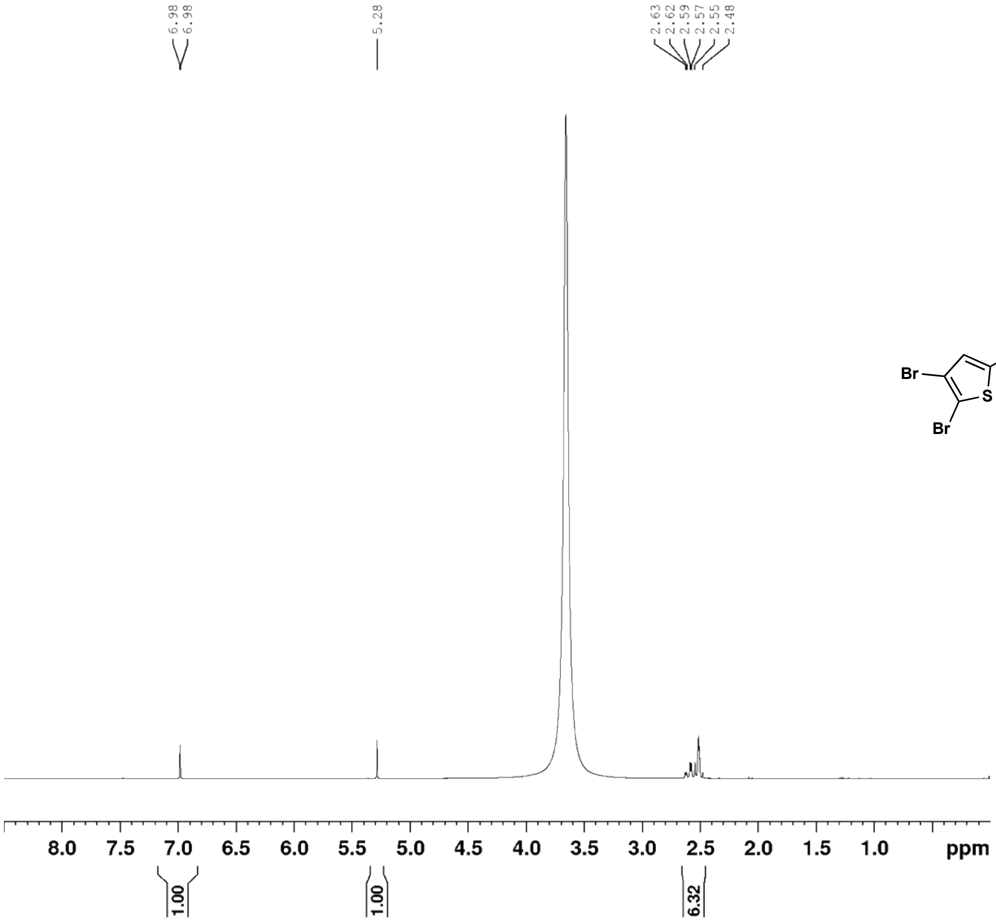

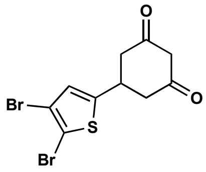

5-(4,5-dibromothiophen-2-yl)cyclohexane-1,3-dione (**34c**) ^{13}C NMR (126 MHz, d_6-DMSO)

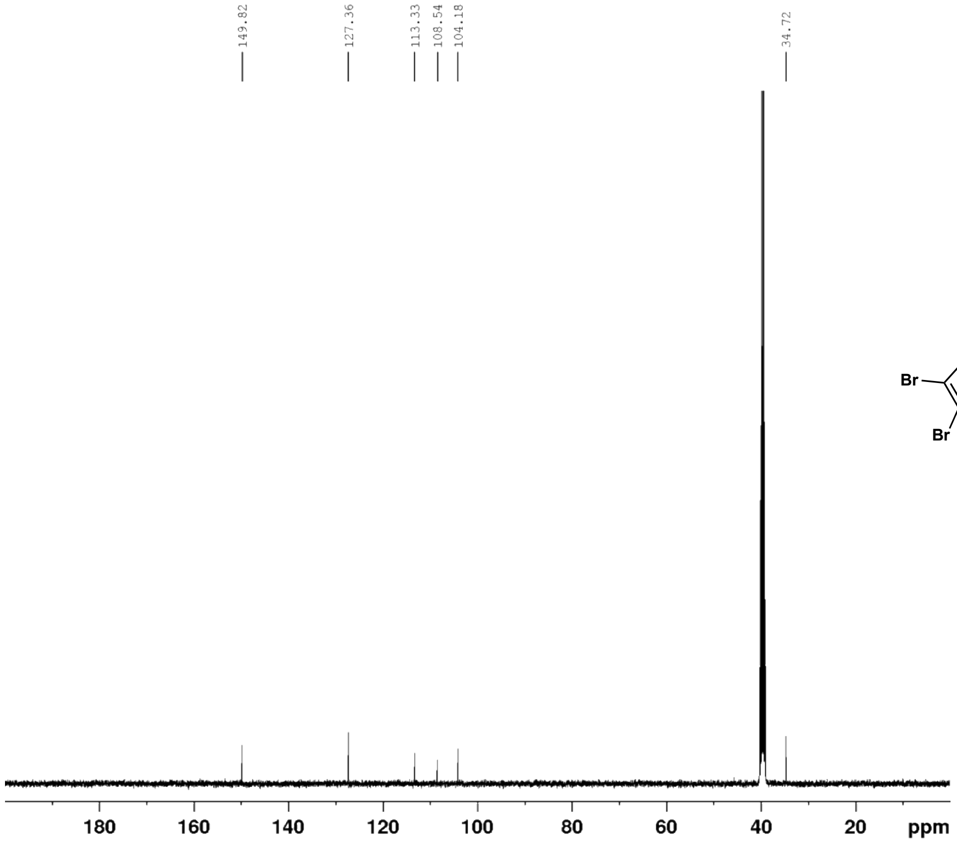

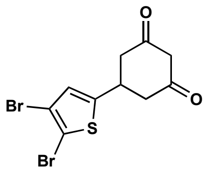

6-(4,5-dibromothiophen-2-yl)-4-oxo-4,5,6,7-tetrahydrobenzofuran-3-carboxylic acid (**34**) ^{1}H NMR (500 MHz, d_6-DMSO)

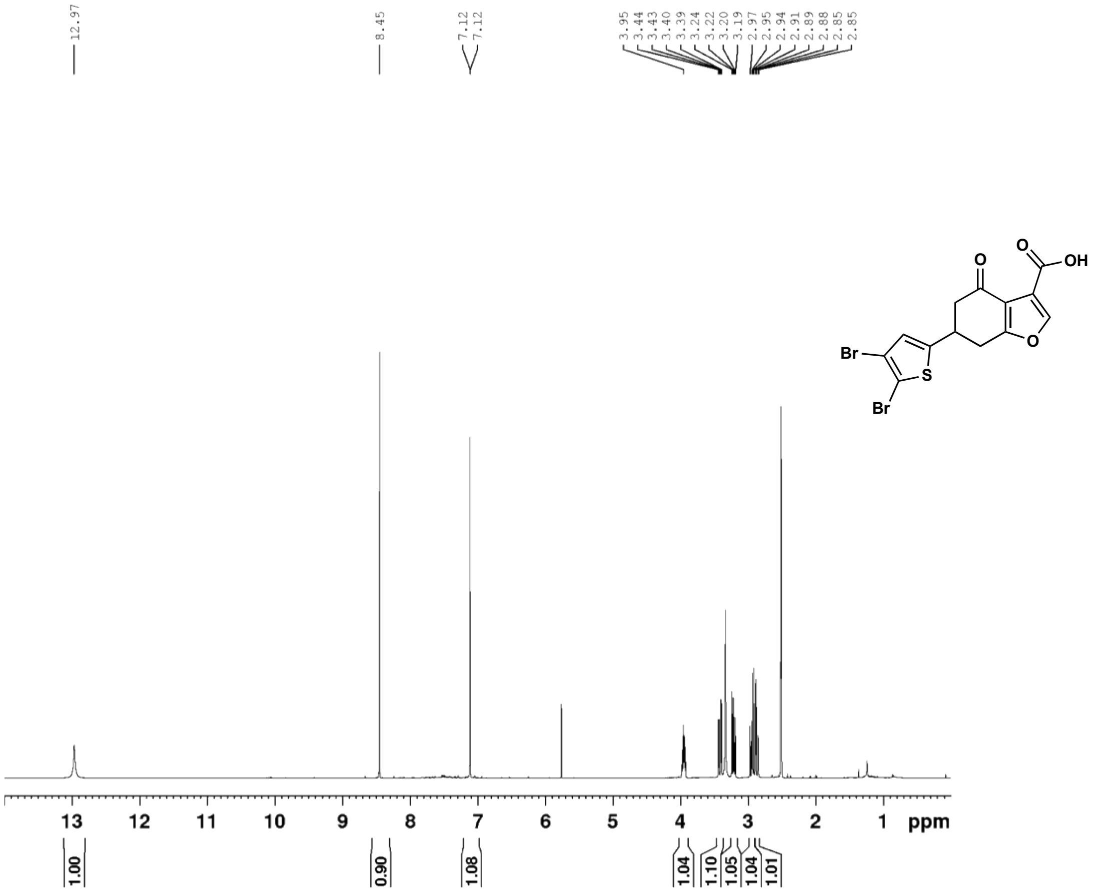

6-(4,5-dibromothiophen-2-yl)-4-oxo-4,5,6,7-tetrahydrobenzofuran-3-carboxylic acid (**34**) ^{13}C NMR (126 MHz, d_6-DMSO)

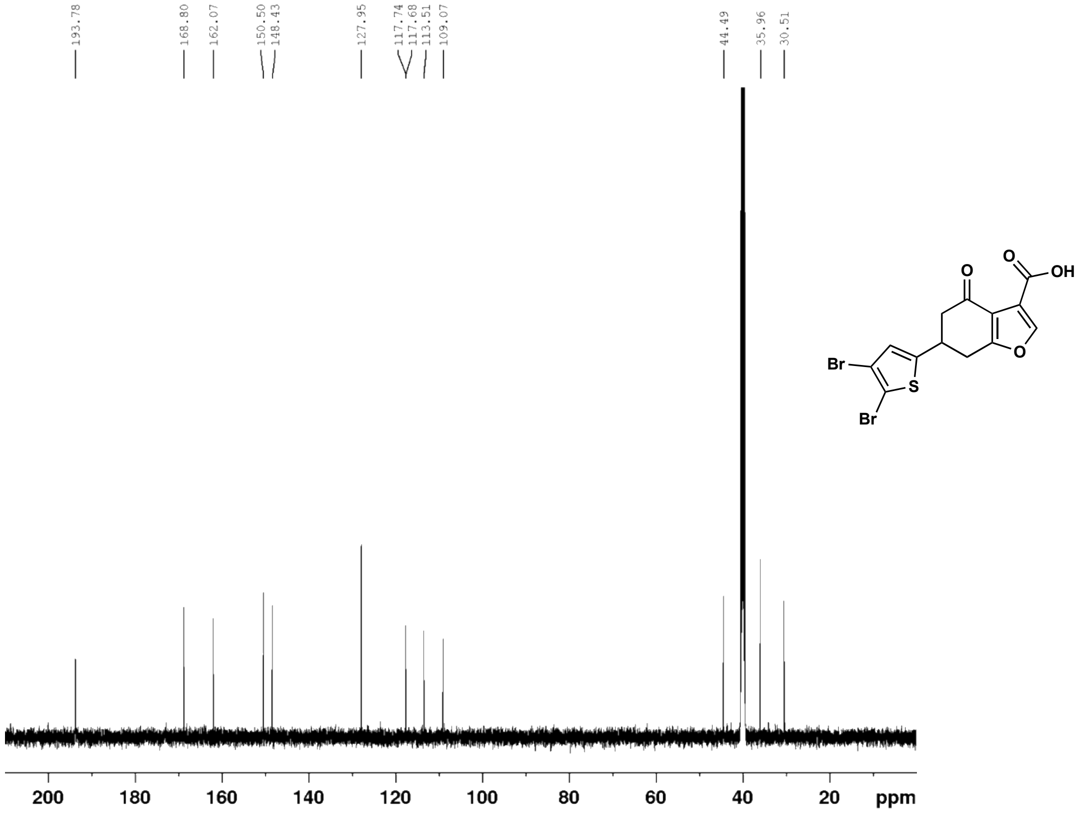

ethyl 2-(1-oxo-1,2,3,4-tetrahydronaphthalen-2-yl)acetate (**36**) ^{1}H NMR (500 MHz, $CDCl_3$)

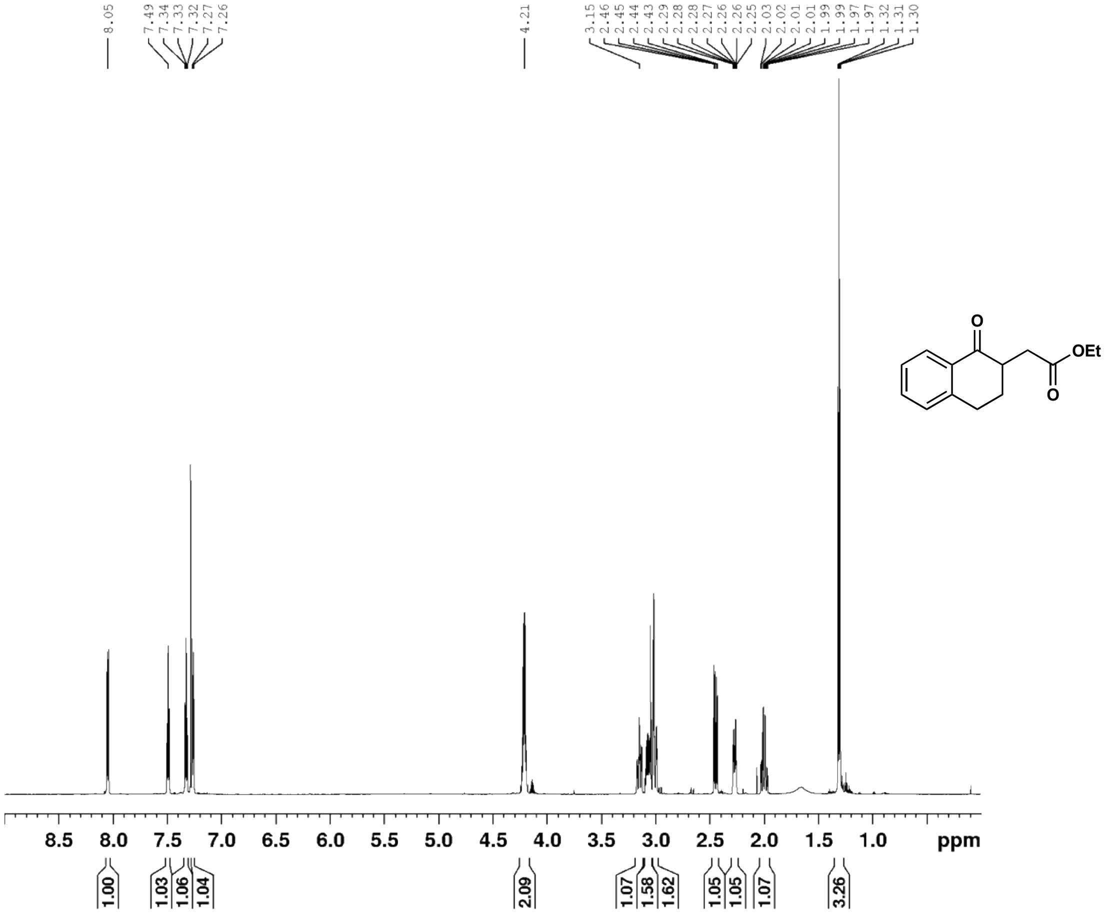

ethyl 2-(1-oxo-1,2,3,4-tetrahydronaphthalen-2-yl)acetate (**36**) ^{13}C NMR (126 MHz, $CDCl_3$)

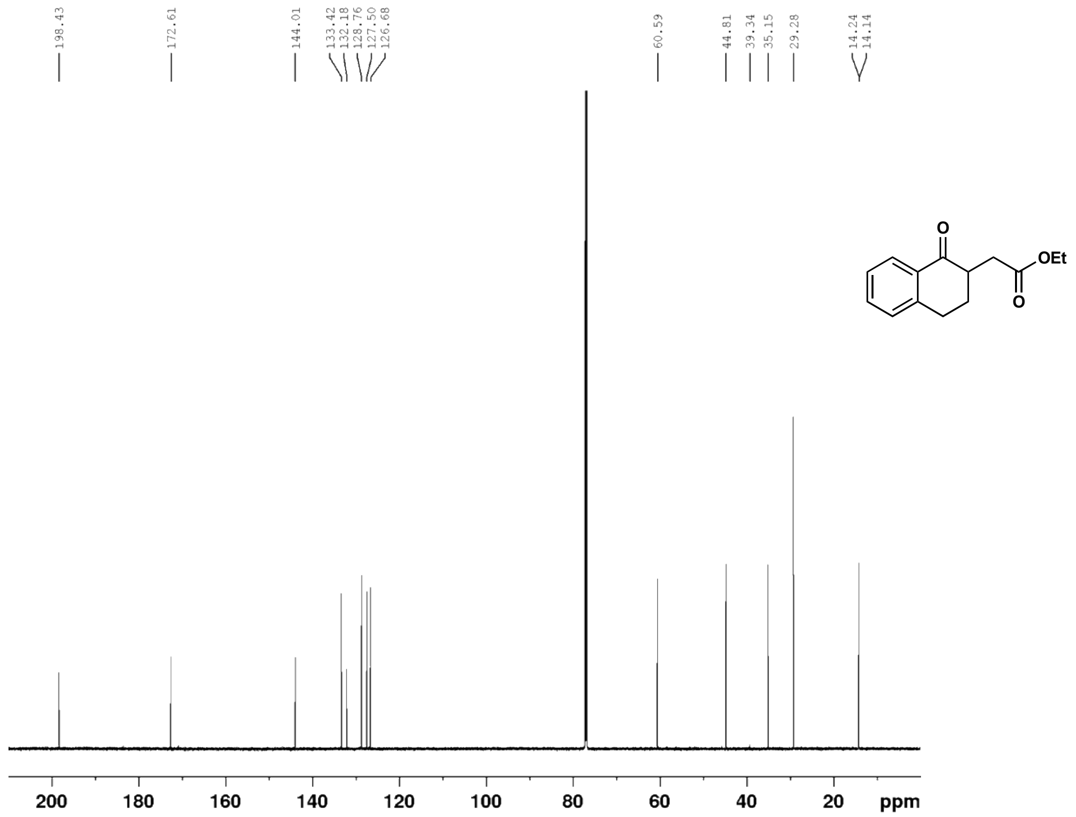

2-(1-oxo-1,2,3,4-tetrahydronaphthalen-2-yl)acetic acid (**37**) ^{1}H NMR (500 MHz, $CDCl_3$)

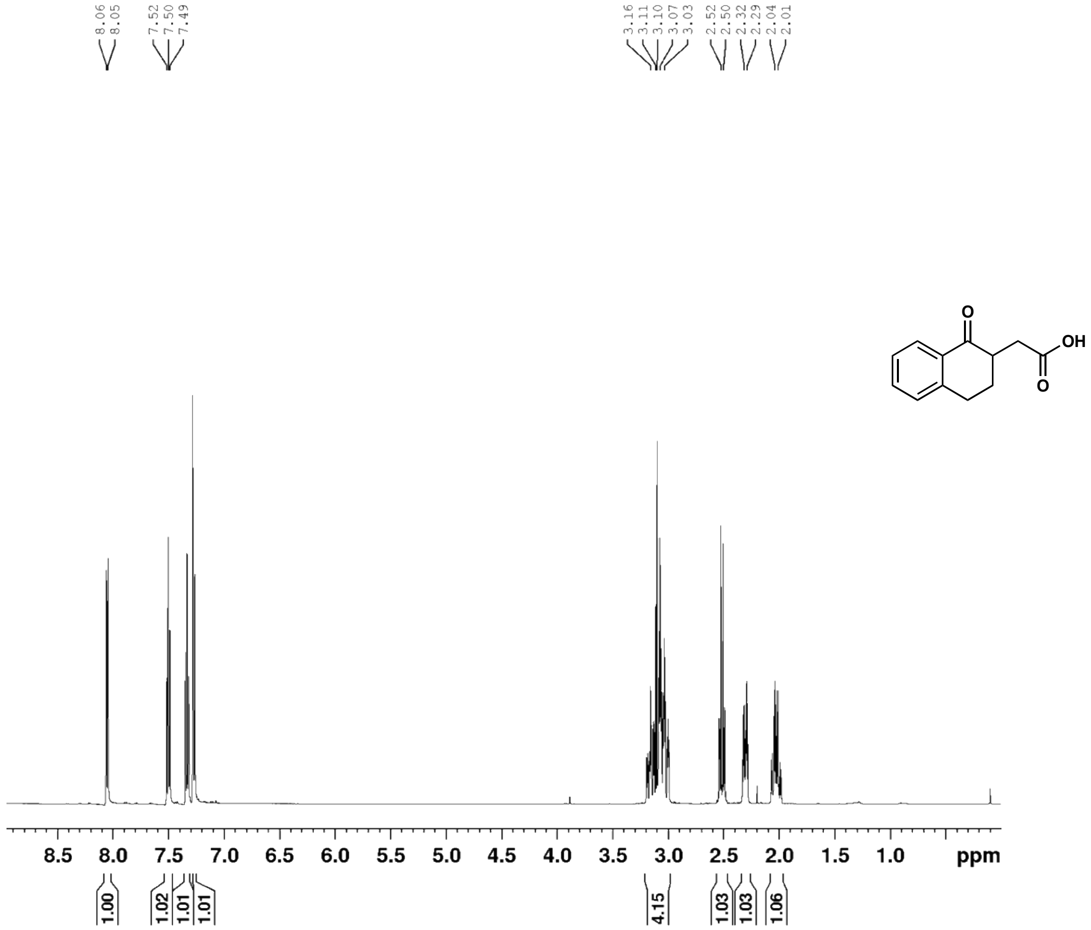

2-(1-oxo-1,2,3,4-tetrahydronaphthalen-2-yl)acetic acid (**37**) ^{13}C NMR (126 MHz, $CDCl_3$)

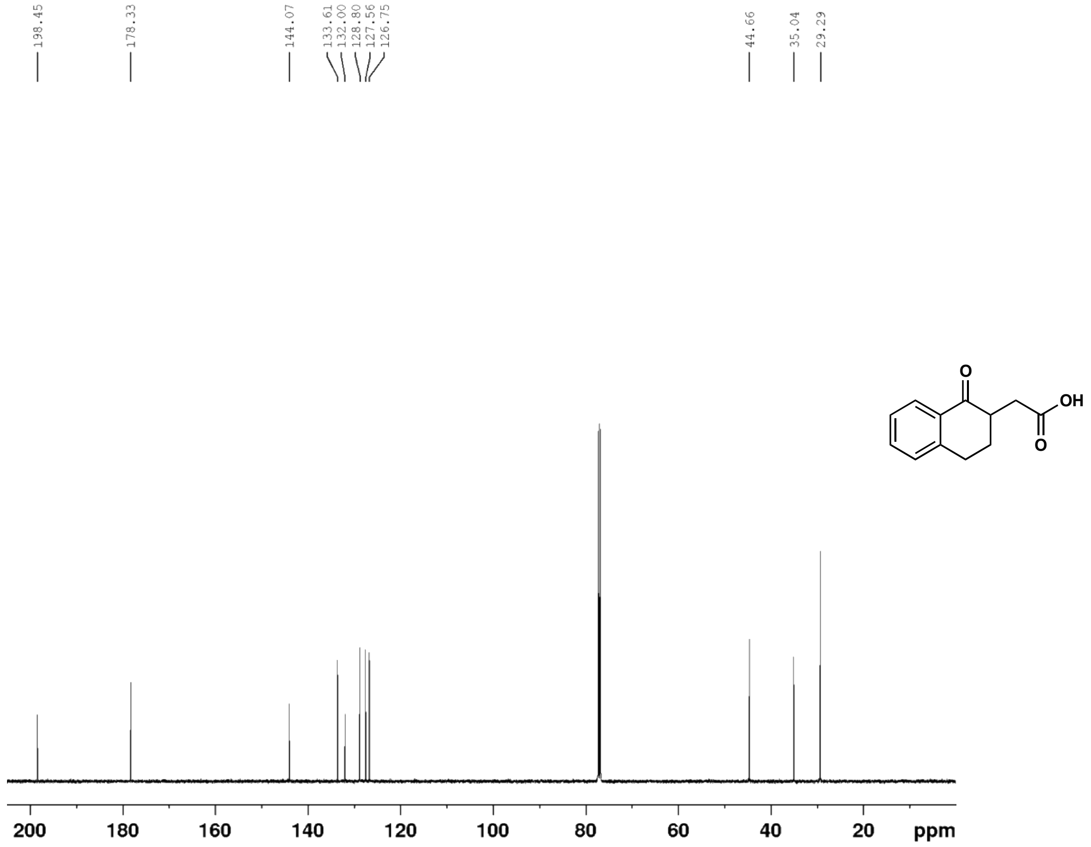

S3 Compounds HPLC Traces

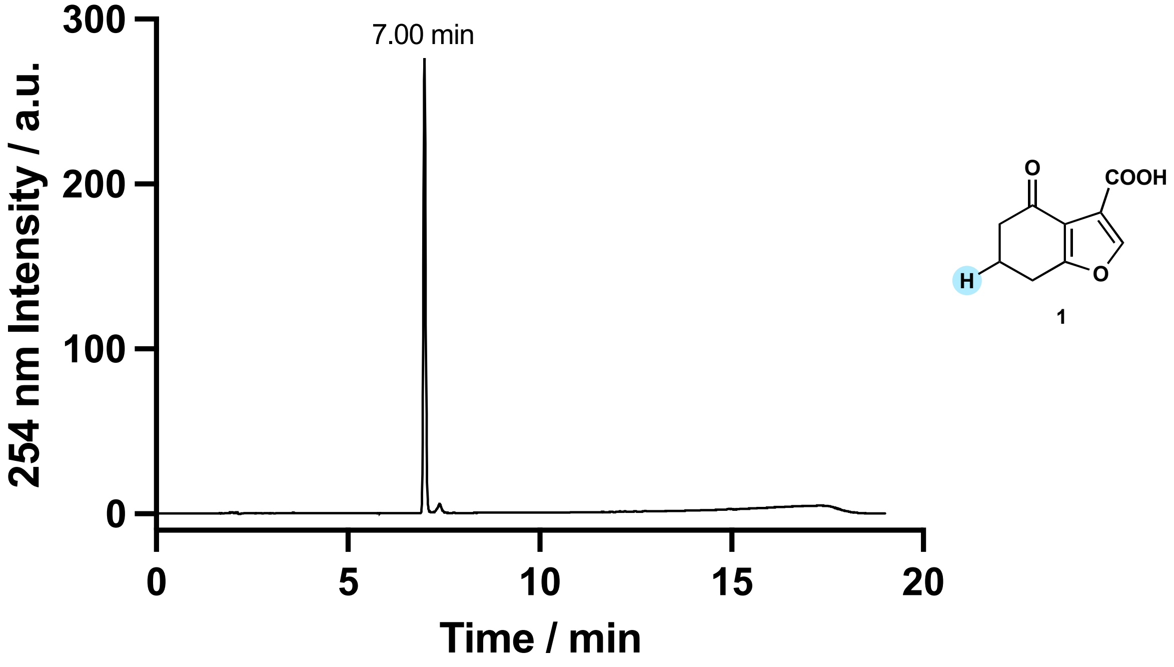

Figure S3 -Compound **1**, HPLC 5–95 % MeCN in H_2O. Purity based on integration is 99 %.

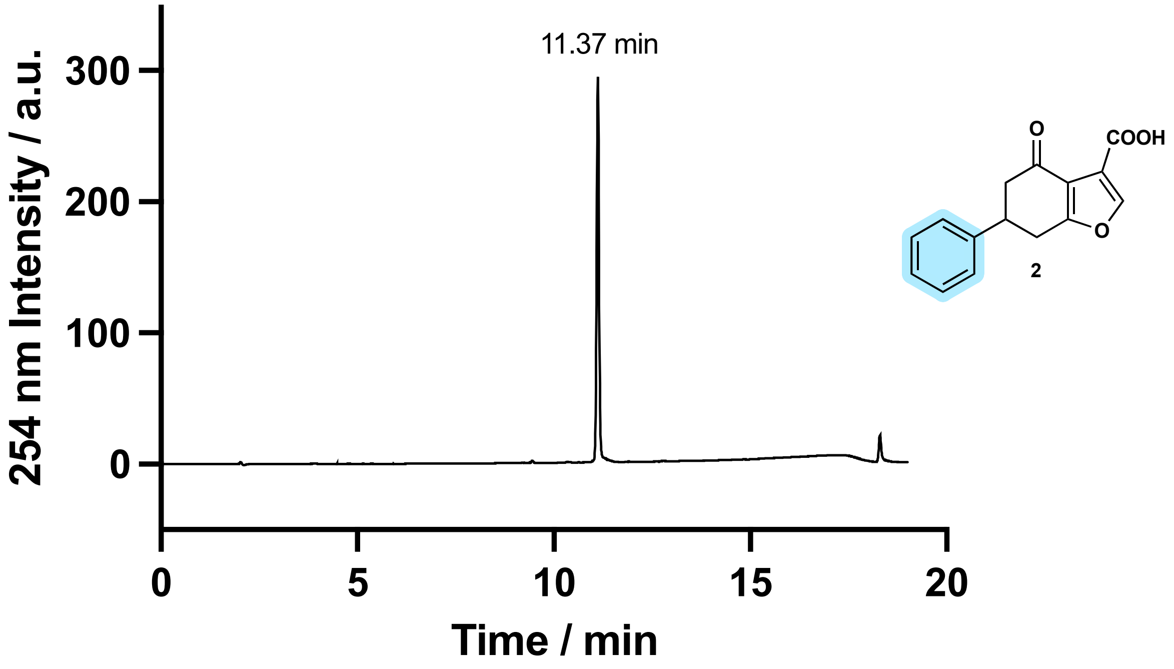

Figure S4 - Compound **2**, HPLC 5–95 % MeCN in H_2O. Purity based on integration is 96 %.

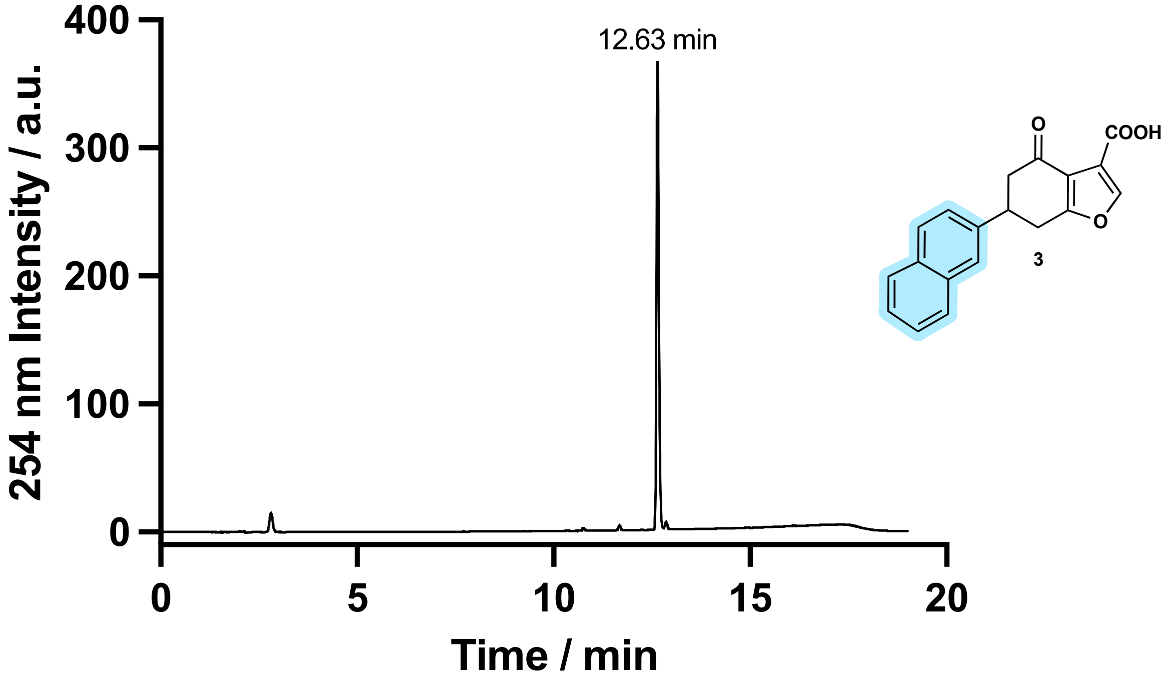

Figure S5 - Compound **3**, HPLC 5–95 % MeCN in H_2O. Purity based on integration is 98 %.

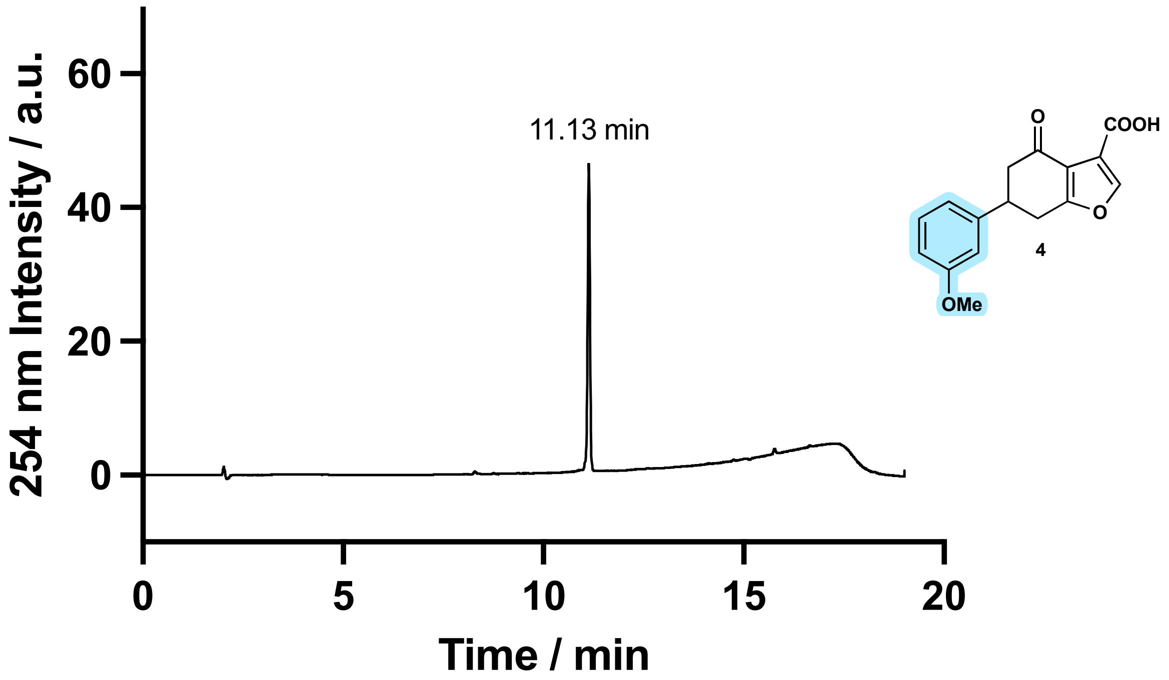

Figure S6 - Compound **4**, HPLC 5–95 % MeCN in H_2O. Purity based on integration is 98 %.

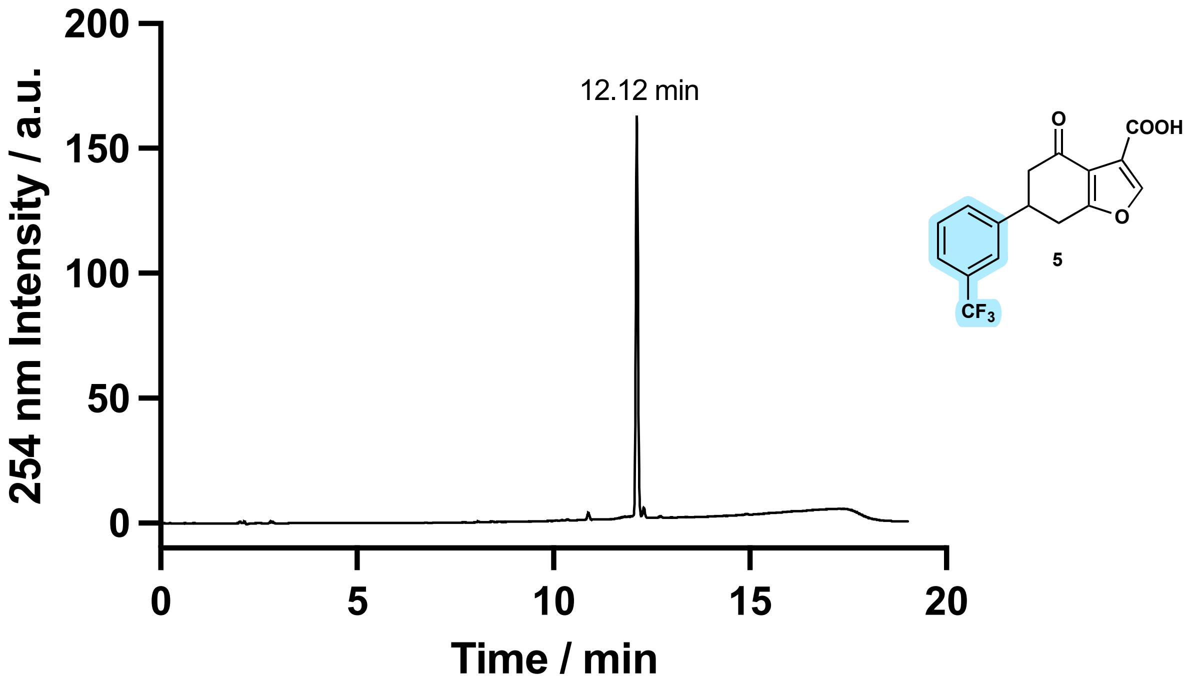

Figure S7 - Compound **5**, HPLC 5–95 % MeCN in H_2O. Purity based on integration is 96 %.

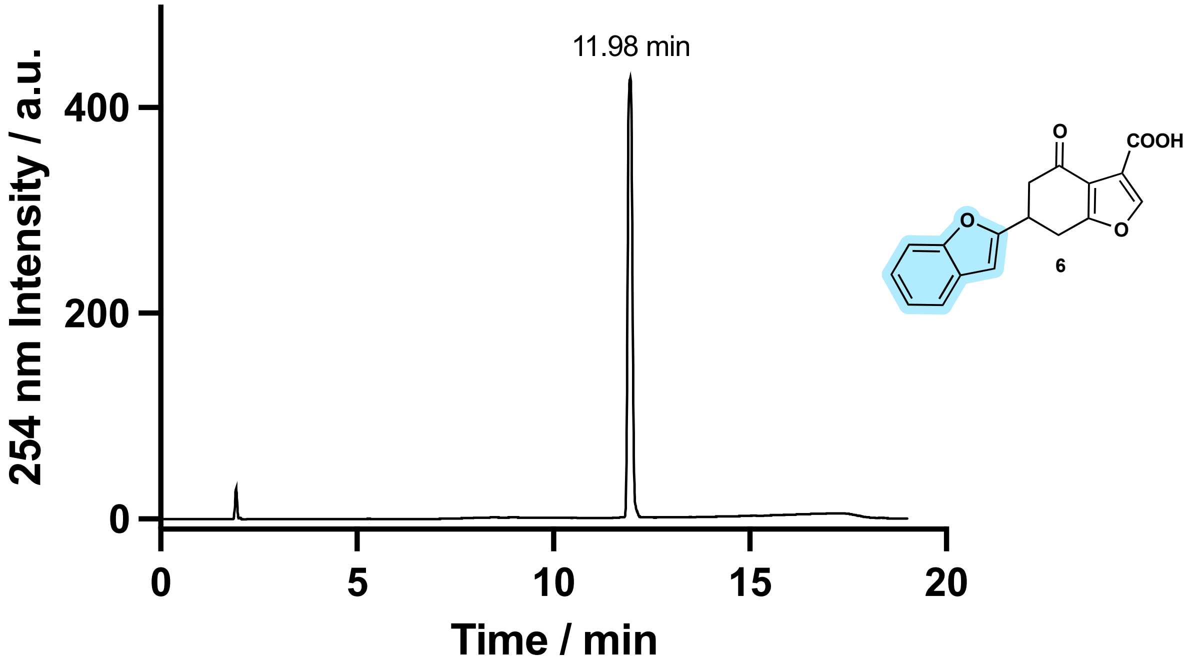

Figure S8 - Compound **6**, HPLC 5–95 % MeCN in H_2O. Purity based on integration is 97 %.

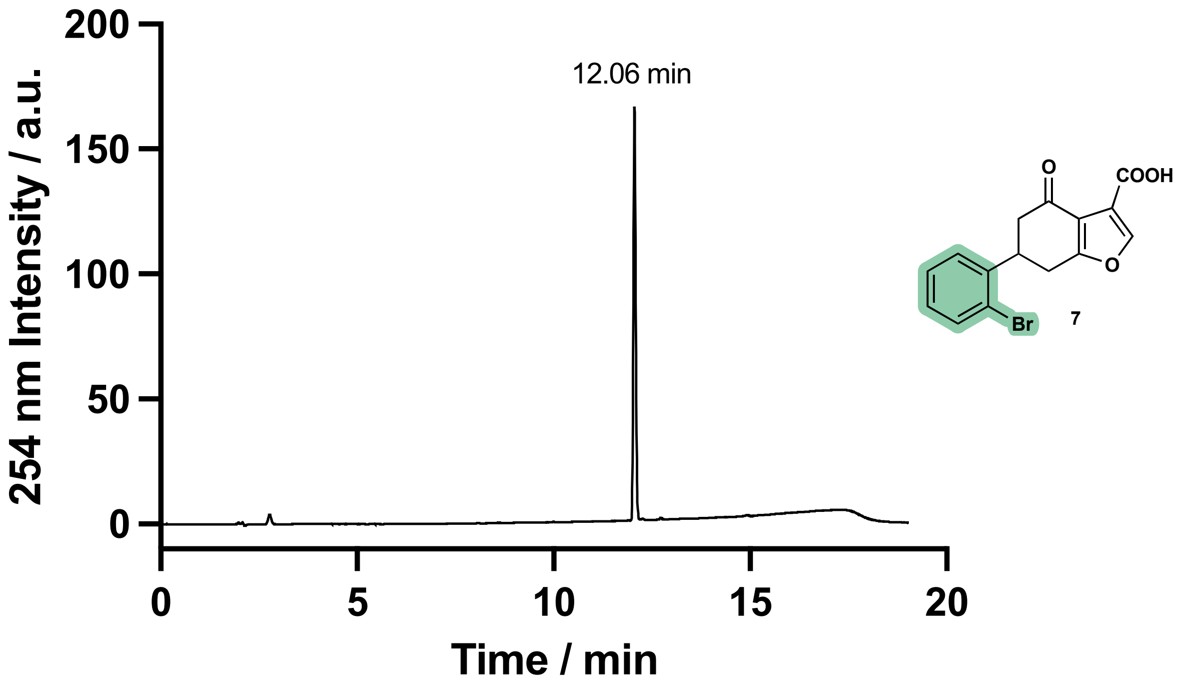

Figure S9 - Compound **7**, HPLC 5–95 % MeCN in H_2O. Purity based on integration is 97 %.

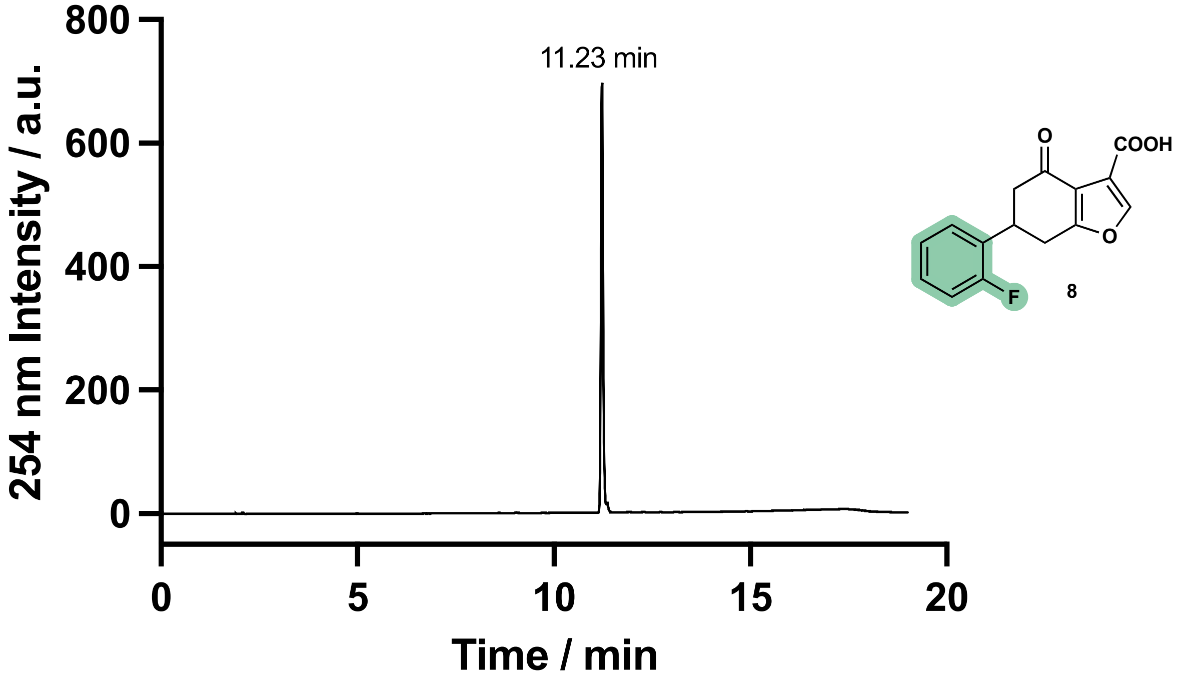

Figure S10 - Compound **8**, HPLC 5–95 % MeCN in H_2O. Purity based on integration is 99 %.

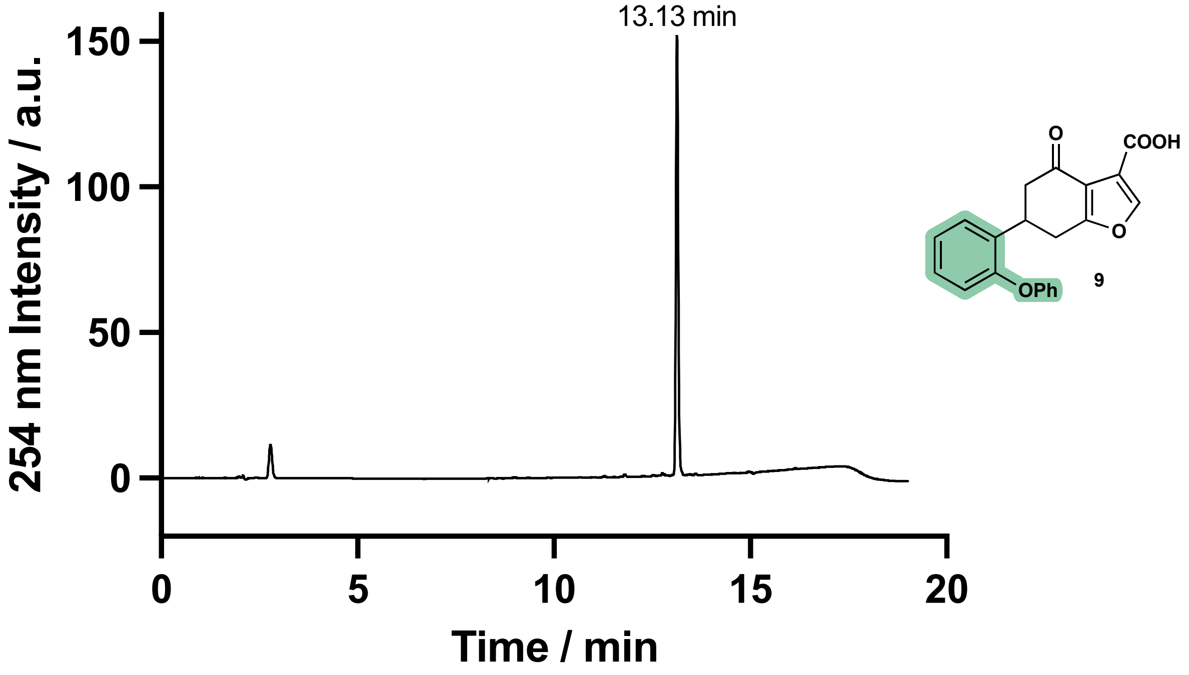

Figure S11 - Compound **9**, HPLC 5–95 % MeCN in H_2O. Purity based on integration is 91 %.

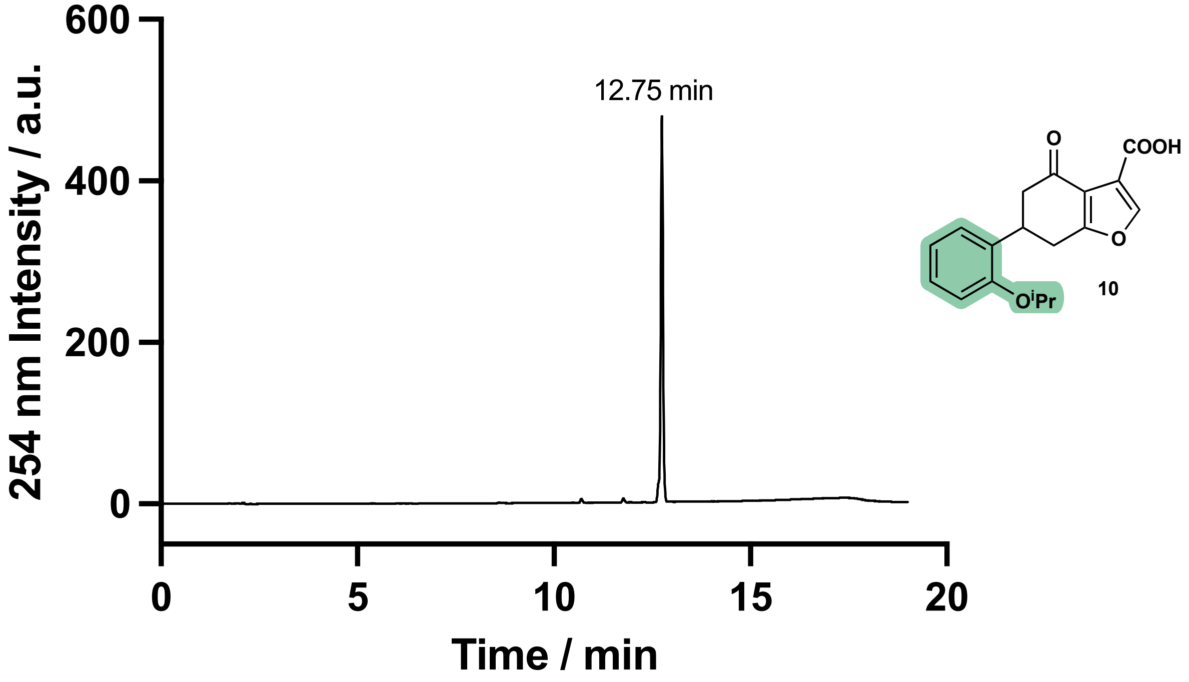

Figure S12 - Compound **10**, HPLC 5–95 % MeCN in H_2O. Purity based on integration is 97 %.

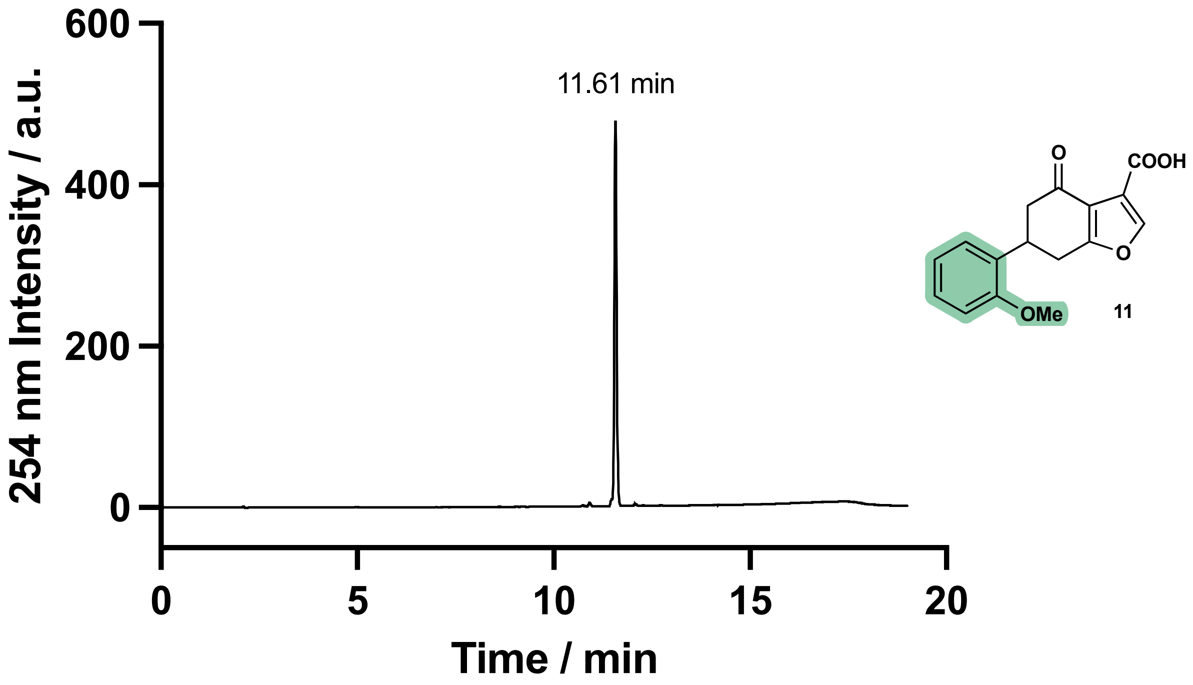

Figure S13 - Compound **11**, HPLC 5–95 % MeCN in H_2O. Purity based on integration is 97 %.

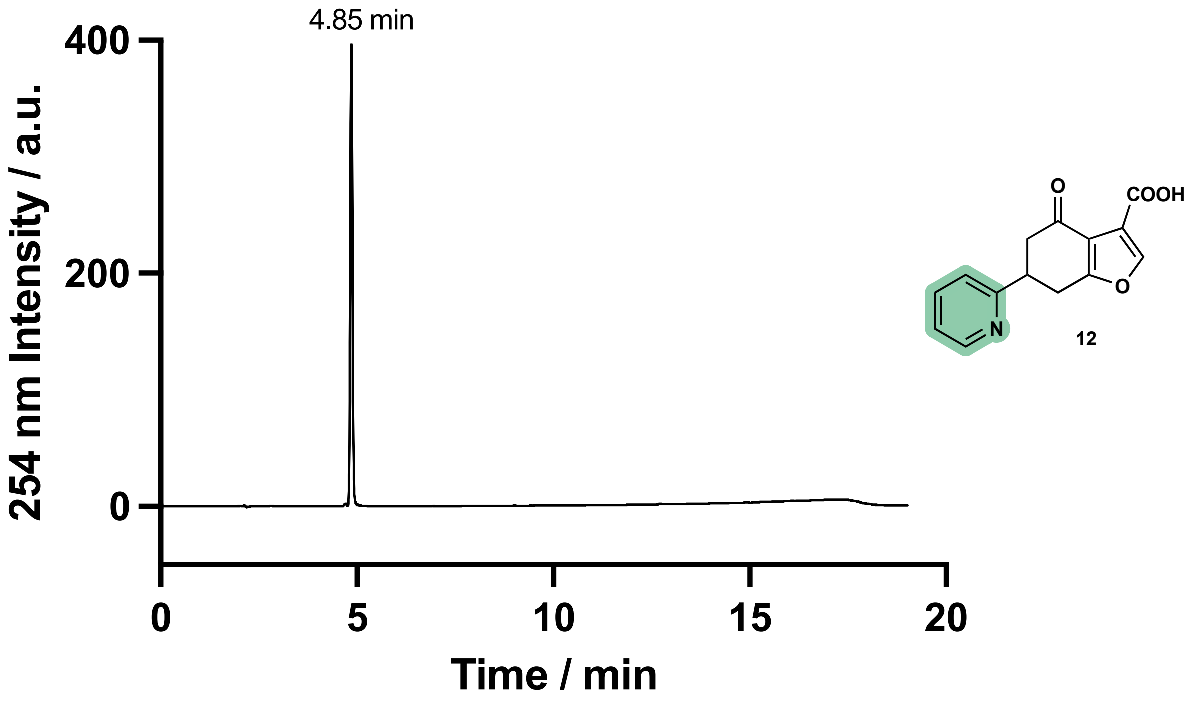

Figure S14 - Compound **12**, HPLC 5–95 % MeCN in H_2O. Purity based on integration is 100 %.

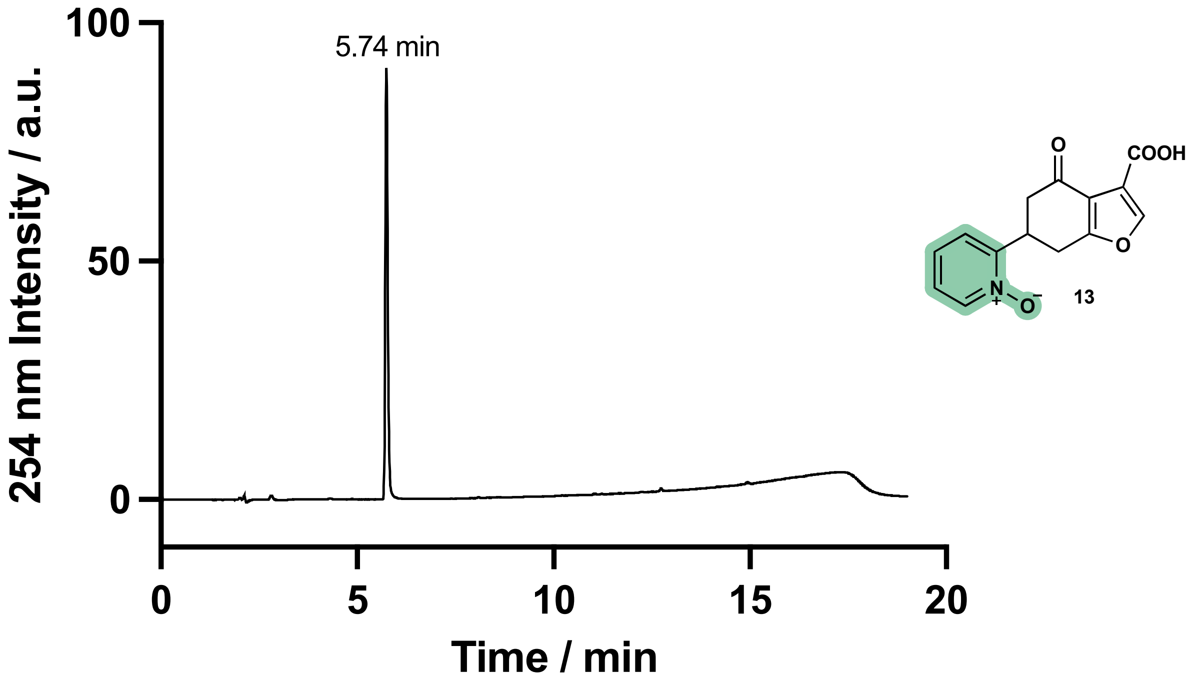

Figure S15 - Compound **13**, HPLC 5–95 % MeCN in H_2O. Purity based on integration is 100 %.

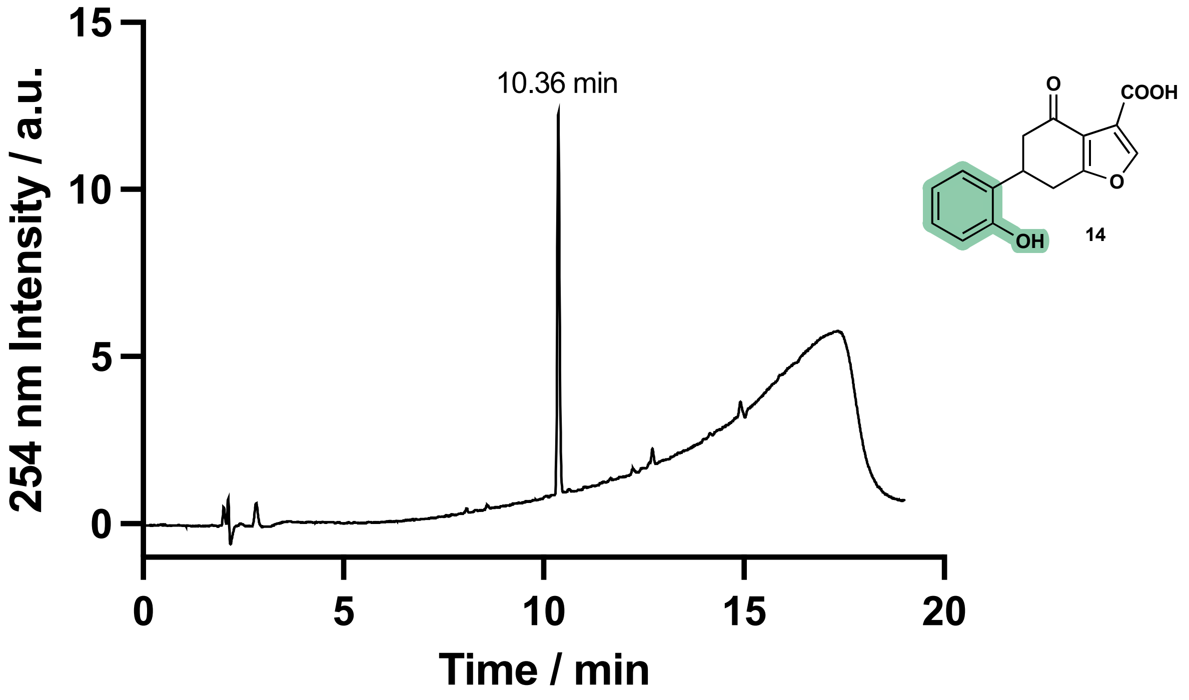

Figure S16 - Compound **14**, HPLC 5–95 % MeCN in H_2O. Purity based on integration is 95 %.

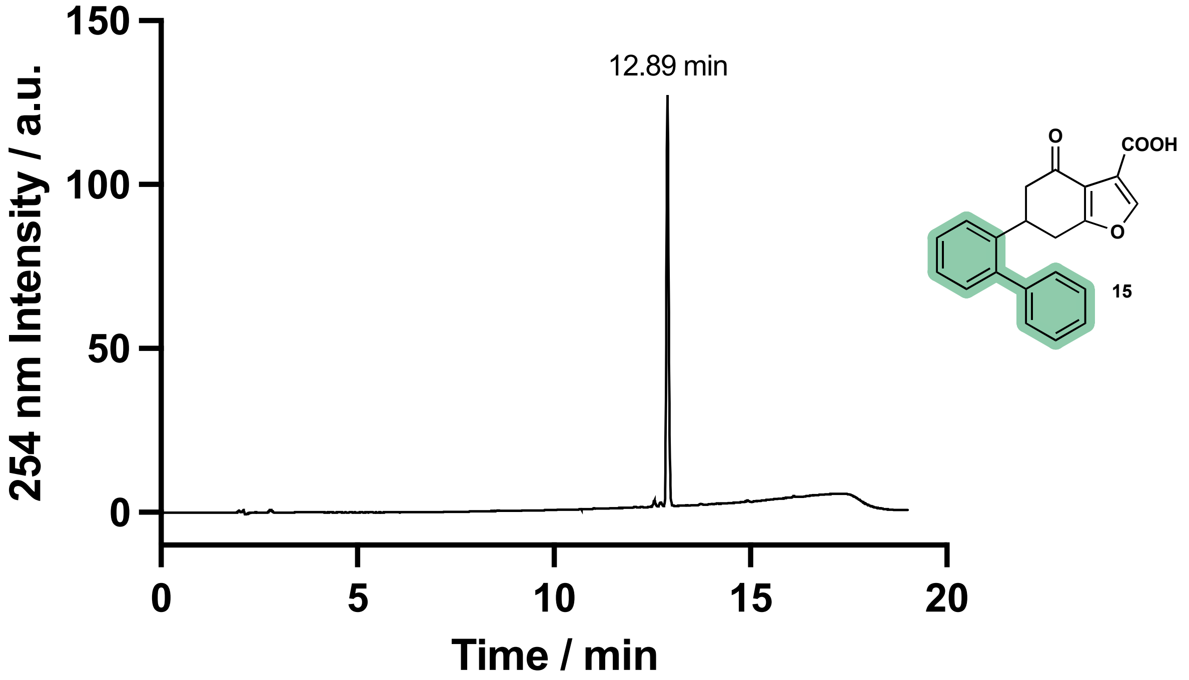

Figure S17 - Compound **15**, HPLC 5–95 % MeCN in H_2O. Purity based on integration is 97 %.

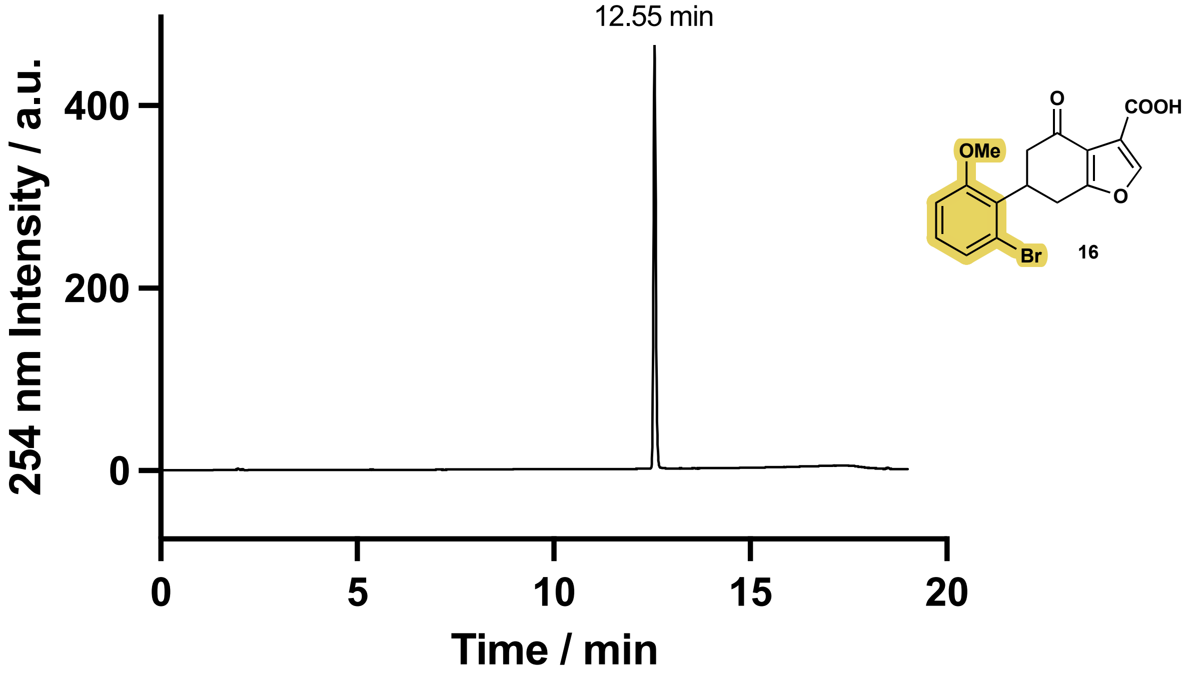

Figure S18 - Compound **16**, HPLC 5–95 % MeCN in H_2O. Purity based on integration is 100 %.

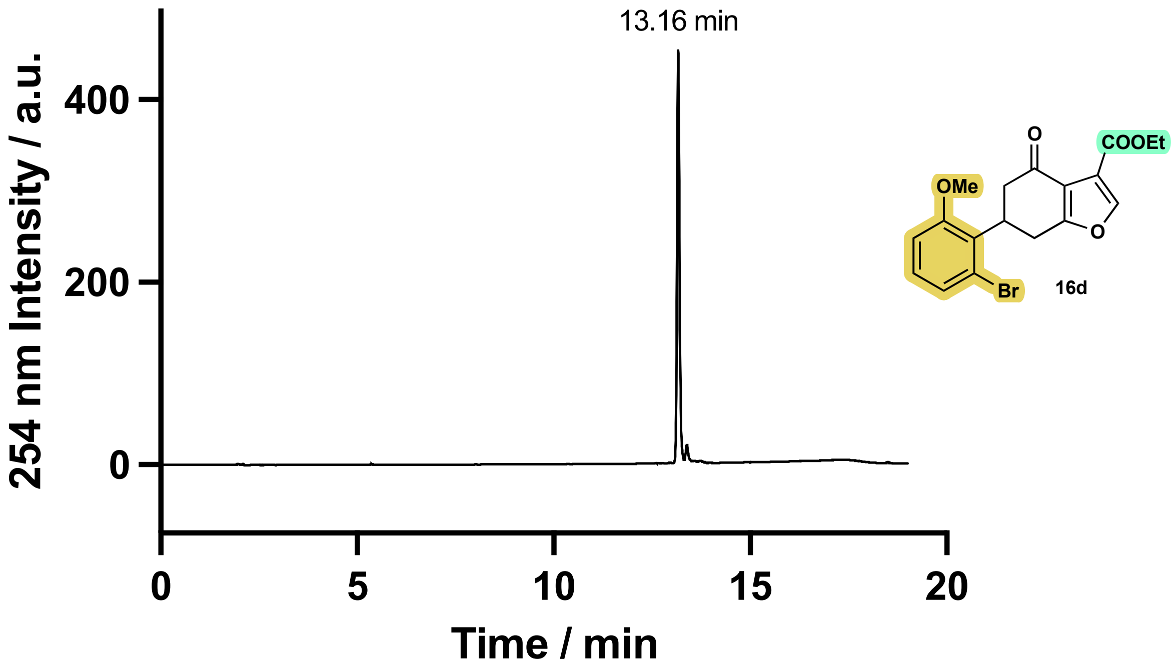

Figure S19 - Compound **16d**, HPLC 5–95 % MeCN in H_2O. Purity based on integration is 95 %.

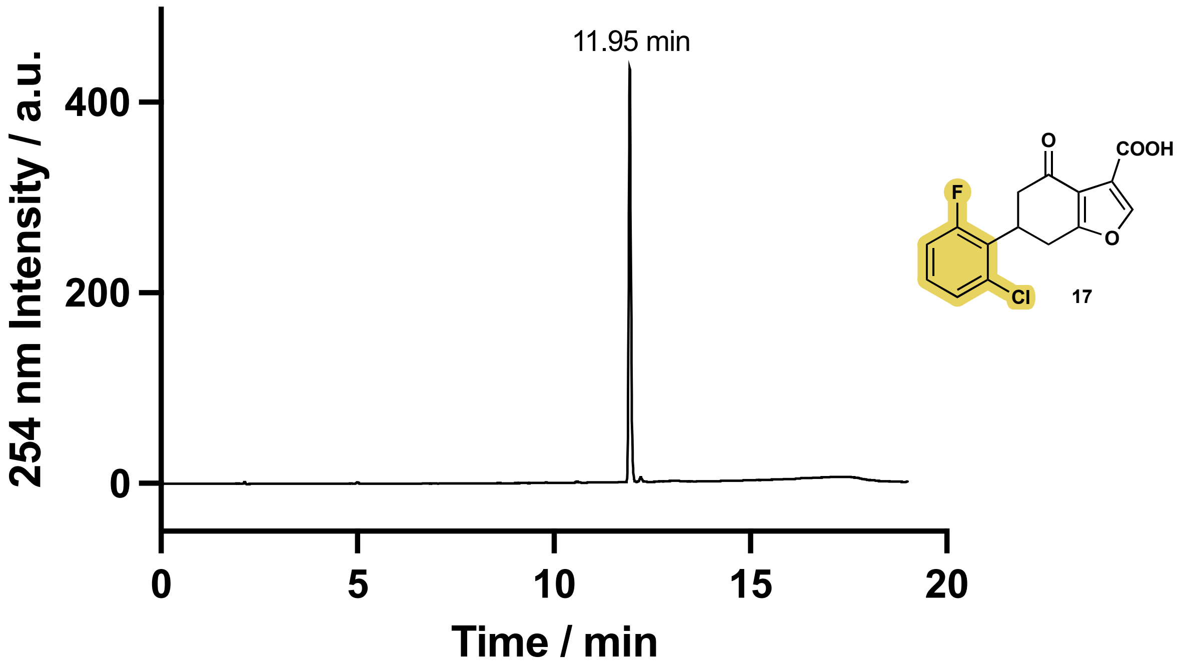

Figure S20 - Compound **17**, HPLC 5–95 % MeCN in H_2O. Purity based on integration is 99 %.

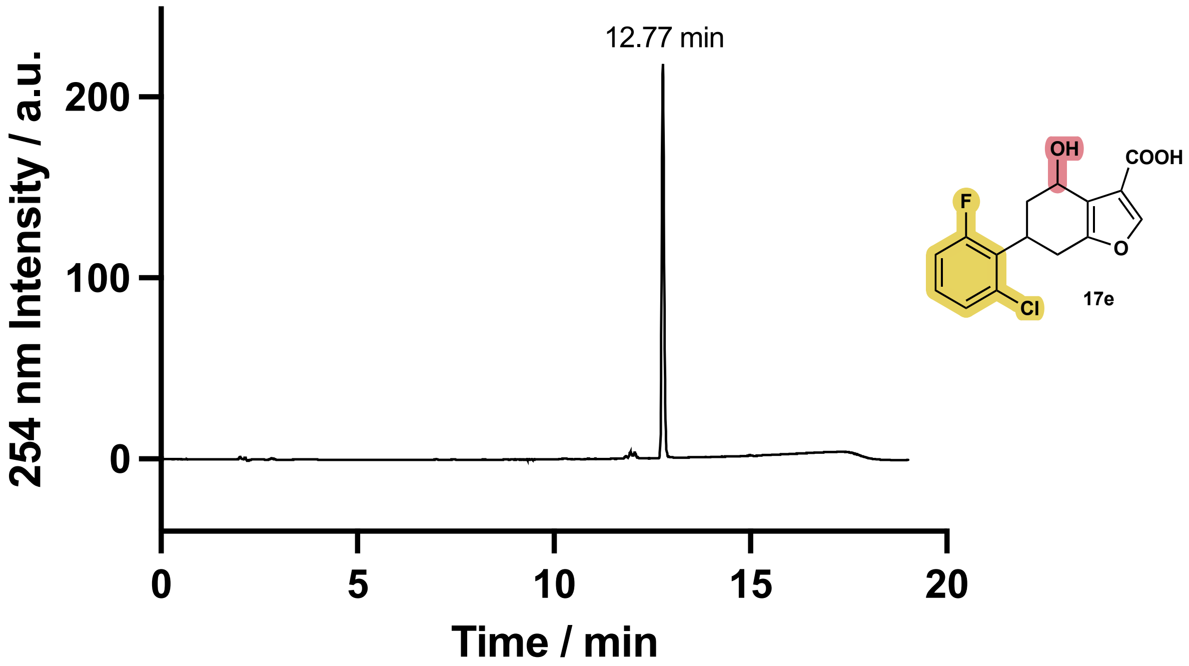

Figure S21 - Compound **17e**, HPLC 5–95 % MeCN in H_2O. Purity based on integration is 96 %.

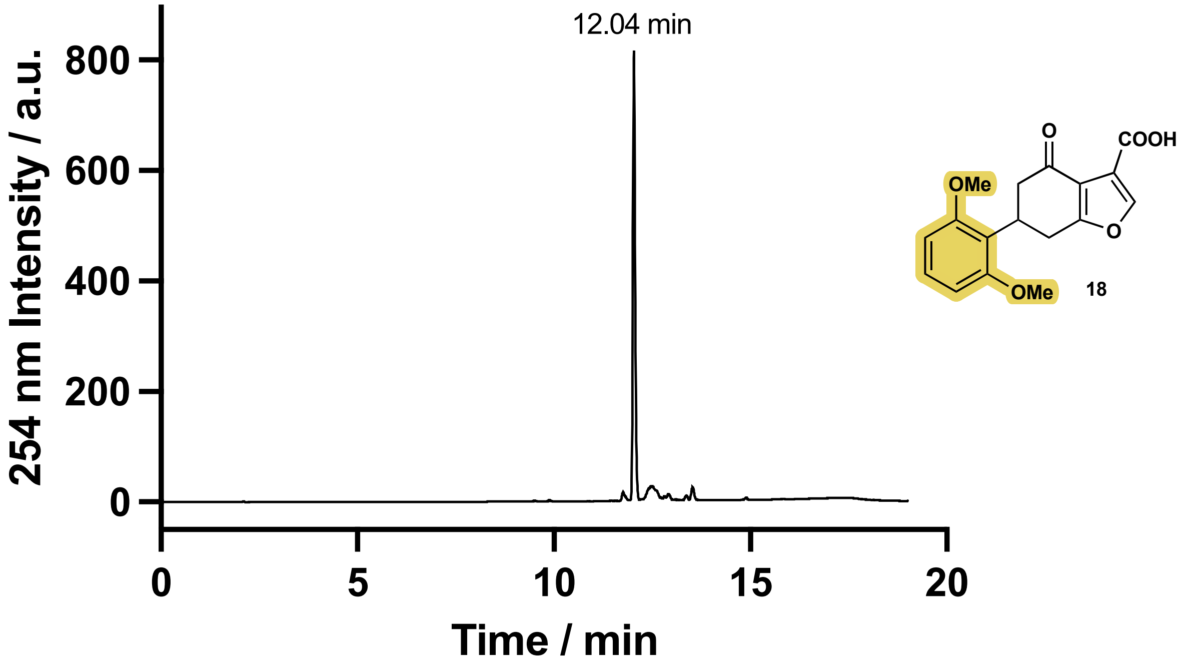

Figure S22 - Compound **18**, HPLC 5–95 % MeCN in H_2O. Purity based on integration is 95 %.

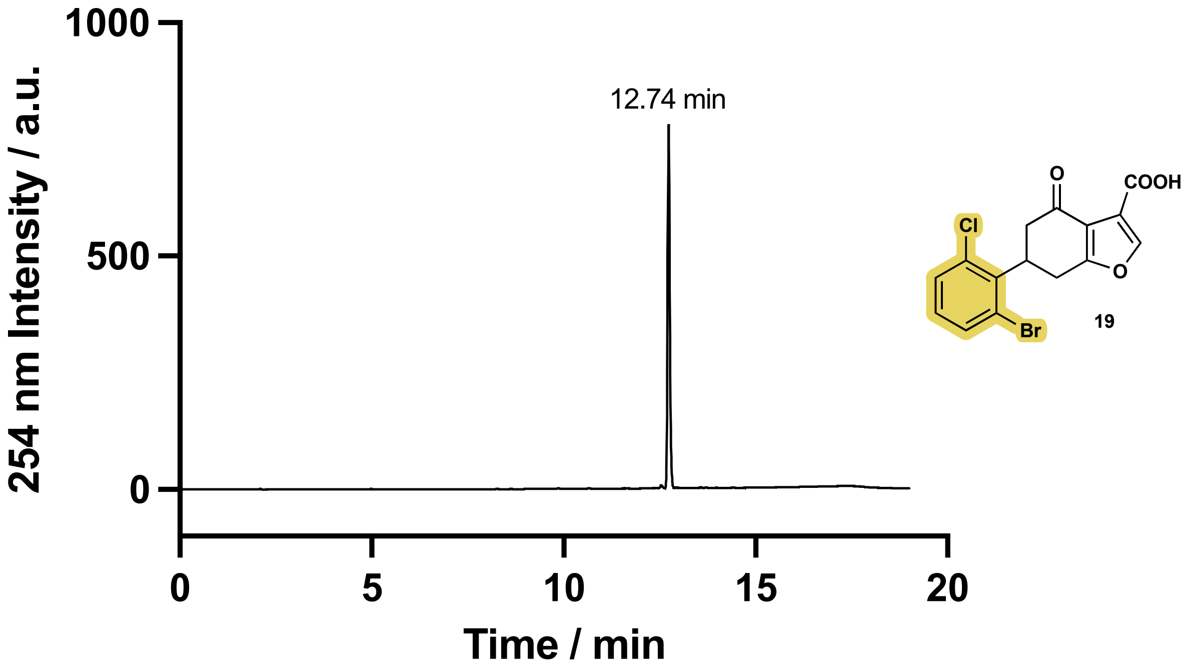

Figure S23 - Compound **19**, HPLC 5–95 % MeCN in H_2O. Purity based on integration is 98 %.

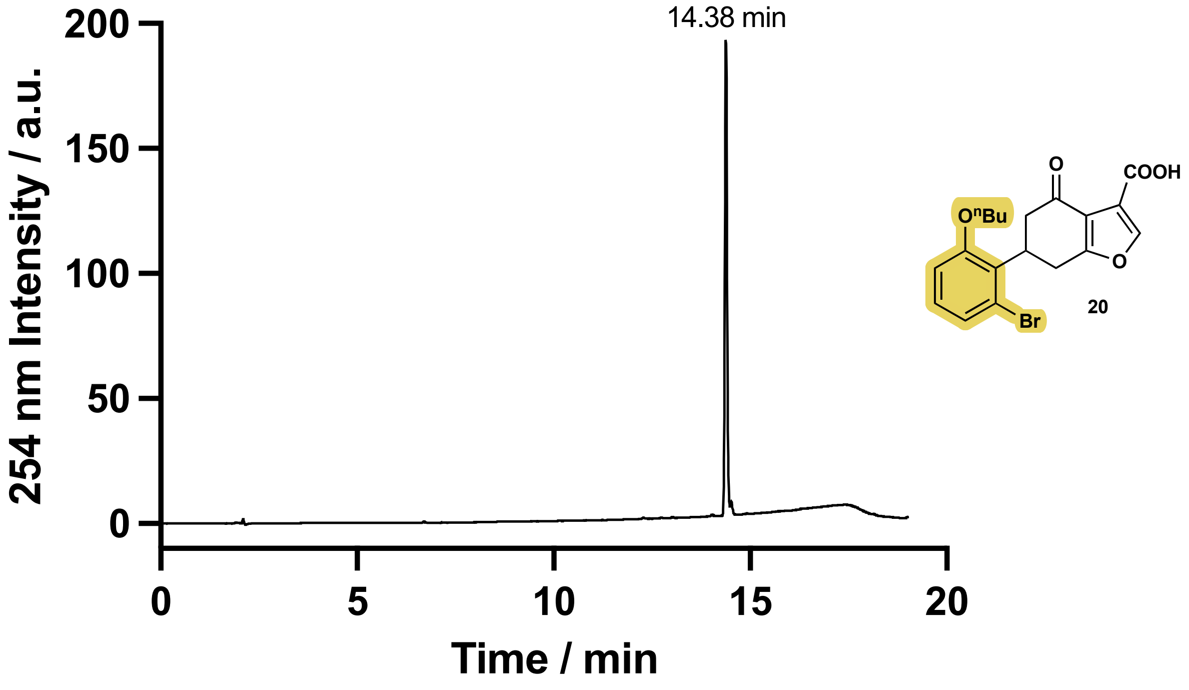

Figure S24 - Compound **20**, HPLC 5–95 % MeCN in H_2O. Purity based on integration is 96 %.

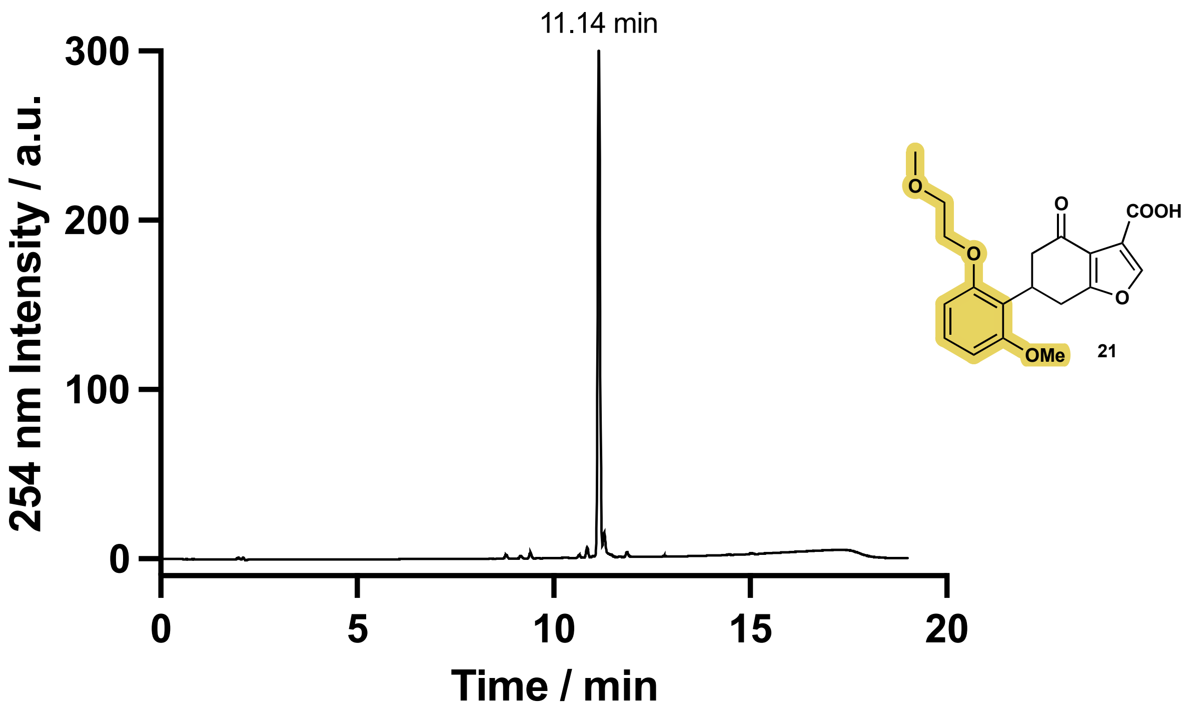

Figure S25 - Compound **21**, HPLC 5–95 % MeCN in H_2O. Purity based on integration is 94 %.

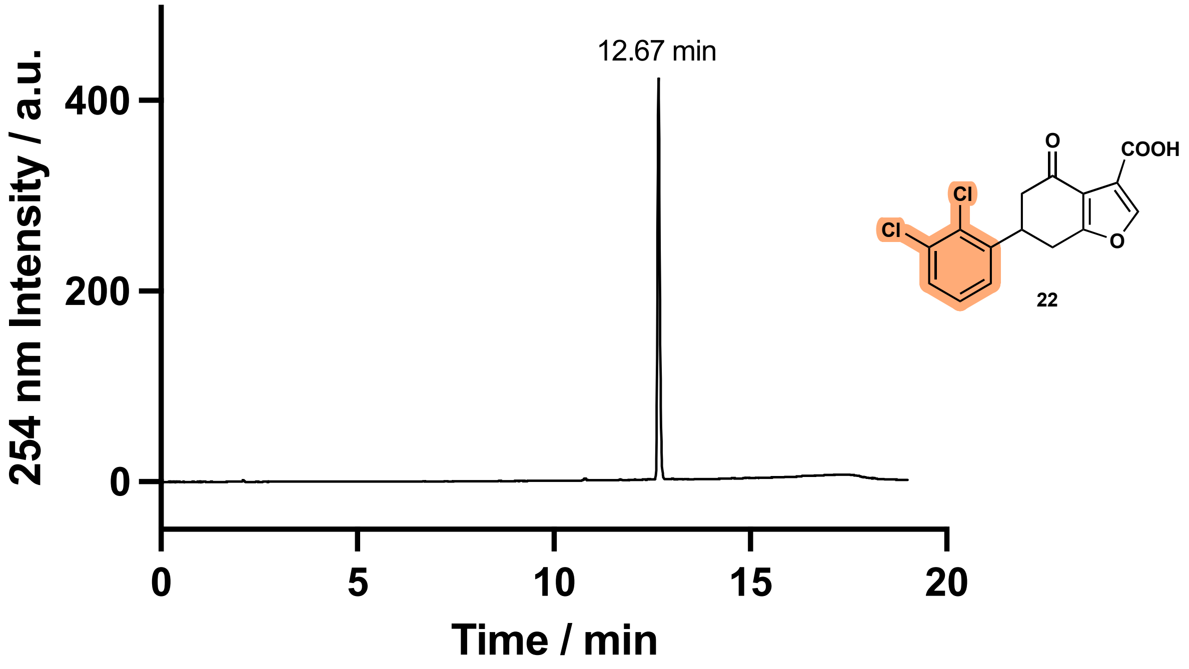

Figure S26 - Compound **22**, HPLC 5–95 % MeCN in H_2O. Purity based on integration is 100 %.

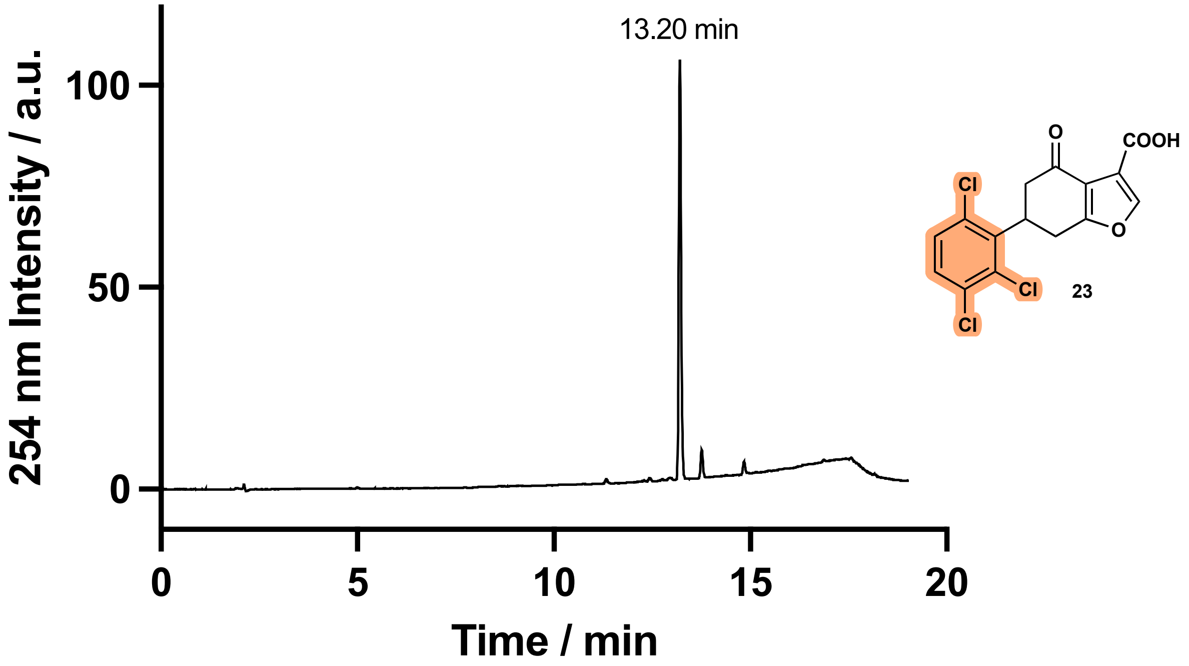

Figure S27 - Compound **23**, HPLC 5–95 % MeCN in H_2O. Purity based on integration is 95 %.

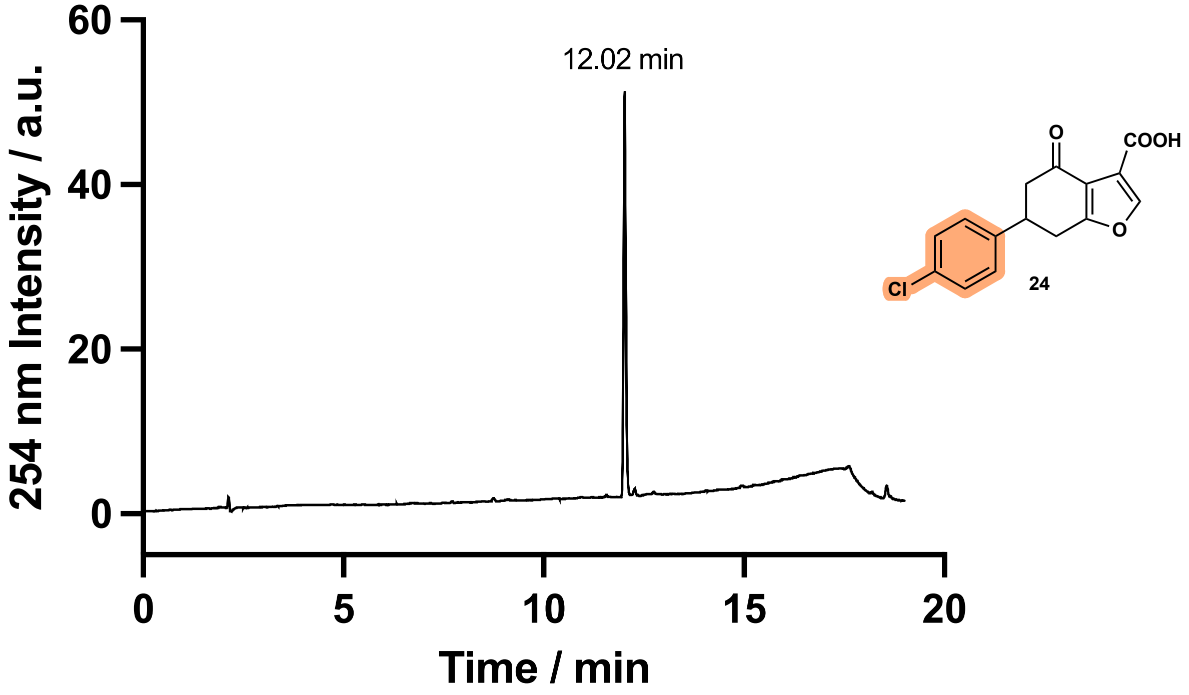

Figure S28 - Compound **24**, HPLC 5–95 % MeCN in H_2O. Purity based on integration is 99 %.

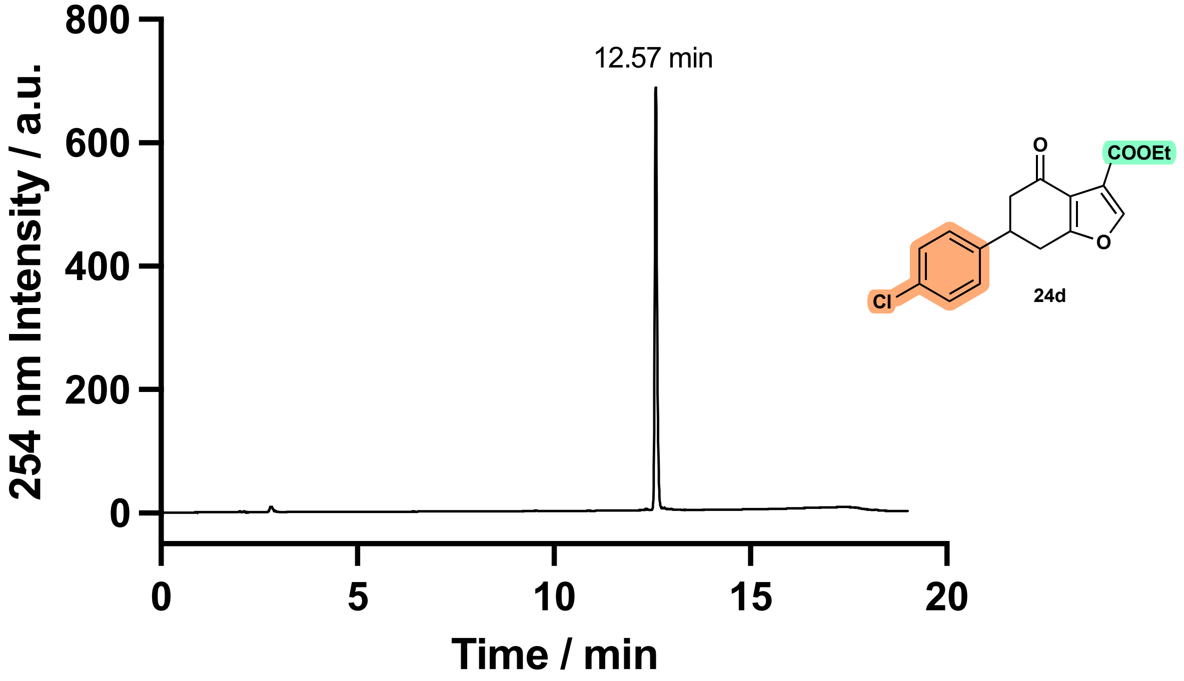

Figure S29 - Compound **24d**, HPLC 5–95 % MeCN in H_2O. Purity based on integration is 99 %.

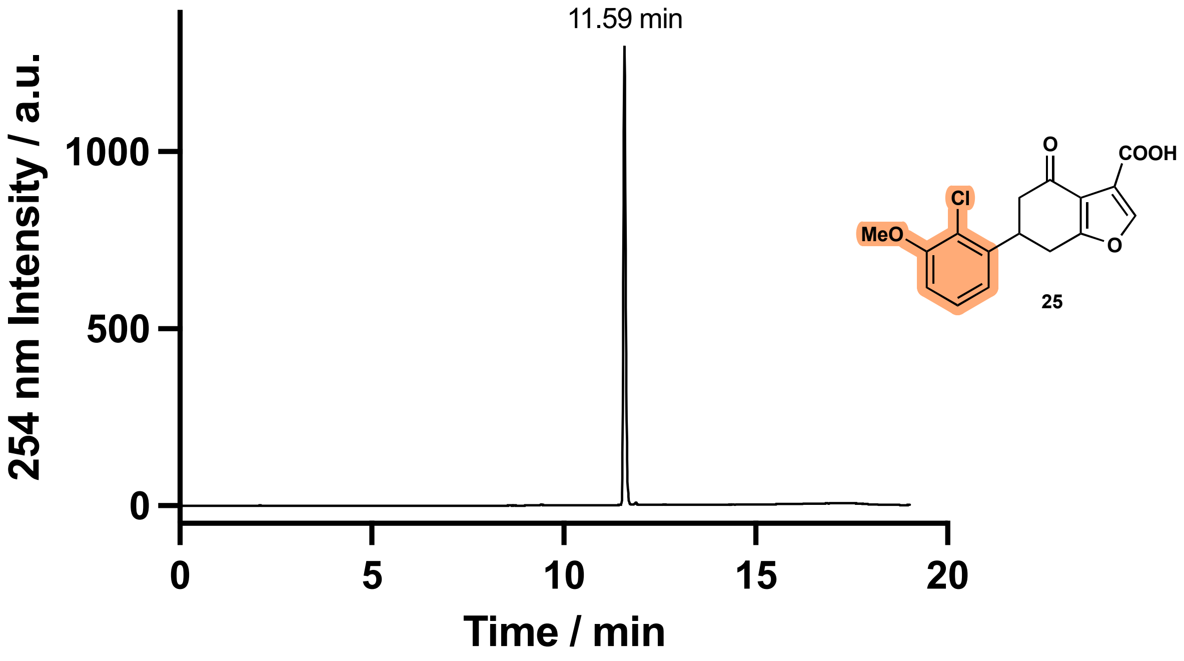

Figure S30 - Compound **25**, HPLC 5–95 % MeCN in H_2O. Purity based on integration is 98 %.

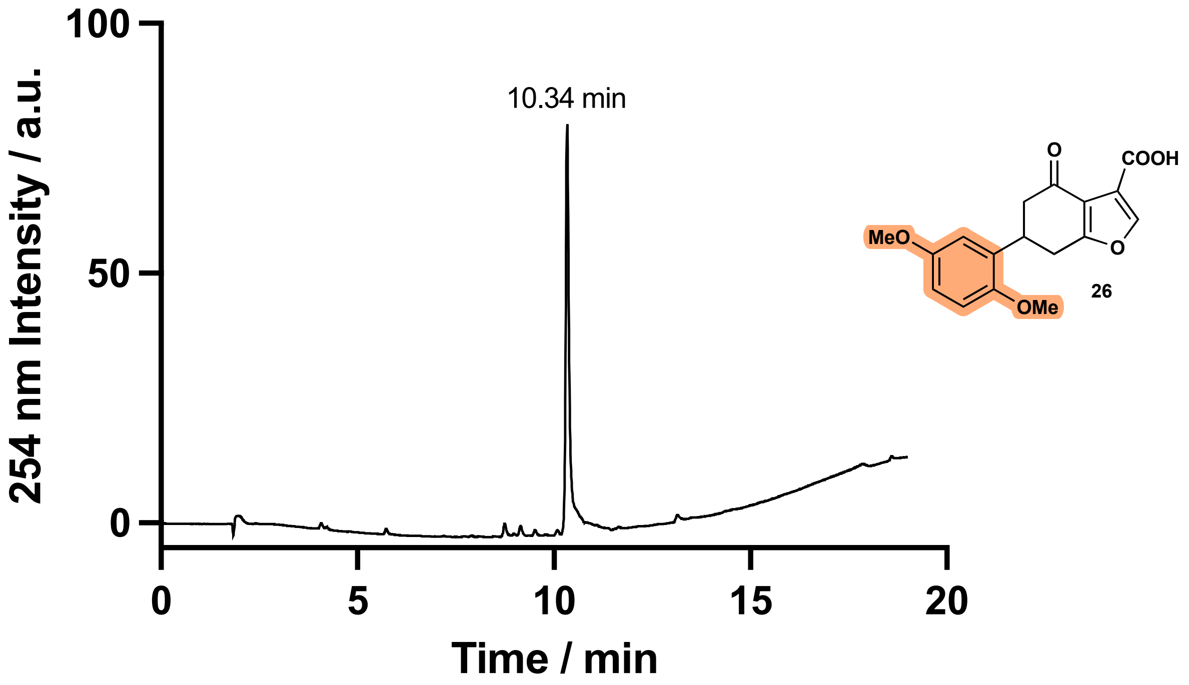

Figure S31 - Compound **26**, HPLC 5–95 % MeCN in H_2O. Purity based on integration is 94 %.

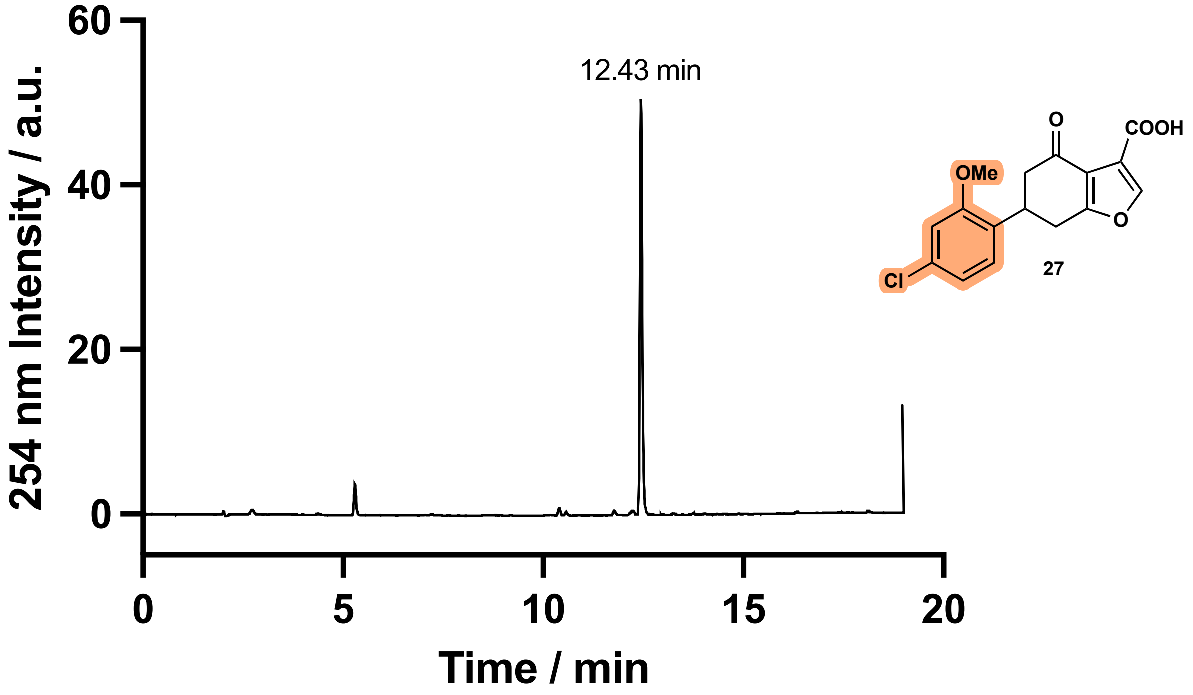

Figure S32 - Compound **27**, HPLC 5–95 % MeCN in H_2O. Purity based on integration is 93 %.

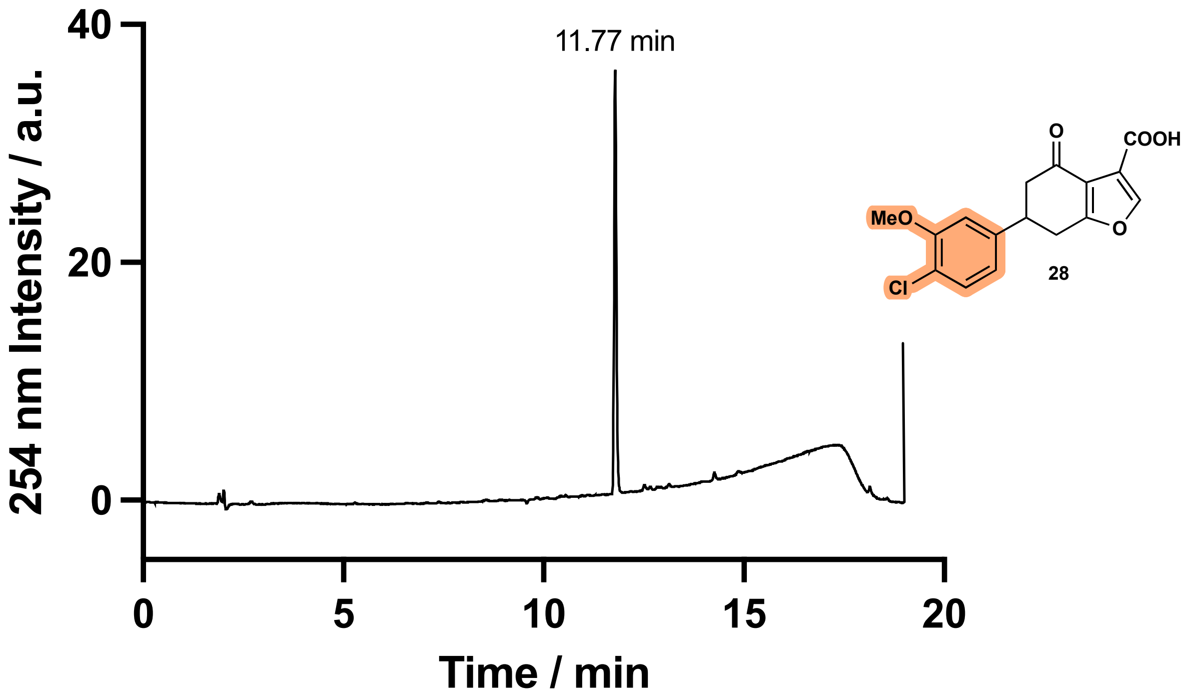

Figure S33 - Compound **28**, HPLC 5–95 % MeCN in H_2O. Purity based on integration is 95 %.

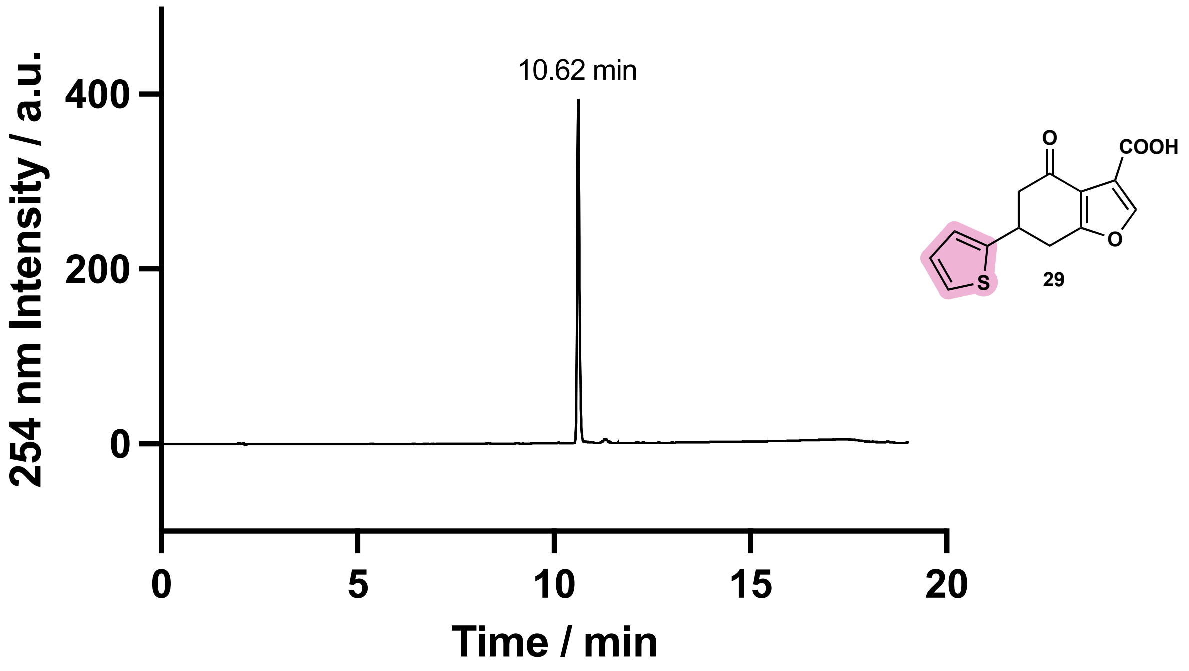

Figure S34 - Compound **29**, HPLC 5–95 % MeCN in H_2O. Purity based on integration is 96 %.

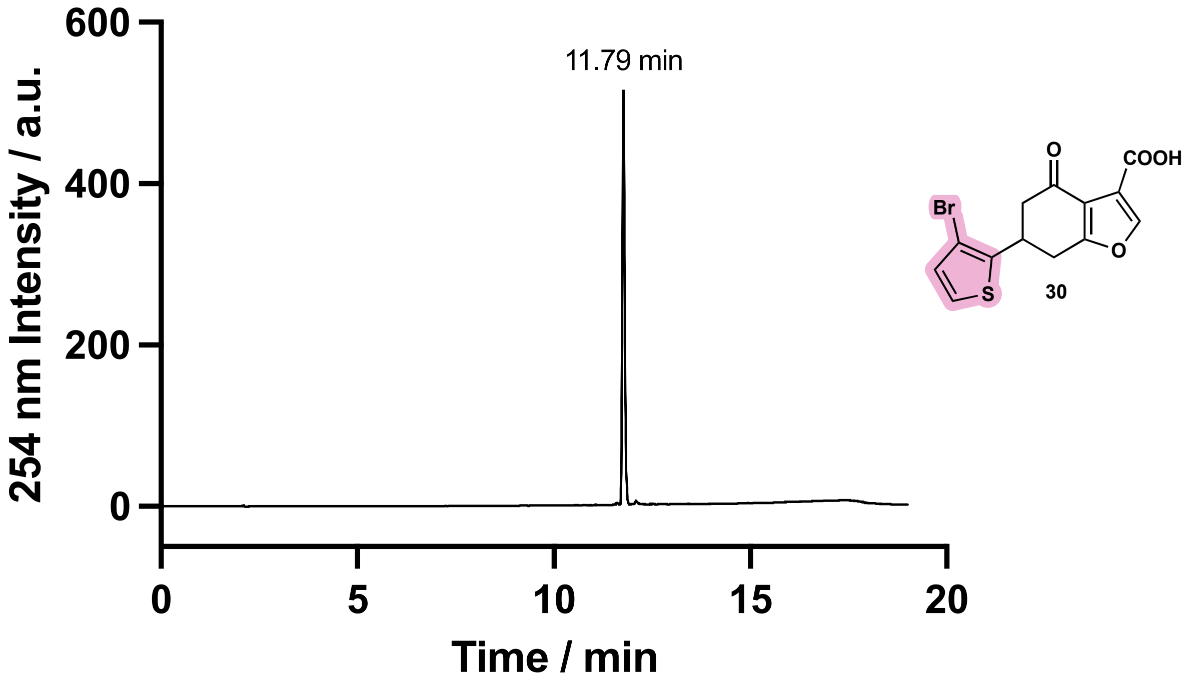

Figure S35 - Compound **30**, HPLC 5–95 % MeCN in H_2O. Purity based on integration is 100 %.

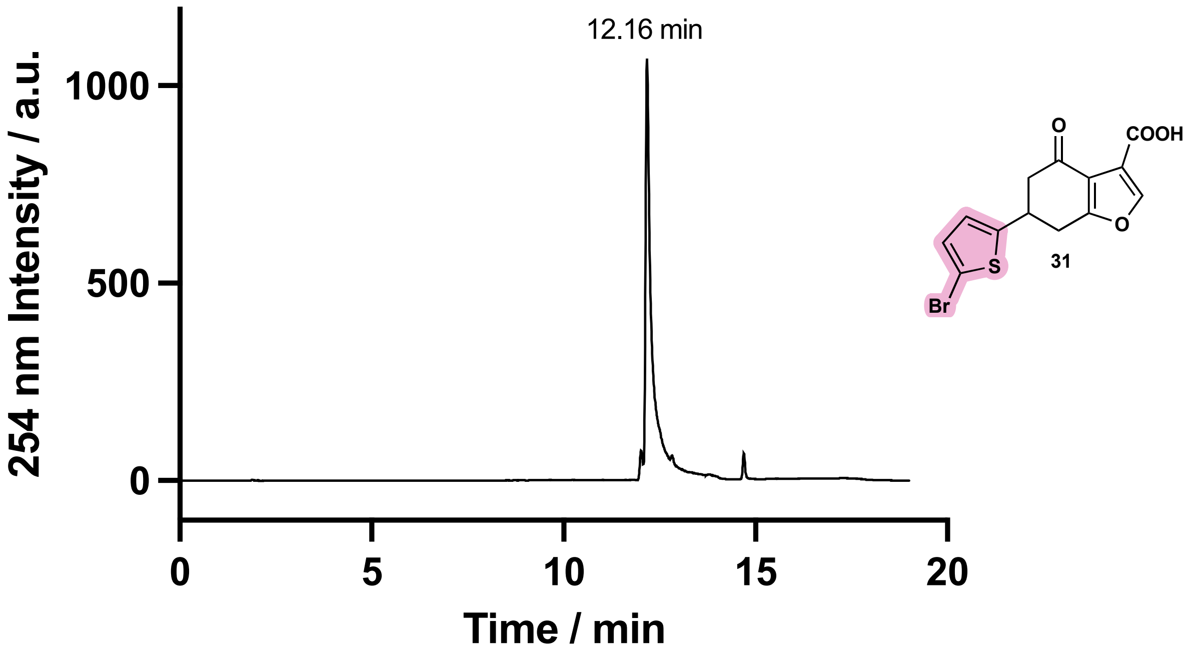

Figure S36 - Compound **31**, HPLC 5–95 % MeCN in H_2O. Purity based on integration is 90 %.

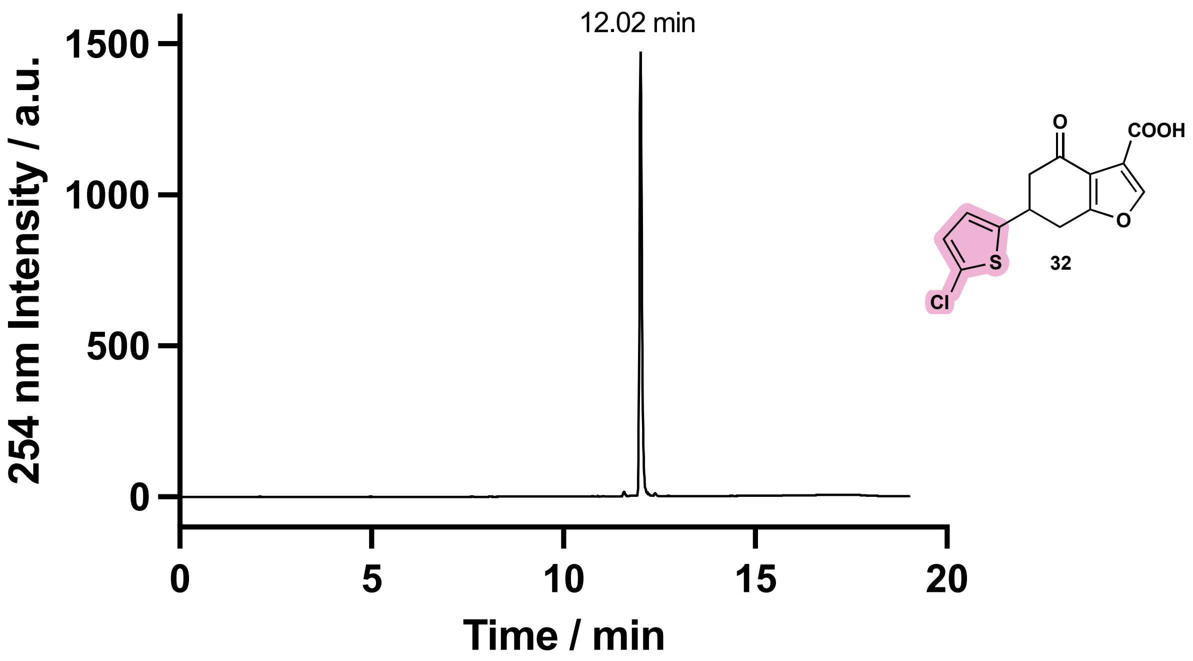

Figure S37 - Compound **32**, HPLC 5–95 % MeCN in H_2O. Purity based on integration is 95 %.

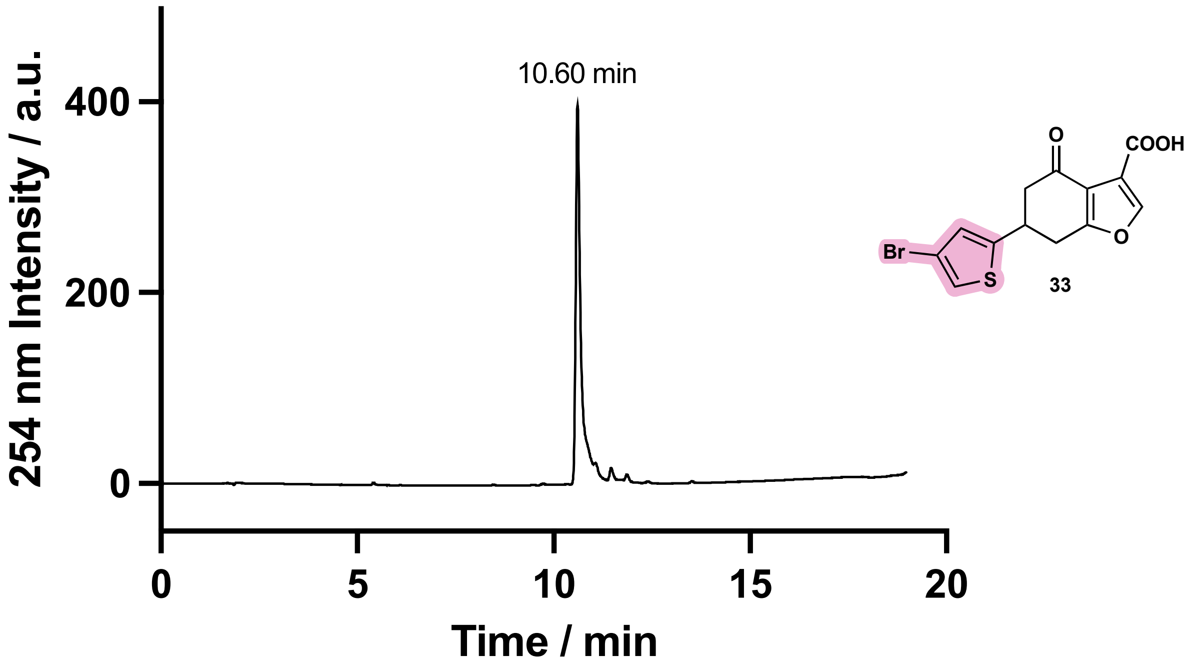

Figure S38 - Compound **33**, HPLC 5–95 % MeCN in H_2O. Purity based on integration is 95 %.

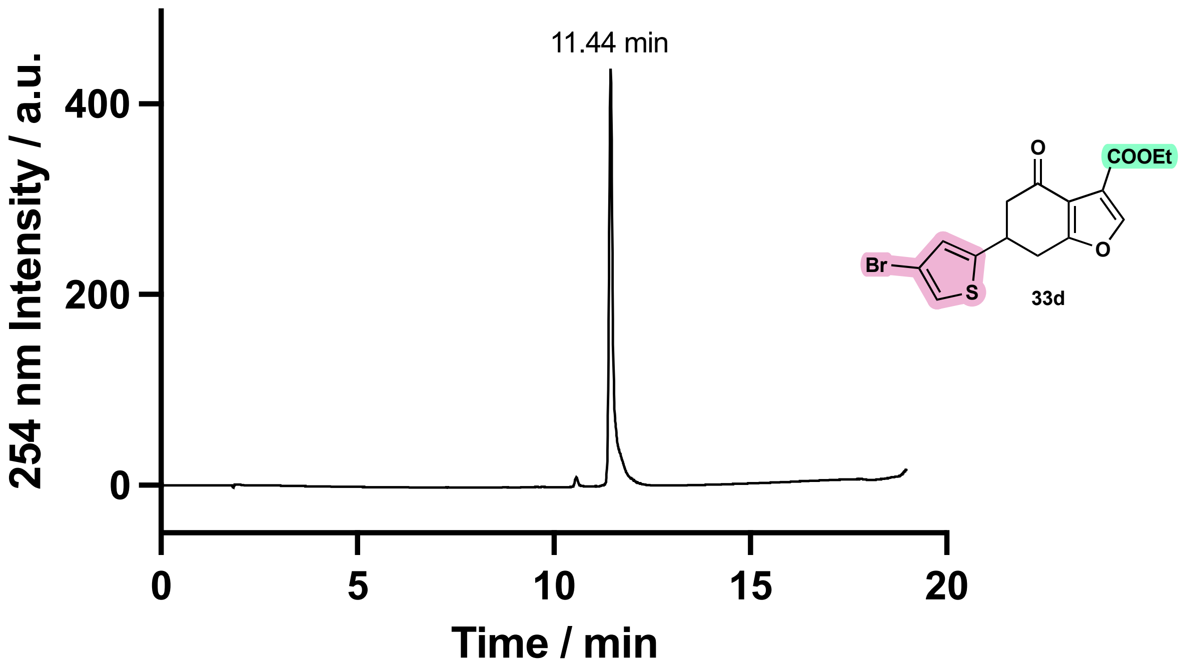

Figure S39 -Compound **33d**, HPLC 5–95 % MeCN in H_2O. Purity based on integration is 95 %.

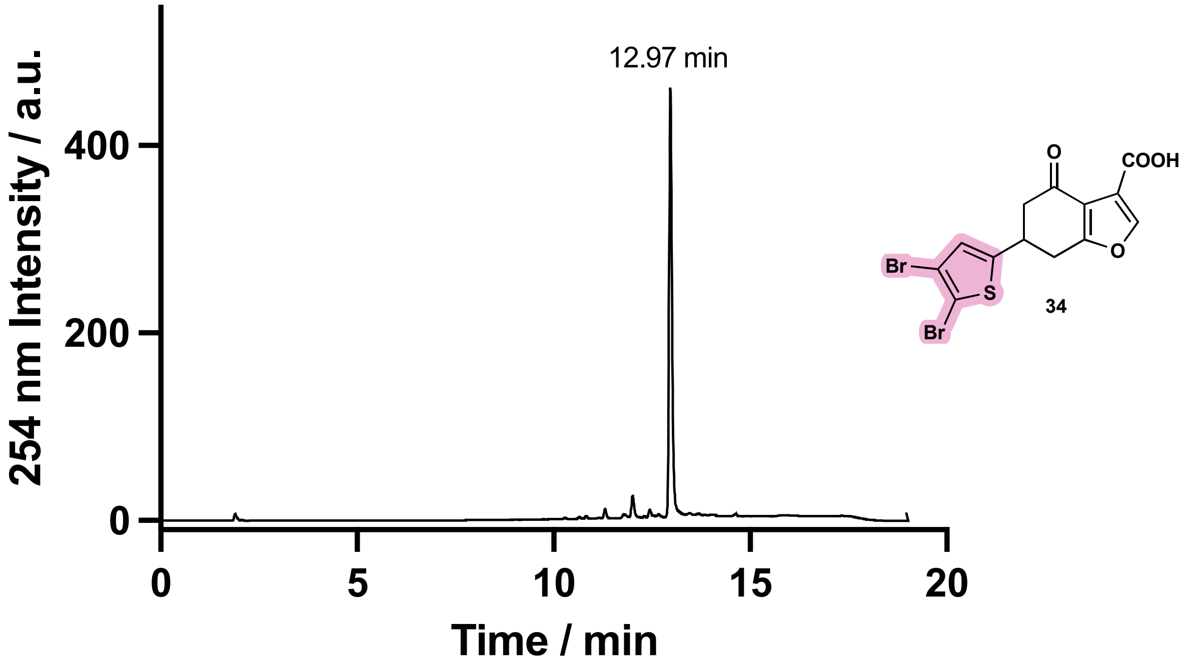

Figure S40 - Compound **34**, HPLC 5–95 % MeCN in H_2O. Purity based on integration is 90 %.

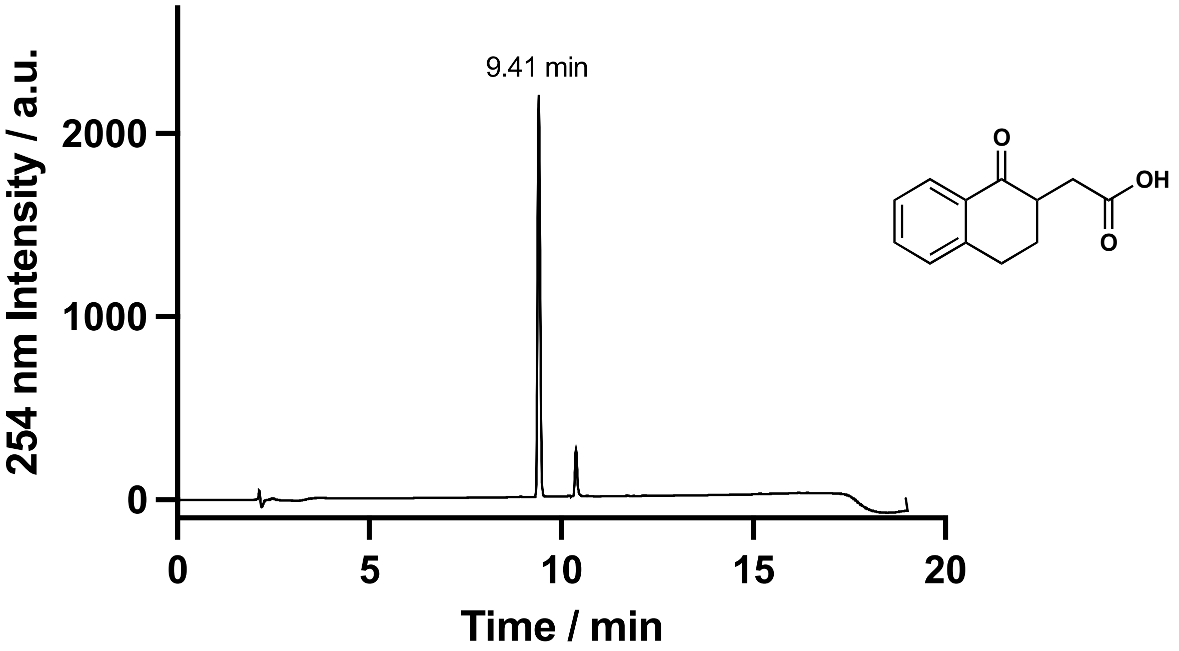

Figure S41 - Compound **37**, HPLC 5–95 % MeCN in H_2O. Purity based on integration is 95 %.

S4 Mass Spectrometry Studies

S4.1 Deconvoluted Spectra for Figure 8A and 8B

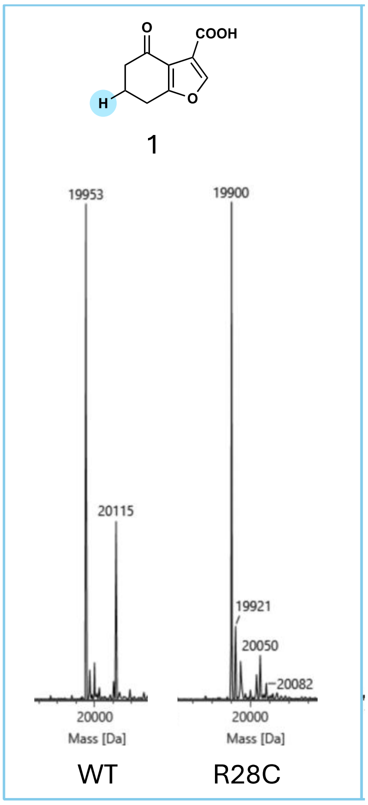

Figure S42 – Mass spectrum of compound **1** (180 Da, 150 μM) incubated at 37 °C for 5 minutes with WT protein (19953 Da, 2.5 μM) and R28C mutant (19900 Da, 2.5 μM) in pH 8 buffer (20 mM HEPES, TCEP 0.5 mM, NaCl 100 mM). Covalent adduct with WT = 19953 + 180 – 18 = 20115 Da.

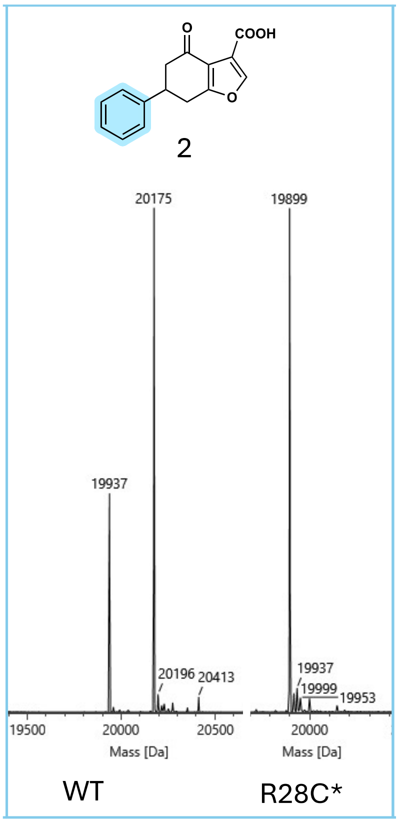

Figure S43 – Mass spectrum of compound **2** (256 Da, 150 µM) incubated at 37 °C for 5 minutes with WT protein (19937 Da, 2.5 µM) and R28C mutant (19900 Da, 2.5 µM) in pH 8 buffer (20 mM HEPES, TCEP 0.5 mM, NaCl 100 mM). Covalent adduct with WT = 19937 + 256 – 18 = 20175 Da.

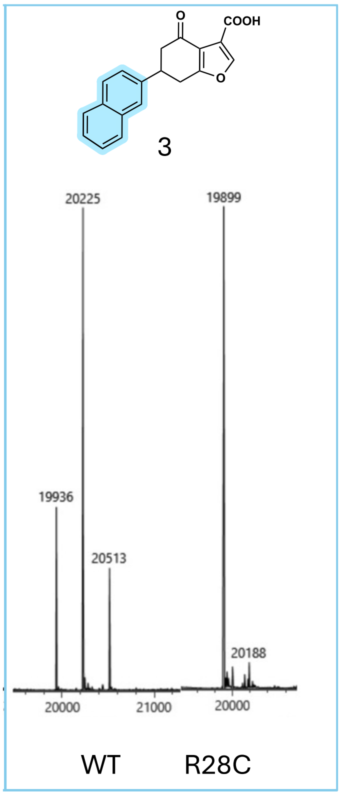

Figure S44 – Mass spectrum of compound **3** (306 Da, 150 µM) incubated at 37 °C for 5 minutes with WT protein (19936 Da, 2.5 µM) and R28C mutant (19900 Da, 2.5 µM) in pH 8 buffer (20 mM HEPES, TCEP 0.5 mM, NaCl 100 mM). Covalent adduct with WT = 19936 + 306 – 18 = 20225 Da.

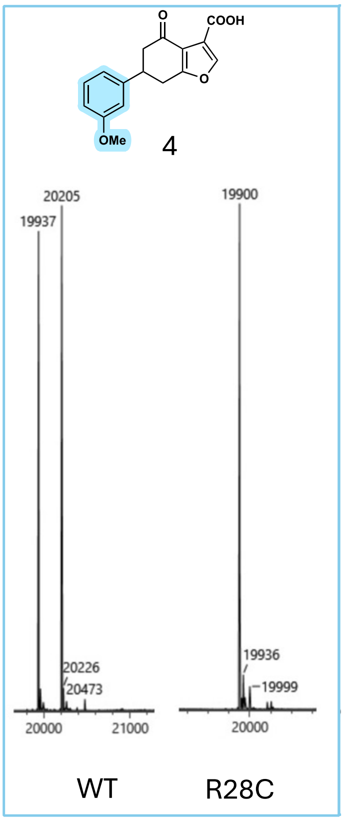

Figure S45 – Mass spectrum of compound **4** (286 Da, 150 μM) incubated at 37 ºC for 5 minutes with WT protein (19937 Da, 2.5 μM) and R28C mutant (19900 Da, 2.5 μM) in pH 8 buffer (20 mM HEPES, TCEP 0.5 mM, NaCl 100 mM). Covalent adduct with WT = 19937 + 286 – 18 = 20205 Da.

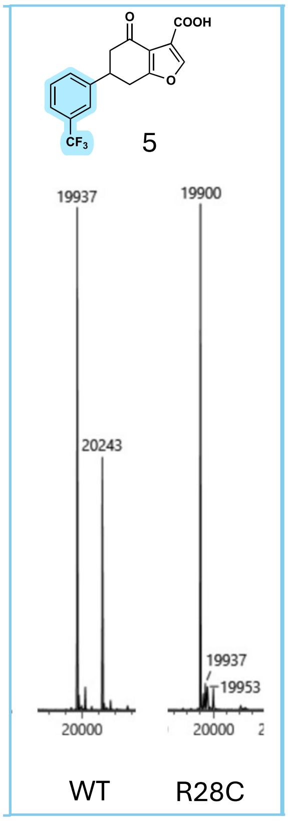

Figure S46 – Mass spectrum of compound **5** (324 Da, 150 μM) incubated at 37 ºC for 5 minutes with WT protein (19937 Da, 2.5 μM) and R28C mutant (19900 Da, 2.5 μM) in pH 8 buffer (20 mM HEPES, TCEP 0.5 mM, NaCl 100 mM). Covalent adduct with WT = 19937 + 324 – 18 = 20243 Da.

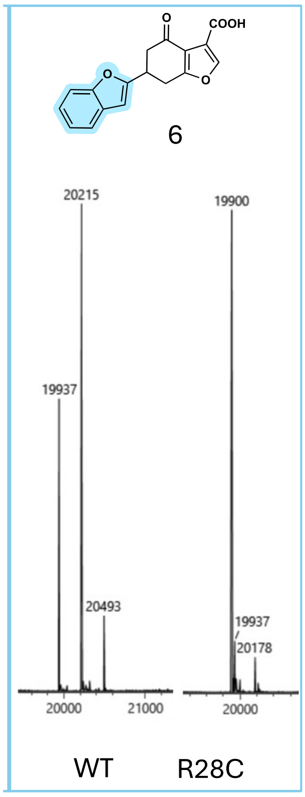

Figure S47 – Mass spectrum of compound **6** (296 Da, 150 µM) incubated at 37 ^{o}C for 5 minutes with WT protein (19937 Da, 2.5 µM) and R28C mutant (19900 Da, 2.5 µM) in pH 8 buffer (20 mM HEPES, TCEP 0.5 mM, NaCl 100 mM). Covalent adduct with WT = 19937 + 296 – 18 = 20215 Da.

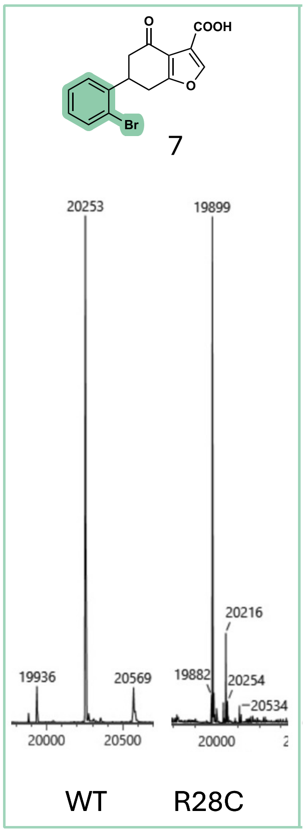

Figure S48 – Mass spectrum of compound **7** (335 Da, 150 µM) incubated at 37 ^{o}C for 5 minutes with WT protein (19936 Da, 2.5 µM) and R28C mutant (19900 Da, 2.5 µM) in pH 8 buffer (20 mM HEPES, TCEP 0.5 mM, NaCl 100 mM). Covalent adduct with WT = 19936 + 335 – 18 = 20253 Da.

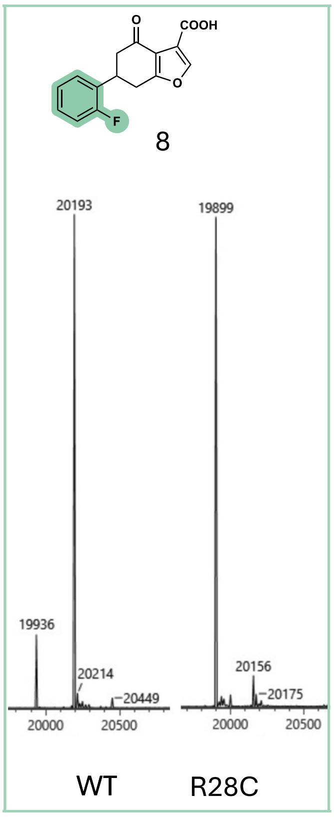

Figure S49 – Mass spectrum of compound **8** (274 Da, 150 μM) incubated at 37 ºC for 5 minutes with WT protein (19936 Da, 2.5 μM) and R28C mutant (19900 Da, 2.5 μM) in pH 8 buffer (20 mM HEPES, TCEP 0.5 mM, NaCl 100 mM). Covalent adduct with WT = 19936 + 274 – 18 = 20192 Da.

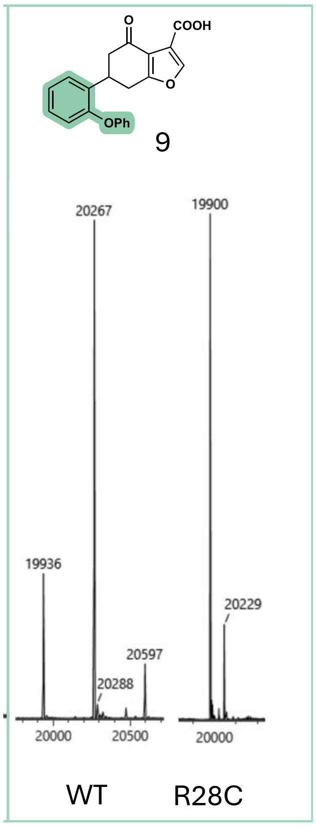

Figure S50 – Mass spectrum of compound **9** (348 Da, 150 μM) incubated at 37 ºC for 5 minutes with WT protein (19936 Da, 2.5 μM) and R28C mutant (19900 Da, 2.5 μM) in pH 8 buffer (20 mM HEPES, TCEP 0.5 mM, NaCl 100 mM). Covalent adduct with WT = 19936 + 348 – 18 = 20266 Da.

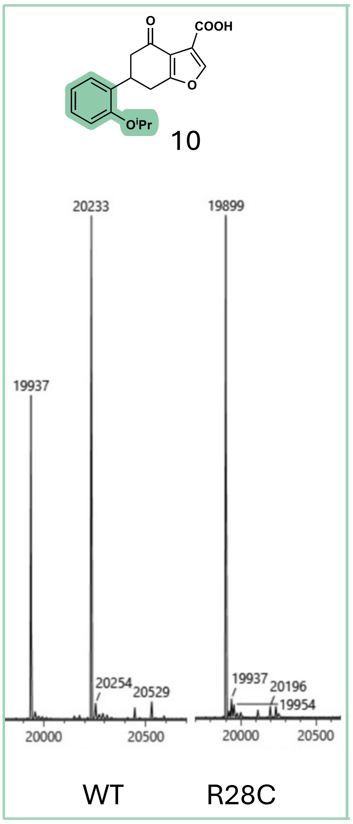

Figure S51 – Mass spectrum of compound **10** (314 Da, 150 μM) incubated at 37 ºC for 5 minutes with WT protein (19937 Da, 2.5 μM) and R28C mutant (19899 Da, 2.5 μM) in pH 8 buffer (20 mM HEPES, TCEP 0.5 mM, NaCl 100 mM). Covalent adduct with WT = 19937 + 314 – 18 = 20233 Da.

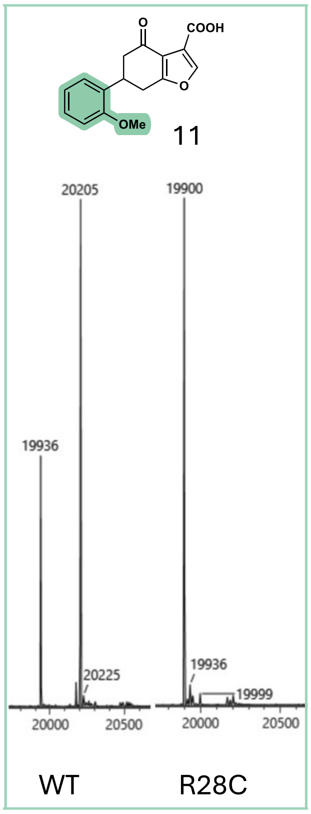

Figure S52 – Mass spectrum of compound **11** (286 Da, 150 μM) incubated at 37 ºC for 5 minutes with WT protein (19936 Da, 2.5 μM) and R28C mutant (19900 Da, 2.5 μM) in pH 8 buffer (20 mM HEPES, TCEP 0.5 mM, NaCl 100 mM). Covalent adduct with WT = 19936 + 286 – 18 = 20204 Da.

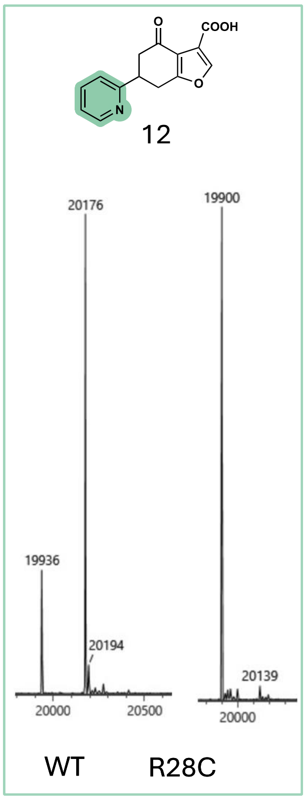

Figure S53 – Mass spectrum of compound **12** (257 Da, 150 μM) incubated at 37 °C for 5 minutes with WT protein (19936 Da, 2.5 μM) and R28C mutant (19900 Da, 2.5 μM) in pH 8 buffer (20 mM HEPES, TCEP 0.5 mM, NaCl 100 mM). Covalent adduct with WT = 19936 + 257 – 18 = 20175 Da.

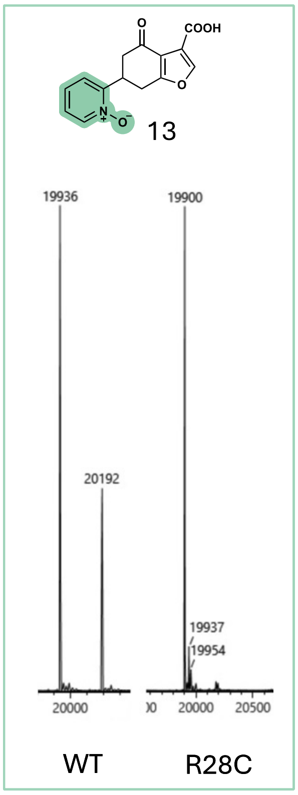

Figure S54 – Mass spectrum of compound **13** (273 Da, 150 μM) incubated at 37 °C for 5 minutes with WT protein (19936 Da, 2.5 μM) and R28C mutant (19900 Da, 2.5 μM) in pH 8 buffer (20 mM HEPES, TCEP 0.5 mM, NaCl 100 mM). Covalent adduct with WT = 19936 + 273 – 18 = 20191 Da.

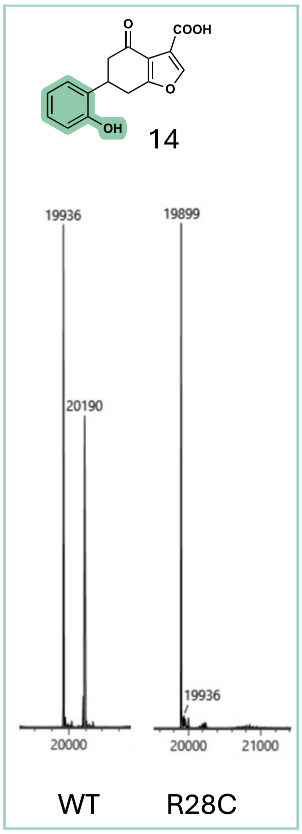

Figure S55 – Mass spectrum of compound **14** (272 Da, 150 µM) incubated at 37 °C for 5 minutes with WT protein (19936 Da, 2.5 µM) and R28C mutant (19900 Da, 2.5 µM) in pH 8 buffer (20 mM HEPES, TCEP 0.5 mM, NaCl 100 mM). Covalent adduct with WT = 19936 + 272 – 18 = 20190 Da.

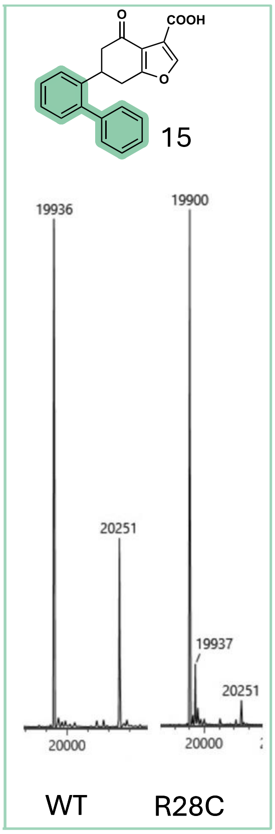

Figure S56 – Mass spectrum of compound **15** (332 Da, 150 µM) incubated at 37 °C for 5 minutes with WT protein (19936 Da, 2.5 µM) and R28C mutant (19900 Da, 2.5 µM) in pH 8 buffer (20 mM HEPES, TCEP 0.5 mM, NaCl 100 mM). Covalent adduct with WT = 19936 + 332 – 18 = 20250 Da.

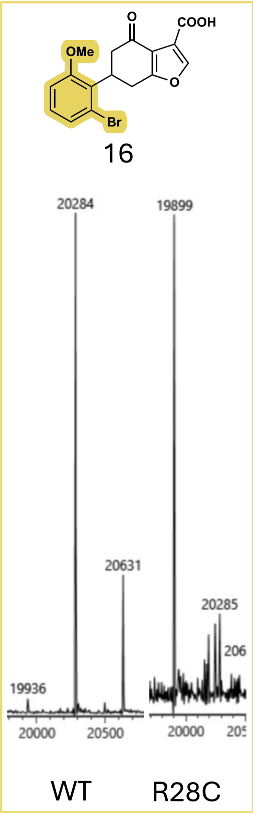

Figure S57 – Mass spectrum of compound **16** (365 Da, 150 μM) incubated at 37 ºC for 5 minutes with WT protein (19936 Da, 2.5 μM) and R28C mutant (19900 Da, 2.5 μM) in pH 8 buffer (20 mM HEPES, TCEP 0.5 mM, NaCl 100 mM). Covalent adduct with WT = 19936 + 365 – 18 = 20283 Da.

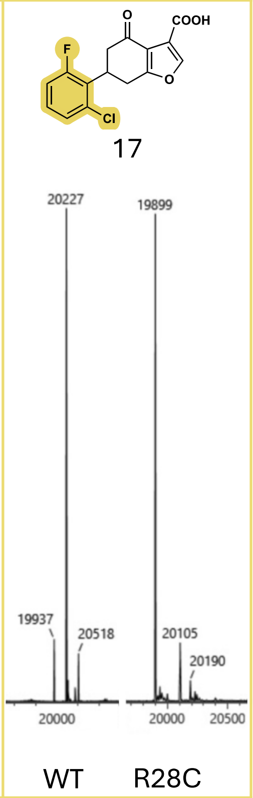

Figure S58 – Mass spectrum of compound **17** (309 Da, 150 μM) incubated at 37 ºC for 5 minutes with WT protein (19937 Da, 2.5 μM) and R28C mutant (19900 Da, 2.5 μM) in pH 8 buffer (20 mM HEPES, TCEP 0.5 mM, NaCl 100 mM). Covalent adduct with WT = 19937 + 309 – 18 = 20228 Da.

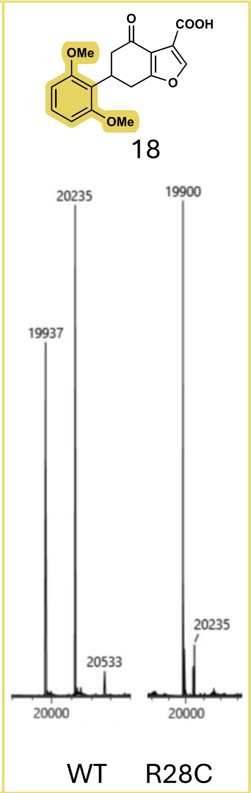

Figure S59 – Mass spectrum of compound **18** (316 Da, 150 µM) incubated at 37 °C for 5 minutes with WT protein (19937 Da, 2.5 µM) and R28C mutant (19900 Da, 2.5 µM) in pH 8 buffer (20 mM HEPES, TCEP 0.5 mM, NaCl 100 mM). Covalent adduct with WT = 19937 + 316 – 18 = 20235 Da.

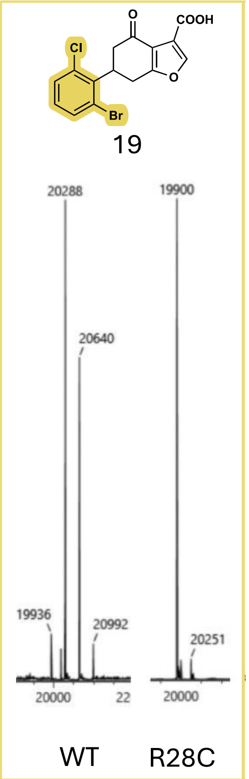

Figure S60 – Mass spectrum of compound **19** (370 Da, 150 µM) incubated at 37 °C for 5 minutes with WT protein (19936 Da, 2.5 µM) and R28C mutant (19900 Da, 2.5 µM) in pH 8 buffer (20 mM HEPES, TCEP 0.5 mM, NaCl 100 mM). Covalent adduct with WT = 19936 + 370 – 18 = 20288 Da.

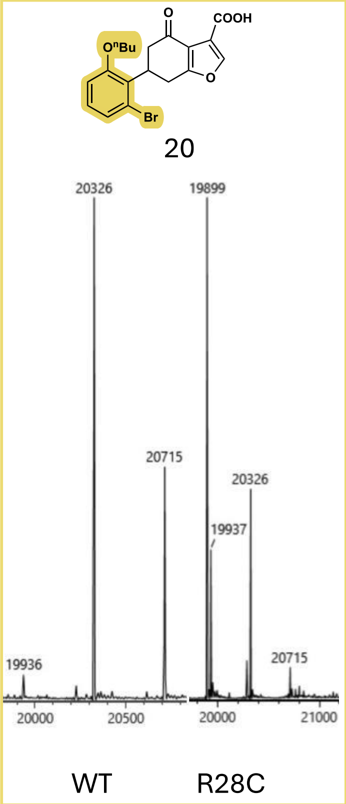

Figure S61 – Mass spectrum of compound **20** (407 Da, 150 μM) incubated at 37 ºC for 5 minutes with WT protein (19936 Da, 2.5 μM) and R28C mutant (19900 Da, 2.5 μM) in pH 8 buffer (20 mM HEPES, TCEP 0.5 mM, NaCl 100 mM). Covalent adduct with WT = 19936 + 407 – 18 = 20325 Da.

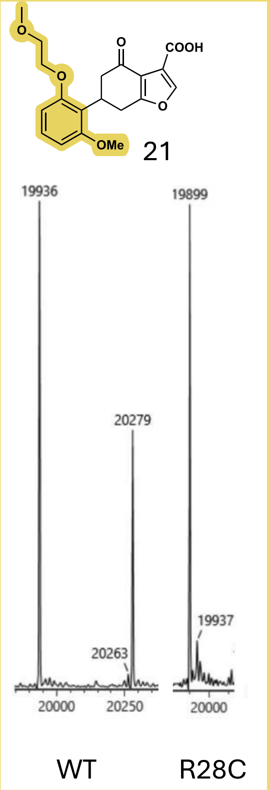

Figure S62 – Mass spectrum of compound **21** (360 Da, 150 μM) incubated at 37 ºC for 5 minutes with WT protein (19936 Da, 2.5 μM) and R28C mutant (19900 Da, 2.5 μM) in pH 8 buffer (20 mM HEPES, TCEP 0.5 mM, NaCl 100 mM). Covalent adduct with WT = 19936 + 360 – 18 = 20278 Da.

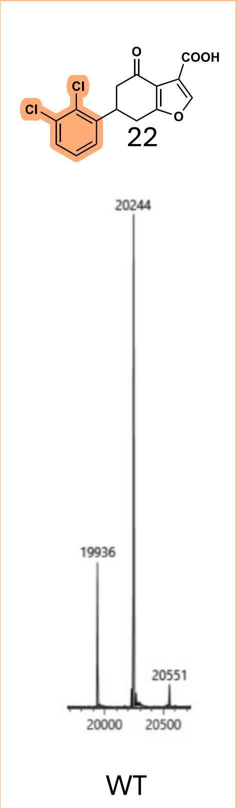

Figure S63 – Mass spectrum of compound **22** (325 Da, 150 μM) incubated at 37 ºC for 5 minutes with WT protein (19936 Da, 2.5 μM) in pH 8 buffer (20 mM HEPES, TCEP 0.5 mM, NaCl 100 mM). Covalent adduct with WT = 19936 + 325 – 18 = 20243 Da.

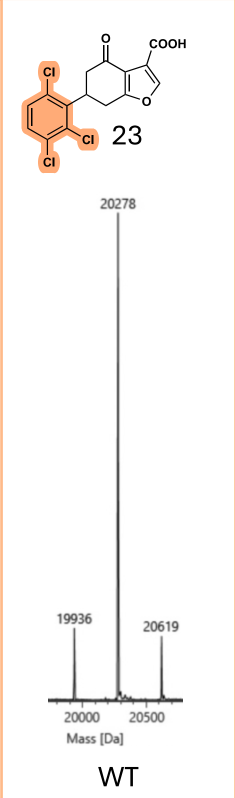

Figure S64 – Mass spectrum of compound **23** (360 Da, 150 μM) incubated at 37 ºC for 5 minutes with WT protein (19936 Da, 2.5 μM) in pH 8 buffer (20 mM HEPES, TCEP 0.5 mM, NaCl 100 mM). Covalent adduct with WT = 19936 + 360 – 18 = 20278 Da.

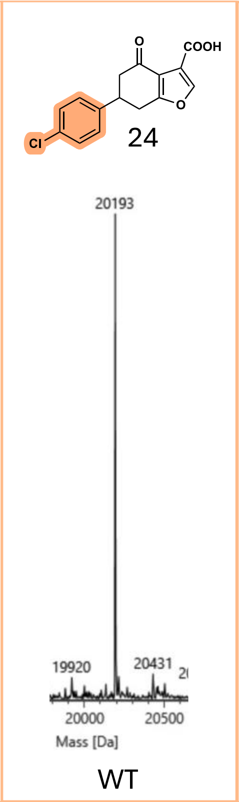

Figure S65 – Mass spectrum of compound **24** (291 Da, 150 μM) incubated at 37 °C for 5 minutes with Ala mutant, binding unaffected, of the WT protein (19920 Da, 2.5 μM) in pH 8 buffer (20 mM HEPES, TCEP 0.5 mM, NaCl 100 mM). Covalent adduct with WT = 19920 + 291 – 18 = 20193 Da.

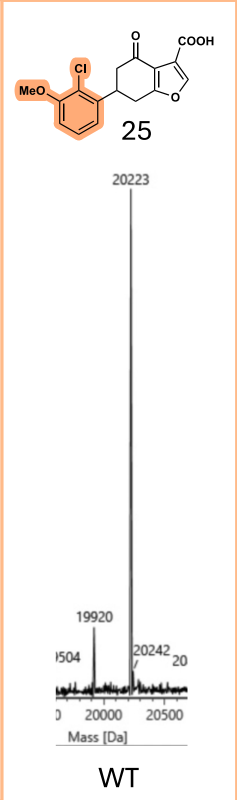

Figure S66 – Mass spectrum of compound **25** (321 Da, 150 μM) incubated at 37 °C for 5 minutes with Ala mutant, binding unaffected, of the WT protein (19920 Da, 2.5 μM) in pH 8 buffer (20 mM HEPES, TCEP 0.5 mM, NaCl 100 mM). Covalent adduct with WT = 19920 + 321 – 18 = 20223 Da.

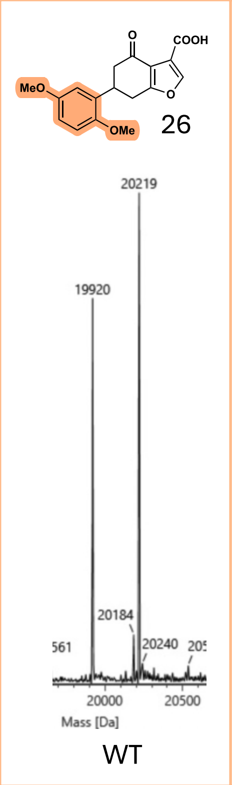

Figure S67 – Mass spectrum of compound **26** (316 Da, 150 µM) incubated at 37 ºC for 5 minutes with Ala mutant, binding unaffected, of the WT protein (19920 Da, 2.5 µM) in pH 8 buffer (20 mM HEPES, TCEP 0.5 mM, NaCl 100 mM). Covalent adduct with WT = 19920 + 316 – 18 = 20218 Da.

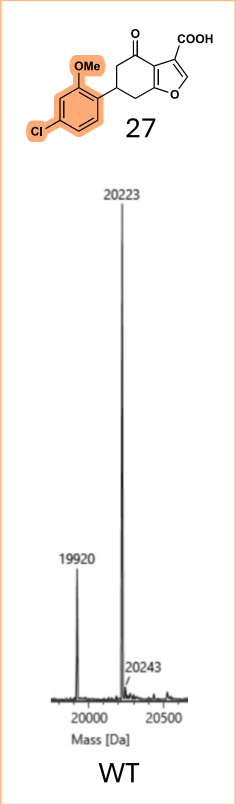

Figure S68 – Mass spectrum of compound **27** (321 Da, 150 µM) incubated at 37 ºC for 5 minutes with Ala mutant, binding unaffected, of the WT protein (19920 Da, 2.5 µM) in pH 8 buffer (20 mM HEPES, TCEP 0.5 mM, NaCl 100 mM). Covalent adduct with WT = 19920 + 321 – 18 = 20223 Da.

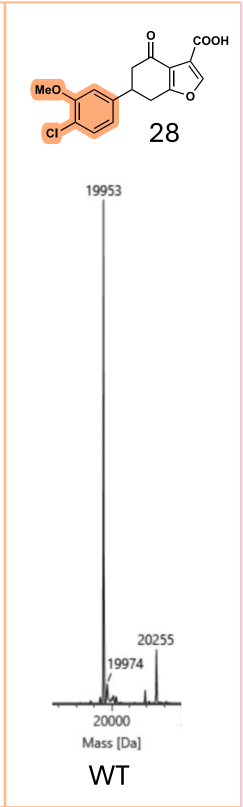

Figure S69 – Mass spectrum of compound **28** (321 Da, 150 µM) incubated at 37 °C for 5 minutes with WT protein (19953 Da, 2.5 µM) in pH 8 buffer (20 mM HEPES, TCEP 0.5 mM, NaCl 100 mM). Covalent adduct with WT = 19953 + 321 – 18 = 20256 Da.

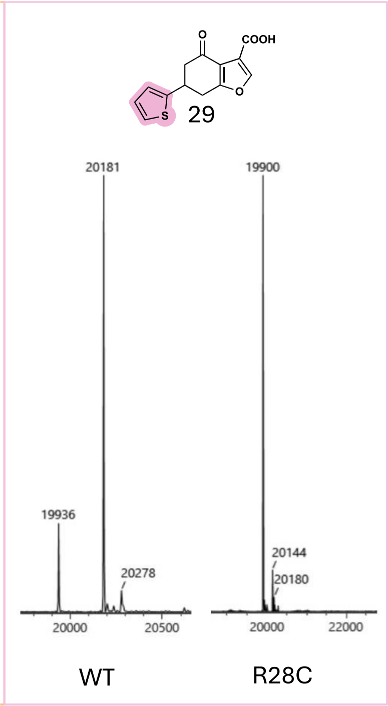

Figure S70 – Mass spectrum of compound **29** (262 Da, 150 µM) incubated at 37 °C for 5 minutes with WT protein (19936 Da, 2.5 µM) and R28C mutant (19900 Da, 2.5 µM) in pH 8 buffer (20 mM HEPES, TCEP 0.5 mM, NaCl 100 mM). Covalent adduct with WT = 19936 + 262 – 18 = 20180 Da.

Table S2 – Mass spectrum of thiophenes **30** - **34** (150 µM) in competition with the unsubstituted thiophene **29** (150 µM), incubated at 37 ºC for 1 hr with WT protein (19936 Da, 2.5 µM) or Ala mutant, binding unaffected, of the WT protein (19920 Da, 2.5 µM) in pH 8 buffer (20 mM HEPES, TCEP 0.5 mM, NaCl 100 mM).

29	30	31	32	33	34
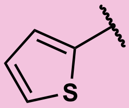 20181/20165 vs.	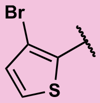	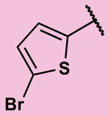	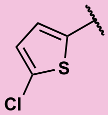	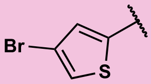	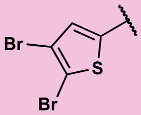
WT/Ala* Mass / Da	19936	19920	19920	19920	19936
WT + lig – 18 / Da	20259	20243	20199	20243	20338
Deconvoluted Spectra	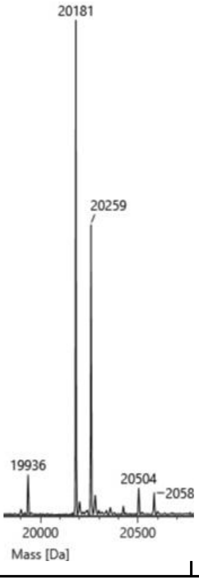	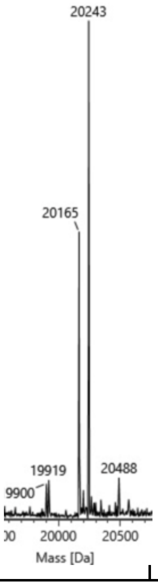	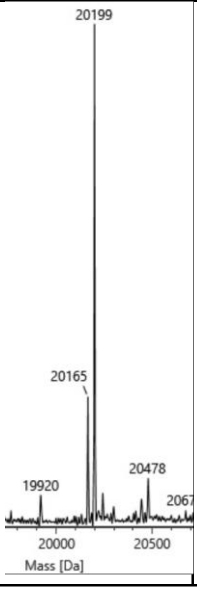	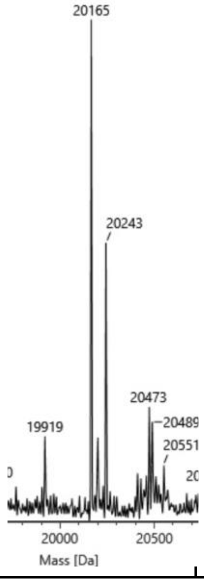	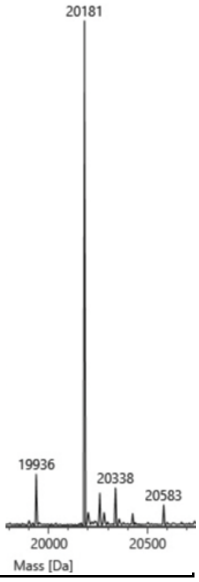

S4.2 Deconvoluted Spectra for Figure 8C, Reversibility Study

Table S3 – A summary of conditions for the reversibility experiment, displaying the structure of compounds **2** and **24** used in the study.

Time / hr	Item Name		
	rm204a – **2** then **24**	204b – **24** then **2**	204e – **2** and **24**
1	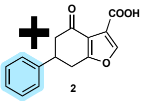	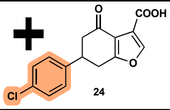	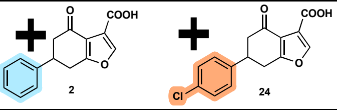
2	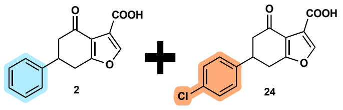	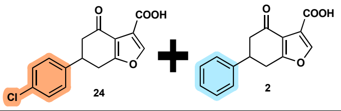	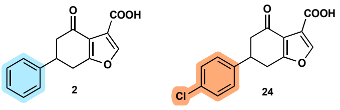
24	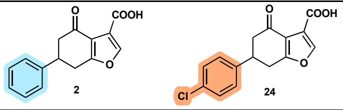	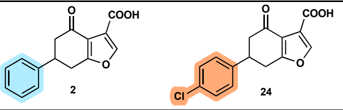	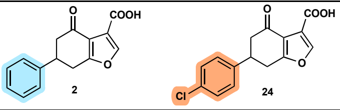

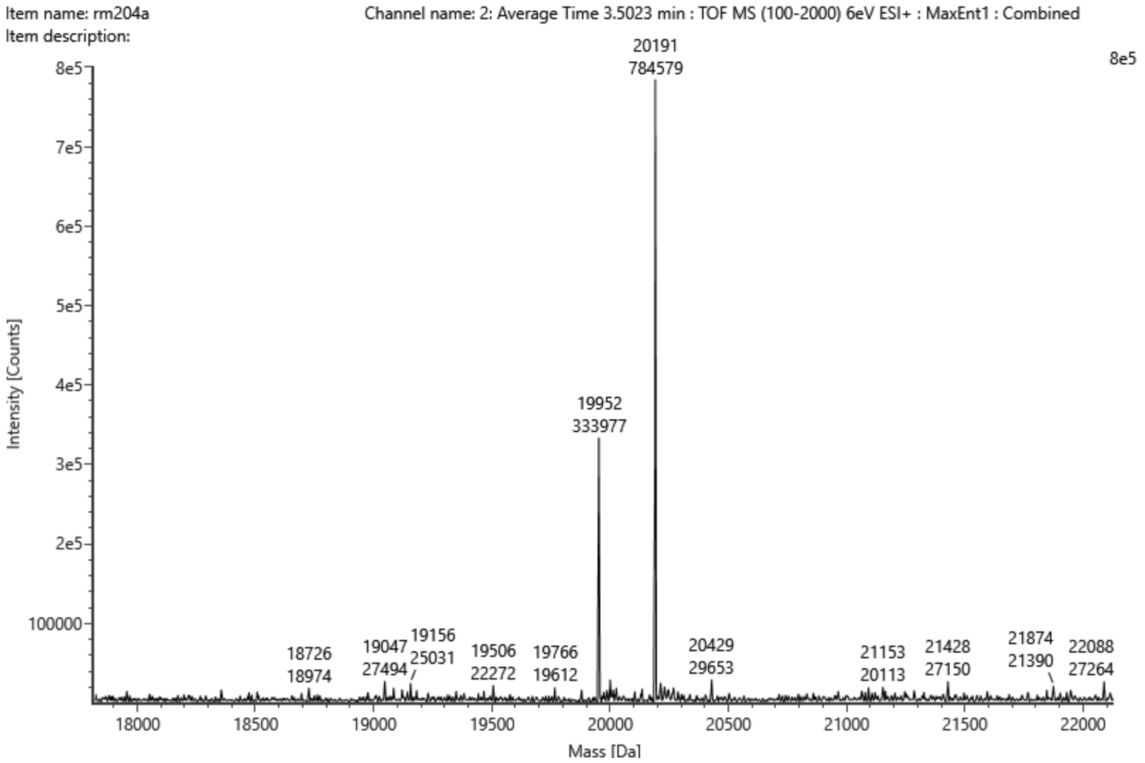

Figure S71 – Mass spectrum with intensities of compound **2** (256 Da, 150 μM) incubated at 37 ºC for 1 hr with WT protein (19952 Da, 2.5 μM) in pH 8 buffer (20 mM HEPES, TCEP 0.5 mM, NaCl 100 mM). Covalent adduct of WT protein + **2** = 19952 + 256 – 18 = 20191 Da.

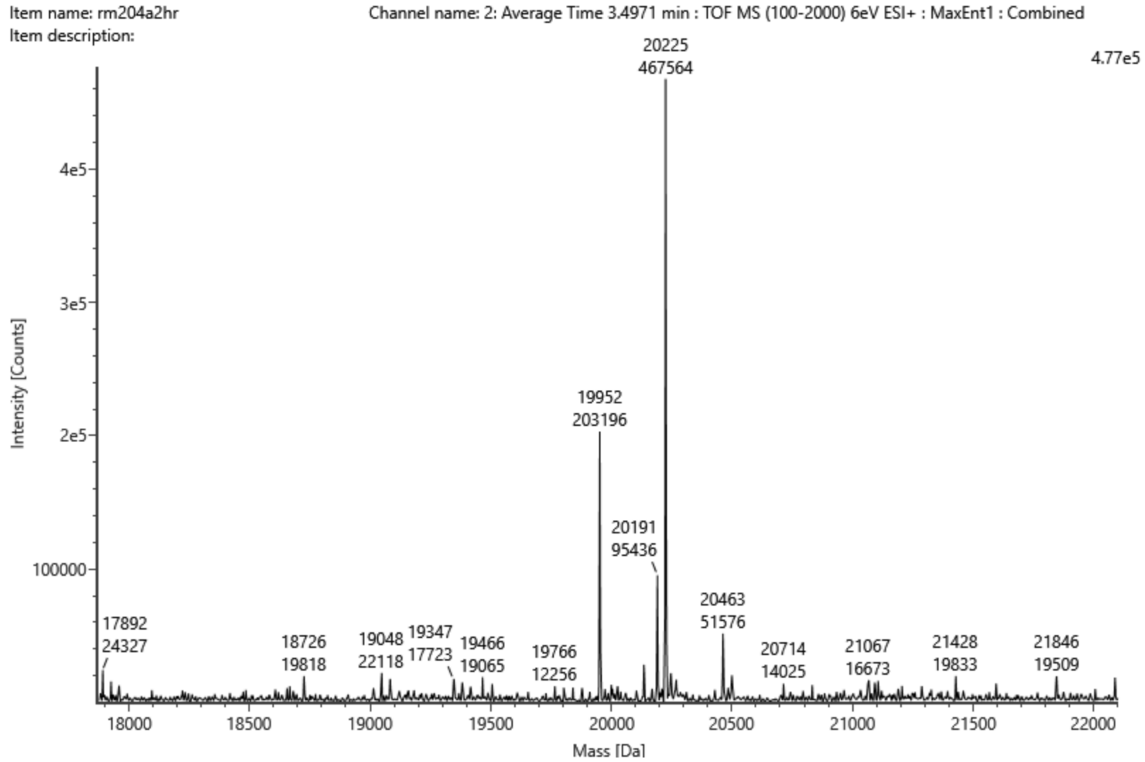

Figure S72 – Mass spectrum with intensities of compound **2** (256 Da, 150 μM) incubated at 37 ºC for 2 hrs, adding compound **24** (291 Da, 150 μM) after 1 hr, with WT protein (19952 Da, 2.5 μM) in pH 8 buffer (20 mM HEPES, TCEP 0.5 mM, NaCl 100 mM). Covalent adduct of WT protein + **2** = 19952 + 256 – 18 = 20191 Da. Covalent adduct of WT protein + **24** = 19952 + 291 – 18 = 20225 Da.

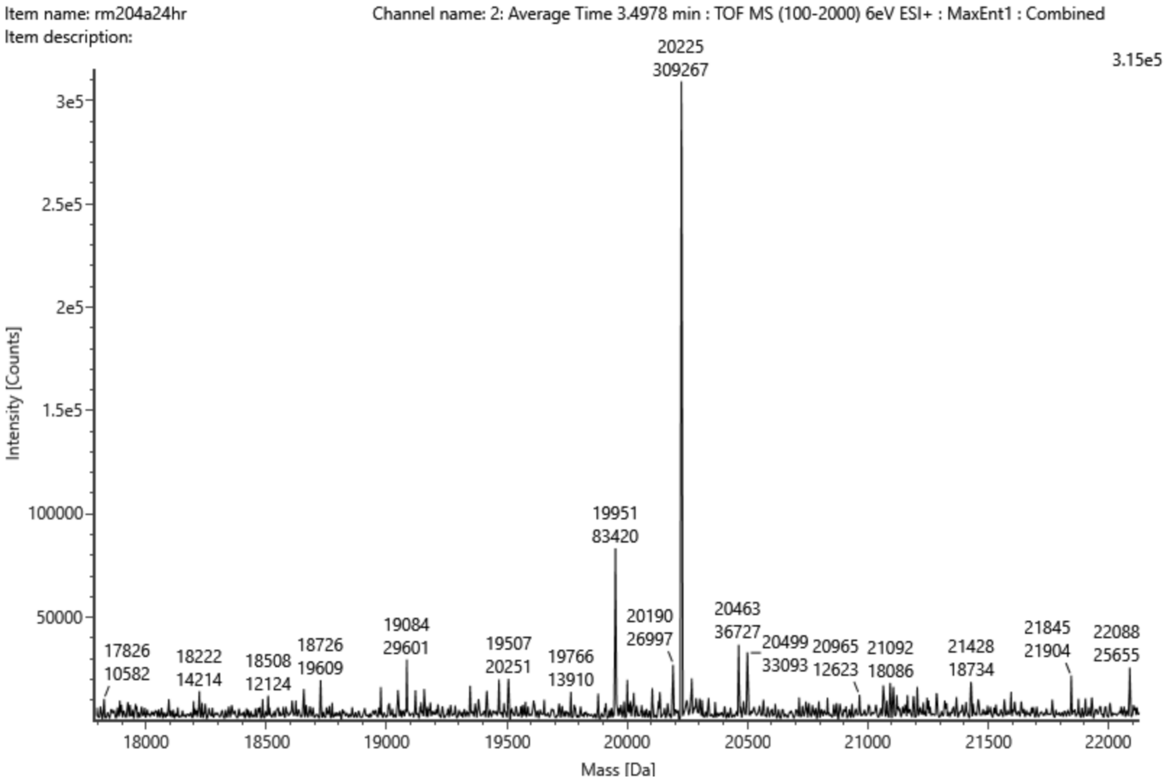

Figure S73 – Mass spectrum with intensities of compound **2** (256 Da, 150 μM) incubated at 37 ºC for 24 hrs, adding compound **24** (291 Da, 150 μM) after 1 hr, with WT protein (19952 Da, 2.5 μM) in pH 8 buffer (20 mM HEPES, TCEP 0.5 mM, NaCl 100 mM). Covalent adduct of WT protein + **2** = 19952 + 256 – 18 = 20191 Da. Covalent adduct of WT protein + **24** = 19952 + 291 – 18 = 20225 Da.

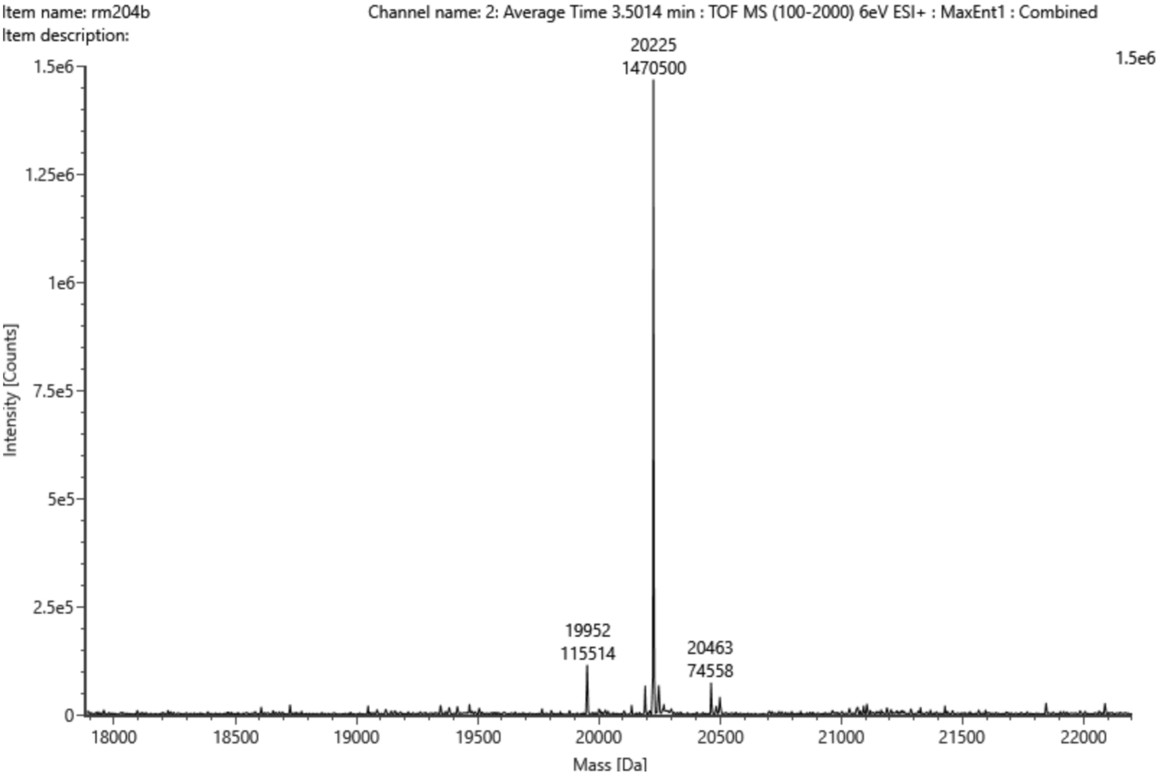

Figure S74 – Mass spectrum with intensities of compound **24** (291 Da, 150 μM) incubated at 37 ºC for 1 hr with WT protein (19952 Da, 2.5 μM) in pH 8 buffer (20 mM HEPES, TCEP 0.5 mM, NaCl 100 mM). Covalent adduct of WT protein + **24** = 19952 + 291 – 18 = 20225 Da.

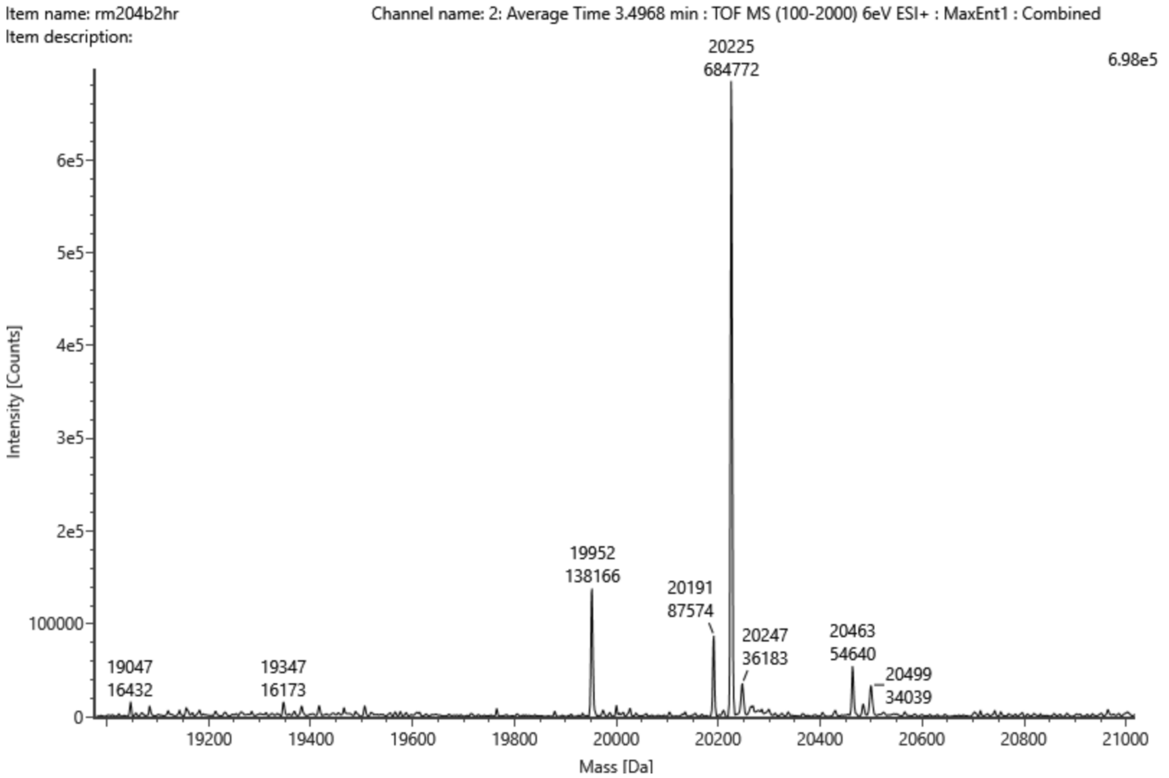

Figure S75 – Mass spectrum with intensities of compound **24** (291 Da, 150 µM) incubated at 37 ºC for 2 hrs, adding compound **2** (256 Da, 150 µM) after 1 hr, with WT protein (19952 Da, 2.5 µM) in pH 8 buffer (20 mM HEPES, TCEP 0.5 mM, NaCl 100 mM). Covalent adduct of WT protein + **24** = 19952 + 291 – 18 = 20225 Da. Covalent adduct of WT protein + **2** = 19952 + 256 – 18 = 20191 Da.

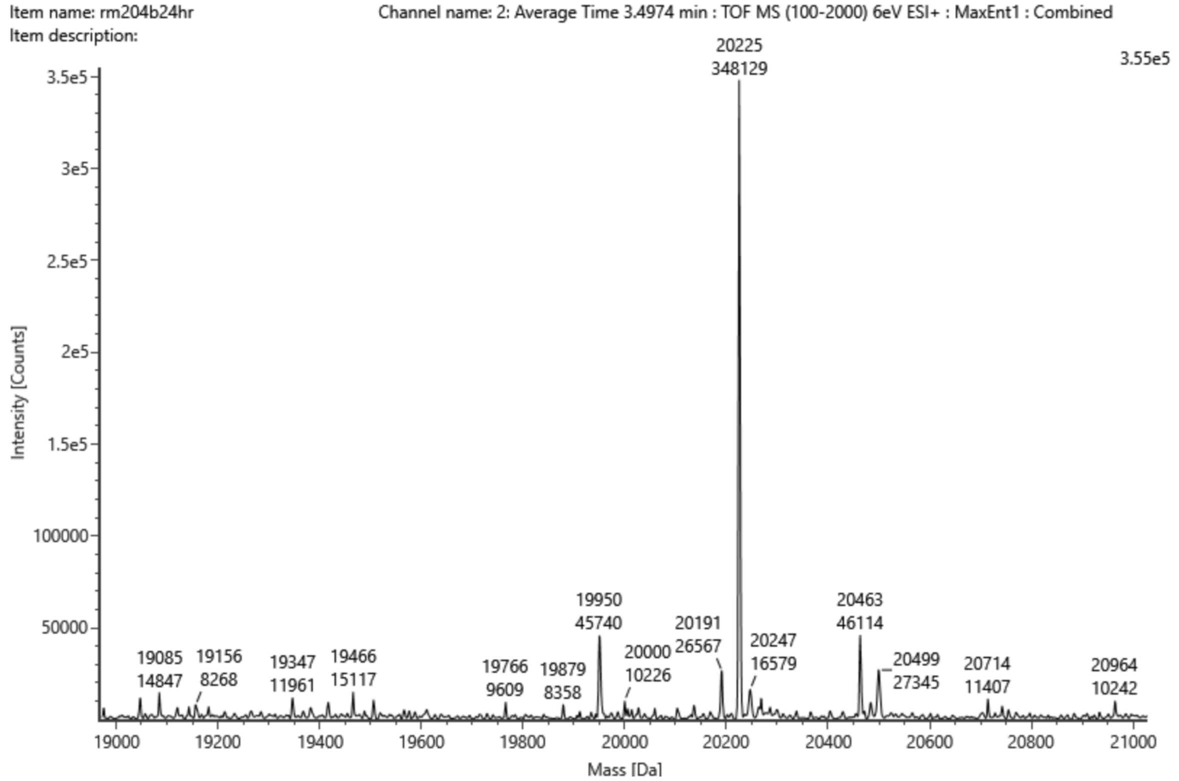

Figure S76 – Mass spectrum with intensities of compound **24** (291 Da, 150 µM) incubated at 37 ºC for 24 hrs, adding compound **2** (256 Da, 150 µM) after 1 hr, with WT protein (19952 Da, 2.5 µM) in pH 8 buffer (20 mM HEPES, TCEP 0.5 mM, NaCl 100 mM). Covalent adduct of WT protein + **24** = 19952 + 291 – 18 = 20225 Da. Covalent adduct of WT protein + **2** = 19952 + 256 – 18 = 20191 Da.

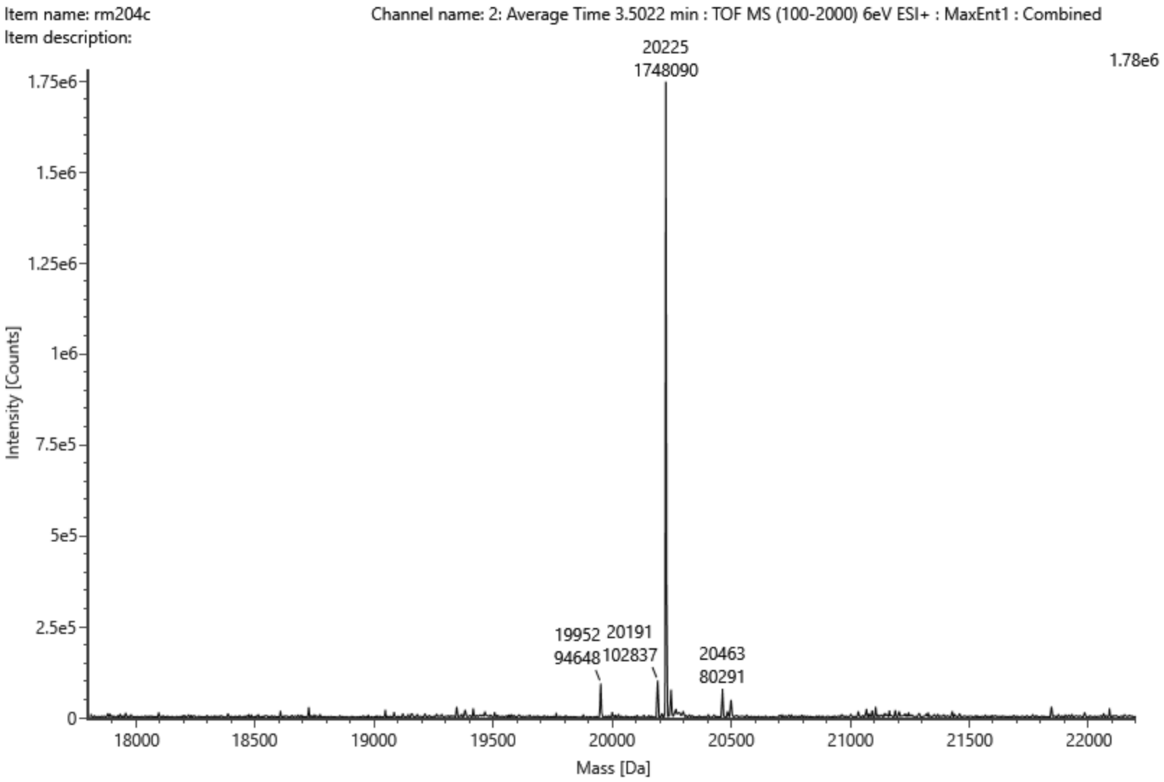

Figure S77 – Mass spectrum with intensities of compound **2** (256 Da, 150 μM) and compound **24** (291 Da, 150 μM) incubated at 37 ºC for 1 hr with WT protein (19952 Da, 2.5 μM) in pH 8 buffer (20 mM HEPES, TCEP 0.5 mM, NaCl 100 mM). Covalent adduct of WT protein + **2** = 19952 + 256 – 18 = 20191 Da. Covalent adduct of WT protein + **24** = 19952 + 291 – 18 = 20225 Da.

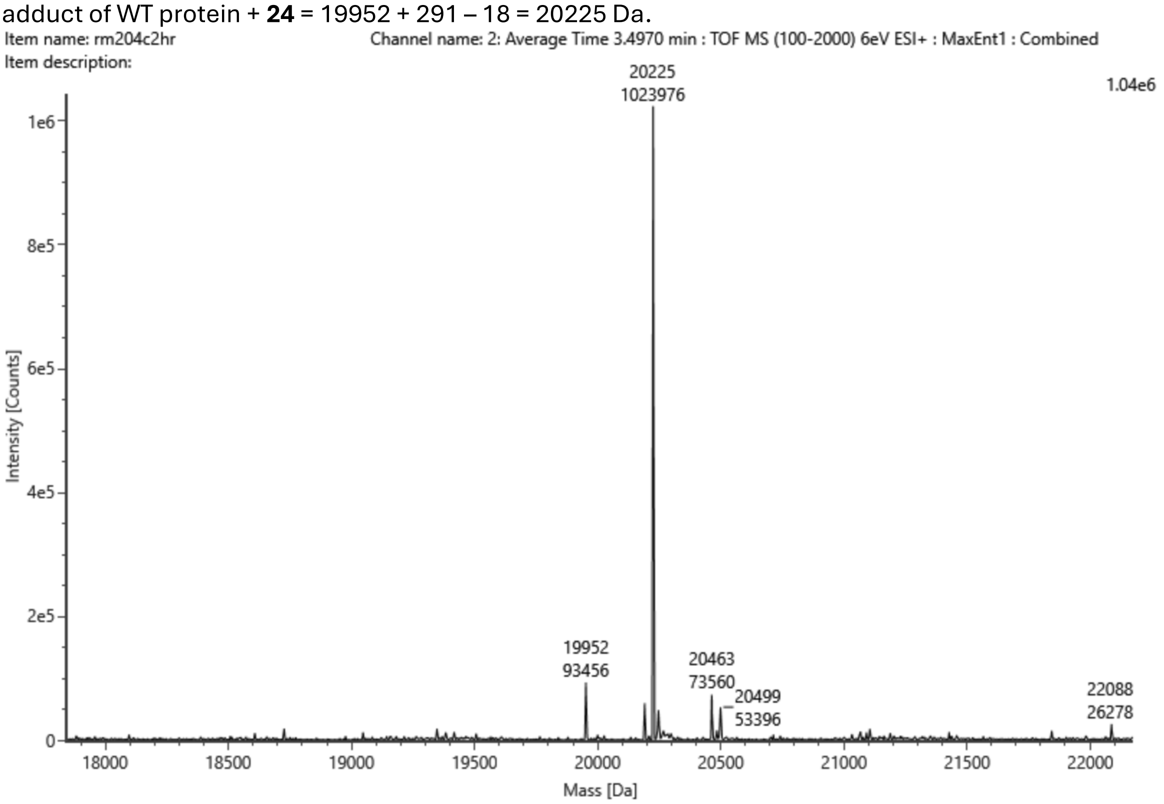

Figure S78 – Mass spectrum with intensities of compound **2** (256 Da, 150 μM) and compound **24** (291 Da, 150 μM) incubated at 37 ºC for 2 hrs with WT protein (19952 Da, 2.5 μM) in pH 8 buffer (20 mM HEPES, TCEP 0.5 mM, NaCl 100 mM). Covalent adduct of WT protein + **2** = 19952 + 256 – 18 = 20191 Da. Covalent adduct of WT protein + **24** = 19952 + 291 – 18 = 20225 Da.

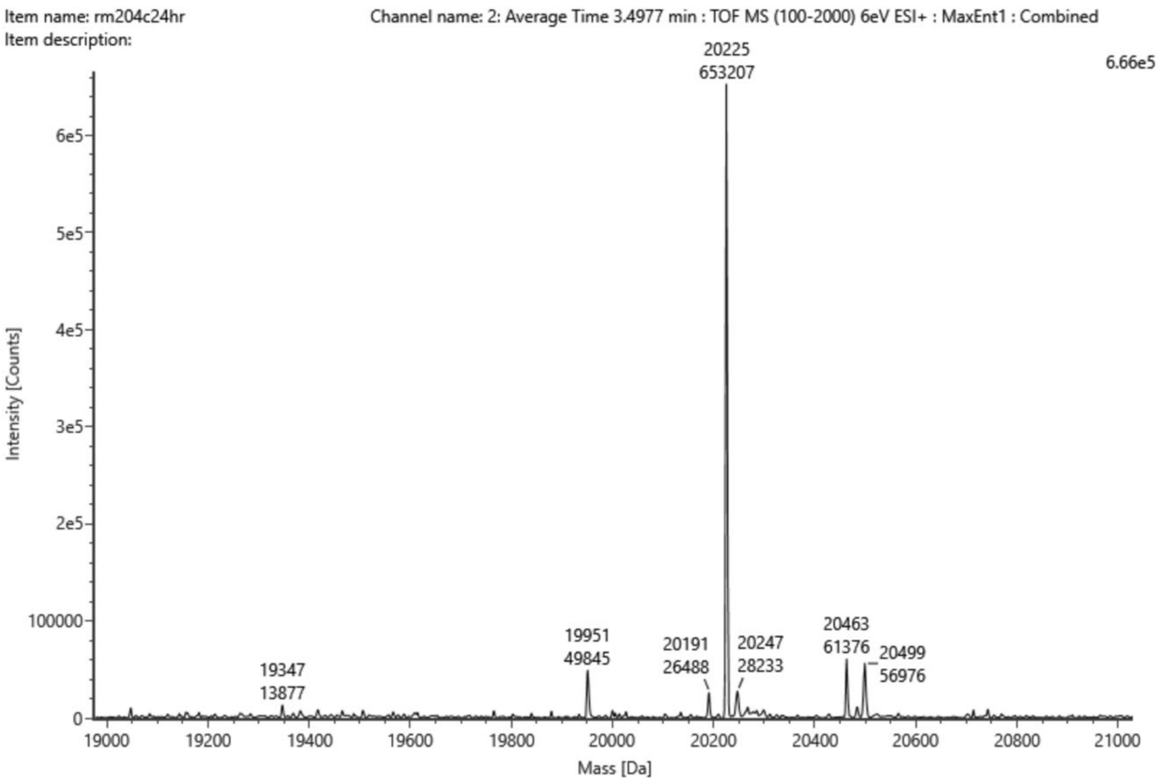

Figure S79 – Mass spectrum with intensities of compound **2** (256 Da, 150 µM) and compound **24** (291 Da, 150 µM) incubated at 37 ºC for 1 hr with WT protein (19952 Da, 2.5 µM) in pH 8 buffer (20 mM HEPES, TCEP 0.5 mM, NaCl 100 mM). Covalent adduct of WT protein + **2** = 19952 + 256 – 18 = 20191 Da. Covalent adduct of WT protein + **24** = 19952 + 291 – 18 = 20225 Da.

S4.3 pH and time dependence of reaction, Figure S80 and Deconvoluted Spectra

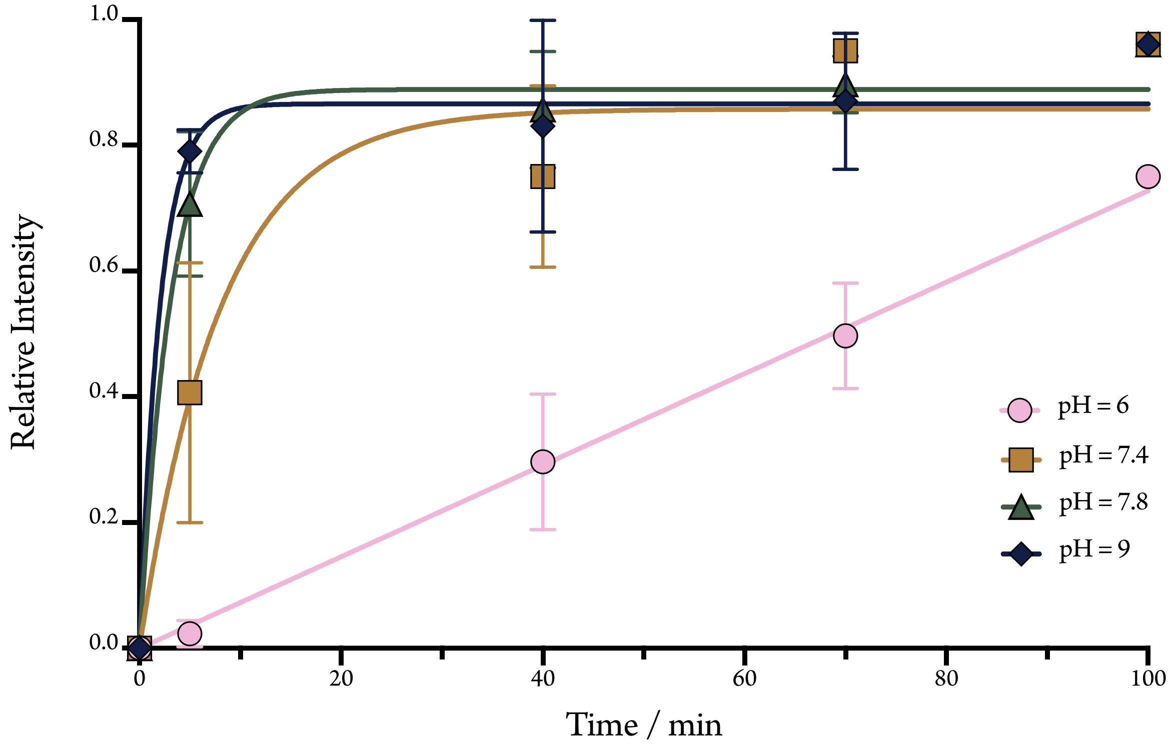

Figure S80 – Investigating pH and time dependence of labelling by comparing the intensity of peaks after incubation of **16** (150 μM) with the WT BTK PH domain (2.5 μM) for 5, 40, 70 and 100 minutes in 20 mM HEPES buffers (100 mM NaCl, 0.5 mM TCEP) of various pH values (pH 6, 7.4, 7.8 and 9). The data does not distinguish single and non-specific labelling as it shows the intensity of total labelling (singly and doubly labelled protein) relative to total protein labelling (singly, doubly and unlabelled protein).

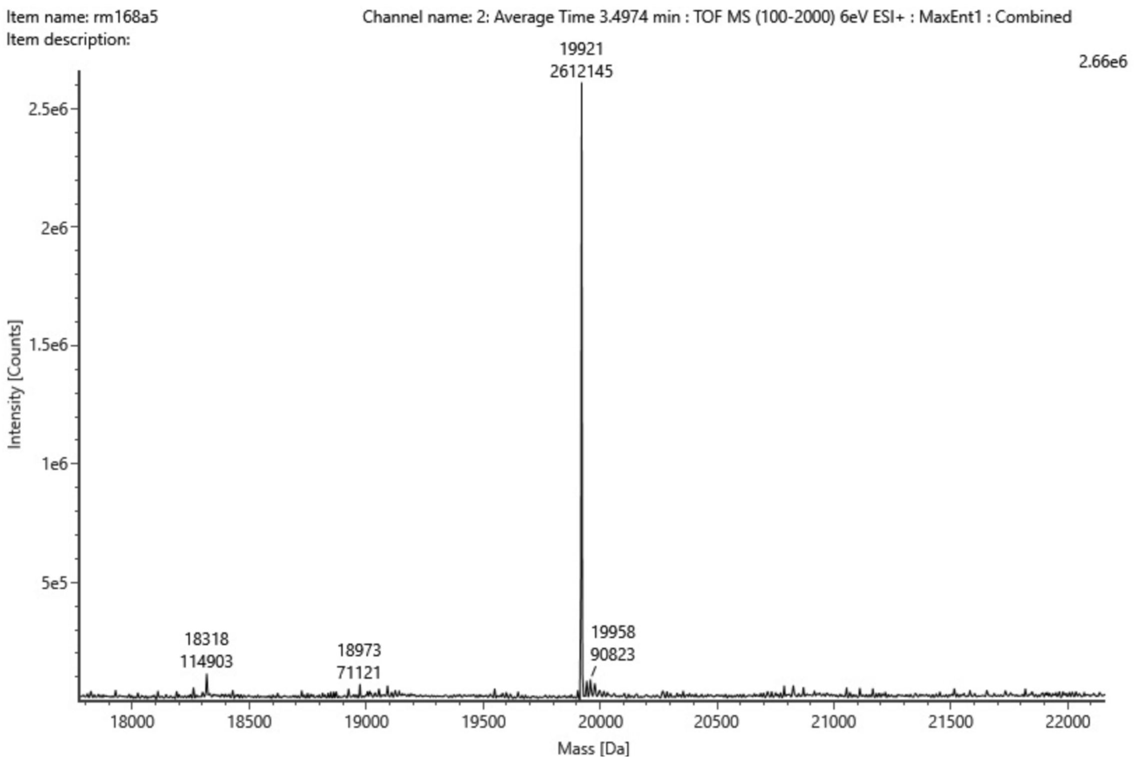

Figure S81 – Mass spectrum with intensities of compound **16** (365 Da, 150 µM) incubated at 37 ºC for 5 minutes with WT protein (19921 Da, 2.5 µM) with pH 6 buffer (20 mM HEPES, TCEP 0.5 mM, NaCl 100 mM).

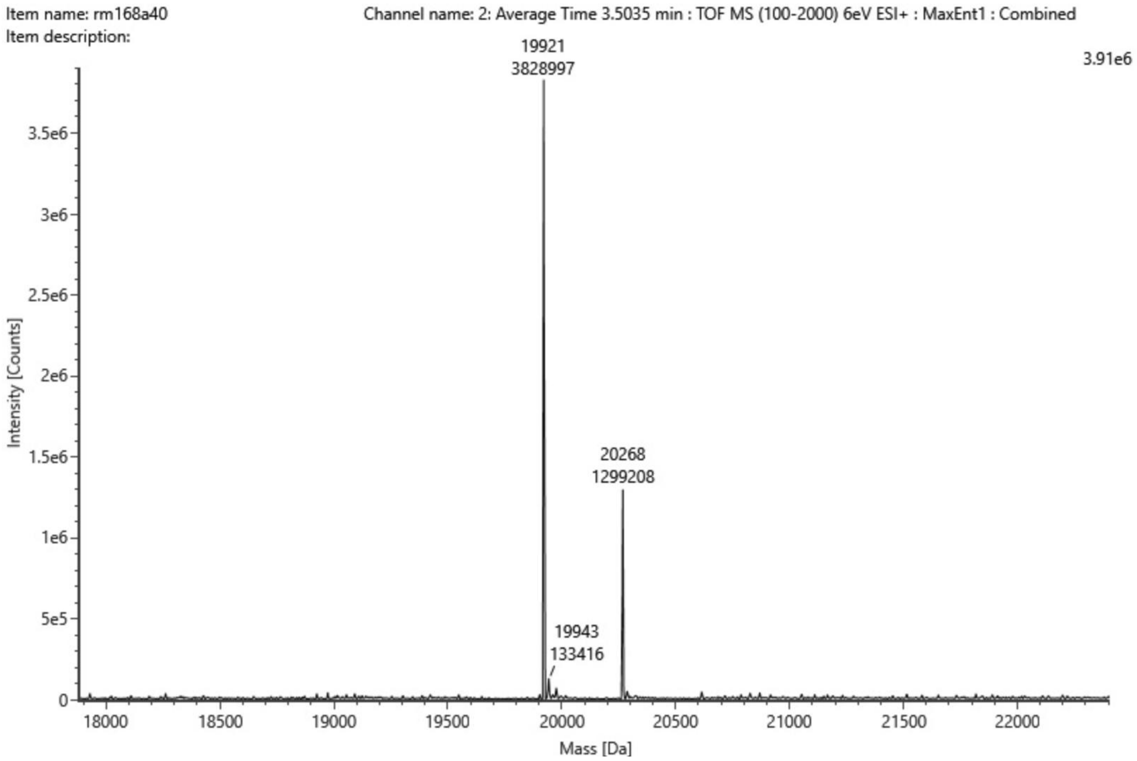

Figure S82 – Mass spectrum with intensities of compound **16** (365 Da, 150 µM) incubated at 37 ºC for 40 minutes with WT protein (19921 Da, 2.5 µM) with pH 6 buffer (20 mM HEPES, TCEP 0.5 mM, NaCl 100 mM). Covalent adduct of WT protein + **16** = 19921 + 365 – 18 = 20267 Da.

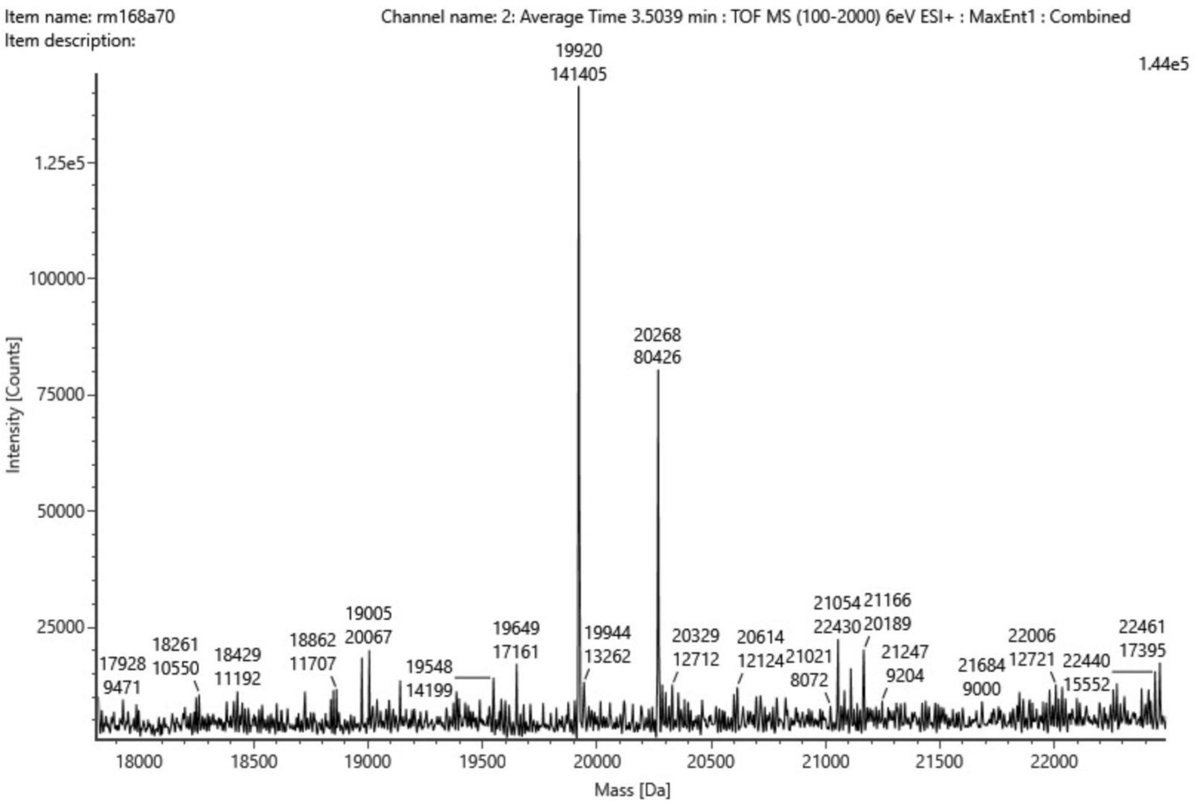

Figure S83 – Mass spectrum with intensities of compound **16** (365 Da, 150 µM) incubated at 37 ºC for 70 minutes with WT protein (19920 Da, 2.5 µM) with pH 6 buffer (20 mM HEPES, TCEP 0.5 mM, NaCl 100 mM). Covalent adduct of WT protein + **16** = 19920 + 365 – 18 = 20267 Da.

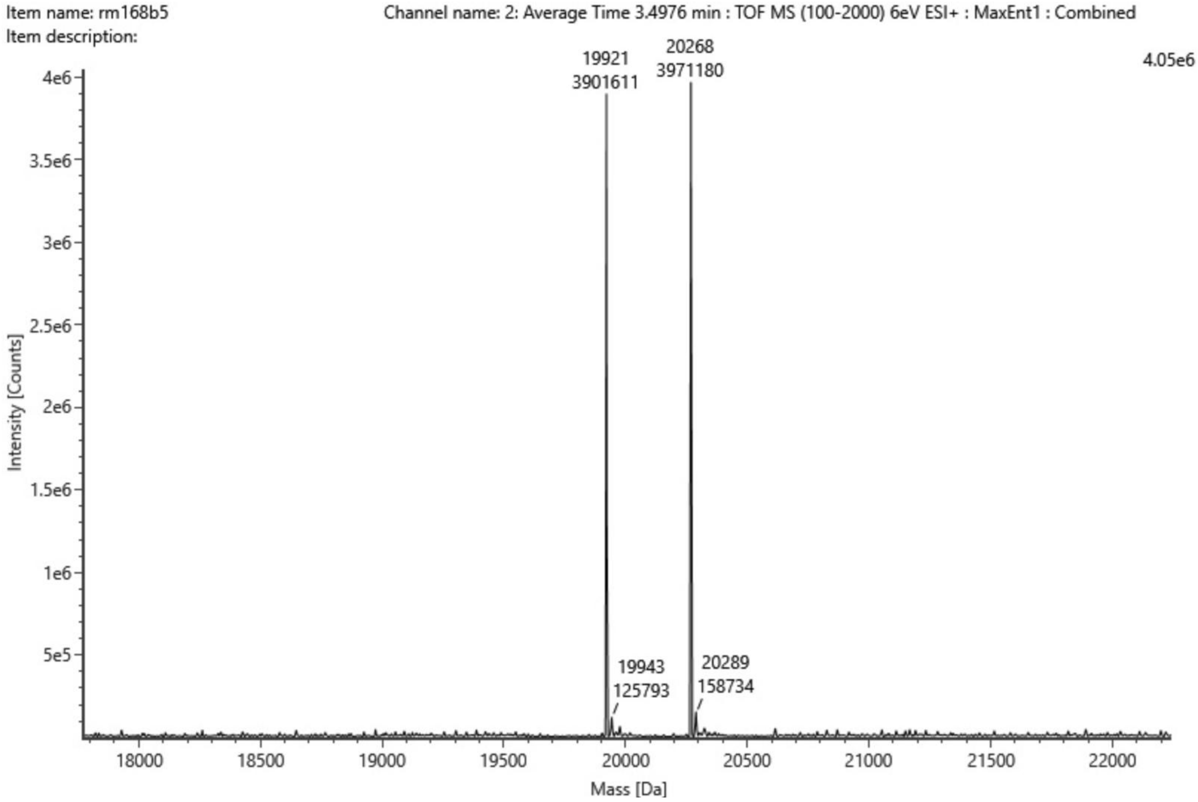

Figure S84 – Mass spectrum with intensities of compound **16** (365 Da, 150 µM) incubated at 37 ºC for 5 minutes with WT protein (19921 Da, 2.5 µM) with pH 7.4 buffer (20 mM HEPES, TCEP 0.5 mM, NaCl 100 mM). Covalent adduct of WT protein + **16** = 19921 + 365 – 18 = 20268 Da.

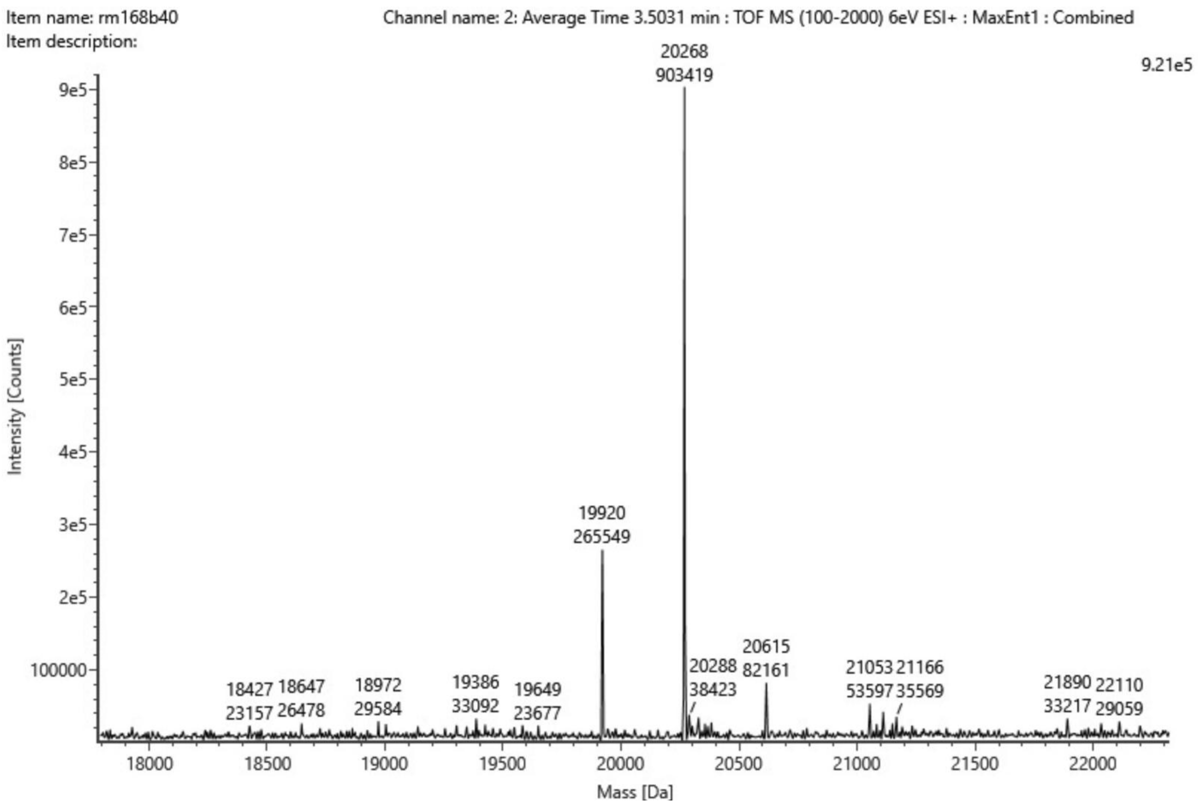

Figure S85 – Mass spectrum with intensities of compound **16** (365 Da, 150 μM) incubated at 37 ºC for 40 minutes with WT protein (19920 Da, 2.5 μM) with pH 7.4 buffer (20 mM HEPES, TCEP 0.5 mM, NaCl 100 mM). Covalent adduct of WT protein + **16** = 19920 + 365 – 18 = 20267 Da.

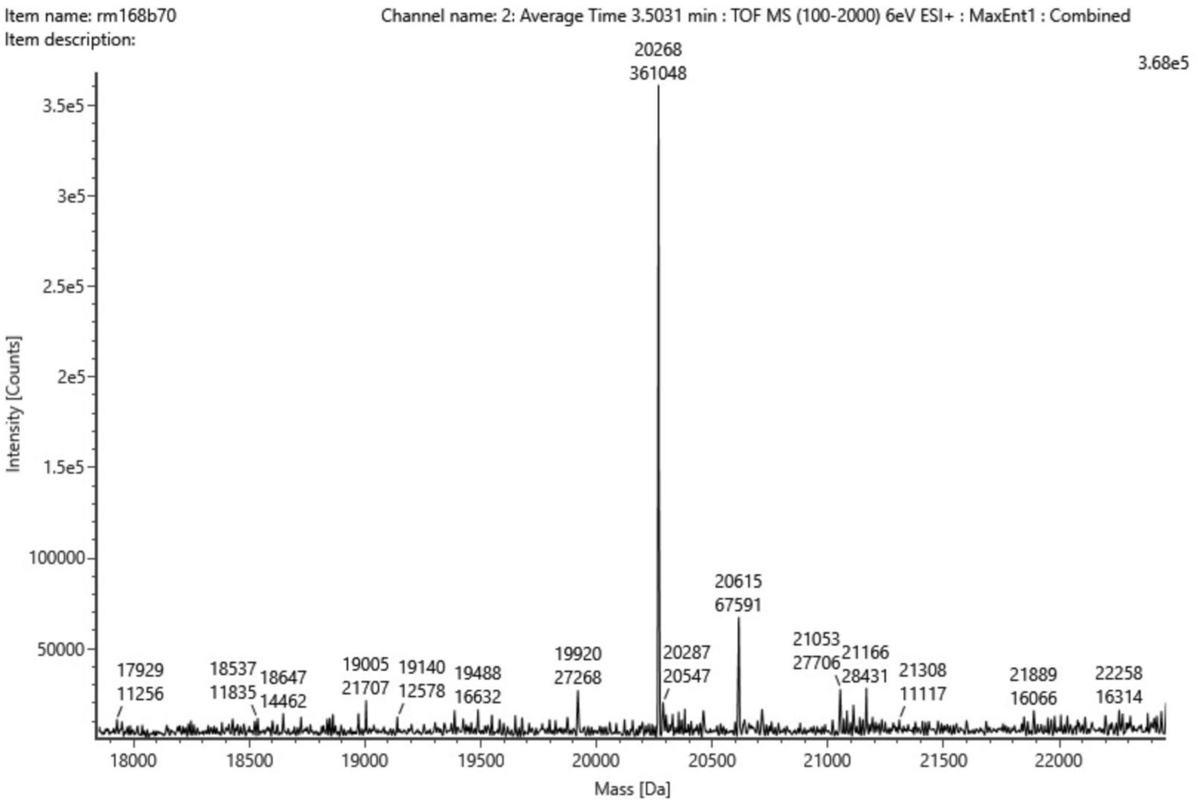

Figure S86 – Mass spectrum with intensities of compound **16** (365 Da, 150 μM) incubated at 37 ºC for 70 minutes with WT protein (19920 Da, 2.5 μM) with pH 7.4 buffer (20 mM HEPES, TCEP 0.5 mM, NaCl 100 mM). Covalent adduct of WT protein + **16** = 19920 + 365 – 18 = 20267 Da.

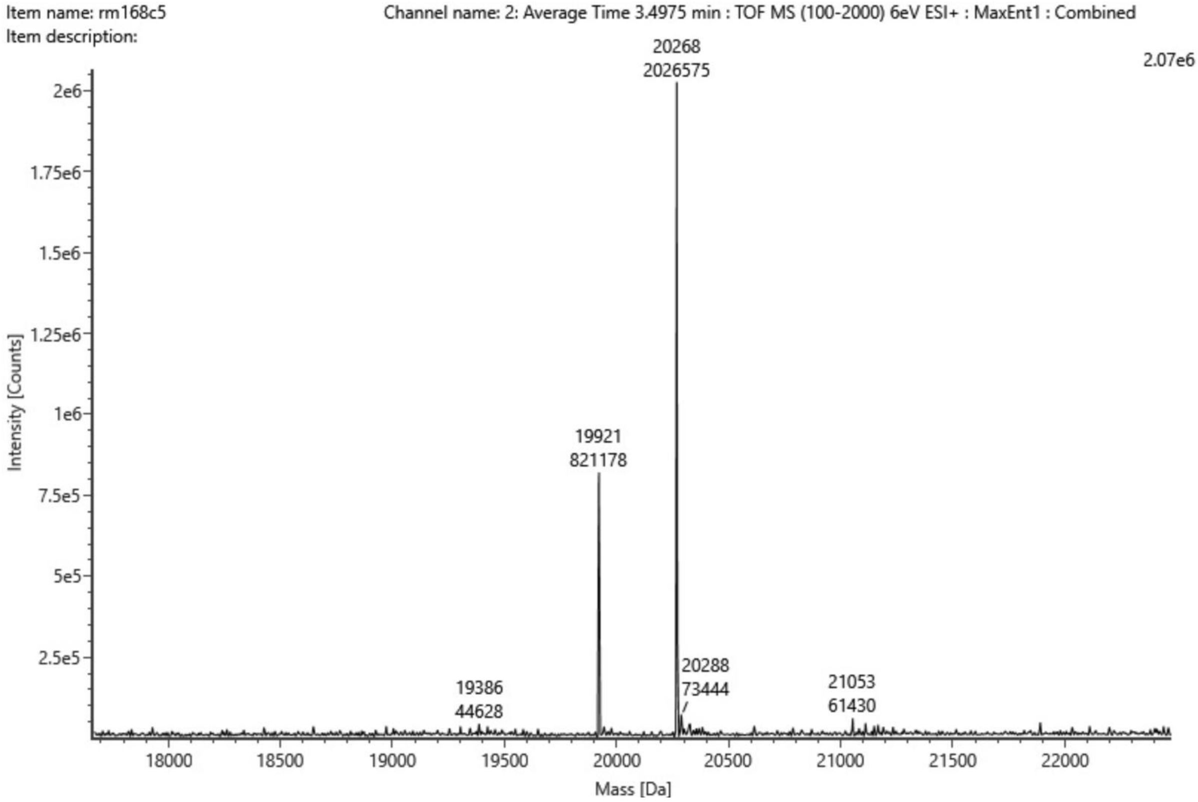

Figure S87 – Mass spectrum with intensities of compound **16** (365 Da, 150 µM) incubated at 37 ºC for 5 minutes with WT protein (19921 Da, 2.5 µM) with pH 7.8 buffer (20 mM HEPES, TCEP 0.5 mM, NaCl 100 mM). Covalent adduct of WT protein + **16** = 19921 + 365 – 18 = 20268 Da.

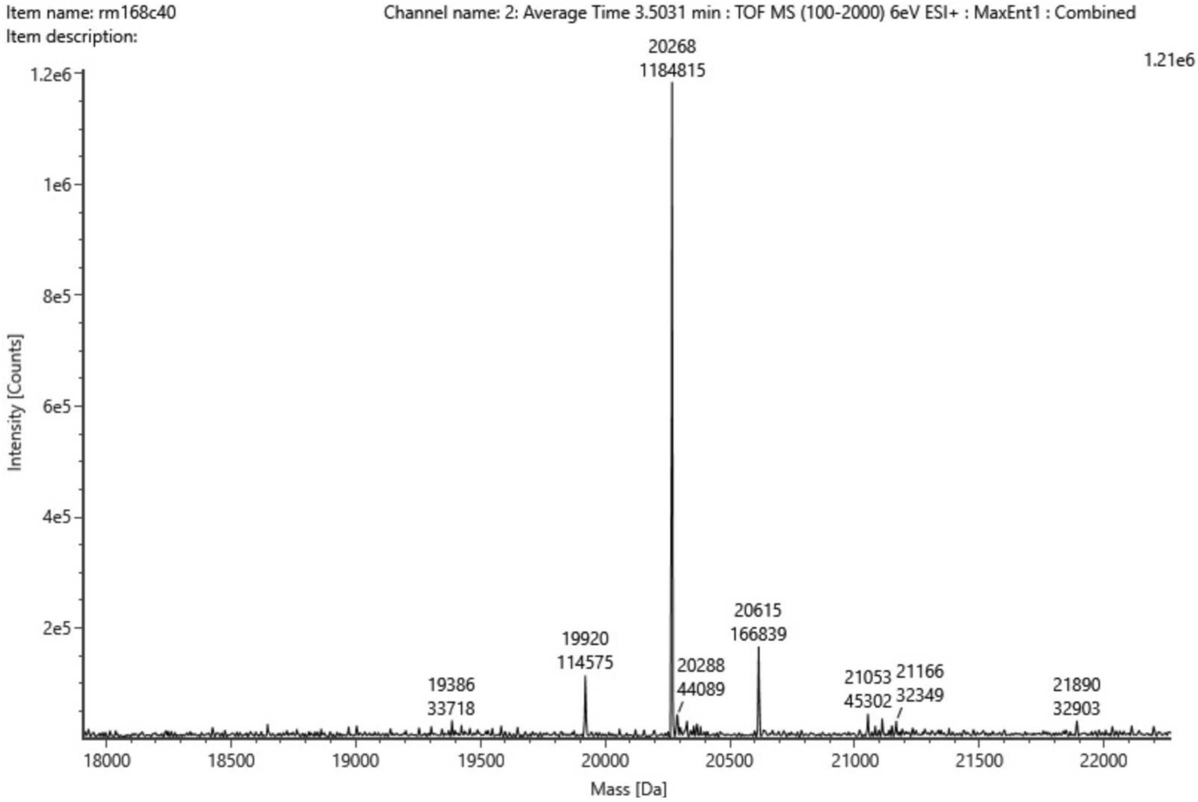

Figure S88 – Mass spectrum with intensities of compound **16** (365 Da, 150 µM) incubated at 37 ºC for 40 minutes with WT protein (19920 Da, 2.5 µM) with pH 7.8 buffer (20 mM HEPES, TCEP 0.5 mM, NaCl 100 mM). Covalent adduct of WT protein + **16** = 19920 + 365 – 18 = 20268 Da.

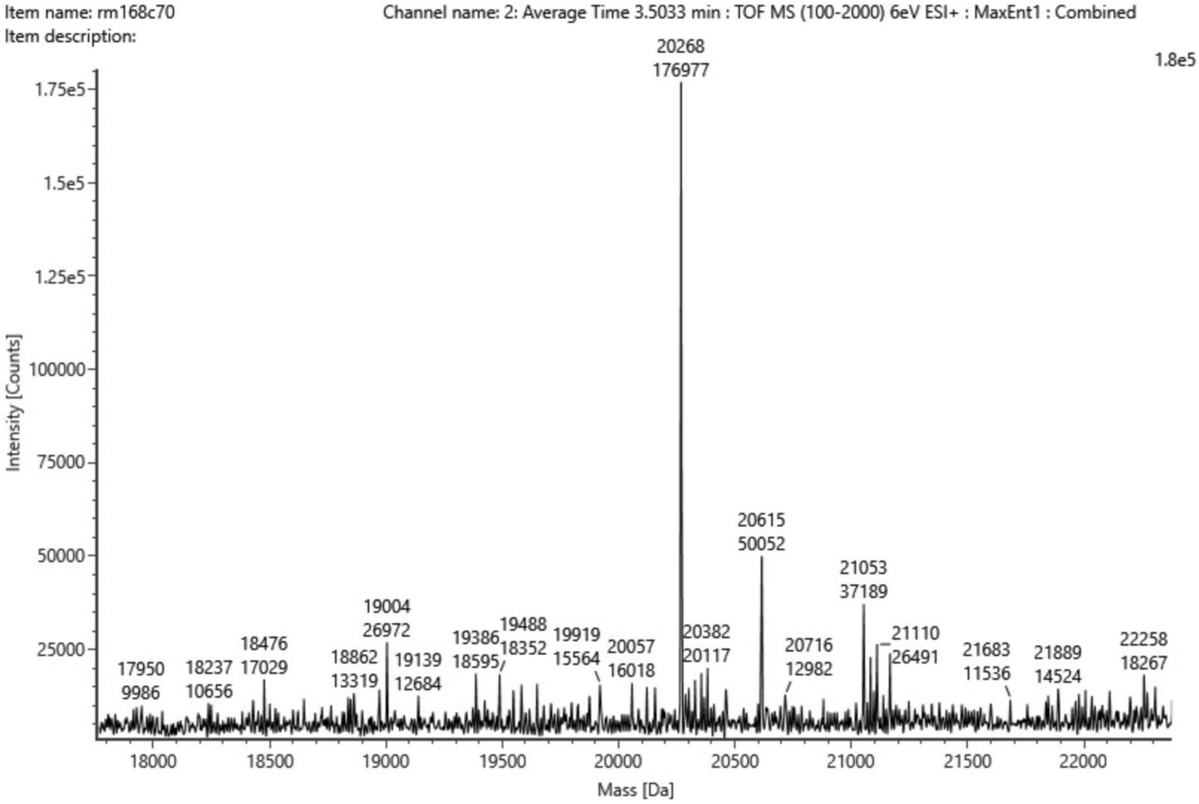

Figure S89 – Mass spectrum with intensities of compound **16** (365 Da, 150 µM) incubated at 37 ºC for 70 minutes with WT protein (19921 Da, 2.5 µM) with pH 7.8 buffer (20 mM HEPES, TCEP 0.5 mM, NaCl 100 mM). Covalent adduct of WT protein + **16** = 19921+ 365 – 18 = 20268 Da.

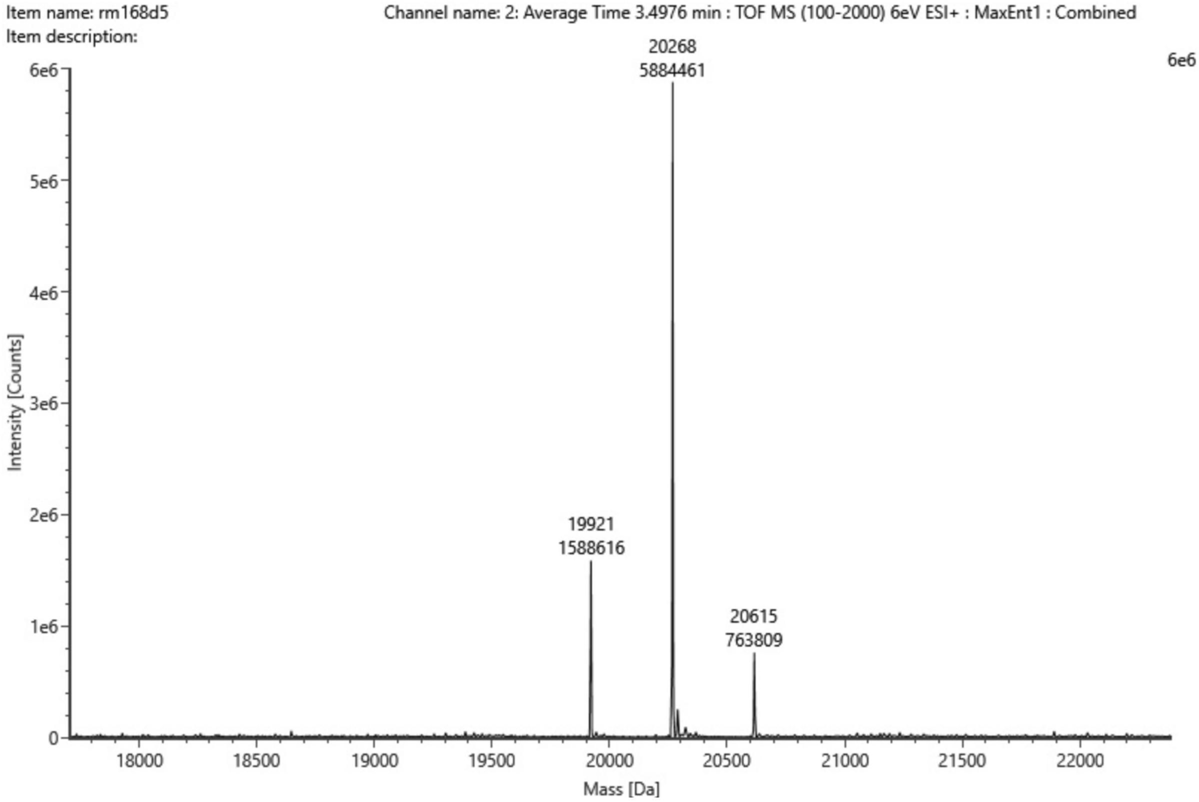

Figure S90 – Mass spectrum with intensities of compound **16** (365 Da, 150 µM) incubated at 37 ºC for 5 minutes with WT protein (19921 Da, 2.5 µM) with pH 9 buffer (20 mM HEPES, TCEP 0.5 mM, NaCl 100 mM). Covalent adduct of WT protein + **16** = 19921+ 365 – 18 = 20268 Da.

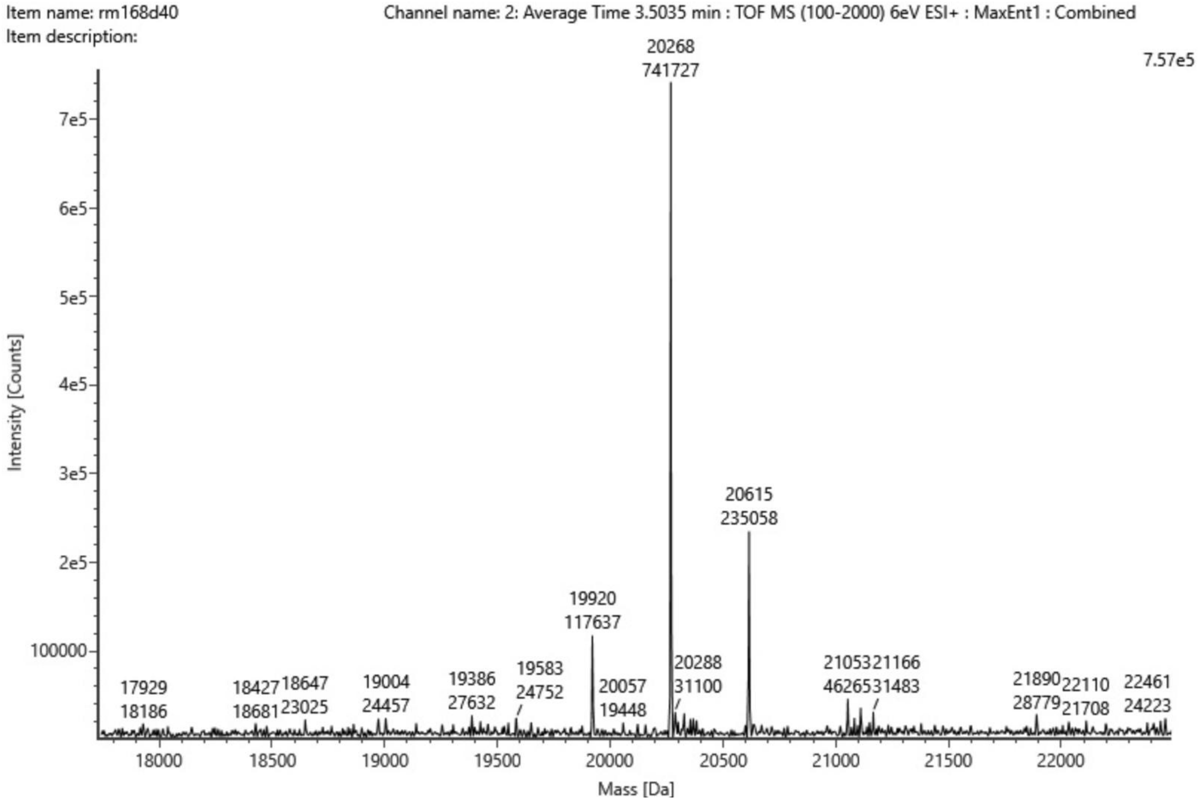

Figure S91 – Mass spectrum with intensities of compound **16** (365 Da, 150 µM) incubated at 37 ºC for 40 minutes with WT protein (19920 Da, 2.5 µM) with pH 9 buffer (20 mM HEPES, TCEP 0.5 mM, NaCl 100 mM). Covalent adduct of WT protein + **16** = 19920 + 365 – 18 = 20267 Da.

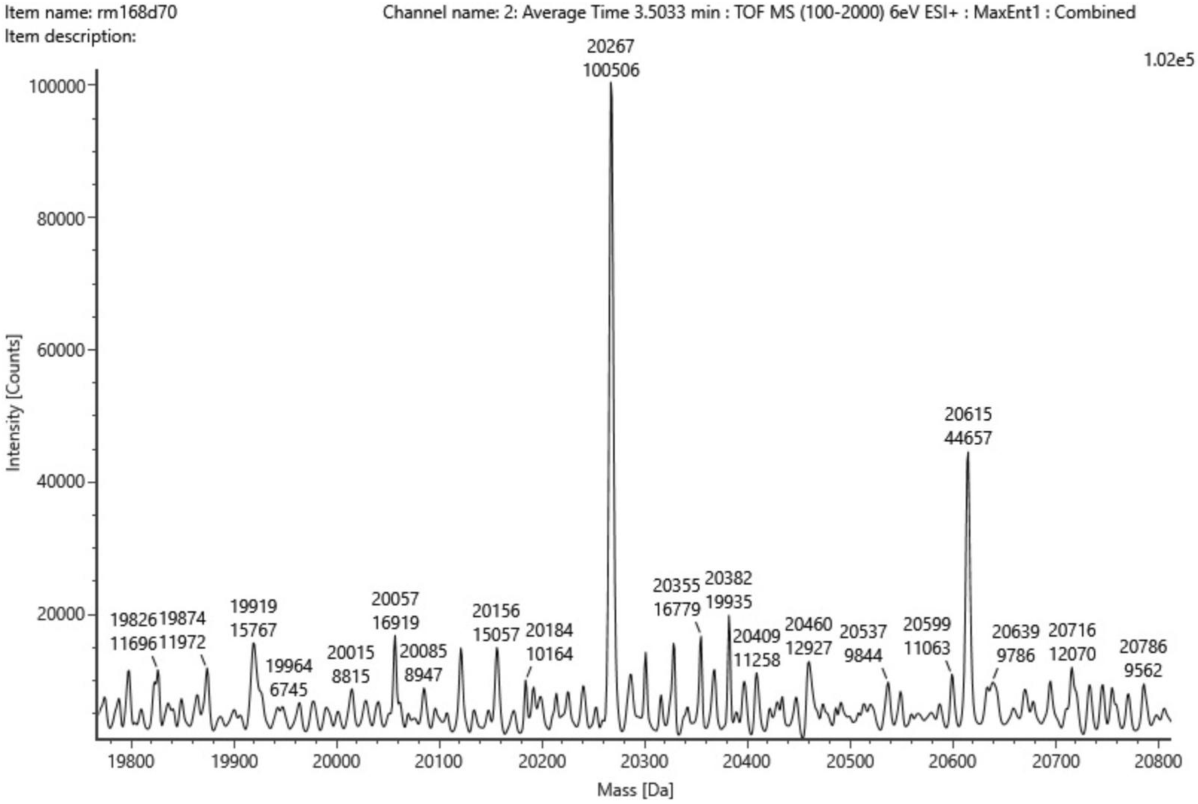

Figure S92 – Mass spectrum with intensities of compound **16** (365 Da, 150 µM) incubated at 37 ºC for 70 minutes with WT protein (19920 Da, 2.5 µM) with pH 9 buffer (20 mM HEPES, TCEP 0.5 mM, NaCl 100 mM). Covalent adduct of WT protein + **16** = 19920 + 365 – 18 = 20267 Da.

S4.4 Negative Controls - Deconvoluted Spectra

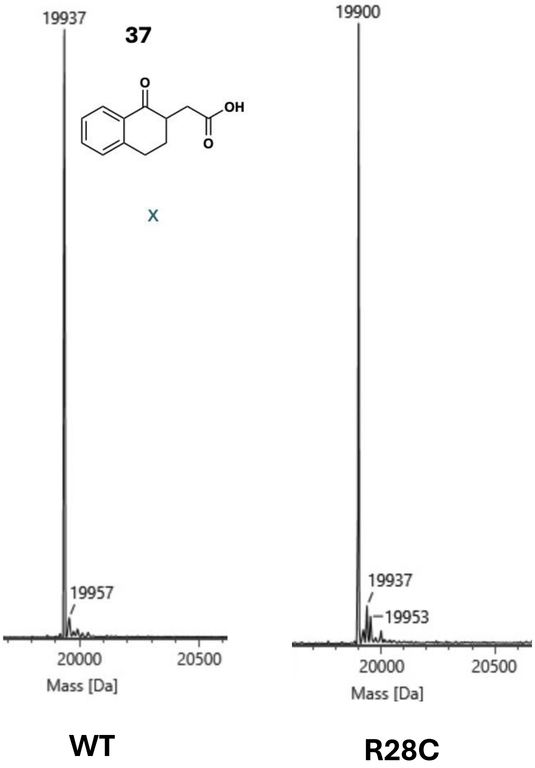

Figure S93 – Mass spectrum of compound **37** (204 Da, 150 µM) incubated at 37 °C for 5 minutes with WT protein (19936 Da, 2.5 µM) and R28C mutant (19900 Da, 2.5 µM) in pH 8 buffer (20 mM HEPES, TCEP 0.5 mM, NaCl 100 mM).

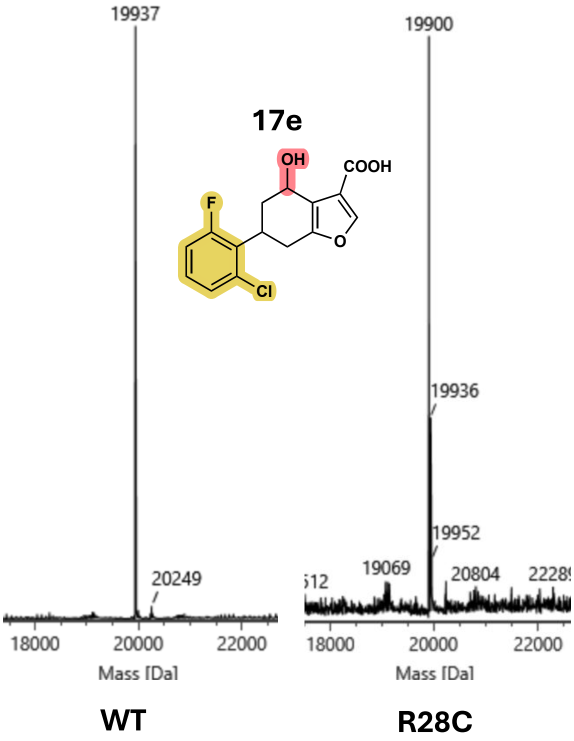

Figure S94 – Mass spectrum of compound **17e** (311 Da, 150 µM) incubated at 37 °C for 5 minutes with WT protein (19937 Da, 2.5 µM) and R28C mutant (19900 Da, 2.5 µM) in pH 8 buffer (20 mM HEPES, TCEP 0.5 mM, NaCl 100 mM). Non-covalent adduct with WT = 19937 + 311 = 20249 Da.

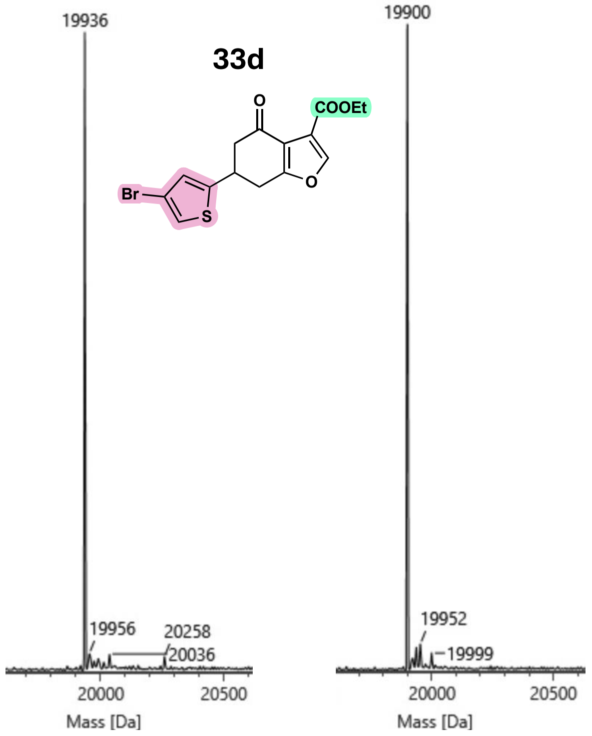

WT **R28C**

Figure S95 – Mass spectrum of compound **29** (369 Da, 150 µM) incubated at 37 ºC for 5 minutes with WT protein (19936 Da, 2.5 µM) and R28C mutant (19900 Da, 2.5 µM) in pH 8 buffer (20 mM HEPES, TCEP 0.5 mM, NaCl 100 mM). Not observed: covalent adduct with WT = 19936 + 369 – 18 = 20287 Da, non-covalent adduct with WT = 19936 + 369 = 20305 Da.

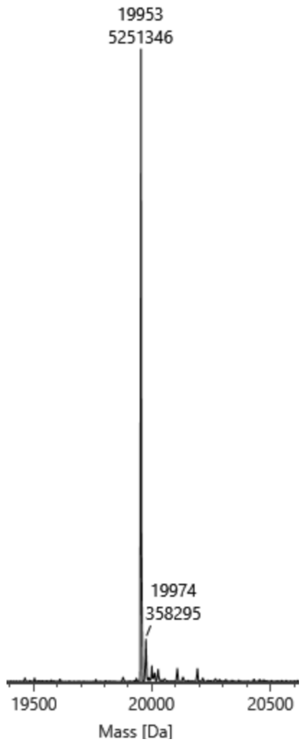

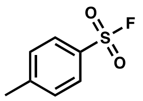

38

WT

Figure S96 – Mass spectrum with intensities of compound **38** (174 Da, 150 µM) incubated at 37 ºC for 5 minutes with WT protein (19953 Da, 2.5 µM) in pH 8 buffer (20 mM HEPES, TCEP 0.5 mM, NaCl 100 mM). Not observed: covalent adduct with WT = 19953 + 174 – 19 = 20108 Da.

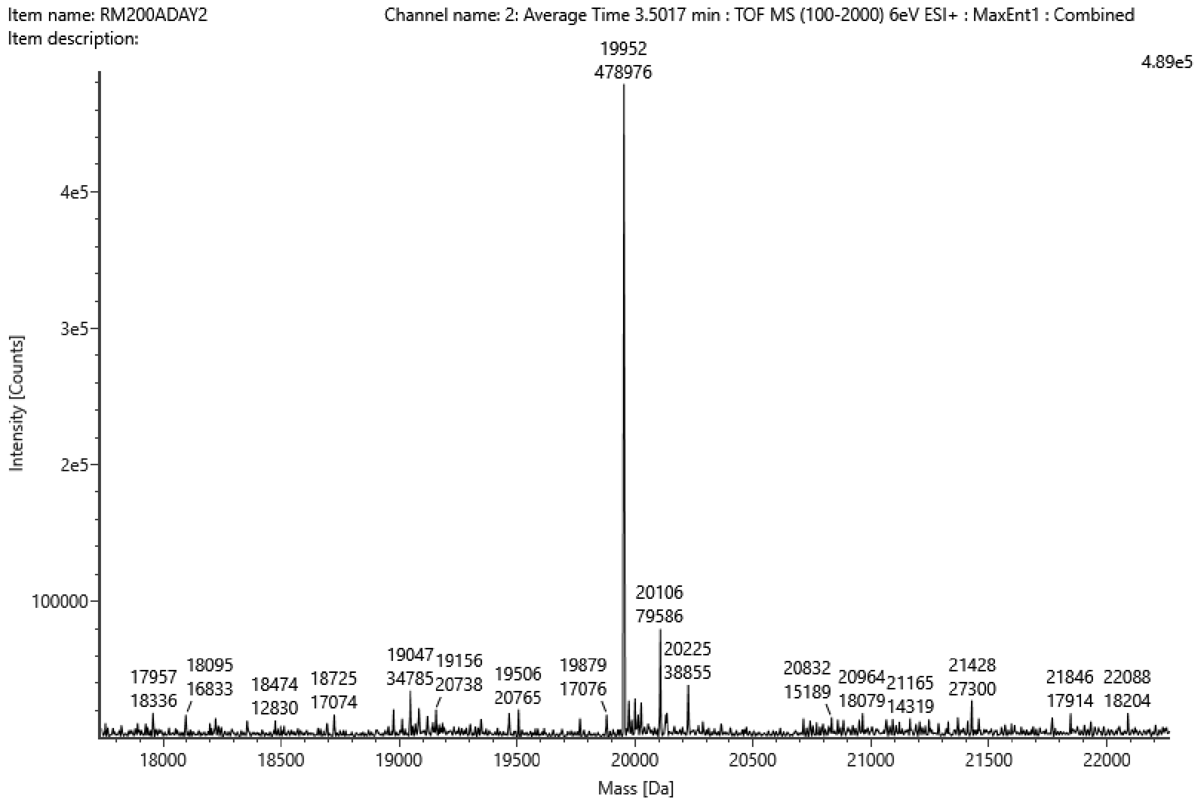

Figure S97 – Mass spectrum with intensities of compound **38** (174 Da, 1 mM) incubated at 37 ºC for 5 minutes and then at RT overnight with WT protein (19952 Da, 2.5 µM) in pH 8 buffer (20 mM HEPES, TCEP 0.5 mM, NaCl 100 mM). Observed: covalent adduct with WT = 19952 + 174 – 19 = 20107 Da.

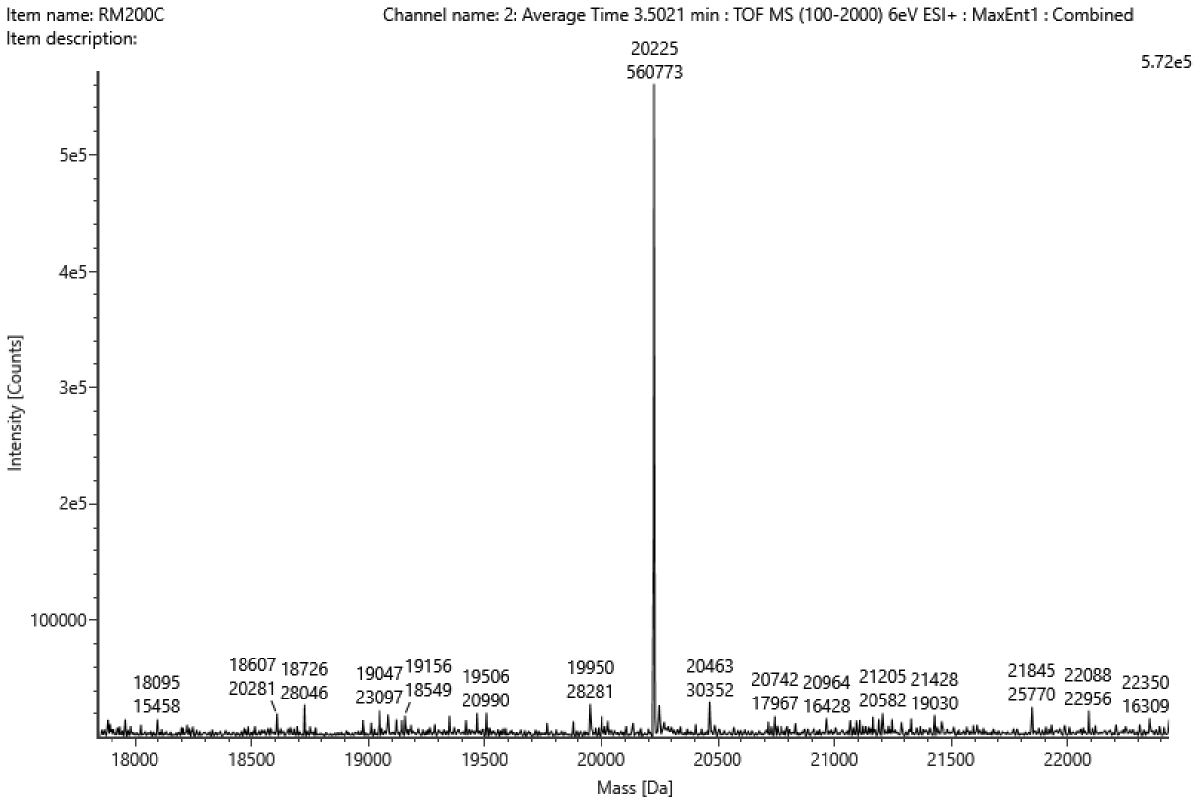

Figure S98 – Mass spectrum with intensities of compound **24** (291 Da, 150 µM) incubated at 37 ºC for 5 minutes and then at RT overnight with WT protein (19952 Da, 2.5 µM) in pH 8 buffer (20 mM HEPES, TCEP 0.5 mM, NaCl 100 mM). Covalent adduct with WT = 19952 + 291 – 18 = 20225 Da..

S4.5 Enantiomerically Pure Ligands

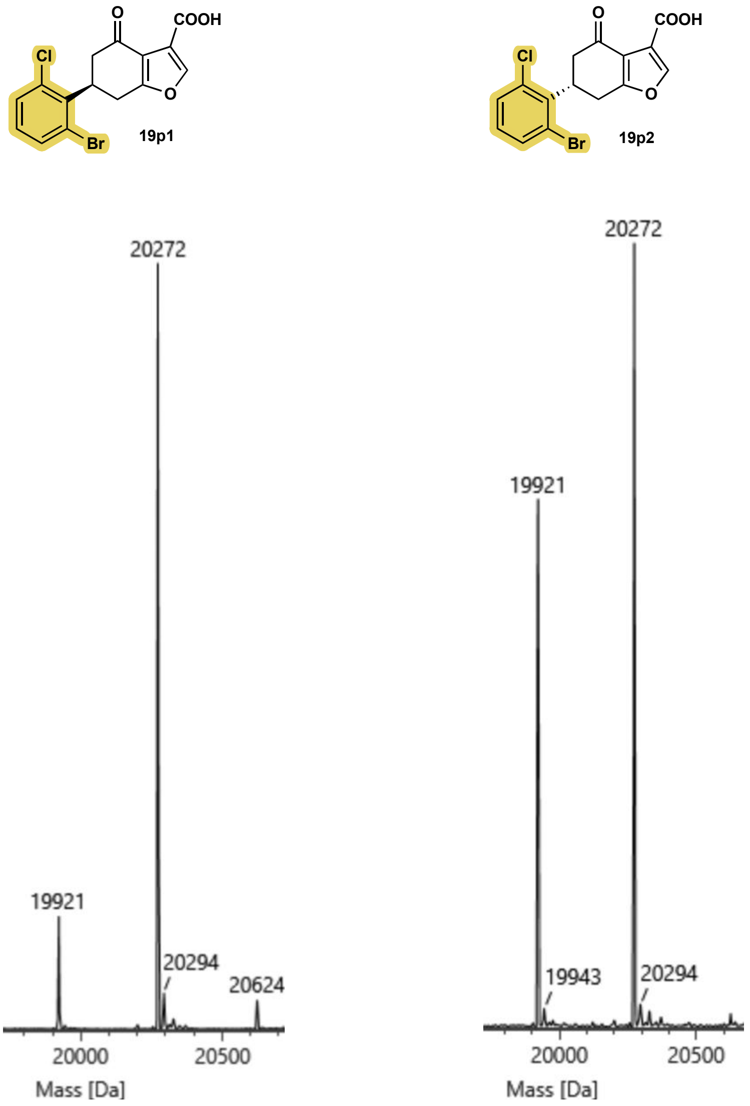

Figure S99 – Mass spectra of enantiomerically pure compounds **19p1** and **19p2** (369 Da, 150 µM) incubated at 37 ºC for 5 minutes with WT protein (19921 Da, 2.5 µM) in pH 8 buffer (20 mM HEPES, TCEP 0.5 mM, NaCl 100 mM). Covalent adduct with WT = 19921 + 369 – 18 = 20272 Da.

S5 MALDI-TOF

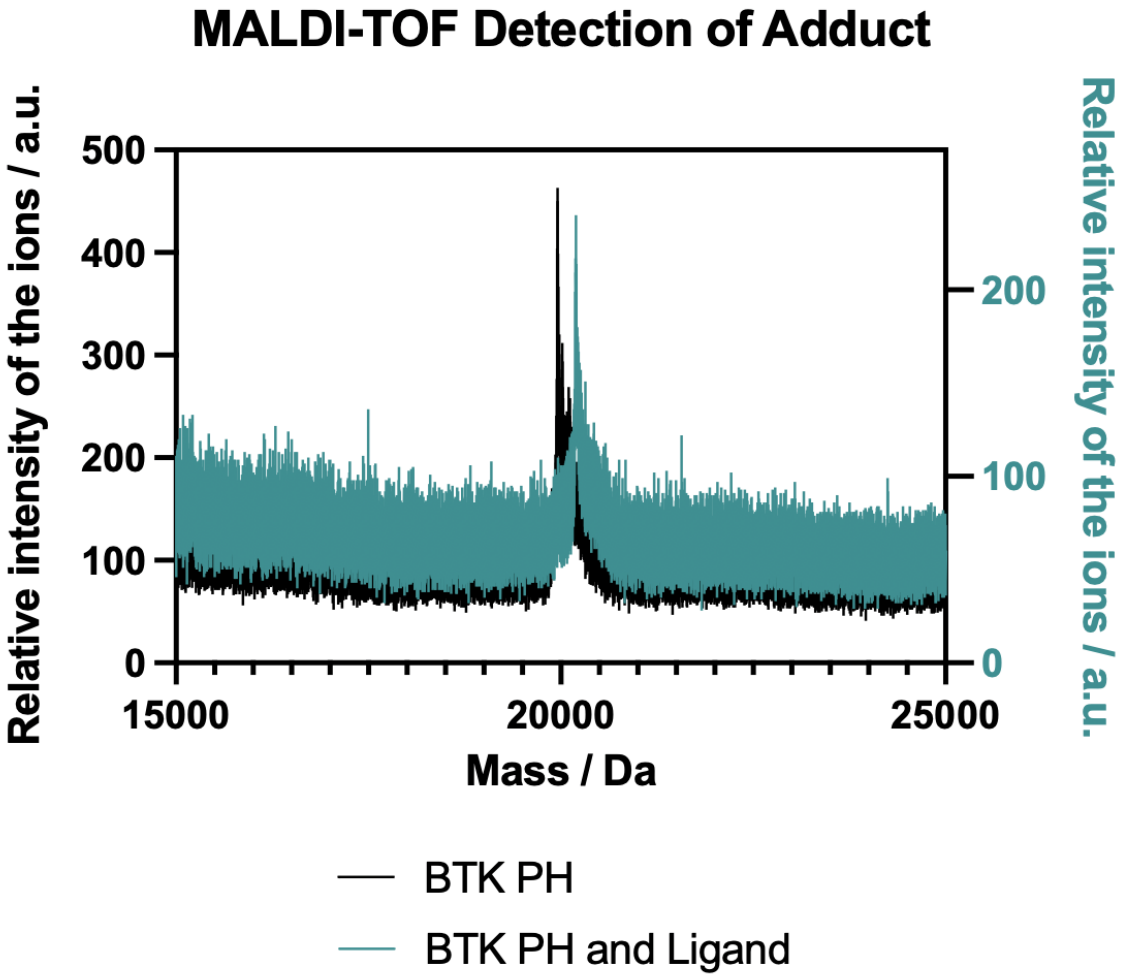

Figure S100 – Matrix Assisted Laser Desorption/Ionization – Time of Flight results with BTK PH domain alone (black) and BTK PH domain with compound **2** (teal). Covalent adduct with WT = 19957 + 256 – 18 = 20195 Da.

S6 Differential Scanning Fluorimetry (DSF) Studies

S6.1 Raw Data for Figure 9

Table S4 – A table to show WT protein (5 µM) T_m values obtained in DSF with parent phenyl, **2**, the ortho fluoro-chloro analogue, **17**, and its reduced counterpart **17e** at varying ligand concentration.

**Conc/ µM**	**T_m values (°C) for Analogue**		
	**2**	**17e**	**17**
**3000**	51.5		59.0
**1500**	51.5	37.0	58.5
**750**	51.0	37.5	57.0
**375**	50.0	38.0	55.5
**187.5**	48.0		53.0
**93.75**	46.0	40.0	51.0
**46.88**	45.0	41.5	49.5
**23.44**	44.5	42.0	47.5
**11.72**	44.0	42.5	45.5
**5.86**	43.5	42.5	44.0
**2.93**	43.5	42.5	44.0
**1.46**	43.5	42.0	43.5
**0.73**	43.0	42.5	43.0
**0.10**	44.0	42.5	43.0

S6.2 Supplementary Figures S101A and S101B

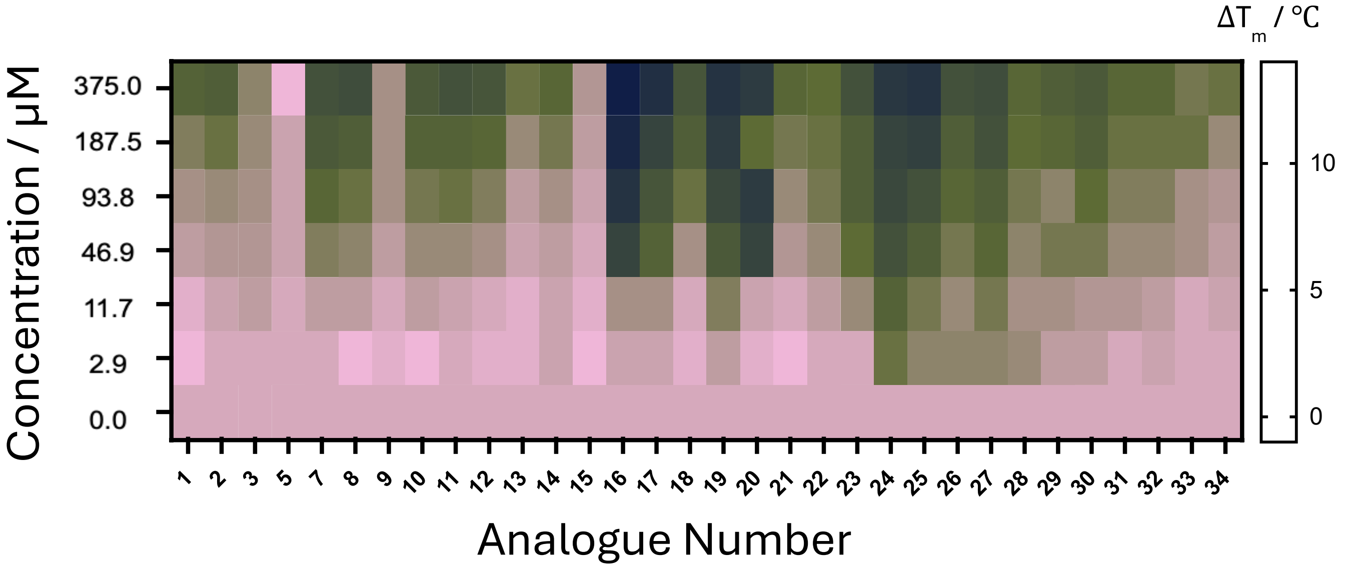

Figure S101A - Differential Scanning Fluorimetry with WT (5 μM).

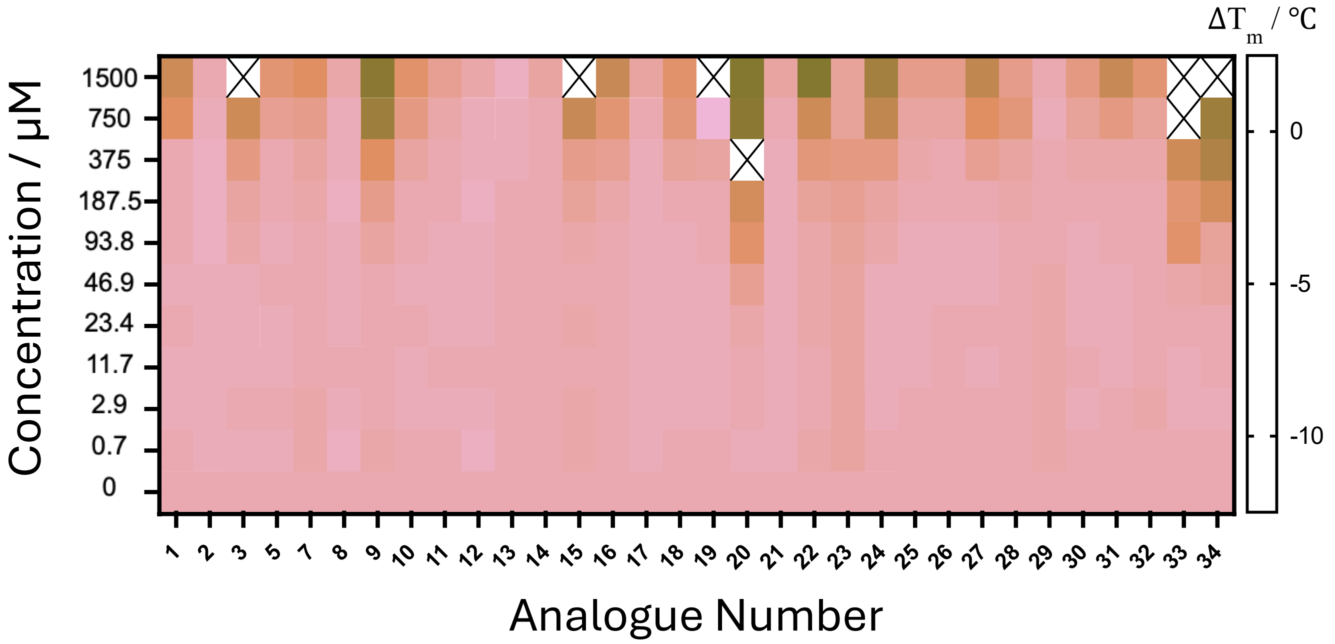

Figure S101B - Differential Scanning Fluorimetry with R28C Mutant (5 μM). Conditions marked as “X” showed now clear melting curve.

S6.3 Raw data for Supplementary Figures S101A and S101B

Table S5 – Raw Data used to calculate Δ T_m for Supp. Figure S101A- T_m values (°C) from DSF with WT (5 µM)

Conc/ µM	T_m values (°C) for Analogue																															
	**1**	**2**	**3**	**5**	**7**	**8**	**9**	**10**	**11**	**12**	**13**	**14**	**15**	**16**	**17**	**18**	**19**	**20**	**21**	**22**	**23**	**24**	**25**	**26**	**27**	**28**	**29**	**30**	**31**	**32**	**33**	**34**
**3000**		51.5	45.5	39.5	52.5	55.5		51.0	53.0	56.5	55.0	53.5		59.0	59.0		55.5		52.0	43.0	54.0											
**1500**		51.5	45.5	42.0	52.5	55.0		51.5	52.5	55.5	52.0	52.5		59.5	58.5	53.0	57.0		51.5	46.0	52.5	55.5	56.0	53.5		49.0	55.0		51.5	52.5		
**750**		51.0	46.0	44.0	53.0	53.5	44.0	51.5	52.0	53.0	50.0	51.0	45.5	58.0	57.0	52.5	57.0	53.0	50.0	48.0	52.0	55.5	55.0	52.5		49.5	54.0	54.5	52.5	53.0		50.0
**375**	49.5	50.0	46.5	42.5	51.5	52.0	45.5	50.5	51.5	51.0	48.0	49.0	45.0	57.5	55.5	51.0	55.0	54.0	49.0	48.5	51.5	54.5	55.0	51.5	52.0	49.0	53.5	54.0	52.5	52.5	51.0	51.5
**187.5**	47.0	48.0	46.0	44.0	50.5	50.0	45.5	49.5	49.5	49.0	46.0	47.5	44.5	56.5	53.0	50.0	54.0	48.5	47.5	48.0	50.0	53.0	53.5	50.0	51.5	48.5	52.5	53.5	51.5	51.5	51.5	49.5
**93.75**	45.5	46.0	45.5	44.0	49.0	48.0	45.5	47.5	48.0	47.0	44.5	45.5	44.0	55.0	51.0	48.0	52.5	54.0	46.0	47.5	50.0	52.5	51.5	49.0	50.0	47.5	50.0	52.0	50.5	50.5	49.0	48.5
**46.88**	44.5	45.0	45.0	44.0	47.0	46.5	44.5	46.0	46.0	45.5	44.0	44.5	43.5	53.0	49.5	45.5	50.5	53.0	45.0	46.0	48.5	51.5	50.0	47.5	49.0	46.5	51.0	51.0	49.5	49.5	49.0	48.0
**23.44**	44.0	44.5	44.5	44.0	45.5	45.0	44.5	45.0	45.0	45.5	43.5	44.5	43.5	50.0	47.5	44.5	49.5	48.0	44.0	45.0	47.0	50.5	49.0	47.0	48.0	46.0	50.5					
**11.72**	43.0	44.0	44.5	43.5	44.5	44.5	43.5	44.5	44.0	43.5	43.0	44.0	43.0	45.5	45.5	43.5	47.0	44.0	43.5	44.5	46.0	49.5	47.5	46.0	47.5	45.5	49.0	48.5	48.5	48.0	47.0	47.5
**5.86**	43.0	43.5	43.5	43.5	43.5	43.5	43.5	43.5	44.0	43.0	43.0	44.0	43.0	44.0	44.0	43.0	46.0	43.5	43.0	43.5	44.0	48.5										
**2.93**	42.5	43.5	43.5	43.5	43.5	42.5	43.0	42.5	43.5	43.0	43.0	44.0	42.5	44.0	44.0	43.0	44.5	43.0	42.5	43.5	43.5	48.0	46.5	46.5	46.5	46.0	48.0	48.0	47.0	47.5	47.0	47.0
**1.46**	42.5	43.5	43.0	43.5	43.0	43.0	43.0	42.5	44.0	42.5	43.0	43.5	43.0	43.5	43.5	43.0	43.5	43.0	42.5	43.0	43.5	47.5										
**0.73**	42.5	43.0	43.5	43.0	43.0	43.0	43.0	42.5	43.0	42.5	42.5	44.0	42.5	43.0	43.0	43.0	44.5	43.0	42.5	43.0	43.0	46.5	46.5	46.0	46.0	46.0	47.5			47.5	47.0	
**0.00**		44.0							43.5			43.5		43.5				43.0				46.0		43.0	44.5		47.0					

Table S6 – Raw Data used to calculate Δ T_m for Supp. Figure S101B - T_m values (°C) from DSF with Mutant (5 μM)

Conc/ μM	T_m values (°C) for Analogue																														
	**1**	**2**	**3**	**5**	**7**	**8**	**9**	**10**	**11**	**12**	**13**	**14**	**15**	**16**	**17**	**18**	**19**	**20**	**21**	**22**	**23**	**24**	**25**	**26**	**27**	**28**	**29**	**30**	**31**	**32**	**33**	**34**
**3000**	41.5	49.0	41.5	42.0	40.5	47.0	36.5	41.5	45.0	47.0	49.0	45.0		38.0	44.0			32.5	45.5		50.5	41.5	43.0	44.5			49.5		40.5			
**1500**	42.5	49.5		45.5	44.5	48.5	37.0	44.5	47.0	48.5	50.5	48.5		42.5	47.5	44.5		36.5	47.5	37.0	50.5	40.5	48.0	48.0	43.0	48.0	50.0	47.5	43.5	46.5		
**750**	44.0	50.0	42.5	47.5	47.0	49.5	38.5	46.0	48.5	49.5	50.0	49.5	42.5	45.5	48.5	45.5	53.0	37.0	48.5	43.0	50.5	43.0	49.5	49.5	45.5	47.0	50.5	49.0	47.5	49.0		40.0
**375**	49.0	50.5	46.0	49.5	48.5	49.5	44.0	48.0	49.0	49.5	50.0	49.5	47.0	47.5	49.0	47.5	49.5	27.0	49.0	46.0	49.0	47.5	50.0	50.5	48.5	49.5	50.0	50.0	50.0	50.0	44.0	41.5
**187.5**	49.0	50.5	48.0	49.5	49.0	50.0	46.5	49.0	49.0	50.0	49.5	49.5	48.0	49.0	49.0	49.0	50.5	43.0	49.0	48.0	50.0	49.5	50.5	50.5	50.5	50.0	50.0	50.5	50.5	50.5	46.5	44.5
**93.75**	49.0	50.5	48.5	50.0	49.5	49.5	48.0	49.0	49.5	49.5	49.5	49.5	49.0	49.5	49.0	49.5	50.5	44.5	49.0	49.0	50.5	50.0	51.0	51.0	51.0	50.5	50.0	51.0	50.5	50.5	46.0	49.0
**46.88**	49.5	50.0	49.5	49.5	49.5	49.5	49.0	49.5	49.5	49.5	49.5	49.5	49.5	49.5	49.0	49.5	51.0	47.0	49.0	49.0	51.0	51.0	51.0	51.0	51.0	50.5	49.5	51.0	51.0	50.5	50.0	49.5
**23.44**	49.0	50.0	49.5	50.0	49.5	49.5	49.0	49.0	49.5	49.5	49.5	49.5	49.0	49.5	49.0	49.5	51.0	48.5	49.0	49.0	51.0	51.0	51.0	50.5	50.5	50.5	49.5	51.0	51.0	50.5	50.5	50.5
**11.72**	49.5	50.0	49.5	50.0	49.5	49.0	49.0	49.5	49.0	49.0	49.5	49.5	49.5	49.5	49.0	49.5	51.0	49.0	49.0	49.5	51.0	51.0	51.0	50.5	51.0	50.5	49.5	50.5	51.0	50.5	51.0	50.5
**5.86**	49.5	50.0	49.5	50.0	49.0	49.5	49.0	49.5	49.5	49.5	49.5	49.5	49.5	49.5	49.0	49.0		49.0	49.0	49.0							49.5	50.5	50.5	50.5	50.5	50.5
**2.93**	49.5	50.0	49.0	49.5	49.0	49.5	49.0	49.5	49.5	49.5	49.5	49.5	49.0	49.5	49.0	49.5	51.0	49.0	49.0	49.5	51.0	51.0	50.5	50.5	50.5	50.5	49.5	51.0	50.5	50.0	51.0	51.0
**1.46**	49.0	50.0	49.0	49.5	49.0	49.5	49.0	49.5	49.0	49.5	49.5	49.5	49.0	49.5	49.0	49.5		49.0	49.0	49.0							49.5			50.5		
**0.73**	49.0	50.0	49.5	50.0	49.0	50.0	48.5	49.0	49.0	50.0	49.5	49.5	49.0	49.5	49.0	49.0	50.5	49.5	49.0	49.0	51.0	50.5	50.5	50.5	50.5	50.5	49.5	50.5	50.5	50.5	50.5	50.5
**0.00**	49.0	49.5	49.0	49.5	49.5	49.0	49.0	49.0		49.0	49.5	49.5	49.5	49.5	48.5	49.0	50.5	49.0	48.5	49.5	52.0	50.5	50.5	50.5	50.5	50.5	50.0	50.5	50.5	50.5	50.5	50.5

Table S7 – Fitting DSF data to $Y = B_{max}*X/(K_d + X)$ using Prism 10.

**Analogue**	**B_{max}**	**K_d / μM**	**R^2**	**log(K_d in μM) from DSF**
1	18.4	795.8	0.98	2.90
2	9.0	190.7	0.99	2.28
3	2.5	29.2	0.90	1.47
4	No data			
5	Undetermined			
6	No data			
7	9.8	81.3	0.99	1.91
8	12.6	175.3	0.99	2.24
9	1.7	30.8	0.66	1.49
10	8.5	99.6	0.97	2.00
11	9.9	121.6	0.99	2.08
12	14.2	312.9	0.98	2.50
13	15.1	1015.0	0.98	3.01
14	11.3	375.9	0.99	2.58
15	4.1	731.9	0.70	2.86
16	15.9	41.5	0.99	1.62
17	15.5	92.9	0.99	1.97
18	10.8	156.8	0.98	2.20
19	13.0	33.9	0.99	1.53
20	10.4	30.8	0.82	1.49
21	9.3	270.1	0.97	2.43
22	5.4	51.9	0.96	1.71
23	9.4	47.3	0.98	1.67
24	10.5	5.8	0.88	0.76
25	11.5	25.8	0.90	1.41
26	8.6	38.3	0.81	1.58
27	7.8	10.6	0.85	1.02
28	5.6	24.0	0.70	1.38
29	6.7	34.5	0.91	1.54
30	7.9	47.7	0.99	1.68
31	5.4	44.6	0.96	1.65
32	6.2	65.3	0.99	1.82
33	5.7	112.6	0.93	2.05
34	4.4	130.8	0.87	2.12

S7 Statistics

S7.1 Normality Tests

Table S8 - Results of the Shapiro-Wilk test performed on the log(K_d) values approximated from DSF.

**Approximate log(K_d) from DSF, Shapiro-Wilk test**	
W	0.9702
P value	0.5245
Passed normality test (alpha=0.05)?	Yes
P value summary	ns

Table S9 - Results of the Shapiro-Wilk test performed on the total labelling relative intensity values from mass spectrometry.

**Total Labelling Relative Intensity from MS, Shapiro-Wilk test**	
W	0.9406
P value	0.0855
Passed normality test (alpha=0.05)?	Yes
P value summary	ns

S7.2 Correlation Tests

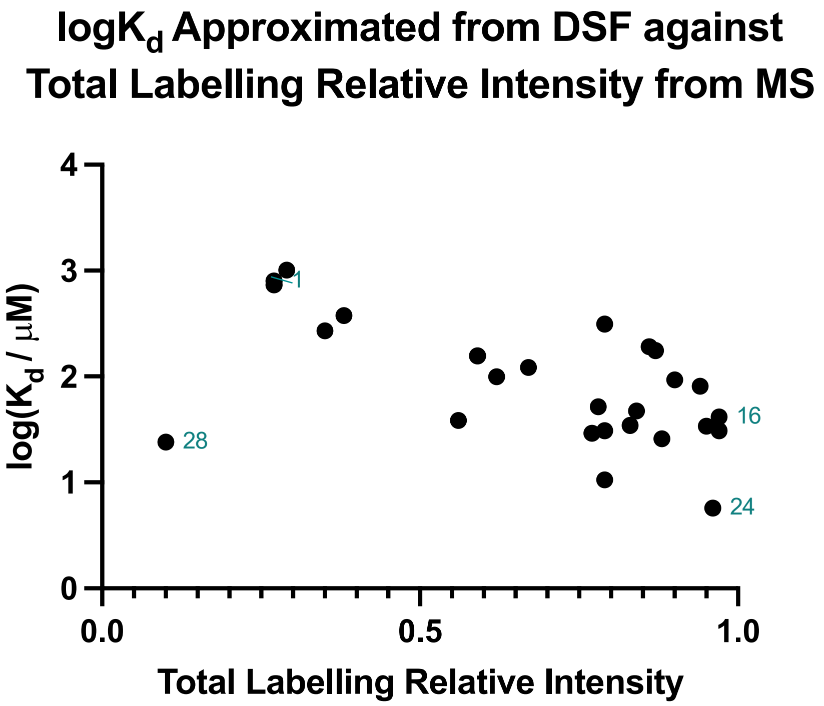

Figure S102 – A graph to show log(K$_d$) values approximated from DSF against Total Labelling Relative Intensity values from mass spectrometry. Pearson r = -0.56, 95 % confidence interval = -0.78 to -0.22, R squared = 0.32, P value (two-tailed) = 0.003. Pearson correlation analysis, appropriate because the data points are independent and the expected relationship is linearly covariant, with x and y values recorded independently and sampled from populations that follow Gaussian distributions, gives a P value < 0.05 suggesting a correlation. The R squared value is low (R > 0.5 implies the model fits the data well). Analogue **28** appears to be anomalous.

S8 Separation of Enantiomers

S8.1 Supercritical fluid chromatography

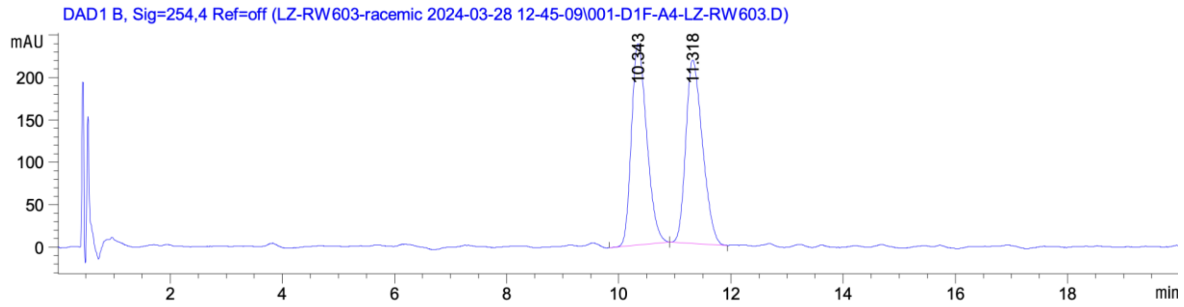

Figure S103 – Chiral LCMS spectrum for a racemic mixture of **19**. Peak 1 and 2 in buffer (20 mM HEPES pH 8, TCEP 0.5 mM, NaCl 100 mM).

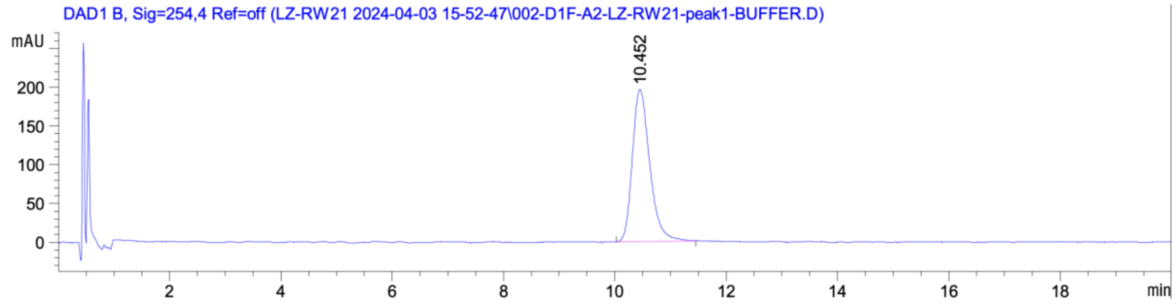

Figure S104 – Chiral LCMS spectrum for t = 0 hr, peak 1 in buffer (20 mM HEPES pH 8, TCEP 0.5 mM, NaCl 100 mM).

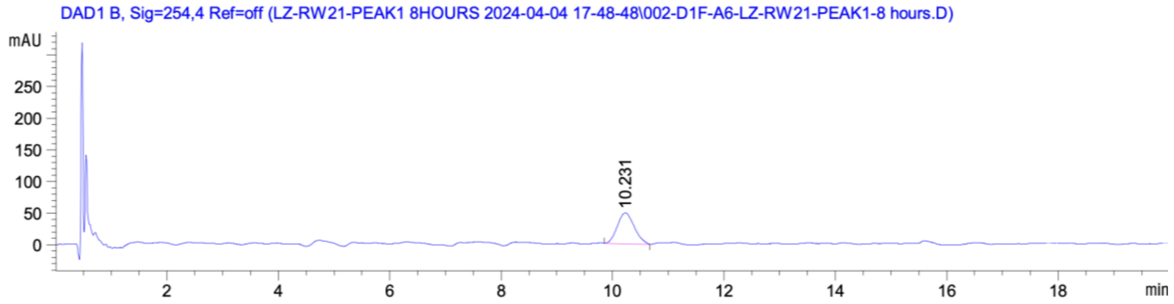

Figure S105 – Chiral LCMS spectrum for t = 8 hr, peak 1 in buffer (20 mM HEPES pH 8, TCEP 0.5 mM, NaCl 100 mM).

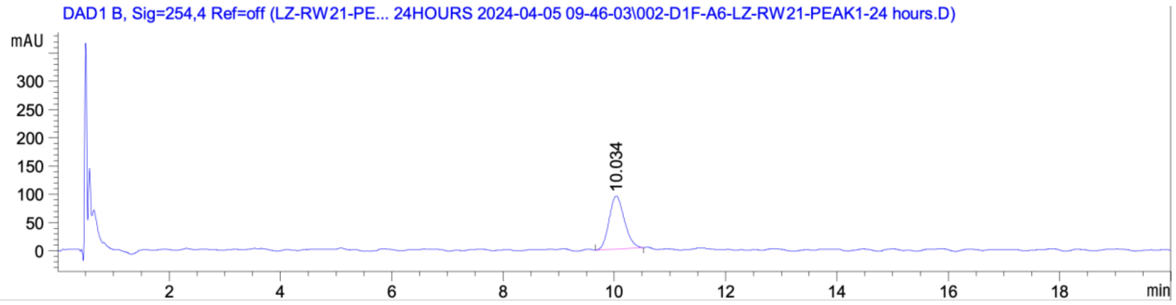

Figure S106 – Chiral LCMS spectrum for t = 24 hr, peak 1 in buffer (20 mM HEPES pH 8, TCEP 0.5 mM, NaCl 100 mM).

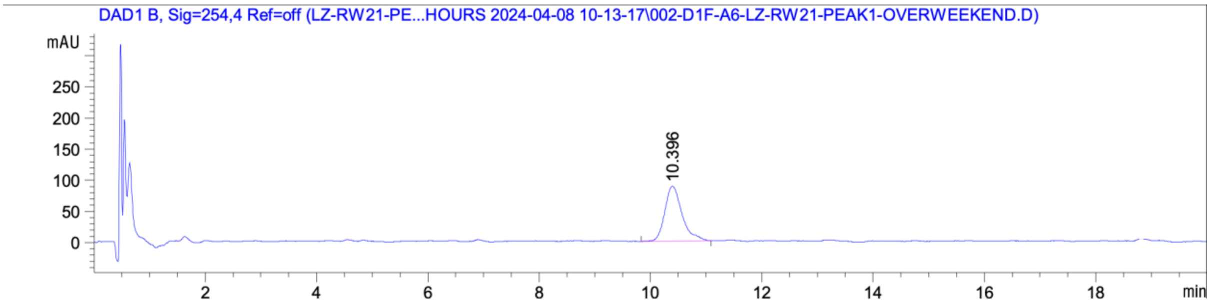

Figure S107 – Chiral LCMS spectrum for t = 96 hr, peak 1 in buffer (20 mM HEPES pH 8, TCEP 0.5 mM, NaCl 100 mM).

S9 Computational Work

S9.1 Mixed Solvent Molecular Dynamics

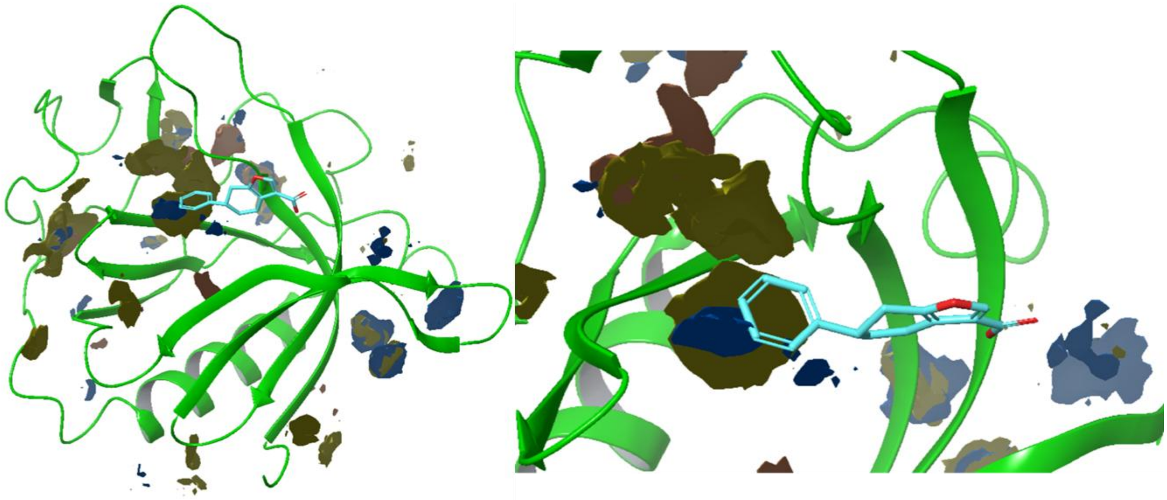

Figure S108 – Hot spot probing of the BTK PH domain with mixed solvent MD simulations. The colour of the pockets represents the type of co-solvent clustering: maroon – acetonitrile; yellow-brown – pyrimidine; blue – isopropanol.

S9.2 Virtual Screening

Virtual screening has been performed (using Schrödinger) in order to probe the pocket with various electronics, H-bonding potentials and degrees of hydrophobicity. R-group enumeration was used to enumerate a library of different substituents (halogens, alkyl and aromatic) on the three positions of the phenyl ring. The enumerated library of mono and di-substituted analogues (n = 192) was then used in virtual screening, with a cut-off for pose retainment of 2.5 kcal/mol. Given the rigorous cut-off, virtual screening yielded valid poses for 95 analogues with various mono and di-substitution patterns. Docking scores were then analysed in boxplots (Figure S109), in order to determine which substituents induce low and high docking scores. Covalent docking score analysis shows halogens in the *ortho* and *para* position are privileged scaffolds, inducing the most negative docking scores. Other emerging privileged groups are isopropoxy and methoxy. Phenoxy, phenyl and trifluoromethyl substituents emerge as destabilising across all three positions. The emerging trends were confirmed by MS and DSF, which highlighted the privileged nature of the halogens in *ortho* and *para* positions, as well as the detrimental effects of extra phenyl rings or trifluoromethyl.

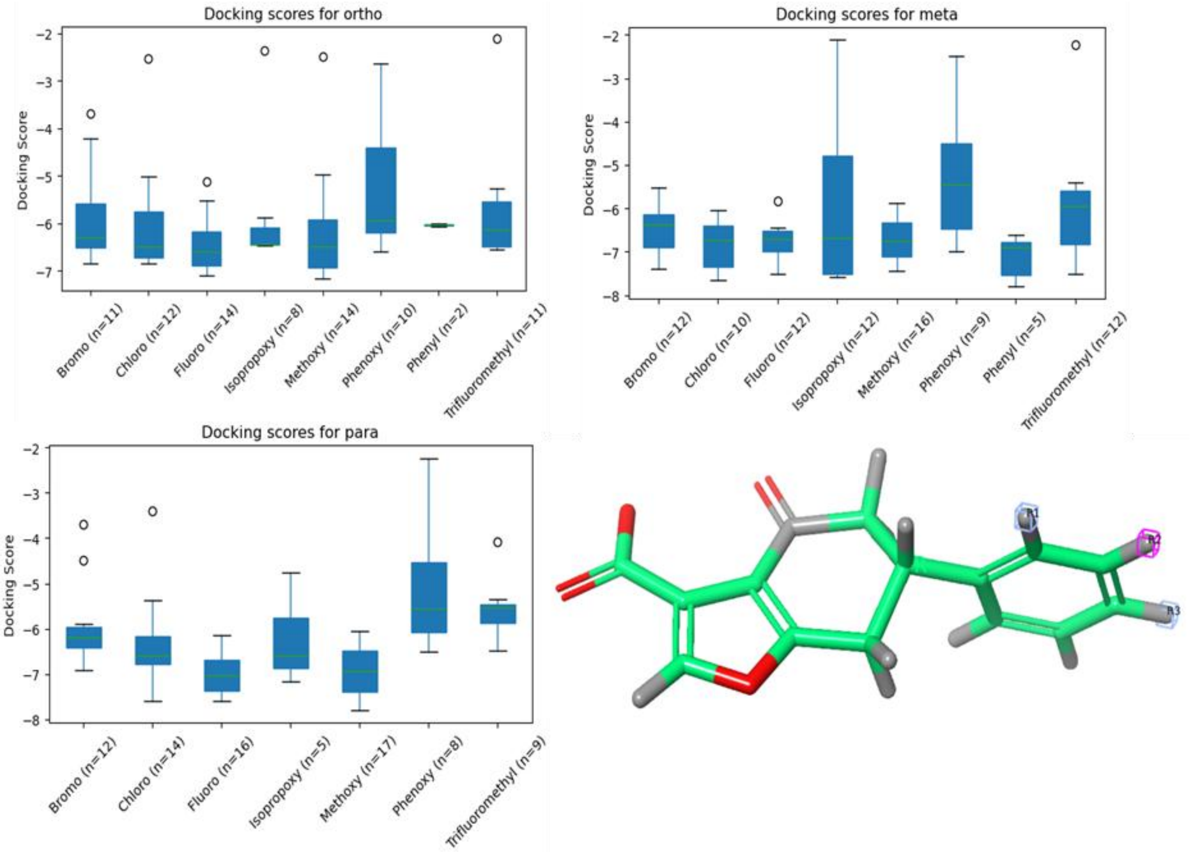

Figure S109 – Virtual screening results of the outcome of R group enumeration across the three positions of the phenyl ring. Docking was performed into PDB: 7I9I.

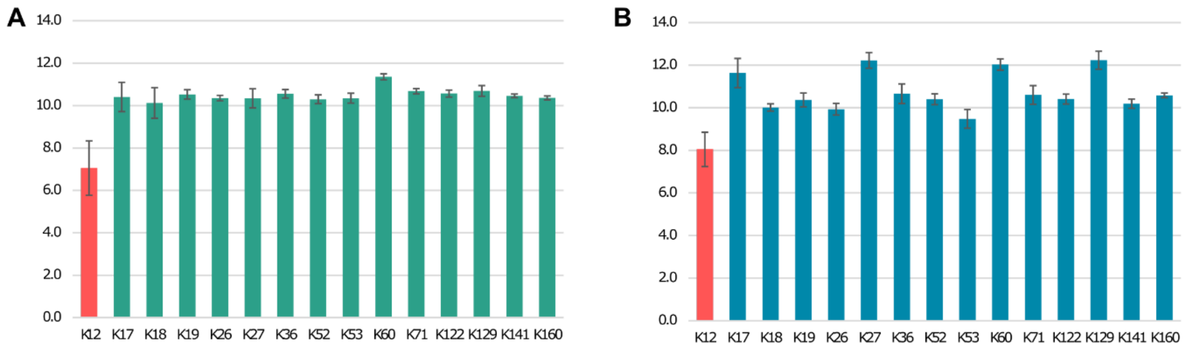

Figure S110. Calculated pKa values for all the lysines in Btk PH domain, calculated with DeepKa (A) and pypKa (B) servers using structures 1B55, 1BWN, 2Z0B and 4Y94 from PDB.

Table S10. Calculated pKa values for Btk K12 and equivalent lysine residues in other PH domains. The PDB coordinates used were 1B55, 1BWN, 2Z0B and 4Y94 for Btk, 1H10, 1UNQ and 2UZR for Akt, 1FGZ, 1FHW, 1FHX and 1FGY for GRP1, 1FAO and 1FB8 for DAPP1, 5D27, 5D3X, 5D3V and 5D3Y for p-Rex1 and 1mai for PLCδ. Number of domains in the table refers to total number of PH domains was used in calculations from all the structures. The pKa difference is calculated using the reference value for lysine amino group used in the two programmes, 10.40 and 10.46 for DeepKa and pypKa, respectively.

Method	Protein	Number of domains	Lys12 equivalent	Average pKa for Lys12	Average pKa difference	Number of other lysines per domain	Average pKa for other lysines
pypKa	Btk	12	K12	8.83 ± 0.80	-2.26	14	10.84 ± 0.79
DeepKa	Btk	12	K12	7.05 ± 1.29	-3.35	14	10.51 ± 0.97
DeepKa	Akt	3	K14	8.24 ± 0.87	-2.16	7	10.71 ± 0.33
DeepKa	GRP1	6	K273	7.32 ± 1.02	-3.19	10	10.46 ± 0.35
DeepKa	Dapp1	2	K173	7.82 ± 0.18	-2.59	8	10.54 ± 0.26
DeepKa	P-Rex1	7	K280	8.08 ± 0.62	-2.32	14	10.42 ±0.32
DeepKa	PLCδ	1	K30	9.42	-0.98	8	10.78 ±0.45
